# Comparative efficacy and tolerability of 32 oral antipsychotics for the acute treatment of adults with multi-episode schizophrenia: a systematic review and network meta-analysis

**DOI:** 10.1016/S0140-6736(19)31135-3

**Published:** 2019-09-14

**Authors:** Maximilian Huhn, Adriani Nikolakopoulou, Johannes Schneider-Thoma, Marc Krause, Myrto Samara, Natalie Peter, Thomas Arndt, Lio Bäckers, Philipp Rothe, Andrea Cipriani, John Davis, Georgia Salanti, Stefan Leucht

**Affiliations:** a Department of Psychiatry and Psychotherapy, Klinikum rechts der Isar, School of Medicine, Technical University of Munich, Munich, Germany; b Institute of Social and Preventive Medicine, University of Bern, Bern, Switzerland; c Institute for Evidence in Medicine (for Cochrane Germany Foundation), University Medical Center Freiburg, Faculty of Medicine, University of Freiburg, Freiburg im Breisgau, Germany; d Department of Forensic Psychiatry and Psychotherapy, University of Ulm, Günzburg District Hospital, Ulm, Germany; e Department of Psychiatry, University of Oxford and Oxford Health NHS Foundation Trust, Warneford Hospital, Oxford, UK; f Psychiatric Institute, University of Illinois at Chicago, Chicago, IL, USA; g Maryland Psychiatric Research Center, Baltimore, MD, USA

## Abstract

**Background:**

Schizophrenia is one of the most common, burdensome, and costly psychiatric disorders in adults worldwide. Antipsychotic drugs are its treatment of choice, but there is controversy about which agent should be used. We aimed to compare and rank antipsychotics by quantifying information from randomised controlled trials.

**Methods:**

We did a network meta-analysis of placebo-controlled and head-to-head randomised controlled trials and compared 32 antipsychotics. We searched Embase, MEDLINE, PsycINFO, PubMed, BIOSIS, Cochrane Central Register of Controlled Trials (CENTRAL), WHO International Clinical Trials Registry Platform, and ClinicalTrials.gov from database inception to Jan 8, 2019. Two authors independently selected studies and extracted data. We included randomised controlled trials in adults with acute symptoms of schizophrenia or related disorders. We excluded studies in patients with treatment resistance, first episode, predominant negative or depressive symptoms, concomitant medical illnesses, and relapse-prevention studies. Our primary outcome was change in overall symptoms measured with standardised rating scales. We also extracted data for eight efficacy and eight safety outcomes. Differences in the findings of the studies were explored in metaregressions and sensitivity analyses. Effect size measures were standardised mean differences, mean differences, or risk ratios with 95% credible intervals (CrIs). Confidence in the evidence was assessed using CINeMA (Confidence in Network Meta-Analysis). The study protocol is registered with PROSPERO, number CRD42014014919.

**Findings:**

We identified 54 417 citations and included 402 studies with data for 53 463 participants. Effect size estimates suggested all antipsychotics reduced overall symptoms more than placebo (although not statistically significant for six drugs), with standardised mean differences ranging from −0·89 (95% CrI −1·08 to −0·71) for clozapine to −0·03 (−0·59 to 0·52) for levomepromazine (40 815 participants). Standardised mean differences compared with placebo for reduction of positive symptoms (31 179 participants) varied from −0·69 (95% CrI −0·86 to −0·52) for amisulpride to −0·17 (−0·31 to −0·04) for brexpiprazole, for negative symptoms (32 015 participants) from −0·62 (−0·84 to −0·39; clozapine) to −0·10 (−0·45 to 0·25; flupentixol), for depressive symptoms (19 683 participants) from −0·90 (−1·36 to −0·44; sulpiride) to 0·04 (−0·39 to 0·47; flupentixol). Risk ratios compared with placebo for all-cause discontinuation (42 672 participants) ranged from 0·52 (0·12 to 0·95; clopenthixol) to 1·15 (0·36 to 1·47; pimozide), for sedation (30 770 participants) from 0·92 (0·17 to 2·03; pimozide) to 10·20 (4·72 to 29·41; zuclopenthixol), for use of antiparkinson medication (24 911 participants) from 0·46 (0·19 to 0·88; clozapine) to 6·14 (4·81 to 6·55; pimozide). Mean differences compared to placebo for weight gain (28 317 participants) ranged from −0·16 kg (−0·73 to 0·40; ziprasidone) to 3·21 kg (2·10 to 4·31; zotepine), for prolactin elevation (21 569 participants) from −77·05 ng/mL (−120·23 to −33·54; clozapine) to 48·51 ng/mL (43·52 to 53·51; paliperidone) and for QTc prolongation (15 467 participants) from −2·21 ms (−4·54 to 0·15; lurasidone) to 23·90 ms (20·56 to 27·33; sertindole). Conclusions for the primary outcome did not substantially change after adjusting for possible effect moderators or in sensitivity analyses (eg, when excluding placebo-controlled studies). The confidence in evidence was often low or very low.

**Interpretation:**

There are some efficacy differences between antipsychotics, but most of them are gradual rather than discrete. Differences in side-effects are more marked. These findings will aid clinicians in balancing risks versus benefits of those drugs available in their countries. They should consider the importance of each outcome, the patients' medical problems, and preferences.

**Funding:**

German Ministry of Education and Research and National Institute for Health Research

## Introduction

Schizophrenia is a common debilitating disorder (1% of the world population are afflicted) with a huge burden for patients, which cost the USA US$155·7 billion in 2013.^1^, ^2^ Antipsychotics are the mainstay of treatment,^3^ but are associated with important side-effects that can cause serious disability or death.^4^ According to WHO,^5^ many drugs are available that vary considerably in their affinity to different synaptic receptors, leading to possible diverging efficacy and safety profiles. Many guidelines recommend newer antipsychotics as the treatment of choice, but older antipsychotics are less costly and are still used worldwide, especially in low-income countries.^6^ Moreover, neither these older antipsychotics nor the more recently approved brexpiprazole and cariprazine have been compared in a comprehensive network meta-analysis.^7^ A clear understanding of the relative risks and benefits is essential for informed decision making. This systematic review and meta-analysis extends our previous work^3^ that combined evidence from 212 randomised trials on 15 antipsychotics and seven outcomes to 402 randomised trials on 32 antipsychotics and placebo and 17 efficacy and safety outcomes, with change in overall symptoms as the primary outcome. We aimed to better inform clinical practice and mental health policies by comparing all licensed second-generation and 17 first-generation antipsychotics in the acute treatment of adults with schizophrenia.

**Research in context**

**Evidence before this study**

Treatment with antipsychotic drugs is the standard for the acute phase of schizophrenia according to most national and international guidelines. Nevertheless, antipsychotic use is debated strongly owing to side-effects and possible brain changes. Because many antipsychotic drugs are available, according to WHO, understanding how the many substances compare with each other is important, ideally ranked in a hierarchy. Due to their diverging receptor binding profiles, different antipsychotics can vary considerably in their efficacy and side-effect profiles. Even with a large evidence base of randomised clinical trials for acute treatment of schizophrenia, many evidence gaps remain, because many substances have never been compared directly in trials, especially older antipsychotics. We searched PubMed for network meta-analyses on the acute treatment of schizophrenic patients with antipsychotics published between database inception and Oct 5, 2018. Using the search terms “antipsychotic” AND “schizophrenia” AND (“network meta-analysis” OR “multiple treatment meta-analysis”), we found several relevant systematic reviews. Some examined specific subgroups, such as children or first-episode patients, whereas others were restricted to specific populations, such as Chinese or Japanese people. Some focused on special outcomes like weight gain or glucose. One network meta-analysis from 2013 did not include the newly approved antipsychotics cariprazine and brexpiprazole and examined only the two older antipsychotics haloperidol and chlorpromazine. Furthermore, not all clinically important efficacy and safety outcomes were studied in this report. Altogether we found no comprehensive and systematic network meta-analysis comparing older and newer antipsychotics for the acute treatment of schizophrenia over several efficacy and safety outcomes.

**Added value of this study**

To our knowledge, this analysis is the largest network meta-analysis done in the field of schizophrenia. It was based on 402 studies including 53 463 participants randomly assigned to 32 different older and newer antipsychotics or placebo. The addition of two new and 15 old antipsychotics is a major extension of a previous report by our group. We investigated ten additional important outcomes, such as specific aspects of efficacy, quality of life, and many more side-effects, and several methodological issues, such as placebo response and sample sizes. The primary outcome was reduction of overall schizophrenic symptoms, but we also examined other domains: reduction in positive and negative symptoms, dropouts, depression, quality of life, and functioning. Newer and older antipsychotics reduced overall symptoms more than placebo and had lower all-cause discontinuation rates than placebo. Differences in side-effects between drugs were often large. Because smaller amounts of data were available for older antipsychotics, comparisons involving them were often more uncertain and their evidence of lower quality. Conclusions for the primary outcome did not substantially change after adjusting for possible effect moderators or in sensitivity analyses (eg, when excluding placebo-controlled studies).

**Implications of all the available evidence**

These findings provide treatment rankings for older and newer antipsychotics for 17 different outcomes, which should inform the decision making process and clinical guidelines internationally.

## Methods

### Search strategy and selection criteria

We did a systematic review and network meta-analysis of placebo-controlled and head-to-head randomised controlled trials (RCTs) according to PRISMA guidelines.^8^ We included RCTs in adults with acute symptoms of schizophrenia or related disorders (such as schizophreniform or schizoaffective disorders). We excluded studies in patients with treatment resistance, first episode, predominant negative or depressive symptoms, concomitant medical illnesses, and relapse-prevention studies.

We included all second-generation (atypical) antipsychotics available in Europe or the USA, placebo and a selection of first-generation (typical or conventional) antipsychotics (benperidol, chlorpromazine, clopenthixol [cis-isomer and trans-isomer], flupentixol, fluphenazine, haloperidol, levomepromazine, loxapine, molindone, penfluridol, perazine, perphenazine, pimozide, sulpiride, thioridazine, thiotixene, trifluoperazine, and zuclopenthixol [cis-isomer]) guided by a survey of 50 international schizophrenia experts.^9^ We excluded intramuscular formulations because they are primarily used for relapse prevention (long-acting) or emergency use (short-acting). We included all flexible-dose studies because they allow the investigators to titrate to optimum dose for the individual patient. In fixed-dose studies, we included target-to-maximum doses according to the International Consensus Study on Antipsychotic Dose.^10^ If studies used several doses, we averaged the results of the individual groups using weighted means.^11^

We included published and unpublished RCTs comparing one antipsychotic with another or with placebo. Trials in which antipsychotics were used as an augmentation or combination strategy were excluded. For subjective outcomes (eg, overall change in symptoms), we included only double-blind studies, because an absence of blinding can exaggerate differences between treatments in this area.^12^ For objective outcomes, open studies were included (appendix pp 271, 272). We included short-term studies with a follow-up period of 3–13 weeks.^13^ Studies with a high risk of bias in sequence generation or allocation concealment according to the Cochrane Collaboration's risk of bias tool were excluded.^11^ We a-priori excluded studies from mainland China owing to quality concerns.^14^ We searched MEDLINE, Cochrane Central Register of Controlled Trials (CENTRAL), Embase, Biosis, PsycINFO, PubMed, ClinicalTrials.gov, WHO International Clinical Trials Registry Platform and the US Food and Drug Administration website without language restrictions from database inception until Jan 8, 2019. The search strategy combined terms for schizophrenia or psychosis and various drug names (appendix pp 25–48). Reference lists of the included studies and previous reviews were screened for additional studies.

At least two reviewers (MH, MK, MS, and Yikang Zhu [Shanghai Mental Health Center]) screened the search results independently, retrieved full-text articles, and checked inclusion criteria. In case of doubt a third reviewer (SL) was involved. Two reviewers independently extracted data and entered them in electronic forms in Microsoft Access 2010 (MH, JS-T, MK, MS, NP, TA, LB, PR, Yikang Zhu, Matteo Rabaioli-Fischer, Susanne Bächer, Leonie Reichelt, and Hannah Röder [Technical University of Munich]). An algorithm checked for conflicting data entries. Differences were discussed, and a third reviewer (SL) was contacted if consensus was not reached. Study authors were contacted in case of missing or unclear information. For dichotomous data we assumed that participants lost to follow-up would not have responded. Missing standard deviations were estimated from test statistics or by using the mean standard deviation of the remaining studies.^15^ Risk of bias in RCTs for the primary outcome was assessed independently using the Cochrane Collaboration's risk of bias tool (appendix pp 273–77).^11^ The overall risk of bias was classified into high, moderate, or low as proposed in a large network meta-analysis for antidepressants.^16^

### Outcomes

The primary outcome was change in overall symptoms of schizophrenia as measured by rating scales, such as the Positive and Negative Syndrome Scale, the Brief Psychiatric Rating Scale, or any other published scale.^17^ Secondary outcomes were all-cause discontinuation, discontinuation due to inefficacy and responder rates (study defined), as well as change in positive, negative, and depressive symptoms, quality of life, and social functioning, measured by means of published rating scales. The following major side effects were examined: use of antiparkinson drugs as a measure of extrapyramidal side-effects, akathisia, weight gain in kg, 7% weight gain or more, prolactin levels, sedation or somnolence, QTc prolongation, and at least one anticholinergic side-effect (appendix pp 271, 272).

### Data analysis

We did a network meta-analysis combining direct and indirect comparisons in a Bayesian hierarchical model using the rjags package (appendix pp 49–52).^18^ Effect sizes were risk ratios for dichotomous outcomes and standardised mean differences (SMDs) for efficacy related continuous outcomes, because different rating scales were used. Mean differences were applied for weight gain, QTc prolongation, and prolactin elevation for clinically palpable results. Data were combined using a random-effects model. Treatments were ranked using the surface under the curve cumulative ranking probabilities. The transitivity assumption was evaluated by comparing the distribution of potential effect modifiers (placebo response, publication year, sample size, baseline severity, mean age, and percentage male) across studies grouped by comparison (appendix pp 54–61). We assumed a common heterogeneity parameter across the various treatment comparisons and presented the between-study variance τ^2^ for each outcome. We characterised the amount of heterogeneity as low, moderate, or high using the first and third quantiles of their empirical distributions.^19^ We evaluated consistency statistically (the agreement of the various sources of evidence) using the design-by-treatment test^20^ and by separating indirect from direct evidence (SIDE test)^21^ using the R netmeta package (appendix pp 223, 224).^22^ We explored residual heterogeneity and inconsistency by several a-priori defined metaregressions (with covariates: placebo response rate, study sample size, study publication year, baseline severity, sponsorship, mean age, and percentage male) and sensitivity analyses (excluding studies at overall high risk of bias, that did a completer analysis, with imputed standard deviations, that were placebo-controlled, with duration more than 6 weeks, that were published before 1990, that were considered failed trials, and with unfair dose comparison, and excluding placebo arms; appendix pp 49–52, 268–70). We used contour-enhanced funnel plots and the trim-and-fill method for the primary outcome to investigate the presence of small-study effects, whereby small studies give different results from the large studies for all comparisons against placebo and against haloperidol.^23^, ^24^

The certainty of evidence produced by the synthesis for each outcome was evaluated using the framework described by Salanti and colleagues^25^ and implemented using the CINeMA (Confidence in Network Meta-Analysis) web application which allows confidence in the results to be graded as high, moderate, low, and very low (appendix pp 238–67).^26^ For the primary outcome we examined the confidence of evidence of all comparisons. For the remaining outcomes we examined the comparisons of antipsychotics versus placebo. The protocol is registered with PROSPERO, number CRD42014014919 (appendix pp 8–24).

### Role of the funding source

The funder of the study had no role in study design, data collection, data analysis, data interpretation, or writing of the report. The corresponding author had full access to all the data in the study and had final responsibility for the decision to submit for publication.

## Results

The search identified 54 417 citations, including 22 074 unique reports, and 2827 full-text articles were retrieved after the exclusion of 19 247 reports on the basis of their titles and abstracts. We screened these 2827 full-text articles and included 550 reports from 402 studies with 53 463 participants (appendix pp 62–154). The sample had the following characteristics: mean age was 37·40 years (SD 5·96), 29 949 (56·02%) participants were male and 23 514 (43·98%) female, and mean illness duration was 11·90 years (SD 5·19; appendix pp 282–84). We excluded studies with high risk of bias for randomisation and allocation, but methods for sequence generation and allocation concealment were often not described in detail and, therefore, were coded as unclear (appendix pp 155–76). The percentage of studies with high, unclear, and low risk of bias for the individual items was: 0%, 73·1%, and 26·9% for randomisation, 0%, 78·4%, and 21·6% for allocation concealment, 10·7%, 38·6%, and 50·7% for blinding of patients and personnel, 13·9%, 34·8%, and 51·3% for rater blinding, 23·9%, 35·6%, and 40·5% for missing outcomes, 27·4%, 20·9%, and 51·7% for selective reporting, and 5·7%, 11·9%, and 82·3% for other biases. The overall risk of bias was rated as high for 92 (23%) studies.

**Figure 1:**
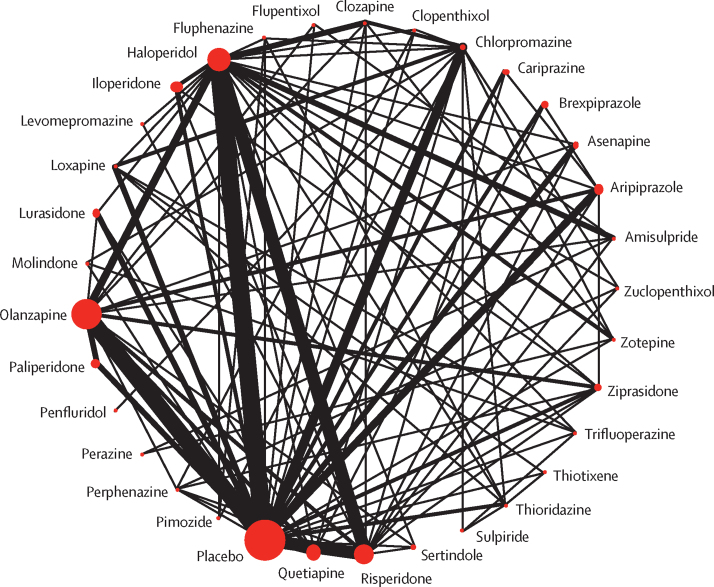
Network plot of overall efficacy The size of the nodes corresponds to the number of participants assigned to each treatment. Treatments with direct comparisons are linked with a line; its thickness corresponds to the number of trials evaluating the comparison.

**Figure 2:**
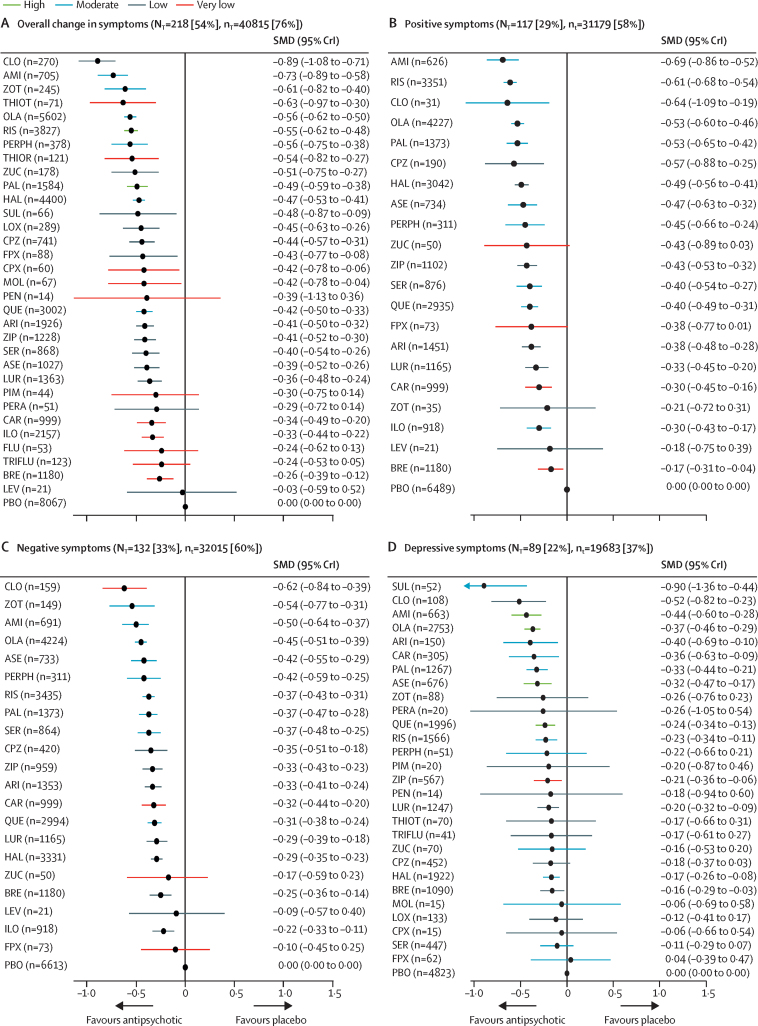

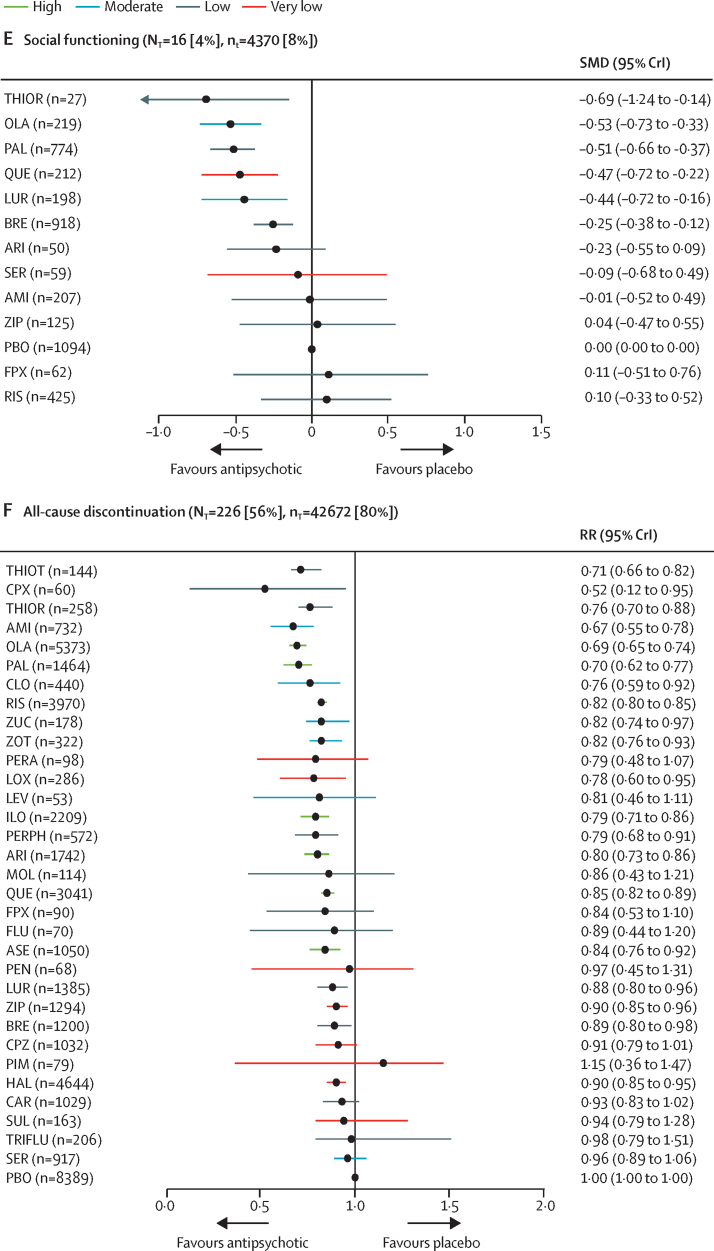
Change in efficacy outcomes (A) Overall change in symptoms. (B) Positive symptoms. (C) Negative symptoms. (D) Depressive symptoms. (E) Social functioning. (F) All-cause discontinuation. Treatments are ranked according to their surface under the curve cumulative ranking and compared with placebo. Effect sizes are presented as standardised mean difference or risk ratio with 95% CrIs. The evidence is graded using the CINeMA system (Confidence in Network Meta-Analysis), an adaption of the GRADE (Grading of Recommendations, Assessment, Development, and Evaluations) approach for network meta-analysis. Colours indicate the confidence in the evidence: green=high, blue=moderate, grey=low, red=very low. N_T_=total number of trials reporting the outcome (percentage of sample). n_T_=total number of participants available for the respective outcome (percentage of sample). SMD=standardised mean difference. CrI=credible interval. RR=risk ratio. AMI=amisulpride. ARI=aripiprazole. ASE=asenapine. BRE=brexpiprazole. CAR=cariprazine. CLO=clozapine. CPX=clopenthixol. CPZ=chlorpromazine. FLU=fluphenazine. FPX=flupentixol. HAL=haloperidol. ILO=iloperidone. LEV=levomepromazine. LOX=loxapine. LUR=lurasidone. MOL=molindone. OLA=olanzapine. PAL=paliperidone. PBO=placebo. PEN=penfluridol. PERA=perazine. PERPH=perphenazine. PIM=pimozide. QUE=quetiapine. RIS=risperidone. SER=sertindole. SUL=sulpiride. THIOR=thioridazine. THIOT=thiotixene. TRIFLU=trifluoperazine. ZIP=ziprasidone. ZOT=zotepine. ZUC=zuclopenthixol.

**Figure 3:**
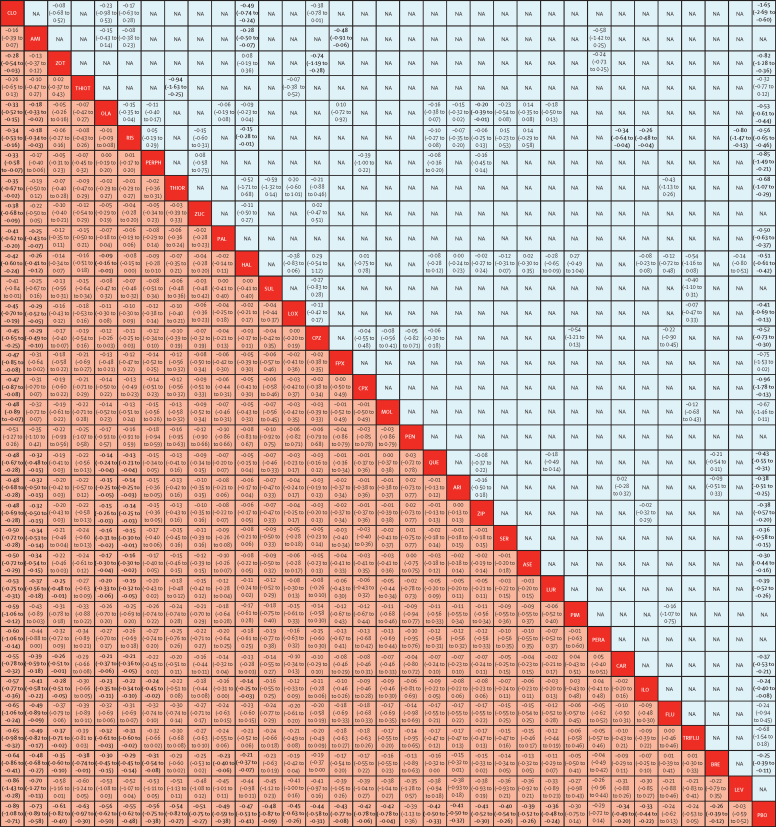
Change in overall symptoms league table Antipsychotics are reported in order of surface under the curve cumulative ranking. Results of the network meta-analysis are presented in the left lower half and results from pairwise meta-analysis in the upper right half, if available. Comparisons between treatments should be read from left to right and the estimate is in the cell in common between the column-defining treatment and the row-defining treatment. In the left lower half, standard mean differences lower than 0 favour the column-defining treatment and in the upper right half, those lower than 0 favour the row-defining treatment. Cells in bold print indicate significant results. NA=not available. AMI=amisulpride. ARI=aripiprazole. ASE=asenapine. BRE=brexpiprazole. CAR=cariprazine. CLO=clozapine. CPX=clopenthixol. CPZ=chlorpromazine. FLU=fluphenazine. FPX=flupentixol. HAL=haloperidol. ILO=iloperidone. LEV=levomepromazine. LOX=loxapine. LUR=lurasidone. MOL=molindone. OLA=olanzapine. PAL=paliperidone. PBO=placebo. PEN=penfluridol. PERA=perazine. PERPH=perphenazine. PIM=pimozide. QUE=quetiapine. RIS=risperidone. SER=sertindole. SUL=sulpiride. THIOR=thioridazine. THIOT=thiotixene. TRIFLU=trifluoperazine. ZIP=ziprasidone. ZOT=zotepine. ZUC=zuclopenthixol.

218 (54%) studies with 40 815 (76%) participants presented usable results for change in overall symptoms (figure 1). 26 (81%) of 32 antipsychotics were associated with significant improvement in symptoms compared with placebo (figure 2A). The SMDs for drugs associated with significant improvement ranged between −0·89 (95% credible interval [CrI] −1·08 to −0·71) for clozapine to −0·26 (−0·39 to −0·12) for brexpiprazole. Clozapine, amisulpride, zotepine, olanzapine, and risperidone reduced overall symptoms significantly more than many other drugs (figure 3). Most differences between the remaining drugs were small or very uncertain.

Secondary efficacy outcomes were reported less frequently especially for older drugs including clozapine (appendix pp 200–21). 117 studies (29%) with 31 179 participants (58%) presented results usable for reduction of positive symptoms (21 antipsychotics). The SMDs for the 17 (81%) drugs that significantly reduced positive symptoms compared with placebo ranged between −0·69 (95% CrI −0·86 to −0·52) for amisulpride to −0·17 (−0·31 to −0·04) for brexpiprazole (figure 2B). Amisulpride, risperidone, olanzapine, paliperidone, and haloperidol were significantly more effective than many other drugs (appendix pp 200–21).

132 studies (33%) with 32 015 (60%) participants reported usable results for negative symptoms (21 antipsychotics). The SMDs for the 18 (86%) antipsychotics that significantly reduced negative symptoms compared with placebo ranged between −0·62 (95% CrI −0·84 to −0·39) for clozapine to −0·22 (−0·33 to −0·11) for iloperidone (figure 2C). Clozapine, amisulpride, olanzapine, and, to a lesser extent, zotepine and risperidone reduced negative symptoms significantly more than many other drugs. Differences between the remaining drugs were uncertain (appendix pp 200–21).

89 studies (22%) with 19 683 participants (37%) reported usable results for depressive symptoms (28 antipsychotics). The SMDs for the 14 (50%) drugs that significantly reduced depressive symptoms compared with placebo ranged between −0·90 (95% CrI −1·36 to −0·44) for sulpiride and −0·16 (−0·29 to −0·03) for brexpiprazole (figure 2D). Sulpiride, clozapine, amisulpride, and olanzapine were associated with significantly more reduction of depressive symptoms compared with many other drugs (appendix pp 200–21), but CrIs were wide.

Only ten studies (3%) with 3341 participants (6%) reported usable quality-of-life data (eight antipsychotics). Because 50% of the network loops were inconsistent, we did a pairwise meta-analysis. Compared with placebo, five antipsychotics significantly improved quality of life, with SMDs ranging from −0·49 (95% CI −0·72 to −0·26) for aripiprazole to −0·18 (−0·34 to −0·02) for paliperidone (appendix p 222).

16 studies (4%) with 4370 participants (8%) presented usable results for social functioning (12 antipsychotics). Based on a small number of studies, thioridazine, olanzapine, paliperidone, quetiapine, lurasidone, and brexpiprazole were associated with significant improvement in social functioning compared with placebo with a SMD range from −0·69 (95% CrI −1·24 to −0·14) for thioridazine to −0·25 (−0·38 to −0·12) for brexpiprazole (figure 2E).

192 studies with 35 115 participants reported study-defined response rates, using very different cutoffs (appendix p 278). 29 (94%) of 31 antipsychotics had significantly higher response rates compared with placebo, with risk ratios ranging from 2·16 (95% CrI 1·53–3·55) for thioridazine to 1·11 (1·01–1·19) for brexpiprazole (appendix p 278).

226 (56%) studies reported all-cause discontinuation rates for 42 672 (80%) participants (32 antipsychotics). Risk ratios for the 20 drugs (63%) that significantly lowered discontinuation rates compared with placebo ranged from 0·52 (95% CrI 0·12–0·95) for clopenthixol to 0·90 (0·85–0·95) for haloperidol (figure 2F). When examining discontinuation due to inefficacy we found comparable results as for the primary outcome overall change in symptoms (appendix p 279).

**Figure 4:**
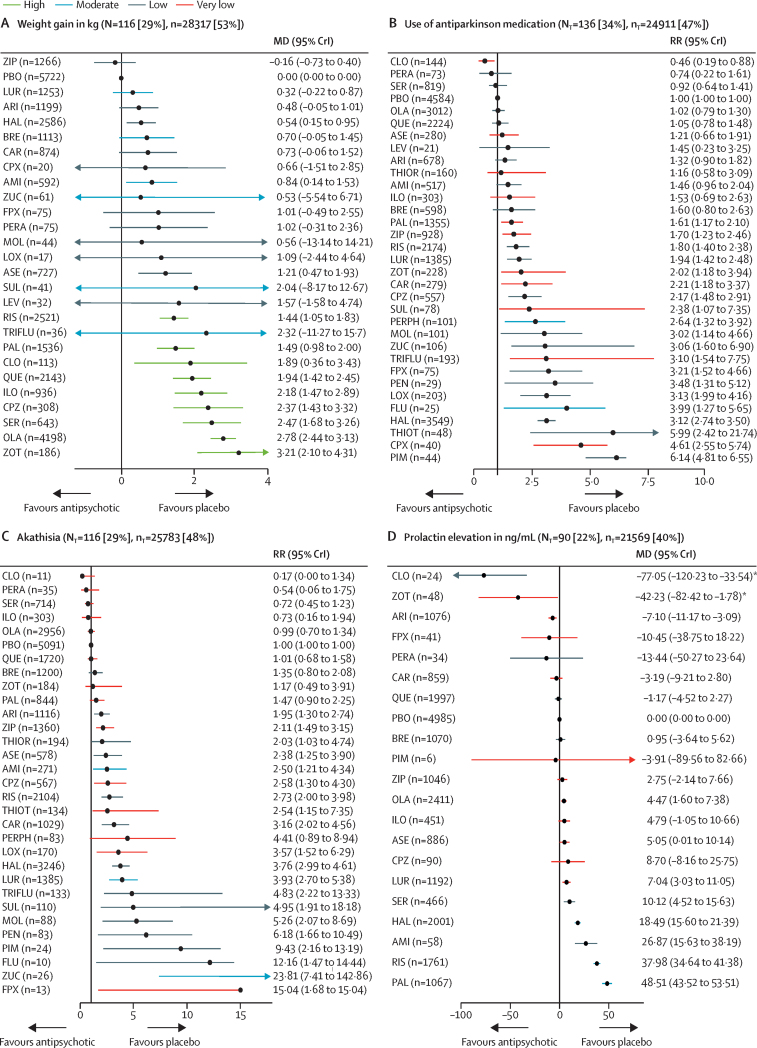

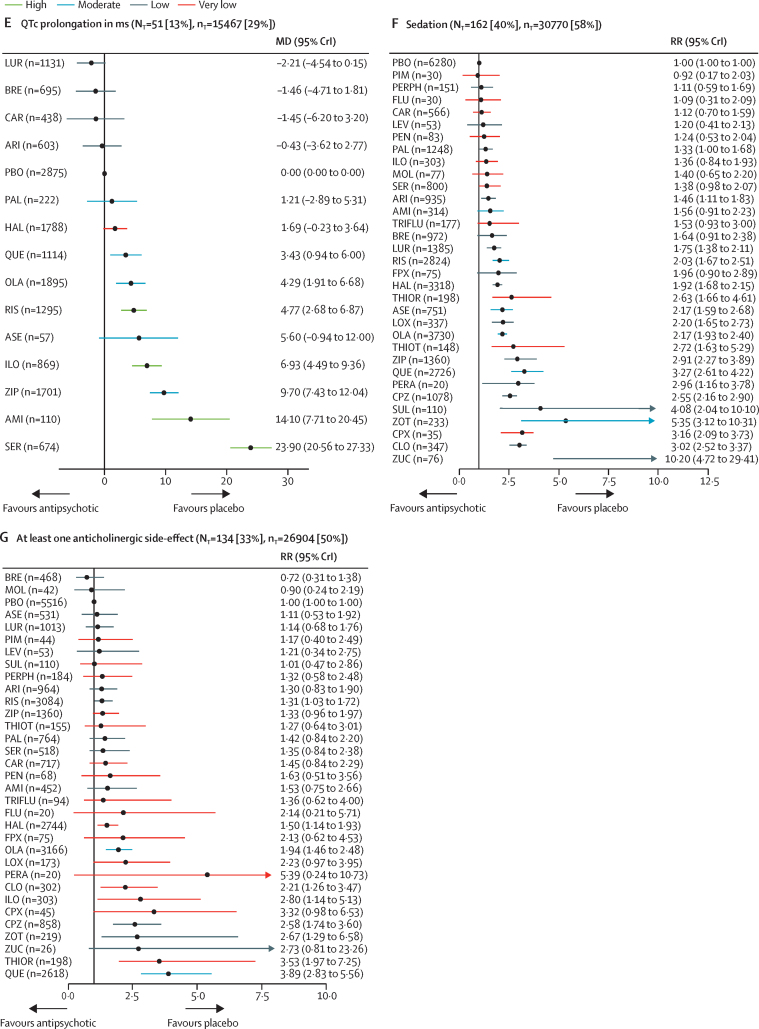
Change in side-effect outcomes (A) Weight gain in kg. (B) Use of antiparkinson medication. (C) Akathisia. (D) Prolactin elevation in ng/mL. (E) QTc prolongation in ms. (F) Sedation. (G) At least one anticholinergic side-effect. Treatments are ranked according to their surface under the curve cumulative ranking and compared with placebo. Effect sizes are presented as mean difference or risk ratio with 95% CrIs. The evidence is graded using CINeMA system (Confidence in Network Meta-Analysis), an adaption of the GRADE (Grading of Recommendations, Assessment, Development, and Evaluations) approach for network meta-analysis. Colours indicate the confidence in the evidence: green=high, blue=moderate, grey=low, red=very low. N_T_=total number of trials reporting the outcome (percentage of sample). n_T_=total number of participants available for the respective outcome (percentage of sample). MD=Mean difference. CrI=credible interval. RR=risk ratio. AMI=amisulpride. ARI=aripiprazole. ASE=asenapine. BRE=brexpiprazole. CAR=cariprazine. CLO=clozapine. CPX=clopenthixol. CPZ=chlorpromazine. FLU=fluphenazine. FPX=flupentixol. HAL=haloperidol. ILO=iloperidone. LEV=levomepromazine. LOX=loxapine. LUR=lurasidone. MOL=molindone. OLA=olanzapine. PAL=paliperidone. PBO=placebo. PEN=penfluridol. PERA=perazine. PERPH=perphenazine. PIM=pimozide. QUE=quetiapine. RIS=risperidone. SER=sertindole. SUL=sulpiride. THIOR=thioridazine. THIOT=thiotixene. TRIFLU=trifluoperazine. ZIP=ziprasidone. ZOT=zotepine. ZUC=zuclopenthixol. *Results for clozapine and zotepine might be statistical artifacts caused by two small outlier studies.

116 studies (29%) with 28 317 (53%) participants presented usable results for weight gain. 12 (46%) of 26 antipsychotics caused significantly more weight gain than placebo with mean differences ranging from 0·54 kg (95% CrI 0·15–0·95) for haloperidol to 3·21 kg (2·10–4·31) for zotepine (figure 4A). Zotepine, olanzapine, and sertindole produced significantly more weight gain than most other drugs (appendix pp 200–21). The hierarchy for patients with at least 7% weight gain was similar, confirming the findings (appendix p 280).

136 studies (34%) with 24 911 (47%) participants reported use of antiparkinson medication. Risk ratios for the 21 (66%) of 32 antipsychotics that were associated with significantly increased use of antiparkinson medication compared with placebo ranged from 1·61 (95% CrI 1·17–2·10) for paliperidone to 6·14 (4·81–6·55) for pimozide (figure 4B). The following drugs were significantly better than haloperidol starting with the best: clozapine, perazine, sertindole, placebo, olanzapine, quetiapine, asenapine, aripiprazole, thioridazine, amisulpride, iloperidone, brexpiprazole, paliperidone, ziprasidone, risperidone, lurasidone, zotepine, and chlorpromazine (appendix pp 200–21). 116 studies (29%) with 25 783 (48%) participants reported results for akathisia. The hierarchy was similar to use of antiparkinson medication, with significant risk ratios for 20 (67%) of 30 drugs ranging from 1·95 (95% CrI 1·30–2·74) for aripiprazole to 23·81 (7·41–142·86) for zuclopenthixol (figure 4C).

90 studies (22%) with 21 569 participants (40%) reported usable results for prolactin. Olanzapine, asenapine, lurasidone, sertindole, haloperidol, amisulpride, risperidone, and paliperidone were associated with significantly elevated prolactin levels (mean difference range 4·47–48·51 ng/mL). For many antipsychotics (eg, sulpiride) prolactin data were not available (figure 4D).

51 studies (13%) with 15 467 participants (29%) reported usable data for QTc prolongation. Seven (50%) of 14 antipsychotics caused significantly more QTc prolongation than placebo with mean differences ranging from 3·43 ms (95% CrI 0·94–6·00) for quetiapine to 23·90 ms (95% CrI 20·56–27·33) for sertindole (figure 4E).

162 studies (40%) with 30 770 participants (58%) reported results for sedation (32 antipsychotics). Risk ratios for the 18 drugs (56%) that were significantly more sedating than placebo ranged from 1·33 (95% CrI 1·00–1·68) for paliperidone to 10·20 (95% CrI 4·72–29·41) for zuclopenthixol, and there was some evidence of sedation for most of the remaining antipsychotics (figure 4F).

134 studies (33%) with 26 904 participants (50%) reported anticholinergic side-effects (32 antipsychotics). This outcome can be affected by use of anticholinergic medication, which is often needed for the treatment of extrapyramidal side-effects. Evidence for significantly higher risk than placebo was present for risperidone, haloperidol, olanzapine, clozapine, iloperidone, chlorpromazine, zotepine, thioridazine, and quetiapine (risk ratio range 1·31–3·89; figure 4G).

Heterogeneity was low to moderate for most outcomes, moderate to high for use of antiparkinson medication, and high for prolactin elevation. SIDE testing showed that the percentage of comparisons with evidence of inconsistency was 2–26% for all outcomes, except for quality of life with 50% comparisons with evidence of inconsistency; therefore, this outcome was examined in a pairwise meta-analysis (appendix p 222). Additionally, prolactin results were significantly inconsistent according to the design-by-treatment interaction test. Because prolactin values vary widely between men and women and assays used in different laboratories, we also applied SMDs, and heterogeneity and inconsistency were substantially lower (appendix pp 223, 224, 281).

The most important differences in terms of study characteristics were that older antipsychotics had less placebo response than newer ones and that the antipsychotics differed in their median baseline severity across studies (appendix pp 53–61). These potential threats to the transitivity assumption and other potential effect modifiers were addressed by metaregressions and sensitivity analyses of the primary outcome, excluding antipsychotics studied in less than 100 participants. The degree of placebo response, which has increased in the past 60 years,^27^ had the greatest effect on heterogeneity. The effect sizes of the individual antipsychotics changed after accounting for response to placebo, but the overall hierarchy did not (appendix pp 177–89). This finding was corroborated by removing placebo groups or placebo-controlled studies in sensitivity analyses (appendix pp 190–99). Publication year, mean participants' age, baseline severity, percentage of male patients, sample size, and sponsoring also did not affect the hierarchy of relative treatment effects compared with the unadjusted analysis (appendix pp 177–89). Sensitivity analyses removing studies with overall high risk of bias, completer analyses, imputed standard deviations, duration more than six weeks, and unfair dose comparisons, failed trials, and trials done before 1990 did not affect the results (appendix pp 190–99).

The certainty of the evidence was low overall (appendix p 242). Concerning the primary outcome, we judged the confidence in the evidence for 75% of the comparisons with placebo to be low or very low (figure 2A), and this was the case for 92% of the comparisons of two antipsychotic drugs (appendix p 242). Many older antipsychotics are among those with poor CINeMA ratings and often have no evidence for several secondary outcomes.

Comparison of the change in overall symptoms of all antipsychotics with haloperidol by use of a contour-enhanced funnel plot did not reveal any asymmetry and the SMD did not change using the trim-and-fill method (appendix p 285). By contrast, comparison of all antipsychotics with placebo revealed that smaller trials exaggerate the effectiveness of the active interventions versus placebo. SMD changed from 0·45 to 0·38, confirming an earlier analysis.^27^

## Discussion

To our knowledge, this analysis is the largest network meta-analysis in the field of schizophrenia, based on 402 studies including 53 463 participants randomly assigned to 32 different first-generation and second-generation antipsychotics or placebo. We extended our previous report^3^ by two second-generation antipsychotics and 15 first-generation antipsychotics and by investigating ten additional important outcomes, including specific aspects of efficacy, quality of life, and many more side-effects, and several methodological issues, including placebo response and sample sizes.^3^

Individual effect size estimates suggest that all antipsychotic drugs reduced overall symptoms more than placebo (not significant for six drugs) with mean effect sizes between −0·89 and −0·03 (median −0·42). However, overlapping CrIs between antipsychotics suggest that differences between most individual drugs were not significant. With few exceptions, only clozapine, amisulpride, zotepine, olanzapine, and risperidone were significantly more efficacious for the primary outcome than other antipsychotics. Readers should consult figure 3, which provides these comparisons. Amisulpride was among the most efficacious antipsychotics, but no placebo-controlled study was available, making this evidence entirely indirect. Nevertheless, amisulpride was significantly superior to placebo in older patients (≥60 years of age; SMD 0·86) and in patients with predominant negative symptoms (SMD 0·47).^28^, ^29^

Mainly newer antipsychotics provided data separately for positive and negative symptoms, but they were similar to data for overall change in symptoms. However, all included studies focused on positive symptoms, because studies with predominant negative symptoms were excluded in this analysis and were evaluated separately.^28^ Whether differences in negative symptoms relate to primary or just secondary negative symptoms is impossible to clarify in populations with positive symptoms. The fact that many drugs improved depressive symptoms more than placebo might also reflects a reduction of anxiety and distress associated with schizophrenia. Nevertheless, aripiprazole, brexpiprazole, cariprazine, lurasidone, and quetiapine are licensed in several countries for major depression and bipolar depression. So is flupentixol, but we did not find an antidepressant effect for it on the basis of sparse data (62 participants).^30^ Many antipsychotics did not have data for quality of life, an important outcome for patients because it combines efficacy and safety. If reported, most drugs showed better effects than placebo. Some but not all drugs also outperformed placebo in terms of social functioning in these short-term studies, an outcome associated with recovery and social reintegration.

Because all-cause discontinuation combines efficacy and tolerability, it has been used as a measure of effectiveness in the CATIE trial.^31^ When reported separately, more patients dropped out due to inefficacy (40%) than due to adverse events (20%) in the included trials so that all-cause discontinuation is primarily an efficacy measure.

Antipsychotics are often taken for a long period, so side-effects have an important role concerning morbidity and adherence and might affect cognition.^32^ Antipsychotics very often scored worse than placebo for side-effect outcomes, with different profiles. In general, older antipsychotics were associated often with more extrapyramidal motor side-effects and prolactin elevation (with noticeable exceptions, such as amisulpride, paliperidone, and risperidone), whereas many newer antipsychotics produced more weight gain and sedation. We consider weight gain to be a good proxy for metabolic side-effects in this already dense review.^33^ Specific metabolic side-effects such as glucose, insulin, homeostatic model assessment for insulin resistance, total cholesterol, LDL cholesterol, HDL cholesterol, and triglycerides will be addressed in future reviews. In contrast to our previous report, we present QTc prolongation in original units (ms), which facilitates clinical interpretation; lurasidone and the partial dopamine agonists were the most benign drugs.

With regard to efficacy and safety outcomes many older antipsychotics, limited by few direct comparisons, performed well compared with newer antipsychotics. This finding is important, because in low-income and middle-income countries, second-generation antipsychotics might not be affordable. However, older studies with negative results could have remained unpublished more frequently, whereas now all clinical trials should be registered. In an analysis of all antipsychotics compared with placebo, contour-enhanced funnel plots suggested the existence of unpublished studies.

Our analysis had limitations. We used strict inclusion criteria to obtain a homogenous sample, nevertheless the included studies were done over a 60-year period, during which study characteristics changed. Checking for consistency revealed few inconsistent loops and low-to-moderate heterogeneity in most outcomes (appendix pp 223, 224), but the overall power to detect inconsistency is low.^16^ Major exceptions were quality of life, for which a network meta-analysis was not calculated, and prolactin increase. Because prolactin results might depend on the laboratory assay used, we calculated SMDs in addition to mean differences (appendix p 281), which reduced heterogeneity strongly. Still, the finding that clozapine and zotepine significantly reduced prolactin compared with placebo (with wide CrIs) might be a statistical artifact driven by outliers, because only two small trials were available. The most important threat to the transitivity assumption of network meta-analysis was the increase of placebo response over the years,^27^, ^34^ because adjusting for placebo response in a metaregression strongly reduced heterogeneity (τ) by 60–63% (appendix pp 179–83). In this metaregression model, the ranking was not substantially different from the primary analysis. Additionally, removing placebo groups, placebo-controlled studies, and failed studies in sensitivity analyses did not substantially change the results nor did metaregressions of six other moderators and further sensitivity analyses, supporting the robustness of the findings. The results of the network meta-analysis were consistent overall with those of pairwise meta-analyses (figure 3) and single studies. For example, a study comparing brexpiprazole with placebo and quetiapine found that brexpiprazole was better than placebo, but worse than quetiapine, similar to the hierarchy of our analysis.^35^ In a long-term study (sponsored by asenapine's manufacturer) olanzapine was significantly better than asenapine.^36^ Thus, we do not believe that placebo response explains all efficacy differences between the compounds. Nevertheless, the statistical methods could not fully account for the heterogeneity,^27^ so some efficacy outcomes might appear larger than they actually are.

Our decision to exclude studies from mainland China reduces the generalisability of the results to this country. However, a literature and telephone interview study suggested that most Chinese trials continue to be of low quality.^14^ Chinese reports are usually very short and communication with the authors is often difficult due to language barriers, thus risk of bias is difficult to assess. Therefore, we a-priori decided to exclude Chinese studies.

Clinical trials exclude suicidal patients, and the severely ill are unlikely to be included in modern trials because providing informed consent is often not possible for them. With a mean duration of illness of 12 years, our sample consisted mainly of chronic patients, who are known to respond worse compared with first-episode patients.^37^ These factors reduce generalisability.

For feasibility reasons our risk of bias assessment focused on the primary outcome; however, risk of bias is outcome-specific (appendix pp 273–77). Moreover, the evidence for many secondary outcomes (eg, social functioning) was based on much lower sample sizes compared with the primary outcome (4370 *vs* 40 815).

These limitations reduced the strength of the derivable recommendations, particularly (but not only) for older antipsychotics, because their effect sizes are based primarily on one or two studies with sample sizes smaller than 100. Small sample sizes leave room for small trial effects, which might have inflated some results. For example, the large effect of clozapine concerning reduction of negative symptoms is based on 159 participants, because clozapine is mainly studied in treatment-resistant patients who were excluded from the analysis. The contribution of direct evidence is small for older drugs, resulting in wide CrIs, higher uncertainty, and lower confidence in the evidence evaluated by CINeMA. The generally smaller amounts of data available for old drugs, except for perphenazine, which had more evidence of good quality from a large trial,^31^ are highlighted in the figures and should be considered in the interpretation of all findings.

Because so many antipsychotic options are available, our results should help health-care providers find the most suitable drug for the individual patient, balancing side-effect profiles and the efficacy of different drugs. We confirm that antipsychotics differ more in their side-effects than in their efficacy. We believe that efficacy differences between compounds exist, but the fact that their measurement is based on subjective rating scales is problematic. The development of objective efficacy measures would render interpretation easier. Clinicians must remember that reported results are averages and that response and side-effects might vary considerably in individual patients.

**This online publication has been corrected. The corrected version first appeared at thelancet.com on September 12, 2019**
