# Department of Error

**DOI:** 10.1016/S0140-6736(19)31677-0

**Published:** 2019-09-14

*Huhn M, Nikolakopoulou A, Schneider-Thoma J, et al. Comparative efficacy and tolerability of 32 oral antipsychotics for the acute treatment of adults with multi-episode schizophrenia: a systematic review and network meta-analysis.* Lancet *2019; **394:** 939–51*—In this Article, the comparison between zotepine and haloperidol in figure 3 has been corrected to “0·08 (–0·19 to 0·36)”. The Declaration of Interests and the Acknowledgments have been updated to detail funding for the work and for Andrea Cipriani from the National Institute for Health Research. The copyright information has also been updated. These corrections have been made to the online version as of September 12, 2019, and the printed version is correct.

