# Supplementary material for: Comparative efficacy and tolerability of 32 oral antipsychotics for the acute treatment of adults with multi-episode schizophrenia: a systematic review and network meta-analysis
Source: Lancet. 2019 Sep 14;394(10202):939–51. doi: 10.1016/S0140-6736(19)31135-3 (PMC6891890; doi:10.1016/S0140-6736(19)31135-3)
Supplement: Supplementary appendix [file mmc1.pdf]

THE LANCET

Supplementary appendix

This appendix formed part of the original submission and has been peer reviewed. We post it as supplied by the authors.

Supplement to: Huhn M, Nikolakopoulou A, Schneider-Thoma J, et al. Comparative efficacy and tolerability of 32 oral antipsychotics for the acute treatment of adults with multi-episode schizophrenia: a systematic review and network meta-analysis. *Lancet* 2019; published online July 11. http://dx.doi.org/10.1016/S0140-6736(19)31135-3.

Appendix

Table of Contents:

Appendix 1: PRISMA Checklist 4
Appendix 2: Protocol 8
2.1. Protocol as published in prospero (Registration number: CRD42014014919) 8
2.2 Changes to the original protocol from November 2014 made 25th of August 2017 23
2.3 Differences between protocol and review 24
Appendix 3: Search Strategy 25
3.1 Original search October 2016 25
3.2 Update search November 08th, 2017 32
3.3 Update search January 08th, 2019 40
Appendix 4: Statistical methods in detail 49
Measures of treatment effect 49
Statistical details for network meta-analysis and meta-regression models 49
Assessment of the transitivity assumption 51
Assessment of inconsistency 52
Appendix 5: Assessment of transitivity 53
5.1 Placebo response 54
5.2 Publication year 56
5.3 Sample size 57
5.4 Baseline severity 58
5.5 Mean age 60
5.6 Percentage male 61
Appendix 6: PRISMA Flow Diagram 62
Appendix 7: List of included studies 64
Appendix 8: Risk of Bias 155
Appendix 9: Metaregression (adjusted models) 177
9.1 Placebo response 179
9.2 Publication year 184
9.3 Sample size 185
9.4 Age 186
9.5 Baseline severity 187
9.6 Male participants 188
9.7 Sponsorship 189
Appendix 10: Sensitivity analyses 190
10.1. Removing placebo controlled trials altogether 191

10.2. Removing only placebo arms from three or more arm trials 192
10.3. Risk of bias 193
10.4. Trial duration 194
10.5. Imputed standard deviations 195
10.6. Intention to treat analysis 196
10.7. Unfair dose 197
10.8. Older studies 198
10.9. Failed trials 199
Appendix 11: League tables 200
Appendix 12: Quality of life: Results from the pairwise meta-analysis 222
Appendix 13: Evaluation of heterogeneity and inconsistency 223
Appendix 14: Networkplots for secondary outcomes 225
14.1 Positive symptoms 225
14.2 Negative symptoms 226
14.3 Depressive symptoms 227
14.4 Quality of life 228
14.5 Social functioning 229
14.6 All-cause discontinuation 230
14.7 Weight gain 231
14.8 Use of antiparkinson medication 232
14.9 Akathisia 233
14.10 Prolactin 234
14.12 QTc prolongation 235
14.13 Sedation 236
14.14 Anticholinergic side effects 237
Appendix 15: Grading the evidence of the network meta-analysis using CINeMA 238
15.1 Reasons for downgrading 238
15.2 Confidence in evidence for all antipsychotics compared to placebo 242
15.3 CINeMA for the primary outcome “overall change in symptoms” 243
Appendix 16: Definition of covariates for metagressions 268
Placebo response rate 268
Study sample size 268
Publication year 268
Baseline severity 268
Sponsorship 268
Mean age 268
Percentage male 268
Appendix 17: Definition of sensitivity analyses 269
Duration 269

Studies with high risk of bias 269
Studies with imputed standard deviation 269
Placebo 269
a) Head to head studies 269
b) Only active arms 269
Studies using intent-to-treat-analysis 269
Only fair doses 269
Older Studies 269
Failed trials 270
Appendix 18: Description of outcome parameters 271
Appendix 19: Classification of individual risk of bias items 273
Appendix 20: Further outcomes 278
20.1 Response to treatment (study defined) 278
20.2 Discontinuation due to inefficacy 279
20.3 Participants with weight gain ≥ 7% 280
20.4 Prolactin elevation (SMD results) 281
Appendix 21: Characteristics of the sample 282
21.1 Descriptives of the overall sample 282
21.2 Descriptives of the overall sample separated by antipsychotics 283
Appendix 22: Funnel plots 285
23. References 286

Appendix 1: PRISMA Checklist

Section/Topic	Item #	Checklist Item	Reported on Page #
TITLE			
Title	1	Identify the report as a systematic review incorporating a network meta-analysis	1
ABSTRACT			
Structured summary	2	Provide a structured summary including, as applicable:<br>Background: main objectives<br>Methods: data sources; study eligibility criteria, participants, and interventions; study appraisal; and *synthesis methods, such as network meta-analysis.*<br>Results: number of studies and participants identified; summary estimates with corresponding confidence/credible intervals.<br>Discussion/Conclusions: limitations; conclusions and implications of findings.<br>Other: primary source of funding; systematic review registration number with registry name.	2
INTRODUCTION			
Rationale	3	Describe the rationale for the review in the context of what is already known.	3, Evidence in context
Objectives	4	Provide an explicit statement of questions being addressed, with reference to participants, interventions, comparisons, outcomes, and study design (PICOS).	3ff, Appendix 2
METHODS			
Protocol and registration	5	Indicate whether a review protocol exists and if and where it can be accessed (e.g., Web address); and, if available, provide registration information, including registration number.	Appendix 2, PROSPERO (CRD42014014919)
Eligibility criteria	6	Specify study characteristics (e.g., PICOS, length of follow-up) and report characteristics (e.g., years considered, language, publication status) used as criteria for eligibility, giving rationale.	3ff Appendix 2,
Information sources	7	Describe all information sources (e.g., databases with dates of coverage, contact with study authors to identify additional studies) in the search and date last searched.	4ff, Appendix 3
Search	8	Present full electronic search strategy for at least one database, including any limits used, such that it could be	4ff, Appendix 3

Study selection	9	State the process for selecting studies (i.e., screening, eligibility, included in systematic review, and, if applicable, included in the meta-analysis).	5ff, Appendix 2

Data collection process	10	Describe method of data extraction from reports (e.g., piloted forms, independently, in duplicate) and any processes for obtaining and confirming data from	5ff, Appendix 2
Data items	11	List and define all variables for which data were sought (e.g., PICOS, funding sources) and any assumptions and simplifications made.	5ff, Appendix 2,
Geometry of the network	S1	Describe methods used to explore the geometry of the treatment network under study and potential biases related to it. This should include how the evidence base has been graphically summarized for presentation, and what characteristics were compiled and used to describe the evidence base to readers.	5ff, Appendix 4
Risk of bias within individual studies	12	Describe methods used for assessing risk of bias of individual studies (including specification of whether this was done at the study or outcome level), and how this information is to be used in any data synthesis.	5ff, Appendix 2, 19
Summary measures	13	State the principal summary measures (e.g., risk ratio, difference in means).	5ff, Appendix 4
Planned methods of analysis	14	Describe the methods of handling data and combining results of studies for each network meta-analysis.	6ff, Appendix 4
Assessment of In-consistency	S2	Describe the statistical methods used to evaluate the agreement of direct and indirect evidence in the treatment network(s) studied. Describe efforts taken to address its presence when found.	6ff, Appendix 4, 13
Risk of bias across studies	15	Specify any assessment of risk of bias that may affect the cumulative evidence (e.g., publication bias, selective reporting within studies).	6ff, Appendix 15, 19
Additional analyses	16	Describe methods of additional analyses if done, indicating which were pre-specified. This may include, but not be limited to, the following:<br>• Sensitivity or subgroup analyses;<br>• Meta-regression analyses;	6ff, Appendix 9, 10, 16,17

RESULTS†

Study selection	17	Give numbers of studies screened, assessed for eligibility, and included in the review, with reasons for exclusions at each stage, ideally with a flow diagram.	7, Appendix 6,PRISMA
Presentation of network structure	S3	Provide a network graph of the included studies to enable visualization of the geometry of the treatment network.	Figure 1, Appendix 14
Summary of network geometry	S4	Provide a brief overview of characteristics of the treatment network. This may include commentary on the abundance of trials and randomized patients for the different interventions and pairwise comparisons in the network, gaps of evidence in the treatment network, and potential biases reflected by the network structure.	Appendix 14
Study characteristics	18	For each study, present characteristics for which data were extracted (e.g., study size, PICOS, follow-up period) and provide the citations.	List of included studies appendix 7
Risk of bias within studies	19	Present data on risk of bias of each study and, if available, any outcome level assessment.	Appendix 8
Results of individual studies	20	For all outcomes considered (benefits or harms), present, for each study: 1) simple summary data for each intervention group, and 2) effect estimates and confidence intervals.	Figure 2, 3 and 4
Synthesis of results	21	Present results of each meta-analysis done, including confidence/credible intervals. If additional summary measures were explored (such as treatment rankings), these should also be presented.	Figure 2, 3 and 4, league tables appendix 11
Exploration for inconsistency	S5	Describe results from investigations of inconsistency. This may include such information as measures of model fit to compare consistency and inconsistency models, *P* values from statistical tests, or summary of inconsistency estimates from different parts of the treatment network.	10f, appendix 13
Risk of bias across studies	22	Present results of any assessment of risk of bias across studies for the evidence base being studied.	10, appendix 15
Results of additional analyses	23	Give results of additional analyses, if done	11, appendix 9 and 10

DISCUSSION			
Summary of evidence	24	Summarize the main findings, including the strength of evidence for each main outcome; consider their relevance to key groups (e.g., healthcare providers, users, and policy- makers).	12ff
Limitations	25	Discuss limitations at study and outcome level (e.g., risk of bias), and at review level (e.g., incomplete retrieval of identified research, reporting bias).	13ff
Conclusions	26	Provide a general interpretation of the results in the context of other evidence, and implications for future research.	13f
FUNDING			
Funding	27	Describe sources of funding for the systematic review and other support (e.g., supply of data); role of funders for the systematic review. This should also include information regarding whether funding has been received from manufacturers of treatments in the network and/or whether some of the authors are content experts with professional	2, 15

PICOS = population, intervention, comparators, outcomes, study design.

Appendix 2: Protocol

2.1. Protocol as published in prospero (Registration number: CRD42014014919)

Review title and timescale

1 Review title

Give the working title of the review. This must be in English. Ideally it should state succinctly the interventions or exposures being reviewed and the associated health or social problem being addressed in the review.

Informing clinicians, patients and guidelines: network meta-analysis on 24 antipsychotic drugs and a broad ragne of important outcomes for schizophrenia.

2 Original language title

For reviews in languages other than English, this field should be used to enter the title in the language of the review. This will be displayed together with the English language title.

3 Anticipated or actual start date

Give the date when the systematic review commenced, or is expected to commence.

01/12/2014

4 Anticipated completion date

Give the date by which the review is expected to be completed.

30/12/2017

5 Stage of review at time of this submission

Indicate the stage of progress of the review by ticking the relevant boxes. Reviews that have progressed beyond the point of completing data extraction at the time of initial registration are not eligible for inclusion in PROSPERO. This field should be updated when any amendments are made to a published record.

The review has not yet started √

Review stage	Started	Completed
Preliminary searches	Yes	No
Piloting of the study selection process	No	No
Formal screening of search results against eligibility criteria	No	No
Data extraction	No	No
Risk of bias (quality) assessment	No	No
Data analysis	No	No

Provide any other relevant information about the stage of the review here.

Review team details

6 Named contact

The named contact acts as the guarantor for the accuracy of the information presented in the register record.

Mr Huhn

7 Named contact email

Enter the electronic mail address of the named contact.

maximilian.huhn@lrz.tum.de

8 Named contact address

Enter the full postal address for the named contact.

Klinik für Psychiatrie und Psychotherapie der TU-Mnchen Klinikum rechts der Isar Ismaningerstr. 22 81675 Mnchen Germany

9 Named contact phone number

Enter the telephone number for the named contact, including international dialing code.

+498941406466

10 Organisational affiliation of the review

Full title of the organisational affiliations for this review, and website address if available. This field may be completed as 'None' if the review is not affiliated to any organisation.

Department of Psychiatry and Psychotherapy, Technische Universität München, Klinikum rechts der Isar

Website address:

http://www.cfdm.de/

11 Review team members and their organisational affiliations

Give the title, first name and last name of all members of the team working directly on the review. Give the organisational affiliations of each member of the review team.

Title	First name	Last name	Affiliation

Professor	Stefan	Leucht	Department of Psychiatry and Psychotherapy, Technische Universität München, Klinikum rechts der Isar
Dr	Maximilian	Huhn	Department of Psychiatry and Psychotherapy, Technische Universität München, Klinikum rechts der Isar
Dr	Johannes	Schneider	Department of Psychiatry and Psychotherapy, Technische Universität München, Klinikum rechts der Isar
Professor	John	Davis	Department of Psychiatry and Psychotherapy, University of Illinois at Chicagots der Isar
Mr	Mark	Krause	Department of Psychiatry and Psychotherapy, Technische Universität München, Klinikum rechts der Isar

12 Funding sources/sponsors

Give details of the individuals, organizations, groups or other legal entities who take responsibility for initiating, managing, sponsoring and/or financing the review. Any unique identification numbers assigned to the review by the individuals or bodies listed should be included.

Bundesministerium für Bildung und Forschung (BMBF) Grant: 01KG1406

13 Conflicts of interest

List any conditions that could lead to actual or perceived undue influence on judgements concerning the main topic investigated in the review.

Are there any actual or potential conflicts of interest?

Yes

Stefan Leucht has received honoraria for lectures from Abbvie, Astra Zeneca, BristolMyersSquibb, ICON, EliLilly, Janssen, Johnson & Johnson, Roche, SanofiAventis, Lundbeck and Pfizer; for consulting/advisory boards from Roche, EliLilly, Medavante, BristolMyersSquibb, Alkermes, Janssen, Johnson & Johnson and Lundbeck. EliLilly has provided medication for a study with SL as primary investigator. Claudia Leucht is Stefan Leucht's spouse so that the same conflict of interest may also relate to her.

14 Collaborators

Give the name, affiliation and role of any individuals or organisations who are working on the review but who are not listed as review team members.

Title	First name	Last name	Organisation details
Professor	Georgia	Salanti	Department of Hygiene and Epidemiology, University of Ioannina
Dr	Adriani	Nikolakopoulou	Department of Hygiene and Epidemiology, University of Ioannina

Review methods

15 Review question(s)

State the question(s) to be addressed / review objectives. Please complete a separate box for each question.

To examine the comparative efficacy, acceptability, and tolerability of twelve second- and twelve first-generation antipsychotic drugs in schizophrenia by applying a network meta-analysis approach.

16 Searches

Give details of the sources to be searched, and any restrictions (e.g. language or publication period). The full search strategy is not required, but may be supplied as a link or attachment.

1. We will search the Cochrane Schizophrenia Group Controlled Trials Register (compiled by regular systematic hand searches and searches of more than 15 databases, clinical trial registers, the Food and Drug Administration web site, and

conference proceedings without language restrictions; available to us until version August 2009), MEDLINE, EMBASE, PsycINFO, Cochrane Library, Pubmed, Biosis, ClinicalTrials.gov and WHO ICTRP. The search phrases will combine terms for schizophrenia (schizophrenia-like psychoses), randomization, and antipsychotic drugs. The exact search terms will be detailed with the an expert librarian (Samantha Roberts, previous trial search coordinator of the Cochrane Schizophrenia Group. Term for MEDLINE: 1 Benperidol/ or Chlorpromazine/ or Clopenthixol/ or Clozapine/ or Flupenthixol/ or Fluphenazine/ or Fluspirilene/ or Haloperidol/ or Methotrimeprazine/ or Loxapine/ or Molindone/ or Penfluridol/ or Perazine/ or Perphenazine/ or Pimozide/ or Risperidone/ or Sulpiride/ or Thioridazine/ or Thiothixene/ or Trifluoperazine/ or Clopenthixol/ (53027) 2 (Amisulpride or Aripiprazole or Asenapine or Benperidol or Brexpiprazole or Cariprazine or Chlorpromazine or Clopenthixol or Clozapine or Flupenthixol or Fluphenazine or Haloperidol or Iloperidone or Levomepromazine or Loxapine or Lurasidone or Molindone or Olanzapine or Paliperidone or Quetiapine or Penfluridol or Perazine or Perphenazine or Pimozide or Risperidone or Sertindole or Sulpiride or Thioridazine or Thiothixene or Trifluoperazine or Ziprasidone or Zotepine or Zuclopenthixol).tw. (57996) 3 or/1-2 (75450) 4 exp schizophrenia/ (90375) 5 exp Paranoid Disorders/ (3848) 6 schizo$.mp. (142254) 7 hebephreni$.mp. (269) 8 oligophreni$.mp. (1063) 9 psychotic$.mp. (52549) 10 psychosis.mp. (27356) 11 psychoses.mp. (19225) 12 or/4-11 (190571) 13 exp clinical trial/ (848995) 14 exp randomized controlled trials/ (104559) 15 exp cross-over studies/ (37337) 16 randomized controlled trial.pt. (413628) 17 clinical trial.pt. (506934) 18 controlled clinical trial.pt. (91880) 19 (clinic$ adj2 trial).mp. (626861) 20 (random$ adj5 control$ adj5 trial$).mp. (558179) 21 (crossover or cross-over).mp. (75106) 22 ((singl$ or double$ or trebl$ or tripl$) adj (blind$ or mask$)).mp. (194708) 23 randomi$.mp. (662230) 24 (random$ adj5 (assign$ or allocat$ or assort$ or reciev$)).mp. (188450) 25 or/13-24 (1227013) 26 3 and 12 and 25 (6235) 2. Previous reviews: We will search previous reviews investigating antipsychotics in general schizophrenia. 3. Personal contact: We will contact the first author of each included study published in the last 30 years for missing information. 4. Drug companies: We will contact the principal manufacturers of the antipsychotic drugs and ask them for further relevant trials and for missing information concerning the identified studies. 5. Hand search: There will be no extra hand search for this review, because a number of psychiatric journals (especially old issues which are important for this project) and the abstract books of major conferences are regularly hand searched anyways for the 'Cochrane Schizophrenia Group Trials Register'. There will be no language restriction applied concerning the literature. As an exception we will exclude Chinese studies which often do not use appropriate randomization procedures and do not report their methods so that it is impossible to check on these issues.

17 URL to search strategy

If you have one, give the link to your search strategy here. Alternatively you can e-mail this to PROSPERO and we will store and link to it.

I give permission for this file to be made publicly available

Yes

18 Condition or domain being studied

Give a short description of the disease, condition or healthcare domain being studied. This could include health and wellbeing outcomes.

Schizophrenia

19 Participants/population

Give summary criteria for the participants or populations being studied by the review. The preferred format includes details of both inclusion and exclusion criteria.

We will include adult people (age >= 18, no upper age limit, no restriction in setting, gender, ethnicity) with schizophrenia or related disorders (such as schizophreniform, or schizoaffective disorders). There is no clear evidence that the latter schizophrenia-like psychoses are caused by fundamentally different disease processes or require different treatment approaches. However, as in our previous report we will exclude studies in treatment resistant patients, in patients with predominant negative symptoms, in patients with concomitant medical illness, and studies in stable patients (mainly relapse prevention studies), because these are different patient populations and it is an important requirement of network meta-analysis to have reasonably homogeneous samples. Studies in which less than 20% of the participants were suffering from other psychiatric disorders than schizophrenia (e.g. depression or mental retardation) will be acceptable. We will include the trials irrespective of the diagnostic criteria used. It is a general strategy of the Cochrane Schizophrenia Group (CSG) to include not only studies that used specific diagnostic criteria such as ICD-10 or DSM-IV, because these criteria are not meticulously used in clinical routine either. This decision should increase generalizability.

20 Intervention(s), exposure(s)

Give full and clear descriptions of the nature of the interventions or the exposures to be reviewed

We will include all second-generation (“atypical”) antipsychotic drugs available in Europe or the US (amisulpride, aripiprazole, asenapine, brexpiprazole, cariprazine, clozapine, iloperidone, lurasidone, olanzapine, paliperidone, quetiapine, risperidone, sertindole, ziprasidone, zotepine), placebo and a selection of first-generation (“typical”, “conventional”) antipsychotics (benperidol, chlopromazine, clopenthixol, flupenthixol, fluphenazine, fluspirilene, haloperidol, levomepromazine, loxapine, methotrimeprazine, molindone, penfluridol, perazine, perphenazine, pimozide, sulpiride, thioridazine, thiothixene, trifluoperazine, zuclopenthixol). Second-generation antipsychotics are in some countries such as the US or Germany nowadays the most frequently prescribed compounds, they are overall more costly (especially the most recent ones which still have patent protection) and they are thus obvious choices. One novel aspect of the network meta-analysis is the inclusion of first-generation antipsychotics. In addition to the reasons already mentioned (the classification in SGAs and FGAs is no longer valid, even in industrialised countries FGAs are still frequently used and FGAs are the mainstay of treatment world-wide), some FGAs suggested excellent properties in recent influential studies (e.g. perphenazine in CATIE (Lieberman et al. 2005) or sulpiride in CUtLASS (Jones et al. 2006)) and some may have atypical properties (e.g. thioridazine, sulpiride, flupenthixol, perazine). To guide our choice of FGAs we conducted a survey of 60 international schizophrenia experts whom we asked to choose 10 of the 52 FGAs listed by the “WHO Collaborating Centre for Drug Statistics” which they find most important for such a meta-analysis (http://www.whocc.no/atc_ddd_methodology/who_collaborating_centre/, detailed results can be found on our website www.cfdm.de/media/doc/Antipsychotic Survey.doc). We added benperidol and perazine which are frequently used FGAs in Germany. The selection includes FGAs from various classes, there are high-potency (e.g. haloperidol), mid-potency (zuclopenthixol, perphenazine) and low-potency (e.g. thioridazine) drugs, butyrophenones (e.g. benperidol, haloperidol), phenothiazines (e.g. fluphenazine), thioxantenes (zuclopenthixol) and a substituted benzamide (sulpiride). We will include all these compounds in any oral forms of administration (for example tablets or liquid). In fixed-dose studies we will only included target to maximum doses according to the International Consensus Study on Antipsychotic dose. We will include all flexible-dose studies, because these allow the investigators to titrate to the adequate dose for the individual patient. We will exclude depot formulations which are mainly used for long-term relapse prevention which is not the focus of this review.

21 Comparator(s)/control

Where relevant, give details of the alternatives against which the main subject/topic of the review will be compared (e.g. another intervention or a non-exposed control group).

In a network meta-analysis each treatment is compared with each other, therefore any treatment can be the comparator. But placebo is a natural reference that will be used in our occasion.

22 Types of study to be included

Give details of the study designs to be included in the review. If there are no restrictions on the types of study design eligible for inclusion, this should be stated.

We will included open and blinded randomized controlled trials (RCTs) comparing one antipsychotic drug with another antipsychotic agent or placebo. Results from non double-blinded trials will be considered only for objective outcomes. Trials in which antipsychotic drugs were used as an augmentation- or combination strategy will be excluded. In the case of cross-over studies we will use only the first cross-over phase to avoid the problem of carry-over effects which are very likely in schizophrenia. We will exclude cluster randomized trials due to the unit-of-analysis-problems associated with this design (it is anyhow unlikely that such studies on our question exist). We will also only include double-blinded studies for subjective outcomes because we recently showed that a lack of blinding can exaggerate differences between treatments in this area. For objective outcomes (e.g. weight gain) blinding is less of a problem. The minimum duration of follow-up will be 3 weeks as shorter trials are unlikely to find significant differences. In addition, short-term results (3 weeks-3 months weeks) and longer term results (>3 months) will be analysed in separate publications.

23 Context

Give summary details of the setting and other relevant characteristics which help define the inclusion or exclusion criteria.

We will include adult people (age >= 18, no upper age limit, no restriction in setting, gender, ethnicity) with schizophrenia, schizophreniform or schizoaffective disorders with an acute exacerbation, primarily irrespective of the diagnostic criteria used. There is no clear evidence that the latter schizophrenia-like psychoses are caused by fundamentally different disease processes or require different treatment approaches. It is also a general strategy of the Cochrane Schizophrenia Group to include not only studies that used specific diagnostic criteria such as ICD-10 or DSM-IV, because these criteria are not meticulously used in clinical routine either. Studies in which less than 20% of the participants were suffering from other psychiatric disorders (e.g. depression or mental retardation) will be included. We will exclude studies in participants with no or only subclinical symptoms at baseline that are usually conducted to address the relapse preventing effects of antipsychotics, studies in patients with predominant negative symptoms and studies including exclusively participants with major concomitant somatic illness or psychiatric disorders (e.g. substance abuse).

24 Primary outcome(s)

Give the most important outcomes.

Mean reduction in overall symptoms of schizophrenia

Give information on timing and effect measures, as appropriate.

The primary outcome will be overall symptoms of schizophrenia as measured by rating scales such as the Positive and Negative Syndrome Scale (PANSS), the Brief Psychiatric Rating Scale (BPRS) or of any other validated scale (e.g. the Manchester Scale) for the assessment of overall schizophrenic symptomatology. Overall symptoms of schizophrenia as measured by such scales was the primary outcome in numerous previous systematic reviews. As not all studies will have used the same scale, we will apply the following hierarchy: mean change of the PANSS total score from baseline to endpoint, if not available mean change of the BPRS, or if again not available the mean values at endpoint of the PANSS/ BPRS. The results of other rating scales will only be used if the instrument has been published in a peer-reviewed journal, because it has been shown that unvalidated schizophrenia scales exaggerate differences. The minimum duration of follow-up will be 3 weeks as shorter trials are unlikely to find significant differences. Outcomes will be classified into short-term results (3 weeks-3 months) where the primary time point will be six weeks, if available, and longer term results (>3 months).

25 Secondary outcomes

List any additional outcomes that will be addressed. If there are no secondary outcomes enter None.

1. Response to treatment (study defined) (s) 2. Change in positive symptoms of schizophrenia (s) 3. Change in negative symptoms of schizophrenia (s) 4. Change in depressive symptoms (s) 5. Dropout due to any reason (all-cause discontinuation) (s) 6. Dropout due to inefficacy of treatment (s) 7. Adverse events a) Use of antiparkinson medication (s) b) Akathisia (s) c) Weight gain d) Prolactin levels e) At least one sexual side-effect (s) f) Sedation/somnolence (s) g) Cardiac side-effects, in particular QTc prolongation h) At least one anticholinergic side-effect (s). i) 8. Patient subjective well-being, quality of life (s) +9. Overall functioning (s) + Subjective outcomes are marked with an (s).

Give information on timing and effect measures, as appropriate.

The minimum duration of follow-up will be 3 weeks as shorter trials are unlikely to find significant differences. Outcomes will be classified into short-term results (3 weeks-3 months) where the primary time point will be six weeks, if available, and longer term results (>3 months).

26 Data extraction (selection and coding)

Give the procedure for selecting studies for the review and extracting data, including the number of researchers involved and how discrepancies will be resolved. List the data to be extracted.

1. Selection of trials: Two reviewers will independently inspect all abstracts identified in the searches. Disagreement will be resolved by discussion, and where doubt still remains, we will acquire the full article for further inspection. Once the full articles are obtained, at least two reviewers will independently decide whether the studies meet the review criteria. If disagreement can not be resolved by discussion, we will resolve it with a third reviewer or seek further information from the study authors. 2. Data extraction: Two reviewers will independently extract data from all selected trials on electronic forms. When disagreement arises we will resolve it by discussion with a third reviewer. Where this is not possible we will contact the study authors.

27 Risk of bias (quality) assessment

State whether and how risk of bias will be assessed, how the quality of individual studies will be assessed, and whether and how this will influence the planned synthesis.

Study quality in terms of sequence generation, allocation concealment, blinding, the completeness of outcome data, selective reporting and other biases will be assessed with the Cochrane Collaboration risk of bias tool.

28 Strategy for data synthesis

Give the planned general approach to be used, for example whether the data to be used will be aggregate or at the level of individual participants, and whether a quantitative or narrative (descriptive) synthesis is planned. Where appropriate a brief outline of analytic approach should be given.

1. Two-step procedure In a first step we will perform series of conventional pair-wise meta-analyses by combining studies that compared the same interventions. In a second step we will then perform a network meta-analysis within a Bayesian framework. 2. Continuous outcomes: The effect size measure for continuous outcomes will be the standardized mean difference (SMD) because we expect that the studies use different rating scales of overall schizophrenia symptomatology, especially the Positive and Negative Syndrome Scale (PANSS) or the Brief Psychiatric Rating Scale (BPRS) (see outcomes, above). Intention-to-treat (ITT) data will be used whenever available. Missing standard deviations: When standard errors instead of standard deviations (SD) are presented, the former will be converted to standard deviations (SDs). If both are missing we will estimate SDs from confidence intervals, t-values, or p-values as described in Section 7.7.3 of the Cochrane Handbook for Systematic Reviews. If none of these options is viable we will contact the original authors. When no information can be obtained we will derive SDs from those of the other studies using a validated

imputation technique. 3. Dichotomous outcomes: The effect size for dichotomous outcomes will be the odds ratio (OR) and its 95% confidence intervals (CIs). The main reason to prefer odds ratios to relative risks is that this measure has mathematical properties that make it more appropriate for network meta-analysis (e.g. the odds ratio is symmetrical). Another reason justifying the calculation of the odds ratios is the expectation that different definitions of 'response to treatment' will be used in the original trials and in such a situation the odds ratio has been shown to yield the most consistent results which are largely independent from the response cut-off used. Therefore, although the relative risk is more intuitive for clinician, the odds ratio has clear advantages for the purpose of our review. Analyses will be carried out in accordance to the 'intention-to-treat' principal when possible ('once randomized always analyze'). Everyone allocated to the intervention will be counted whether they completed the follow up or not. If the authors applied such a strategy, we will use their results. If the original authors presented only the results of the per-protocol or completer population, we will assume that those participants lost to follow-up would not have changed in a given outcome. In terms of efficacy this means that they would be conservatively considered to have not responded to treatment. In terms of tolerability it would mean that participants would not have developed a side-effect which we feel is appropriate, because otherwise side-effects, many of which are rare, would be overestimated. Applying this approach led to meaningful results in our previous reports on which we build. 4. Assessment of heterogeneity The heterogeneity (variability in relative treatment effects within the same treatment comparison) will be measured with the tau-squared (the variance of the random effects distribution). The heterogeneity variance will be assumed common across the various treatment comparisons and the empirical distributions will be used to characterise the amount of heterogeneity as low, moderate or high using the first and third quantiles (https://www.ncbi.nlm.nih.gov/pubmed/26679486 and https://www.ncbi.nlm.nih.gov/pubmed /22461129). Potential reasons for heterogeneity will be explored by subgroup analysis and meta-regressions (see 8. below). 5. Assessment of the transitivity assumption Joint analysis of treatments can be misleading if the network is substantially intransitive. We will need to investigate the distribution of clinical and methodological variables that can act as effect modifiers across treatment comparisons. The main features, which have been demonstrated to date to moderate efficacy of antipsychotics, at least compared to placebo, are the degree of placebo response (which has increased over the years) and industry sponsorship. Less robust factors include severity of illness at baseline, gender, chronicity and publication year. We will investigate if these variables are similarly distributed across studies grouped by comparison, whereas it is clear a priori that publication year, a composite of various factors, will differ between older and more recent antipsychotics. We will consider that placebo response in schizophrenia has increased over the years and that there could be differences between placebo-controlled trials and head-to-head trials as it is known from antidepressant trials in major depressive disorder. 6. Network meta-analyses We assume that patients who fulfill the inclusion criteria outlined above are equally likely to be randomised to any of the antipsychotic that we plan to compare. If the collected studies appear to be sufficiently similar with respect to the distribution of effect modifiers (refer to "assessment of transitivity assumption" section), we will

conduct a random effects NMA to synthesize all evidence for each outcome, and obtain a comprehensive ranking of all treatments. We will use arm-level data and the binomial likelihood for dichotomous outcomes. We will account for the correlations induced by multi-arm studies by employing multivariate distributions. We will assume a single heterogeneity parameter for each network. We will present the summary ORs or SMD for all pairwise comparisons in a league table. We will also estimate the prediction intervals to assess how much the common heterogeneity affects the relative effect with respect to the extra uncertainty anticipated in a future study. To rank the various treatments for each outcome, we will use the surface under the cumulative ranking curve (SUCRA) and the mean ranks. 7. Assessment of inconsistency The strategical and conceptual evaluation of transitivity will be supplemented with a statistical evaluation of consistency, the agreement between direct and indirect evidence. We will employ local as well as global methods to evaluate consistency. Local methods detect 'hot spots' of inconsistency, evidence loops that are inconsistent or comparisons for which direct and indirect evidence disagree. We will employ the loop-specific approach to evaluate inconsistency within each loop of evidence, and a method that separates direct evidence from indirect evidence provided by the entire network. We will also evaluate consistency in the entire network by calculating the I2 for network heterogeneity, inconsistency, and for both. Tests for inconsistency are known to have low power, and empirical evidence has suggested that 10% of evidence loops published in the medical literature are expected to be inconsistent. Therefore, interpretation of the statistical inference about inconsistency will be carried out with caution and possible sources of inconsistency will be explored even in the absence of evidence for inconsistency. 8. Exploring heterogeneity and inconsistency and sensitivity analyses We expect small amounts of heterogeneity and inconsistency to be present given the variety of study settings we plan to include. We will explore whether treatment effect for the primary outcome is robust in subgroup analyses and network meta-regression using the characteristics presented under 'analysis of subgroups or subsets'. 9. Publication bias We will explore the association between study size and effect size with a comparison-adjusted funnel plot that has been adapted to network meta-analysis. 10. Statistical software The analysis and presentation of results will be performed using the Stata packages network and network_graphs, the R package netmeta and self-programmed codes in OpenBUGS.

29 Analysis of subgroups or subsets

Give any planned exploration of subgroups or subsets within the review. 'None planned' is a valid response if no subgroup analyses are planned.

The following potential effect moderators of the primary outcome will be explored by subgroup or meta-regression analysis: 1. Dose of the antipsychotics in olanzapine-equivalents according to Gardner et al. 2010 2. Publication date (to address the effect of possibly generally decreasing effect sizes over time) 3. Severity of illness at baseline 4. Industry sponsorship 5. Length of follow-up 6. Mean participant age 7. Percentage men 8. Degree of placebo response 9. Small

versus large studies by regressing on the variance of the estimated effect size Sensitivity analyses will be performed as follows 1. Excluding studies characterized as pertaining to high risk of bias (defined as in the protocol of “GRISELDA”, a NMA on antidepressants) 2. Excluding studies that presented only completer analyses. 3. Excluding placebo-controlled studies 4. Excluding studies with imputed standard deviations.

Review general information

30 Type and method of review

Select the type of review and the review method from the drop down list.

Intervention, Systematic review

31 Language

Select the language(s) in which the review is being written and will be made available, from the drop down list. Use the control key to select more than one language.

English, German

Will a summary/abstract be made available in English?

Yes

32 Country

Select the country in which the review is being carried out from the drop down list. For multi-national collaborations select all the countries involved. Use the control key to select more than one country.

Germany

33 Other registration details

Give the name of any organisation where the systematic review title or protocol is registered together with any unique identification number assigned. If extracted data will be stored and made available through a repository such as the Systematic Review Data Repository (SRDR), details and a link should be included here.

34 Reference and/or URL for published protocol

Give the citation for the published protocol, if there is one.

Give the link to the published protocol, if there is one. This may be to an external site or to a protocol deposited with CRD in pdf format.

I give permission for this file to be made publicly available

Yes

35 Dissemination plans

Give brief details of plans for communicating essential messages from the review to the appropriate audiences.

The results will be published in major psychiatric journals and presented at major international and German psychiatric conferences. Our findings will be rapidly implemented in national and international treatment guidelines, for some of which Stefan Leucht is a co-author.

Do you intend to publish the review on completion?

Yes

36 Keywords

Give words or phrases that best describe the review. (One word per box, create a new box for each term)

schizophrenia

antipsychotics

meta-analysis

placebo

FGA

SGA

37 Details of any existing review of the same topic by the same authors

Give details of earlier versions of the systematic review if an update of an existing review is being registered, including full bibliographic reference if possible.

38 Current review status

Review status should be updated when the review is completed and when it is published.

Ongoing

39 Any additional information

Provide any further information the review team consider relevant to the registration of the review.

40 Details of final report/publication(s)

This field should be left empty until details of the completed review are available.

Give the full citation for the final report or publication of the systematic review.

Give the URL where available.

2.2 Changes to the original protocol from November 2014 made 25th of August 2017

11. Review team members: we updated the team members

16. Searches: we described our search strategy in more detail.

20. Interventions: we added the two newly approved antipsychotics brexpiprazole and cariprazine
21: Comparator: we rewrote these sentences to make it clear that in a network metaanalysis all comparisons are compared with each other.

25. Secondary outcomes: we shortened the section and removed some outcomes

28. Strategy for data synthesis: we described our statistical approach in more detail

29. Analysis of subsets: we removed some planned subgroup analysis, because we realized that some of the proposed analyses are not meaningful.

2.3 Differences between protocol and review

Due to requests by peer-reviewers of the manuscript, a number of changes and additions to the original protocol had to be made which are summarised in the following text:

- One reviewer requested risk ratios instead of odd´s ratios for dichotomous outcomes and weighted mean differences instead of standardized mean differences for continous outcomes. We followed these suggestions and updated the analyses accordingly.
- One reviewer requested a sensitivity analysis exluding trials published before 1990
- One reviewer requested a sensitivty analysis exluding “failed trials”
- The following outcomes were requested by the reviewers:
    - Participants with weight gain >7% from baseline
    - Responder rates (study defined)
    - Discontinuation due to inefficacy

Appendix 3: Search Strategy

3.1 Original search October 2016

Database: Ovid MEDLINE(R) <1946 to September Week 4 2016>

Search Strategy:

----

1 (Acepromazine or Acetophenazine or Amisulpride or Aripiprazole or Asenapine or Benperidol or Blonanserin or Bromperidol or Butaperazine or Carpipramine or Chlorproethazine or Chlorpromazine or Chlorprothixene or Clocapramine or Clopenthixol or Clopentixol or Clothiapine or Clotiapine or Clozapine or Cyamemazine or Cyamepromazine or Dixyrazine or Droperidol or Fluanisone or Flupehenazine or Flupenthixol or Flupentixol or Fluphenazine or Fluspirilen or Fluspirilene or Haloperidol or Iloperidone or Levomepromazine or Levosulpiride or Lithium or Loxapine or Loxapinsuccinate or Lurasidone or Melperone or Mepazine or Mesoridazine or Methotrimeprazine or Molindone or Moperone or Mosapramine or Olanzapine or Oxypertine or Paliperidone or Penfluridol or Perazine or Periciazine or Pericyazine or Perospirone or Perphenazine or Pimozide or Pipamperone or Pipothiazine or Pipotiazine or Prochlorperazine or Promazine or Promethazine or Prothipendyl or Quetiapine or Remoxipiride or Reserpine or Riospirone or Risperdal or Risperidone or Seroquel or Sertindole or Stelazine or Sulpiride or Sultopride or Thiopropazate or Thioproperazine or Thioridazine or Tiospirone or Thiothixene or Tiapride or Tiotixene or Trifluoperazine or Trifluperidol or trifluoperidol or Triflupromazine or trifluperazine or Veralipride or Ziprasidone or Zotepine or Zuclopenthixol).mp. (134617)

2 (Antipsychoti$ or Anti-psychotic$ or Neurolepic$ or Neurolept$).mp. (68689)

3 Antipsychotic Agents/ (48799)

4 or/1-3 (168445)

5 exp Placebos/ (33734)

6 placebo.tw. (169937)

7 or/5-6 (183564)

8 exp schizophrenia/ (99224)

9 exp Paranoid Disorders/ (3931)

10 schizo$.mp. (143591)

11 hebephreni$.mp. (272)

12 oligophreni$.mp. (1094)

13 psychotic$.mp. (58621)

14 psychosis.mp. (27678)

15 psychoses.mp. (20127)

16 or/8-15 (193019)

17 exp clinical trial/ (759975)

18 exp randomized controlled trials/ (112472)

19 exp double-blind method/ (139724)

20 exp single-blind method/ (22909)

21 exp cross-over studies/ (39763)

22 randomized controlled trial.pt. (432377)

23 clinical trial.pt. (506031)

24 controlled clinical trial.pt. (91773)

25 (clinic$ adj2 trial).mp. (627099)

26 (random$ adj5 control$ adj5 trial$).mp. (572524)

27 (crossover or cross-over).mp. (71611)

28 ((singl$ or double$ or trebl$ or tripl$) adj (blind$ or mask$)).mp. (192757)

29 randomi$.mp. (660138)

30 (random$ adj5 (assign$ or allocat$ or assort$ or reciev$)).mp. (186901)

31 or/17-30 (1112912)

32 4 and 7 and 16 and 31 (2591)

33 limit 32 to ed=20140607-20161011 (263)

Database: Embase <1974 to 2016 Week 41>

Search Strategy:

----

1 (Acepromazine or Acetophenazine or Amisulpride or Aripiprazole or Asenapine or Benperidol or Blonanserin or Bromperidol or Butaperazine or Carpipramine or Chlorproethazine or Chlorpromazine or Chlorprothixene or Clocapramine or Clopenthixol or Clopentixol or Clothiapine or Clotiapine or Clozapine or Cyamemazine or Cyamepromazine or Dixyrazine or Droperidol or Fluanisone or Flupehenazine or Flupenthixol or Flupentixol or Fluphenazine or Fluspirilen or Fluspirilene or Haloperidol or Iloperidone or Levomepromazine or Levosulpiride or Lithium or Loxapine or Loxapinsuccinate or Lurasidone or Melperone or Mepazine or Mesoridazine or Methotrimeprazine or Molindone or Moperone or Mosapramine or Olanzapine or Oxypertine or Paliperidone or Penfluridol or Perazine or Periciazine or Pericyazine or Perospirone or Perphenazine or Pimozide or Pipamperone or Pipothiazine or Pipotiazine or Prochlorperazine or Promazine or Promethazine or Prothipendyl or Quetiapine or Remoxipiride or Reserpine or Riospirone or Risperdal or Risperidone or Seroquel or Sertindole or Stelazine or Sulpiride or Sultopride or Thiopropazate or Thioproperazine or Thioridazine or Tiospirone or Thiothixene or Tiapride or Tiotixene or Trifluoperazine or Trifluperidol or trifluoperidol or Triflupromazine or trifluperazine or Veralipride or Ziprasidone or Zotepine or Zuclopenthixol).mp. (274234)

2 (Antipsychoti$ or Anti-psychotic$ or Neurolepic$ or Neurolept$).mp. (110686)

3 neuroleptic agent/ (73095)

4 or/1-3 (320703)

5 exp placebo/ (323475)

6 placebo.tw. (244672)

7 or/5-6 (383830)

8 exp schizophrenia/ (168572)

9 exp psychosis/ (254834)

10 schizo$.mp. (200370)

11 hebephreni$.mp. (915)

12 oligophreni$.mp. (1686)

13 psychotic$.mp. (45296)

14 psychosis.mp. (114103)

15 psychoses.mp. (11477)

16 or/8-15 (302053)

17 (clin$ adj2 trial).mp. (1254866)

18 ((singl$ or doubl$ or trebl$ or tripl$) adj (blind$ or mask$)).mp. (247881)

19 (random$ adj5 (assign$ or allocat$)).mp. (145518)

20 randomi$.mp. (908448)

21 crossover.mp. (80017)

22 exp randomized-controlled-trial/ (453734)

23 exp double-blind-procedure/ (136371)

24 exp crossover-procedure/ (53184)

25 exp single-blind-procedure/ (25985)

26 exp randomization/ (82914)

27 or/17-26 (1798092)

28 4 and 7 and 16 and 27 (5986)

29 limit 28 to dd=20140607-20161011 (313)

Database: PsycINFO <1806 to October Week 1 2016>

Search Strategy:

----

1 (Acepromazine or Acetophenazine or Amisulpride or Aripiprazole or Asenapine or Benperidol or Blonanserin or Bromperidol or Butaperazine or Carpipramine or Chlorproethazine or Chlorpromazine or Chlorprothixene or Clocapramine or Clopenthixol or Clopentixol or Clothiapine or Clotiapine or Clozapine or Cyamemazine or Cyamepromazine or Dixyrazine or Droperidol or Fluanisone or Flupehenazine or Flupenthixol or Flupentixol or Fluphenazine or Fluspirilen or Fluspirilene or Haloperidol or Iloperidone or Levomepromazine or Levosulpiride or Lithium or Loxapine or Loxapinsuccinate or Lurasidone or Melperone or Mepazine or Mesoridazine or Methotrimeprazine or Molindone or Moperone or Mosapramine or Olanzapine or Oxypertine or Paliperidone or Penfluridol or Perazine or Periciazine or Pericyazine or Perospirone or Perphenazine or Pimozide or Pipamperone or Pipothiazine or Pipotiazine or Prochlorperazine or Promazine or Promethazine or Prothipendyl or Quetiapine or Remoxipiride or Reserpine or Riospirone or Risperdal or Risperidone or Seroquel or Sertindole or Stelazine or Sulpiride or Sultopride or Thiopropazate or Thioproperazine or Thioridazine or Tiospirone or Thiothixene or Tiapride or Tiotixene or Trifluoperazine or Trifluperidol or trifluoperidol or Triflupromazine or trifluperazine or Veralipride or Ziprasidone or Zotepine or Zuclopenthixol).mp. (40310)

2 (Antipsychoti$ or Anti-psychotic$ or Neurolepic$ or Neurolept$).mp. (37083)

3 neuroleptic drugs/ (18603)

4 or/1-3 (59597)

5 exp Placebo/ (4685)

6 placebo.tw. (35064)

7 or/5-6 (35154)

8 exp Schizophrenia/ (80097)

9 exp psychosis/ (102153)

10 schizo$.mp. (121444)

11 hebephreni$.mp. (535)

12 oligophreni$.mp. (520)

13 psychotic$.mp. (41605)

14 psychosis.mp. (47236)

15 psychoses.mp. (14892)

16 or/8-15 (167684)

17 ((singl$ or doubl$ or trebl$ or tripl$) adj (blind$ or mask$)).mp. (22523)

18 (random$ adj5 (assign$ or allocat$)).mp. (35986)

19 randomi$.mp. (63633)

20 crossover.mp. (6185)

21 or/17-20 (103533)

22 7 and 16 and 21 (2403)

23 limit 22 to up=20140607-20161011 (311)

Pubmed

#19	Search **(#1 and #6 and #10 and #16 and #18)**	123
#18	Search **("2014/06/07"[Date - Create] : "2016/10/11"[Date - Create])**	2660576
#16	Search **(#12 or #14 or #15)**	95195
#15	Search **((schizo* or hebephreni* or oligophreni* or psychotic* or psychosis or psychoses)[Text Word])**	19
#14	Search **"Paranoid Disorders"[Mesh]**	3876
#12	Search **"Schizophrenia"[Mesh]**	92621
#10	Search **(#8 or #9)**	193347
#9	Search **placebo[Text Word]**	179921
#8	Search **"Placebos"[Mesh]**	33244
#6	Search **(#2 or #3 or #5)**	183439
#5	Search **"Antipsychotic Agents"[Mesh]**	46802
#3	Search **((Antipsychoti* or Anti-psychotic* or Neurolepic* or Neurolept*))**	71007
#2	Search **((Acepromazine or Acetophenazine or Amisulpride or Aripiprazole or Asenapine or Benperidol or Blonanserin or Bromperidol or Butaperazine or Carpipramine or Chlorproethazine or Chlorpromazine or Chlorprothixene or Clocapramine or Clopenthixol or Clopentixol or Clothiapine or Clotiapine or Clozapine or Cyamemazine or Cyamepromazine or Dixyrazine or Droperidol or Fluanisone or Flupehenazine or Flupenthixol or Flupentixol or Fluphenazine or Fluspirilen or Fluspirilene or Haloperidol or Iloperidone or Levomepromazine or Levosulpiride or Lithium or Loxapine or Loxapinsuccinate or Lurasidone or Melperone or Mepazine or Mesoridazine or Methotrimeprazine or Molindone or Moperone or Mosapramine or Olanzapine or Oxypertine or Paliperidone or Penfluridol or Perazine or Periciazine or Pericyazine or Perospirone or Perphenazine or Pimozide or Pipamperone or Pipothiazine or Pipotiazine or Prochlorperazine or Promazine or Promethazine or Prothipendyl or Quetiapine or Remoxipiride or Reserpine or Riospirone or Risperdal or Risperidone or Seroquel or Sertindole or Stelazine or Sulpiride or Sultopride or Thiopropazate or Thioproperazine or Thioridazine or Tiospirone or Thiothixene or Tiapride or Tiotixene or Trifluoperazine or Trifluperidol or trifluoperidol or Triflupromazine or trifluperazine or Veralipride or Ziprasidone or Zotepine or Zuclopenthixol))**	148349
#1	Search **((randomized controlled trial[pt]) OR (controlled clinical trial[pt]) OR (randomized[tiab]) OR (placebo[tiab]) OR (drug therapy[sh]) OR (randomly[tiab]) OR (trial[tiab]) OR (groups[tiab])) NOT (animals[mh] NOT humans[mh])**	3359830

Cochrane

ID Search

#1 (Acepromazine or Acetophenazine or Amisulpride or Aripiprazole or Asenapine or Benperidol or Blonanserin or Bromperidol or Butaperazine or Carpipramine or Chlorproethazine or Chlorpromazine or Chlorprothixene or Clocapramine or Clopenthixol or Clopentixol or Clothiapine or Clotiapine or Clozapine or Cyamemazine or Cyamepromazine or Dixyrazine or Droperidol or Fluanisone or Flupehenazine or Flupenthixol or Flupentixol or Fluphenazine or Fluspirilen or Fluspirilene or Haloperidol or Iloperidone or Levomepromazine or Levosulpiride or Lithium or Loxapine or Loxapinsuccinate or Lurasidone or Melperone or Mepazine or Mesoridazine or Methotrimeprazine or Molindone or Moperone or Mosapramine or Olanzapine or Oxypertine or Paliperidone or Penfluridol or Perazine or Periciazine or Pericyazine or Perospirone or Perphenazine or Pimozide or Pipamperone or Pipothiazine or Pipotiazine or Prochlorperazine or Promazine or Promethazine or Prothipendyl or Quetiapine or Remoxipiride or Reserpine or Riospirone or Risperdal or Risperidone or Seroquel or Sertindole or Stelazine or Sulpiride or Sultopride or Thiopropazate or Thioproperazine or Thioridazine or Tiospirone or Thiothixene or Tiapride or Tiotixene or Trifluoperazine or Trifluperidol or trifluoperidol or

Triflupromazine or trifluperazine or Veralipride or Ziprasidone or Zotepine or Zuclopenthixol):ti,ab,kw (Word variations have been searched)

#2 (Antipsychoti* or Anti-psychotic* or Neurolepic* or Neurolept*):ti,ab,kw (Word variations have been searched)

#3 MeSH descriptor: [Antipsychotic Agents] explode all trees

#4 #1 or #2 or #3

#5 MeSH descriptor: [Placebos] explode all trees

#6 Placebo:ti,ab,kw (Word variations have been searched)

#7 #5 or #6

#8 MeSH descriptor: [Schizophrenia] explode all trees

#9 MeSH descriptor: [Paranoid Disorders] explode all trees

#10 schizo* or hebephreni* or oligophreni* or psychotic* or psychosis or psychoses:ti,ab,kw (Word variations have been searched)

#11 #8 or #9 or #10

#12 #4 and #7 and #11 in Trials limited to publication year 2014-2016 (312)

BIOSIS

# 16	**172**	#15 AND #14 AND #13 AND #10 AND #1<br>*Indexes=BCI Timespan=2014-2016*
# 15	**15,374**	**TOPIC:** ((schizo* or hebephreni* or oligophreni* or psychotic* or psychosis or psychoses)) *OR* **TITLE:** ((schizo* or hebephreni* or oligophreni* or psychotic* or psychosis or psychoses))<br>*Indexes=BCI Timespan=2014-2016*
# 14	**12,809**	**TOPIC:** (Placebo*) *OR* **TITLE:** (Placebo*)<br>*Indexes=BCI Timespan=2014-2016*
# 13	**12,645**	#12 OR #11<br>*Indexes=BCI Timespan=2014-2016*
# 12	**8,278**	**TOPIC:** (Antipsychoti* or Anti-psychotic* or Neurolepic* or Neurolept*) *OR* **TITLE:** (Antipsychoti* or Anti-psychotic* or Neurolepic* or Neurolept*)<br>*Indexes=BCI Timespan=2014-2016*
# 11	**7,453**	**TOPIC:** (Acepromazine or Acetophenazine or Amisulpride or Aripiprazole or Asenapine or Benperidol or Blonanserin or Bromperidol or Butaperazine or Carpipramine or Chlorproethazine or Chlorpromazine or Chlorprothixene or Clocapramine or Clopenthixol or Clopentixol or Clothiapine or Clotiapine or Clozapine or Cyamemazine or Cyamepromazine or Dixyrazine or Droperidol

or Fluanisone or Flupehenazine or Flupenthixol or Flupentixol or Fluphenazine or Fluspirilen or Fluspirilene or Haloperidol or Iloperidone or Levomepromazine or Levosulpiride or Lithium or Loxapine or Loxapinsuccinate or Lurasidone or Melperone or Mepazine or Mesoridazine or Methotrimeprazine or Molindone or Moperone or Mosapramine or Olanzapine or Oxypertine or Paliperidone or Penfluridol or Perazine or Periciazine or Pericyazine or Perospirone or Perphenazine or Pimozide or Pipamperone or Pipothiazine or Pipotiazine or Prochlorperazine or Promazine or Promethazine or Prothipendyl or Quetiapine or Remoxipiride or Reserpine or Riospirone or Risperdal or Risperidone or Seroquel or Sertindole or Stelazine or Sulpiride or Sultopride or Thiopropazate or Thioproperazine or Thioridazine or Tiospirone or Thiothixene or Tiapride or Tiotixene or Trifluoperazine or Trifluperidol or trifluoperidol or Triflupromazine or trifluperazine or Veralipride or Ziprasidone or Zotepine or Zuclopenthixol) *OR* **TITLE:** (Acepromazine or Acetophenazine or Amisulpride or Aripiprazole or Asenapine or Benperidol or Blonanserin or Bromperidol or Butaperazine or Carpipramine or Chlorproethazine or Chlorpromazine or Chlorprothixene or Clocapramine or Clopenthixol or Clopentixol or Clothiapine or Clotiapine or Clozapine or Cyamemazine or Cyamepromazine or Dixyrazine or Droperidol or Fluanisone or Flupehenazine or Flupenthixol or Flupentixol or Fluphenazine or Fluspirilen or Fluspirilene or Haloperidol or Iloperidone or Levomepromazine or Levosulpiride or Lithium or Loxapine or Loxapinsuccinate or Lurasidone or Melperone or Mepazine or Mesoridazine or Methotrimeprazine or Molindone or Moperone or Mosapramine or Olanzapine or Oxypertine or Paliperidone or Penfluridol or Perazine or Periciazine or Pericyazine or Perospirone or Perphenazine or Pimozide or Pipamperone or Pipothiazine or Pipotiazine or Prochlorperazine or Promazine or Promethazine or Prothipendyl or Quetiapine or Remoxipiride or Reserpine or Riospirone or Risperdal or Risperidone or Seroquel or Sertindole or Stelazine or Sulpiride or Sultopride or Thiopropazate or Thioproperazine or Thioridazine or Tiospirone or Thiothixene or Tiapride or Tiotixene or Trifluoperazine or Trifluperidol or trifluoperidol or Triflupromazine or trifluperazine or Veralipride or Ziprasidone or Zotepine or Zuclopenthixol)

Indexes=BCI Timespan=2014-2016

10 **48,402** #9 OR #8 OR #7 OR #6 OR #3 OR #2

Indexes=BCI Timespan=2014-2016

9 **4,108** TS=crossover* OR TI=crossover*

Indexes=BCI Timespan=2014-2016

8 **65** TS=(randomi* Near/1 assign*) or TI=(randomi* Near/1 assign*)

Indexes=BCI Timespan=2014-2016

7 **14** TS=(randomi* Near/1 allocate*) or TI=(randomi* Near/1 allocate*)

Indexes=BCI Timespan=2014-2016

6 **10,813** #5 AND #4

Indexes=BCI Timespan=2014-2016

5 **22,480** TS=(mask* OR blind*) OR TI=(mask* OR blind*)

Indexes=BCI Timespan=2014-2016

4 **250,320** TS=(singl* OR Doubl* OR Tripl* OR Trebl*) OR TI=(singl* OR Doubl* OR Tripl* OR Trebl*)

Indexes=BCI Timespan=2014-2016

3 **43,534** TI=(randomi*) OR TS=(randomi*)

Indexes=BCI Timespan=2014-2016

2 **27,545** TS=(Randomized clinical trial*) OR TI=(Randomized clinical trial*)

Indexes=BCI Timespan=2014-2016

1 **923,561** TA=(Hominidae)

Indexes=BCI Timespan=2014-2016

3.2 Update search November 08th, 2017

Ovid MEDLINE(R) Epub Ahead of Print, In-Process & Other Non-Indexed Citations, Ovid MEDLINE(R) Daily and Ovid MEDLINE(R) <1946 to Present> 08-11-17

1 Benperidol/ or Chlorpromazine/ or Clopenthixol/ or Clozapine/ or Flupenthixol/ or Fluphenazine/ or Fluspirilene/ or Haloperidol/ or Methotrimeprazine/ or Loxapine/ or Molindone/ or Penfluridol/ or Perazine/ or Perphenazine/ or Pimozide/ or Risperidone/ or Sulpiride/ or Thioridazine/ or Thiothixene/ or Trifluoperazine/ or Clopenthixol/ (59220)
2 (Amisulpride or Aripiprazole or Asenapine or Benperidol or Brexpiprazole or Cariprazine or Chlorpromazine or Clopenthixol or Clozapine or Flupenthixol or Fluphenazine or Fluspirilene or Haloperidol or Iloperidone or Levomepromazine or Loxapine or Lurasidone or Molindone or Olanzapine or Paliperidone or Quetiapine or Penfluridol or Perazine or Perphenazine or Pimozide or Risperidone or Sertindole or Sulpiride or Thioridazine or Thiothixene or Trifluoperazine or Ziprasidone or Zotepine or Zuclopenthixol).tw. (66930)
3 or/1-2 (85727)
4 exp schizophrenia/ (102222)
5 exp Paranoid Disorders/ (4115)
6 schizo$.mp. (165120)
7 hebephreni$.mp. (285)
8 oligophreni$.mp. (1135)
9 psychotic$.mp. (66059)
10 psychosis.mp. (34850)
11 psychoses.mp. (21241)
12 or/4-11 (222249)
13 exp clinical trial/ (859559)
14 exp randomized controlled trials/ (124530)
15 exp cross-over studies/ (45209)
16 randomized controlled trial.pt. (498672)
17 clinical trial.pt. (548437)
18 controlled clinical trial.pt. (99309)
19 (clinic$ adj2 trial).mp. (719211)

20 (random$ adj5 control$ adj5 trial$).mp. (686492)
21 (crossover or cross-over).mp. (91835)
22 ((singl$ or double$ or trebl$ or tripl$) adj (blind$ or mask$)).mp. (231374)
23 randomi$.mp. (825577)
24 (random$ adj5 (assign$ or allocat$ or assort$ or reciev$)).mp. (228557)
25 or/13-24 (1354690)
26 3 and 12 and 25 (6843)
27 limit 26 to ed=20161117-20171108 (142)

Embase <1974 to 2017 Week 45> 08-11-17

1 Amisulpride/ or Aripiprazole/ or Asenapine/ or Benperidol/ or Brexpiprazole/ or Cariprazine/ or Chlorpromazine/ or Clopenthixol/ or Clozapine/ or Flupenthixol/ or Fluphenazine/ or Fluspirilene/ or Haloperidol/ or Iloperidone/ or Levomepromazine/ or Loxapine/ or Lurasidone/ or Molindone/ or Olanzapine/ or Paliperidone/ or Quetiapine/ or Penfluridol/ or Perazine/ or Perphenazine/ or Pimozide/ or Risperidone/ or Sertindole/ or Sulpiride/ or Thioridazine/ or Tiotixene/ or Trifluoperazine/ or Ziprasidone/ or Zotepine/ or Zuclopenthixol/ (164376)
2 (Amisulpride or Aripiprazole or Asenapine or Benperidol or Brexpiprazole or Cariprazine or Chlorpromazine or Clopenthixol or Clozapine or Flupenthixol or Fluphenazine or Fluspirilene or Haloperidol or Iloperidone or Levomepromazine or Loxapine or Lurasidone or Molindone or Olanzapine or Paliperidone or Quetiapine or Penfluridol or Perazine or Perphenazine or Pimozide or Risperidone or Sertindole or Sulpiride or Thioridazine or Thiothixene or Trifluoperazine or Ziprasidone or Zotepine or Zuclopenthixol).tw. (80449)
3 or/1-2 (170244)
4 exp schizophrenia/ (171035)
5 exp psychosis/ (261313)
6 schizo$.mp. (213225)
7 hebephreni$.mp. (942)
8 oligophreni$.mp. (1550)
9 psychotic$.mp. (48580)
10 psychosis.mp. (117286)
11 psychoses.mp. (12019)
12 or/4-11 (319625)
13 (clin$ adj2 trial).mp. (1411147)
14 ((singl$ or doubl$ or trebl$ or tripl$) adj (blind$ or mask$)).mp. (266600)
15 (random$ adj5 (assign$ or allocat$)).mp. (159704)
16 randomi$.mp. (1010534)
17 crossover.mp. (85681)
18 exp randomized-controlled-trial/ (480672)
19 exp crossover-procedure/ (54013)
20 exp randomization/ (76341)
21 or/13-20 (1984816)
22 3 and 12 and 21 (14661)

PsycINFO <1806 to October Week 5 2017> 08-11-17

1 Aripiprazole/ or Chlorpromazine/ or Clozapine/ or Fluphenazine/ or Haloperidol/ or Loxapine/ or Molindone/ or Olanzapine/ or Quetiapine/ or Perphenazine/ or Pimozide/ or Risperidone/ or Sulpiride/ or Thioridazine/ or Thiothixene/ or Trifluoperazine/ (18864)
2 (Amisulpride or Aripiprazole or Asenapine or Benperidol or Brexpiprazole or Cariprazine or Chlorpromazine or Clopenthixol or Clozapine or Flupenthixol or Fluphenazine or Fluspirilene or Haloperidol or Iloperidone or Levomepromazine or Loxapine or Lurasidone or Molindone or

Olanzapine or Paliperidone or Quetiapine or Penfluridol or Perazine or Perphenazine or Pimozide or Risperidone or Sertindole or Sulpiride or Thioridazine or Thiothixene or Trifluoperazine or Ziprasidone or Zotepine or Zuclopenthixol).tw. (30668)

3 or/1-2 (30799)
4 exp schizophrenia/ (83402)
5 exp Schizoaffective Disorder/ (2896)
6 exp schizophreniform disorder/ (339)
7 schizo$.mp. (126961)
8 exp psychosis/ (106639)
9 hebephreni$.mp. (539)
10 oligophreni$.mp. (521)
11 psychotic$.mp. (43539)
12 psychosis.mp. (49799)
13 psychoses.mp. (15056)
14 or/4-13 (175047)
15 ((singl$ or doubl$ or trebl$ or tripl$) adj (blind$ or mask$)).mp. (23680)
16 (random$ adj5 (assign$ or allocat$)).mp. (38788)
17 randomi$.mp. (70694)
18 crossover.mp. (6597)
19 or/15-18 (112844)
20 3 and 14 and 19 (2845)

Cochrane Library 08-11-17

#1 MeSH descriptor: [Benperidol] this term only
#2 MeSH descriptor: [Chlorpromazine] this term only
#3 MeSH descriptor: [Clopenthixol] this term only
#4 MeSH descriptor: [Clozapine] this term only
#5 MeSH descriptor: [Flupenthixol] this term only
#6 MeSH descriptor: [Fluphenazine] this term only
#7 MeSH descriptor: [Fluspirilene] this term only
#8 MeSH descriptor: [Haloperidol] this term only
#9 MeSH descriptor: [Methotrimeprazine] this term only
#10 MeSH descriptor: [Loxapine] this term only
#11 MeSH descriptor: [Molindone] this term only
#12 MeSH descriptor: [Penfluridol] this term only
#13 MeSH descriptor: [Perazine] this term only
#14 MeSH descriptor: [Perphenazine] this term only
#15 MeSH descriptor: [Pimozide] this term only
#16 MeSH descriptor: [Risperidone] this term only
#17 MeSH descriptor: [Sulpiride] this term only
#18 MeSH descriptor: [Thioridazine] this term only
#19 MeSH descriptor: [Thiothixene] this term only
#20 MeSH descriptor: [Trifluoperazine] this term only
#21 (Amisulpride or Aripiprazole or Asenapine or Benperidol or Brexpiprazole or Cariprazine or Chlorpromazine or Clopenthixol or Clozapine or Flupenthixol or Fluphenazine or Fluspirilene or Haloperidol or Iloperidone or Levomepromazine or Loxapine or Lurasidone or Molindone or Olanzapine or Paliperidone or Quetiapine or Penfluridol or Perazine or Perphenazine or Pimozide or Risperidone or Sertindole or Sulpiride or Thioridazine or Thiothixene or Trifluoperazine or Ziprasidone or Zotepine or Zuclopenthixol):ti,ab,kw (Word variations have been searched)
#22 #1 or #2 or #3 or #4 or #5 or #6 or #7 or #8 or #9 or #10 or #11 or #12 or #13 or #14 or #15 or #16 or #17 or #18 or #19 or #20 or #21

#23 MeSH descriptor: [Schizophrenia] explode all trees
#24 MeSH descriptor: [Paranoid Disorders] explode all trees
#25 (schizo* or hebephrenic* or oligophreni* or psychotic* or psychosis or psychoses):ti,ab,kw (Word variations have been searched)
#26 #23 or #24 or #25
#27 #22 and #26 in Trials = 6132

Pubmed 08-11-17

#10 Search (#8 and #9) 146
#9 Search ("2016/11/17"[Date - Entrez] : "2017/11/08"[Date - Entrez]) 1087861
#8 Search ((#3 and #6 and #7)) 5365
#7 Search (((randomized controlled trial[pt] OR controlled clinical trial[pt] OR randomized[tiab] OR placebo[tiab] OR clinical trials as topic[mesh:noexp] OR randomly[tiab] OR trial[ti] NOT (animals[mh] NOT humans [mh])))) 1033765
#6 Search ((#4 or #5)) 199259
#5 Search ((("Schizophrenia"[Mesh]) OR "Psychotic Disorders"[Mesh])) 131315
#4 Search (((schizo*[Title/Abstract] OR hebephrenic*[Title/Abstract] OR oligophreni*[Title/Abstract] OR psychotic*[Title/Abstract] OR psychosis[Title/Abstract] OR psychoses[Title/Abstract]))) 170390
#3 Search ((#1 or #2)) 81037
#2 Search (((Amisulpride[Title/Abstract] OR Aripiprazole[Title/Abstract] OR Asenapine[Title/Abstract] OR Benperidol[Title/Abstract] OR Brexpiprazole[Title/Abstract] OR Cariprazine[Title/Abstract] OR Chlorpromazine[Title/Abstract] OR Clopenthixol[Title/Abstract] OR Clozapine[Title/Abstract] OR Flupenthixol[Title/Abstract] OR Fluphenazine[Title/Abstract] OR Fluspirilene[Title/Abstract] OR Haloperidol[Title/Abstract] OR Iloperidone[Title/Abstract] OR Levomepromazine[Title/Abstract] OR Loxapine[Title/Abstract] OR Lurasidone[Title/Abstract] OR Molindone[Title/Abstract] OR Olanzapine[Title/Abstract] OR Paliperidone[Title/Abstract] OR Quetiapine[Title/Abstract] OR Penfluridol[Title/Abstract] OR Perazine[Title/Abstract] OR Perphenazine[Title/Abstract] OR Pimozide[Title/Abstract] OR Risperidone[Title/Abstract] OR Sertindole[Title/Abstract] OR Sulpiride[Title/Abstract] OR Thioridazine[Title/Abstract] OR Thiothixene[Title/Abstract] OR Trifluoperazine[Title/Abstract] OR Ziprasidone[Title/Abstract] OR Zotepine[Title/Abstract] OR Zuclopenthixol[Title/Abstract]))) 65192
#1 Search (("Brexpiprazole" [Supplementary Concept] or "sultopride" [Supplementary Concept] or "aripiprazole" [Supplementary Concept] or "Asenapine" [Supplementary Concept] or "Benperidol"[Mesh] or "cariprazine" [Supplementary Concept] or "Chlorpromazine"[Mesh] or "Clopenthixol"[Mesh] or "Clozapine"[Mesh] or "Flupenthixol"[Mesh] or "Fluphenazine"[Mesh] or "Fluspirilene"[Mesh] or "Haloperidol"[Mesh] or "iloperidone" [Supplementary Concept] or "Methotrimeprazine"[Mesh] or "Loxapine"[Mesh] or "lurasidone" [Supplementary Concept] or "Molindone"[Mesh] or "olanzapine" [Supplementary Concept] or "paliperidone" [Supplementary Concept] or "quetiapine" [Supplementary Concept] or "Penfluridol"[Mesh] or "Perazine"[Mesh] or "Perphenazine"[Mesh] or "Pimozide"[Mesh] or "Risperidone"[Mesh] or "sertindole" [Supplementary Concept] or "Sulpiride"[Mesh] or "Thioridazine"[Mesh] or "Thiothixene"[Mesh] or "Trifluoperazine"[Mesh] or "ziprasidone" [Supplementary Concept] or "zotepine" [Supplementary Concept] or "Clopenthixol"[Mesh])) 60649

Biosis 08-11-17

# 12 2,225 #11 AND #10 AND #9
Indexes=BCI Timespan=All years
# 11 69,571 **TOPIC:** (Amisulpride or Aripiprazole or Asenapine or Benperidol or Brexpiprazole or Cariprazine or Chlorpromazine or Clopenthixol or Clozapine or Flupenthixol or Fluphenazine or

Fluspirilene or Haloperidol or Iloperidone or Levomepromazine or Loxapine or Lurasidone or Molindone or Olanzapine or Paliperidone or Quetiapine or Penfluridol or Perazine or Perphenazine or Pimozide or Risperidone or Sertindole or Sulpiride or Thioridazine or Thiothixene or Trifluoperazine or Ziprasidone or Zotepine or Zuclopenthixol)
Indexes=BCI Timespan=All years
# 10 151,674 **TOPIC:** (schizo* or hebephrenic* OR oligophreni* OR psychotic* OR psychosis OR psychoses)
Indexes=BCI Timespan=All years
# 9 380,429 #8 OR #7 OR #6 OR #5 OR #2 OR #1
Indexes=BCI Timespan=All years
# 8 39,548 **TOPIC:** (crossover*)
Indexes=BCI Timespan=All years
# 7 462 **TOPIC:** (randomi* Near/1 assign*)
Indexes=BCI Timespan=All years
# 6 79 **TOPIC:** (randomi* Near/1 allocate*)
Indexes=BCI Timespan=All years
# 5 121,630 #4 AND #3
Indexes=BCI Timespan=All years
# 4 223,836 **TOPIC:** (mask* OR blind*)
Indexes=BCI Timespan=All years
# 3 2,240,458 **TOPIC:** (singl* OR Doubl* OR Tripl* OR Trebl*)
Indexes=BCI Timespan=All years
# 2 313,760 **TOPIC:** (randomi*)
Indexes=BCI Timespan=All years
# 1 163,861 **TOPIC:** (Randomized clinical trial*)
Indexes=BCI Timespan=All years

Clinicaltrials.gov 09-11-17

First posted 18/11/16-09/11/17
Intervention Amisulpride / Condition Schizophrenia / other terms Random = 0
Intervention Aripiprazole / Condition Schizophrenia / other terms Random = 3
Intervention Asenapine / Condition Schizophrenia / other terms Random = 0
Intervention Benperidol / Condition Schizophrenia / other terms Random = 0
Intervention Brexpiprazole / Condition Schizophrenia / other terms Random = 0
Intervention Cariprazine / Condition Schizophrenia / other terms Random = 0
Intervention Chlorpromazine / Condition Schizophrenia / other terms Random = 0
Intervention Clopenthixol / Condition Schizophrenia / other terms Random = 0
Intervention Clozapine / Condition Schizophrenia / other terms Random = 2
Intervention Flupenthixol / Condition Schizophrenia / other terms Random = 0
Intervention Fluphenazine / Condition Schizophrenia / other terms Random = 0
Intervention Fluspirilene / Condition Schizophrenia / other terms Random = 0
Intervention Haloperidol / Condition Schizophrenia / other terms Random = 1
Intervention Iloperidone / Condition Schizophrenia / other terms Random = 0
Intervention Levomepromazine / Condition Schizophrenia / other terms Random = 0
Intervention Loxapine / Condition Schizophrenia / other terms Random = 0
Intervention Lurasidone / Condition Schizophrenia / other terms Random = 1
Intervention Molindone / Condition Schizophrenia / other terms Random = 0
Intervention Olanzapine / Condition Schizophrenia / other terms Random = 3
Intervention Paliperidone / Condition Schizophrenia / other terms Random = 1
Intervention Quetiapine / Condition Schizophrenia / other terms Random = 0
Intervention Penfluridol / Condition Schizophrenia / other terms Random = 0
Intervention Perazine / Condition Schizophrenia / other terms Random = 0

Intervention Perphenazine / Condition Schizophrenia / other terms Random = 0
Intervention Pimozide / Condition Schizophrenia / other terms Random = 0
Intervention Risperidone / Condition Schizophrenia / other terms Random = 5
Intervention Sertindole / Condition Schizophrenia / other terms Random = 0
Intervention Sulpiride / Condition Schizophrenia / other terms Random = 0
Intervention Thioridazine / Condition Schizophrenia / other terms Random = 0
Intervention Thiothixene / Condition Schizophrenia / other terms Random = 0
Intervention Trifluoperazine / Condition Schizophrenia / other terms Random = 0
Intervention Ziprasidone / Condition Schizophrenia / other terms Random = 0
Intervention Zotepine / Condition Schizophrenia / other terms Random = 0
Intervention Zuclopenthixol / Condition Schizophrenia / other terms Random = 0
Intervention Amisulpride / Condition Schizophreniform / other terms Random = 0
Intervention Aripiprazole / Condition Schizophreniform / other terms Random = 0
Intervention Asenapine / Condition Schizophreniform / other terms Random = 0
Intervention Benperidol / Condition Schizophreniform / other terms Random = 0
Intervention Brexpiprazole / Condition Schizophreniform / other terms Random = 0
Intervention Cariprazine / Condition Schizophreniform / other terms Random = 0
Intervention Chlorpromazine / Condition Schizophreniform / other terms Random = 0
Intervention Clopenthixol / Condition Schizophreniform / other terms Random = 0
Intervention Clozapine / Condition Schizophreniform / other terms Random = 0
Intervention Flupenthixol / Condition Schizophreniform / other terms Random = 0
Intervention Fluphenazine / Condition Schizophreniform / other terms Random = 0
Intervention Fluspirilene / Condition Schizophreniform / other terms Random = 0
Intervention Haloperidol / Condition Schizophreniform / other terms Random = 0
Intervention Iloperidone / Condition Schizophreniform / other terms Random = 0
Intervention Levomepromazine / Condition Schizophreniform / other terms Random = 0
Intervention Loxapine / Condition Schizophreniform / other terms Random = 0
Intervention Lurasidone / Condition Schizophreniform / other terms Random = 0
Intervention Molindone / Condition Schizophreniform / other terms Random = 0
Intervention Olanzapine / Condition Schizophreniform / other terms Random = 1
Intervention Paliperidone / Condition Schizophreniform / other terms Random = 0
Intervention Quetiapine / Condition Schizophreniform / other terms Random = 0
Intervention Penfluridol / Condition Schizophreniform / other terms Random = 0
Intervention Perazine / Condition Schizophreniform / other terms Random = 0
Intervention Perphenazine / Condition Schizophreniform / other terms Random = 0
Intervention Pimozide / Condition Schizophreniform / other terms Random = 0
Intervention Risperidone / Condition Schizophreniform / other terms Random = 0
Intervention Sertindole / Condition Schizophreniform / other terms Random = 0
Intervention Sulpiride / Condition Schizophreniform / other terms Random = 0
Intervention Thioridazine / Condition Schizophreniform / other terms Random = 0
Intervention Thiothixene / Condition Schizophreniform / other terms Random = 0
Intervention Trifluoperazine / Condition Schizophreniform / other terms Random = 0
Intervention Ziprasidone / Condition Schizophreniform / other terms Random = 0
Intervention Zotepine / Condition Schizophreniform / other terms Random = 0
Intervention Zuclopenthixol / Condition Schizophreniform / other terms Random = 0
Intervention Amisulpride / Condition Schizoaffective / other terms Random = 0
Intervention Aripiprazole / Condition Schizoaffective / other terms Random = 0
Intervention Asenapine / Condition Schizoaffective / other terms Random = 0
Intervention Benperidol / Condition Schizoaffective / other terms Random = 0
Intervention Brexpiprazole / Condition Schizoaffective / other terms Random = 0
Intervention Cariprazine / Condition Schizoaffective / other terms Random = 0
Intervention Chlorpromazine / Condition Schizoaffective / other terms Random = 0
Intervention Clopenthixol / Condition Schizoaffective / other terms Random = 0

Intervention Clozapine / Condition Schizoaffective / other terms Random = 0
Intervention Flupenthixol / Condition Schizoaffective / other terms Random = 0
Intervention Fluphenazine / Condition Schizoaffective / other terms Random = 0
Intervention Fluspirilene / Condition Schizoaffective / other terms Random = 0
Intervention Haloperidol / Condition Schizoaffective / other terms Random = 0
Intervention Iloperidone / Condition Schizoaffective / other terms Random = 0
Intervention Levomepromazine / Condition Schizoaffective / other terms Random = 0
Intervention Loxapine / Condition Schizoaffective / other terms Random = 0
Intervention Lurasidone / Condition Schizoaffective / other terms Random = 0
Intervention Molindone / Condition Schizoaffective / other terms Random = 0
Intervention Olanzapine / Condition Schizoaffective / other terms Random = 0
Intervention Paliperidone / Condition Schizoaffective / other terms Random = 0
Intervention Quetiapine / Condition Schizoaffective / other terms Random = 0
Intervention Penfluridol / Condition Schizoaffective / other terms Random = 0
Intervention Perazine / Condition Schizoaffective / other terms Random = 0
Intervention Perphenazine / Condition Schizoaffective / other terms Random = 0
Intervention Pimozide / Condition Schizoaffective / other terms Random = 0
Intervention Risperidone / Condition Schizoaffective / other terms Random = 0
Intervention Sertindole / Condition Schizoaffective / other terms Random = 0
Intervention Sulpiride / Condition Schizoaffective / other terms Random = 0
Intervention Thioridazine / Condition Schizoaffective / other terms Random = 0
Intervention Thiothixene / Condition Schizoaffective / other terms Random = 0
Intervention Trifluoperazine / Condition Schizoaffective / other terms Random = 0
Intervention Ziprasidone / Condition Schizoaffective / other terms Random = 0
Intervention Zotepine / Condition Schizoaffective / other terms Random = 0
Intervention Zuclopenthixol / Condition Schizoaffective / other terms Random = 0
Intervention Amisulpride / Condition Psychosis / other terms Random = 0
Intervention Aripiprazole / Condition Psychosis / other terms Random = 5
Intervention Asenapine / Condition Psychosis / other terms Random = 0
Intervention Benperidol / Condition Psychosis / other terms Random = 0
Intervention Brexpiprazole / Condition Psychosis / other terms Random = 6
Intervention Cariprazine / Condition Psychosis / other terms Random = 0
Intervention Chlorpromazine / Condition Psychosis / other terms Random = 1
Intervention Clopenthixol / Condition Psychosis / other terms Random = 0
Intervention Clozapine / Condition Psychosis / other terms Random = 2
Intervention Flupenthixol / Condition Psychosis / other terms Random = 0
Intervention Fluphenazine / Condition Psychosis / other terms Random = 0
Intervention Fluspirilene / Condition Psychosis / other terms Random = 0
Intervention Haloperidol / Condition Psychosis / other terms Random = 5
Intervention Iloperidone / Condition Psychosis / other terms Random = 0
Intervention Levomepromazine / Condition Psychosis / other terms Random = 0
Intervention Loxapine / Condition Psychosis / other terms Random = 0
Intervention Lurasidone / Condition Psychosis / other terms Random = 2
Intervention Molindone / Condition Psychosis / other terms Random = 0
Intervention Olanzapine / Condition Psychosis / other terms Random = 4
Intervention Paliperidone / Condition Psychosis / other terms Random = 1
Intervention Quetiapine / Condition Psychosis / other terms Random = 3
Intervention Penfluridol / Condition Psychosis / other terms Random = 0
Intervention Perazine / Condition Psychosis / other terms Random = 0
Intervention Perphenazine / Condition Psychosis / other terms Random = 0
Intervention Pimozide / Condition Psychosis / other terms Random = 0
Intervention Risperidone / Condition Psychosis / other terms Random = 6
Intervention Sertindole / Condition Psychosis / other terms Random = 0

Intervention Sulpiride / Condition Psychosis / other terms Random = 0
Intervention Thioridazine / Condition Psychosis / other terms Random = 0
Intervention Thiothixene / Condition Psychosis / other terms Random = 0
Intervention Trifluoperazine / Condition Psychosis / other terms Random = 0
Intervention Ziprasidone / Condition Psychosis / other terms Random = 0
Intervention Zotepine / Condition Psychosis / other terms Random = 0
Intervention Zuclopenthixol / Condition Psychosis / other terms Random = 0
Total = 52

WHO ICTRP 09-11-17

Amisulpride and schizo* and random* = 0
Aripiprazole and schizo* and random* = 4
Asenapine and schizo* and random* = 0
Benperidol and schizo* and random* = 0
Brexpiprazole and schizo* and random* = 1
Cariprazine and schizo* and random* = 0
Chlorpromazine and schizo* and random* = 0
Clopenthixol and schizo* and random* = 0
Clozapine and schizo* and random* = 4
Flupenthixol and schizo* and random* = 1
Fluphenazine and schizo* and random* = 1
Fluspirilene and schizo* and random* = 0
Haloperidol and schizo* and random* = 0
Iloperidone and schizo* and random* = 0
Levomepromazine and schizo* and random* = 0
Loxapine and schizo* and random* = 0
Lurasidone and schizo* and random* = 2
Molindone and schizo* and random* = 0
Olanzapine and schizo* and random* = 5
Paliperidone and schizo* and random* = 3
Quetiapine and schizo* and random* = 2
Penfluridol and schizo* and random* = 0
Perazine and schizo* and random* = 0
Perphenazine and schizo* and random* = 0
Pimozide and schizo* and random* = 0
Risperidone and schizo* and random* = 5
Sertindole and schizo* and random* = 0
Sulpiride and schizo* and random* = 0
Thioridazine and schizo* and random* = 0
Thiothixene and schizo* and random* = 0
Trifluoperazine and schizo* and random* = 0
Ziprasidone and schizo* and random* = 0
Zotepine and schizo* and random* = 0
Zuclopenthixol and schizo* and random* = 1
Amisulpride and psycho* and random* = 0
Aripiprazole and psycho* and random* = 1
Asenapine and psycho* and random* = 0
Benperidol and psycho* and random* = 0
Brexpiprazole and psycho* and random*= 0
Cariprazine and psycho* and random* = 0
Chlorpromazine and psycho* and random* = 0
Clopenthixol and psycho* and random* = 0

Clozapine and psycho* and random* = 0
Flupenthixol and psycho* and random* = 3
Fluphenazine and psycho* and random* = 1
Fluspirilene and psycho* and random* = 0
Haloperidol and psycho* and random* = 2
Iloperidone and psycho* and random* = 0
Levomepromazine and psycho* and random* = 0
Loxapine and psycho* and random* = 1
Lurasidone and psycho* and random* = 1
Molindone and psycho* and random* = 0
Olanzapine and psycho* and random* = 2
Paliperidone and psycho* and random* = 1
Quetiapine and psycho* and random* = 1
Penfluridol and psycho* and random* = 0
Perazine and psycho* and random* = 0
Perphenazine and psycho* and random* = 0
Pimozide and psycho* and random* = 0
Risperidone and psycho* and random* = 1
Sertindole and psycho* and random* = 0
Sulpiride and psycho* and random* = 0
Thioridazine and psycho* and random* = 0
Thiothixene and psycho* and random* = 0
Trifluoperazine and psycho* and random* = 0
Ziprasidone and psycho* and random* = 0
Zotepine and psycho* and random* = 0
Zuclopenthixol and psycho* and random* = 1
Total = 44

3.3 Update search January 08th, 2019

Ovid MEDLINE(R) Epub Ahead of Print, In-Process & Other Non-Indexed Citations, Ovid MEDLINE(R) Daily and Ovid MEDLINE(R) <1946 to Present> 08-01-19

1 Benperidol/ or Chlorpromazine/ or Clopenthixol/ or Clozapine/ or Flupenthixol/ or Fluphenazine/ or Fluspirilene/ or Haloperidol/ or Methotrimeprazine/ or Loxapine/ or Molindone/ or Penfluridol/ or Perazine/ or Perphenazine/ or Pimozide/ or Risperidone/ or Sulpiride/ or Thioridazine/ or Thiothixene/ or Trifluoperazine/ or Clopenthixol/ (56275)
2 (Amisulpride or Aripiprazole or Asenapine or Benperidol or Cariprazine or Chlorpromazine or Clopenthixol or Clozapine or Flupenthixol or Fluphenazine or Fluspirilene or Haloperidol or Iloperidone or Levomepromazine or Loxapine or Lurasidone or Molindone or Olanzapine or Paliperidone or Quetiapine or Penfluridol or Perazine or Perphenazine or Pimozide or Risperidone or Sertindole or Sulpiride or Thioridazine or Thiothixene or Trifluoperazine or Ziprasidone or Zotepine or Zuclopenthixol).tw. (63499)
3 or/1-2 (81540)
4 exp schizophrenia/ (99304)
5 exp Paranoid Disorders/ (3997)
6 schizo$.mp. (158756)
7 hebephreni$.mp. (273)
8 oligophreni$.mp. (1104)
9 psychotic$.mp. (63780)
10 psychosis.mp. (33641)
11 psychoses.mp. (20460)
12 or/4-11 (214000)
13 exp clinical trial/ (814440)

14 exp randomized controlled trials/ (122359)
15 exp cross-over studies/ (44247)
16 randomized controlled trial.pt. (473543)
17 clinical trial.pt. (513891)
18 controlled clinical trial.pt. (92824)
19 (clinic$ adj2 trial).mp. (677857)
20 (random$ adj5 control$ adj5 trial$).mp. (659625)
21 (crossover or cross-over).mp. (87540)
22 ((singl$ or double$ or trebl$ or tripl$) adj (blind$ or mask$)).mp. (218786)
23 randomi$.mp. (794630)
24 (random$ adj5 (assign$ or allocat$ or assort$ or reciev$)).mp. (221727)
25 or/13-24 (1302166)
26 3 and 12 and 25 (6491)
27 limit 26 to ed=20171108-20190108 (223)

Embase <1946 to January 07, 2019> 08-01-19

1 Amisulpride/ or Aripiprazole/ or Asenapine/ or Benperidol/ or Brexpiprazole/ or Cariprazine/ or Chlorpromazine/ or Clopenthixol/ or Clozapine/ or Flupenthixol/ or Fluphenazine/ or Fluspirilene/ or Haloperidol/ or Iloperidone/ or Levomepromazine/ or Loxapine/ or Lurasidone/ or Molindone/ or Olanzapine/ or Paliperidone/ or Quetiapine/ or Penfluridol/ or Perazine/ or Perphenazine/ or Pimozide/ or Risperidone/ or Sertindole/ or Sulpiride/ or Thioridazine/ or Tiotixene/ or Trifluoperazine/ or Ziprasidone/ or Zotepine/ or Zuclopenthixol/ (159603)
2 (Amisulpride or Aripiprazole or Asenapine or Benperidol or Brexpiprazole or Cariprazine or Chlorpromazine or Clopenthixol or Clozapine or Flupenthixol or Fluphenazine or Fluspirilene or Haloperidol or Iloperidone or Levomepromazine or Loxapine or Lurasidone or Molindone or Olanzapine or Paliperidone or Quetiapine or Penfluridol or Perazine or Perphenazine or Pimozide or Risperidone or Sertindole or Sulpiride or Thioridazine or Thiothixene or Trifluoperazine or Ziprasidone or Zotepine or Zuclopenthixol).tw. (79613)
3 or/1-2 (165326)
4 exp schizophrenia/ (166874)
5 exp psychosis/ (257141)
6 schizo$.mp. (213154)
7 hebephreni$.mp. (877)
8 oligophreni$.mp. (1268)
9 psychotic$.mp. (50503)
10 psychosis.mp. (116097)
11 psychoses.mp. (9765)
12 or/4-11 (317083)
13 (clin$ adj2 trial).mp. (1456592)
14 ((singl$ or doubl$ or trebl$ or tripl$) adj (blind$ or mask$)).mp. (281503)
15 (random$ adj5 (assign$ or allocat$)).mp. (172480)
16 randomi$.mp. (1095115)
17 crossover.mp. (90541)
18 exp randomized-controlled-trial/ (529503)
19 exp crossover-procedure/ (57710)
20 exp randomization/ (80721)
21 or/13-20 (2099842)
22 3 and 12 and 21 (14749)
23 2017??.em. (1929949)
24 2018??.em. (2406120)
25 "201901".em. (60646)
26 or/23-25 (4396715)

27 22 and 26 (1921)

PsycINFO <1806 to December Week 5 2018> 08-01-19

1 Aripiprazole/ or Chlorpromazine/ or Clozapine/ or Fluphenazine/ or Haloperidol/ or Loxapine/ or Molindone/ or Olanzapine/ or Quetiapine/ or Perphenazine/ or Pimozide/ or Risperidone/ or Sulpiride/ or Thioridazine/ or Thiothixene/ or Trifluoperazine/ (19269)
2 (Amisulpride or Aripiprazole or Asenapine or Benperidol or Cariprazine or Chlorpromazine or Clopenthixol or Clozapine or Flupenthixol or Fluphenazine or Fluspirilene or Haloperidol or Iloperidone or Levomepromazine or Loxapine or Lurasidone or Molindone or Olanzapine or Paliperidone or Quetiapine or Penfluridol or Perazine or Perphenazine or Pimozide or Risperidone or Sertindole or Sulpiride or Thioridazine or Thiothixene or Trifluoperazine or Ziprasidone or Zotepine or Zuclopenthixol).tw. (31517)
3 or/1-2 (31648)
4 exp schizophrenia/ (85848)
5 exp Schizoaffective Disorder/ (2962)
6 exp schizophreniform disorder/ (343)
7 schizo$.mp. (131634)
8 exp psychosis/ (110002)
9 hebephreni$.mp. (543)
10 oligophreni$.mp. (522)
11 psychotic$.mp. (45235)
12 psychosis.mp. (52047)
13 psychoses.mp. (15190)
14 or/4-13 (181282)
15 ((singl$ or doubl$ or trebl$ or tripl$) adj (blind$ or mask$)).mp. (24705)
16 (random$ adj5 (assign$ or allocat$)).mp. (41339)
17 randomi$.mp. (77268)
18 crossover.mp. (6986)
19 or/15-18 (121521)
20 3 and 14 and 19 (2944)

Cochrane Library 09-01-19

#1 MeSH descriptor: [Benperidol] this term only
#2 MeSH descriptor: [Chlorpromazine] this term only
#3 MeSH descriptor: [Clopenthixol] this term only
#4 MeSH descriptor: [Clozapine] this term only
#5 MeSH descriptor: [Flupenthixol] this term only
#6 MeSH descriptor: [Fluphenazine] this term only
#7 MeSH descriptor: [Fluspirilene] this term only
#8 MeSH descriptor: [Haloperidol] this term only
#9 MeSH descriptor: [Methotrimeprazine] this term only
#10 MeSH descriptor: [Loxapine] this term only
#11 MeSH descriptor: [Molindone] this term only
#12 MeSH descriptor: [Penfluridol] this term only
#13 MeSH descriptor: [Perazine] this term only
#14 MeSH descriptor: [Perphenazine] this term only
#15 MeSH descriptor: [Pimozide] this term only
#16 MeSH descriptor: [Risperidone] this term only
#17 MeSH descriptor: [Sulpiride] this term only
#18 MeSH descriptor: [Thioridazine] this term only

#19 MeSH descriptor: [Thiothixene] this term only
#20 MeSH descriptor: [Trifluoperazine] this term only
#21 (Amisulpride or Aripiprazole or Asenapine or Benperidol or Brexpiprazole or Cariprazine or Chlorpromazine or Clopenthixol or Clozapine or Flupenthixol or Fluphenazine or Fluspirilene or Haloperidol or Iloperidone or Levomepromazine or Loxapine or Lurasidone or Molindone or Olanzapine or Paliperidone or Quetiapine or Penfluridol or Perazine or Perphenazine or Pimozide or Risperidone or Sertindole or Sulpiride or Thioridazine or Thiothixene or Trifluoperazine or Ziprasidone or Zotepine or Zuclopenthixol):ti,ab,kw (Word variations have been searched)
#22 #1 or #2 or #3 or #4 or #5 or #6 or #7 or #8 or #9 or #10 or #11 or #12 or #13 or #14 or #15 or #16 or #17 or #18 or #19 or #20 or #21
#23 MeSH descriptor: [Schizophrenia] explode all trees
#24 MeSH descriptor: [Paranoid Disorders] explode all trees
#25 (schizo* or hebephrenic* or oligophreni* or psychotic* or psychosis or psychoses):ti,ab,kw (Word variations have been searched)
#26 #23 or #24 or #25
#27 #22 and #26 in Trials with Cochrane Library publication date from Nov 2017 to Jan 2019, in Trials= 2151

Pubmed 09-01-19

#10 Search (#8 and #9) 172
#9 Search ("2017/11/08"[Date - Entrez] : "2019/01/08"[Date - Entrez])
#8 Search ((#3 and #6 and #7))
#7 Search (((randomized controlled trial[pt] OR controlled clinical trial[pt] OR randomized[tiab] OR placebo[tiab] OR clinical trials as topic[mesh:noexp] OR randomly[tiab] OR trial[ti] NOT (animals[mh] NOT humans [mh]))))
#6 Search ((#4 or #5))
#5 Search ((("Schizophrenia"[Mesh]) OR "Psychotic Disorders"[Mesh]))
#4 Search (((schizo*[Title/Abstract] OR hebephrenic*[Title/Abstract] OR oligophreni*[Title/Abstract] OR psychotic*[Title/Abstract] OR psychosis[Title/Abstract] OR psychoses[Title/Abstract])))
#3 Search ((#1 or #2))
#2 Search (((Amisulpride[Title/Abstract] OR Aripiprazole[Title/Abstract] OR Asenapine[Title/Abstract] OR Benperidol[Title/Abstract] OR Brexpiprazole[Title/Abstract] OR Cariprazine[Title/Abstract] OR Chlorpromazine[Title/Abstract] OR Clopenthixol[Title/Abstract] OR Clozapine[Title/Abstract] OR Flupenthixol[Title/Abstract] OR Fluphenazine[Title/Abstract] OR Fluspirilene[Title/Abstract] OR Haloperidol[Title/Abstract] OR Iloperidone[Title/Abstract] OR Levomepromazine[Title/Abstract] OR Loxapine[Title/Abstract] OR Lurasidone[Title/Abstract] OR Molindone[Title/Abstract] OR Olanzapine[Title/Abstract] OR Paliperidone[Title/Abstract] OR Quetiapine[Title/Abstract] OR Penfluridol[Title/Abstract] OR Perazine[Title/Abstract] OR Perphenazine[Title/Abstract] OR Pimozide[Title/Abstract] OR Risperidone[Title/Abstract] OR Sertindole[Title/Abstract] OR Sulpiride[Title/Abstract] OR Thioridazine[Title/Abstract] OR Thiothixene[Title/Abstract] OR Trifluoperazine[Title/Abstract] OR Ziprasidone[Title/Abstract] OR Zotepine[Title/Abstract] OR Zuclopenthixol[Title/Abstract])))
#1 Search (("Brexpiprazole" [Supplementary Concept] or "sultopride" [Supplementary Concept] or "aripiprazole" [Supplementary Concept] or "Asenapine" [Supplementary Concept] or "Benperidol"[Mesh] or "cariprazine" [Supplementary Concept] or "Chlorpromazine"[Mesh] or "Clopenthixol"[Mesh] or "Clozapine"[Mesh] or "Flupenthixol"[Mesh] or "Fluphenazine"[Mesh] or "Fluspirilene"[Mesh] or "Haloperidol"[Mesh] or "iloperidone" [Supplementary Concept] or "Methotrimeprazine"[Mesh] or "Loxapine"[Mesh] or "lurasidone" [Supplementary Concept] or "Molindone"[Mesh] or "olanzapine" [Supplementary Concept] or "paliperidone" [Supplementary Concept] or "quetiapine" [Supplementary Concept] or "Penfluridol"[Mesh] or "Perazine"[Mesh]

or "Perphenazine"[Mesh] or "Pimozide"[Mesh] or "Risperidone"[Mesh] or "sertindole" [Supplementary Concept] or "Sulpiride"[Mesh] or "Thioridazine"[Mesh] or "Thiothixene"[Mesh] or "Trifluoperazine"[Mesh] or "ziprasidone" [Supplementary Concept] or "zotepine" [Supplementary Concept] or "Clopenthixol"[Mesh]))

Biosis 08-01-19

# 12 2302 #11 AND #10 AND #9
Indexes=BCI Timespan=All years
# 11 **TOPIC:** (Amisulpride or Aripiprazole or Asenapine or Benperidol or Brexpiprazole or Cariprazine or Chlorpromazine or Clopenthixol or Clozapine or Flupenthixol or Fluphenazine or Fluspirilene or Haloperidol or Iloperidone or Levomepromazine or Loxapine or Lurasidone or Molindone or Olanzapine or Paliperidone or Quetiapine or Penfluridol or Perazine or Perphenazine or Pimozide or Risperidone or Sertindole or Sulpiride or Thioridazine or Thiothixene or Trifluoperazine or Ziprasidone or Zotepine or Zuclopenthixol)
Indexes=BCI Timespan=All years
# 10 **TOPIC:** (schizo* or hebephrenic* OR oligophreni* OR psychotic* OR psychosis OR psychoses)
Indexes=BCI Timespan=All years
# 9 #8 OR #7 OR #6 OR #5 OR #2 OR #1
Indexes=BCI Timespan=All years
# 8 **TOPIC:** (crossover*)
Indexes=BCI Timespan=All years
# 7 **TOPIC:** (randomi* Near/1 assign*)
Indexes=BCI Timespan=All years
# 6 **TOPIC:** (randomi* Near/1 allocate*)
Indexes=BCI Timespan=All years
# 5 #4 AND #3
Indexes=BCI Timespan=All years
# 4 **TOPIC:** (mask* OR blind*)
Indexes=BCI Timespan=All years
# 3 **TOPIC:** (singl* OR Doubl* OR Tripl* OR Trebl*)
Indexes=BCI Timespan=All years
# 2 **TOPIC:** (randomi*)
Indexes=BCI Timespan=All years
# 1 **TOPIC:** (Randomized clinical trial*)
Indexes=BCI Timespan=All years

Clinicaltrials.gov 10-01-19

First posted 09/11/17-10/01/19
Intervention Amisulpride / Condition Schizophrenia / other terms Random = 1
Intervention Aripiprazole / Condition Schizophrenia / other terms Random = 5
Intervention Asenapine / Condition Schizophrenia / other terms Random = 1
Intervention Benperidol / Condition Schizophrenia / other terms Random = 0
Intervention Brexpiprazole / Condition Schizophrenia / other terms Random = 2
Intervention Cariprazine / Condition Schizophrenia / other terms Random = 1
Intervention Chlorpromazine / Condition Schizophrenia / other terms Random = 0
Intervention Clopenthixol / Condition Schizophrenia / other terms Random = 0
Intervention Clozapine / Condition Schizophrenia / other terms Random = 3
Intervention Flupenthixol / Condition Schizophrenia / other terms Random = 0
Intervention Fluphenazine / Condition Schizophrenia / other terms Random = 0

Intervention Fluspirilene / Condition Schizophrenia / other terms Random = 0
Intervention Haloperidol / Condition Schizophrenia / other terms Random = 0
Intervention Iloperidone / Condition Schizophrenia / other terms Random = 1
Intervention Levomepromazine / Condition Schizophrenia / other terms Random = 0
Intervention Loxapine / Condition Schizophrenia / other terms Random = 0
Intervention Lurasidone / Condition Schizophrenia / other terms Random = 4
Intervention Molindone / Condition Schizophrenia / other terms Random = 0
Intervention Olanzapine / Condition Schizophrenia / other terms Random = 7
Intervention Paliperidone / Condition Schizophrenia / other terms Random = 10
Intervention Quetiapine / Condition Schizophrenia / other terms Random = 3
Intervention Penfluridol / Condition Schizophrenia / other terms Random = 0
Intervention Perazine / Condition Schizophrenia / other terms Random = 0
Intervention Perphenazine / Condition Schizophrenia / other terms Random = 0
Intervention Pimozide / Condition Schizophrenia / other terms Random = 0
Intervention Risperidone / Condition Schizophrenia / other terms Random = 6
Intervention Sertindole / Condition Schizophrenia / other terms Random = 0
Intervention Sulpiride / Condition Schizophrenia / other terms Random = 0
Intervention Thioridazine / Condition Schizophrenia / other terms Random = 0
Intervention Thiothixene / Condition Schizophrenia / other terms Random = 0
Intervention Trifluoperazine / Condition Schizophrenia / other terms Random = 0
Intervention Ziprasidone / Condition Schizophrenia / other terms Random = 3
Intervention Zotepine / Condition Schizophrenia / other terms Random = 0
Intervention Zuclopenthixol / Condition Schizophrenia / other terms Random = 0
Intervention Amisulpride / Condition Schizophreniform / other terms Random = 0
Intervention Aripiprazole / Condition Schizophreniform / other terms Random = 0
Intervention Asenapine / Condition Schizophreniform / other terms Random = 0
Intervention Benperidol / Condition Schizophreniform / other terms Random = 0
Intervention Brexpiprazole / Condition Schizophreniform / other terms Random = 0
Intervention Cariprazine / Condition Schizophreniform / other terms Random = 0
Intervention Chlorpromazine / Condition Schizophreniform / other terms Random = 0
Intervention Clopenthixol / Condition Schizophreniform / other terms Random = 0
Intervention Clozapine / Condition Schizophreniform / other terms Random = 0
Intervention Flupenthixol / Condition Schizophreniform / other terms Random = 0
Intervention Fluphenazine / Condition Schizophreniform / other terms Random = 0
Intervention Fluspirilene / Condition Schizophreniform / other terms Random = 0
Intervention Haloperidol / Condition Schizophreniform / other terms Random = 0
Intervention Iloperidone / Condition Schizophreniform / other terms Random = 0
Intervention Levomepromazine / Condition Schizophreniform / other terms Random = 0
Intervention Loxapine / Condition Schizophreniform / other terms Random = 0
Intervention Lurasidone / Condition Schizophreniform / other terms Random = 0
Intervention Molindone / Condition Schizophreniform / other terms Random = 0
Intervention Olanzapine / Condition Schizophreniform / other terms Random = 1
Intervention Paliperidone / Condition Schizophreniform / other terms Random = 0
Intervention Quetiapine / Condition Schizophreniform / other terms Random = 0
Intervention Penfluridol / Condition Schizophreniform / other terms Random = 0
Intervention Perazine / Condition Schizophreniform / other terms Random = 0
Intervention Perphenazine / Condition Schizophreniform / other terms Random = 0
Intervention Pimozide / Condition Schizophreniform / other terms Random = 0
Intervention Risperidone / Condition Schizophreniform / other terms Random = 0
Intervention Sertindole / Condition Schizophreniform / other terms Random = 0
Intervention Sulpiride / Condition Schizophreniform / other terms Random = 0
Intervention Thioridazine / Condition Schizophreniform / other terms Random = 0
Intervention Thiothixene / Condition Schizophreniform / other terms Random = 0

Intervention Trifluoperazine / Condition Schizophreniform / other terms Random = 0
Intervention Ziprasidone / Condition Schizophreniform / other terms Random = 0
Intervention Zotepine / Condition Schizophreniform / other terms Random = 0
Intervention Zuclopenthixol / Condition Schizophreniform / other terms Random = 0
Intervention Amisulpride / Condition Schizoaffective / other terms Random = 0
Intervention Aripiprazole / Condition Schizoaffective / other terms Random = 0
Intervention Asenapine / Condition Schizoaffective / other terms Random = 0
Intervention Benperidol / Condition Schizoaffective / other terms Random = 0
Intervention Brexpiprazole / Condition Schizoaffective / other terms Random = 1
Intervention Cariprazine / Condition Schizoaffective / other terms Random = 0
Intervention Chlorpromazine / Condition Schizoaffective / other terms Random = 0
Intervention Clopenthixol / Condition Schizoaffective / other terms Random = 0
Intervention Clozapine / Condition Schizoaffective / other terms Random = 0
Intervention Flupenthixol / Condition Schizoaffective / other terms Random = 0
Intervention Fluphenazine / Condition Schizoaffective / other terms Random = 0
Intervention Fluspirilene / Condition Schizoaffective / other terms Random = 0
Intervention Haloperidol / Condition Schizoaffective / other terms Random = 0
Intervention Iloperidone / Condition Schizoaffective / other terms Random = 0
Intervention Levomepromazine / Condition Schizoaffective / other terms Random = 0
Intervention Loxapine / Condition Schizoaffective / other terms Random = 0
Intervention Lurasidone / Condition Schizoaffective / other terms Random = 0
Intervention Molindone / Condition Schizoaffective / other terms Random = 0
Intervention Olanzapine / Condition Schizoaffective / other terms Random = 0
Intervention Paliperidone / Condition Schizoaffective / other terms Random = 1
Intervention Quetiapine / Condition Schizoaffective / other terms Random = 0
Intervention Penfluridol / Condition Schizoaffective / other terms Random = 0
Intervention Perazine / Condition Schizoaffective / other terms Random = 0
Intervention Perphenazine / Condition Schizoaffective / other terms Random = 0
Intervention Pimozide / Condition Schizoaffective / other terms Random = 0
Intervention Risperidone / Condition Schizoaffective / other terms Random = 0
Intervention Sertindole / Condition Schizoaffective / other terms Random = 0
Intervention Sulpiride / Condition Schizoaffective / other terms Random = 0
Intervention Thioridazine / Condition Schizoaffective / other terms Random = 0
Intervention Thiothixene / Condition Schizoaffective / other terms Random = 0
Intervention Trifluoperazine / Condition Schizoaffective / other terms Random = 0
Intervention Ziprasidone / Condition Schizoaffective / other terms Random = 0
Intervention Zotepine / Condition Schizoaffective / other terms Random = 0
Intervention Zuclopenthixol / Condition Schizoaffective / other terms Random = 0
Intervention Amisulpride / Condition Psychosis / other terms Random = 1
Intervention Aripiprazole / Condition Psychosis / other terms Random = 9
Intervention Asenapine / Condition Psychosis / other terms Random = 1
Intervention Benperidol / Condition Psychosis / other terms Random = 0
Intervention Brexpiprazole / Condition Psychosis / other terms Random = 8
Intervention Cariprazine / Condition Psychosis / other terms Random = 3
Intervention Chlorpromazine / Condition Psychosis / other terms Random = 0
Intervention Clopenthixol / Condition Psychosis / other terms Random = 0
Intervention Clozapine / Condition Psychosis / other terms Random = 3
Intervention Flupenthixol / Condition Psychosis / other terms Random = 0
Intervention Fluphenazine / Condition Psychosis / other terms Random = 0
Intervention Fluspirilene / Condition Psychosis / other terms Random = 0
Intervention Haloperidol / Condition Psychosis / other terms Random = 3
Intervention Iloperidone / Condition Psychosis / other terms Random = 1
Intervention Levomepromazine / Condition Psychosis / other terms Random = 0

Intervention Loxapine / Condition Psychosis / other terms Random = 0
Intervention Lurasidone / Condition Psychosis / other terms Random = 8
Intervention Molindone / Condition Psychosis / other terms Random = 0
Intervention Olanzapine / Condition Psychosis / other terms Random = 6
Intervention Paliperidone / Condition Psychosis / other terms Random = 10
Intervention Quetiapine / Condition Psychosis / other terms Random = 6
Intervention Penfluridol / Condition Psychosis / other terms Random = 0
Intervention Perazine / Condition Psychosis / other terms Random = 0
Intervention Perphenazine / Condition Psychosis / other terms Random = 1
Intervention Pimozide / Condition Psychosis / other terms Random = 0
Intervention Risperidone / Condition Psychosis / other terms Random = 6
Intervention Sertindole / Condition Psychosis / other terms Random = 0
Intervention Sulpiride / Condition Psychosis / other terms Random = 0
Intervention Thioridazine / Condition Psychosis / other terms Random = 0
Intervention Thiothixene / Condition Psychosis / other terms Random = 0
Intervention Trifluoperazine / Condition Psychosis / other terms Random = 0
Intervention Ziprasidone / Condition Psychosis / other terms Random = 3
Intervention Zotepine / Condition Psychosis / other terms Random = 0
Intervention Zuclopenthixol / Condition Psychosis / other terms Random = 0
Total = 119

WHO ICTRP 10-01-19

Amisulpride and schizo* and random* = 1
Aripiprazole and schizo* and random* = 5
Asenapine and schizo* and random* = 1
Benperidol and schizo* and random* = 0
Brexpiprazole and schizo* and random* = 2
Cariprazine and schizo* and random* = 2
Chlorpromazine and schizo* and random* = 1
Clopenthixol and schizo* and random* = 0
Clozapine and schizo* and random* = 1
Flupenthixol and schizo* and random* = 0
Fluphenazine and schizo* and random* = 0
Fluspirilene and schizo* and random* = 0
Haloperidol and schizo* and random* = 1
Iloperidone and schizo* and random* = 0
Levomepromazine and schizo* and random* = 0
Loxapine and schizo* and random* = 0
Lurasidone and schizo* and random* = 6
Molindone and schizo* and random* = 0
Olanzapine and schizo* and random* = 6
Paliperidone and schizo* and random* = 10
Quetiapine and schizo* and random* = 4
Penfluridol and schizo* and random* = 0
Perazine and schizo* and random* = 0
Perphenazine and schizo* and random* = 0
Pimozide and schizo* and random* = 0
Risperidone and schizo* and random* = 8
Sertindole and schizo* and random* = 0
Sulpiride and schizo* and random* = 0
Thioridazine and schizo* and random* = 0
Thiothixene and schizo* and random* = 0

Trifluoperazine and schizo* and random* = 7
Ziprasidone and schizo* and random* = 1
Zotepine and schizo* and random* = 0
Zuclopenthixol and schizo* and random* = 0
Amisulpride and psycho* and random* = 1
Aripiprazole and psycho* and random* = 2
Asenapine and psycho* and random* = 0
Benperidol and psycho* and random* = 0
Brexpiprazole and psycho* and random*= 0
Cariprazine and psycho* and random* = 0
Chlorpromazine and psycho* and random* = 2
Clopenthixol and psycho* and random* = 0
Clozapine and psycho* and random* = 0
Flupenthixol and psycho* and random* = 0
Fluphenazine and psycho* and random* = 0
Fluspirilene and psycho* and random* = 0
Haloperidol and psycho* and random* = 2
Iloperidone and psycho* and random* = 0
Levomepromazine and psycho* and random* = 0
Loxapine and psycho* and random* = 0
Lurasidone and psycho* and random* = 2
Molindone and psycho* and random* = 0
Olanzapine and psycho* and random* = 3
Paliperidone and psycho* and random* = 1
Quetiapine and psycho* and random* = 2
Penfluridol and psycho* and random* = 0
Perazine and psycho* and random* = 0
Perphenazine and psycho* and random* = 0
Pimozide and psycho* and random* = 0
Risperidone and psycho* and random* = 1
Sertindole and psycho* and random* = 0
Sulpiride and psycho* and random* = 0
Thioridazine and psycho* and random* = 0
Thiothixene and psycho* and random* = 0
Trifluoperazine and psycho* and random* = 16
Ziprasidone and psycho* and random* = 0
Zotepine and psycho* and random* = 0
Zuclopenthixol and psycho* and random* = 0
Total = 88

Appendix 4: Statistical methods in detail

Measures of treatment effect

The effect size for dichotomous outcomes was the odds ratio (OR) and its 95% confidence intervals (CIs). A reviewer requested risk ratios (RR) as effect size for dichotomous outcomes, as these are easier to understand for clinicians. We followed the reviewer´s suggestion and changed the effect size for dichotomous otucomes to RRs. Everyone allocated to the intervention was counted whether they completed the follow up or not. If the authors applied such a strategy, we used their results. If the original authors presented only the results of the per-protocol or completer population, we assumed that those participants lost to follow-up would not have changed in a given outcome. In terms of efficacy this means that they were conservatively considered to have not responded to treatment. In terms of tolerability it would meant that participants would not have developed a side-effect which we think is appropriate, because otherwise side-effects, many of which are rare, would have been overestimated.

The effect size measure for continuous outcomes was the standardized mean difference (SMD) because we expected that the studies use different rating scales of overall schizophrenia symptomatology, especially the Positive and Negative Syndrome Scale (PANSS) or the Brief Psychiatric Rating Scale (BPRS). Missing standard deviations: When standard errors instead of standard deviations (SD) were presented, the former was converted to standard deviations (SDs). If both were missing we estimated SDs from confidence intervals, t-values, or p-values as described in Section 7.7.3 of the Cochrane Handbook for Systematic Reviews.[1] When no information could be obtained we derived SDs from those of the other studies using a validated imputation technique.[2]

Statistical details for network meta-analysis and meta-regression models

We used standard network meta-analysis and meta-regression models fitted in JAGS using the rjags package in R.[3] We accounted for the correlations induced by multi-arm studies by employing multivariate distributions. We assumed a single heterogeneity parameter for each network. We presented the summary RRs or SMD for all pairwise comparisons in a league table. We also estimated the prediction intervals to assess how much the common heterogeneity affects the relative effect with respect to the extra uncertainty

anticipated in a future study. To rank the various treatments for each outcome, we used the surface under the cumulative ranking curve (SUCRA) and the mean ranks. If data was too sparse for a network meta-analytic approach, we did a simple pairwise meta-analysis using random effects model.

The heterogeneity (variability in relative treatment effects within the same treatment comparison) was measured with the tau-squared (the variance of the random effects distribution). The heterogeneity variance was assumed common across the various treatment comparisons and the empirical distributions were to characterise the amount of heterogeneity as low, moderate or high using the first and third quantiles[3]. Potential reasons for heterogeneity were explored by subgroup analysis and meta-regressions.

For every study $i = 1, \dots, N$ we denote with $y_{i,k}$ the symptoms score (either final or change from baseline) in arm $k = 1, \dots, K$, $sd_{i,k}$ the standard deviation and $n_{i,k}$ is the sample size in arm k. Then the normal likelihood is employed to $y_{i,k} \sim N(\varphi_{i,k}, (sd_{i,k})^2 / n_{i,k})$ in each arm and then we parametrize to the study-specific standardised mean difference $\theta^*_{i,k}$

$$\text{logit}(\varphi_{i,1}) = u_i / S_i$$

$$\text{logit}(\varphi_{i,k}) = (u_i + \theta^*_{i,k}) / S_i \quad \text{for } k \geq 2,$$

where S_i is the pooled standard deviation in the study arms.

Network meta-analysis:

For the model without covariates we set $\theta^*_{i,k} = \theta_{i,k}$ and

$\theta_{i,2}, \dots, \theta_{i,K} \sim MVN(\mu_2, \dots, \mu_K, \boldsymbol{T})$

The matrix T involves the heterogeneity standard deviation τ.

Each $\mu_2, \dots, \mu_K$ corresponds into a treatment comparison, e.g. $\mu_k \equiv \mu_{AB}$; then we assume consistency by putting $\mu_{AB} = d_A - d_B$, with $d_A = 0$ for an arbitrarily selected reference treatment.

Network meta-regression:

In the network meta-regression models we set

$\theta^*_{i,k} = \theta_{i,k} + \beta_{1,\kappa} \times (x_i - ref_x)$

In the model with independent and consistent coefficients we define $\beta_{1,\kappa} = \beta_\kappa$

if treatment 1 is placebo; otherwise $\beta_{1,\kappa} \equiv \beta_{AB} = \beta_B - \beta_A$ for any treatments A, B.

In the model with exchangeable coefficients we set $\beta_\kappa \sim N(B, \tau_B^2)$.

Network meta-regression for placebo response:

In placebo-controlled studies the covariate x_i is defined as the improvement in symptoms on the PANSS scale in the placebo arm. This variable is unobserved in head-to-head studies. Previous research has shown a strong association between year of study ($Year_i$) and placebo response,[4] hence the x_i values were stochastically imputed for head-to-head studies using the following process, nested within the meta-regression model.

$x_i \sim N(X_i, \sigma_x^2)$

$X_i = a + b \times Year_i$

Note: in all meta-regression analyses we excluded drugs that have been studied in less than 100 participants to ensure convergence of the models.

Priors:

We assume vague normal priors $N(0,10^4)$ for the paraemters u_i and $d_B, d_{C,} d_D$, ...

and β_k (if assumed independent) or B (if β_k are assumed exchangeable), a and b.

For variance parameters we employed: $\tau \sim U(0,5)$, $\tau_B \sim U(0,1)$, $1/\sigma_x^2 \sim gamma(0.001,0.001)$

All models were run using 100 000 iterations after an initial burn-in of 10 000; a thinning of 10 was used. Convergence was evaluated by monitoring the mixing of several chains with different initial values.

Assessment of the transitivity assumption

We assumed that patients who fulfilled the inclusion criteria outlined above are equally likely to be randomised to any of the antipsychotic that we planned to compare. Nevertheless we inspected the distribution of potential effect modifiers with boxplots (appendix 5). We investigateed the distribution of clinical and methodological variables that can act as effect modifiers across treatment comparisons. The main features, which have been demonstrated to date to moderate efficacy of antipsychotics, at least compared to placebo, were the degree of placebo response (which has increased over the years) and industry sponsorship. Less robust factors included severity of illness at baseline, gender, chronicity and publication year. We investigated if these variables were similarly distributed across studies grouped by comparison, whereas it was clear a priori that publication year, a composite of various factors, will differ between older and more recent antipsychotics. We considered that placebo response in schizophrenia has increased over the years[4] and that there could be differences between placebo-controlled trials and head-to-head trials as it is known from antidepressant trials in major depressive disorder.

Assessment of inconsistency

The strategical and conceptual evaluation of transitivity was supplemented with a statistical evaluation of consistency, the agreement between direct and indirect evidence. We employed local as well as global methods to evaluate consistency. Local methods detected ‘hot spots’ of inconsistency, evidence loops that were inconsistent or comparisons for which direct and indirect evidence disagreed. We employed the loop-specific approach to evaluate inconsistency within each loop of evidence, and a method that separated direct evidence from indirect evidence provided by the entire network.

Appendix 5: Assessment of transitivity

Before conducting the statistical analysis we assessed whether the trials included in the NMA were on average similar in terms of characteristics that might modify the treatment effect (so that the transitivity assumption is plausible). Indirect comparisons, in contrast to direct comparisons, are not protected by randomisation and may be confounded by differences between the trials. In our analysis we deemed the following parameters as possible confounders: placebo response, publication year, mean age, baseline severity, percentage of male participants and number randomized based on results from a prior analysis.[4]

The plausibility of the transitivity assumption was evaluated by comparing the distribution of these potential effect modifiers across studies grouped by comparison. The impact of the individual parameters was assessed by several metaregressions (appendix 9) and sensitivity analyses (appendix 10).

5.1 Placebo response

To examine the distribution of placebo response over the individual antipsychotics, we examined the change in the "Positive and Negative Symptom Scale of Schizophrenia" (PANSS) points from baseline to endpoint of the placebo arms. As expected the range of placebo response was large between -28 points and 17 points on the PANSS scale. In older studies patients responded only a little in the placebo arm, if at all (e.g. chlorpromazine, median five points PANSS worse). In contrast participants in placebo arms of newer compounds responded considerably (e.g. brexpiprazole, median 14 points PANSS better). The median response in placebo arms was six points on the PANSS scale. The impact of the placebo response was therefore examined in several metaregressions (appendix 9.1) and sensitivity analyses (appendix 10).

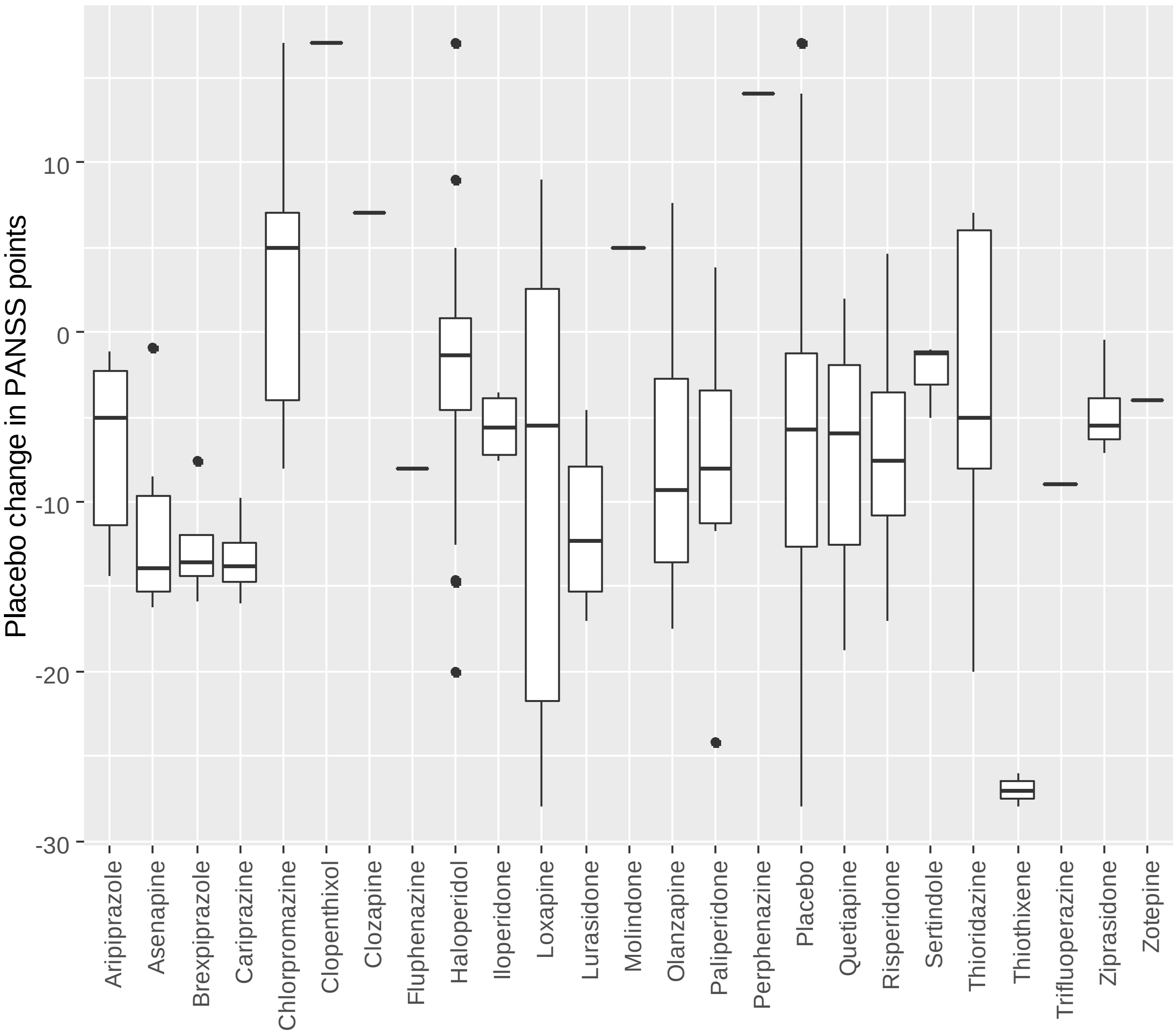

Figure 5.1: Boxplot for distribution of placebo response across comparisons

The boxplot presents the change in the “Positive and Negative Symptom Scale of Schizophrenia” (PANSS) points from baseline to endpoint of the placebo comparators of the individual antipsychotics. If original data was reported on the “Brief Psychiatry Rating Scale”, data was transformed using validated formulas.[5]

5.2 Publication year

We examined the distribution of publication year over the individual antipsychotics. The overall range was between 1967 and 2018 with a median of 2000. Obviously, the individual compounds differed considerably with regards to publication year, because some substances were only approved recently. We examined publication year as a potential effect modifier, because it can be a proxy parameter for a number of factors that may have changed over the years (e.g. changes in trial design, monitoring, trial populations etc), that could have possibly influenced the effect sizes.

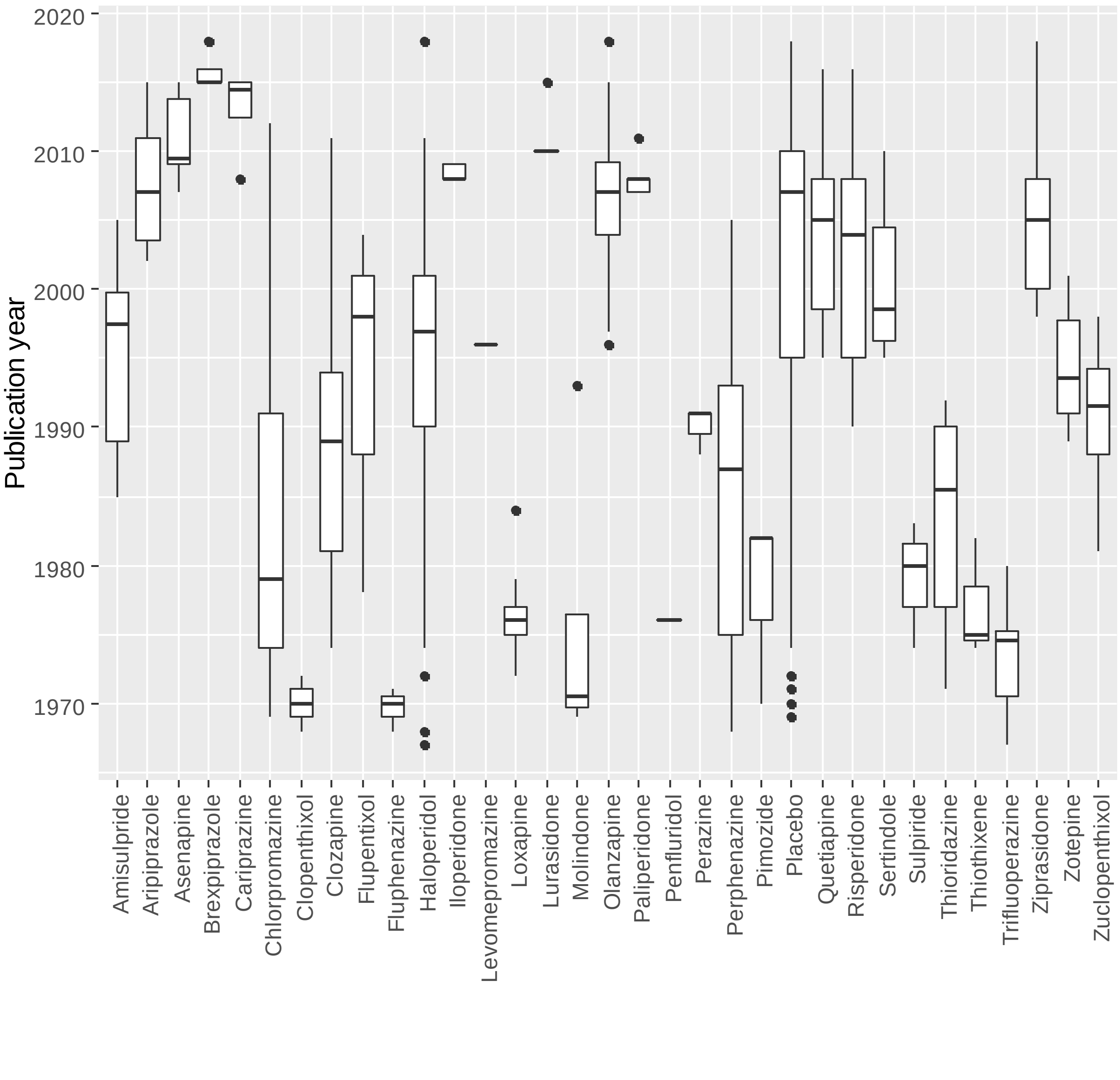

Figure 5.2: Boxplot for distribution of publication year across comparisons

5.3 Sample size

We examined the distribution of sample size over the individual antipsychotics. The overall sample size range was 4 to 1336 with a median of 50. Overall there were smaller sample sizes in older antipsychotics. Sample size is accounted for by more weight given to larger studies in meta-analysis. In addition, we inspected the effects of sample size as a potential effect modifier.

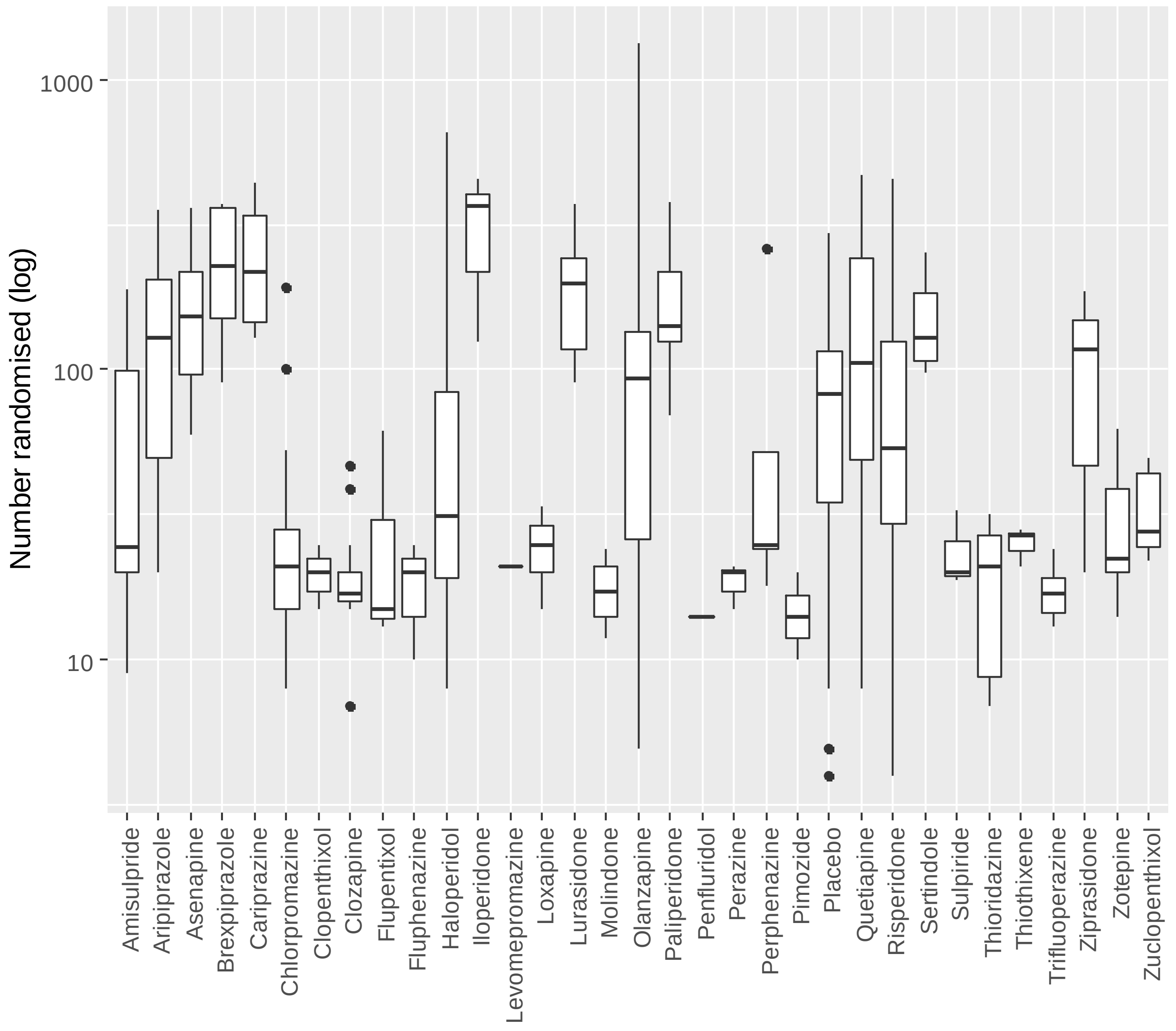

Figure 5.3: Boxplot for distribution of sample sizes across comparisons

Due to the large range sample sizes are presented on a logarithmic scale.

5.4 Baseline severity

We examined the distribution of baseline severity on the positive and negative symptom scale of schizophrenia (PANSS) over the individual antipsychotics. The median PANSS points at baseline were 95. Most antipsychotics, with a few exceptions, had a baseline severity between 90 and 110 PANSS points. The impact of baseline severity was examined in a metaregression (appendix 9.5).

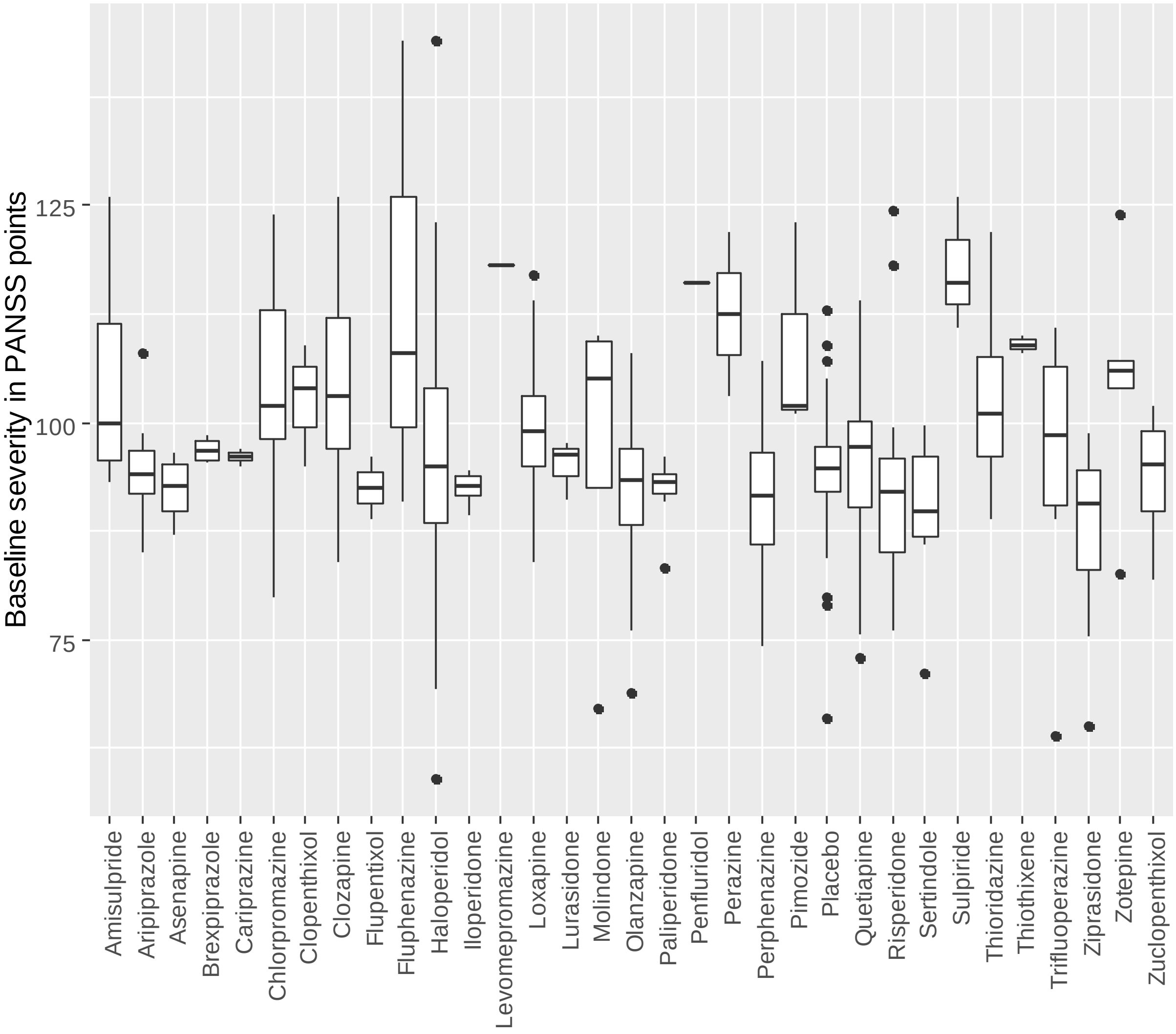

Figure 5.4: Boxplot for distribution of baseline severity across comparisons

Baseline severity values are presented using the “Positive and Negative Symptom rating scale of schizophrenia”=PANSS. If original data was reported on the “Brief Psychiatry Rating Scale”, data was transformed using validated formulas.[5]

5.5 Mean age

We examined the distribution of mean age over the individual antipsychotics. The overall median mean age was 38 with most antipsychotics between the age of 30 and 40.

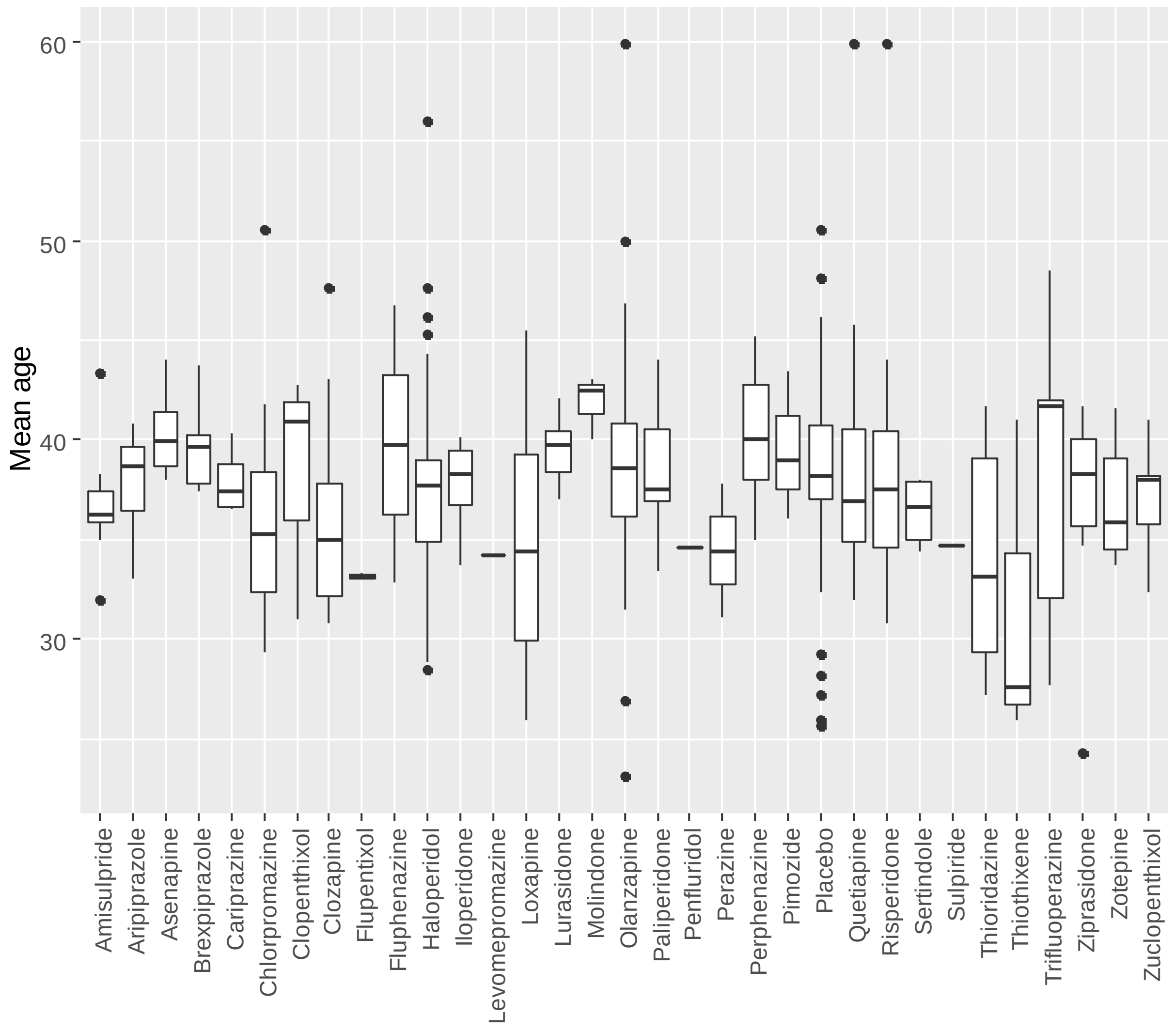

Figure 5.5: Boxplot for distribution of mean age across comparisons

5.6 Percentage male

We examined the distribution of percentage male over the individual antipsychotics. Most antipsychotics had more than 50 % male participants with a median 66%. Some studies had only male or only female participants.

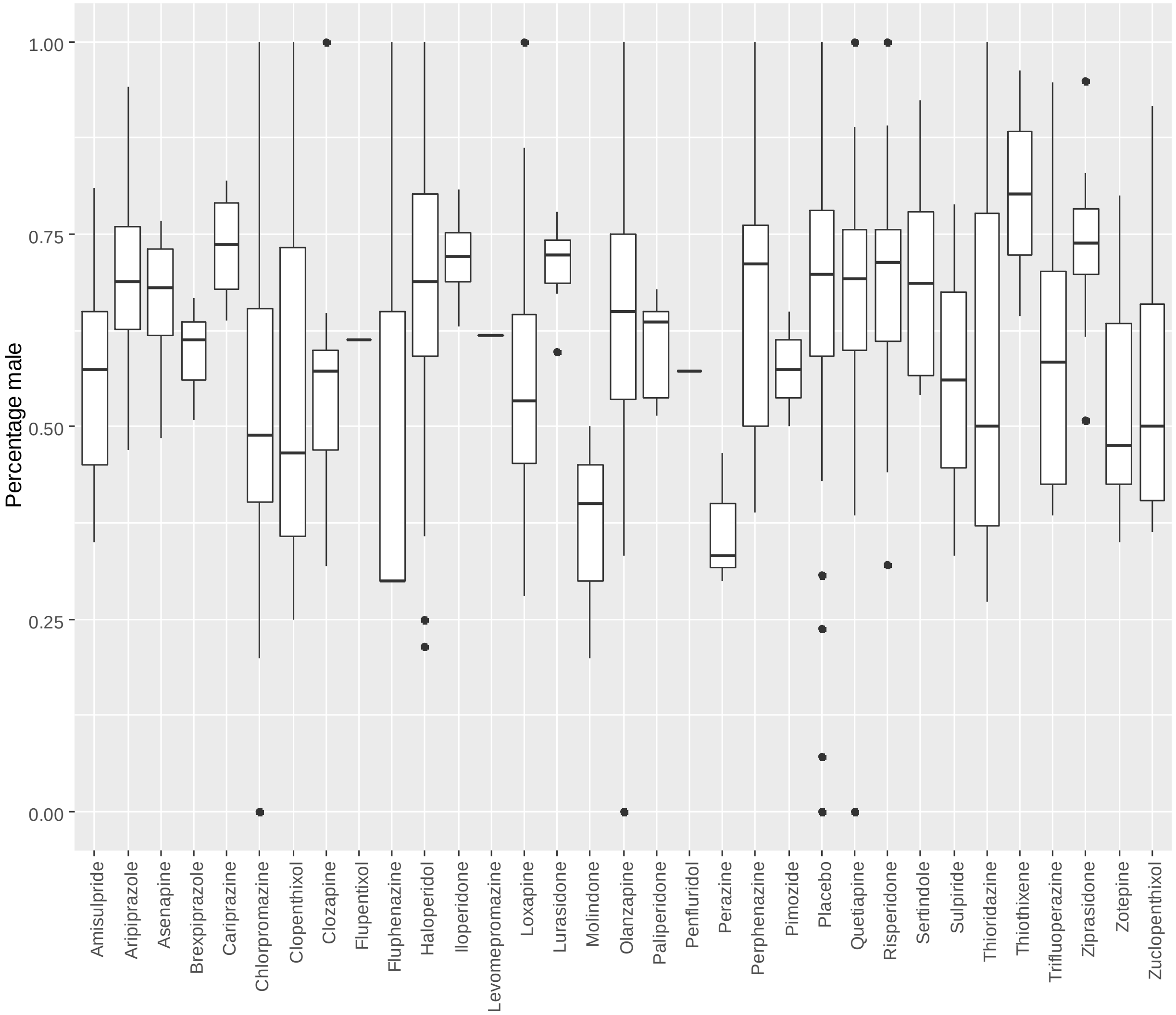

Figure 5.6: Boxplot for distribution of percentage male across comparisons

Appendix 6: PRISMA Flow Diagram

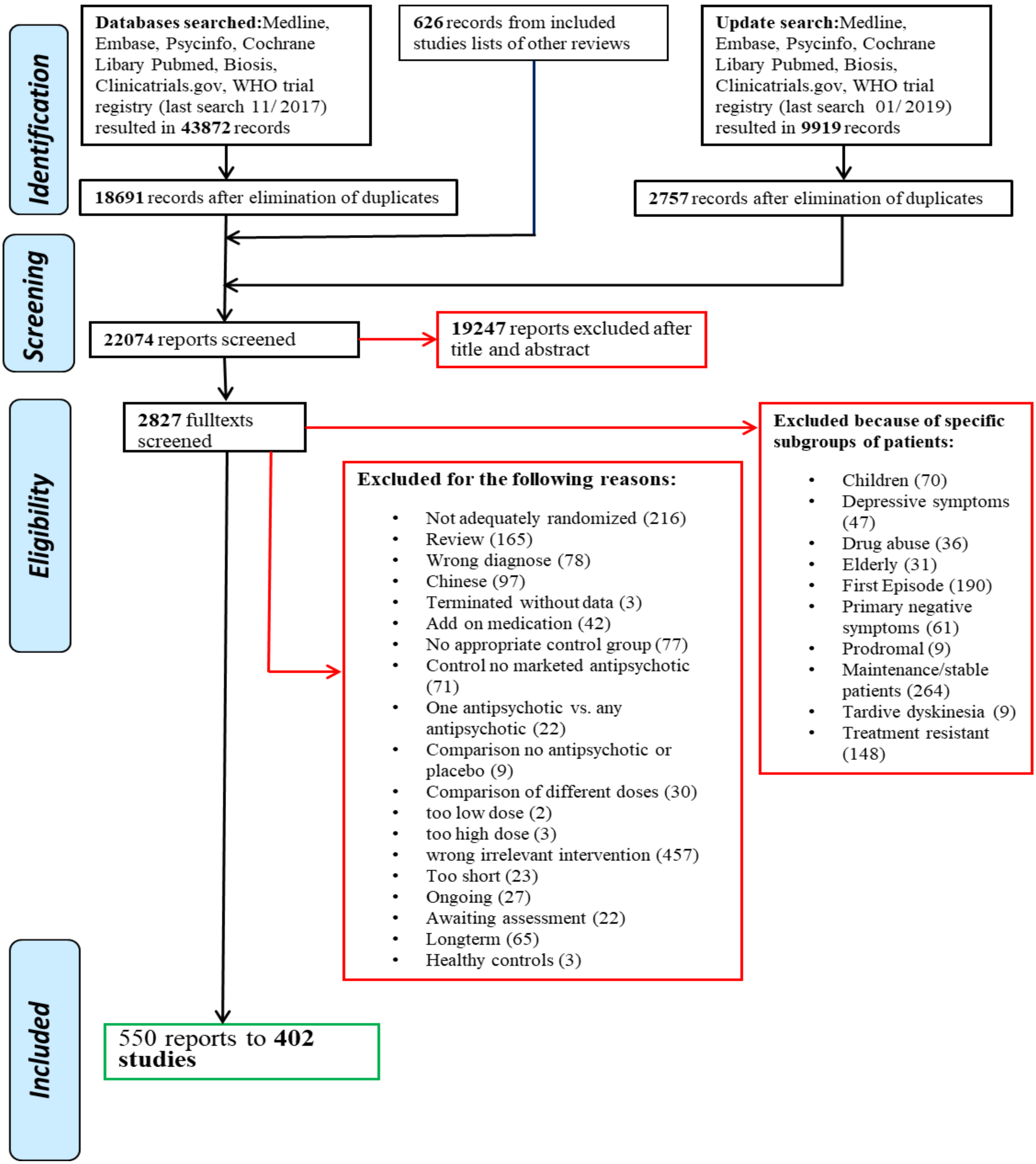

From: Moher D, Liberati A, Tetzlaff J, Altman DG, The PRISMA Group (2009). *P*referred *R*eporting *I*tems for *S*ystematic Reviews and *M*eta-*A*nalyses: The PRISMA Statement. PLoS Med 6(7): e1000097. doi:10.1371/journal.pmed1000097

References

1. Wang J, Sampson S. Sulpiride versus placebo for schizophrenia. The Cochrane database of systematic reviews 2014; (4):CD007811.
2. Wahlbeck K, Cheine M, Essali MA. Clozapine versus typical neuroleptic medication for schizophrenia. The Cochrane database of systematic reviews 2000; (2):CD000059.
3. Soares BG, Fenton M, Chue P. Sulpiride for schizophrenia. The Cochrane database of systematic reviews 2000; (2):CD001162.
4. Shen X, Xia J, Adams CE. Flupenthixol versus placebo for schizophrenia. COCHRANE DATABASE OF SYSTEMATIC REVIEWS 2012; (11).
5. Mothi M, Sampson S. Pimozide for schizophrenia or related psychoses. The Cochrane database of systematic reviews 2013; (11):CD001949.
6. Matar HE, Almerie MQ, Sampson S. Fluphenazine (oral) versus placebo for schizophrenia (Review). COCHRANE DATABASE OF SYSTEMATIC REVIEWS 2013; (7).
7. Marques LO, Lima MS, Soares BGO. Trifluoperazine for schizophrenia. The Cochrane database of systematic reviews 2004; (1):CD003545.
8. Leucht S, Helfer B, Hartung B. Perazine for schizophrenia. The Cochrane database of systematic reviews 2014; (1):CD002832.
9. Leucht S, Hartung B. Benperidol for schizophrenia. The Cochrane database of systematic reviews 2005; (2):CD003083.
10. Lacey M, Jayaram MB. Zuclopenthixol versus placebo for schizophrenia. The Cochrane database of systematic reviews 2015; (12):CD010598.
11. Hartung B, Sampson S, Leucht S. Perphenazine for schizophrenia. The Cochrane database of systematic reviews 2015; (3):CD003443.
12. Fenton M, Rathbone J, Reilly J, Sultana A. Thioridazine for schizophrenia. The Cochrane database of systematic reviews 2007; (3):CD001944.
13. Chakrabarti A, Bagnall A, Chue P, Fenton M, Palaniswamy V, Wong W et al. Loxapine for schizophrenia. The Cochrane database of systematic reviews 2007; (4):CD001943.
14. Asenjo Lobos C, Komossa K, Rummel-Kluge C, Hunger H, Schmid F, Schwarz S et al. Clozapine versus other atypical antipsychotics for schizophrenia. COCHRANE DATABASE OF SYSTEMATIC REVIEWS 2010; (11).
15. Adams CE, Bergman H, Irving CB, Lawrie S. Haloperidol versus placebo for schizophrenia. The Cochrane database of systematic reviews 2013; (11):CD003082.
16. Adams CE, Awad GA, Rathbone J, Thornley B, Soares-Weiser K. Chlorpromazine versus placebo for schizophrenia. COCHRANE DATABASE OF SYSTEMATIC REVIEWS 2014; (1).
17. Zhu, Yikang; Krause, Marc; Huhn, Maximilian; Rothe, Philipp; Schneider-Thoma, Johannes; Chaimani, Anna et al. (2017): Antipsychotic drugs for the acute treatment of patients with a first episode of schizophrenia: a systematic review with pairwise and network meta-analyses. In: The lancet. Psychiatry 4 (9), S. 694–705. DOI: 10.1016/S2215-0366(17)30270-5.
18. Samara, Myrto T.; Dold, Markus; Gianatsi, Myrsini; Nikolakopoulou, Adriani; Helfer, Bartosz; Salanti, Georgia; Leucht, Stefan (2016): Efficacy, Acceptability, and Tolerability of Antipsychotics in Treatment-Resistant Schizophrenia: A Network Meta-analysis. In: JAMA psychiatry 73 (3), S. 199–210. DOI: 10.1001/jamapsychiatry.2015.2955.

Appendix 7: List of included studies

Study name	Antipsychotics and daily dose in mg, on flexible dosage mean value (range)	N	Duration in weeks	Mean duration of illness in years	Diagnosis	Blinding
A1281046[1]	Risperidone 5 (2-8)	29	12	n.i.	schizophrenia, schizoaffective and schizophreniform disorder (DSM-III-R)	2
	Ziprasidone 120 (80-160)	29	12	n.i.	schizophrenia, schizoaffective and schizophreniform disorder (DSM-III-R)	2
A1281050[2]	Haloperidol 12.4 (5-20)	122	6	n.i.	schizophrenia or schizoaffective disorder (DSM-IV)	1
	Ziprasidone 108.1 (40-154)	130	6	n.i.	schizophrenia or schizoaffective disorder (DSM-IV)	1
Abdolahian 2008[3]	Risperidone 6 (4-8)	35	8	n.i.	chronic schizophrenia (DSM-IV)	2
	Haloperidol 12.5 (10-15)	30	8	n.i.	chronic schizophrenia (DSM-IV)	2
Addington 2004[4]	Risperidone 7.4 (6-10)	147	8	9.29	acute exacerbation of schizophrenia or schizoaffective disorder (DSM-III-R)	2
	Ziprasidone 114.2 (80-160)	149	8	9.92	acute exacerbation of schizophrenia or schizoaffective disorder (DSM-III-R)	2
Ahmed 2007[5]	Placebo	150	6	n.i.	schizophrenia acute exacerbation (DSM-IV-TR)	2
	Olanzapine 15 (15-15)	150	6	n.i.	schizophrenia acute exacerbation (DSM-IV-TR)	2

Study name	Antipsychotics and daily dose in mg, on flexible dosage mean value (range)	N	Duration in weeks	Mean duration of illness in years	Diagnosis	Blinding
Ahn 2007[6]	Risperidone 4.9 (2-8)	56	12	3.8	schizophrenia or schizoaffective disorder (DSM-IV)	1
	Ziprasidone 132.2 (80-160)	56	12	11.9	schizophrenia or schizoaffective disorder (DSM-IV)	1
Allan 1998[7]	Haloperidol 10 (4-16)	10	6	n.i.	schizophrenia (clinical diagnosis)	2
	Olanzapine 20 (5-20)	13	6	n.i.	schizophrenia (clinical diagnosis)	2
Amakusa 1973[8]	Sulpiride 700 (400-1000)	32	6	n.i.	schizophrenia (clinical Diagnosis)	2
	Perphenazine 21 (12-30)	32	6	n.i.	schizophrenia (clinical Diagnosis)	2
Angst 1971[9]	Clozapine 136.53 (n.i.-n.i.)	32	4.29	n.i.	schizophrenia (ICD-9)	2
	Levomepromazin 180.26 (n.i.-n.i.)	32	4.29	n.i.	schizophrenia (ICD-9)	2
Anumonye 1976[10]	Chlorpromazine 450 (300-600)	12	4	n.i.	schizophrenia (DSM-III-R)	1
	Pimozide 2.5 (1-4)	12	4	n.i.	schizophrenia (DSM-III-R)	1
Arvanitis 1997[11]	Placebo	51	6	14	acute exacerbation of chronic or subchronic schizophrenia (DSM-III-R)	2
	Quetiapine 677 (600-750)	105	6	13.97	acute exacerbation of chronic or subchronic schizophrenia (DSM-III-R)	2
	Haloperidol 12 (12-12)	52	6	16	acute exacerbation of chronic or subchronic schizophrenia (DSM-III-R)	2

Study name	Antipsychotics and daily dose in mg, on flexible dosage mean value (range)	N	Duration in weeks	Mean duration of illness in years	Diagnosis	Blinding
Atmaca 2002[12]	Haloperidol 10 (10-10)	17	6	n.i.	schizophrenia (DSM-IV)	0
	Quetiapine 600 (600-600)	18	6	n.i.	schizophrenia (DSM-IV)	0
Augustin 1996[13]	Haloperidol n.i. (n.i.-n.i.)	18	6	n.i.	schizophrenia (clinical diagnosis)	0
	Placebo	18	6	n.i.	schizophrenia (clinical diagnosis)	0
Avasthi 2001[14]	Olanzapine 12.5 (5-20)	17	12	n.i.	schizophrenia (DSM-IV)	0
	Haloperidol 12.5 (5-20)	10	12	n.i.	schizophrenia (DSM-IV)	0
Azorin 2006[15]	Risperidone 6.6 (4-10)	89	12	n.i.	schizophrenia of the paranoid, disorganized, catatonic, or undifferentiated type (DSM-IV)	2
	Sertindole 16.2 (12-24)	98	12	n.i.	schizophrenia of the paranoid, disorganized, catatonic, or undifferentiated type (DSM-IV)	2
Baker 1959[16]	Chlorpromazine 270 (300-300)	7	5	n.i.	schizophrenia (clinical diagnosis)	2
	Placebo	8	5	n.i.	schizophrenia (clinical diagnosis)	2
Balasubramanian 1991[17]	Chlorpromazine 600 (100-600)	44	10	n.i.	acute functional psychosis (clinical Diagnosis)	2
	Zuclopenthixol 75 (25-150)	50	10	n.i.	acute functional psychosis (clinical Diagnosis)	2
Ban 1975e[18]	Chlorpromazine 500 (200-800)	10	12	n.i.	chronic and acute schizophrenia (clinical diagnosis)	2

Study name	Antipsychotics and daily dose in mg, on flexible dosage mean value (range)	N	Duration in weeks	Mean duration of illness in years	Diagnosis	Blinding
	Thiothixene 25 (10-40)	10	12	n.i.	chronic and acute schizophrenia (clinical diagnosis)	2
	Placebo	10	12	n.i.	chronic and acute schizophrenia (clinical diagnosis)	2
Barbato 2007c[19]	Olanzapine 15 (15-15)	150	6	n.i.	acutely exacerbated schizophrenia (DSM-IV)	2
	Placebo	145	6	n.i.	acutely exacerbated schizophrenia (DSM-IV)	2
Barbato 2007d[19]	Haloperidol 10 (10-10)	52	6	n.i.	acute exacerbated schizophrenia (DSM-IV)	2
	Placebo	51	6	n.i.	acute exacerbated schizophrenia (DSM-IV)	2
Beasley 1996a[20]	Placebo	50	6	14	schizophrenia (DSM-III-R)	2
	Olanzapine 10 (10-10)	50	6	18	schizophrenia (DSM-III-R)	2
Beasley 1996b[21]	Haloperidol 16.4 (10-20)	69	6	15	schizophrenia with an acute exacerbation (DSM-III-R)	2
	Olanzapine 14.04 (7.5-17.5)	133	6	13.97	schizophrenia with an acute exacerbation (DSM-III-R)	2
	Placebo	68	6	13	schizophrenia with an acute exacerbation (DSM-III-R)	2
Beasley 1997[22]	Haloperidol 17.6 (10-20)	81	6	12.6	schizophrenia with acute exacerbation (DSM-III-R)	2

Study name	Antipsychotics and daily dose in mg, on flexible dosage mean value (range)	N	Duration in weeks	Mean duration of illness in years	Diagnosis	Blinding
	Olanzapine 13.88 (7.5-17.5)	175	6	12.1	schizophrenia with acute exacerbation (DSM-III-R)	2
Bechelli 1983[23]	Haloperidol 11.5 (10-20)	30	3	n.i.	acute schizophrenia (ICD-9)	2
	Placebo	31	3	n.i.	acute schizophrenia (ICD-9)	2
Bergling 1975[24]	Thioridazine n.i. (n.i.-n.i.)	24	8	n.i.	schizophrenic or paranoid syndromes (clinical Diagnosis)	2
	Thiothixene n.i. (n.i.-n.i.)	18	8	n.i.	schizophrenic or paranoid syndromes (clinical Diagnosis)	2
Bernardo 2001[25]	Haloperidol 10 (10-10)	13	4	n.i.	schizophrenia or schizophreniform disorder, acute psychosis (DSM-IV)	2
	Olanzapine 10 (10-10)	14	4	n.i.	schizophrenia or schizophreniform disorder, acute psychosis (DSM-IV)	2
Bhowmick 2010[26]	Olanzapine 15 (10-20)	40	12	2.2	schizophrenia (DSM-IV)	1
	Amisulpride 450 (100-800)	40	12	2.1	schizophrenia (DSM-IV)	1
Bishop 1963c[27]	Chlorpromazine 400 (0-800)	10	10	n.i.	chronic schizophrenia (clinical Diagnosis)	2
	Placebo	10	10	n.i.	chronic schizophrenia (clinical Diagnosis)	2
Bishop 1964[28]	Placebo	14	10	11.7	chronic schizophrenia (clinical diagnosis)	2
	Trifluoperazine 20 (0-40)	14	10	12.4	chronic schizophrenia (clinical diagnosis)	2

Study name	Antipsychotics and daily dose in mg, on flexible dosage mean value (range)	N	Duration in weeks	Mean duration of illness in years	Diagnosis	Blinding
Bishop 1970[29]	Loxapine 120 (120-120)	12	10	17.7	chronic schizophrenic (clinical Diagnosis)	2
	Trifluoperazine 60 (60-60)	12	10	16.6	chronic schizophrenic (clinical Diagnosis)	2
Blin 1996[30]	Risperidone 7.4 (4-12)	21	4	n.i.	acute exacerbation of schizophrenia (DSM-III-R)	2
	Haloperidol 7.6 (4-12)	20	4	n.i.	acute exacerbation of schizophrenia (DSM-III-R)	2
	Levomepromazin 100 (50-150)	21	4	n.i.	acute exacerbation of schizophrenia (DSM-III-R)	2
Boehle 1995[31]	Clozapine n.i. (n.i.-n.i.)	n.i.	8	n.i.	schizophrenic inpatients (clinical Diagnosis)	2
	Fluphenazine n.i. (n.i.-n.i.)	n.i.	8	n.i.	schizophrenic inpatients (clinical Diagnosis)	2
Borison 1989[32]	Thioridazine 450 (150-750)	8	6	n.i.	schizophrenia (DSM-III)	2
	Haloperidol 45 (15-75)	8	6	n.i.	schizophrenia (DSM-III)	2
	Placebo	8	6	n.i.	schizophrenia (DSM-III)	2
Borison 1991a[33]	Chlorpromazine 800 (400-1600)	9	4	n.i.	schizophrenia acute exacerbation (DSM-III)	2
	Placebo	10	4	n.i.	schizophrenia acute exacerbation (DSM-III)	2
Borison 1992[34]	Placebo	54	6	16.5	acute exacerbation schizophrenia (DSM-III-R)	2

Study name	Antipsychotics and daily dose in mg, on flexible dosage mean value (range)	N	Duration in weeks	Mean duration of illness in years	Diagnosis	Blinding
	Risperidone 7.8 (2-10)	53	6	15.9	acute exacerbation schizophrenia (DSM-III-R)	2
	Haloperidol 15 (4-20)	53	6	12.8	acute exacerbation schizophrenia (DSM-III-R)	2
Borison 1996[35]	Placebo	55	6	15	chronic or subchronic schizophrenia with acute exacerbation (DSM-III-R)	2
	Quetiapine 307 (75-750)	54	6	14	chronic or subchronic schizophrenia with acute exacerbation (DSM-III-R)	2
Boulay 2007[36]	Olanzapine 12 (2.5-20)	14	8	1.68	schizophrenia or schizoaffective disorder (DSM-IV)	2
	Haloperidol 9.32 (2.5-20)	13	8	2.64	schizophrenia or schizoaffective disorder (DSM-IV)	2
Bratfos 1979[37]	Chlorpromazine 450 (225-675)	39	4	n.i.	schizophrenia, reactive psychoses, chronic psychoses, paranoid psychoses (clinical Diagnosis)	2
	Sulpiride 1200 (600-1800)	32	4	n.i.	schizophrenia, reactive psychoses, chronic psychoses, paranoid psychoses (clinical Diagnosis)	2
Brauzer 1971[38]	Molindone 25 (10-n.i.)	12	12	n.i.	schizophrenic, paranoid and non-paranoid, organic brain syndrome (clinical Diagnosis)	2
	Trifluoperazine 13 (5-n.i.)	13	12	n.i.	schizophrenic, paranoid and non-paranoid, organic brain syndrome (clinical Diagnosis)	2

Study name	Antipsychotics and daily dose in mg, on flexible dosage mean value (range)	N	Duration in weeks	Mean duration of illness in years	Diagnosis	Blinding
Brook 1998[39]	Haloperidol 10 (10-10)	20	4	n.i.	schizophrenia or schizophreniform disorder (clinical Diagnosis)	2
	Zuclopenthixol 25 (25-25)	24	4	n.i.	schizophrenia or schizophreniform disorder (clinical Diagnosis)	2
Brook 2005[40]	Haloperidol 11.5 (5-20)	138	6	8.78	schizophrenia or schizoaffective disorder, acute exacerbation (DSM-IV)	1
	Ziprasidone 116 (80-160)	429	6	9.2	schizophrenia or schizoaffective disorder, acute exacerbation (DSM-IV)	1
Buchsbaum 2009[41]	Haloperidol 10 (4-16)	n.i.	6	n.i.	schizophrenia (clinical Diagnosis)	2
	Sertindole 18 (12-24)	n.i.	6	n.i.	schizophrenia (clinical Diagnosis)	2
Bueno 1979[42]	Haloperidol 5.4 (2-n.i.)	20	6	4.26	schizophrenia (clinical Diagnosis)	2
	Loxapine 58.3 (20-n.i.)	20	6	4.42	schizophrenia (clinical Diagnosis)	2
Bugarski-Kirola[43]	Placebo	79	4	12.5	acute exacerbation of schizophrenia (DSM-IV)	2
	Olanzapine 15 (15-15)	63	4	13.9	acute exacerbation of schizophrenia (DSM-IV)	2
Burnett 1975[44]	Chlorpromazine 375 (150-600)	n.i.	4	23.1	chronic schizophrenic (clinical Diagnosis)	2
	Thiothixene 37.5 (15-60)	n.i.	4	23.1	chronic schizophrenic (clinical Diagnosis)	2
Butler 2000[45]	Thioridazine 187.5 (75-300)	11	5	5.7	chronic schizophrenia (clinical diagnosis)	2

Study name	Antipsychotics and daily dose in mg, on flexible dosage mean value (range)	N	Duration in weeks	Mean duration of illness in years	Diagnosis	Blinding
	Zotepine 187.5 (75-300)	12	5	7	chronic schizophrenia (clinical diagnosis)	2
Camara 1978[46]	Loxapine 137.9 (25-150)	25	8	n.i.	schizophrenia (clinical Diagnosis)	0
	Thioridazine 264.68 (100-800)	25	8	n.i.	schizophrenia (clinical Diagnosis)	0
Canive 2006[47]	Risperidone 6 (0-6)	4	8	n.i.	schizophrenia (DSM-IV)	2
	Olanzapine 15 (n.i.-15)	5	8	n.i.	schizophrenia (DSM-IV)	2
Cantillon 2014[48]	Aripiprazole 15 (15-15)	20	4	n.i.	subjects with an acute exacerbation of schizophrenia or<br><br>schizoaffective disorder (DSM-IV-TR)	2
	Placebo	39	4	n.i.	subjects with an acute exacerbation of schizophrenia or<br><br>schizoaffective disorder (DSM-IV-TR)	2
Carranza 1974[49]	Sulpiride 900 (600-1200)	20	8	n.i.	paranoid schizophrenia (clinical Diagnosis)	2
	Thioridazine 600 (300-900)	20	8	n.i.	paranoid schizophrenia (clinical Diagnosis)	2
Casey 1960[50]	Placebo	178	12	10	Schizophrenic reaction, chronic and acute (clinical diagnosis)	2
	Chlorpromazine 400 (400-400)	170	12	10	Schizophrenic reaction, chronic and acute (clinical diagnosis)	2
Casey 2008[51]	Risperidone 6 (6-6)	120	6	n.i.	patients with an acute	2

Study name	Antipsychotics and daily dose in mg, on flexible dosage mean value (range)	N	Duration in weeks	Mean duration of illness in years	Diagnosis	Blinding
					exacerbation of schizophrenia (DSM-IV-TR)	
	Placebo	119	6	n.i.	patients with an acute<br><br>exacerbation of schizophrenia (DSM-IV-TR)	2
Cassano 1975[52]	Haloperidol 5 (0.5-10.5)	36	4.29	n.i.	Paranoid or hebephrenic schizophrenia (clinical Diagnosis)	2
	Sulpiride 1000 (100-2300)	34	4.29	n.i.	Paranoid or hebephrenic schizophrenia (clinical Diagnosis)	2
Cavallaro 2001[53]	Haloperidol 6.5 (5-10)	16	6	1.7	subchronic schizophrenia (DSM-III-R)	2
	Risperidone 6.25 (5-10)	17	6	1.4	subchronic schizophrenia (DSM-III-R)	2
Ceskova 1993[54]	Haloperidol 6.4 (2.9-9.9)	31	8	12.5	acute schizophrenia or schizoaffective psychosis (ICD-9)	2
	Risperidone 6 (2.5-9.5)	31	8	8.3	acute schizophrenia or schizoaffective psychosis (ICD-9)	2
Ceskova 1994[55]	Perphenazine n.i. (n.i.-n.i.)	n.i.	3	8.5	acute attack or exacerbation of schizo-<br><br>phrenic disease (ICD-10)	2
	Risperidone n.i. (n.i.-n.i.)	n.i.	3	8.5	acute attack or exacerbation of schizo-<br><br>phrenic disease (ICD-10)	2
Cetin 2010[56]	Amisulpride n.i. (n.i.-n.i.)	10	12	n.i.	schizophrenia (clinical Diagnosis)	0

Study name	Antipsychotics and daily dose in mg, on flexible dosage mean value (range)	N	Duration in weeks	Mean duration of illness in years	Diagnosis	Blinding
	Aripiprazole n.i. (n.i.-n.i.)	10	12	n.i.	schizophrenia (clinical Diagnosis)	0
	Paliperidone n.i. (n.i.-n.i.)	10	12	n.i.	schizophrenia (clinical Diagnosis)	0
	Quetiapine n.i. (n.i.-n.i.)	10	12	n.i.	schizophrenia (clinical Diagnosis)	0
Chan 2007[57]	Risperidone 6 (6-6)	34	4	n.i.	schizophrenia or schizoaffective disorder with an acute relapse (DSM-IV)	2
	Aripiprazole 15 (15-15)	49	4	n.i.	schizophrenia or schizoaffective disorder with an acute relapse (DSM-IV)	2
Charalampous 1974, 00559[58]	Loxapine 147.5 (50-150)	20	4	2.5	schizophrenia (clinical diagnosis)	2
	Thiothixene 51.9 (20-60)	21	4	2.5	schizophrenia (clinical diagnosis)	2
	Placebo	19	4	2.5	schizophrenia (clinical diagnosis)	2
Chen 2018[59]	Olanzapine 15 (10-20)	53	12	8.96	schizophrenia (DSM-IV)	0
	Risperidone 5 (4-6)	28	12	7.24	schizophrenia (DSM-IV)	0
	Paliperidone 9 (6-12)	30	12	10	schizophrenia (DSM-IV)	0
Chiu 1976[60]	Chlorpromazine 300 (300-300)	31	6	6.4	schizophrenia (clinical diagnosis)	2
	Clozapine 300 (300-300)	33	6	6.4	schizophrenia (clinical diagnosis)	2
Chouinard 1970[61]	Fluphenazine 9 (3-15)	10	12	n.i.	schizophrenia (clinical Diagnosis)	2

Study name	Antipsychotics and daily dose in mg, on flexible dosage mean value (range)	N	Duration in weeks	Mean duration of illness in years	Diagnosis	Blinding
	Pimozide 12 (4-20)	10	12	n.i.	schizophrenia (clinical Diagnosis)	2
Chouinard 1975[62]	Placebo	24	12	n.i.	schizophrenia (clinical Diagnosis)	2
	Perphenazine 20 (20-20)	24	12	n.i.	schizophrenia (clinical Diagnosis)	2
Chouinard 1976[63]	Chlorpromazine 600 (300-900)	19	3	n.i.	schizophrenia (DSM-II)	2
	Penfluridol 80 (40-120)	14	3	n.i.	schizophrenia (DSM-II)	2
Chouinard 1982[64]	Chlorpromazine 960 (300-n.i.)	20	4	n.i.	schizophrenia (Research Diagnostic Criteria)	2
	Pimozide 37.8 (10-n.i.)	20	4	n.i.	schizophrenia (Research Diagnostic Criteria)	2
Chouinard 1990[65]	Chlorpromazine 555 (300-1200)	21	4	14.5	schizophrenia (DSM-III)	2
	Placebo	21	4	13.4	schizophrenia (DSM-III)	2
Chouinard 1993[66]	Placebo	22	8	16	chronic schizophrenia (DSM-III-R)	2
	Risperidone 6 (6-6)	22	8	16	chronic schizophrenia (DSM-III-R)	2
	Haloperidol 20 (20-20)	21	8	16	chronic schizophrenia (DSM-III-R)	2
Chung 2000[67]	Haloperidol 10.67 (9-15)	15	6	3.46	schizophrenia, schizophreniform disorder, schizoaffective disorder, delusional disorder, brief psychotic disorder (DSM-IV)	0

Study name	Antipsychotics and daily dose in mg, on flexible dosage mean value (range)	N	Duration in weeks	Mean duration of illness in years	Diagnosis	Blinding
	Risperidone 4.83 (n.i.-n.i.)	18	6	3.11	schizophrenia, schizophreniform disorder, schizoaffective<br><br>disorder, delusional disorder, brief psychotic disorder (DSM-IV)	0
Chung 2012[68]	Olanzapine 16.65 (n.i.-n.i.)	60	12	8.86	schizophrenia (DSM-IV-TR)	0
	Paliperidone 7.59 (n.i.-n.i.)	30	12	10.21	schizophrenia (DSM-IV-TR)	0
	Risperidone 4.6 (n.i.-n.i.)	30	12	10.61	schizophrenia (DSM-IV-TR)	0
Citrome 2015[69]	Aripiprazole 18.2 (10-20)	33	6	19.3	schizophrenia (DSM-IV-TR)	0
	Brexpiprazole 3.58 (1-4)	64	6	16.6	schizophrenia (DSM-IV-TR)	0
Ciurezu 1976[70]	Haloperidol 9 (4-20)	20	5.7	n.i.	schizophrenia (clinical Diagnosis)	2
	Clozapine 402 (100-900)	20	5.7	n.i.	schizophrenia (clinical Diagnosis)	2
Clark 1967[71]	Chlorpromazine 678 (n.i.-800)	51	10	n.i.	chronic schizophrenia (clinical diagnosis)	2
	Placebo	21	10	n.i.	chronic schizophrenia (clinical diagnosis)	2
Clark 1969[72]	Placebo	16	12	n.i.	chronic schizophrenia (clinical diagnosis)	2
	Haloperidol 9 (3-15)	14	12	n.i.	chronic schizophrenia (clinical diagnosis)	2
	Chlorpromazine 900 (200-1600)	14	12	n.i.	chronic schizophrenia (clinical diagnosis)	2

Study name	Antipsychotics and daily dose in mg, on flexible dosage mean value (range)	N	Duration in weeks	Mean duration of illness in years	Diagnosis	Blinding
Clark 1970a[73]	Chlorpromazine 684 (200-1000)	15	12	n.i.	chronic schizophrenia (clinical diagnosis)	2
	Molindone 68.2 (20-100)	15	12	n.i.	chronic schizophrenia (clinical diagnosis)	2
	Placebo	14	12	n.i.	chronic schizophrenia (clinical diagnosis)	2
Clark 1971a[74]	Thioridazine 760 (0-1000)	22	4	n.i.	acutely exacerbated chronic schizophrenia (clinical diagnosis)	2
	Placebo	21	4	n.i.	acutely exacerbated chronic schizophrenia (clinical diagnosis)	2
	Fluphenazine 7.28 (0-10)	20	4	n.i.	acutely exacerbated chronic schizophrenia (clinical diagnosis)	2
	Chlorpromazine 718 (0-1000)	23	4	n.i.	acutely exacerbated chronic schizophrenia (clinical diagnosis)	2
Clark 1972[75]	Chlorpromazine 816.71 (200-1000)	19	12	n.i.	chronic schizophrenia (clinical diagnosis)	2
	Loxapine 80.62 (20-100)	18	12	n.i.	chronic schizophrenia (clinical diagnosis)	2
	Placebo	18	12	n.i.	chronic schizophrenia (clinical diagnosis)	2
Clark 1975[76]	Placebo	13	4	9.5	chronic schizophrenia (clinical diagnosis)	2
	Trifluoperazine 36 (10-50)	15	4	9.9	chronic schizophrenia (clinical diagnosis)	2
	Loxapine 71 (20-100)	15	4	7.6	chronic schizophrenia (clinical diagnosis)	2
Clark 1977 12 weeks, 02912[77]	Placebo	13	12	17.08	chronic schizophrenic patients (DSM-II)	2

**Study name**	**Antipsychotics and daily dose in mg, on flexible dosage mean value (range)**	**N**	**Duration in weeks**	**Mean duration of illness in years**	**Diagnosis**	**Blinding**
	Loxapine 69.232 (40-50)	25	12	#NV	chronic schizophrenic patients (DSM-II)	2
Clark 1977a[78]	Chlorpromazine 550 (100-1000)	9	12	17.89	chronic schizophrenia (clinical diagnosis)	2
	Placebo	9	12	25.67	chronic schizophrenia (clinical diagnosis)	2
Claus 1992[79]	Haloperidol 10.3 (10.3-10.3)	22	12	13.6	schizophrenia with chronic course (DSM-III-R)	2
	Risperidone 12 (12-12)	22	12	14.6	schizophrenia with chronic course (DSM-III-R)	2
Conley 2001[80]	Olanzapine 13.1 (5-20)	189	8	15.4	schizophrenia or schizoaffective disorder (DSM-IV)	2
	Risperidone 4.7 (2-6)	188	8	16.5	schizophrenia or schizoaffective disorder (DSM-IV)	2
Cooper 2000a[81]	Placebo	53	8	10.28	acute episode of schizophrenia or acute exacerbation of (sub-)chronic schizophrenia (DSM-III-R)	2
	Chlorpromazine 400 (200-600)	53	8	11.88	acute episode of schizophrenia or acute exacerbation of (sub-)chronic schizophrenia (DSM-III-R)	2
	Zotepine 225 (150-300)	53	8	10.78	acute episode of schizophrenia or acute exacerbation of (sub-)chronic schizophrenia (DSM-III-R)	2
Copolov 2000[82]	Quetiapine 455 (300-800)	221	6	12	acute exacerbation of chronic or subchronic schizophrenia (DSM-III-R)	2

Study name	Antipsychotics and daily dose in mg, on flexible dosage mean value (range)	N	Duration in weeks	Mean duration of illness in years	Diagnosis	Blinding
	Haloperidol 8 (6-16)	227	6	12	acute exacerbation of chronic or subchronic schizophrenia (DSM-III-R)	2
Correll 2015[83]	Placebo	184	6	12.3	schizophrenia (DSM-IV-TR)	2
	Brexpiprazole 2.99447513812155 (2-4)	362	6	#NV	schizophrenia (DSM-IV-TR)	2
Corrigan 2004[84]	Placebo	87	6	14.2	schizophrenia (DSM-IV)	2
	Olanzapine 15 (15-15)	93	6	12.3	schizophrenia (DSM-IV)	2
Corripio 2005[85]	Ziprasidone 100 (80-120)	10	n.i.	n.i.	schizophreniform disorders or schizophrenia with acute psychosis exacerbation (DSM-IV)	1
	Haloperidol 12.5 (5-20)	10	n.i.	n.i.	schizophreniform disorders or schizophrenia with acute psychosis exacerbation (DSM-IV)	1
Cosar 1999[86]	Chlorpromazine 454 (n.i.-n.i.)	40	13	n.i.	chronic schizophrenia (DSM-IV)	0
	Clozapine 462.661 (n.i.-n.i.)	40	13	n.i.	chronic schizophrenia (DSM-IV)	0
	Haloperidol 34.75 (n.i.-n.i.)	40	13	n.i.	chronic schizophrenia (DSM-IV)	0
	Sulpiride 696 (n.i.-n.i.)	40	13	n.i.	chronic schizophrenia (DSM-IV)	0
Costa e Silva 1989[87]	Haloperidol 28 (20-30)	20	3	n.i.	schizophrenia (ICD-9)	2
	Amisulpride 850 (800-1200)	20	3	n.i.	schizophrenia (ICD-9)	2

Study name	Antipsychotics and daily dose in mg, on flexible dosage mean value (range)	N	Duration in weeks	Mean duration of illness in years	Diagnosis	Blinding
Crowley 1981[88]	Thiothixene 3.2 (2-n.i.)	n.i.	3	n.i.	schizophrenia (clinical Diagnosis)	2
	Thioridazine 80 (50-n.i.)	n.i.	3	n.i.	schizophrenia (clinical Diagnosis)	2
Cutler 2006[89]	Placebo	88	6	18.3	schizophrenia acute relapse (DSM-IV)	2
	Aripiprazole 10 (10-10)	94	6	14.8	schizophrenia acute relapse (DSM-IV)	2
Cutler 2008[90]	Iloperidone 24 (24-24)	303	4	n.i.	schizophrenia (DSM-IV)	2
	Ziprasidone 160 (160-160)	151	4	n.i.	schizophrenia (DSM-IV)	2
	Placebo	152	4	n.i.	schizophrenia (DSM-IV)	2
Cutler 2008a[91]	Placebo	117	6	18.3	Acute schizophrenia<br><br>schizophrenia (DSM-IV)	2
	Quetiapine 651.339285714286 (600-400)	448	6	#NV	Acute schizophrenia<br><br>schizophrenia (DSM-IV)	2
Daniel 1999[92]	Placebo	92	6	14.7	acute exacerbation of (sub-) chronic schizophrenia or schizoaffective disorder (DSM-III-R)	2
	Ziprasidone 160 (160-160)	104	6	14.4	acute exacerbation of (sub-) chronic schizophrenia or schizoaffective disorder (DSM-III-R)	2
Davidson 2007[93]	Placebo	123	6	12.8	schizophrenia acute episode (DSM-IV)	2
	Olanzapine 10 (10-10)	128	6	11.9	schizophrenia acute episode (DSM-IV)	2

Study name	Antipsychotics and daily dose in mg, on flexible dosage mean value (range)	N	Duration in weeks	Mean duration of illness in years	Diagnosis	Blinding
	Paliperidone 9 (9-9)	125	6	11	schizophrenia acute episode (DSM-IV)	2
de Boer 2011[94]	Aripiprazole 12.6 (7.5-30)	23	6	n.i.	schizophrenia or related psychiatric disorders (DSM-IV)	0
	Risperidone 3.2 (1-6)	21	6	n.i.	schizophrenia or related psychiatric disorders (DSM-IV)	0
de Oliviera 2009[95]	Aripiprazole 19.4 (15-30)	66	8	12	schizophrenia or schizoaffective disorder (DSM-IV-TR)	0
	Haloperidol 10.1 (10-15)	33	8	11.5	schizophrenia or schizoaffective disorder (DSM-IV-TR)	0
Dehnel 1968[96]	Clopenthixol 132.5 (25-250)	25	12	n.i.	schizophrenia (clinical Diagnosis)	2
	Perphenazine 51.2 (8-80)	25	12	n.i.	schizophrenia (clinical Diagnosis)	2
DeJong 1965[97]	Thioridazine 300 (100-500)	n.i.	13	n.i.	chronic schizophrenia (clinical Diagnosis)	2
	Trifluoperazine 12 (4-20)	n.i.	13	n.i.	chronic schizophrenia (clinical Diagnosis)	2
Delcker 1990[98]	Haloperidol 22.5 (5-40)	20	6	14.3	schizophrenia (ICD-9)	2
	Amisulpride 600 (490-1000)	21	6	17.3	schizophrenia (ICD-9)	2
dElia 1974[99]	Pimozide 3.9 (3-5)	10	5	n.i.	chronic schizophrenia (clinical Diagnosis)	0
	Trifluoperazine 4.95 (3-7.5)	10	5	n.i.	chronic schizophrenia (clinical Diagnosis)	0
DeMartinis 2012[100]	Risperidone 6 (6-6)	37	4	n.i.	schizophrenia (DSM-IV-TR)	2

Study name	Antipsychotics and daily dose in mg, on flexible dosage mean value (range)	N	Duration in weeks	Mean duration of illness in years	Diagnosis	Blinding
	Placebo	74	4	n.i.	schizophrenia (DSM-IV-TR)	2
Denber 1972[101]	Thiothixene 49.06 (5-60)	15	5.14	n.i.	schizophrenia (clinical Diagnosis)	2
	Trifluoperazine 58.7 (5-60)	15	5.14	n.i.	schizophrenia (clinical Diagnosis)	2
Dieterle 1991[102]	Perazine 350 (75-675)	20	4	n.i.	schizophrenia or schizoaffective disorder (ICD-9)	2
	Zotepine 240 (50-450)	20	4	n.i.	schizophrenia or schizoaffective disorder (ICD-9)	2
Dolnak 1996[103]	Haloperidol 12.5 (5-20)	n.i.	12	n.i.	schizophrenia or schizoaffective disorder (DSM-III-R)	2
	Olanzapine 12.5 (5-20)	n.i.	12	n.i.	schizophrenia or schizoaffective disorder (DSM-III-R)	2
Dolnak 2001[104]	Olanzapine n.i. (n.i.-n.i.)	n.i.	8	n.i.	schizophrenia (DSM-IV)	2
	Risperidone n.i. (n.i.-n.i.)	n.i.	8	n.i.	schizophrenia (DSM-IV)	2
Dossenbach 2007[105]	Olanzapine 14.9 (5-20)	83	6	10.2	schizophrenia (DSM-IV)	0
	Chlorpromazine 433.1 (200-800)	40	6	10.2	schizophrenia (DSM-IV)	0
Downing 2014[106]	Placebo	295	6	14.5	schizophrenia who had experienced an exacerbation of symptoms (DSM-IV)	2

Study name	Antipsychotics and daily dose in mg, on flexible dosage mean value (range)	N	Duration in weeks	Mean duration of illness in years	Diagnosis	Blinding
	Risperidone 4 (4-4)	143	6	15.2	schizophrenia who had experienced an exacerbation of symptoms (DSM-IV)	2
Dube 1976[107]	Chlorpromazine 320 (200-800)	26	12	n.i.	symptoms of functional psychoses (clinical Diagnosis)	2
	Loxapine 34.3 (20-80)	26	12	n.i.	symptoms of functional psychoses (clinical Diagnosis)	2
Duggan 2005[108]	Olanzapine 12.5 (5-20)	53	6	n.i.	schizophrenia, schizophreniform and schizoaffective disorder (DSM-IV)	0
	Haloperidol 10.75 (1.5-20)	51	6	n.i.	schizophrenia, schizophreniform and schizoaffective disorder (DSM-IV)	0
Durgam 2014[109]	Risperidone 4 (4-4)	140	6	12.3	acute exacerbation of schizophrenia (DSM-IV-TR)	2
	Placebo	151	6	11.6	acute exacerbation of schizophrenia (DSM-IV-TR)	2
	Cariprazine 3.00684931506849 (1.5-3)	438	6	#NV	acute exacerbation of schizophrenia (DSM-IV-TR)	2
Dutta 2014[110]	Asenapine n.i. (n.i.-n.i.)	41	24	n.i.	schizophrenia (DSM-V)	1
	Clozapine n.i. (n.i.-n.i.)	38	24	n.i.	schizophrenia (DSM-V)	1
	Ziprasidone n.i. (n.i.-n.i.)	40	24	n.i.	schizophrenia (DSM-V)	1
Ebrinc 2004[111]	Amisulpride 567 (400-800)	20	6	n.i.	schizophrenia (DSM-IV)	0

Study name	Antipsychotics and daily dose in mg, on flexible dosage mean value (range)	N	Duration in weeks	Mean duration of illness in years	Diagnosis	Blinding
	Haloperidol 18.66 (15-30)	20	6	n.i.	schizophrenia (DSM-IV)	0
Edwards 1980[112]	Sulpiride 1212 (600-1800)	19	6	n.i.	schizophrenia (clinical Diagnosis)	2
	Trifluoperazine 27.7 (15-45)	19	6	n.i.	schizophrenia (clinical Diagnosis)	2
Egan 2013[113]	Olanzapine 15 (15-15)	47	4	10.7	schizophrenia, acutly psychotic (DSM-IV-TR)	2
	Placebo	83	4	11.2	schizophrenia, acutly psychotic (DSM-IV-TR)	2
Ehmann 1987[114]	Flupentixol 27 (8-84)	n.i.	12	n.i.	schizophrenia (DSM-III)	2
	Haloperidol 33 (10-84)	n.i.	12	n.i.	schizophrenia (DSM-III)	2
Ehrlich 2012[115]	Olanzapine 14.95 (7.5-30)	21	12	n.i.	schizophrenia or schizoaffective (DSM-IV)	0
	Ziprasidone 102.63 (60-160)	16	12	n.i.	schizophrenia or schizoaffective (DSM-IV)	0
Engelhardt 1969, 03748[116]	Chlorpromazine 180 (50-800)	103	12	n.i.	chronic schizophrenia (clinical diagnosis)	2
	Placebo	99	12	n.i.	chronic schizophrenia (clinical diagnosis)	2
ENLIGHTEN-1[117]	Olanzapine 18.4 (n.i.-n.i.)	133	4	n.i.	acute exacerbation of schizophrenia (clinical Diagnosis)	2
	Placebo	134	4	n.i.	acute exacerbation of schizophrenia (clinical Diagnosis)	2
Erlandsen 1981[118]	Clozapine 225 (50-400)	19	5.7	15	schizophrenia (clinical Diagnosis)	2

Study name	Antipsychotics and daily dose in mg, on flexible dosage mean value (range)	N	Duration in weeks	Mean duration of illness in years	Diagnosis	Blinding
	Haloperidol 4.5 (1-8)	21	5.7	15	schizophrenia (clinical Diagnosis)	2
Escobar 1985[119]	Haloperidol 32.4 (10-60)	15	4	n.i.	schizophrenic disorder (DSM-III)	2
	Molindone 160 (50-400)	15	4	n.i.	schizophrenic disorder (DSM-III)	2
Evans 1972[120]	Placebo	27	3	n.i.	newly hospitalized schizophrenic patients (clinical diagnosis)	2
	Thioridazine 400 (400-400)	27	3	n.i.	newly hospitalized schizophrenic patients (clinical diagnosis)	2
Fabre 1995[121]	Placebo	4	3	12	(sub-)chronic schizophrenia (DSM-III-R)	2
	Quetiapine 137.5 (25-250)	8	3	12	(sub-)chronic schizophrenia (DSM-III-R)	2
Fakra 2008[122]	Haloperidol 7.96 (n.i.-n.i.)	15	4	16.9	schizophrenia (DSM-IV)	0
	Risperidone 5.78 (n.i.-n.i.)	15	4	13.3	schizophrenia (DSM-IV)	0
Faustman 1995[123]	Quetiapine 375 (250-500)	5	6	n.i.	schizophrenic (clinical Diagnosis)	2
	Placebo 0 (n.i.-n.i.)	4	6	n.i.	schizophrenic (clinical Diagnosis)	2
Fischer-Cornellsen 1976a[124]	Clopenthixol 100 (n.i.-n.i.)	36	6	n.i.	Moderate to severe acute paranoid schizophrenics (clinical Diagnosis)	2
	Clozapine n.i. (n.i.-n.i.)	38	6	n.i.	Moderate to severe acute paranoid schizophrenics (clinical Diagnosis)	2

Study name	Antipsychotics and daily dose in mg, on flexible dosage mean value (range)	N	Duration in weeks	Mean duration of illness in years	Diagnosis	Blinding
Fischer-Cornellsen 1976b[124]	Trifluoperazine 30 (n.i.-n.i.)	36	6	n.i.	Moderate to severe acute paranoid schizophrenics (clinical Diagnosis)	2
	Clozapine n.i. (n.i.-n.i.)	36	6	n.i.	Moderate to severe acute paranoid schizophrenics (clinical Diagnosis)	2
Fleischhacker 1989[125]	Haloperidol 14.5 (n.i.-n.i.)	20	6	n.i.	paranoid schizophrenia (DSM-III)	2
	Zotepine 309 (n.i.-n.i.)	20	6	n.i.	paranoid schizophrenia (DSM-III)	2
Fleischhacker 2009_6weeks[126]	Olanzapine 15.4 (10-20)	348	6	10.5	schizophrenia, acute relapse (DSM-IV)	2
	Aripiprazole 23 (15-30)	355	6	9.7	schizophrenia, acute relapse (DSM-IV)	2
Fleming 1959[127]	Chlorpromazine 113 (50-150)	12	4	16.7	psychotic, mostly chronic schizophrenia (clinical diagnosis)	2
	Placebo	12	4	16.7	psychotic, mostly chronic schizophrenia (clinical diagnosis)	2
Fleming 1968[128]	Chlorpromazine n.i. (n.i.-n.i.)	n.i.	4	16.7	schizophrenics (clinical Diagnosis)	2
	Placebo n.i. (n.i.-n.i.)	n.i.	4	16.7	schizophrenics (clinical Diagnosis)	2
Freeman 1969[129]	Molindone 80 (20-140)	20	8	n.i.	schizophrenia (clinical Diagnosis)	2
	Trifluoperazine 40 (10-70)	20	8	n.i.	schizophrenia (clinical Diagnosis)	2
Fruensgaard 1978acute[130]	Loxapine 54.4 (20-150)	12	3	n.i.	schizophrenia (clinical Diagnosis)	2
	Perphenazine 34 (16-120)	10	3	n.i.	schizophrenia (clinical Diagnosis)	2

Study name	Antipsychotics and daily dose in mg, on flexible dosage mean value (range)	N	Duration in weeks	Mean duration of illness in years	Diagnosis	Blinding
Fruensgaard 1978chronic[131]	Loxapine 81.1 (20-150)	11	12	n.i.	schizophrenia (clinical Diagnosis)	2
	Perphenazine 90.1 (16-120)	14	12	n.i.	schizophrenia (clinical Diagnosis)	2
Gallant 1963[132]	Chlorpromazine 400 (0-800)	n.i.	12	n.i.	chronic schizophrenics (clinical diagnosis)	2
	Placebo	n.i.	12	n.i.	chronic schizophrenics (clinical diagnosis)	2
Gallant 1966[133]	Thioridazine 450 (100-800)	18	11	n.i.	chronic schizophrenic patients (clinical Diagnosis)	2
	Thiothixene 11.25 (2.5-20)	18	11	n.i.	chronic schizophrenic patients (clinical Diagnosis)	2
Gallant 1967[134]	Chlorpromazine 400 (0-800)	19	4	n.i.	acute schizophrenia (clinical Diagnosis)	2
	Haloperidol 8 (0-16)	19	4	n.i.	acute schizophrenia (clinical Diagnosis)	2
Gallant 1968[135]	Molindone 45 (10-80)	12	11	n.i.	chronic schizophrenia (clinical Diagnosis)	2
	Trifluoperazine 45 (10-80)	12	11	n.i.	chronic schizophrenia (clinical Diagnosis)	2
Garcia 2009[136]	Haloperidol 10 (10-10)	60	6	n.i.	acute exacerbation of schizophrenia (DSM-IV-TR)	2
	Placebo	64	6	n.i.	acute exacerbation of schizophrenia (DSM-IV-TR)	2
Garry 1962b[137]	Placebo	26	12	12.2	chronic schizophrenia (clinical diagnosis)	2
	Haloperidol 4.2 (0.75-6)	26	12	16.8	chronic schizophrenia (clinical diagnosis)	2

Study name	Antipsychotics and daily dose in mg, on flexible dosage mean value (range)	N	Duration in weeks	Mean duration of illness in years	Diagnosis	Blinding
Gattaz 2004[138]	Flupentixol 12.5 (5-20)	13	4	5.5	schizophrenia (DSM-IV)	2
	Olanzapine 12.5 (5-20)	15	4	5	schizophrenia (DSM-IV)	2
Geffen 2012[139]	Risperidone 6.8 (2-8)	91	6	8.26	acute exacerbation of chronic schizophrenia (DSM-IV-TR)	2
	Placebo	93	6	9.34	acute exacerbation of chronic schizophrenia (DSM-IV-TR)	2
Gelenberg 1979[140]	Chlorpromazine 606 (50-1800)	8	4	n.i.	schizophrenia (DSM-II)	2
	Clozapine 279 (25-900)	7	4	n.i.	schizophrenia (DSM-II)	2
Gerlach 1974[141]	Clozapine 200 (100-800)	10	11.7	15	schizophrenia (clinical Diagnosis)	1
	Haloperidol 10 (3-32)	10	11.7	15	schizophrenia (clinical Diagnosis)	1
Gerlach 1975[142]	Clozapine 225 (225-225)	4	3	27.25	schizophrenia (clinical Diagnosis)	2
	Haloperidol 9 (9-9)	4	3	27.25	schizophrenia (clinical Diagnosis)	2
Ghaleiha 2011[143]	Risperidone 6 (6-6)	17	8	7.95	chronic schizophrenia (DSM-IV-TR)	2
	Clozapine 300 (300-300)	17	8	7.77	chronic schizophrenia (DSM-IV-TR)	2
	Haloperidol 15 (15-15)	17	8	7.26	chronic schizophrenia (DSM-IV-TR)	2
Goff 1998[144]	Haloperidol 15 (15-15)	17	4	13.6	chronic or subchronic schizophrenia or schizoaffective disorder (DSM-III-R)	2

Study name	Antipsychotics and daily dose in mg, on flexible dosage mean value (range)	N	Duration in weeks	Mean duration of illness in years	Diagnosis	Blinding
	Ziprasidone 160 (160-160)	20	4	18.3	chronic or subchronic schizophrenia or schizoaffective disorder (DSM-III-R)	2
Goldberg 1972[145]	Chlorpromazine 680 (200-1600)	n.i.	5	n.i.	acutely ill schizophrenic patients (clinical diagnosis)	2
	Placebo	n.i.	5	n.i.	acutely ill schizophrenic patients (clinical diagnosis)	2
Goldstein 1966[146]	Haloperidol 15 (10-20)	10	n.i.	n.i.	acute psychosis (clinical Diagnosis)	2
	Trifluoperazine 13.5 (7-20)	8	n.i.	n.i.	acute psychosis (clinical Diagnosis)	2
Goldstein 1969[147]	Haloperidol n.i. (n.i.-n.i.)	n.i.	5	n.i.	psychosis (clinical Diagnosis)	2
	Perphenazine n.i. (n.i.-n.i.)	n.i.	5	n.i.	psychosis (clinical Diagnosis)	2
Gowardman 1973[148]	Haloperidol 10.15 (n.i.-14)	10	13	18	schizophrenia (clinical Diagnosis)	2
	Pimozide 5.1 (n.i.-6)	10	13	23.2	schizophrenia (clinical Diagnosis)	2
Granacher 1982[149]	Thioridazine 450 (100-800)	27	12	n.i.	psychotic inpatients (clinical Diagnosis)	2
	Thiothixene 35 (10-60)	27	12	n.i.	psychotic inpatients (clinical Diagnosis)	2
Grootens 2009[150]	Ziprasidone 104 (80-160)	39	8	n.i.	schizophrenia, schizoaffective disorder, or schizophreniform disorder (DSM-IV)	2
	Olanzapine 14 (10-20)	35	8	n.i.	schizophrenia, schizoaffective disorder, or schizophreniform disorder (DSM-IV)	2

Study name	Antipsychotics and daily dose in mg, on flexible dosage mean value (range)	N	Duration in weeks	Mean duration of illness in years	Diagnosis	Blinding
Guirguis 1977[151]	Clozapine 262.5 (75-450)	22	7	8.1	acute schizophrenia (clinical Diagnosis)	2
	Chlorpromazine 525 (150-900)	28	7	8.4	acute schizophrenia (clinical Diagnosis)	2
Gupta 2017[152]	Quetiapine n.i. (n.i.-n.i.)	10	8	n.i.	schizophrenia (DSM-V)	0
	Risperidone n.i. (n.i.-n.i.)	10	8	n.i.	schizophrenia (DSM-V)	0
	Aripiprazole n.i. (n.i.-n.i.)	10	8	n.i.	schizophrenia (DSM-V)	0
	Olanzapine n.i. (n.i.-n.i.)	10	8	n.i.	schizophrenia (DSM-V)	0
Guz 2002[153]	Olanzapine 15 (10-20)	22	8	n.i.	schizophrenia (DSM-IV)	0
	Risperidone 4 (2-6)	18	8	n.i.	schizophrenia (DSM-IV)	0
Haas 1982[154]	Pimozide 20.36 (10-60)	14	4	n.i.	schizophrenia (ICD-9)	2
	Haloperidol 23.75 (10-60)	15	4	n.i.	schizophrenia (ICD-9)	2
Hadlik 1970[155]	Thiothixene 30 (30-30)	n.i.	3	n.i.	schizophrenia (clinical Diagnosis)	2
	Perphenazine 24 (24-24)	n.i.	3	n.i.	schizophrenia (clinical Diagnosis)	2
Hale 2000[156]	Haloperidol 10 (10-10)	125	8	9.5	schizophrenia (DSM-III-R)	2
	Sertindole 18 (16-20)	255	8	7.76	schizophrenia (DSM-III-R)	2
Hall 1955[157]	Chlorpromazine 600 (450-750)	87	9	n.i.	semi-disturbed schizophrenia (clinical diagnosis)	2

Study name	Antipsychotics and daily dose in mg, on flexible dosage mean value (range)	N	Duration in weeks	Mean duration of illness in years	Diagnosis	Blinding
	Placebo	88	9	n.i.	semi-disturbed schizophrenia (clinical diagnosis)	2
Hall 1968[158]	Haloperidol 6.95 (2-10)	25	12	n.i.	schizophrenic (clinical Diagnosis)	2
	Fluphenazine 17.86 (5-25)	25	12	n.i.	schizophrenic (clinical Diagnosis)	2
Harnryd 1984[159]	Chlorpromazine 400 (400-400)	25	8	n.i.	schizophrenia (clinical Diagnosis)	2
	Sulpiride 800 (800-800)	25	8	n.i.	schizophrenia (clinical Diagnosis)	2
Hatta 2009[160]	Olanzapine 17.4 (10-20)	17	8	12.86	schizophrenia, acute schizophrenia-like psychotic disorder, schizoaffective disorder (ICD-10)	1
	Quetiapine 579 (300-750)	20	8	8.49	schizophrenia, acute schizophrenia-like psychotic disorder, schizoaffective disorder (ICD-10)	1
	Aripiprazole 23.6 (12-30)	22	8	11.84	schizophrenia, acute schizophrenia-like psychotic disorder, schizoaffective disorder (ICD-10)	1
	Risperidone 7.2 (3-12)	21	8	15.95	schizophrenia, acute schizophrenia-like psychotic disorder, schizoaffective disorder (ICD-10)	1
Hatta 2013[161]	Olanzapine 23 (10-40)	22	8	9.95	schizophrenia, schizophreniform disorder, schizoaffective disorder (DSM-IV-TR)	1

Study name	Antipsychotics and daily dose in mg, on flexible dosage mean value (range)	N	Duration in weeks	Mean duration of illness in years	Diagnosis	Blinding
	Risperidone 6.9 (3-12)	20	8	7.43	schizophrenia, schizophreniform disorder, schizoaffective disorder (DSM-IV-TR)	1
Haug 1959[162]	Placebo 0 (n.i.-n.i.)	n.i.	4	n.i.	chronic psychotic patients (clinical Diagnosis)	2
	Thioridazine n.i. (n.i.-n.i.)	n.i.	4	n.i.	chronic psychotic patients (clinical Diagnosis)	2
Heikkila 1981[163]	Haloperidol 10 (2-24)	33	12	n.i.	schizophrenia or other psychosis (clinical Diagnosis)	2
	Zuclopenthixol 40 (10-75)	30	12	n.i.	schizophrenia or other psychosis (clinical Diagnosis)	2
Heikkilae 1992[164]	Haloperidol 10.3 (2-30)	23	8	n.i.	acute schizophrenia, exacerbation of chronic schizophrenia, paranoid states, reactive paranoid psychosis (ICD-9)	2
	Zuclopenthixol 33.5 (10-75)	26	8	n.i.	acute schizophrenia, exacerbation of chronic schizophrenia, paranoid states, reactive paranoid psychosis (ICD-9)	2
Heikkinen 1993[165]	Chlorpromazine 500 (200-500)	21	8	n.i.	acutely psychotic patients who fulfilled DSM-lll-R criteria for schizophrenia (DSM-III-R)	2
	Molindone 100 (100-100)	24	8	n.i.	acutely psychotic patients who fulfilled DSM-lll-R criteria for schizophrenia (DSM-III-R)	2

Study name	Antipsychotics and daily dose in mg, on flexible dosage mean value (range)	N	Duration in weeks	Mean duration of illness in years	Diagnosis	Blinding
Heinrich 1994[166]	Clozapine 400 (400-400)	20	4	n.i.	acute schizophrenia or schizoaffective disorder (ICD-9)	2
	Risperidone 6 (4-8)	40	4	n.i.	acute schizophrenia or schizoaffective disorder (ICD-9)	2
Hera 041-021[167]	Asenapine 14.9 (10-20)	208	6	n.i.	acute exacerbation of schizophrenia (DSM-IV)	2
	Placebo	106	6	n.i.	acute exacerbation of schizophrenia (DSM-IV)	2
	Olanzapine 15 (15-15)	103	6	n.i.	acute exacerbation of schizophrenia (DSM-IV)	2
Hera 041-022[168]	Asenapine 15 (10-20)	91	6	n.i.	acute exacerbation of schizophrenia (DSM-IV)	2
	Olanzapine 15 (10-20)	93	6	n.i.	acute exacerbation of schizophrenia (DSM-IV)	2
	Placebo	93	6	n.i.	acute exacerbation of schizophrenia (DSM-IV)	2
Herrera 1990, 00640[169]	Placebo	5	4	9.62	acute schizophrenia (DSM-III)	2
	Thioridazine 700 (400-1000)	9	4	9.62	acute schizophrenia (DSM-III)	2
Hogan 1992[170]	Chlorpromazine n.i. (n.i.-n.i.)	25	3	n.i.	schizophrenia (WHO Flexible System)	0
	Haloperidol n.i. (n.i.-n.i.)	30	3	n.i.	schizophrenia (WHO Flexible System)	0
Honigfeld 1984a[171]	Placebo	8	4	n.i.	acute schizophrenia (clinical Diagnosis)	2

Study name	Antipsychotics and daily dose in mg, on flexible dosage mean value (range)	N	Duration in weeks	Mean duration of illness in years	Diagnosis	Blinding
	Clozapine 608.25 (n.i.-900)	16	4	n.i.	acute schizophrenia (clinical Diagnosis)	2
	Chlorpromazine 1183.25 (n.i.-1800)	15	4	n.i.	acute schizophrenia (clinical Diagnosis)	2
Honigfeld 1984c[171]	Haloperidol 7.6 (n.i.-n.i.)	40	5.7	n.i.	schizophrenia (clinical Diagnosis)	2
	Clozapine 397 (n.i.-n.i.)	39	5.7	n.i.	schizophrenia (clinical Diagnosis)	2
Honigfeld 1984d[171]	Clozapine 310 (50-1000)	110	5.7	7	schizophrenia (ICD-9)	2
	Chlorpromazine 360 (25-900)	113	5.7	6.9	schizophrenia (ICD-9)	2
Howell 1961[172]	Fluphenazine 5 (5-5)	n.i.	8	n.i.	schizophrenia (clinical Diagnosis)	2
	Placebo	n.i.	8	n.i.	schizophrenia (clinical Diagnosis)	2
	Trifluoperazine 12.5 (12.5-12.5)	n.i.	8	n.i.	schizophrenia (clinical Diagnosis)	2
Hoyberg 1993[173]	Risperidone 8.5 (5-15)	55	8	n.i.	chronic schizophrenia with acute exacerbation (DSM-III-R)	2
	Perphenazine 28 (16-48)	52	8	n.i.	chronic schizophrenia with acute exacerbation (DSM-III-R)	2
Huttunen 1995[174]	Risperidone 8 (2-20)	48	6	11	acute exacerbation of schizophrenia or schizophreniform disorder (DSM-III-R)	2
	Zuclopenthixol 38 (10-100)	50	6	14	acute exacerbation of schizophrenia or schizophreniform disorder (DSM-III-R)	2

Study name	Antipsychotics and daily dose in mg, on flexible dosage mean value (range)	N	Duration in weeks	Mean duration of illness in years	Diagnosis	Blinding
Hwang 2001[175]	Zotepine 150 (150-150)	35	6	9.2	schizophrenia (ICD-10)	2
	Haloperidol 9 (9-9)	35	6	9.1	schizophrenia (ICD-10)	2
Hwang 2003[176]	Risperidone 6.88 (4-8)	25	6	13.4	schizophrenia (DSM-IV)	2
	Amisulpride 630 (400-800)	23	6	13.3	schizophrenia (DSM-IV)	2
Hwang 2012[177]	Aripiprazole 15 (15-15)	49	4	n.i.	schizophrenia or schizoaffective disorder (DSM-IV)	2
	Risperidone 6 (6-6)	34	4	n.i.	schizophrenia or schizoaffective disorder (DSM-IV)	2
Imai 1980[178]	Perphenazine 18 (12-24)	49	8	n.i.	schizophrenia (clinical Diagnosis)	2
	Zotepine 112.5 (75-150)	46	8	n.i.	schizophrenia (clinical Diagnosis)	2
Ingole 2009[179]	Olanzapine 10 (10-10)	30	12	n.i.	schizophrenia (DSM-IV)	0
	Risperidone 6 (6-6)	30	12	n.i.	schizophrenia (DSM-IV)	0
Ishigooka 2001[180]	Olanzapine 10.31 (5-15)	93	8	15.8	schizophrenia (ICD-10)	2
	Haloperidol 7.36 (4-12)	89	8	17.5	schizophrenia (ICD-10)	2
Ishigooka 2018[181]	Placebo	116	6	17.4	schizophrenia (DSM-IV-TR)	2
	Brexpiprazole 3 (2-4)	228	6	15.69035088	schizophrenia (DSM-IV-TR)	2
Itil 1971[182]	Trifluoperazine 85 (10-160)	30	12	n.i.	schizophrenia (clinical Diagnosis)	2

Study name	Antipsychotics and daily dose in mg, on flexible dosage mean value (range)	N	Duration in weeks	Mean duration of illness in years	Diagnosis	Blinding
	Molindone 85 (10-160)	30	12	n.i.	schizophrenia (clinical Diagnosis)	2
Itoh 1976[183]	Penfluridol 60 (20-100)	54	12	n.i.	schizophrenia (clinical diagnosis)	2
	Perphenazine 34 (8-60)	51	12	n.i.	schizophrenia (clinical diagnosis)	2
Itoh 1977[184]	Clozapine 287.5 (75-500)	47	12	n.i.	schizophrenia (clinical Diagnosis)	2
	Haloperidol 8.625 (2.25-15)	41	12	n.i.	schizophrenia (clinical Diagnosis)	2
Janicak 2001[185]	Haloperidol 10.8 (4-20)	32	6	18.9	schizoaffective disorder (DSM-IV)	2
	Risperidone 5.5 (2-10)	30	6	20	schizoaffective disorder (DSM-IV)	2
Jann 1997[186]	Placebo	18	6	n.i.	schizophrenia (DSM-III-R)	2
	Haloperidol 40 (15-75)	18	6	n.i.	schizophrenia (DSM-III-R)	2
Janssen CR012625[187]	Paliperidone 6 (6-6)	136	6	17.92	schizophrenia with acute symptoms (DSM-IV)	2
	Placebo	138	6	17.92	schizophrenia with acute symptoms (DSM-IV)	2
	Olanzapine 10 (10-10)	47	6	17.92	schizophrenia with acute symptoms (DSM-IV)	2
Jindal 2013[188]	Aripiprazole 12.5 (10-20)	30	6	n.i.	schizophrenia (ICD-10)	2
	Olanzapine 11.01 (10-20)	30	6	n.i.	schizophrenia (ICD-10)	2

Study name	Antipsychotics and daily dose in mg, on flexible dosage mean value (range)	N	Duration in weeks	Mean duration of illness in years	Diagnosis	Blinding
Johnson NCT00397033[189]	Paliperidone 8.55 (3-12)	209	6	11.46	acute exacerbation of schizoaffective disorder (DSM-IV)	2
	Placebo	107	6	10.5	acute exacerbation of schizoaffective disorder (DSM-IV)	2
Johnson NCT00412373[190]	Placebo	95	6	13.3	schizoaffective disorder (DSM-IV)	2
	Paliperidone 8.6 (3-12)	216	6	13.3	schizoaffective disorder (DSM-IV)	2
Johnson NCT00524043[191]	Paliperidone 6 (6-6)	70	6	15.3	acute exacerbation of schizophrenia (DSM-IV)	2
	Placebo	65	6	11.9	acute exacerbation of schizophrenia (DSM-IV)	2
Johnstone 1978, 01073[192]	α-Flupenthixol 8.357 (6-9)	15	4	n.i.	schizophrenic patients (PSE)	2
	Placebo	15	4	n.i.	schizophrenic patients (PSE)	2
Judd 1973[193]	Thioridazine 400 (400-400)	12	3	n.i.	newly admitted schizophrenics (clinical diagnosis)	2
	Placebo	12	3	n.i.	newly admitted schizophrenics (clinical diagnosis)	2
Kahn 2007[194]	Placebo	118	6	8	acute schizophrenia (DSM-IV)	2
	Quetiapine 551 (400-800)	470	6	7.82	acute schizophrenia (DSM-IV)	2
Kane 2002[195]	Aripiprazole 22.5 (15-30)	204	4	16.6	schizophrenia or schizoaffective disorder, acute relapse (DSM-IV)	2

Study name	Antipsychotics and daily dose in mg, on flexible dosage mean value (range)	N	Duration in weeks	Mean duration of illness in years	Diagnosis	Blinding
	Haloperidol 10 (10-10)	104	4	16	schizophrenia or schizoaffective disorder, acute relapse (DSM-IV)	2
	Placebo	106	4	16	schizophrenia or schizoaffective disorder, acute relapse (DSM-IV)	2
Kane 2007b[196]	Olanzapine 10 (10-10)	128	6	9.8	acute episode of schizophrenia (DSM-IV)	2
	Paliperidone 9.1 (6-12)	375	6	10.31	acute episode of schizophrenia (DSM-IV)	2
	Placebo	127	6	9.9	acute episode of schizophrenia (DSM-IV)	2
Kane 2009 8 weeks[197]	Olanzapine 16.7 (10-20)	281	8	16.5	schizophrenia (DSM-IV-TR)	2
	Aripiprazole 20.93 (10-30)	285	8	16.3	schizophrenia (DSM-IV-TR)	2
Kane 2010a[198]	Haloperidol 8 (8-8)	115	6	12.5	schizophrenia acute exacerbation (DSM-IV-TR)	2
	Placebo	123	6	12.5	schizophrenia acute exacerbation (DSM-IV-TR)	2
	Asenapine 14.9 (10-20)	220	6	12.5	schizophrenia acute exacerbation (DSM-IV-TR)	2
Kane 2015[199]	Placebo	184	6	13.7	schizophrenia (DSM-IV-TR)	2
	Brexpiprazole 2.99459459459459 (2-4)	370	6	#NV	schizophrenia (DSM-IV-TR)	2
Kaushal 2012[200]	Olanzapine 5 (5-5)	30	8	n.i.	schizophrenia, schzopreniform disorder or schizoaffective	0

Study name	Antipsychotics and daily dose in mg, on flexible dosage mean value (range)	N	Duration in weeks	Mean duration of illness in years	Diagnosis	Blinding
					disorder (ICD-10)	
	Risperidone 2 (2-2)	30	8	n.i.	schizophrenia, schzopreniform disorder or schizoaffective disorder (ICD-10)	0
Keck 1998[201]	Ziprasidone 120 (120-120)	47	4	14.6	acute exacerbation of subchronic or chronic schizophrenia or schizoaffective disorder (DSM-III-R)	2
	Placebo	48	4	17.3	acute exacerbation of subchronic or chronic schizophrenia or schizoaffective disorder (DSM-III-R)	2
Kenway 1971[202]	Fluphenazine 4.9 (2.5-15)	15	4	6.9	schizophrenia (clinical Diagnosis)	2
	Pimozide 2.9 (1.5-9)	15	4	6.9	schizophrenia (clinical Diagnosis)	2
Khorana 1988[203]	Penfluridol 20 (n.i.-n.i.)	15	12	n.i.	chronic schizophrenic patients (clinical Diagnosis)	2
	Trifluoperazine 10 (10-10)	17	12	n.i.	chronic schizophrenic patients (clinical Diagnosis)	2
Kiloh 1976acute[204]	Loxapine 43.08 (n.i.-n.i.)	30	12	n.i.	schizophrenia (clinical Diagnosis)	2
	Trifluoperazine 27.4 (n.i.-n.i.)	27	12	n.i.	schizophrenia (clinical Diagnosis)	2
King 1959[205]	Placebo	24	10	n.i.	schizophrenia (clinical diagnosis)	2
	Chlorpromazine 400 (400-400)	24	10	n.i.	schizophrenia (clinical diagnosis)	2

Study name	Antipsychotics and daily dose in mg, on flexible dosage mean value (range)	N	Duration in weeks	Mean duration of illness in years	Diagnosis	Blinding
Kingstone 1970[206]	Chlorpromazine 435 (150-1800)	21	3	3.1	acute psychotic patients (clinical Diagnosis)	2
	Clopenthixol 122 (75-600)	20	3	2.4	acute psychotic patients (clinical Diagnosis)	2
Kinon 2011[207]	Olanzapine 15 (15-15)	62	4	15	schizophrenia (DSM-IV)	2
	Placebo	122	4	12.5	schizophrenia (DSM-IV)	2
Klein 1973[208]	Placebo	42	6	n.i.	schizophrenia (clinical diagnosis)	2
	Chlorpromazine 750 (300-1200)	46	6	n.i.	schizophrenia (clinical diagnosis)	2
Klein 1985[209]	Amisulpride 473 (n.i.-n.i.)	9	4	6.18	schizophrenia, acute or chronic (ICD-9)	2
	Haloperidol n.i. (n.i.-n.i.)	10	4	6.18	schizophrenia, acute or chronic (ICD-9)	2
Klieser 1989[210]	Placebo	16	3	11.7	acute schizophrenia (DSM-III)	2
	Haloperidol 20 (20-20)	22	3	11.7	acute schizophrenia (DSM-III)	2
Klieser 1990[211]	Haloperidol 20 (20-20)	16	6	17.2	Chronic schizophrenia (clinical Diagnosis)	2
	Clozapine 400 (400-400)	16	6	17.2	Chronic schizophrenia (clinical Diagnosis)	2
Klieser 1991[212]	Zotepine 225 (225-225)	14	4	n.i.	acute schizophrenia, acute schizophrenic episode, schizodominant schizoaffective psychosis (ICD-9)	2

Study name	Antipsychotics and daily dose in mg, on flexible dosage mean value (range)	N	Duration in weeks	Mean duration of illness in years	Diagnosis	Blinding
	Haloperidol 12 (12-12)	12	4	n.i.	acute schizophrenia, acute schizophrenic episode, schizodominant schizoaffective psychosis (ICD-9)	2
Klieser 1994[213]	Clozapine 350 (n.i.-n.i.)	17	4	4.9	acute schizophrenia, paranoid type (ICD-9)	2
	Haloperidol 16 (16-16)	17	4	5.1	acute schizophrenia, paranoid type (ICD-9)	2
Klimke 1993[214]	Haloperidol 15 (15-15)	25	3	6.7	schizophrenics (ICD-9)	2
	Perazine 300 (300-300)	25	3	6.7	schizophrenics (ICD-9)	2
Kluge 2007[215]	Clozapine 266.7 (100-400)	15	6	6.7	schizophrenia, schizophreniform or schizoaffective disorder (DSM-IV)	2
	Olanzapine 21.2 (5-25)	15	6	4.8	schizophrenia, schizophreniform or schizoaffective disorder (DSM-IV)	2
Knegtering 2004[216]	Quetiapine 580 (200-1200)	25	6	n.i.	schizophrenia or a related psychosis such as schizophreni- form, schizoaffective or brief psychotic episode (DSM-IV)	0
	Risperidone 3.2 (1-6)	26	6	n.i.	schizophrenia or a related psychosis such as schizophreni- form, schizoaffective or brief psychotic episode (DSM-IV)	0
Knegtering 2006[217]	Olanzapine 9.4 (5-15)	25	6	n.i.	patients with schizophrenia (DSM-IV)	0

Study name	Antipsychotics and daily dose in mg, on flexible dosage mean value (range)	N	Duration in weeks	Mean duration of illness in years	Diagnosis	Blinding
	Risperidone 3.4 (2-6)	21	6	n.i.	patients with schizophrenia (DSM-IV)	0
Kordas 1968[218]	Chlorpromazine 300 (300-300)	18	4	n.i.	chronic schizophrenic (clinical Diagnosis)	2
	Clopenthixol 150 (150-150)	18	4	n.i.	chronic schizophrenic (clinical Diagnosis)	2
	Placebo	18	4	n.i.	chronic schizophrenic (clinical Diagnosis)	2
Kramer 1978[219]	Loxapine 78 (40-200)	29	4	n.i.	schizophrenia (DSM-II)	2
	Thioridazine 526 (100-1000)	27	4	n.i.	schizophrenia (DSM-II)	2
Kurland 1961[220]	Placebo	37	6	n.i.	predominantly schizophrenia (clinical diagnosis)	2
	Chlorpromazine 401.35 (300-1200)	33	6	n.i.	predominantly schizophrenia (clinical diagnosis)	2
	Perphenazine 30.83 (24-96)	36	6	n.i.	predominantly schizophrenia (clinical diagnosis)	2
Lahti 2009[221]	Haloperidol 10.4 (5-20)	14	6	15.3	schizophrenia (DSM-IV)	2
	Olanzapine 15.9 (12.5-25)	18	6	11.3	schizophrenia (DSM-IV)	2
Lamure 2003[222]	Haloperidol n.i. (n.i.-n.i.)	45	12.86	14.45	schizophrenia (DSM-III-R)	0
	Zuclopenthixol n.i. (n.i.-n.i.)	43	12.86	11.7	schizophrenia (DSM-III-R)	0
Lemmer 1993[223]	Haloperidol n.i. (n.i.-n.i.)	n.i.	4	n.i.	acute schizophrenic patients (n.i.)	2
	Placebo	n.i.	4	n.i.	acute schizophrenic patients (n.i.)	2

Study name	Antipsychotics and daily dose in mg, on flexible dosage mean value (range)	N	Duration in weeks	Mean duration of illness in years	Diagnosis	Blinding
Lemperiere 1985[224]	Chlorpromazine 330 (n.i.-n.i.)	32	6	n.i.	schizophrenia (Feighner Criteria)	0
	Haloperidol 18 (n.i.-n.i.)	33	6	n.i.	schizophrenia (Feighner Criteria)	0
Leon 1974[225]	Clozapine 600 (100-1600)	25	6	n.i.	schizophrenia (DSM-II)	2
	Chlorpromazine 600 (100-1600)	25	6	n.i.	schizophrenia (DSM-II)	2
Levita 1961[226]	Placebo	9	13	n.i.	schizophrenia (clinical diagnosis)	2
	Chlorpromazine 225 (50-400)	13	13	n.i.	schizophrenia (clinical diagnosis)	2
Li 2012[227]	Quetiapine 630.47 (600-750)	60	6	8.5	schizophrenia (DSM-IV)	1
	Risperidone 3.57 (3-4)	59	6	8	schizophrenia (DSM-IV)	1
Lieberman 2005 12weeks[228]	Risperidone 3.9 (1.5-6)	341	12	n.i.	schizophrenia (DSM-IV)	2
	Ziprasidone 112.8 (40-160)	185	12	n.i.	schizophrenia (DSM-IV)	2
	Quetiapine 543.4 (200-800)	337	12	n.i.	schizophrenia (DSM-IV)	2
	Perphenazine 20.8 (8-32)	261	12	n.i.	schizophrenia (DSM-IV)	2
	Olanzapine 20.1 (7.5-30)	336	12	n.i.	schizophrenia (DSM-IV)	2
Liebermann 2015[229]	Placebo	85	4	16.7	schizophrenia (DSM-IV)	2
	Risperidone 4 (4-4)	82	4	15.2	schizophrenia (DSM-IV)	2

Study name	Antipsychotics and daily dose in mg, on flexible dosage mean value (range)	N	Duration in weeks	Mean duration of illness in years	Diagnosis	Blinding
Liemburg 2011[230]	Aripiprazole 12.6 (5-30)	20	6	n.i.	schizophrenia (DSM-IV)	0
	Risperidone 3.2 (1-5)	20	6	n.i.	schizophrenia (DSM-IV)	0
Lin 2003[231]	Clozapine 377.1 (n.i.-n.i.)	24	12	n.i.	schizophrenia (DSM-IV)	1
	Zotepine 397.1 (n.i.-n.i.)	35	12	n.i.	schizophrenia (DSM-IV)	1
Lindenmayer 2008[232]	Quetiapine 667 (600-800)	267	6	15.45	schizophrenia, acute exacerbation (DSM-IV)	2
	Placebo	84	6	14.7	schizophrenia, acute exacerbation (DSM-IV)	2
Litman 2016[233]	Placebo 0 (n.i.-n.i.)	55	4	n.i.	schizophrenia (DSM-IV)	2
	Risperidone 4 (4-4)	31	4	n.i.	schizophrenia (DSM-IV)	2
Litmann 2014[234]	Placebo	41	4	n.i.	schizophrenia, symptomatic patients but medically stable (DSM-IV)	2
	Olanzapine 15 (15-15)	22	4	n.i.	schizophrenia, symptomatic patients but medically stable (DSM-IV)	2
Little 1958[235]	Chlorpromazine 150 (150-150)	n.i.	3	n.i.	schizophrenia, paraphrenia, depression, organic causes, neurosis (clinical diagnosis)	2
	Placebo	n.i.	3	n.i.	schizophrenia, paraphrenia, depression, organic causes, neurosis (clinical diagnosis)	2
Liu 2000[236]	Haloperidol n.i. (n.i.-n.i.)	28	12	8.4	schizophrenic disorders (DSM-III-R)	2

Study name	Antipsychotics and daily dose in mg, on flexible dosage mean value (range)	N	Duration in weeks	Mean duration of illness in years	Diagnosis	Blinding
	Risperidone n.i. (n.i.-n.i.)	28	12	7.3	schizophrenic disorders (DSM-III-R)	2
Loebel 2015a[237]	Lurasidone 97.37 (80-160)	199	6	14.1	acute exacerbation of schizophrenia (DSM-IV-TR)	2
	Placebo	112	6	14.2	acute exacerbation of schizophrenia (DSM-IV-TR)	2
Loza 1999[238]	Chlorpromazine 465 (200-800)	14	6	11.1	schizophrenia (DSM-IV)	0
	Olanzapine 12.5 (5-20)	27	6	11.1	schizophrenia (DSM-IV)	0
Loza 2006[239]	Olanzapine 12.5 (5-20)	39	8	n.i.	schizophrenia (clinical Diagnosis)	2
	Risperidone 5 (2-8)	40	8	n.i.	schizophrenia (clinical Diagnosis)	2
Lublin 1991[240]	Haloperidol 6.2 (2.5-18)	7	3	#NV	schizophrenia (clinical Diagnosis)	1
	Zuclopenthixol 16.5 (6-50)	8	3	24.1	schizophrenia (clinical Diagnosis)	1
Luckey 1967[241]	Haloperidol 10 (5-15)	13	12	n.i.	chronic schizophrenia (clinical Diagnosis)	2
	Trifluoperazine 20 (10-30)	13	12	n.i.	chronic schizophrenia (clinical Diagnosis)	2
Lundbeck NCT00864045[242]	Sertindole 16 (12-20)	198	12	n.i.	Chronic schizophrenia (DSM-IV-TR)	2
	Olanzapine 15 (10-20)	196	12	n.i.	Chronic schizophrenia (DSM-IV-TR)	2
Maat 2014[243]	Aripiprazole 17 (7.5-30)	38	8	n.i.	schizophrenia (DSM-IV-TR)	0

Study name	Antipsychotics and daily dose in mg, on flexible dosage mean value (range)	N	Duration in weeks	Mean duration of illness in years	Diagnosis	Blinding
	Risperidone 3.55 (1-6)	42	8	n.i.	schizophrenia (DSM-IV-TR)	0
Mahadevan 1991[244]	Sulpiride 700 (200-1200)	31	10	n.i.	schizophrenia (Research Diagnostic Criteria)	1
	Zuclopenthixol 87.5 (25-150)	30	10	n.i.	schizophrenia (Research Diagnostic Criteria)	1
Mahal 1976[245]	Chlorpromazine 250 (200-300)	27	9	0.49	schizophrenia (clinical diagnosis)	2
	Placebo	27	9	0.49	schizophrenia (clinical diagnosis)	2
Marder 1994[246]	Risperidone 6 (6-6)	64	8	15.8	schizophrenia (DSM-III-R)	2
	Placebo	66	8	15.2	schizophrenia (DSM-III-R)	2
	Haloperidol 20 (20-20)	66	8	15.4	schizophrenia (DSM-III-R)	2
Marder 2007c[247]	Paliperidone 9 (6-12)	224	6	16.4	acute exacerbation of schizophrenia (DSM-IV)	2
	Olanzapine 10 (10-10)	110	6	15.95	acute exacerbation of schizophrenia (DSM-IV)	2
	Placebo	110	6	16	acute exacerbation of schizophrenia (DSM-IV)	2
Martin 2002[248]	Olanzapine 12 (5-20)	188	8	8.12	schizophrenia or schizophreniform disorder (DSM-IV)	2
	Amisulpride 489 (200-800)	189	8	9.56	schizophrenia or schizophreniform disorder (DSM-IV)	2

Study name	Antipsychotics and daily dose in mg, on flexible dosage mean value (range)	N	Duration in weeks	Mean duration of illness in years	Diagnosis	Blinding
McCue2006[249]	Aripiprazole 21.8 (n.i.-n.i.)	63	3	14.9	schizophrenia, schizoaffective disorder or schizophreniform disorder (DSM-IV)	0
	Ziprasidone 151.2 (n.i.-n.i.)	59	3	12.9	schizophrenia, schizoaffective disorder or schizophreniform disorder (DSM-IV)	0
	Olanzapine 19.1 (n.i.-n.i.)	58	3	11.7	schizophrenia, schizoaffective disorder or schizophreniform disorder (DSM-IV)	0
	Quetiapine 652.5 (n.i.-n.i.)	62	3	14.5	schizophrenia, schizoaffective disorder or schizophreniform disorder (DSM-IV)	0
	Haloperidol 16 (n.i.-n.i.)	61	3	12.2	schizophrenia, schizoaffective disorder or schizophreniform disorder (DSM-IV)	0
	Risperidone 5.2 (n.i.-n.i.)	65	3	13.1	schizophrenia, schizoaffective disorder or schizophreniform disorder (DSM-IV)	0
McEvoy 2007b[250]	Aripiprazole 17.42718447 (15-20)	206	6	16.49	acute exacerbation of schizophrenia (DSM-IV)	2
	Placebo	108	6	16.7	acute exacerbation of schizophrenia (DSM-IV)	2
McInnes 1978[251]	Placebo	9	13	n.i.	chronic schizophrenia (clinical diagnosis)	2
	Pimozide n.i. (n.i.-n.i.)	9	13	n.i.	chronic schizophrenia (clinical diagnosis)	2
McQuade 2004_6weeks[252]	Olanzapine 16.5 (10-20)	161	6	14.05	schizophrenia, acute relapse (DSM-IV)	2

Study name	Antipsychotics and daily dose in mg, on flexible dosage mean value (range)	N	Duration in weeks	Mean duration of illness in years	Diagnosis	Blinding
	Aripiprazole 25.1 (15-30)	156	6	13.74	schizophrenia, acute relapse (DSM-IV)	2
Meltzer 2004[253]	Placebo	98	6	n.i.	acute schizophrenia or schizoaffective disorder (DSM-IV)	2
	Haloperidol 10 (10-10)	98	6	n.i.	acute schizophrenia or schizoaffective disorder (DSM-IV)	2
Meltzer 2007a[254]	Placebo	149	6	n.i.	acute exacerbation of schizophrenia (DSM-IV-TR)	2
	Risperidone 6 (6-6)	154	6	n.i.	acute exacerbation of schizophrenia (DSM-IV-TR)	2
Menon 1972[255]	Placebo	20	10	n.i.	chronic schizophrenia (clinical diagnosis)	1
	Trifluoperazine 15 (15-15)	20	10	n.i.	chronic schizophrenia (clinical diagnosis)	1
Mesotten 1991[256]	Haloperidol 11 (2-20)	32	8	n.i.	schizophrenia, other serious psychotic disorders (DSM-III)	2
	Risperidone 11 (2-20)	28	8	n.i.	schizophrenia, other serious psychotic disorders (DSM-III)	2
Meyer-Lindenberg 1997[257]	Zotepine 300 (150-450)	25	6	7.55	schizophrenia (DSM-III-R)	2
	Clozapine 300 (150-450)	25	6	7.55	schizophrenia (DSM-III-R)	2
Mezquita 1972[258]	Placebo 0 (n.i.-n.i.)	43	12	n.i.	schizophrenia (clinical Diagnosis)	0
	Sulpiride 1100 (800-1400)	46	12	n.i.	schizophrenia (clinical Diagnosis)	0
Min 1993[259]	Haloperidol 8.9 (8.9-8.9)	19	8	10.6	chronic schizophrenic disorder (DSM-III-R)	2

Study name	Antipsychotics and daily dose in mg, on flexible dosage mean value (range)	N	Duration in weeks	Mean duration of illness in years	Diagnosis	Blinding
	Risperidone 7.5 (7.5-7.5)	16	8	10.6	chronic schizophrenic disorder (DSM-III-R)	2
Mirabzadeh 2014[260]	Risperidone 6 (6-6)	37	8	n.i.	schizophrenia (DSM-IV-TR)	0
	Haloperidol 15 (15-15)	29	8	n.i.	schizophrenia (DSM-IV-TR)	0
Möller 1997[261]	Amisulpride 700 (600-800)	95	6	10.1	Chronic or subchronic schizophrenia (DSM-III-R)	2
	Haloperidol 17.5 (15-20)	96	6	9	Chronic or subchronic schizophrenia (DSM-III-R)	2
Montgomery 1992[262]	Placebo	33	4	n.i.	schizophrenia, acute exacerbation (DSM-III)	2
	Thioridazine 400 (400-400)	32	4	n.i.	schizophrenia, acute exacerbation (DSM-III)	2
Moore 1975[263]	Chlorpromazine 430 (200-1200)	29	6	n.i.	schizophrenia (clinical Diagnosis)	2
	Loxapine 36 (20-120)	29	6	n.i.	schizophrenia (clinical Diagnosis)	2
Moosavi 2015[264]	Quetiapine 498 (100-800)	45	4	n.i.	schizophrenia or schizophreniform disorder (DSM-IV-R)	2
	Risperidone 5.6 (2-8)	45	4	n.i.	schizophrenia or schizophreniform disorder (DSM-IV-R)	2
Mori 2004[265]	Risperidone 7.37 (1-12)	19	8	34.51	schizophrenia (DSM-IV)	2
	Olanzapine 16.5 (2.5-20)	20	8	34.51	schizophrenia (DSM-IV)	2

Study name	Antipsychotics and daily dose in mg, on flexible dosage mean value (range)	N	Duration in weeks	Mean duration of illness in years	Diagnosis	Blinding
	Quetiapine 432.5 (50-750)	20	8	34.51	schizophrenia (DSM-IV)	2
Morris 1970[266]	Pimozide 9 (3-9)	15	8	19.2	schizophrenia (clinical Diagnosis)	2
	Fluphenazine 15 (5-15)	15	8	19.2	schizophrenia (clinical Diagnosis)	2
Moyano 1975[267]	Loxapine 50 (20-80)	25	12	n.i.	schizophrenia (clinical Diagnosis)	2
	Trifluoperazine 30 (20-40)	24	12	n.i.	schizophrenia (clinical Diagnosis)	2
Murasaki 1993[268]	Risperidone 6.1 (2-12)	97	8	n.i.	schizophrenia (ICD-9, DSM-III-R)	2
	Haloperidol 5.8 (2-12)	95	8	n.i.	schizophrenia (ICD-9, DSM-III-R)	2
Murasaki 2001[269]	Haloperidol 6.7 (4.5-18)	97	8	16.9	schizophrenia (ICD-10)	2
	Quetiapine 226 (150-600)	100	8	17.5	schizophrenia (ICD-10)	2
Nagesh 2016[270]	Asenapine n.i. (10-20)	30	6	n.i.	schizophrenic disorder (ICD-10)	0
	Iloperidone n.i. (12-24)	30	6	n.i.	schizophrenic disorder (ICD-10)	0
Nam 2004[271]	Haloperidol 17 (n.i.-n.i.)	20	12	12.55	schizoprehnia (DSM-IV)	0
	Risperidone 5 (n.i.-n.i.)	20	12	14.55	schizoprehnia (DSM-IV)	0
	Clozapine 290 (n.i.-n.i.)	20	12	17.7	schizoprehnia (DSM-IV)	0
NCT00350467[272]	Olanzapine 10 (5-15)	145	6	n.i.	schizophrenia (DSM-IV)	2
	Paliperidone 7.5 (3-12)	141	6	n.i.	schizophrenia (DSM-IV)	2

**Study name**	**Antipsychotics and daily dose in mg, on flexible dosage mean value (range)**	**N**	**Duration in weeks**	**Mean duration of illness in years**	**Diagnosis**	**Blinding**
NCT00563706[273]	Risperidone 4 (4-4)	43	4	n.i.	schizophrenia (DSM-IV-TR)	2
	Placebo 0 (n.i.-n.i.)	37	4	n.i.	schizophrenia (DSM-IV-TR)	2
NCT00882518[274]	Chlorpromazine 450 (300-600)	192	6	n.i.	schizophrenia (clinical Diagnosis)	2
	Quetiapine 600 (400-800)	196	6	n.i.	schizophrenia (clinical Diagnosis)	2
NCT00905307[275]	Aripiprazole 15 (15-15)	50	6	n.i.	schizophrenia (DSM-IV-TR)	2
	Brexpiprazole 2.5 (2.5-2.5)	90	6	n.i.	schizophrenia (DSM-IV-TR)	2
	Placebo	95	6	n.i.	schizophrenia (DSM-IV-TR)	2
NCT01098110[276]	Placebo	174	6	n.i.	schizophrenia (DSM-IV-TR)	2
	Asenapine 15.0837988826816 (20-10)	358	6	#NV	schizophrenia (DSM-IV-TR)	2
NCT01104766[277]	Placebo	153	6	12.5	acute exacerbation of schizophrenia (DSM-IV-TR)	2
	Aripiprazole 10 (10-10)	152	6	12.4	acute exacerbation of schizophrenia (DSM-IV-TR)	2
	Cariprazine 4.50961538461539 (3-6)	312	6	#NV	acute exacerbation of schizophrenia (DSM-IV-TR)	2
NCT01617187[278]	Asenapine 10 (10-10)	113	6	n.i.	schizophrenia of paranoid, disorganized, or undifferentiated subtype (DSM-IV-TR)	2

Study name	Antipsychotics and daily dose in mg, on flexible dosage mean value (range)	N	Duration in weeks	Mean duration of illness in years	Diagnosis	Blinding
	Olanzapine 15 (15-15)	46	6	n.i.	schizophrenia of paranoid, disorganized, or undifferentiated subtype (DSM-IV-TR)	2
	Placebo 0 (n.i.-n.i.)	103	6	n.i.	schizophrenia of paranoid, disorganized, or undifferentiated subtype (DSM-IV-TR)	2
NCT01810380[279]	Brexpiprazole 3 (2-4)	150	6	12.88	DSM-IV-TR (DSM-IV-TR)	2
	Quetiapine 600 (400-800)	154	6	13.78	DSM-IV-TR (DSM-IV-TR)	2
	Placebo 0 (n.i.-n.i.)	163	6	14.15	DSM-IV-TR (DSM-IV-TR)	2
Nishizono 1994[280]	Zotepine 225 (0-450)	60	4	n.i.	schizophrenia (n.i.)	2
	Haloperidol 10.5 (0-21)	57	4	n.i.	schizophrenia (n.i.)	2
	Chlorpromazine 225 (0-450)	52	4	n.i.	schizophrenia (n.i.)	2
Nistico 1974,[281]	Placebo	20	6	n.i.	hospitalized chronic schizophrenics (clinical diagnosis)	2
	Penfluridol 40 (30-50)	20	6	n.i.	hospitalized chronic schizophrenics (clinical diagnosis)	2
O Brien 1974[282]	Haloperidol 11.5 (3-20)	15	3	n.i.	schizophrenia (clinical Diagnosis)	2
	Trifluoperazine 28.5 (9-48)	15	3	n.i.	schizophrenia (clinical Diagnosis)	2
Ortega-Soto 1997[283]	Olanzapine 17.5 (n.i.-n.i.)	n.i.	8	n.i.	schizophrenia (DSM-IV)	2

Study name	Antipsychotics and daily dose in mg, on flexible dosage mean value (range)	N	Duration in weeks	Mean duration of illness in years	Diagnosis	Blinding
	Risperidone 7.5 (n.i.-n.i.)	n.i.	8	n.i.	schizophrenia (DSM-IV)	2
Ozguven 2004[284]	Olanzapine 23 (23-23)	15	6	n.i.	schizophrenia, Schizoaffective Disorder, Schizophreniform Disorder (DSM-IV)	1
	Quetiapine 826.67 (826.67-826.67)	19	6	n.i.	schizophrenia, Schizoaffective Disorder, Schizophreniform Disorder (DSM-IV)	1
Paprocki 1976[285]	Haloperidol n.i. (n.i.-n.i.)	25	12.86	n.i.	schizophrenia (clinical Diagnosis)	2
	Loxapine n.i. (n.i.-n.i.)	25	12.86	n.i.	schizophrenia (clinical Diagnosis)	2
Park 2013[286]	Olanzapine 11.6 (5-20)	10	12	n.i.	schizophrenia or schizoaffective disorder (DSM-IV korean version)	0
	Ziprasidone 109 (20-160)	10	12	n.i.	schizophrenia or schizoaffective disorder (DSM-IV korean version)	0
Pathiraja 1995[287]	Placebo	7	n.i.	n.i.	schizophrenia (DSM-III-R)	2
	Risperidone 9 (2-16)	18	n.i.	n.i.	schizophrenia (DSM-III-R)	2
	Haloperidol 12.5 (5-20)	10	n.i.	n.i.	schizophrenia (DSM-III-R)	2
Patil 2007[288]	Olanzapine 15 (15-15)	34	4	n.i.	schizophrenia (DSM-IV-TR)	2
	Placebo	63	4	n.i.	schizophrenia (DSM-IV-TR)	2
Payne 1960[289]	Chlorpromazine 237.5 (75-300)	7	6	n.i.	chronic schizophrenia (clinical diagnosis)	2

Study name	Antipsychotics and daily dose in mg, on flexible dosage mean value (range)	N	Duration in weeks	Mean duration of illness in years	Diagnosis	Blinding
	Placebo	7	6	n.i.	chronic schizophrenia (clinical diagnosis)	2
Petit 1996[290]	Haloperidol 15.7 (10-20)	63	8	10.558	acute exacerbation of schizophrenia (DSM-III-R)	2
	Zotepine 241.5 (150-300)	63	8	12.725	acute exacerbation of schizophrenia (DSM-III-R)	2
Peuskens 1995[291]	Risperidone 6.01 (4-8)	457	8	16.85	chronic schizophrenia (DSM-III-R)	2
	Haloperidol 10 (10-10)	226	8	16.1	chronic schizophrenia (DSM-III-R)	2
Peuskens 1997[292]	Quetiapine 407 (75-750)	101	6	n.i.	acute exacerbation of chronic/subchronic schizophrenia, or schizophreniform disorder (DSM-III-R)	2
	Chlorpromazine 384 (100-750)	100	6	n.i.	acute exacerbation of chronic/subchronic schizophrenia, or schizophreniform disorder (DSM-III-R)	2
Peuskens 1999[293]	Risperidone 8 (8-8)	113	8	10.2	acute exacerbation of schizophrenia (DSM-IV)	2
	Amisulpride 800 (800-800)	115	8	7.9	acute exacerbation of schizophrenia (DSM-IV)	2
Pfizer 2008[294]	Placebo	n.i.	3	n.i.	schizophrenia acute exacerbation (DSM-IV)	2
	Aripiprazole 15 (15-15)	n.i.	3	n.i.	schizophrenia acute exacerbation (DSM-IV)	2

Study name	Antipsychotics and daily dose in mg, on flexible dosage mean value (range)	N	Duration in weeks	Mean duration of illness in years	Diagnosis	Blinding
Pi 1990, 01400[295]	Thioridazine 500 (200-800)	7	4	8.41	chronic schizophrenics with acute exacerbation (DSM-III)	2
	Placebo	5	4	8.41	chronic schizophrenics with acute exacerbation (DSM-III)	2
Pichot 1983[296]	Chlorpromazine 256 (200-300)	28	3	n.i.	schizophrenia (clinical Diagnosis)	2
	Sulpiride 1050 (800-1200)	33	3	n.i.	schizophrenia (clinical Diagnosis)	2
Potkin 1994[297]	Clozapine 460 (25-400)	n.i.	5	n.i.	schizophrenia (DSM-III-R)	2
	Placebo 0 (n.i.-n.i.)	n.i.	5	n.i.	schizophrenia (DSM-III-R)	2
Potkin 2003[298]	Aripiprazole 25 (20-30)	202	4	n.i.	schizophrenia or schizoaffective disorder (DSM-IV)	2
	Placebo	103	4	n.i.	schizophrenia or schizoaffective disorder (DSM-IV)	2
	Risperidone 6 (6-6)	99	4	n.i.	schizophrenia or schizoaffective disorder (DSM-IV)	2
Potkin 2007c[299]	Risperidone 6 (6-6)	60	6	n.i.	acute exacerbation of schizophrenia (DSM-IV)	2
	Placebo	62	6	n.i.	acute exacerbation of schizophrenia (DSM-IV)	2
	Asenapine 10 (10-10)	60	6	n.i.	acute exacerbation of schizophrenia (DSM-IV)	2
Potter 1989[300]	Clozapine 325 (50-600)	17	8	5.8	schizophrenia (DSM-III)	2

Study name	Antipsychotics and daily dose in mg, on flexible dosage mean value (range)	N	Duration in weeks	Mean duration of illness in years	Diagnosis	Blinding
	Chlorpromazine 350 (100-600)	20	8	5	schizophrenia (DSM-III)	2
Protocol 128-301[301]	Ziprasidone 120 (40-200)	243	12	n.i.	schizophrenia or schizoaffective disorder (DSM-III-R)	2
Protocol 128-301[301]	Haloperidol 15 (10-20)	238	12	n.i.	schizophrenia or schizoaffective disorder (DSM-III-R)	2
Protocol ZIP-NY-97-019[302]	Olanzapine 12.4 (5-20)	34	12	n.i.	schizophrenia or schizoaffective disorder (DSM-IV)	2
Protocol ZIP-NY-97-019[302]	Ziprasidone 105.7 (80-320)	33	12	n.i.	schizophrenia or schizoaffective disorder (DSM-IV)	2
Puech 1998[303]	Amisulpride 600 (400-800)	129	4	9.65	chronic or subchronic schizophrenia with acute exacerbation (DSM-III-R)	2
Puech 1998[303]	Haloperidol 16 (16-16)	64	4	10.7	chronic or subchronic schizophrenia with acute exacerbation (DSM-III-R)	2
Ramsay 1970[304]	Molindone 120 (120-120)	10	12	17.5	chronic schizophrenia (clinical Diagnosis)	2
Ramsay 1970[304]	Trifluoperazine 60 (60-60)	10	12	17.5	chronic schizophrenia (clinical Diagnosis)	2
Ramu 1999[305]	Chlorpromazine 250 (300-300)	27	8	n.i.	acute schizophrenia (clinical diagnosis)	2
Ramu 1999[305]	Placebo	27	8	n.i.	acute schizophrenia (clinical diagnosis)	2
Ramu 1999a[306]	Chlorpromazine 375 (300-450)	22	11	4.7	chronic schizophrenia (clinical diagnosis)	2

Study name	Antipsychotics and daily dose in mg, on flexible dosage mean value (range)	N	Duration in weeks	Mean duration of illness in years	Diagnosis	Blinding
	Placebo	20	11	4.63	chronic schizophrenia (clinical diagnosis)	2
Reardon 1966[307]	Chlorpromazine 600 (600-600)	11	4	n.i.	acute paranoid schizophrenia (Bleulerian criteria)	1
	Placebo	12	4	n.i.	acute paranoid schizophrenia (Bleulerian criteria)	1
	Trifluoperazine 40 (40-40)	11	4	n.i.	acute paranoid schizophrenia (Bleulerian criteria)	1
Remvig 1987[308]	Perphenazine 30 (8-72)	18	12	6	Acute psychosis (clinical Diagnosis)	2
	Zuclopenthixol 37 (10-120)	22	12	12	Acute psychosis (clinical Diagnosis)	2
Rickels 1978[309]	Chlorpromazine 926 (400-1600)	40	3	n.i.	schizophrenic (clinical Diagnosis)	2
	Thiothixene 44.3 (20-80)	39	3	n.i.	schizophrenic (clinical Diagnosis)	2
Riedel 2007[310]	Olanzapine 15.82 (10-20)	26	8	4.71	schizophrenia, acute episode (DSM-IV)	2
	Quetiapine 586.86 (400-800)	26	8	8.44	schizophrenia, acute episode (DSM-IV)	2
Rifkin 1984[311]	Chlorpromazine 1288 (300-1500)	33	4	n.i.	schizophrenia (clinical Diagnosis)	2
	Loxapine 128.6 (30-150)	31	4	n.i.	schizophrenia (clinical Diagnosis)	2
Rodova 1973[312]	Clozapine 364.35 (147.8-580.9)	n.i.	3	n.i.	schizophrenia (clinical Diagnosis)	2
	Perphenazine 40.8 (17.8-63.8)	n.i.	3	n.i.	schizophrenia (clinical Diagnosis)	2

Study name	Antipsychotics and daily dose in mg, on flexible dosage mean value (range)	N	Duration in weeks	Mean duration of illness in years	Diagnosis	Blinding
Rosenheck 2003w6[313]	Haloperidol 11.2 (5-20)	150	6	21.8	schizophrenia, schizoaffective disorder (DSM-IV)	2
	Olanzapine 11.4 (5-20)	159	6	23.1	schizophrenia, schizoaffective disorder (DSM-IV)	2
Rubin 1971[314]	Haloperidol 11 (2-20)	10	6	n.i.	schizophrenia (clinical Diagnosis)	2
	Trifluoperazine 33 (6-60)	8	6	n.i.	schizophrenia (clinical Diagnosis)	2
Ruiz Navarro 1976[315]	Chlorpromazine 600 (600-600)	5	12	n.i.	chronic schizophrenics (clinical Diagnosis)	2
	Thiothixene 30 (30-30)	5	12	n.i.	chronic schizophrenics (clinical Diagnosis)	2
Rüther 1988[316]	Amisulpride 482 (400-1000)	15	4	n.i.	schizophrenia (295,1-295,7) (ICD-9)	2
	Perazine 476 (400-1000)	15	4	n.i.	schizophrenia (295,1-295,7) (ICD-9)	2
Sacchetti 2008[317]	Olanzapine 15.1 (10-20)	25	8	7	schizophrenia (DSM-IV-TR)	1
	Quetiapine 590 (400-800)	25	8	13	schizophrenia (DSM-IV-TR)	1
	Risperidone 5.1 (4-8)	25	8	10	schizophrenia (DSM-IV-TR)	1
Safa 2008[318]	Olanzapine 17.5 (15-20)	32	12	n.i.	schizophrenia or schizoaffective disorder (DSM-IV-TR)	2
	Risperidone 9 (6-12)	31	12	n.i.	schizophrenia or schizoaffective disorder (DSM-IV-TR)	2

Study name	Antipsychotics and daily dose in mg, on flexible dosage mean value (range)	N	Duration in weeks	Mean duration of illness in years	Diagnosis	Blinding
Sakalis 1977[319]	Chlorpromazine 1000 (1000-1000)	33	4	n.i.	schizophrenia, newly hospitalized (Research diagnosis)	2
	Placebo	17	4	n.i.	schizophrenia, newly hospitalized (Research diagnosis)	2
Sandison 1960[320]	Thioridazine 300 (300-300)	8	12	6	schizophrenia (clinical Diagnosis)	2
	Placebo	7	12	5	schizophrenia (clinical Diagnosis)	2
Sarai 1987[321]	Zotepine 187.5 (75-300)	48	8	n.i.	schizophrenia (clinical Diagnosis)	2
	Thiothixene 37.5 (15-60)	46	8	n.i.	schizophrenia (clinical Diagnosis)	2
Saretsky 1966[322]	Chlorpromazine 400 (400-400)	20	13	n.i.	schizophrenic reaction (clinical diagnosis)	2
	Placebo	20	13	n.i.	schizophrenic reaction (clinical diagnosis)	2
Sato 2012[323]	Aripiprazole n.i. (n.i.-n.i.)	n.i.	8	13.1	schizophrenia (DSM-IV)	1
	Risperidone n.i. (n.i.-n.i.)	n.i.	8	13.1	schizophrenia (DSM-IV)	1
Schennach 2018[324]	Haloperidol 7.5 (6-9)	58	4	n.i.	schizophrenia (DSM-IV)	2
	Ziprasidone 140 (120-160)	54	4	n.i.	schizophrenia (DSM-IV)	2
Schiele 1975[325]	Chlorpromazine 1100 (200-1500)	24	12	n.i.	diagnosis of chronic schizophrenia (clinical Diagnosis)	2
	Loxapine 110 (20-150)	26	12	n.i.	diagnosis of chronic schizophrenia (clinical Diagnosis)	2

Study name	Antipsychotics and daily dose in mg, on flexible dosage mean value (range)	N	Duration in weeks	Mean duration of illness in years	Diagnosis	Blinding
Schimmelmann 2005[326]	Amisulpride 400 (100-700)	15	5.7	7.2	schizophrenia and schizophreniform disorder (ICD-10)	0
	Clozapine 100 (25-275)	5	5.7	7.2	schizophrenia and schizophreniform disorder (ICD-10)	0
	Olanzapine 15 (5-15)	14	5.7	7.2	schizophrenia and schizophreniform disorder (ICD-10)	0
	Risperidone 4 (2-6)	11	5.7	7.2	schizophrenia and schizophreniform disorder (ICD-10)	0
	Zotepine 275 (75-325)	18	5.7	7.2	schizophrenia and schizophreniform disorder (ICD-10)	0
Schmidt 1982[327]	Haloperidol 24.5 (15-45)	15	4	2.7	acute paranoid schizophrenia (ICD 295.3) (ICD-9)	2
	Perazine 676 (300-900)	17	4	4.8	acute paranoid schizophrenia (ICD 295.3) (ICD-9)	2
Schmidt 2014[328]	Placebo	101	6	10.9	schizophrenia, acute schizophrenia (DSM-IV)	2
	Olanzapine 15 (15-15)	93	6	10.7	schizophrenia, acute schizophrenia (DSM-IV)	2
See 1999[329]	Risperidone 5 (4-6)	10	5	9.1	chronic schizphrenia (DSM-IV)	2
	Haloperidol 22.5 (15-30)	10	5	11.6	chronic schizphrenia (DSM-IV)	2
Selman 1976[330]	Haloperidol 8.8 (4-12)	29	12	n.i.	acute exacerbation of chronic schizophrenia (clinical diagnosis)	2

Study name	Antipsychotics and daily dose in mg, on flexible dosage mean value (range)	N	Duration in weeks	Mean duration of illness in years	Diagnosis	Blinding
	Placebo	29	12	n.i.	acute exacerbation of chronic schizophrenia (clinical diagnosis)	2
	Loxapine 110 (50-150)	29	12	n.i.	acute exacerbation of chronic schizophrenia (clinical diagnosis)	2
Serafetinides 1972[331]	Chlorpromazine 830 (200-1000)	14	12	17	chronic schizophrenia (clinical diagnosis)	2
	Clopenthixol 205 (50-250)	15	12	15	chronic schizophrenia (clinical diagnosis)	2
	Haloperidol 12.3 (3-15)	14	12	13	chronic schizophrenia (clinical diagnosis)	2
	Placebo	14	12	14	chronic schizophrenia (clinical diagnosis)	2
Sergi 2007[332]	Risperidone 4 (4-4)	40	8	n.i.	schizophrenia, schizoaffective disorder (DSM-IV)	2
	Haloperidol 8 (8-8)	20	8	n.i.	schizophrenia, schizoaffective disorder (DSM-IV)	2
	Olanzapine 15 (15-15)	40	8	n.i.	schizophrenia, schizoaffective disorder (DSM-IV)	2
Seth 1979[333]	Trifluoperazine 25 (5-45)	31	12	n.i.	chronic schizophrenic (clinical Diagnosis)	2
	Loxapine 55 (20-90)	33	12	n.i.	chronic schizophrenic (clinical Diagnosis)	2
Shah 2011[334]	Olanzapine 12.5 (5-20)	105	6	n.i.	schizophrenia (DSM-IV)	2
	Paliperidone 7.5 (3-12)	109	6	n.i.	schizophrenia (DSM-IV)	2
Shen 2014[335]	Placebo	78	6	n.i.	acute schizophrenia (DSM-IV-TR)	2

Study name	Antipsychotics and daily dose in mg, on flexible dosage mean value (range)	N	Duration in weeks	Mean duration of illness in years	Diagnosis	Blinding
	Olanzapine 15 (15-15)	77	6	n.i.	acute schizophrenia (DSM-IV-TR)	2
Sheperd 1956, 01660[336]	Placebo	8	6	n.i.	schizophrenia (clinical diagnosis)	2
	Chlorpromazine 300 (300-300)	8	6	n.i.	schizophrenia (clinical diagnosis)	2
Shopsin 1972[337]	Chlorpromazine 750 (300-1200)	15	3	n.i.	schizophrenia (clinical Diagnosis)	2
	Loxapine 75 (30-120)	15	3	n.i.	schizophrenia (clinical Diagnosis)	2
Silverstone 1984[338]	Haloperidol 22.2 (5-50)	12	4	n.i.	Acute schizophrenic illness (clinical Diagnosis)	2
	Pimozide 21.6 (5-40)	10	4	n.i.	Acute schizophrenic illness (clinical Diagnosis)	2
Simpson 1971[339]	Molindone 75 (n.i.-120)	27	4	n.i.	schizophrenia (clinical Diagnosis)	2
	Trifluoperazine 35 (n.i.-45)	25	4	n.i.	schizophrenia (clinical Diagnosis)	2
Simpson 1976[340]	Loxapine 73.8 (n.i.-n.i.)	24	4	n.i.	schizophrenia (clinical Diagnosis)	2
	Trifluoperazine 35.25 (n.i.-n.i.)	19	4	n.i.	schizophrenia (clinical Diagnosis)	2
Simpson 2004[341]	Olanzapine 11.3 (5-15)	133	6	14	schizophrenia or schizoaffective disorder (DSM-IV)	2
	Ziprasidone 129.9 (80-160)	136	6	15.4	schizophrenia or schizoaffective disorder (DSM-IV)	2
Singer 1974[342]	Chlorpromazine 196 (75-600)	20	5.7	3.3	acute schizophrenia (clinical Diagnosis)	2

Study name	Antipsychotics and daily dose in mg, on flexible dosage mean value (range)	N	Duration in weeks	Mean duration of illness in years	Diagnosis	Blinding
	Clozapine 155 (50-300)	20	5.7	1.7	acute schizophrenia (clinical Diagnosis)	2
Singh 1975[343]	Chlorpromazine 677.8 (300-1200)	9	6	n.i.	schizophrenia (clinical Diagnosis)	2
	Haloperidol 7.5 (3-60)	9	6	n.i.	schizophrenia (clinical Diagnosis)	2
Small 1997[344]	Quetiapine 360 (250-750)	96	6	n.i.	(sub-)chronic schizophrenia with acute exacerbation (DSM-III-R)	2
	Placebo	96	6	n.i.	(sub-)chronic schizophrenia with acute exacerbation (DSM-III-R)	2
Sonmez 2009[345]	Risperidone 6 (4-8)	11	6	n.i.	schizophrenia or schizoaffective disorder (DSM-IV-TR)	0
	Ziprasidone 120 (80-160)	11	6	n.i.	schizophrenia or schizoaffective disorder (DSM-IV-TR)	0
Spohn 1977[346]	Placebo	20	8	n.i.	chronic schizophrenia (clinical diagnosis)	2
	Chlorpromazine n.i. (200-n.i.)	20	8	n.i.	chronic schizophrenia (clinical diagnosis)	2
Steinbook 1973[347]	Chlorpromazine 850 (200-1500)	28	6	n.i.	schizophrenia (clinical Diagnosis)	2
	Loxapine 85 (20-150)	26	6	n.i.	schizophrenia (clinical Diagnosis)	2
Study 006[348]	Lurasidone 79.6 (40-120)	99	6	n.i.	schizophrenia (DSM-IV)	2
	Placebo	50	6	n.i.	schizophrenia (DSM-IV)	2

Study name	Antipsychotics and daily dose in mg, on flexible dosage mean value (range)	N	Duration in weeks	Mean duration of illness in years	Diagnosis	Blinding
Study 049[349]	Haloperidol 10 (10-10)	73	6	n.i.	acute exacerbation of schizophrenia (DSM-IV)	2
	Lurasidone 60.3 (40-80)	140	6	n.i.	acute exacerbation of schizophrenia (DSM-IV)	2
	Placebo	72	6	n.i.	acute exacerbation of schizophrenia (DSM-IV)	2
Study 115 2000[350]	Placebo	83	6	n.i.	(sub-)chronic schizophrenia (DSM-III-R)	2
	Ziprasidone 161.9512195 (120-200)	164	6	n.i.	(sub-)chronic schizophrenia (DSM-III-R)	2
	Haloperidol 15 (15-15)	85	6	n.i.	(sub-)chronic schizophrenia (DSM-III-R)	2
Study 196[351]	Placebo	90	6	n.i.	schizophreniform or schizoaffective disorder or catatonic or residual subtypes of schizophernia (DSM-IV)	2
	Lurasidone 80 (80-80)	90	6	n.i.	schizophreniform or schizoaffective disorder or catatonic or residual subtypes of schizophernia (DSM-IV)	2
Study 229[352]	Lurasidone 79.9 (40-120)	372	6	14.37	schizophernia (DSM-IV)	2
	Placebo	128	6	14	schizophernia (DSM-IV)	2
Study 231[353]	Placebo	116	6	12.6	acute exacerbation of schizophernia (DSM-IV)	2

Study name	Antipsychotics and daily dose in mg, on flexible dosage mean value (range)	N	Duration in weeks	Mean duration of illness in years	Diagnosis	Blinding
	Olanzapine 15 (15-15)	123	6	13.2	acute exacerbation of schizophernia (DSM-IV)	2
	Lurasidone 79.8 (40-120)	239	6	14	acute exacerbation of schizophernia (DSM-IV)	2
Study 233[354]	Quetiapine 600 (600-600)	120	6	12.4	acute exacerbation of schizophernia (DSM-IV-TR)	2
	Lurasidone 119.35 (80-160)	246	6	11.45	acute exacerbation of schizophernia (DSM-IV-TR)	2
	Placebo	122	6	11.3	acute exacerbation of schizophernia (DSM-IV-TR)	2
Study 3000[355]	Placebo	127	6	15.6	acute exacerbation of schizophrenia (DSM-IV)	2
	Haloperidol 15 (15-15)	124	6	15.6	acute exacerbation of schizophrenia (DSM-IV)	2
	Iloperidone 12 (12-12)	124	6	15.6	acute exacerbation of schizophrenia (DSM-IV)	2
Study 3001[356]	Haloperidol 11.9 (5-20)	146	6	12	schizophrenia or schizoaffective disorder (DSM-IV)	2
	Iloperidone 11.4 (4-16)	454	6	12	schizophrenia or schizoaffective disorder (DSM-IV)	2
Study 3002[356]	Iloperidone 12.9 (4-16)	420	6	9.7	schizophrenia or schizoaffective disorder (DSM-IV)	2
	Haloperidol 14 (5-20)	137	6	9.7	schizophrenia or schizoaffective disorder (DSM-IV)	2

Study name	Antipsychotics and daily dose in mg, on flexible dosage mean value (range)	N	Duration in weeks	Mean duration of illness in years	Diagnosis	Blinding
Study 3003[356]	Haloperidol 14.5 (5-20)	122	6	13.4	schizophrenia or schizoaffective disorder (DSM-IV)	2
	Iloperidone 13.3 (4-16)	365	6	13.4	schizophrenia or schizoaffective disorder (DSM-IV)	2
Study 3004[355]	Iloperidone 14.58 (10-16)	154	6	n.i.	schizophrenia and schizoaffective disorder (DSM-IV)	2
	Placebo	156	6	n.i.	schizophrenia and schizoaffective disorder (DSM-IV)	2
	Risperidone 7.02 (4-8)	153	6	n.i.	schizophrenia and schizoaffective disorder (DSM-IV)	2
Study 3005[355]	Risperidone 7.09 (6-8)	157	6	14.4	acute exacerbation of schizophrenia and schizoaffective disorder (DSM-IV)	2
	Iloperidone 18 (12-24)	389	6	n.i.	acute exacerbation of schizophrenia and schizoaffective disorder (DSM-IV)	2
	Placebo	160	6	15.6	acute exacerbation of schizophrenia and schizoaffective disorder (DSM-IV)	2
Study 93202 2002[357]	Aripiprazole 30 (30-30)	34	4	n.i.	schizophrenia acute relapse (DSM-III-R)	2
	Haloperidol 20 (20-20)	34	4	n.i.	schizophrenia acute relapse (DSM-III-R)	2
	Placebo	35	4	n.i.	schizophrenia acute relapse (DSM-III-R)	2
Study 94202 2002[358]	Aripiprazole 30 (30-30)	61	4	n.i.	schizophrenia acute relapse (DSM-IV)	2

Study name	Antipsychotics and daily dose in mg, on flexible dosage mean value (range)	N	Duration in weeks	Mean duration of illness in years	Diagnosis	Blinding
	Haloperidol 10 (10-10)	63	4	n.i.	schizophrenia acute relapse (DSM-IV)	2
	Placebo	64	4	n.i.	schizophrenia acute relapse (DSM-IV)	2
Study RGH-MD-03[359]	Placebo	130	6	17.7	schizophrenia (DSM-IV-TR)	2
	Cariprazine 3.83 (1.5-4.5)	128	6	17.2	schizophrenia (DSM-IV-TR)	2
Study RGH-MD-05[360]	Cariprazine 5.2 (3-6)	151	6	11.3	schizophrenia (DSM-IV-TR)	2
	Placebo	147	6	11	schizophrenia (DSM-IV-TR)	2
Study RIS-USA-72 1996[361]	Risperidone 5.91 (4-8)	163	4	n.i.	schizophrenia (DSM-IV)	2
	Placebo	83	4	n.i.	schizophrenia (DSM-IV)	2
Svendsen 1961[362]	Chlorpromazine n.i. (n.i.-n.i.)	n.i.	12	n.i.	schizophrenia (clinical Diagnosis)	2
	Thioridazine n.i. (n.i.-n.i.)	n.i.	12	n.i.	schizophrenia (clinical Diagnosis)	2
Svestka 1972[363]	Perphenazine 100 (8-192)	n.i.	3	n.i.	schizophrenia or schizoaffective disorder (clinical Diagnosis)	0
	Pimozide 15.5 (1-30)	n.i.	3	n.i.	schizophrenia or schizoaffective disorder (clinical Diagnosis)	0
Svestka 1990[364]	Haloperidol 11 (2-20)	18	8	7.7	schizophrenia or schizoaffective disorder (ICD-9)	2
	Risperidone 11 (2-20)	18	8	8.3	schizophrenia or schizoaffective disorder (ICD-9)	2

Study name	Antipsychotics and daily dose in mg, on flexible dosage mean value (range)	N	Duration in weeks	Mean duration of illness in years	Diagnosis	Blinding
Svestka 1990a[365]	Sulpiride n.i. (n.i.-n.i.)	n.i.	3	n.i.	schizophrenia or schizoaffective disorder (clinical Diagnosis)	2
	Perphenazine n.i. (n.i.-n.i.)	n.i.	3	n.i.	schizophrenia or schizoaffective disorder (clinical Diagnosis)	2
Svestka 2003a[366]	Quetiapine 677.3 (50-700)	22	6	7.07	Acute schizophrenia or schizoaffective disorder (ICD-10)	2
	Olanzapine 19.5 (10-20)	20	6	7.03	Acute schizophrenia or schizoaffective disorder (ICD-10)	2
Svestka 2005[367]	Olanzapine n.i. (n.i.-n.i.)	24	6	n.i.	acute schizophrenia or schizoaffective disorder (ICD-10)	2
	Ziprasidone n.i. (n.i.-n.i.)	24	6	n.i.	acute schizophrenia or schizoaffective disorder (ICD-10)	2
Tamrakar 2006[368]	Haloperidol 15 (10-20)	18	6	n.i.	schizophrenia (ICD-10)	0
	Risperidone 5 (4-6)	18	6	n.i.	schizophrenia (ICD-10)	0
Taneli 2003[369]	Quetiapine 487 (50-750)	45	12	n.i.	exacerbation of schizophrenia of chronic or subchronic schizophrenia (DSM-IV)	0
	Haloperidol 10 (5-20)	34	12	n.i.	exacerbation of schizophrenia of chronic or subchronic schizophrenia (DSM-IV)	0
Tapp 2005[370]	Haloperidol 12.5 (5-20)	9	12	20.75	schizophrenia or schizoaffective disorder (n.i.)	2
	Quetiapine 450 (300-600)	11	12	20.75	schizophrenia or schizoaffective disorder (n.i.)	2

Study name	Antipsychotics and daily dose in mg, on flexible dosage mean value (range)	N	Duration in weeks	Mean duration of illness in years	Diagnosis	Blinding
Tetreault 1969a[371]	Chlorpromazine 600 (600-600)	15	12	n.i.	chronic schizophrenia (clinical diagnosis)	2
	Placebo	15	12	n.i.	chronic schizophrenia (clinical diagnosis)	2
Tollefson 1997[372]	Olanzapine 13.2 (5-20)	1336	6	14.21	schizophrenia, schizophreniform disorder, schizoaffective disorder (DSM-III-R)	2
	Haloperidol 11.8 (5-20)	660	6	14.9	schizophrenia, schizophreniform disorder, schizoaffective disorder (DSM-III-R)	2
Tuason 1984[373]	Chlorpromazine 570 (300-1500)	34	4	n.i.	schizophrenia, paranoid type (clinical Diagnosis)	2
	Loxapine 59 (30-150)	34	4	n.i.	schizophrenia, paranoid type (clinical Diagnosis)	2
Tybura 2014[374]	Perazine 450 (300-600)	60	12	n.i.	schizophrenia (ICD-10)	0
	Olanzapine 15 (10-20)	72	12	n.i.	schizophrenia (ICD-10)	0
	Ziprasidone 140 (120-160)	59	12	n.i.	schizophrenia (ICD-10)	0
van Bruggen 2003[375]	Risperidone 4.4 (1-8)	26	6	2.1	first or second episode schizophrenia (DSM-IV)	0
	Olanzapine 15.6 (5-30)	18	6	3.8	first or second episode schizophrenia (DSM-IV)	0
van der Velde 1975[376]	Placebo	28	6	n.i.	schizophrenia, acute exacerbation (clinical diagnosis)	2

Study name	Antipsychotics and daily dose in mg, on flexible dosage mean value (range)	N	Duration in weeks	Mean duration of illness in years	Diagnosis	Blinding
	Thiothixene 50 (40-60)	28	6	n.i.	schizophrenia, acute exacerbation (clinical diagnosis)	2
	Loxapine 102.5 (100-150)	26	6	n.i.	schizophrenia, acute exacerbation (clinical diagnosis)	2
van Kammen 1996[377]	Sertindole 16.11 (12-20)	105	5.7	13.3	schizophrenia (DSM-III-R)	2
	Placebo	48	5.7	13.8	schizophrenia (DSM-III-R)	2
Vichaya 1971[378]	Haloperidol 4.5 (4.5-4.5)	15	6	n.i.	chronic schizophrenia (clinical diagnosis)	2
	Placebo	15	6	n.i.	chronic schizophrenia (clinical diagnosis)	2
Wagner 2005[379]	Amisulpride 511.1 (400-800)	26	8	9.8	schizophrenia (DSM-IV, ICD-10)	2
	Olanzapine 15 (10-20)	26	8	7	schizophrenia (DSM-IV, ICD-10)	2
Waldrop 1961[380]	Chlorpromazine 740 (n.i.-800)	78	13	n.i.	schizophrenia (clinical Diagnosis)	2
	Thioridazine 740 (n.i.-800)	78	13	n.i.	schizophrenia (clinical Diagnosis)	2
Walsh 1959[381]	Placebo	22	8	n.i.	chronic schizophrenia (clinical diagnosis)	2
	Chlorpromazine 255 (75-300)	22	8	n.i.	chronic schizophrenia (clinical diagnosis)	2
Weston 1973[382]	Haloperidol 5.3 (4.5-9)	42	12	21.5	schizophrenia (clinical Diagnosis)	2
	Thioridazine 330 (300-600)	44	12	20.6	schizophrenia (clinical Diagnosis)	2

Study name	Antipsychotics and daily dose in mg, on flexible dosage mean value (range)	N	Duration in weeks	Mean duration of illness in years	Diagnosis	Blinding
Wetzel 1991[383]	Zotepine 250 (100-600)	20	4	n.i.	schizophrenia (ICD-9)	2
	Perazine 500 (150-900)	21	4	n.i.	schizophrenia (ICD-9)	2
Wetzel 1998[384]	Flupentixol 22.6 (15-25)	62	6	n.i.	schizophrenia; paranoid or undifferentiated (DSM-III-R)	2
	Amisulpride 956 (600-1000)	70	6	n.i.	schizophrenia; paranoid or undifferentiated (DSM-III-R)	2
Wynn 2007[385]	Haloperidol 8 (8-8)	11	8	n.i.	schizophrenia, schizoaffective disorder (DSM-IV)	2
	Olanzapine 15 (15-15)	21	8	n.i.	schizophrenia, schizoaffective disorder (DSM-IV)	2
	Risperidone 4 (4-4)	19	8	n.i.	schizophrenia, schizoaffective disorder (DSM-IV)	2
Yamashita 2004[386]	Olanzapine 16.5 (2.5-20)	20	8	n.i.	schizophrenia (DSM-IV)	0
	Risperidone 7.4 (1-12)	20	8	n.i.	schizophrenia (DSM-IV)	0
	Quetiapine 432.5 (50-750)	28	8	n.i.	schizophrenia (DSM-IV)	0
Yen 2004[387]	Risperidone 4.4 (1-12)	21	12	10.5	schizophrenia (DSM-III-R)	1
	Haloperidol 11.2 (2-20)	20	12	11.5	schizophrenia (DSM-III-R)	1
Zborowski 1995[388]	Haloperidol 16 (16-16)	115	8	15.4	schizophrenia (DSM-III-R/DSM-IV)	2
	Placebo	116	8	15.4	schizophrenia (DSM-III-R/DSM-IV)	2

Study name	Antipsychotics and daily dose in mg, on flexible dosage mean value (range)	N	Duration in weeks	Mean duration of illness in years	Diagnosis	Blinding
	Sertindole 20 (20-20)	117	8	15.7	schizophrenia (DSM-III-R/DSM-IV)	2
Zhang 2010[389]	Haloperidol 20 (0-40)	30	4	n.i.	schizophrenic patients (clinical Diagnosis)	1
	Quetiapine 400 (0-800)	30	4	n.i.	schizophrenic patients (clinical Diagnosis)	1
Zhang 2011[390]	Risperidone 3.8 (2-6)	121	6	5.3	schizophrenia (Chinese Classification of Mental Disorders)	2
	Ziprasidone 118.5 (80-160)	118	6	6.4	schizophrenia (Chinese Classification of Mental Disorders)	2
Zhong 2006[391]	Risperidone 5.2 (2-8)	335	8	n.i.	schizophrenia (DSM-IV)	2
	Quetiapine 525 (200-800)	338	8	n.i.	schizophrenia (DSM-IV)	2
Ziegler 1989[392]	Amisulpride 525 (300-750)	20	4	5	schizophrenia (ICD-9)	2
	Haloperidol 12.25 (2.5-22.5)	20	4	7.5	schizophrenia (ICD-9)	2
Zimbroff 1997[393]	Sertindole 15.78 (12-20)	144	8	16.04	schizophrenia (DSM-III-R/DSM-IV)	2
	Haloperidol 12.09 (8-16)	137	8	16.79	schizophrenia (DSM-III-R/DSM-IV)	2
	Placebo	73	8	16.6	schizophrenia (DSM-III-R/DSM-IV)	2
Zimbroff 2007[394]	Aripiprazole 20.9 (10-30)	129	4	n.i.	schizophrenia or schizoaffective disorder (DSM-IV)	2
	Ziprasidone 149 (80-160)	127	4	n.i.	schizophrenia or schizoaffective disorder (DSM-IV)	2

References

1 A1281046 S. Study report on study a1281046. *Pfizer, data on file* 2005.

2 A1281050 S. Study report on study a1281050. *Pfizer, data on file* 2004.

3 Abdolahian E, Mohareri F, Bordbar MRF. Haloperidol versus risperidone: A comparison of beneficial effect on cognitive function of patients with chronic schizophrenia. *Iranian Journal of Psychiatry and Behavioral Sciences* 2008; **2**: 14–20.

4 Addington DE, Pantelis C, Dineen M, Benattia I, Romano SJ. Efficacy and tolerability of ziprasidone versus risperidone in patients with acute exacerbation of schizophrenia or schizoaffective disorder: An 8-week, double-blind, multicenter trial. *Journal of clinical Psychiatry* 2004; **65**: 1624–33.

5 Ahmed S, Casey D. E., Yeung P. P., Barbato L. M., Heisterberg J., Shapira N. A. Lipid profile among patients with schizophrenia randomized to bifeprunox, placebo, or olanzapine: A comparison of results: 11th international congress on schizophrenia research; 2007 mar 28-apr 1, colorado springs, colorado, usa. 2007.

6 Ahn YM, Lee KY, Kim KS, Kim DK, Kim YH, Kim CY. Body weight gain and metabolic changes in patients with schizophrenia during 12-week randomized treatment of ziprasidone and risperidone: Korean journal of psychopharmacology. 2007; **18**: 92–102.

7 Allan ER, Sison CE, Alpert M, Connolly B, Crichton J. The relationship between negative symptoms of schizophrenia and extrapyramidal side effects with haloperidol and olanzapine. *Psychopharmacology Bulletin* 1998; **34**: 71–4.

8 Amakusa T, Majima T. The comparison of therapeutic effects of fk-880 (sulpiride) and perphenazine in schizophrenia by a double-blind controlled study. *Juntendo Igako* 1973; **19**: 239–49.

9 Angst J, Jaenicke U, Padrutt A, Scharfetter C. Ergebnisse eines doppelblindversuches von hf 1854 * (8-chlor-11-(4-methyl-1-piperazinyl)-5H-dibenzo (b, e) (1,4) diazepin) im vergleich zu levomepromazin. *Pharmacopsychiatry* 1971; **4**: 192–200.

10 Anumonye A, Onibuwe-Johnson T, Marinho AA. clinical trial of pimozide. *West African Journal of Pharmacology & Drug Research* 1976; **3**: 17–24.

11 Arvanitis LA, Miller BG. Multiple fixed doses of Seroquel (quetiapine) in patients with acute exacerbation of schizophrenia: A comparison with haloperidol and placebo. The seroquel trial 13 study group. *Biological Psychiatry* 1997; **42**: 233–46.

12 Atmaca M, Kuloglu M, Tezcan E, Canatan H, Gecici O. Quetiapine is not associated with increase in prolactin secretion in contrast to haloperidol. *Archives of Medical Research* 2002; **33**: 562–5.

13 Augustin BG, Jann MW, Crabtree BL, Pitts WM, Carter JG. Plasma alpha-one acid glycoprotein (aag) and haloperidol (h) concentrations in schizophrenic patients: 20th collegium internationale neuro-psychopharmacologicum congress; 1996 jun 23-27; melbourne, australia. 1996.

14 Avasthi A, Kulhara P, Kakkar N. Olanzapine in the treatment of schizophrenia: An open label comparative clinical trial from north india. *Indian Journal of Psychiatry* 2001; **43**: 257–63.

15 Azorin JM, Strub N, Loft H. A double-blind, controlled study of sertindole versus risperidone in the treatment of moderate-to-severe schizophrenia. *International clinical Psychopharmacology* 2006; **21**: 49–56.

16 Baker JP. A controlled trial of ethylcrotonylurea. *JMentSci* 1959; **105**: 852–62.

17 Balasubramanian K, Baloch N, Briscoe MH *et al.* A double blind multicentre comparison of oral zuclopenthixol and oral chlorpromazine in the treatment of acute psychosis: British journal of clinical research. 1991; **2**: 149–56.

18 Ban TA, Lehmann HE, Sterlin C, Climan M. Comprehesiven clinical studies with thiothixene. *Diseases of the Nervous System* 1975; **36**: 473–7.

19 Barbato LMNJWHJYPPSNA. Efficacy and metabolic profile of bifeprunox in patients with schizophrenia: 11th international congress on schizophrenia research; 2007 mar 28-apr 1, colorado springs, colorado, usa. 2007.

20 Beasley, C. M., Jr., Sanger T, Satterlee W, Tollefson G, Tran P, Hamilton S. Olanzapine versus placebo: Results of a double-blind, fixed-dose olanzapine trial. *Psychopharmacology* 1996; **124**: 159–67.

21 Beasley, C. M., Jr., Tollefson G, Tran P, Satterlee W, Sanger T, Hamilton S. Olanzapine versus placebo and haloperidol: Acute phase results of the north american double-blind olanzapine trial. *Neuropsychopharmacology* 1996; **14**: 111–23.

22 Beasley, C. M., Jr., Hamilton SH, Crawford AM *et al.* Olanzapine versus haloperidol: Acute phase results of the international double-blind olanzapine trial. *European Neuropsychopharmacology* 1997; **7**: 125–37.

23 Bechelli LP, Ruffino-Netto A, Hetem G. A double-blind controlled trial of pipotiazine, haloperidol and placebo in recently-hospitalized acute schizophrenic patients. *Brazilian Journal of Medical & Biological Research* 1983; **16**: 305–11.

24 Bergling R, Mjorndal T, Oreland L, Rapp W, Wold S. Plasma levels and clinical effects of thioridazine and thiothixene. *Journal of clinical Pharmacology* 1975; **15**: 178–86.

25 Bernardo M, Parellada E, Lomena F *et al.* Double-blind olanzapine vs. Haloperidol d2 dopamine receptor blockade in schizophrenic patients: A baseline-endpoint. *Psychiatry Research* 2001; **107**: 87–97.

26 Bhowmick S, Hazra A, Ghosh M. Amisulpride versus olanzapine in the treatment of schizophrenia in indian patients: Randomized controlled trial. *Australian & New Zealand Journal of Psychiatry* 2010; **44**: 237–42.

27 Bishop MPGDM. Behavioral toxicity associated with benzquinamide (quantril) therapy in schizophrenic patients: American journal of psychiatry. 1963; **120**: 180–1.

28 Bishop MP, Gallant DM, Nesselhof W, Sprehe DJ. A controlled evaluation of butaperazine in chronic schizophrenic patients: Diseases of the nervous system. 1964; **25**: 674–83.

29 Bishop MP, Gallant DM. Loxapine: A controlled evaluation in chronic schizophrenic patients. *Current Therapeutic Research, clinical & Experimental* 1970; **12**: 594–7.

30 Blin O, Azorin JM, Bouhours P. Antipsychotic and anxiolytic properties of risperidone, haloperidol, and methotrimeprazine in schizophrenic patients. *Journal of clinical Psychopharmacology* 1996; **16**: 38–44.

31 Boehle C, Volz HP, Hornstein C *et al.* Neuropsychological performance of clozapine treated schizophrenics: Pharmacopsychiatry. 1995; **28**: 166.

32 Borison RL, Sinha D, Haverstock S, McLarnon MC, Diamond BI. Efficacy and safety of tiospirone vs. Haloperidol and thioridazine in a double-blind, placebo-controlled trial. *Psychopharmacology Bulletin* 1989; **25**: 190–3.

33 Borison RL, Diamond BI, Dren AT. Does sigma receptor antagonism predict clinical antipsychotic efficacy? *Psychopharmacology Bulletin* 1991; **27**: 103–6.

34 Borison RL, Pathiraja AP, Diamond BI, Meibach RC. Risperidone: clinical safety and efficacy in schizophrenia. *Psychopharmacology Bulletin* 1992; **28**: 213–8.

35 Borison RL, Arvanitis LA, Miller BG. ICI 204,636, an atypical antipsychotic: Efficacy and safety in a multicenter, placebo-controlled trial in patients with schizophrenia. U.S. SEROQUEL study group. *J Clin Psychopharmacol* 1996; **16**: 158–69.

36 Boulay LJ, Labelle A, Bourget D *et al.* Dissociating medication effects from learning and practice effects in a neurocognitive study of schizophrenia: Olanzapine versus haloperidol. *Cognitive Neuropsychiatry* 2007; **12**: 322–38.

37 Bratfos O, Haug JO. Comparison of sulpiride and chlorpromazine in psychoses. A double-blind multicentre study. *Acta Psychiatrica Scandinavica* 1979; **60**: 1–9.

38 Brauzer B, Goldstein BJ. A clinical comparison of molindone hydrochloride with trifluoperazine in psychotic outpatients. *Current Therapeutic Research, clinical & Experimental* 1971; **13**: 152–7.

39 Brook S, Berk M, Selemani S, Kolloori J, Nzo I. A randomized controlled double blind study of zuclopenthixol acetate compared to haloperidol in acute psychosis. *Human Psychopharmacology* 1998; **13**: 17–20.

40 Brook S, Walden J, Benattia I, Siu CO, Romano SJ. Ziprasidone and haloperidol in the treatment of acute exacerbation of schizophrenia and schizoaffective disorder: Comparison of intramuscular and oral formulations in a 6-week, randomized, blinded-assessment study. *Psychopharmacology* 2005; **178**: 514–23.

41 Buchsbaum MS, Haznedar M, Newmark RE *et al.* FDG-pet and mri imaging of the effects of sertindole and haloperidol in the prefrontal lobe in schizophrenia. *Schizophrenia Research* 2009; **114**: 161–71.

42 Bueno JR. A double-blind comparative clinical trial with loxapine succinate and haloperidol in the treatment of schizophrenia. [Portuguese] ensaio clinico comparativo e duplo-cego com succinato de loxapina e haloperidol no tratamento da esquizofrenia. *Folha Medica* 1979; **78**: 47–52.

43 Bugarski-Kirola D, Wang A, Abi-Saab D, Blattler T. A phase ii/iii trial of bitopertin monotherapy compared with placebo in patients with an acute exacerbation of schizophrenia - results from the candlelyte study. *European Neuropsychopharmacology* 2014; **24**: 1024–36.

44 Burnett GB, Little SR, Graham N, Forrest AD. The assessment of thiothixene in chronic schizophrenia. A double-blind controlled trial. *Diseases of the Nervous System* 1975; **36**: 625–9.

45 Butler Andrew Wighton Claire, P. Welch Jack A. Tweed Bill D. Byrom Chris Reynolds A. The efficacy of zotepine in schizophrenia: A meta-analysis of bprs and improvement scale scores. *International Journal of Psychiatry in clinical Practice* 2000; **4**: 19–27.

46 Camara W, Paula AJM. A clinical comparative trial between loxapine succinate and thioridazine in the treatment of psychotic patients. [Portuguese] ensaio clinico comparativo com succinato de loxapine e thioridazine no trattamento de estados psicoticos. *Folha Medica* 1978; **77**: 73–9.

47 Canive JM, Miller GA, Irwin JG *et al.* Efficacy of olanzapine and risperidone in schizophrenia: A randomized double-blind crossover design. *Psychopharmacology Bulletin* 2006; **39**: 105–16.

48 Cantillon M. Efficacy and safety of novel dopamine serotonin stabilizer rp 5063 in acute schizophrenia and schizoaffective disorder. *Schizophrenia Research* 2014; **153**: S22.

49 Carranza J, Toro L. Double blind evaluation of sulpiride and thioridazine in paranoid schizophrenia. *Journal de Pharmacologie* 1974; **5**: 16.

50 Casey JF, Bennett IF, Lindley CJ, Hollister LE, Gordon MH, Springer NN. Drug therapy in schizophrenia. A controlled study of the relative effectiveness of chlorpromazine, promazine, phenobarbital, and placebo. *AMA Arch Gen Psychiatry* 1960; **2**: 210–20.

51 Casey DE, Sands EE, Heisterberg J, Yang HM. Efficacy and safety of bifeprunox in patients with an acute exacerbation of schizophrenia: Results from a randomized, double-blind, placebo-controlled, multicenter, dose-finding study. *Psychopharmacology* 2008; **200**: 317–31.

52 Cassano GB, Castrogiovanni P, Conti L, Bonollo L. Sulpiride versus haloperidol in schizophrenia: A double-blind comparative trial. *Current Therapeutic Research, clinical & Experimental* 1975; **17**: 189–201.

53 Cavallaro R, Mistretta P, Cocchi F, Manzato M, Smeraldi E. Differential efficacy of risperidone versus haloperidol in psychopathological subtypes of subchronic schizophrenia. *Human Psychopharmacology* 2001; **16**: 439–48.

54 Ceskova E, Svestka J. Double-blind comparison of risperidone and haloperidol in schizophrenic and schizoaffective psychoses. *Pharmacopsychiatry* 1993; **26**: 121–4.

55 Ceskova E, Svestka J. Risperidone vs. Perphenazine-a double blind comparison and prolactin plasma levels. *Psychiatria Danubina* 1994; **6**: 151–5.

56 Cetin M. A comparative study of atypical antipsychotics on sexual dysfunction. *International Journal of Neuropsychopharmacology* 2010; **13**: 89.

57 Chan HY, Lin WW, Lin SK *et al.* Efficacy and safety of aripiprazole in the acute treatment of schizophrenia in chinese patients with risperidone as an active control: A randomized trial. *Journal of clinical Psychiatry* 2007; **68**: 29–36.

58 Charalampous KD, Freemesser GF, Malev J, Ford K. Loxapine succinate: A controlled double-blind study in schizophrenia. *Current Therapeutic Research, clinical & Experimental* 1974; **16**: 829–37.

59 Chen YL, Chen KP, Chiu CC, Tai MH, Lung FW. Early predictors of poor treatment response in patients with schizophrenia treated with atypical antipsychotics. *BMC Psychiatry Vol 11 Feb 2011, ArtID 21* 2018; **18 (1) (no pagination)**. DOI:10.1186/s12888-018-1950-1.

60 Chiu E, Burrows G, Stevenson J. Double blind comparison of clozapine with chlorpromazine in acute schizophrenic illness. *Australian and New Zealand Journal of Psychiatry* 1976; **10**: 343–7.

61 Chouinard G, Lehmann HE, Ban TA. Pimozide in the treatment of chronic schizophrenic patients. *Current Therapeutic Research, clinical & Experimental* 1970; **12**: 598–603.

62 Chouinard G, Annable L, Serrano M, Albert JM, Charette R. Amitriptyline-perphenazine interaction in ambulatory schizophrenic patients. A controled study of drug interaction. *Archives of General Psychiatry* 1975; **32**: 1295–307.

63 Chouinard G, Annable L. Penfluridol in the treatment of newly admitted schizophrenic patients in a brief therapy unit: American journal of psychiatry. 1976; **133**: 850–3.

64 Chouinard G, Annable L. Pimozide in the treatment of newly admitted schizophrenic patients. *Psychopharmacology* 1982; **76**: 13–9.

65 Chouinard G. A placebo-controlled clinical trial of remoxipride and chlorpromazine in newly admitted schizophrenic patients with acute exacerbation. *Acta Psychiatrica Scandinavica, Supplementum* 1990; **358**: 111–9.

66 Chouinard G, Jones B, Remington G *et al.* A canadian multicenter placebo-controlled study of fixed doses of risperidone and haloperidol in the treatment of chronic schizophrenic patients.[Erratum appears in j clin psychopharmacol 1993 apr;13(2): 149]. *Journal of clinical Psychopharmacology* 1993; **13**: 25–40.

67 Chung YC, Park KH, Kim DJ, Park KY. Prolactin response to the administration of risperidone and haloperidol in patients with schizophrenia and other psychotic disorder: Korean journal of psychopharmacology. 2000; **11**: 343–9.

68 Chung TS, Lung FW. Different impacts of aquaporin 4 and maoa allele variation among olanzapine, risperidone, and paliperidone in schizophrenia. *Journal of clinical Psychopharmacology* 2012; **32**: 394–7.

69 Citrome L, Ota A, Nagamizu K, Perry P, Weiller E, Baker RA. The effect of brexpiprazole (opc-34712) and aripiprazole in adult patients with acute schizophrenia: Results from a randomized, exploratory study. *International clinical Psychopharmacology* 2016; **31**: 192–201.

70 Ciurezu T, Ionescu R, Nica Udangiu S *et al.* [Double-blind clinical study of hf 1854 (lx 100-129, clozapine or leponex) as compared with haloperidol]. *Neurologie et Psychiatrie* 1976; **14**: 29–34.

71 Clark ML, Ray TS, Paredes A *et al.* Chlorpromazine in women with chronic schizophrenia: The effect on cholesterol levels and cholesterol-behavior relationships. *Psychosomatic Medicine* 1967; **29**: 634–42.

72 Clark. Haloperidol versus chlorpromazine versus placebo: Psychopharmacology bulletin. 1969; **5**: 57–9.

73 Clark ML, Huber WK, Sakata K, Fowles DC, Serafetinides EA. Molindone in chronic schizophrenia. *clinical Pharmacology & Therapeutics* 1970; **11**: 680–8.

74 Clark ML, Huber WK, Charalampous KD, Serafetinides EA, Trousdale W, Colmore JP. Drug treatment in newly admitted schizophrenic patients. *Archives of General Psychiatry* 1971; **25**: 404–9.

75 Clark ML, Huber WK, Sullivan J, Wood F, Costiloe JP. Evaluation of loxapine succinate in chronic schizophrenia. *Diseases of the Nervous System* 1972; **33**: 783–91.

76 Clark ML, Paredes A, Costiloe JP, Wood F, Barrett A. Loxapine in newly admitted chronic schizophrenic patients. *Journal of clinical Pharmacology* 1975; **15**: 286–94.

77 Clark ML, Paredes A, Costiloe JP, Fulkerson FG, Wood F. Evaluation of two dose levels of loxapine succinate in chronic schizophrenia. *Diseases of the Nervous System* 1977; **38**: 7–10.

78 Clark ML, Paredes A, Costiloe JP, Wood F. Evaluation of butaclamol in chronic schizophrenic patients. *Journal of clinical Pharmacology* 1977; **17**: 529–36.

79 Claus A, Bollen J, Cuyper H de *et al.* Risperidone versus haloperidol in the treatment of chronic schizophrenic inpatients: A multicentre double-blind comparative study. *Acta Psychiatrica Scandinavica* 1992; **85**: 295–305.

80 Conley RR, Mahmoud R. A randomized double-blind study of risperidone and olanzapine in the treatment of schizophrenia or schizoaffective disorder.[Erratum appears in am j psychiatry 2001 oct;158(10): 1759]. *American Journal of Psychiatry* 2001; **158**: 765–74.

81 Cooper SJ, Tweed J, Raniwalla J, Butler A, Welch C. A placebo-controlled comparison of zotepine versus chlorpromazine in patients with acute exacerbation of schizophrenia. *Acta Psychiatrica Scandinavica* 2000; **101**: 218–25.

82 Copolov DL, Link CG, Kowalcyk B. A multicentre, double-blind, randomized comparison of quetiapine (ici 204,636, 'seroquel') and haloperidol in schizophrenia.[Erratum appears in psychol med 2000 jul;30(4): 991]. *Psychological Medicine* 2000; **30**: 95–105.

83 Correll CU, Skuban A, Ouyang J *et al.* Efficacy and safety of brexpiprazole for the treatment of acute schizophrenia: A 6-week randomized, double-blind, placebo-controlled trial. *American Journal of Psychiatry* 2015; **172**: 870–80.

84 Corrigan MH, Gallen CC, Bonura ML, Merchant KM, Sonepiprazole Study G. Effectiveness of the selective d4 antagonist sonepiprazole in schizophrenia: A placebo-controlled trial. *Biological Psychiatry* 2004; **55**: 445–51.

85 Corripio I, Catafau AM, Perez V *et al.* Striatal dopaminergic d2 receptor occupancy and clinical efficacy in psychosis exacerbation: A 123I-ibzm study with ziprasidone and haloperidol.[Erratum appears in prog neuropsychopharmacol biol psychiatry. 2006 aug 30;30(6):1186]. *Progress in Neuro-Psychopharmacology & Biological Psychiatry* 2005; **29**: 91–6.

86 Cosar B, Candansayar S, Taner E, Isik E. Comparison of efficacy of clozapine, sulpiride, chlorpromazine and haloperidol in chronic schizophrenic patients therapy: Journal of the european college of neuropsychopharmacology. 1999; **9**: S287.

87 Costa e Silva, J. A. Comparative double-blind study of amisulpride versus haloperidol in the treatment of acute psychotic states. In: Amisulpride. Paris: Expansion scientifique francaise, 1989: 93–104.

88 Crowley TJ, Hydinger-Macdonald M. Motility, parkinsonism, and prolactin with thiothixene and thioridazine. *Archives of General Psychiatry* 1981; **38**: 668–75.

89 Cutler AJ, Marcus RN, Hardy SA, O'Donnell A, Carson WH, McQuade RD. The efficacy and safety of lower doses of aripiprazole for the treatment of patients with acute exacerbation of schizophrenia. *Cns Spectrums* 2006; **11**: 691–702; quiz 719.

90 Cutler AJ, Kalali AH, Weiden PJ, Hamilton J, Wolfgang CD. Four-week, double-blind, placebo- and ziprasidone-controlled trial of iloperidone in patients with acute exacerbations of schizophrenia. *Journal of clinical Psychopharmacology* 2008; **28**: S20–8.

91 Cutler AJ, Tran-Johnson T, Kalali A, Astrom M, Brecher M, Meulien D. A failed 6-week,randomized, double-blind, placebo-controlled study of once-daily extended release quetiapine fumarate in patients with acute schizophrenia: Lessons learned. *Psychopharmacology Bulletin* 2010; **43**: 37–69.

92 Daniel DG, Zimbroff DL, Potkin SG, Reeves KR, Harrigan EP, Lakshminarayanan M. Ziprasidone 80 mg/day and 160 mg/day in the acute exacerbation of schizophrenia and schizoaffective disorder: A 6-week placebo-controlled trial. Ziprasidone study group. *Neuropsychopharmacology* 1999; **20**: 491–505.

93 Davidson M, Emsley R, Kramer M *et al.* Efficacy, safety and early response of paliperidone extended-release tablets (paliperidone er): Results of a 6-week, randomized, placebo-controlled study.[Erratum appears in schizophr res. 2007 nov;96(1-3):273-4]. *Schizophrenia Research* 2007; **93**: 117–30.

94 Boer MK de, Wiersma D, Bous J *et al.* A randomized open-label comparison of the impact of aripiprazole versus risperidone on sexual functioning (ras study). *Journal of clinical Psychopharmacology* 2011; **31**: 523–5.

95 Oliveira IR de, Elkis H, Gattaz WF *et al.* Aripiprazole for patients with schizophrenia and schizoaffective disorder: An open-label, randomized, study versus haloperidol. *Cns Spectrums* 2009; **14**: 93–102.

96 Dehnel LL, Vestre ND, Schiele BC. A controlled comparison of clopenthixol and perphenazine in a chronic schizophrenic population. *Current Therapeutic Research, clinical & Experimental* 1968; **10**: 169–76.

97 Jong J de. Efficacy of phenothiazines without antiparkinson drugs. *Diseases of the Nervous System* 1965; **26**: 702–4.

98 Delcker A, Schoon ML, Oczkowski B, Gaertner HJ. Amisulpride versus haloperidol in treatment of schizophrenic patients–results of a double-blind study. *Pharmacopsychiatry* 1990; **23**: 125–30.

99 d'Elia G, Perris C, Rapp W. Objective evaluation of eeg amplitude in chronic schizophrenic patients during a controlled trial of pimozide and trifluoperazine. *Acta Psychiatrica Scandinavica, Supplementum* 1974; **249**: 78–86.

100 DeMartinis NA. Results of a phase 2A proof-of-concept trial with a pde10a inhibitor in the treatment of acute exacerbation of schizophrenia. *Biological Psychiatry* 2012; **1)**: 17S–8S.

101 Denber HC, Turns D. Double blind comparison of thiothixene and trifluoperazine in acute schizophrenia. *Psychosomatics* 1972; **13**: 100–4.

102 Dieterle DM, Miller-Spahn F, Ackenheil M. Efficacy and tolerance of zotepine in a double-blind comparison with perazin in schizophrenics. [German] wirksamkeit und vertraglichkeit von zotepin im doppelblindvergleich mit perazin bei schizophrenen patienten. *Fortschritte der Neurologie Psychiatrie* 1991; **59**: 18–22.

103 Dolnak DR, Minn K, Wieneke M, Watson C, Espinoza S, Ra. Olanzapine versus haloperidol in the treatment of schizophrenia: 149th annual meeting of the american psychiatric association. New york, new york, usa. 4-9th may, 1996. 1996.

104 Dolnak R, Rapaport MH. A prospective, randomized, doubleblind study examining functioning in schizophrenic patients treated with olanzapine and risperidone: Schizophrenia research (abstracts of the viii international congress on schizophrenia research; 2001 april 28-may 2; british columbia, canada). 2001; **49**: 225–6.

105 Dossenbach M, Treuer T, Kryzhanovskaya L *et al.* Olanzapine versus chlorpromazine in the treatment of schizophrenia: A pooled analysis of four 6-week, randomized, open-label studies in the middle east and north africa. *Journal of clinical Psychopharmacology* 2007; **27**: 329–37.

106 Downing AM, Kinon BJ, Millen BA *et al.* A double-blind, placebo-controlled comparator study of ly2140023 monohydrate in patients with schizophrenia. *BMC Psychiatry* 2014; **14**: 351.

107 Dube KC, Kumar N. Loxapine succinate: A comparative study with chlorpromazine. *Current Therapeutic Research, clinical & Experimental* 1976; **19**: 653–60.

108 HGFH K. Data on file. *Data supplied to the Cochrane Schizophrenia Group* 1999.

109 Durgam S, Starace A, Li D *et al.* An evaluation of the safety and efficacy of cariprazine in patients with acute exacerbation of schizophrenia: A phase ii, randomized clinical trial. *Schizophrenia Research* 2014; **152**: 450–7.

110 Dutta T, Bhowmick S, Mitra M, Nath S, Chatterjee RN, Bhattacharjee S. A comparative study of efficacy and safety of three atypical antipsychotic agents: Asenapine, ziprasidone and clozapine in indian schizophrenic patients: A randomized controlled trial. *Indian Journal of Pharmacology* 2014; **46**: S9.

111 Ebrinc S, Semiz UB, Basoglu C *et al.* Efficacy and safety of amisulpride treatment in schizophrenia: Comparison with haloperidol. [Turkish] sizofrenili hastalarin tedavisinde amisulpridin etkililigi ve emniyeti: Haloperidol ile bir karsilastirma. *Klinik Psikofarmakoloji Bulteni* 2004; **14**: 143–9.

112 Edwards JG, Alexander JR, Alexander MS, Gordon A, Zutchi T. Controlled trial of sulpiride in chronic schizophrenic patients. *British Journal of Psychiatry* 1980; **137**: 522–9.

113 Egan MF, Zhao X, Smith A *et al.* Randomized controlled study of the t-type calcium channel antagonist mk-8998 for the treatment of acute psychosis in patients with schizophrenia. *Human Psychopharmacology* 2013; **28**: 124–33.

114 Ehmann TS, Delva NJ, Beninger RJ. Flupenthixol in chronic schizophrenic inpatients: A controlled comparison with haloperidol. *Journal of clinical Psychopharmacology* 1987; **7**: 173–5.

115 Ehrlich S, Leopold K, Merle JV *et al.* Trajectories of agouti-related protein and leptin levels during antipsychotic-associated weight gain in patients with schizophrenia. *Journal of clinical Psychopharmacology* 2012; **32**: 767–72.

116 Engelhardt DM, Margolis RA, Rudorfer L, Paley HM. Physician bias and the double-blind. *Archives of General Psychiatry* 1969; **20**: 315–20.

117 McDonnell D, Potkin SG, Simmons A, Jiang Y, DiPetrillo L, Silverman B. A phase 3 study to determine the antipsychotic efficacy and safety of alks 3831 in adult patients with acute exacerbation of schizophrenia. *Schizophrenia Bulletin* 2018; **44 (Supplement 1)**: S312.

118 Erlandsen C. Trial of a new neuroleptic drug, leponex (clozapine) in long-standing schizophrenia. [Norwegian]. *Nordisk Psykiatrisk Tidsskrift* 1981; **35**: 248–53.

119 Escobar JI, Mann JJ, Keller J, Wilkins J, Mason B, Mills MJ. Comparison of injectable molindone and haloperidol followed by oral dosage forms in acutely ill schizophrenics. *Journal of clinical Psychiatry* 1985; **46**: 15–9.

120 Evans JR, Rodnick EH, Goldstein MJ, Judd LL. Premorbid adjustment, phenothiazine treatment, and remission in acute schizophrenics. *Archives of General Psychiatry* 1972; **27**: 486–90.

121 Fabre Jr LF, Arvanitis L, Pultz J, Jones VM, Malick JB, Slotnick VB. ICI 2044,636, a novel, atypical antipsychotic: Early indication of safety and efficacy in patients with chronic and subchronic schizophrenia. *clinical Therapeutics* 1995; **17**: 366–78.

122 Fakra E, Khalfa S, Da Fonseca D *et al.* Effect of risperidone versus haloperidol on emotional responding in schizophrenic patients. *Psychopharmacology* 2008; **200**: 261–72.

123 Faustman WO, Ringo DL, Lauriello J *et al.* Effects if seroquel (ici 204 636) on platelet serotonin-2 binding in schizophrenia conference abstract: Schizophrenia research (the vth international congress on schizophrenia research, wormsprings, va usa. 6th-12th april, 1995.). 1995; **1, 2**.

124 Fischer Cornelssen K, Ferner U, Steiner H. Multicenter trial of psychotropic drugs (multihospital trial). [German] multifokale psychopharmakaprufung ('multihospital trial'). *Arzneimittel-Forschung/Drug Research* 1974; **24**: 1706–24.

125 Fleischhacker WW, Barnas C, Stuppack CH, Unterweger B, Miller C, Hinterhuber H. Zotepine vs. Haloperidol in paranoid schizophrenia: A double-blind trial. *Psychopharmacology Bulletin* 1989; **25**: 97–100.

126 Fleischhacker WW, McQuade RD, Marcus RN, Archibald D, Swanink R, Carson WH. A double-blind, randomized comparative study of aripiprazole and olanzapine in patients with schizophrenia. *Biological Psychiatry* 2009; **65**: 510–7.

127 Fleming BG, Spencer AM, Whitelaw EM. A controlled comparative investigation of the effects of promazine, chlorpromazine, and a placebo in chronic psychosis. *J Ment Sci* 1959; **105**: 349–58.

128 Fleming B, Currie J. Investigation of a new compound, b.w.203, and of chlorpromazine in the treatment of psychosis: Journal-of-mental-science-(br-j-psychiatry-from-1963). 1968; **104**: 749–57.

129 Freeman H, Frederick AN. Comparison of trifluoperazine and molindone in chronic schizophrenic patients. *Current Therapeutic Research, clinical & Experimental* 1969; **11**: 670–6.

130 Fruensgaard K, Wollenberg J, Hansen KM, Fensbo C, Sihm F. Loxapine versus perphenazine in psychotic patients. A double-blind, randomized, multicentre trial. *Current Medical Research & Opinion* 1978; **5**: 601–7.

131 Fruensgaard K, Korsgaard S, Jorgensen H, Jensen K. Loxapine versus haloperidol parenterally in acute psychosis with agitation. A double-blind study. *Acta Psychiatrica Scandinavica* 1977; **56**: 256–64.

132 Gallant DM, Bishop MP, Steele CA, NOBLIN CD. The relationship between serotonin antagonism and tranquilizing activity. *American Journal of Psychiatry* 1963; **119**: 882.

133 Gallant DM, Bishop MP, Timmons E, Gould AR. Thiothixene (p-4657B): A controlled evaluation in chronic schizophrenic patients. *Current Therapeutic Research, clinical & Experimental* 1966; **8**: 153–8.

134 Gallant DM, Bishop M, Guerrero-Figueroa R. Effects of two butyrophenone compounds on acute schizophrenic patients: Speculation on the neurophysiologic sites of action. *International Journal of Neuropsychiatry* 1967; **3**: Suppl 1:53–60.

135 Gallant DM, Bishop MP. Molindone: A controlled evaluation in chronic schizophrenic patients. *Current Therapeutic Research, clinical & Experimental* 1968; **10**: 441–7.

136 Garcia E, Robert M, Peris F, Nakamura H, Sato N, Terazawa Y. The efficacy and safety of blonanserin compared with haloperidol in acute-phase schizophrenia: A randomized, double-blind, placebo-controlled, multicentre study. *CNS Drugs* 2009; **23**: 615–25.

137 Garry JW, Leonard TJ. Haloperidol: A controlled trial in chronic schizophrenia. *J Ment Sci* 1962; **108**: 105–7.

138 Gattaz WF, Diehl A, Geuppert MS *et al.* Olanzapine versus flupenthixol in the treatment of inpatients with schizophrenia: A randomized double-blind trial. *Pharmacopsychiatry* 2004; **37**: 279–85.

139 Geffen Y, Keefe R, Rabinowitz J, Anand R, Davidson M. Bl-1020, a new gamma-aminobutyric acid-enhanced antipsychotic: Results of 6-week, randomized, double-blind, controlled, efficacy and safety study. *Journal of clinical Psychiatry* 2012; **73**: e1168–74.

140 Gelenberg AJ, Doller JC. Clozapine versus chlorpromazine for the treatment of schizophrenia: Preliminary results from a double-blind study. *Journal of clinical Psychiatry* 1979; **40**: 238–40.

141 Gerlach J, Koppelhus P, Helweg E, Monrad A. Clozapine and haloperidol in a single-blind cross-over trial: Therapeutic and biochemical aspects in the treatment of schizophrenia. *Acta Psychiatrica Scandinavica* 1974; **50**: 410–24.

142 Gerlach J, Thorsen K, Fog R. Extrapyramidal reactions and amine metabolites in cerebrospinal fluid during haloperidol and clozapine treatment of schizophrenic patients. *Psychopharmacologia* 1975; **40**: 341–50.

143 Ghaleiha A, Honarbakhsh N, Boroumand MA *et al.* Correlation of adenosinergic activity with superior efficacy of clozapine for treatment of chronic schizophrenia: A double blind randomised trial. *Human Psychopharmacology* 2011; **26**: 120–4.

144 Goff DC, Posever T, Herz L *et al.* An exploratory haloperidol-controlled dose-finding study of ziprasidone in hospitalized patients with schizophrenia or schizoaffective disorder. *Journal of clinical Psychopharmacology* 1998; **18**: 296–304.

145 Goldberg SC, Frosch WA, Drossman AK, Schooler NR, Johnson GF. Prediction of response to phenothiazines in schizophrenia. A crossvalidation study. *Archives of General Psychiatry* 1972; **26**: 367–73.

146 Goldstein BJ, Clyde DJ. Haloperidol in controlling the symptoms of acute psychoses. II. A double-blind evaluation of haloperidol and trifluoperazine. *Current Therapeutic Research, clinical & Experimental* 1966; **8**: 236–40.

147 Goldstein BJ, Brauzer B, Clyde DJ, Caldwell JM. The differential prediction of response to two anti-psychotic drugs. *Psychosomatics* 1969; **10**: 193–7.

148 Gowardman M, Barrer B, Brown RA. Pimozide (r6238) in chronic schizophrenia: Double blind trial. *New Zealand Medical Journal* 1973; **78**: 487–91.

149 Granacher RP, Ruth DD. A comparison of thioridazine (mellaril) and thiothixene (navane) in the treatment of hospitalized psychotic patients. *Current Therapeutic Research* 1982; **31**: 692–705.

150 Grootens KP, van Veelen NM, Peuskens J *et al.* Ziprasidone vs olanzapine in recent-onset schizophrenia and schizoaffective disorder: Results of an 8-week double-blind randomized controlled trial. *Schizophrenia Bulletin* 2011; **37**: 352–61.

151 Guirguis E, Voineskos G, Gray J, Schlieman E. Clozapine (leponex) vs chlorpromazine (largactil) in acute schizophrenia: (A double-blind controlled study). *Current Therapeutic Research* 1977; **21**: 707–19.

152 Gupta S, Gupta P, Sharma M. Comparative profile of atypical antipsychotics in patients of schizophrenia. *Indian journal of psychiatry Conference: 70th annual national conference of indian psychiatric society, ANCIPS 2018 India* 2018; **60**: S75.

153 Guz H, Ozkan A. Comparison of risperidone and olanzapine in schizophrenia. [Turkish] sizofrenide risperidon ile olanzapinin karsilastirilmasi. *Ondokuz Mayis Universitesi Tip Dergisi* 2002; **19**: 51–7.

154 Haas S, Beckmann H. Pimozide versus haloperidol in acute schizophrenia. A double blind controlled study. *Pharmacopsychiatria* 1982; **15**: 70–4.

155 Hadlik J, Svestka J, Nahunek K, Rodova A. [Controlled studies with thiothixene and perphenazine in schizophrenic psychoses]. *Activitas Nervosa Superior* 1970; **12**: 60–1.

156 Hale Jean-Michel Azorin Siegfried Kasper Wolfgang Maier Erkka Syvalahti Michael Van Der Burght Mogens Sloth-Nielsen Allan Wehnert, A. Sertindole is associated with a low level of extrapyramidal symptoms in schizophrenic patients: Results of a phase iii trial. *International Journal of Psychiatry in clinical Practice* 2000; **4**: 47–54.

157 Hall RADDJ. A study of chlorpromazine: Methodology and results with chronic semi-disturbed schizophrenics: Journal of nervous and mental disease. 1955; **122**: 301–14.

158 Hall WB, Vestre ND, Schiele BC, Zimmermann R. A controlled comparison of haloperidol and fluphenazine in chronic treatment-resistant schizophrenics. *Diseases of the Nervous System* 1968; **29**: 405–8.

159 Harnryd C, Bjerkenstedt L, Bjork K *et al.* clinical evaluation of sulpiride in schizophrenic patients–a double-blind comparison with chlorpromazine. *Acta Psychiatrica Scandinavica, Supplementum* 1984; **311**: 7–30.

160 Hatta K, Sato K, Hamakawa H *et al.* Effectiveness of second-generation antipsychotics with acute-phase schizophrenia. *Schizophrenia Research* 2009; **113**: 49–55.

161 Hatta K, Takebayashi H, Sudo Y *et al.* The possibility that requiring high-dose olanzapine cannot be explained by pharmacokinetics in the treatment of acute-phase schizophrenia. *Psychiatry Research* 2013; **210**: 396–401.

162 Haug JO. [A controlled clinical trial of thioridazine (melleril); a new phenothiazine derivative]. *Tidsskrift for Den Norske Laegeforening* 1959; **79**: 317–21 passim.

163 Heikkila L, Laitinen J, Vartiainen H. Cis(Z)-clopenthixol and haloperidol in chronic schizophrenic patients–a double-blind clinical multicentre investigation. *Acta Psychiatrica Scandinavica, Supplementum* 1981; **294**: 30–8.

164 Heikkila L, Eliander H, Vartiainen H, Turunen M, Pedersen V. Zuclopenthixol and haloperidol in patients with acute psychotic states. A double-blind, multi-centre study. *Current Medical Research & Opinion* 1992; **12**: 594–603.

165 Heikkinen H, Outakoski J, Merilainen V, Tuomi A, Huttunen MO. Molindone and weight loss. *Journal of clinical Psychiatry* 1993; **54**: 160–1.

166 Heinrich K, Klieser E, Lehmann E, Kinzler E, Hruschka H. Risperidone versus clozapine in the treatment of schizophrenic patients with acute symptoms: A double blind, randomized trial. *Progress in Neuro-Psychopharmacology & Biological Psychiatry* 1994; **18**: 129–37.

167 041-021 SH. A multicenter, randomized, double-blind, fixed-dose, 6-week trial of the efficacy and safety of asenapine compared with placebo using olanzapine postive control in subjects with an acute exacerbation of schizophrenia. *Center for drug evaluation and researchApplication number 22-117Medical review(s)http://wwwfgagov* 2009.

168 041-022 SH. A multicenter, randomized, double-blind, flexible-dose, 6-week trial of the efficacy and safety of asenapine compared with placebo using olanzapine postive control in subjects with an acute exacerbation of schizophrenia. *Center for drug evaluation and researchApplication number 22-117Medical review(s)http://wwwfgagov* 2009.

169 Herrera J, Costa J, Sramek J *et al.* The efficacy of sustained-release thioridazine in the treatment of schizophrenic inpatients. *Current Therapeutic Research - clinical and Experimental* 1990; **48**: 1006–11.

170 Hogan TP, Awad AG. Subjective response to neuroleptics and outcome in schizophrenia: A re-examination comparing two measures. *Psychological Medicine* 1992; **22**: 347–52.

171 Honigfeld G, Patin J, Singer J. Clozapine: Antipsychotic activity in treatment-resistant schizophrenics. *Advances in Therapy* 1984; **1**: 77–97.

172 Howell RJ, Brown, H. M., Jr., Beaghler HE. A comparison of fluphenazine, trifluoperazine and a placebo in the context of an active treatment unit. *J Nerv Ment Dis* 1961; **132**: 522–30.

173 Hoyberg OJ, Fensbo C, Remvig J, Lingjaerde O, Sloth-Nielsen M, Salvesen I. Risperidone versus perphenazine in the treatment of chronic schizophrenic patients with acute exacerbations. *Acta Psychiatrica Scandinavica* 1993; **88**: 395–402.

174 Huttunen MO, Piepponen T, Rantanen H, Larmo I, Nyholm R, Raitasuo V. Risperidone versus zuclopenthixol in the treatment of acute schizophrenic episodes: A double-blind parallel-group trial. *Acta Psychiatrica Scandinavica* 1995; **91**: 271–7.

175 Hwang TJ, Lin SK, Lin HN. Efficacy and safety of zotepine for the treatment of taiwanese schizophrenic patients: A double-blind comparison with haloperidol. *Journal of the Formosan Medical Association* 2001; **100**: 811–6.

176 Hwang TJ, Lee SM, Sun HJ *et al.* Amisulpride versus risperidone in the treatment of schizophrenic patients: A double-blind pilot study in taiwan. *Journal of the Formosan Medical Association* 2003; **102**: 30–6.

177 Hwang TJ, Chan HY, Lin WW *et al.* Aripiprazole versus risperidone in the treatment of acutely relapsed patients with schizophrenia in taiwan: A randomized controlled trial. *European Neuropsychopharmacology* 2005; **15**: S498.

178 Imai H, Nakamura M, Fukui Y *et al.* Comparison of efficacy of zotepine and perphenazine in schizophrenia by double-blind, controlled study. *JpnJNeuropsyhophar* 1980; **2**: 285–99.

179 Ingole S, Belorkar NR, Waradkar P, Shrivastava M. Comparison of effects of olanzapine and risperidone on body mass index and blood sugar level in schizophrenic patients. *Indian Journal of Physiology & Pharmacology* 2009; **53**: 47–54.

180 Ishigooka J, Inada T, Miura S. Olanzapine versus haloperidol in the treatment of patients with chronic schizophrenia: Results of the japan multicenter, double-blind olanzapine trial. *Psychiatry & clinical Neurosciences* 2001; **55**: 403–14.

181 Ishigooka J, Iwashita S, Tadori Y. Efficacy and safety of brexpiprazole for the treatment of acute schizophrenia in japan: A 6-week, randomized, double-blind, placebo-controlled study. *Psychiatry Clin Neurosci* 2018; **72**: 692–700.

182 Itil TM, Polvan N, Ucok A, Eper E, Guven F, Hsu W. Comparison of the clinical and electroencephalographical effects of molindone and trifluoperazine in acute schizophrenic patients. *Behavioral Neuropsychiatry* 1971; **3**: 25–32.

183 Itoh H, Miura S, Yagi G, Ogita K, Ohtsuka N, Koga Y. Comparison of clinical effects of penfluridol, a long-acting oral neuroleptic, and perphenazine in schizophrenia using double-blind technique: Rinsho hyoka. 1976; **4**.

184 Itoh H, Miura S, Yagi G, Sakurai S, Ohtsuka N. Some methodological considerations for the clinical evaluation of neuroleptics–comparative effects of clozapine and haloperidol on schizophrenics. *Folia Psychiatrica et Neurologica Japonica* 1977; **31**: 17–24.

185 Janicak PG, Keck, P. E., Jr., Davis JM *et al.* A double-blind, randomized, prospective evaluation of the efficacy and safety of risperidone versus haloperidol in the treatment of schizoaffective disorder. *Journal of clinical Psychopharmacology* 2001; **21**: 360–8.

186 Jann MW, Crabtree BL, Pitts WM, Lam YW, Carter JG. Plasma alpha-one acid glycoprotein and haloperidol concentrations in schizophrenic patients. *Neuropsychobiology* 1997; **36**: 32–6.

187 Hirayasu Y, Tomioka M, Iizumi M, Kikuchi H. A double-blind, placebo-controlled, comparative study of paliperidone extended release (er) tablets in patients with schizophrenia. *JpnJClinPsychopharmacol* 2010; **13**: 2077–103.

188 Jindal KC, Singh GP, Munjal V. Aripiprazole versus olanzapine in the treatment of schizophrenia: A clinical study from india. *International Journal of Psychiatry in clinical Practice* 2013; **17**: 21–9.

189 Canuso C, Lindenmayer JP, Carothers J, Kosik-Gonzalez C, Turkoz I, Schooler N. Randomized, double-blind, placebo-controlled study of two dose ranges of paliperidone er in the treatment of subjects with schizoaffective disorder. *Biological Psychiatry* 2009; **1)**: 213S–4S.

190 Canuso CM, Schooler N, Carothers J *et al.* Paliperidone extended-release in schizoaffective disorder: A randomized, controlled study comparing a flexible dose with placebo in patients treated with and without antidepressants and/or mood stabilizers. *Journal of clinical Psychopharmacology* 2010; **30**: 487–95.

191 Coppola D, Melkote R, Lannie C *et al.* Efficacy and safety of paliperidone extended release 1.5 mg/day-a double-blind, placebo- and active-controlled, study in the treatment of patients with schizophrenia. *Psychopharmacology Bulletin* 2011; **44**.

192 Johnstone EC, Crow TJ, Frith CD, Carney MW, Price JS. Mechanism of the antipsychotic effect in the treatment of acute schizophrenia. *Lancet* 1978; **1**: 848–51.

193 Judd LL, Goldstein MJ, Rodnick EH. Phenothiazine effects in good premorbid schizophrenics divided into paranoid nonparanoid status. *Archives of General Psychiatry* 1973; **29**: 207–11.

194 Kahn RS, Schulz SC, Palazov VD *et al.* Efficacy and tolerability of once-daily extended release quetiapine fumarate in acute schizophrenia: A randomized, double-blind, placebo-controlled study. *Journal of clinical Psychiatry* 2007; **68**: 832–42.

195 Kane JM, Carson WH, Saha AR *et al.* Efficacy and safety of aripiprazole and haloperidol versus placebo in patients with schizophrenia and schizoaffective disorder. *Journal of clinical Psychiatry* 2002; **63**: 763–71.

196 Kane J, Canas F, Kramer M *et al.* Treatment of schizophrenia with paliperidone extended-release tablets: A 6-week placebo-controlled trial. *Schizophrenia Research* 2007; **90**: 147–61.

197 Kane JM, Osuntokun O, Kryzhanovskaya LA *et al.* A 28-week, randomized, double-blind study of olanzapine versus aripiprazole in the treatment of schizophrenia. *Journal of clinical Psychiatry* 2009; **70**: 572–81.

198 Kane JM, Cohen M, Zhao J, Alphs L, Panagides J. Efficacy and safety of asenapine in a placebo- and haloperidol-controlled trial in patients with acute exacerbation of schizophrenia. *Journal of clinical Psychopharmacology* 2010; **30**: 106–15.

199 Kane JM, Skuban A, Ouyang J *et al.* A multicenter, randomized, double-blind, controlled phase 3 trial of fixed-dose brexpiprazole for the treatment of adults with acute schizophrenia. *Schizophrenia Research* 2015; **164**: 127–35.

200 Kaushal J, Bhutani G, Gupta R. Comparison of fasting blood sugar and serum lipid profile changes after treatment with atypical antipsychotics olanzapine and risperidone. *Singapore Medical Journal* 2012; **53**: 488–92.

201 Keck, P., Jr., Buffenstein A, Ferguson J *et al.* Ziprasidone 40 and 120 mg/day in the acute exacerbation of schizophrenia and schizoaffective disorder: A 4-week placebo-controlled trial. *Psychopharmacology* 1998; **140**: 173–84.

202 Kenway AK, Masheter HC. Pimozide compared with fluphenazine in schizophrenia. *British Journal of clinical Practice* 1971; **25**: 69–72.

203 Khorana AB, Patel Y. Comparative short-term evaluation of penfluridol and trifluoperazine in chronic schizophrenia. *Indian Journal of Physiology & Pharmacology* 1988; **32**: 293–8.

204 Kiloh LG, Williams SE, Grant DA, Whetton PS. A double-blind comparative trial of loxapine and trifluoperazine in acute and chronic schizophrenic patients. *Journal of International Medical Research* 1976; **4**: 441–8.

205 King PDWW. Comparison of proclorperazine and chlorpromazine in hospitalized chronic schizophrenics: American journal of psychiatry. 1959; **115**: 1026–7.

206 Kingstone E, Kolivakis T, Kossatz I. Double blind study of clopenthixol and chlorpromazine in acute hospitalized schizophrenics. *Internationale Zeitschrift fur Klinische Pharmakologie, Therapie und Toxikologie* 1970; **3**: 41–5.

207 Kinon BJ, Zhang L, Millen BA *et al.* A multicenter, inpatient, phase 2, double-blind, placebo-controlled dose-ranging study of ly2140023 monohydrate in patients with dsm-iv schizophrenia. *Journal of clinical Psychopharmacology* 2011; **31**: 349–55.

208 Klein DF, Rosen B. Premorbid asocial adjustment and response to phenothiazine treatment among schizophrenic inpatients. *Archives of General Psychiatry* 1973; **29**: 480–5.

209 Klein HE, Dieterle D, Rüther E, Eben E, Nedopil N, Hippius H. A double blind comparison of amisulpride vs. Haloperidol in acute schizophrenic patients. In: Pichot P, Berner P, Wolf R, Thau K, eds. Psychiatry the state of the art. Boston, MA: Springer US, 1985: 687–91.

210 Klieser E, Lehmann E. Experimental examination of trazodone. *clinical Neuropharmacology* 1989; **12 Suppl 1**: S18–24.

211 Klieser E, Schnöll H. Klinisch-pharmakologische studien zur behandlung schizophrener minussymptomatik. *Neuere Ansätze zur Diagnostik und Therapie schizophrener Minussymptomatik* 1990;: 217–22.

212 Klieser E, Lehmann E, Tegeler J. [Double-blind comparison of 3 x 75 mg zotepine und 3 x 4 mg haloperidol in acute schizophrenic patients]. *Fortschritte der Neurologie-Psychiatrie* 1991; **59 Suppl 1**: 14–7.

213 Klieser E, Strauss WH, Lemmer W. The tolerability and efficacy of the atypical neuroleptic remoxipride compared with clozapine and haloperidol in acute schizophrenia. *Acta Psychiatrica Scandinavica, Supplementum* 1994; **380**: 68–73.

214 Klimke A, Klieser E, Lehmann E, Miele L. Initial improvement as a criterion for drug choice in acute schizophrenia. *Pharmacopsychiatry* 1993; **26**: 25–9.

215 Kluge M, Schuld A, Himmerich H *et al.* Clozapine and olanzapine are associated with food craving and binge eating: Results from a randomized double-blind study. *Journal of clinical Psychopharmacology* 2007; **27**: 662–6.

216 Knegtering R, Castelein S, Bous H *et al.* A randomized open-label study of the impact of quetiapine versus risperidone on sexual functioning. *Journal of clinical Psychopharmacology* 2004; **24**: 56–61.

217 Knegtering H, Boks M, Blijd C, Castelein S, Van den Bosch, R. J., Wiersma D. A randomized open-label comparison of the impact of olanzapine versus risperidone on sexual functioning. *Journal of Sex & Marital Therapy* 2006; **32**: 315–26.

218 Kordas SK, Kazamias NG, Georgas JG, Papadokostakis JG. Clopenthixol: A controlled trial in chronic hospitalized schizophrenic patients. *British Journal of Psychiatry* 1968; **114**: 833–6.

219 Kramer M, Roth T, Salis PJ. Relative efficacy and safety of loxapine succinate (loxitane) and thioridazine hydrochloride (mellaril) in the treatment of acute schizophrenia. *Current Therapeutic Research - clinical and Experimental* 1978; **23**: 619–31.

220 Kurland AA, Hanlon TE, Tatom MH, Ota KY, Simopoulos AM. The comparative effectiveness of six phenothiazine compounds, phenobarbital and intert placebo in the treatment of acutely ill patients: Global measures of severity of illness: Journal of nervous and mental disease. 1961; **133**: 1–18.

221 Lahti AC, Weiler MA, Holcomb HH, Tamminga CA, Cropsey KL. Modulation of limbic circuitry predicts treatment response to antipsychotic medication: A functional imaging study in schizophrenia. *Neuropsychopharmacology* 2009; **34**: 2675–90.

222 Lamure M, Toumi M, Chabannes JP, Dansette GY, Benyaya J, Hansen K. Zuclopenthixol versus haloperidol: An observational randomised pharmacoeconomic evaluation of patients with chronic schizophrenia exhibiting acute psychosis. *International Journal of Psychiatry in clinical Practice* 2003; **7**: 177–85.

223 Lemmer W, Klieser E, Klimke A. Experimental comparison of the efficacy of the dopamine autoreceptor agonist pramipexole versus haloperidol and placebo in acute schizophrenics: Pharmacopsychiatry. 1993; **26**: 176.

224 Lemperiere T, Lepine JP, Rouillon F, Feline A, Ferrand I, Ades J. [Evolution of depressive and psychotic symptomatology in hospitalised schizophrenic patients treated with neuroleptics (controlled study: Haloperidol vs chlorpromazine)]: Encephale. 1985; **11**: 279.

225 Leon CA, Estrada H. Therapeutic effects of clozapine on psychotic symptoms. (clinical evaluation using the double blind method). [Spanish] efectos terapeuticos de la clozapina (1) sobre los sintomas de psicosis. (Evaluacion clinica utilizando el metodo doble ciego). *RevColombPsiquiatr* 1974; **3**: 309–20.

226 Levita E. Effects of chlorpromazine and promazine on perseveration. *Journal of General Psychology* 1961; **65**: 181–7.

227 Li Y, Li H, Liu Y, Yan X, Yue Y, Qian M. Comparison of quetiapine and risperidone in chinese han patients with schizophrenia: Results of a single-blind, randomized study. *Current Medical Research & Opinion* 2012; **28**: 1725–32.

228 Lieberman JA, Stroup TS, McEvoy JP *et al.* Effectiveness of antipsychotic drugs in patients with chronic schizophrenia.[Erratum appears in n engl j med. 2010 sep 9;363(11): 1092-3]. *New England Journal of Medicine* 2005; **353**: 1209–23.

229 Lieberman JA, Davis RE, Correll CU *et al.* ITI-007 for the treatment of schizophrenia: A 4-week randomized, double-blind, controlled trial. *Biol Psychiatry* 2015. DOI:10.1016/j.biopsych.2015.08.026.

230 Liemburg E, Aleman A, Bous J, Hollander K, Knegtering H. An open randomized pilot trial on the differential effects of aripiprazole versus risperidone on anhedonia and subjective well-being. *Pharmacopsychiatry* 2011; **44**: 109–13.

231 Lin CC, Chiu HJ, Chen JY *et al.* Switching from clozapine to zotepine in patients with schizophrenia: A 12-week prospective, randomized, rater blind, and parallel study. *Journal of clinical Psychopharmacology* 2013; **33**: 211–4.

232 Lindenmayer JP, Brown D, Liu S, Brecher M, Meulien D. The efficacy and tolerability of once-daily extended release quetiapine fumarate in hospitalized patients with acute schizophrenia: A 6-week randomized, double-blind, placebo-controlled study. *Psychopharmacology Bulletin* 2008; **41**: 11–35.

233 Litman RE, Smith MA, Doherty JJ *et al.* AZD8529, a positive allosteric modulator at the mGluR2 receptor, does not improve symptoms in schizophrenia: A proof of principle study. *Abstracts of the 4th Biennial Schizophrenia International Research Conference* 2016; **172**: 152–7.

234 Litman RE, Smith MA, Desai DG, Simpson T, Sweitzer D, Kanes SJ. The selective neurokinin 3 antagonist azd2624 does not improve symptoms or cognition in schizophrenia: A proof-of-principle study. *Journal of clinical Psychopharmacology* 2014; **34**: 199–204.

235 Little JC. A double-blind controlled comparison of the effects of chlorpromazine, barbiturate and a placebo in 142 chronic psychotic in-patients. *Journal of Mental Science* 1958; **104**: 334–49.

236 Liu SK, Chen WJ, Chang CJ, Lin HN. Effects of atypical neuroleptics on sustained attention deficits in schizophrenia: A trial of risperidone versus haloperidol. *Neuropsychopharmacology* 2000; **22**: 311–9.

237 Loebel A, Silva R, Goldman R *et al.* Lurasidone dose escalation in early nonresponding patients with schizophrenia: A randomized, placebo-controlled study. *J Clin Psychiatry* 2016. DOI:10.4088/JCP.16m10698.

238 Loza N, El-Dosoky AM, Okasha TA *et al.* Olanzapine compared to chlorpromazine in acute schizophrenia: Journal of the european college of neuropsychopharmacology. 1999; **9**: S291.

239 Loza B, Czernikiewicz A, Roszkowska A, Szulc A. Atypical antipsychotics: The prosocial capacity. Double-blind, randomized, prospective study of olanzapine and risperidone treatment of schizophrenia: Cognitive, awareness and quality of life report. *International Journal of Neuropsychopharmacology* 2006; **9**: S271.

240 Lublin H, Gerlach J, Hagert U *et al.* Zuclopenthixol, a combined dopamine d1/d2 antagonist, versus haloperidol, a dopamine d2 antagonist, in tardive dyskinesia. *European Neuropsychopharmacology* 1991; **1**: 541–8.

241 Luckey WT, Schiele BC. A comparison of haloperidol and trifluoperazine. (A double-blind, controlled study on chronic schizophrenic outpatients). *Diseases of the Nervous System* 1967; **28**: 181–6.

242 Kwon JS, Mittoux A, Hwang JY, Ong A, Cai Zj, Su TP. The efficacy and safety of 12 weeks of treatment with sertindole or olanzapine in patients with chronic schizophrenia who did not respond successfully to their previous treatments: A randomized, double-blind, parallel-group, flexible-dose study. *International clinical Psychopharmacology* 2012; **27**: 326–35.

243 Maat A, Cahn W, Gijsman HJ, Hovens JE, Kahn RS, Aleman A. Open, randomized trial of the effects of aripiprazole versus risperidone on social cognition in schizophrenia. *European Neuropsychopharmacology* 2014; **24**: 575–84.

244 Mahadevan K, Gadhvi HM, Suri AK *et al.* A multicentre comparison of oral zuclopenthixol dihydrochloride and oral sulpiride in the treatment of acute schizophrenia: British journal of clinical research. 1991; **2**: 13–20.

245 Mahal AS, Ramu NG, Chaturvedi DD. Double blind controlled study of brahmyadiyoga and tagara in the management of various types of unmada (schizophrenia). *Indian Journal of Psychiatry* 1976; **18**: 283–92.

246 Marder SR, Meibach RC. Risperidone in the treatment of schizophrenia. *American Journal of Psychiatry* 1994; **151**: 825–35.

247 Marder SR, Kramer M, Ford L *et al.* Efficacy and safety of paliperidone extended-release tablets: Results of a 6-week, randomized, placebo-controlled study. *Biological Psychiatry* 2007; **62**: 1363–70.

248 Martin S, Ljo H, Peuskens J *et al.* A double-blind, randomised comparative trial of amisulpride versus olanzapine in the treatment of schizophrenia: Short-term results at two months. *Current Medical Research & Opinion* 2002; **18**: 355–62.

249 McCue RE, Waheed R, Urcuyo L *et al.* Comparative effectiveness of second-generation antipsychotics and haloperidol in acute schizophrenia. *British Journal of Psychiatry* 2006; **189**: 433–40.

250 McEvoy JP, Daniel DG, Carson, W. H., Jr., McQuade RD, Marcus RN. A randomized, double-blind, placebo-controlled, study of the efficacy and safety of aripiprazole 10, 15 or 20 mg/day for the treatment of patients with acute exacerbations of schizophrenia. *Journal of Psychiatric Research* 2007; **41**: 895–905.

251 McInnes EJ, Walker F, Snelling E. The observer interaction variable in evaluation of socialising properties of pimozide (orap). *New Zealand Medical Journal* 1978; **87**: 170–2.

252 McQuade RD, Stock E, Marcus R *et al.* A comparison of weight change during treatment with olanzapine or aripiprazole: Results from a randomized, double-blind study. *Journal of clinical Psychiatry* 2004; **65 Suppl 18**: 47–56.

253 Meltzer HY, Arvanitis L, Bauer D, Rein W, Meta-Trial Study G. Placebo-controlled evaluation of four novel compounds for the treatment of schizophrenia and schizoaffective disorder. *American Journal of Psychiatry* 2004; **161**: 975–84.

254 Meltzer HYBLMHJYPPSNA. A randomized, double-blind, placebo-controlled efficacy and safety study of bifeprunox as treatment for patients with acutely exacerbated schizophrenia: 11th international congress on schizophrenia research; 2007 mar 28-apr 1, colorado springs, colorado, usa. 2007.

255 Menon MS, Ramachandran V. A controlled clinical trial of trifluperidol on a group of chronic schizophrenic patients. *Current Therapeutic Research, clinical & Experimental* 1972; **14**: 17–21.

256 Mesotten F. Risperidone versus haloperidol in the treatment of chronic psychotic patients: A multicentre double-blind study. *unpublished clinical report* 1991.

257 Meyer-Lindenberg A, Gruppe H, Bauer U, Lis S, Krieger S, Gallhofer B. Improvement of cognitive function in schizophrenic patients receiving clozapine or zotepine: Results from a double-blind study. *Pharmacopsychiatry* 1997; **30**: 35–42.

258 Mezquita Blanco J, Cubillo Sanchez J, Aizpiri Diaz J, Zubia Zubia B. [clinical trial with sulpiride and placebo in chronic schizophrenics (study of 89 patients using the harris-letemendia-willems scale)]. *Archivos de Neurobiologia* 1972; **35**: 453–72.

259 Min SK, Rhee CS, Kim CE, Kang DY. Risperidone versus haloperidol in the treatment of chronic schizophrenic patients: A parallel group double-blind comparative trial. *Yonsei Medical Journal* 1993; **34**: 179–90.

260 Mirabzadeh A, Kimiaghalam P, Fadai F, Samiei M, Daneshmand R. The therapeutic effectiveness of risperidone on negative symptoms of schizophrenia in comparison with haloperidol: A randomized clinical trial. *Iranian Journal of Neuroscience* 2014; **5**: 212–7.

261 Moller HJ, Boyer P, Fleurot O, Rein W. Improvement of acute exacerbations of schizophrenia with amisulpride: A comparison with haloperidol. PROD-aslp study group. *Psychopharmacology* 1997; **132**: 396–401.

262 Montgomery SA, Green M, Rimon R *et al.* Inadequate treatment response to des-enkephalin-gamma-endorphin compared with thioridazine and placebo in schizophrenia. *Acta Psychiatrica Scandinavica* 1992; **86**: 97–103.

263 Moore DF. Treatment of acute schizophrenia with loxapine succinate (loxitane) in a controlled study with chlorpromazine. *Current Therapeutic Research, clinical & Experimental* 1975; **18**: 172–80.

264 Moosavi SM, Ahmadi M, Mojtahedi D, Yazdani J, M BM. Comparison of quetiapine and risperidone in treatment of acute psychosis: A double-blind, randomized-controlled study. *Global Journal of Health Science* 2015; **7**: 41952.

265 Mori K, Nagao M, Yamashita H, Morinobu S, Yamawaki S. Effect of switching to atypical antipsychotics on memory in patients with chronic schizophrenia. *Progress in Neuro-Psychopharmacology & Biological Psychiatry* 2004; **28**: 659–65.

266 Morris PA, MacKenzie DH, Masheter HC. A comparative double blind trial of pimozide and fluphenazine in chronic schizophrenia. *British Journal of Psychiatry* 1970; **117**: 683–4.

267 Moyano CZ. A double blind comparison of loxitane(TM) loxapine succinate and trifluoperazine hydrochloride in chronic schizophrenic patients. *Disease of the Nervous System* 1975; **36**: 301–4.

268 Murasaki M, Miura S, Yamashita I *et al.* Efficacy of a new antipsychotic, risperidone, on schizophrenia. A comparative double blind study with haloperidol. *Rinsho Hyoka* 1993; **21**: 221–59.

269 Murasaki M, Koyama T, Fukushima Y *et al.* clinical evaluation of quetiapine fumarate on schizophrenia: Comparative double-blind study with haloperidol. *Seishin Igaku* 2001; **4**: 127–55.

270 Nagesh HN, Anil Kumar Nagaraj, Kishore MS, Narendra kumar MS. A randomized prospective comparative study of weight gain between asenapine and iloperidone in patients with psychosis -. *National Journal of Physiology, Pharmacy and Pharmacology* 2017; **7**: 94–8.

271 Nam CW, Yang BH, Lee JN. The influences of risperidone and clozapine on body weight and glucose level in patients with chronic schizophrenia: Korean journal of biological psychiatry. 2004; **11**: 127–35.

272 NCT00350467. A randomized, active-controlled, double-blind, parallel-goup study of the efficacy and safety of extended release(ER) paliperidone in the treatment of schizophrenia: Http://clinicaltrials.gov/show/nct00350467</web_address>. 2006.

273 NCT00563706. Study evaluating vabicaserin in subjects with schizophrenia: Http://clinicaltrials.gov/show/nct00563706</web_address>. 2007.

274 NCT00882518. Efficacy and safety of quetiapine fumarate in the treatment of schizophrenic patients: Http://clinicaltrials.gov/show/nct00882518</web_address>. 2009.

275 Kane JM, Skuban A, Hobart M *et al.* Overview of short- and long-term tolerability and safety of brexpiprazole in patients with schizophrenia. *Schizophrenia Research* 2016; **174**: 93–8.

276 Kinoshita T, Bai YM, Kim JH, Miyake M, Oshima N. Efficacy and safety of asenapine in asian patients with an acute exacerbation of schizophrenia: A multicentre, randomized, double-blind, 6-week, placebo-controlled study. *Psychopharmacology* 2016; **233**: 2663–74.

277 Durgam S, Cutler AJ, Lu K *et al.* Cariprazine in acute exacerbation of schizophrenia: A fixed-dose, phase 3, randomized, double-blind, placebo- and active-controlled trial. *Journal of clinical Psychiatry* 2015; **76**: e1574–82.

278 Landbloom R, Mackle M, Wu X *et al.* Asenapine for the treatment of adults with an acute exacerbation of schizophrenia: Results from a randomized, double-blind, fixed-dose, placebo-controlled trial with olanzapine as an active control. *CNS Spectr* 2016;: 1–9.

279 Marder SR, Hakala MJ, Josiassen MK *et al.* Brexpiprazole in patients with schizophrenia: Overview of short- and long-term phase 3 controlled studies. *Acta Neuropsychiatr* 2016;: 1–13.

280 Nishizono M. A comparative trial zotepine, chlorpromazine and haloperidol in schizophrenic patients. *Neuropsychopharmacology* 1994; **10 (suppl.)**: 30S.

281 Nistico G, Ragozzine D, Marano V. A comparative study of penfluridol and flupentixol in the treatment of chronic schizophrenia. *Journal of clinical Pharmacology* 1974; **14**: 476–82.

282 O'Brien CP, DiGiacomo JN, Webb W. Management of hostile, suspicious patients. Trifluoperazine versus haloperidol. *Diseases of the Nervous System* 1974; **35**: 75–8.

283 Ortega-Soto HA, Apiquian R, Ulloa RE *et al.* Olanzapine vs risperidone. A double blind trial in mexican patients conference abstract: Regional meeting of the collegium internationale neuropsychopharmacologicum and the colegio mexicano de neuropsucofarmacologia, acapulco, mexico. August 21-23rd, 1997. 1997.

284 Ozguven HD, Baskak B, Oner O, Atbasoglu C. Metabolic effects of olanzapine and quetiapine: A six-week randomized, single blind, controlled study. *Open Neuropsychopharmacology Journal* 2011; **4**: 10–7.

285 Paprocki J, Barcala Peixoto MP, Andrade NM. A controlled double-blind comparison between loxapine and haloperidol in acute newly hospitalized schizophrenic patients. *Psychopharmacology Bulletin* 1976; **12**: 32–4.

286 Park S, Yi KK, Kim MS, Hong JP. Effects of ziprasidone and olanzapine on body composition and metabolic parameters: An open-label comparative pilot study. *Behavioral & Brain Functions [Electronic Resource]: BBF* 2013; **9**: 27.

287 Pathiraja AP, Diamond BI, Borison RL, Meibech RC, Anand R. Relationship between creatine phosphokinase, psychotic symptoms and novel antipsychotic drugs conference abstract: Schizophrenia research (the vth international congress on schizophrenia research, wormsprings, va usa. 6th-12th april, 1995.). 1995; **1, 2**.

288 Patil ST, Zhang L, Martenyi F *et al.* Activation of mGlu2/3 receptors as a new approach to treat schizophrenia: A randomized phase 2 clinical trial.[Erratum appears in nat med. 2007 oct;13(10):1264]. *Nature Medicine* 2007; **13**: 1102–7.

289 Payne P. A comparison of trifluopromazine, chlorpromazine, and a placebo in twenty-one chronic schizophrenic patients. 1960.

290 Petit M, Raniwalla J, Tweed J, Leutenegger E, Dollfus S, Kelly F. A comparison of an atypical and typical antipsychotic, zotepine versus haloperidol in patients with acute exacerbation of schizophrenia: A parallel-group double-blind trial. *Psychopharmacology Bulletin* 1996; **32**: 81–7.

291 Peuskens J. Risperidone in the treatment of patients with chronic schizophrenia: A multi-national, multi-centre, double-blind, parallel-group study versus haloperidol. Risperidone study group. *British Journal of Psychiatry* 1995; **166**: 712–26; discussion 727–33.

292 Peuskens J, Link CG. A comparison of quetiapine and chlorpromazine in the treatment of schizophrenia. *Acta Psychiatrica Scandinavica* 1997; **96**: 265–73.

293 Peuskens J, Bech P, Moller HJ, Bale R, Fleurot O, Rein W. Amisulpride vs. Risperidone in the treatment of acute exacerbations of schizophrenia. Amisulpride study group. *Psychiatry Research* 1999; **88**: 107–17.

294 Pfizer. Dose-response study to evaluate safety, efficacy, and pharmacokinetics of pf-00217830 compared with placebo in acute exacerbation of schizophrenia. 2007.

295 Pi E, Sramek J, Johnson T *et al.* Subjective neuroleptic response and treatment outcome under open and double-blind conditions - a preliminary report. *Progress in Neuro-Psychopharmacology and Biological Psychiatry* 1990; **14**: 921–8.

296 Pichot P, Dreyfus JF. Sulpiride and chlorpromazine in treatment of acute psychoses: Die therapiewoche. 1983; **33**: 4571–4.

297 Potkin SG, Buchsbaum MS, Jin Y *et al.* Clozapine effects of glucose metabolic rate in striatum and frontal cortex. *Journal of clinical Psychiatry* 1994; **55**: 63–6.

298 Potkin SG, Saha AR, Kujawa MJ *et al.* Aripiprazole, an antipsychotic with a novel mechanism of action, and risperidone vs placebo in patients with schizophrenia and schizoaffective disorder. *Archives of General Psychiatry* 2003; **60**: 681–90.

299 Potkin SG, Cohen M, Panagides J. Efficacy and tolerability of asenapine in acute schizophrenia: A placebo- and risperidone-controlled trial. *Journal of clinical Psychiatry* 2007; **68**: 1492–500.

300 Potter WZ, Ko GN, Zhang LD, Yan WW. Clozapine in china: A review and preview of us/prc collaboration. *Psychopharmacology* 1989; **99 Suppl**: S87–91.

301 128-301 S. Study report of study 128-301. *Pfizer, data on file* 1997.

302 ZIP-NY-97-019 S. Study report on study zip-ny-97-019. *Pfizer, data on file* 2004.

303 Puech A, Fleurot O, Rein W. Amisulpride, and atypical antipsychotic, in the treatment of acute episodes of schizophrenia: A dose-ranging study vs. Haloperidol. The amisulpride study group. *Acta Psychiatrica Scandinavica* 1998; **98**: 65–72.

304 Ramsay RA, Ban TA, Lehmann HE, Saxena BM, Bennett J. A comparative study of molindone and trifluoperazine. *Current Therapeutic Research, clinical & Experimental* 1970; **12**: 438–40.

305 Ramu MG, Chaturvedi DD, Venkataram BS *et al.* A double blind controlled study on the role of bahmyadiyoga and tagara in navonmada (acute schizophrenia). In: Ayurvedic management of unmada (schizophrenia). 1999.

306 Ramu MG, Chaturvedi DD, Venkataram BS *et al.* A double blind controlled study on the role of bahmyadiyoga and tagara in jirnomada (chronic schizophrenia). In: Ayurvedic management of unmada (schizophrenia). 1999: 77–88.

307 Reardon JD, Abrams S. Acute paranoid schizophrenia. (Treatment with chlorpromazine, trifluoperazine and placebo). *Diseases of the Nervous System* 1966; **27**: 265–70.

308 Remvig J, Larsen H, Rask P, Skausig OB, Skov S, Stromgren LS. Zuclopenthixol and perphenazine in patients with acute psychotic states. A double-blind multicentre study. *Pharmacopsychiatry* 1987; **20**: 147–54.

309 Rickels K, Byrdy H, Valentine J, Postel W, Norstad N, Downing R. Double-blind trial of thiothixene and chlorpromazine in acute schizophrenia. *International Pharmacopsychiatry* 1978; **13**: 50–7.

310 Riedel M, Muller N, Spellmann I *et al.* Efficacy of olanzapine versus quetiapine on cognitive dysfunctions in patients with an acute episode of schizophrenia. *European Archives of Psychiatry & clinical Neuroscience* 2007; **257**: 402–12.

311 Rifkin A, Rieder E, Sarantakos S, Saraf K, Kane J. Is loxapine more effective than chlorpromazine in paranoid schizophrenia? *American Journal of Psychiatry* 1984; **141**: 1411–3.

312 Rodova A, Svestka J, Nahunek K, Ceskova E. A blind comparison of clozapine and perphenazine in schizophrenics: Activitas nervosa superior. 1973; **15**: 94–5.

313 Rosenheck R, Perlick D, Bingham S *et al.* Effectiveness and cost of olanzapine and haloperidol in the treatment of schizophrenia: A randomized controlled trial. *JAMA* 2003; **290**: 2693–702.

314 Rubin R. A double-blind comparison of the onset of activity of haloperidol and trifluoperazine. *Alabama Journal of Medical Sciences* 1971; **8**: 414–8.

315 Ruiz-Navarro JF, Vergara L, Deutsch M. Proceedings: A comparative study of thiothixene and chlorpromazine in chronic schizophrenic patients: Application of a special psychometric test battery. *Psychopharmacology Bulletin* 1976; **12**: 38–9.

316 Rüther E, J. B. Therapievergleich von aminosultoprid (dan 2163) und perazin bei schizophrenen patienten. In: H. H, Hippius H, R. T, eds. Therapie mit neuroleptika - perazin. Stuttgart New York: Georg Thieme Verlag, 1988: 65–70.

317 Sacchetti E, Valsecchi P, Parrinello G, Group Q. A randomized, flexible-dose, quasi-naturalistic comparison of quetiapine, risperidone, and olanzapine in the short-term treatment of schizophrenia: The querisola trial. *Schizophrenia Research* 2008; **98**: 55–65.

318 Safa M, Sadr S, Delfan B, Saki M, Javad Tarrahi M. Metabolic effects of olanzapine and risperidone in patients with psychotic disorders. *International Journal of Psychiatry in clinical Practice* 2008; **12**: 299–302.

319 Sakalis G, Chan TL, Sathananthan G, Schooler N, Goldberg S, Gershon S. Relationships among clinical response, extrapyramidal syndrome and plasma chlorpromazine and metabolite ratios. *Communications in Psychopharmacology* 1977; **1**: 157–66.

320 Sandison RAWECJDC. clinical trials with melleril (tp21) in the treatment of schizophrenia. A two-year study: Journal of mental science. 1960; **106**: 732–41.

321 Sarai K, Okada M. Comparison of efficacy of zotepine and thiothixene in schizophrenia in a double-blind study. *Pharmacopsychiatry* 1987; **20**: 38–46.

322 Saretsky T. Effects of chlorpromazine on primary-process thought manifestations. *Journal of Abnormal Psychology* 1966; **71**: 247–52.

323 Sato G, Yoshimura S, Yamashita H, Okamoto Y, Yamawaki S. The neurocognitive effects of aripiprazole compared with risperidone in the treatment of schizophrenia. *Hiroshima Journal of Medical Sciences* 2012; **61**: 75–83.

324 Schennach R, Riedel M, Spellmann I *et al.* Comparing schizophrenia patients with a predicted high/low risk of nonresponse receiving treatment with ziprasidone and haloperidol: A randomized-controlled study. *Pharmacopsychiatry* 2018. DOI:10.1055/a-0669-9461.

325 Tuason VB, Escobar JI, Garvey M, Schiele B. LOXAPINE vs. CHLORPROMAZINE in paranoid schizophrenia a double-blind study. *Journal of clinical Psychiatry* 1984; **45**: 158–63.

326 Schimmelmann BG, Moritz S, Karow A *et al.* Correlates of subjective well-being in schizophrenic patients treated with atypical antipsychotics. *International Journal of Psychiatry in clinical Practice* 2005; **9**: 94–8.

327 Schmidt LG, Schussler G, Kappes CV, Muller-Oerlinghausen B. A double-blind comparative-trial of 2 oral neuroleptics (perazine vs. Haloperidol) in different dosages for acute schizophrenic patients: Der nervenarzt. 1982; **53**: 530–6.

328 Schmidt ME, Kent JM, Daly E *et al.* A double-blind, randomized, placebo-controlled study with jnj-37822681, a novel, highly selective, fast dissociating d2 receptor antagonist in the treatment of acute exacerbation of schizophrenia. *European Neuropsychopharmacology* 2012; **22**: 721–33.

329 See RE, Fido AA, Maurice M, Ibrahim MM, Salama GM. Risperidone-induced increase of plasma norepinephrine is not correlated with symptom improvement in chronic schizophrenia. *Biological Psychiatry* 1999; **45**: 1653–6.

330 Selman FB, McClure RF, Helwig H. Loxapine succinate: A double-blind comparison with haloperidol and placebo in acute schizophrenics. *Current Therapeutic Research, clinical & Experimental* 1976; **19**: 645–52.

331 Serafetinides EA, Willis D, Clark ML. Haloperidol, clopenthixol, and chlorpromazine in chronic schizophrenia. *Journal of Nervous & Mental Disease* 1972; **155**: 366–9.

332 Sergi MJ, Green MF, Widmark C *et al.* Social cognition [corrected] and neurocognition: Effects of risperidone, olanzapine, and haloperidol.[Erratum appears in am j psychiatry. 2007 nov;164(11):1766]. *American Journal of Psychiatry* 2007; **164**: 1585–92.

333 Seth S, Mahal AS, Kumar KA. A double blind comparative trial of loxapine and trifluoperazine in chronic schizophrenic patients. *Current Therapeutic Research - clinical and Experimental* 1979; **25**: 320–9.

334 Shah S, Joshi D. Tolerability and efficacy of paliperidone er compared to olanzapine in the treatment of schizophrenia: A randomized, double-blind, multicentric trial. *Industrial Psychiatry Journal* 2011; **20**: 25–31.

335 Shen JH, Zhao Y, Rosenzweig-Lipson S *et al.* A 6-week randomized, double-blind, placebo-controlled, comparator referenced trial of vabicaserin in acute schizophrenia. *Journal of Psychiatric Research* 2014; **53**: 14–22.

336 Shepherd M, Watt DC. A controlled clinical study of chlorpromazine and reserpine in chronic schizophrenia: Journal of neurochemistry. 1956; **19**: 232–5.

337 Shopsin B, Pearson E, Gershon S, Collins P. A controlled double-blind comparison between loxapine succinate and chlorpromazine in acute newly hospitalized schizophrenic patients. *Current Therapeutic Research, clinical & Experimental* 1972; **14**: 739–48.

338 Silverstone T, Cookson J, Ball R *et al.* The relationship of dopamine receptor blockade to clinical response in schizophrenic patients treated with pimozide or haloperidol. *Journal of Psychiatric Research* 1984; **18**: 255–68.

339 Simpson GM, Amin M, Edwards JG. A double-blind comparison of molindone and trifluoperazine in the treatment of acute schizophrenia. *Journal of clinical Pharmacology & New Drugs* 1971; **11**: 227–36.

340 Simpson GM, Cuculic Z. A double-blind comparison of loxapine succinate and trifluoperazine in newly admitted schizophrenic patients. *Journal of clinical Pharmacology* 1976; **16**: 60–5.

341 Simpson GM, Glick ID, Weiden PJ, Romano SJ, Siu CO. Randomized, controlled, double-blind multicenter comparison of the efficacy and tolerability of ziprasidone and olanzapine in acutely ill inpatients with schizophrenia or schizoaffective disorder. *American Journal of Psychiatry* 2004; **161**: 1837–47.

342 Singer K, Law SK. A double blind comparison of clozapine (leponex) and chlorpromazine in schizophrenia of acute symptomatology. *Journal of International Medical Research* 1974; **2**: 433–5.

343 Singh MM, Kay SR. A comparative study of haloperidol and chlorpromazine in terms of clinical effects and therapeutic reversal with benztropine in schizophrenia. Theoretical implications for potency differences among neuroleptics. *Psychopharmacologia* 1975; **43**: 103–13.

344 Small JG, Hirsch SR, Arvanitis LA, Miller BG, Link CG. Quetiapine in patients with schizophrenia. A high- and low-dose double-blind comparison with placebo. Seroquel study group. *Archives of General Psychiatry* 1997; **54**: 549–57.

345 Sonmez B, Vardar E, Altun GD, Abay E, Bedel D. Ziprasidone versus risperidone: Comparison of clinical efficacy and cardiac, extrapyramidal, and metabolic side effects in patients with acute exacerbation of schizophrenia and schizoaffective disorders. *Klinik Psikofarmakoloji Bulteni / Bulletin of clinical Psychopharmacology* 2009; **19**: 101–12.

346 Spohn HE, Lacoursiere RB, Thompson K, Coyne L. Phenothiazine effects on psychological and psychophysiological dysfunction in chronic schizophrenics. *Archives of General Psychiatry* 1977; **34**: 633–44.

347 Steinbook RM, Goldstein BJ, Brauzer B, Moreno SS, Jacobson AF. Loxapine: A double-blind comparison with chlorpromazine in acute schizophrenic patients. *Current Therapeutic Research, clinical & Experimental* 1973; **15**: 1–7.

348 Ogasa M, Kimura T, Nakamura M, Guarino J. Lurasidone in the treatment of schizophrenia: A 6-week, placebo-controlled study. *Psychopharmacology* 2013; **225**: 519–30.

349 Potkin SG, Kimura T, Guarino J. A 6-week, double-blind, placebo- and haloperidol-controlled, phase ii study of lurasidone in patients with acute schizophrenia.[Erratum appears in ther adv psychopharmacol. 2015 dec;5(6): 369; pmid: 26836398]. *Therapeutic Advances in Psychopharmacology* 2015; **5**: 322–31.

350 2000 S1. Center for drug evaluation and research approval package for application number 20-825. Medical review. *http://wwwfdagov* 2000.

351 Nakamura M, Ogasa M, Guarino J *et al.* Lurasidone in the treatment of acute schizophrenia: A double-blind, placebo-controlled trial. *Journal of clinical Psychiatry* 2009; **70**: 829–36.

352 Nasrallah HA, Silva R, Phillips D *et al.* Lurasidone for the treatment of acutely psychotic patients with schizophrenia: A 6-week, randomized, placebo-controlled study. *Journal of Psychiatric Research* 2013; **47**: 670–7.

353 Meltzer HY, Cucchiaro J, Silva R *et al.* Lurasidone in the treatment of schizophrenia: A randomized, double-blind, placebo- and olanzapine-controlled study. *American Journal of Psychiatry* 2011; **168**: 957–67.

354 Harvey PD, Loebel A, Cucchiaro J, Phillips D, Siu C. Is quality of life related to cognitive performance or negative symptoms in patients with schizophrenia? Results from a double-blind, active-controlled, lurasidone extension study. *Neuropsychopharmacology* 2013; **38**: S515.

355 Potkin SG, Litman RE, Torres R, Wolfgang CD. Efficacy of iloperidone in the treatment of schizophrenia: Initial phase 3 studies. *Journal of clinical Psychopharmacology* 2008; **28**: S4–11.

356 Feeney J, Wolfgang C, Polymeropoulos M, Baroldi P, Hamilton J. The comparative efficacy of iloperidone and haloperidol across four short-term controlled trials: (Poster nr1-026). *Presented at the 162nd Annual Meeting of the American Psychiatric Association, San Francisco*, 2009.

357 2002 S9. Center for drug evaluation and research. Application number 21-436. Medical review(s). *http://wwwfdagov* 2002.

358 2002 S9. Center for drug evaluation and research. Application number 21-436. Medical review(s). *http://wwwfdagov* 2002.

359 Durgam S, Litman RE, Papadakis K, Li D, Nemeth G, Laszlovszky I. Cariprazine in the treatment of schizophrenia: A proof-of-concept trial. *International clinical Psychopharmacology* 2016; **31**: 61–8.

360 Kane JM, Zukin S, Wang Y *et al.* Efficacy and safety of cariprazine in acute exacerbation of schizophrenia: Results from an international, phase iii clinical trial. *Journal of clinical Psychopharmacology* 2015; **35**: 367–73.

361 1996 SR-U-7. Office of clinical pharmacology and biopharmacy review. NDA number: 20272. *Janssen-Cilag, data on file* 1996.

362 Svendsen BB, Faurbye A, Kristjansen P. Comparison of the effect of thioridazine and chlorpromazine on chronic schizophrenic psychosis using double blind technique. *Psychopharmacologia* 1961; **2**: 446–55.

363 Svestka J, Nahunek K. A comparison of pimozide with perphenazine in the treatment of acute schizophrenic psychoses. *Activitas Nervosa Superior* 1972; **14**: 93–4.

364 Svestka J, Ceskova E, Rysanek R, Obrovska V. Double-blind clinical comparison of riperidon and haloperidol in acute schizophrenic and schizoaffective psychoses. *Activitas Nervosa Superior* 1990; **32**: 237–8.

365 Svestka J, Rysanek R, Nahunek K, Ceskova E. [Results of treatment of schizophrenic and schizoaffective patients with sulpiride (eglonyl alkaloid) in comparison with perphenazine (perfenazin spofa)]. *Ceskoslovenska Psychiatrie* 1990; **86**: 145–56.

366 Svestka J, Synek O, Zourkova A. A double-blind comparison of olanzapine and quetiapine in treatment of acute exacerbations of schizophrenic or schizoaffective disorders. *European Neuropsychopharmacology* 2003; **13**: S292.

367 Svestka J, Synek O, Zourkova A. A double-blind comparison of olanzapine and ziprasidone in treatment of acute exacerbations of schizophrenic or schizoaffective disorders. *PsychiatrDanub* 2005.

368 Tamrakar SM, Nepal MK, Koirala NR, Sharma VD, Gurung CK, Adhikari SR. An open, randomized, comparative study of efficacy and safety of risperidone and haloperidol in schizophrenia. *Kathmandu University Medical Journal* 2006; **4**: 152–60.

369 Taneli B, Alptekin K, Bilici M *et al.* Comparison of efficacy and tolerability of quetiapine and haloperidol in acute exacerbation of chronic or subchronic schizophrenia. *EurNeuropsychopharmacol* 2003; **13**: S287–7.

370 Tapp A, Wood AE, Kilzieh N, Kennedy A. Double-blind comparison of quetiapine and haloperidol on cognitive functioning in patients with schizophrenia. *Schizophrenia Bulletin* 2005; **31**: 379.

371 Tetreault L, Bordeleau JM, Gauthier R, Vulpe M, Lapointe L. Comparative study of tps-23, chlorpromazine and placebo in chronic schizophrenic patients. *Diseases of the Nervous System* 1969; **30**: Suppl:74–84.

372 Tollefson GD, Beasley, C. M., Jr., Tran PV *et al.* Olanzapine versus haloperidol in the treatment of schizophrenia and schizoaffective and schizophreniform disorders: Results of an international collaborative trial. *American Journal of Psychiatry* 1997; **154**: 457–65.

373 Schiele BC. Loxapine succinate: A controlled double-blind study in chronic schizophrenia. *Diseases of the Nervous System* 1975; **36**: 361–4.

374 Tybura P, Trzesniowska-Drukala B, Bienkowski P *et al.* Pharmacogenetics of adverse events in schizophrenia treatment: Comparison study of ziprasidone, olanzapine and perazine. *Psychiatry Research* 2014; **219**: 261–7.

375 van Bruggen J, Tijssen J, Dingemans P, Gersons B, Linszen D. Symptom response and side-effects of olanzapine and risperidone in young adults with recent onset schizophrenia. *International clinical Psychopharmacology* 2003; **18**: 341–6.

376 van der Velde, C. D., Kiltie H. Effectiveness of loxapine succinate in acute schizophrenia: A comparative study with thiothixene. *Current Therapeutic Research, clinical & Experimental* 1975; **17**: 1–12.

377 van Kammen DP, McEvoy JP, Targum SD, Kardatzke D, Sebree TB. A randomized, controlled, dose-ranging trial of sertindole in patients with schizophrenia. *Psychopharmacology* 1996; **124**: 168–75.

378 Vichaiya V. clinical trial of haloperidol in schizophrenia. *Journal of Psychiatric Association of Thailand* 1971; **16**: 31–43.

379 Wagner M, Quednow BB, Westheide J, Schlaepfer TE, Maier W, Kuhn KU. Cognitive improvement in schizophrenic patients does not require a serotonergic mechanism: Randomized controlled trial of olanzapine vs amisulpride. *Neuropsychopharmacology* 2005; **30**: 381–90.

380 Waldrop FN, Robertson RH, Vourlekis A. A comparison of the therapeutic and toxic effects of thioridazine and chlorpromazine in chronic schizophrenic patients. *Comprehensive Psychiatry* 1961; **2**: 96–105.

381 Walsh GP, Walton D, Black DA. The relative efficacy of Vespral and chlorpromazine in the treatment of a group of chronic schizophrenic patients. *British Journal of Psychiatry* 1959; **105**: 199–209.

382 Weston MJ, Bentley R, Unwin A, Morris M, Harper MA. A comparative trial of haloperidol and thioridazine: Management of chronic schizophrenia. *Australian & New Zealand Journal of Psychiatry* 1973; **7**: 52–7.

383 Wetzel H, Bardeleben U von, Holsboer F, Benkert O. [Zotepine versus perazine in patients with paranoid schizophrenia: A double-blind controlled trial of its effectiveness]. *Fortschritte der Neurologie-Psychiatrie* 1991; **59 Suppl 1**: 23–9.

384 Wetzel H, Grunder G, Hillert A *et al.* Amisulpride versus flupentixol in schizophrenia with predominantly positive symptomatology – a double-blind controlled study comparing a selective d2-like antagonist to a mixed d1-/d2-like antagonist. The amisulpride study group. *Psychopharmacology* 1998; **137**: 223–32.

385 Wynn JK, Green MF, Sprock J *et al.* Effects of olanzapine, risperidone and haloperidol on prepulse inhibition in schizophrenia patients: A double-blind, randomized controlled trial. *Schizophrenia Research* 2007; **95**: 134–42.

386 Yamashita H, Mori K, Nagao M, Okamoto Y, Morinobu S, Yamawaki S. Effects of changing from typical to atypical antipsychotic drugs on subjective sleep quality in patients with schizophrenia in a japanese population. *Journal of clinical Psychiatry* 2004; **65**: 1525–30.

387 Yen YC, Lung FW, Chong MY. Adverse effects of risperidone and haloperidol treatment in schizophrenia. *Progress in Neuro-Psychopharmacology & Biological Psychiatry* 2004; **28**: 285–90.

388 Zborowski J, Schmitz P, Staser J *et al.* Efficacy and safety of sertindole in a trial of schizophrenic patients. *BiolPsychiatry* 1995; **37**: 661–2.

389 Zhang B. A 4-week, randomized, rater-blinded, parallelgroup, haloperidol-controlled study to evaluate the efficacy of quetiapine fumarate in improving sleep quality in the patients with schizophrenia. *International Journal of Neuropsychopharmacology* 2010; **13**: 113–4.

390 Zhang H, Li H, Shu L *et al.* Double-blind comparison of ziprasidone and risperidone in the treatment of chinese patients with acute exacerbation of schizophrenia. *Neuropsychiatric Disease & Treatment* 2011; **7**: 77–85.

391 Zhong KX, Sweitzer DE, Hamer RM, Lieberman JA. Comparison of quetiapine and risperidone in the treatment of schizophrenia: A randomized, double-blind, flexible-dose, 8-week study. *Journal of clinical Psychiatry* 2006; **67**: 1093–103.

392 Ziegler B. Study of the efficacy of a substituted benzamide amisulpride, versus haloperidol, in productive schizophrenia. In: Amisulpride. Paris: Expansion scientifique francaise, 1989: 73–81.

393 Zimbroff DL, Kane JM, Tamminga CA *et al.* Controlled, dose-response study of sertindole and haloperidol in the treatment of schizophrenia. Sertindole study group. *American Journal of Psychiatry* 1997; **154**: 782–91.

394 Zimbroff D, Warrington L, Loebel A, Yang R, Siu C. Comparison of ziprasidone and aripiprazole in acutely ill patients with schizophrenia or schizoaffective disorder: A randomized, double-blind, 4-week study. *International clinical Psychopharmacology* 2007; **22**: 363–70

The number of references (394) is lower than the number of included studies (402), because some publications reported on several studies

Appendix 8: Risk of Bias

The following table shows the risk of bias assessment for the individual domains.

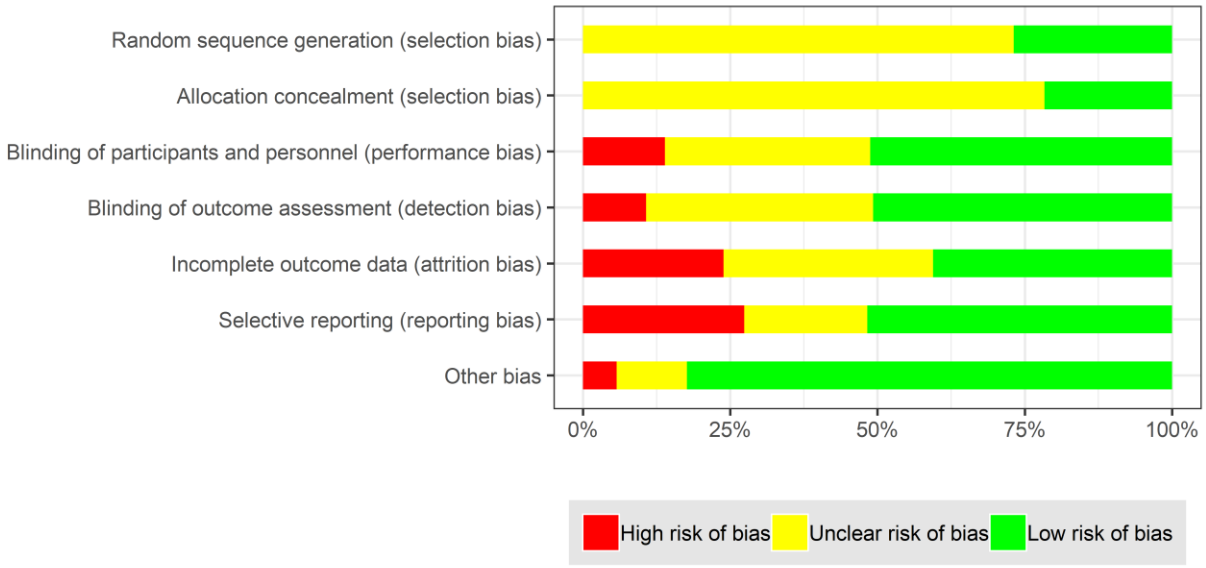

Figure 8.1: Risk of bias assessment for the individual domains

Risk of bias assessment for the individual studies:

Study	Random sequence generation (selection bias)	Allocation concealment (selection bias)	Blinding of participants and personnel (performance bias)	Blinding of outcome assessment (detection bias)	Incomplete outcome data (attrition bias)	Selective reporting (reporting bias)	Other bias
A1281046	?	?	+	?	?	?	?
A1281050	?	?	?	?	?	?	?
Abdolahian 2008	?	?	?	?	?	+	+
Addington 2004	+	+	?	?	+	+	+
Ahmed 2007	?	?	?	?	?	?	?
Ahn 2007	?	?	-	?	?	?	?
Allan 1998	?	?	?	?	?	?	+
Amakusa 1973	?	+	+	+	-	?	+
Angst 1971	?	?	?	+	-	-	-
Anumonye 1976	?	?	?	?	+	-	+
Arvanitis 1997	?	?	?	?	+	+	+
Atmaca 2002	?	?	?	?	+	-	+
Augustin 1996	?	?	?	?	?	?	?
Avasthi 2001	?	?	-	-	+	+	+
Azorin 2006	?	?	?	?	+	+	-
Baker 1959	?	+	+	?	+	+	+
Balasubramanian 1991	?	?	+	+	+	+	+
Ban 1975e	?	?	+	+	-	-	+

Study	Random sequence generation (selection bias)	Allocation concealment (selection bias)	Blinding of participants and personnel (performance bias)	Blinding of outcome assessment (detection bias)	Incomplete outcome data (attrition bias)	Selective reporting (reporting bias)	Other bias
Barbato 2007c	?	?	?	?	?	?	?
Barbato 2007d	?	?	?	?	?	?	?
Beasley 1996a	+	+	+	+	+	+	+
Beasley 1996b	+	+	?	?	+	+	+
Beasley 1997	+	+	+	+	+	+	+
Bechelli 1983	+	?	+	?	-	-	-
Bergling 1975	+	?	+	+	-	-	+
Bernardo 2001	?	?	?	?	?	?	+
Bhowmick 2010	+	?	-	-	?	+	+
Bishop 1963c	?	?	?	+	?	-	+
Bishop 1964	?	?	+	+	?	+	+
Bishop 1970	?	?	?	?	+	-	+
Blin 1996	?	?	+	+	+	+	+
Boehle 1995	?	?	?	?	?	?	?
Borison 1989	?	?	?	?	-	+	+
Borison 1991a	?	?	?	?	?	-	+
Borison 1992	+	?	+	+	+	+	-
Borison 1996	?	?	+	+	+	+	+
Boulay 2007	?	?	?	?	+	-	+
Bratfos 1979	?	?	+	+	-	-	-

Study	Random sequence generation (selection bias)	Allocation concealment (selection bias)	Blinding of participants and personnel (performance bias)	Blinding of outcome assessment (detection bias)	Incomplete outcome data (attrition bias)	Selective reporting (reporting bias)	Other bias
Brauzer 1971	?	?	+	+	-	+	+
Brook 1998	?	?	?	+	-	+	+
Brook 2005	+	+	-	?	+	+	+
Buchsbaum 2009	?	?	?	?	?	?	+
Bueno 1979	?	?	+	+	?	-	+
Bugarski-Kirola	?	?	?	?	+	+	+
Burnett 1975	?	?	?	?	?	?	+
Butler 2000	?	?	+	-	+	-	?
Camara 1978	?	?	-	-	?	-	+
Canive 2006	+	?	?	?	-	+	+
Cantillon 2014	?	?	?	?	?	?	+
Carranza 1974	?	?	?	?	-	+	+
Casey 1960	?	+	+	+	-	-	+
Casey 2008	?	?	?	?	+	-	+
Cassano 1975	?	?	+	+	?	-	+
Cavallaro 2001	?	+	+	+	-	-	+
Ceskova 1993	?	?	?	?	?	+	+
Ceskova 1994	?	?	+	+	-	?	+
Cetin 2010	?	?	-	-	?	?	?
Chan 2007	+	+	+	+	+	+	+

Study	Random sequence generation (selection bias)	Allocation concealment (selection bias)	Blinding of participants and personnel (performance bias)	Blinding of outcome assessment (detection bias)	Incomplete outcome data (attrition bias)	Selective reporting (reporting bias)	Other bias
Charalampous 1974, 00559	?	?	+	+	?	-	+
Chen 2018	?	?	-	-	-	+	+
Chiu 1976	?	?	+	+	-	-	-
Chouinard 1970	?	?	+	+	?	+	+
Chouinard 1975	?	?	+	+	?	+	+
Chouinard 1976	?	?	?	?	-	+	+
Chouinard 1982	?	?	+	+	+	-	+
Chouinard 1990	?	?	+	+	?	?	-
Chouinard 1993	?	?	+	+	+	+	+
Chung 2000	?	?	?	?	?	?	?
Chung 2012	?	?	-	-	-	-	+
Citrome 2015	?	+	-	-	+	+	+
Ciurezu 1976	?	+	+	+	+	-	+
Clark 1967	+	+	+	+	?	-	+
Clark 1969	?	?	?	?	?	-	-
Clark 1970a	?	?	+	+	+	+	+
Clark 1971a	?	?	+	+	?	-	+
Clark 1972	?	?	+	+	-	+	+
Clark 1975	+	?	+	+	+	+	+
Clark 1977 12 weeks, 02912	?	?	+	+	-	+	+

Study	Random sequence generation (selection bias)	Allocation concealment (selection bias)	Blinding of participants and personnel (performance bias)	Blinding of outcome assessment (detection bias)	Incomplete outcome data (attrition bias)	Selective reporting (reporting bias)	Other bias
Clark 1977a	?	?	?	?	+	-	-
Claus 1992	?	?	-	-	+	+	+
Conley 2001	?	?	?	?	+	+	+
Cooper 2000a	?	?	+	+	+	+	+
Copolov 2000	?	?	?	?	+	+	+
Correll 2015	+	?	?	?	+	+	+
Corrigan 2004	+	+	+	+	+	-	+
Corripio 2005	?	?	-	?	+	-	+
Cosar 1999	?	?	-	-	?	?	?
Costa e Silva 1989	+	+	+	+	-	?	-
Crowley 1981	?	?	?	?	?	?	+
Cutler 2006	?	?	+	+	+	+	+
Cutler 2008	+	+	+	+	-	+	+
Cutler 2008a	?	?	+	+	+	+	+
Daniel 1999	+	?	?	?	+	+	+
Davidson 2007	+	+	+	+	+	+	+
de Boer 2011	?	?	-	-	-	?	+
de Oliviera 2009	?	?	-	-	+	+	+
Dehnel 1968	?	+	+	+	+	+	+
DeJong 1965	?	?	+	+	-	+	+

Study	Random sequence generation (selection bias)	Allocation concealment (selection bias)	Blinding of participants and personnel (performance bias)	Blinding of outcome assessment (detection bias)	Incomplete outcome data (attrition bias)	Selective reporting (reporting bias)	Other bias
Delcker 1990	?	?	?	?	?	?	+
dElia 1974	?	?	?	?	?	-	+
DeMartinis 2012	?	?	+	+	+	+	+
Denber 1972	?	?	+	+	-	+	+
Dieterle 1991	?	?	+	+	?	+	+
Dolnak 1996	?	?	?	?	?	?	?
Dolnak 2001	?	?	?	?	?	?	?
Dossenbach 2007	?	?	-	-	+	-	+
Downing 2014	?	?	+	+	+	+	+
Dube 1976	?	?	+	+	-	+	+
Duggan 2005	+	?	-	-	?	?	+
Durgam 2014	?	?	?	?	+	+	+
Dutta 2014	?	?	-	?	?	?	?
Ebrinc 2004	?	?	-	-	?	?	?
Edwards 1980	?	?	+	+	-	+	+
Egan 2013	+	+	+	+	+	+	+
Ehmann 1987	?	?	+	+	?	+	+
Ehrlich 2012	?	?	-	-	-	-	+
Engelhardt 1969, 03748	?	?	+	+	?	?	+
ENLIGHTEN-1	?	?	?	?	+	+	+

Study	Random sequence generation (selection bias)	Allocation concealment (selection bias)	Blinding of participants and personnel (performance bias)	Blinding of outcome assessment (detection bias)	Incomplete outcome data (attrition bias)	Selective reporting (reporting bias)	Other bias
Erlandsen 1981	?	?	-	-	?	?	+
Escobar 1985	?	?	+	+	-	-	+
Evans 1972	?	?	+	+	?	-	+
Fabre 1995	?	?	?	?	+	+	+
Fakra 2008	?	?	-	-	-	+	+
Faustman 1995	?	?	?	?	?	?	?
Fischer-Cornellsen 1976a	?	?	+	+	?	-	-
Fischer-Cornellsen 1976b	?	?	+	+	?	-	-
Fleischhacker 1989	+	+	+	+	-	-	+
Fleischhacker 2009_6weeks	+	+	+	+	+	+	+
Fleming 1959	?	?	+	+	?	-	+
Fleming 1968	?	?	+	+	?	?	?
Freeman 1969	?	?	+	+	-	+	+
Fruensgaard 1978acute	?	?	?	?	-	-	+
Fruensgaard 1978chronic	?	?	?	?	-	-	+
Gallant 1963	?	?	?	?	?	?	+
Gallant 1966	?	?	+	+	?	+	+
Gallant 1967	?	?	+	+	?	-	+
Gallant 1968	?	?	?	?	-	-	+
Garcia 2009	+	?	+	+	+	-	+

Study	Random sequence generation (selection bias)	Allocation concealment (selection bias)	Blinding of participants and personnel (performance bias)	Blinding of outcome assessment (detection bias)	Incomplete outcome data (attrition bias)	Selective reporting (reporting bias)	Other bias
Garry 1962b	+	?	?	?	-	-	+
Gattaz 2004	?	?	?	?	+	+	+
Geffen 2012	+	+	?	?	+	+	+
Gelenberg 1979	?	?	?	?	-	+	-
Gerlach 1974	?	?	-	-	+	-	+
Gerlach 1975	?	?	+	+	?	+	+
Ghaleiha 2011	+	?	?	?	-	-	+
Goff 1998	?	?	?	?	+	+	+
Goldberg 1972	?	?	+	+	-	-	+
Goldstein 1966	?	?	+	+	-	+	+
Goldstein 1969	?	?	+	+	?	-	+
Gowardman 1973	?	+	+	+	+	+	+
Granacher 1982	+	?	+	+	+	+	+
Grootens 2009	?	?	+	?	?	+	+
Guirguis 1977	?	+	+	+	-	-	-
Gupta 2017	?	?	-	-	-	-	?
Guz 2002	?	?	?	?	?	?	?
Haas 1982	?	?	+	+	?	-	+
Hadlik 1970	?	?	?	?	?	?	?
Hale 2000	+	+	+	+	+	+	+

Study	Random sequence generation (selection bias)	Allocation concealment (selection bias)	Blinding of participants and personnel (performance bias)	Blinding of outcome assessment (detection bias)	Incomplete outcome data (attrition bias)	Selective reporting (reporting bias)	Other bias
Hall 1955	?	?	+	+	?	-	+
Hall 1968	?	?	+	+	+	+	+
Harnryd 1984	?	?	+	+	?	+	+
Hatta 2009	+	+	+	-	+	+	-
Hatta 2013	+	+	-	?	+	+	+
Haug 1959	?	?	?	?	?	?	?
Heikkila 1981	?	?	?	?	-	+	+
Heikkilae 1992	?	?	+	+	+	+	+
Heikkinen 1993	?	?	?	?	?	-	+
Heinrich 1994	?	?	?	?	-	-	+
Hera 041-021	?	?	+	?	?	?	?
Hera 041-022	?	?	+	?	+	?	?
Herrera 1990, 00640	?	?	+	+	+	+	+
Hogan 1992	?	?	-	-	?	-	+
Honigfeld 1984a	?	?	+	+	?	?	+
Honigfeld 1984c	?	?	?	?	?	?	+
Honigfeld 1984d	?	?	+	+	?	-	+
Howell 1961	+	?	+	+	?	?	+
Hoyberg 1993	?	?	+	+	+	+	+
Huttunen 1995	?	?	?	?	+	-	+

Study	Random sequence generation (selection bias)	Allocation concealment (selection bias)	Blinding of participants and personnel (performance bias)	Blinding of outcome assessment (detection bias)	Incomplete outcome data (attrition bias)	Selective reporting (reporting bias)	Other bias
Hwang 2001	?	?	+	+	+	+	+
Hwang 2003	?	?	+	+	+	+	+
Hwang 2012	?	?	?	?	?	?	?
Imai 1980	?	?	+	+	?	-	+
Ingole 2009	?	?	-	-	+	+	+
Ishigooka 2001	?	?	?	?	+	+	+
Ishigooka 2018	+	+	?	?	+	+	+
Itil 1971	?	?	?	?	+	+	+
Itoh 1976	?	?	?	?	?	+	+
Itoh 1977	?	?	?	?	+	-	+
Janicak 2001	?	?	+	+	+	+	+
Jann 1997	?	?	+	+	+	?	+
Janssen CR012625	?	?	+	+	?	?	+
Jindal 2013	+	?	+	+	-	+	+
Johnson NCT00397033	+	+	+	+	+	+	+
Johnson NCT00412373	+	+	+	+	+	+	+
Johnson NCT00524043	+	+	+	+	+	+	+
Johnstone 1978, 01073	?	?	+	+	+	+	+
Judd 1973	?	?	?	-	?	-	+
Kahn 2007	?	?	+	+	+	+	+

Study	Random sequence generation (selection bias)	Allocation concealment (selection bias)	Blinding of participants and personnel (performance bias)	Blinding of outcome assessment (detection bias)	Incomplete outcome data (attrition bias)	Selective reporting (reporting bias)	Other bias
Kane 2002	?	?	?	?	+	+	+
Kane 2007b	+	+	+	+	+	+	+
Kane 2009 8 weeks	?	?	?	?	+	+	+
Kane 2010a	?	?	+	+	?	+	+
Kane 2015	+	+	+	+	+	+	+
Kaushal 2012	+	?	-	-	?	-	+
Keck 1998	+	?	?	?	+	+	+
Kenway 1971	?	?	+	+	+	+	-
Khorana 1988	?	?	+	+	-	-	+
Kiloh 1976acute	?	?	+	+	-	-	+
King 1959	?	?	?	?	?	-	+
Kingstone 1970	?	?	+	+	?	+	+
Kinon 2011	+	+	+	+	-	+	+
Klein 1973	?	?	+	+	?	?	+
Klein 1985	+	+	?	?	-	-	+
Klieser 1989	+	?	?	?	?	+	+
Klieser 1990	?	?	?	?	?	?	+
Klieser 1991	?	?	?	?	?	?	+
Klieser 1994	?	?	?	?	-	-	+
Klimke 1993	?	?	?	?	+	-	+

Study	Random sequence generation (selection bias)	Allocation concealment (selection bias)	Blinding of participants and personnel (performance bias)	Blinding of outcome assessment (detection bias)	Incomplete outcome data (attrition bias)	Selective reporting (reporting bias)	Other bias
Kluge 2007	+	+	+	+	+	+	+
Knegtering 2004	?	?	-	-	-	-	?
Knegtering 2006	?	?	-	-	+	-	+
Kordas 1968	?	?	+	+	?	-	+
Kramer 1978	?	?	+	+	-	+	+
Kurland 1961	?	?	+	+	-	-	+
Lahti 2009	?	?	+	+	-	+	+
Lamure 2003	?	?	-	-	-	+	+
Lemmer 1993	?	?	?	?	?	?	?
Lemperiere 1985	?	?	?	?	?	?	+
Leon 1974	+	?	-	-	?	+	+
Levita 1961	?	?	+	+	?	-	+
Li 2012	+	?	-	+	+	+	+
Lieberman 2005 12weeks	?	?	+	+	+	+	+
Liebermann 2015	+	?	+	+	+	+	+
Liemburg 2011	?	?	-	-	-	-	+
Lin 2003	?	?	-	?	+	+	-
Lindenmayer 2008	+	+	+	+	+	+	+
Litman 2016	+	?	+	+	+	+	+
Litmann 2014	+	+	+	+	+	+	+

Study	Random sequence generation (selection bias)	Allocation concealment (selection bias)	Blinding of participants and personnel (performance bias)	Blinding of outcome assessment (detection bias)	Incomplete outcome data (attrition bias)	Selective reporting (reporting bias)	Other bias
Little 1958	?	?	+	+	?	-	+
Liu 2000	+	?	?	?	-	-	+
Loebel 2015a	+	+	+	+	+	+	+
Loza 1999	?	?	-	-	+	+	+
Loza 2006	?	?	?	?	?	-	+
Lublin 1991	?	?	+	?	?	?	+
Luckey 1967	?	?	+	+	?	+	+
Lundbeck NCT00864045	?	?	+	+	+	+	+
Maat 2014	?	?	-	-	-	-	+
Mahadevan 1991	?	?	-	+	-	+	+
Mahal 1976	?	?	+	+	-	+	+
Marder 1994	+	?	+	+	+	+	+
Marder 2007c	+	+	+	+	+	+	+
Martin 2002	+	+	+	+	+	+	+
McCue2006	+	?	-	-	+	+	+
McEvoy 2007b	+	+	+	+	+	+	+
McInnes 1978	+	?	?	?	?	-	+
McQuade 2004_6weeks	?	?	?	?	-	+	-
Meltzer 2004	+	?	?	?	+	+	+
Meltzer 2007a	?	?	?	?	+	?	?

Study	Random sequence generation (selection bias)	Allocation concealment (selection bias)	Blinding of participants and personnel (performance bias)	Blinding of outcome assessment (detection bias)	Incomplete outcome data (attrition bias)	Selective reporting (reporting bias)	Other bias
Menon 1972	?	?	-	+	?	+	+
Mesotten 1991	?	?	+	+	?	?	?
Meyer-Lindenberg 1997	+	+	+	+	+	?	+
Mezquita 1972	?	?	-	-	?	+	+
Min 1993	+	+	+	+	+	-	+
Mirabzadeh 2014	?	?	?	?	?	+	-
Möller 1997	+	+	+	+	+	+	+
Montgomery 1992	?	?	+	+	+	-	+
Moore 1975	?	?	+	+	?	+	+
Moosavi 2015	+	?	?	?	-	+	+
Mori 2004	?	?	?	?	-	-	+
Morris 1970	?	?	+	+	?	?	+
Moyano 1975	?	?	+	+	-	+	+
Murasaki 1993	?	?	?	?	?	+	+
Murasaki 2001	?	?	?	?	?	+	+
Nagesh 2016	?	?	-	-	-	-	+
Nam 2004	?	?	-	?	?	?	?
NCT00350467	?	?	+	+	?	+	+
NCT00563706	?	?	+	+	+	+	+
NCT00882518	?	?	?	?	+	+	-

Study	Random sequence generation (selection bias)	Allocation concealment (selection bias)	Blinding of participants and personnel (performance bias)	Blinding of outcome assessment (detection bias)	Incomplete outcome data (attrition bias)	Selective reporting (reporting bias)	Other bias
NCT00905307	+	+	+	+	+	+	+
NCT01098110	+	?	+	+	+	+	+
NCT01104766	?	?	?	?	+	+	+
NCT01617187	+	?	+	+	+	+	+
NCT01810380	?	?	?	?	+	+	+
Nishizono 1994	?	+	?	?	?	?	?
Nistico 1974,	?	?	+	+	-	-	+
O Brien 1974	?	?	+	+	-	-	+
Ortega-Soto 1997	?	?	?	?	?	?	?
Ozguven 2004	+	+	-	?	?	-	?
Paprocki 1976	?	?	?	?	?	-	+
Park 2013	?	?	-	?	-	+	+
Pathiraja 1995	?	?	?	?	?	?	?
Patil 2007	?	?	?	?	+	-	+
Payne 1960	?	+	+	+	?	+	+
Petit 1996	?	?	+	+	+	+	+
Peuskens 1995	+	+	+	+	+	+	+
Peuskens 1997	+	+	-	-	+	+	+
Peuskens 1999	+	+	+	+	+	+	+
Pfizer 2008	?	?	?	?	?	?	?

Study	Random sequence generation (selection bias)	Allocation concealment (selection bias)	Blinding of participants and personnel (performance bias)	Blinding of outcome assessment (detection bias)	Incomplete outcome data (attrition bias)	Selective reporting (reporting bias)	Other bias
Pi 1990, 01400	?	?	+	+	?	-	+
Pichot 1983	+	?	+	+	+	+	+
Potkin 1994	?	?	+	+	?	?	+
Potkin 2003	?	?	?	?	+	-	+
Potkin 2007c	?	?	+	+	+	+	+
Potter 1989	?	?	?	?	?	?	?
Protocol 128-301	?	?	?	?	?	?	?
Protocol ZIP-NY-97-019	?	?	?	?	?	?	?
Puech 1998	+	+	+	+	+	+	+
Ramsay 1970	?	?	+	+	?	-	+
Ramu 1999	?	+	+	+	-	-	+
Ramu 1999a	?	?	+	+	-	-	+
Reardon 1966	?	?	-	+	-	+	+
Remvig 1987	?	?	+	?	+	+	+
Rickels 1978	?	?	+	+	?	-	+
Riedel 2007	?	?	?	?	-	+	+
Rifkin 1984	?	?	+	+	-	?	+
Rodova 1973	?	?	?	?	?	-	+
Rosenheck 2003w6	+	+	+	+	?	+	-
Rubin 1971	?	?	+	+	?	+	-

Study	Random sequence generation (selection bias)	Allocation concealment (selection bias)	Blinding of participants and personnel (performance bias)	Blinding of outcome assessment (detection bias)	Incomplete outcome data (attrition bias)	Selective reporting (reporting bias)	Other bias
Ruiz Navarro 1976	?	?	?	?	?	?	?
Rüther 1988	+	+	+	+	?	+	+
Sacchetti 2008	+	?	-	?	-	?	+
Safa 2008	?	?	?	?	-	+	+
Sakalis 1977	?	?	+	+	-	-	+
Sandison 1960	?	+	+	+	?	-	+
Sarai 1987	?	+	?	?	?	-	+
Saretsky 1966	?	?	+	+	?	+	+
Sato 2012	?	?	-	?	-	?	?
Schennach 2018	?	?	?	?	+	+	+
Schiele 1975	?	?	+	+	?	?	+
Schimmelmann 2005	?	?	?	?	+	+	+
Schmidt 1982	?	?	?	-	-	+	+
Schmidt 2014	+	+	+	+	+	-	+
See 1999	?	?	?	?	?	-	+
Selman 1976	?	?	+	+	+	+	+
Serafetinides 1972	?	?	+	+	-	+	+
Sergi 2007	+	?	?	?	-	-	+
Seth 1979	?	?	+	+	-	+	+
Shah 2011	?	?	+	+	+	-	?

Study	Random sequence generation (selection bias)	Allocation concealment (selection bias)	Blinding of participants and personnel (performance bias)	Blinding of outcome assessment (detection bias)	Incomplete outcome data (attrition bias)	Selective reporting (reporting bias)	Other bias
Shen 2014	+	+	?	?	-	-	+
Sheperd 1956, 01660	?	?	+	+	+	-	+
Shopsin 1972	?	?	+	+	?	?	+
Silverstone 1984	?	?	+	+	-	+	+
Simpson 1971	?	+	+	+	?	+	+
Simpson 1976	?	?	+	+	?	+	+
Simpson 2004	+	+	+	+	+	-	+
Singer 1974	?	?	?	?	?	-	+
Singh 1975	+	?	+	+	-	?	+
Small 1997	+	?	?	?	+	+	+
Sonmez 2009	?	?	-	-	-	?	?
Spohn 1977	?	+	+	+	-	+	+
Steinbook 1973	?	?	+	+	?	+	+
Study 006	+	+	+	+	+	+	+
Study 049	+	+	+	+	+	+	+
Study 115 2000	+	?	?	?	+	?	+
Study 196	+	+	+	+	+	+	+
Study 229	+	+	+	+	+	+	+
Study 231	+	+	+	+	+	+	+
Study 233	+	+	+	+	+	+	+

Study	Random sequence generation (selection bias)	Allocation concealment (selection bias)	Blinding of participants and personnel (performance bias)	Blinding of outcome assessment (detection bias)	Incomplete outcome data (attrition bias)	Selective reporting (reporting bias)	Other bias
Study 3000	+	+	+	+	+	+	+
Study 3001	?	?	?	?	?	?	+
Study 3002	?	?	?	?	?	?	?
Study 3003	?	?	?	?	?	?	?
Study 3004	+	+	+	+	+	+	+
Study 3005	+	+	+	+	+	+	+
Study 93202 2002	?	?	?	?	+	+	+
Study 94202 2002	?	?	+	+	+	+	+
Study RGH-MD-03	?	?	?	?	+	?	+
Study RGH-MD-05	?	?	?	?	+	+	+
Study RIS-USA-72 1996	?	?	+	+	+	+	+
Svendsen 1961	+	+	+	+	-	?	+
Svestka 1972	?	?	?	?	?	-	+
Svestka 1990	?	?	?	?	?	?	+
Svestka 1990a	?	?	?	?	?	?	?
Svestka 2003a	+	+	+	+	?	+	+
Svestka 2005	+	+	+	+	?	+	+
Tamrakar 2006	?	?	-	-	-	+	+
Taneli 2003	?	?	-	-	+	?	?
Tapp 2005	?	?	?	?	?	?	?

Study	Random sequence generation (selection bias)	Allocation concealment (selection bias)	Blinding of participants and personnel (performance bias)	Blinding of outcome assessment (detection bias)	Incomplete outcome data (attrition bias)	Selective reporting (reporting bias)	Other bias
Tetreault 1969a	+	?	+	+	?	+	+
Tollefson 1997	+	+	?	?	-	+	+
Tuason 1984	?	?	+	+	?	+	+
Tybura 2014	+	+	-	+	-	?	+
van Bruggen 2003	?	?	-	-	-	+	+
van der Velde 1975	?	?	+	+	+	?	+
van Kammen 1996	+	?	?	?	+	+	+
Vichaya 1971	?	+	-	-	+	-	+
Wagner 2005	+	+	?	?	+	+	+
Waldrop 1961	?	?	+	+	-	+	+
Walsh 1959	?	+	+	+	+	-	+
Weston 1973	?	?	+	+	-	+	+
Wetzel 1991	?	?	?	?	-	+	+
Wetzel 1998	+	+	+	+	+	+	-
Wynn 2007	+	?	?	?	-	-	+
Yamashita 2004	?	?	-	-	+	+	+
Yen 2004	?	?	-	?	+	+	+
Zborowski 1995	+	+	+	+	+	+	+
Zhang 2010	?	?	?	?	?	?	?
Zhang 2011	+	+	+	+	+	+	+

Study	Random sequence generation (selection bias)	Allocation concealment (selection bias)	Blinding of participants and personnel (performance bias)	Blinding of outcome assessment (detection bias)	Incomplete outcome data (attrition bias)	Selective reporting (reporting bias)	Other bias
Zhong 2006	+	+	+	+	+	+	+
Ziegler 1989	+	+	?	?	-	+	+
Zimbroff 1997	+	+	?	?	+	-	+
Zimbroff 2007	+	+	+	+	+	+	+

Appendix 9: Metaregression (adjusted models)

The following paragraph lists the the adjusted models for the primary outcome overall change in symptoms. Because of poor convergence, we excluded drugs with lower than 100 people randomized (Figure 9). The overall ranking did not change considerably in all examined moderators.

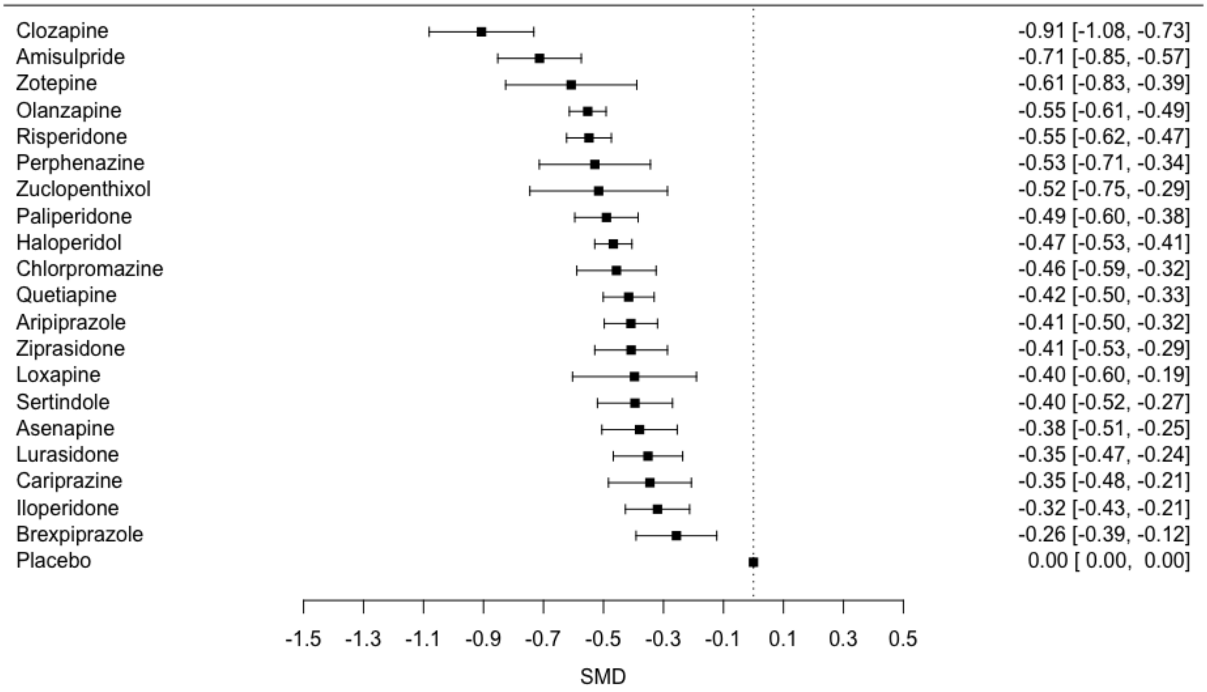

Figure 9: Overall change in symptoms for for drugs with at least 100 participants
Treatments are ranked according to standardised mean difference (SMD) compared to placebo. Treatments crossing the y-axis are not significantly different from placebo.

Changes in heterogeneity

Below we present the results from the changes in heterogeneity in each meta-regression model.

Meta-regression			
Covariate	τ	% of variance explained	Coefficients modelled as
Unadjusted	0.118	-	-
Placebo	0.072	-62.77	drug-specific
Placebo	0.075	-59.60	common
Year of publication	0.111	-11.51	common
Sample size	0.123	8.65	drug-specific
Baseline severity	0.121	5.15	drug-specific
Mean age	0.111	-11.51	drug-specific
Percentage males	0.112	-9.91	drug-specific
Sponsoring	0.107	-17.78	common

Table 9.1: Changes in heterogeneity of all metaregressions

9.1 Placebo response

The study publication year ranges between 1967 and 2018. At the same time, the response to placebo (measured as change in PANSS in the placebo arm) increases over time as shown in the graph below, as also shown in a previous publication.[4]

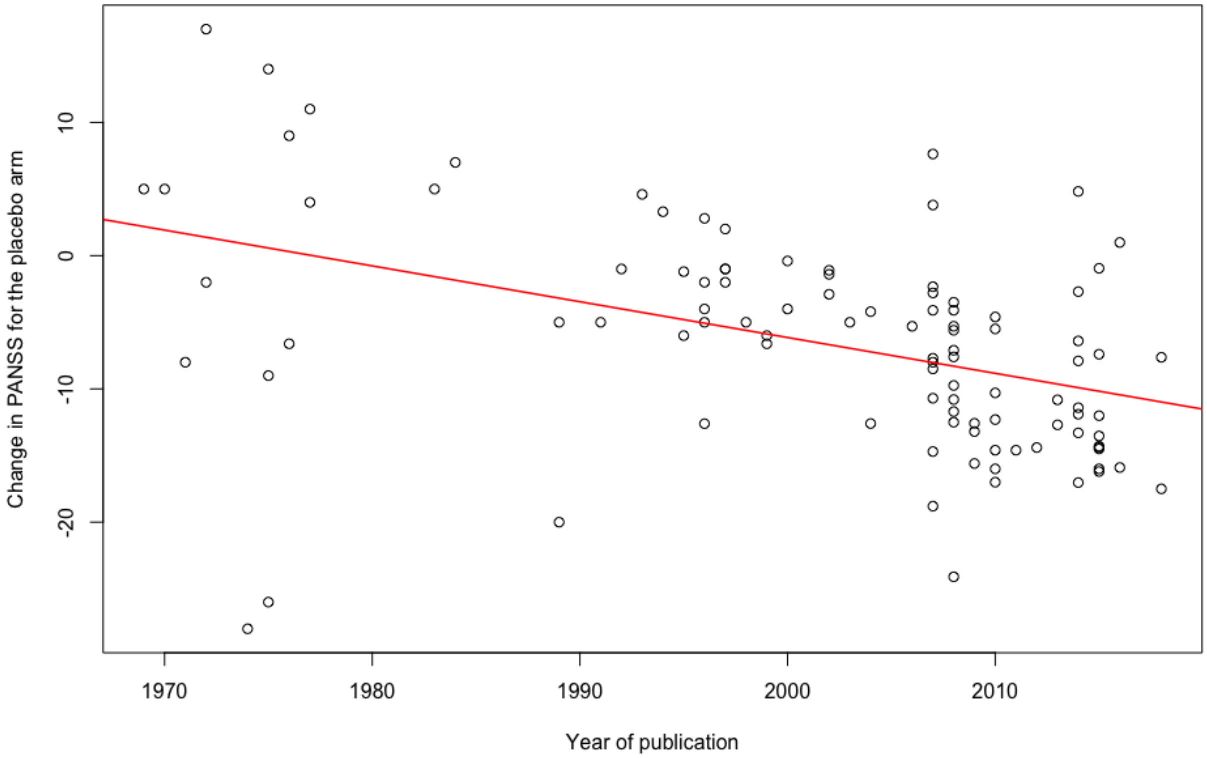

Fig. 9.1.1: Change of placebo response over time

The mean change in the PANSS score in the placebo arm was -6.62 with median -6.40 and IQR -12.60 to -2.00.

Some drugs were studied primarily in old studies while some others are newer. The table below shows the mean year of publication in study arms:

Drug name	Mean of year of randomisation
Amisulpride	1995
Aripiprazole	2008
Asenapine	2011
Brexpiprazole	2016
Cariprazine	2013
Chlorpromazine	1982
Clopenthixol	1970
Clozapine	1989
Flupentixol	1993
Fluphenazine	1970
Haloperidol	1995
Iloperidone	2008
Levomepromazine	1996
Loxapine	1976
Lurasidone	2011
Molindone	1976
Olanzapine	2007
Paliperidone	2008
Penfluridol	1976
Perazine	1990
Perphenazine	1986
Pimozide	1978
Placebo	2001
Quetiapine	2004
Risperidone	2003
Sertindole	2001
Sulpiride	1979
Thioridazine	1983
Thiothixene	1977
Trifluoperazine	1973
Ziprasidone	2005
Zotepine	1994
Zuclopenthixol	1991

To address whether and how much the degree of placebo-response had and impact on the results, we fit two network meta-regression models where the standardized mean difference (SMD) was a function of the change in PANSS for the placebo arm as described in the methods (appendix 4).

In one analysis we assumed a common coefficient across drugs, because as shown in Figure 9.1.2 below the direction of the effect (except for cariprazine) was always the same. In the second analysis we used specific coefficients for each compound. In both analyses in studies without a placebo-arm, we fit a hierarchical model where a response to placebo value was stochastically imputed as a prediction from the year of the study publication.

In the following figure 9.1.2 we present both the drug-specific and the mean coefficients β to assess the impact of placebo-response:

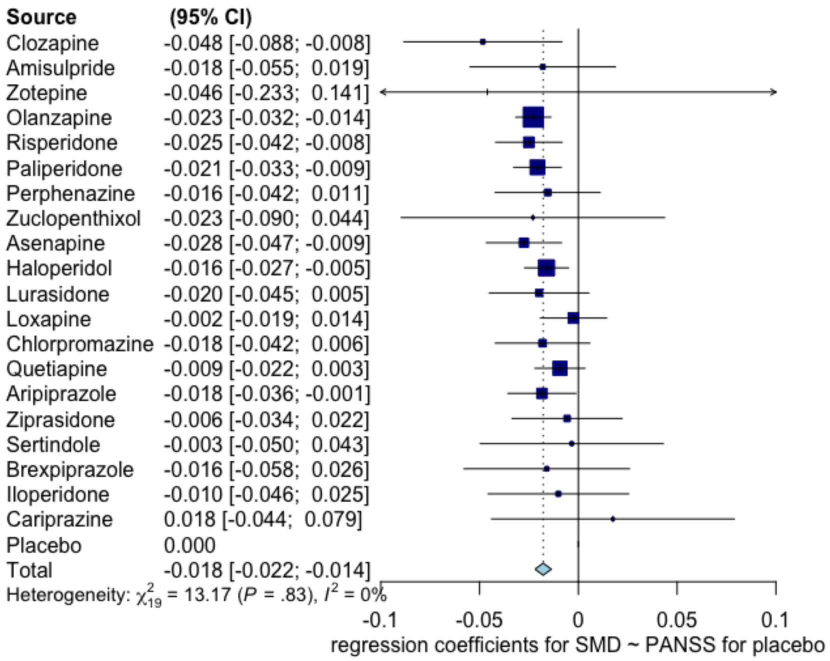

Figure 9.1.2: Coefficients of individual drugs (comparison-specific coefficients)
Regressions coefficents of placebo response for the individual drugs presented as change standardized mean difference per point change in placebo group. The pooled regression coefficient suggests that for a 10-units increase in PANSS there is -0.018 [-0.022; -0.014] difference in SMD.

In Figure 9.1.3 we present the result of the first model using drug specific coefficients. The SMDs presented below are the intercepts from the meta-regression model and they represent the SMDs for an imaginary situation where patients in placebo change their PANSS score from baseline by -6 units which was the median placebo response in the studies. We see that overall the hierarchies do not change much.

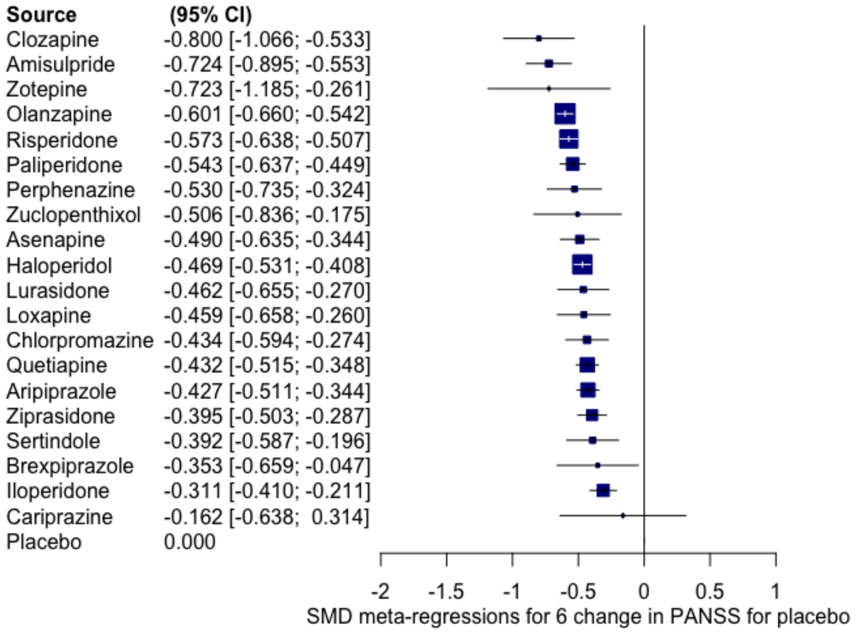

Figure 9.1.3: Placebo response (comparison-specific coefficients) Placebo response was adapted to a six point PANSS decrease from baseline using comparison specific coefficients. Treatments are ranked according to standardised mean difference (SMD) compared to placebo. Treatments crossing the y-axis are not significantly different from placebo. The heterogeneity standard deviation drops from 0.118 to 0.072 (63% relative change in the heterogeneity variance).

In Figure 9.1.4 we present the results using a common coefficient for all drugs. Here, the hierarchy of the effect sizes is even less changed than in the analysis with drug specific coefficients:

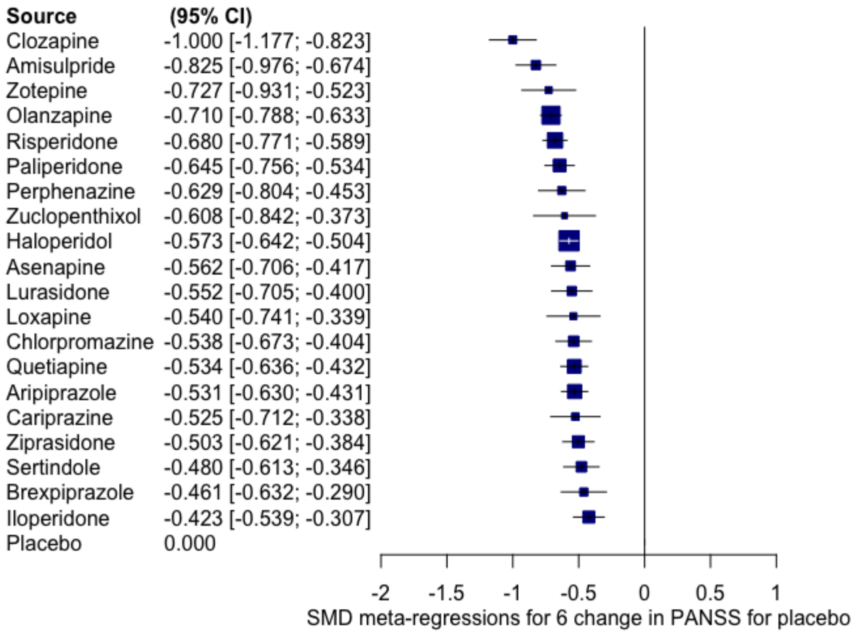

Figure 9.1.4: Placebo response (common coefficient) Placebo response was adapted to a six point PANSS decrease from baseline using a common coefficient drawn from the same distribution. Treatments are ranked according to standardised mean difference (SMD) compared to placebo. Treatments crossing the y-axis are not significantly different from placebo. The common coefficent B with 95% CrI are -0.017 (-0.023, -0.011) and the heterogeneity is close to the one estimated from the more flexible model ($\tau = 0.075$)

In summary, we interprete the findings such that adjusting the results by either model did not materially change the results much. This finding was corroborated by two sensivity analyses excluding placebo-controlled studies altogether. **These analyses are particularly important because they are not affected by placebo-response, at all. Please see appendix 10.1 and 10.2 for details.**

9.2 Publication year

When the model was adjusted for publication in year 2016, loxapine as an older drug had a lower SMD compared to the unadjusted model. The remaining hierarchy was similar to the unadjusted model.

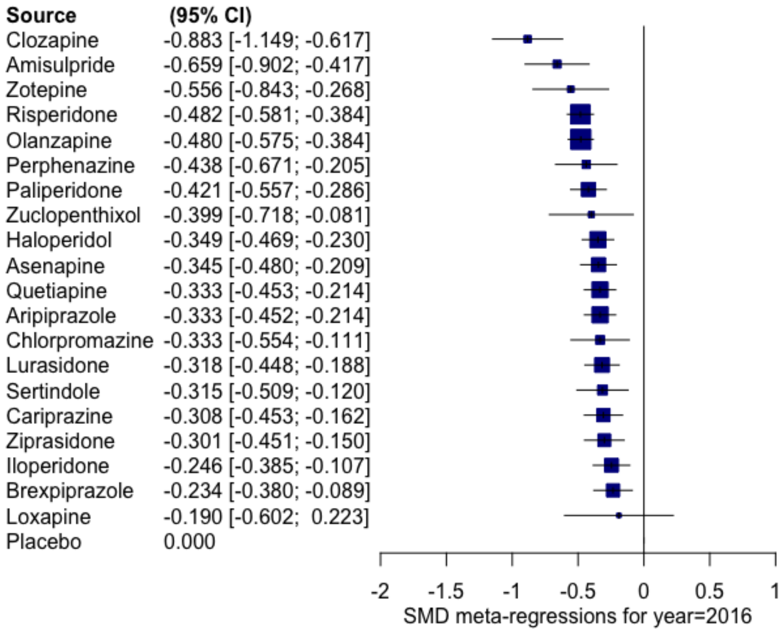

Figure 9.2: Forest plot overall change in symptoms adjusted for publication year 2016
Treatments are ranked according to standardised mean difference (SMD) compared to placebo. Treatments crossing the y-axis are not significantly different from placebo.

9.3 Sample size

When the model was adjusted for an infinetely large study, no significant changes in the hierarchy were observed.

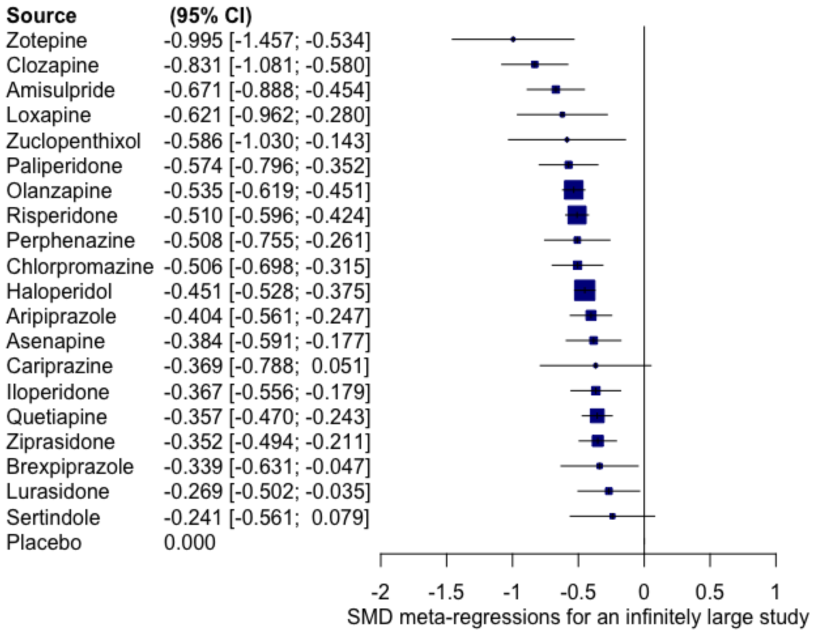

Figure 9.3: Forest plot overall change in symptoms adjusted for infinetely large sample size
Treatments are ranked according to standardised mean difference (SMD) compared to placebo. Treatments crossing the y-axis are not significantly different from placebo.

9.4 Age

When the model was adjusted for mean age 40, some drugs had higher SMDs, but the hierachy from the unadjusted model remained.

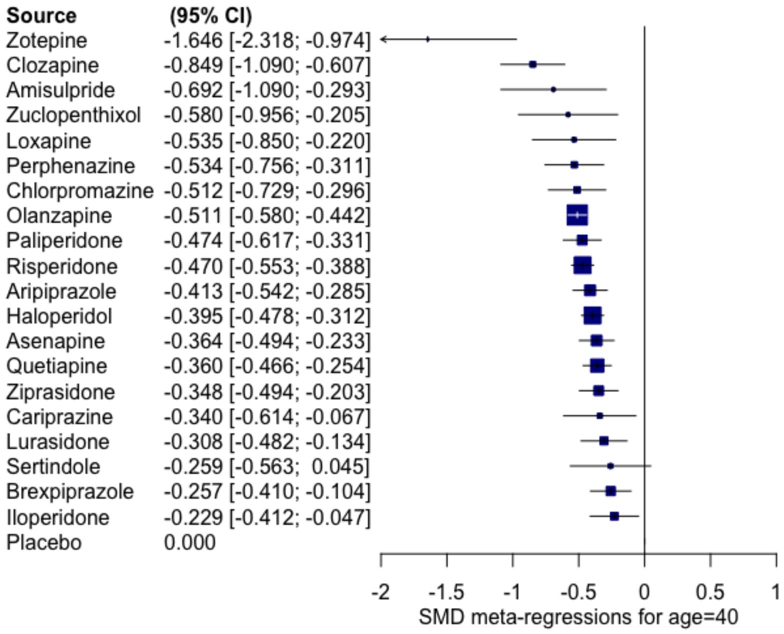

Figure 9.4: Forest plot overall change in symptoms adjusted for mean age 40
Treatments are ranked according to standardised mean difference (SMD) compared to placebo. Treatments crossing the y-axis are not significantly different from placebo.

9.5 Baseline severity

When the model was adjusted for to a baseline severit of 94 on the Positive and Negative Syndrome Scale, no significant changes in the hierarchy were observed.

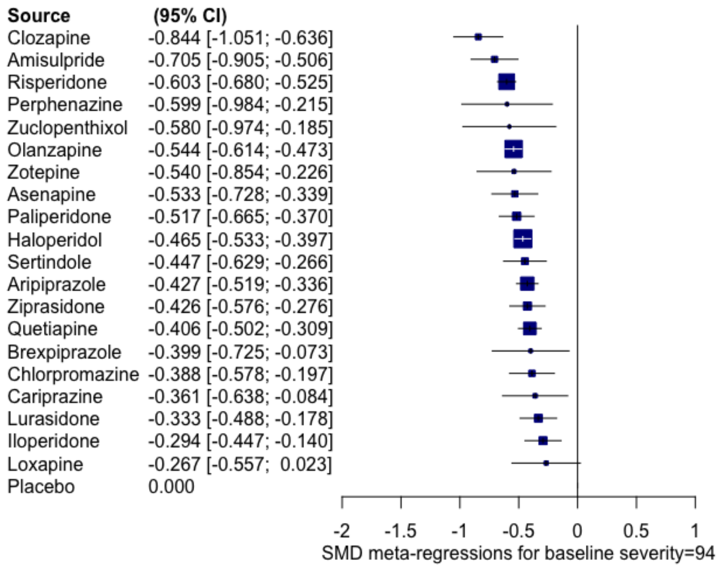

Figure 9.5 Forest plot overall change in symptoms adjusted for baseline severity
Treatments are ranked according to standardised mean difference (SMD) compared to placebo. Treatments crossing the y-axis are not significantly different from placebo.

9.6 Male participants

When the model was adjusted for 50% male participants, no significant changes in the hierarchy were observed.

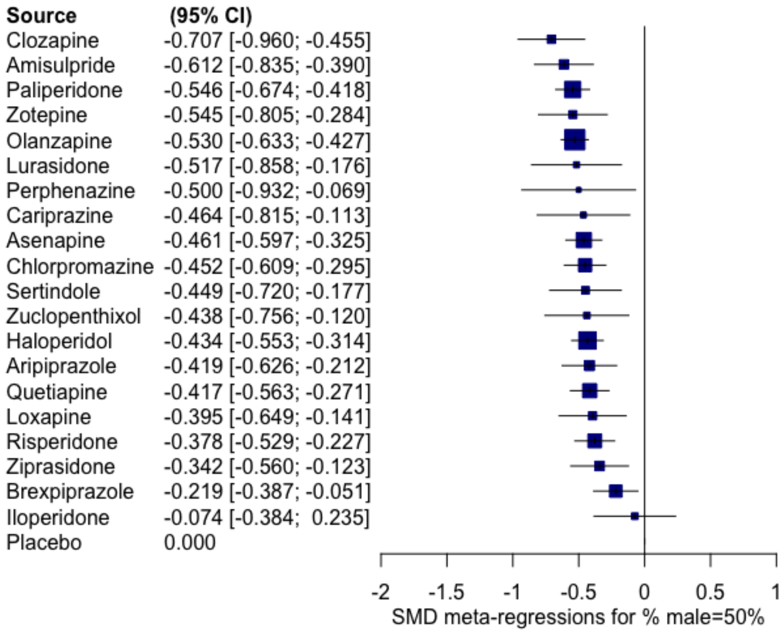

Figure 9.6: Forest plot overall change in symptoms adjusted for 50% male participants
Treatments are ranked according to standardised mean difference (SMD) compared to placebo. Treatments crossing the y-axis are not significantly different from placebo.

9.7 Sponsorship

When the model was adjusted for the situation where all drugs in a trial are sponsored or not sponsored - so that the sponsoring interests do not bias the result towards any of the arms- no significant changes in the hierarchy were observed.

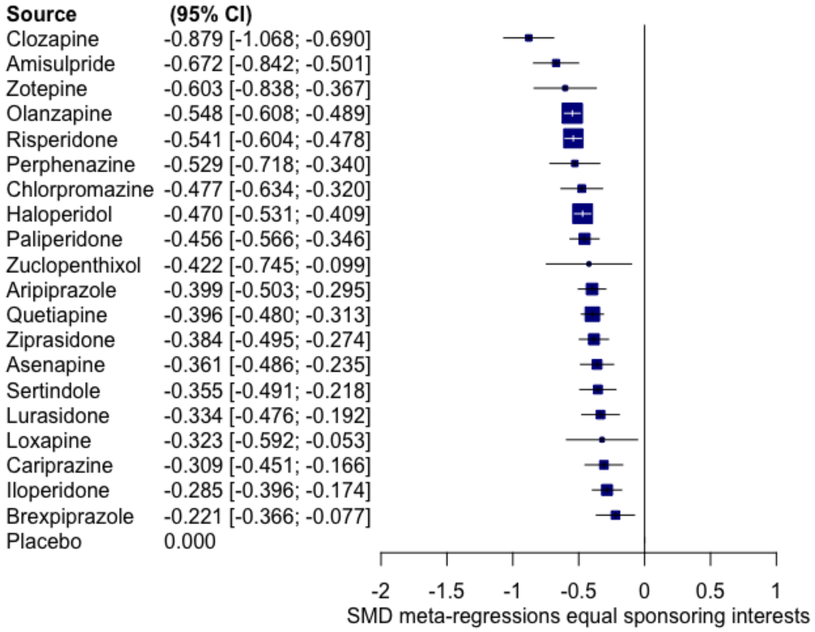

Figure 9.7: Forest plot overall change in symptoms adjusted for sponsorship
Treatments are ranked according to standardised mean difference (SMD) compared to placebo. Treatments crossing the y-axis are not significantly different from placebo.

Appendix 10: Sensitivity analyses

To check for factors that could bias the effect we removed studies due to these criteria in several sensitivity analyses.

Changes in heterogeneity

Below we present the results from the changes in heterogeneity in each sensitivity analysis.

Sensitivity analysis			
Including only studies with	τ	% of variance explained	Number of studies included
Unadjusted	0.118	-	
Low and moderate overall risk of bias	0.11	-13.10	177
Duration between 4 and 8 weeks	0.115	-5.02	165
Original standard deviations	0.112	-9.91	158
Active arms (no placebo control)	0.08	-54.04	89
Excluding placebo arms from three arm trials	0.082	-51.71	126
Intention-to-treat	0.113	-8.30	147
Fair dose (<less than 50% difference in mean dose)	0.121	5.15	108
Non-Failed Studies	0.084	-49.32	178
Publication year after 1990	0.109	-14.67	157

10.1. Removing placebo controlled trials altogether

As it can be seen from the table above to remove the placebo groups from the analysis had the most important impact on the results (approximately 55% reduction of the variance). We therefore analysed the impact of removing placebo controlled studies in two ways.

In the first approach we excluded placebo-controlled studies altogether.

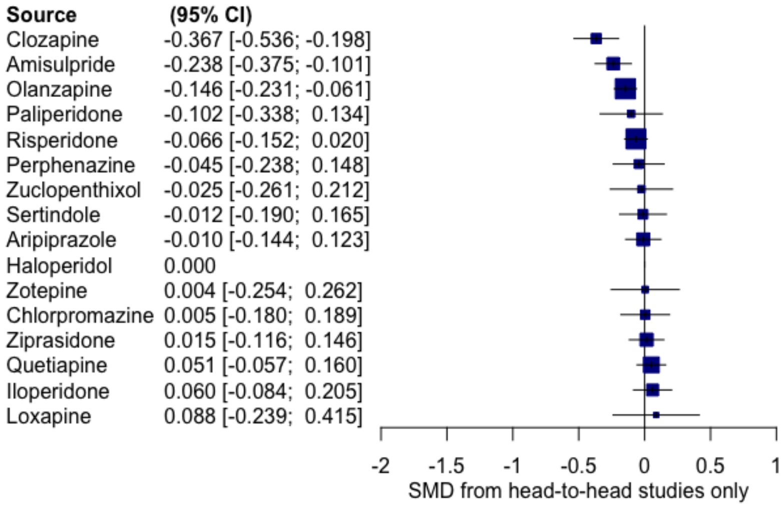

Figure 10.1: Exclusion of trials with placebo arms
All drugs are compared to haloperidol as a reference. Treatments are ranked according to standardised mean difference (SMD) compared to haloperidol. Treatments crossing the y-axis are not significantly different from placebo.

10.2. Removing only placebo arms from three or more arm trials

In the second approach we excluded only the placebo arms, but left the antipsychotic arms of three (or more) arm trials from the analysis. After excluding placebo controlled trials, the hierarchy did not change considerably.

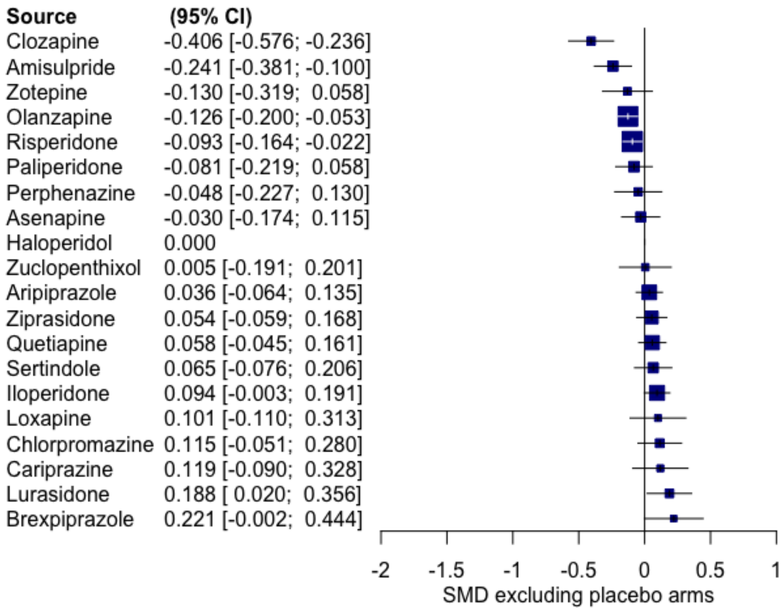

Figure 10.2: Exclusion of placebo arms in multiarm trials
All drugs are compared to haloperidol as a reference. Treatments are ranked according to standardised mean difference (SMD) compared to haloperidol.

10.3. Risk of bias

After excluding trials with high overall risk of bias, the hierarchy did not change significantly.

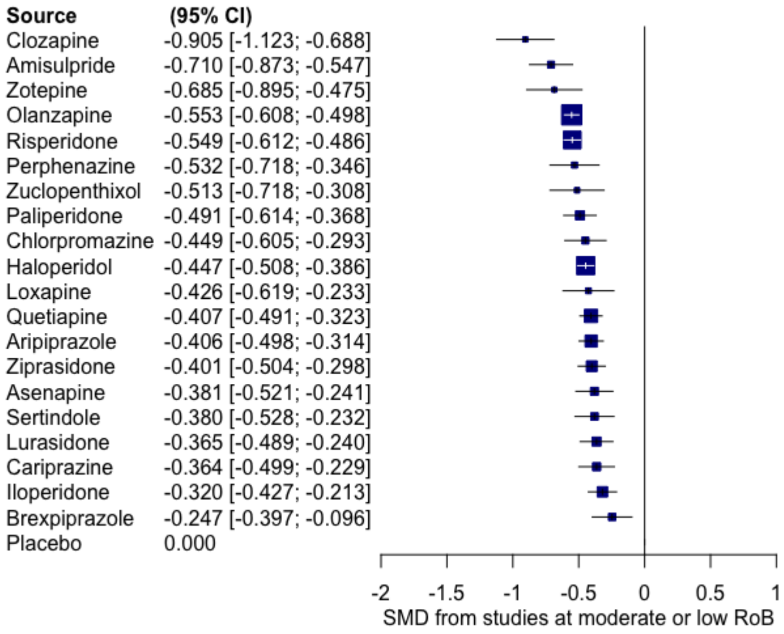

Figure 10.3: Exclusion of trials with high overall risk of bias
Treatments are ranked according to standardised mean difference (SMD) compared to placebo. Treatments crossing the y-axis are not significantly different from placebo.

10.4. Trial duration

After excluding trials with less than four or more than eight weeks duration, the hierarchy did not change significantly.

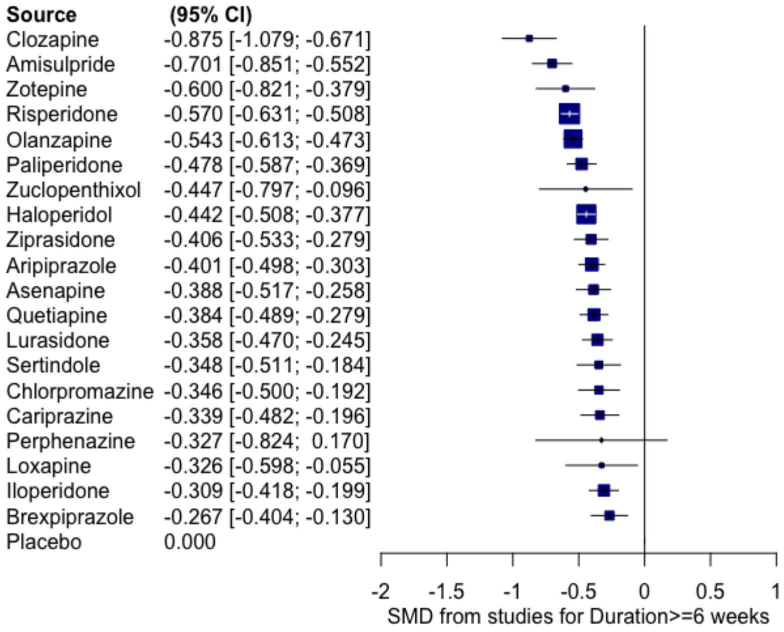

Figure 10.4: Exclusion of trials with less than four or more than eight weeks duration
Treatments are ranked according to standardised mean difference (SMD) compared to placebo. Treatments crossing the y-axis are not significantly different from placebo.

10.5. Imputed standard deviations

After excluding trials with imputed standard deviations, loxapine had a lower SMD as in the unadjusted model, but the remaining hierarchy did not change significantly.

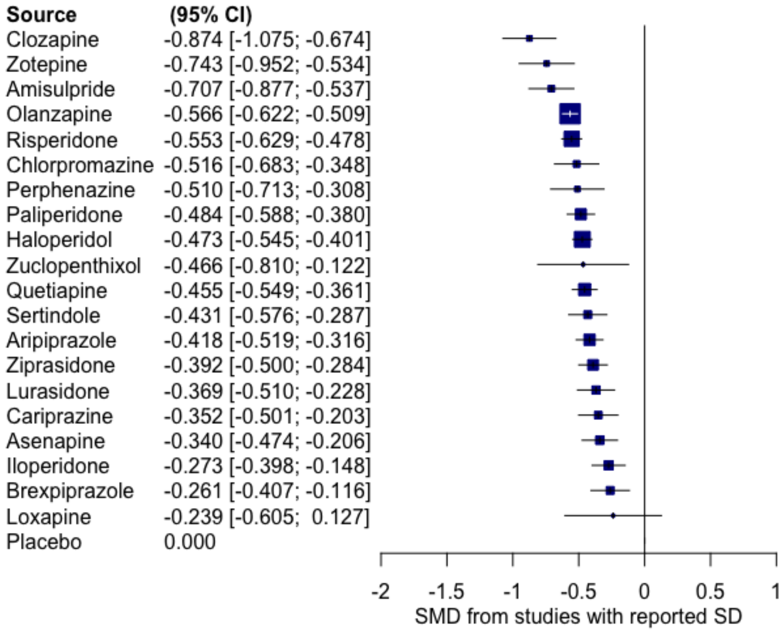

Figure 10.5: Exclusion of trials with imputed standard deviations
Treatments are ranked according to standardised mean difference (SMD) compared to placebo. Treatments crossing the y-axis are not significantly different from placebo.

10.6. Intention to treat analysis

After excluding studies, that did not use ITT, the hierarchy did not change significantly.

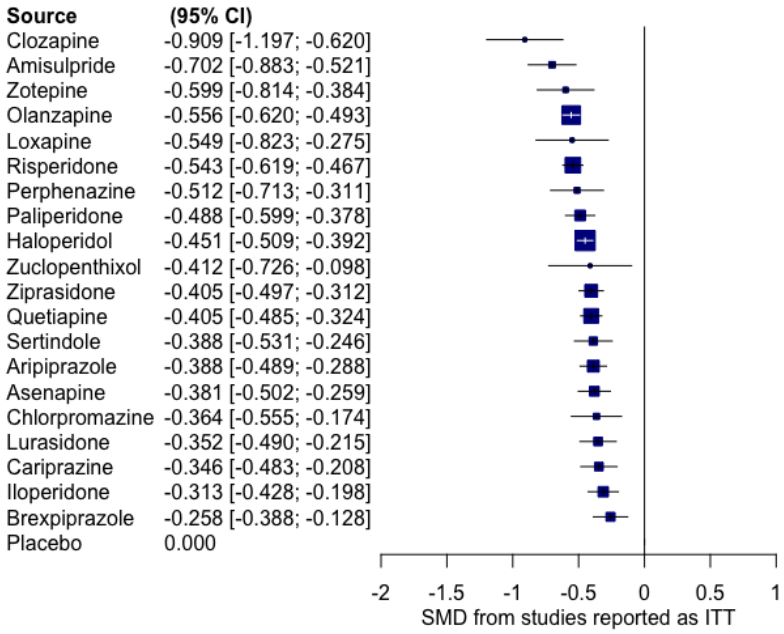

Figure 10.6: Exclusion of trials without ITT
Treatments are ranked according to standardised mean difference (SMD) compared to placebo. Treatments crossing the y-axis are not significantly different from placebo.

10.7. Unfair dose

Unfairs doses were defined as the olanzapineequivalent 50% lower or higher as the comparator. . For this analysis we excluded studies in which the lowest dose was 50% less than the largest dose olanzapinequivalents. In case a study compared haloperidol 2mg (olanzapine equivalence dose =4mg) to olanzapine 10mg we excluded the study. We calculated the mean olanzapine equivalence doses for each drug arm according to the "International Consensus Study on Antipsychotic dose".[6]

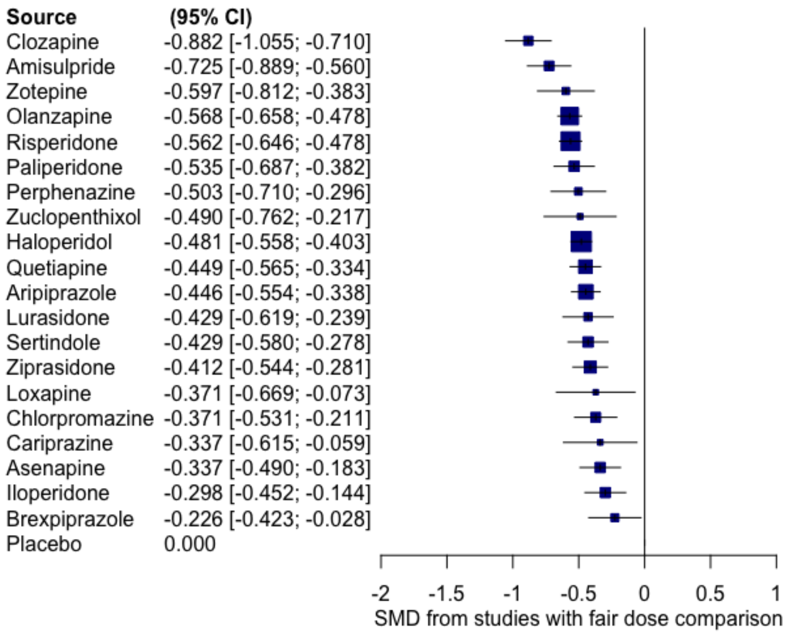

Figure 10.7: Exclusion of trials with unfair doses
Treatments are ranked according to standardised mean difference (SMD) compared to placebo. Treatments crossing the y-axis are not significantly different from placebo.

10.8. Older studies

We assumed that older studies are different concerning patient charateristics and study quality. To check if this factors influence the results considerably we excluded studies published before 1990. After excluding these studies the hierarchy did not change considerably.

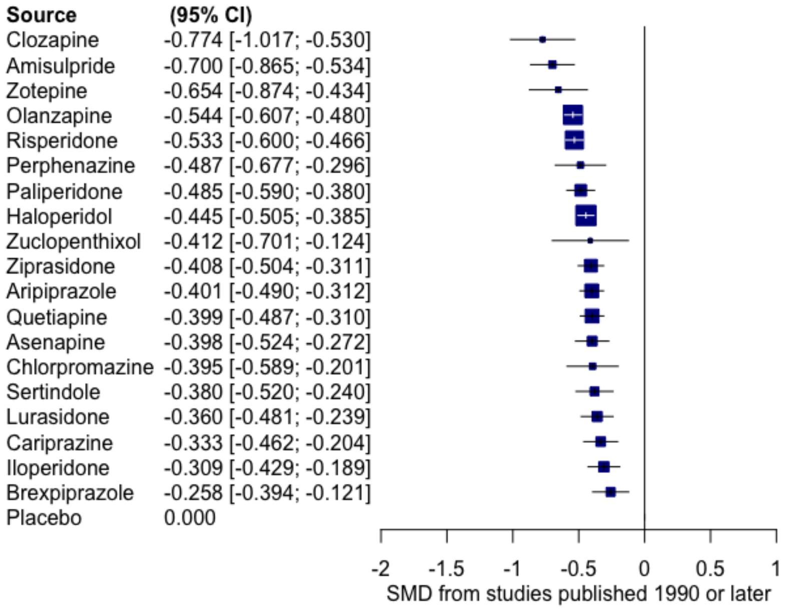

Figure 10.8: Exclusion of trials published before 1990
Treatments are ranked according to standardised mean difference (SMD) compared to placebo. Treatments crossing the y-axis are not significantly different from placebo.

10.9. Failed trials

In recent years some trials examining new compounds were termed "failed". In these trials well established efficacious compounds as olanzapine did not separate significantly from placebo. After excludsion of these trials the hierarchy did not change considerably.

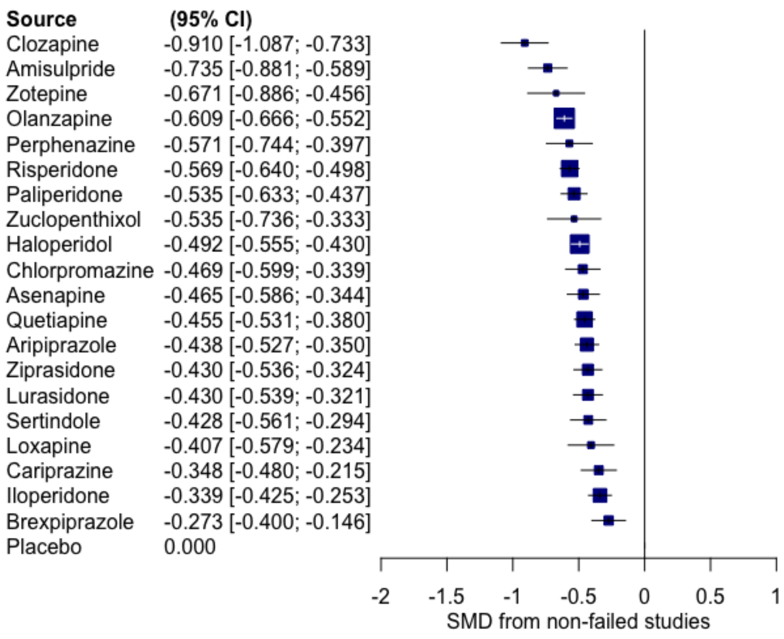

Figure 10.9: Exclusion of failed trials

Treatments are ranked according to standardised mean difference (SMD) compared to placebo. Treatments crossing the y-axis are not significantly different from placebo.

Appendix 11: League tables

Interpretation of the league tables: Drugs are reported in order of efficacy ranking. Results of the **network meta-analysis** are presented in the **left lower half** and results from **pairwise** comparisons in the **upper right half**, if available. Comparisons between treatments should be read from left to right and the estimate is in the cell in common between the column-defining treatment and the row-defining treatment. Continuous outcomes (e.g. change in overall symptoms) are reported as standard mean differences or mean differences (SMDs/MDs). In the left half SMDs/MDs lower than 0 favor the column-defining treatment, in the upper right half SMDs/MDs lower than 0 favor the row defining treatment. Dichotomous outcomes (e.g. all-cause discontinuation) are reported as risk ratios (RRs). In the left lower half, odds ratios (RRs) lower than 1 favor the column-defining treatment, in the upper right half RRs lower than 1 favor the row defining treatment. Cells in bold print indicate significant results.

Table 11.1: Positive symptoms

**AMI**	-0.04<br>-0.33, 0.25	NA	-0.15<br>-0.42, 0.12	NA	NA	-0.22<br>-0.48, 0.03	NA	NA	NA	NA	NA	NA	-0.39<br>-0.80, 0.02	NA	NA	NA	NA	NA	NA	NA	NA
-0.08<br>-0.25,0.09	**RIS**	-0.17<br>-0.90, 0.55	0.06<br>-0.12, 0.25	NA	NA	**-0.24<br>-0.40,-0.09**	0.05<br>-0.37, 0.48	-0.06<br>-0.29, 0.17	-0.18<br>-0.63, 0.27	-0.12<br>-0.30, 0.06	0.11<br>-0.26, 0.48	-0.10<br>-0.27, 0.06	NA	-0.19<br>-0.46, 0.08	NA	**-0.39<br>-0.68,-0.10**	NA	**-0.29<br>-0.50,-0.08**	**-0.68<br>-1.34,-0.02**	NA	**-0.63<br>-0.73,-0.52**
-0.06<br>-0.53,0.42	0.03<br>-0.42,0.48	**CLO**	-0.38<br>-1.13, 0.37	NA	NA	-0.00<br>-0.73, 0.72	NA	NA	NA	NA	NA	NA	NA	NA	NA	NA	NA	NA	NA	NA	NA
-0.16<br>-0.33,0	-0.08<br>-0.17,0.01	-0.1<br>-0.55,0.34	**OLA**	0.04<br>-0.11, 0.19	NA	-0.09<br>-0.24, 0.06	-0.05<br>-0.46, 0.36	-0.16<br>-0.42, 0.11	NA	**-0.26<br>-0.50,-0.01**	0.04<br>-0.25, 0.33	-0.23<br>-0.46, 0.00	0.11<br>-0.71, 0.92	-0.11<br>-0.35, 0.14	-0.22<br>-0.53, 0.08	NA	NA	NA	NA	NA	**-0.52<br>-0.62,-0.42**
-0.16<br>-0.35,0.04	-0.07<br>-0.21,0.06	-0.1<br>-0.56,0.36	0<br>-0.11,0.12	**PAL**	NA	NA	NA	NA	NA	NA	NA	NA	NA	NA	NA	NA	NA	NA	NA	NA	**-0.52<br>-0.65,-0.39**
-0.13<br>-0.48,0.22	-0.04<br>-0.36,0.27	-0.07<br>-0.62,0.48	0.03<br>-0.29,0.35	0.03<br>-0.3,0.36	**CPZ**	NA	NA	NA	NA	NA	NA	-0.17<br>-0.46, 0.12	NA	NA	NA	NA	NA	NA	NA	NA	NA
**-0.2<br>-0.37,-0.04**	**-0.12<br>-0.21,-0.04**	-0.15<br>-0.59,0.3	-0.04<br>-0.13,0.04	-0.05<br>-0.17,0.08	-0.08<br>-0.39,0.24	**HAL**	0.03<br>-0.28, 0.34	NA	NA	0.05<br>-0.20, 0.30	**-0.23<br>-0.41,-0.05**	-0.17<br>-0.36, 0.02	NA	-0.07<br>-0.33, 0.18	**-0.37<br>-0.73,-0.01**	NA	-0.28<br>-0.80, 0.23	-0.20<br>-0.53, 0.14	-0.10<br>-0.75, 0.54	NA	**-0.54<br>-0.64,-0.43**
-0.22<br>-0.44,0	-0.13<br>-0.3,0.03	-0.16<br>-0.63,0.31	-0.06<br>-0.22,0.1	-0.06<br>-0.25,0.13	-0.09<br>-0.44,0.26	-0.01<br>-0.18,0.15	**ASE**	NA	NA	NA	NA	NA	NA	NA	NA	NA	NA	NA	NA	NA	**-0.43<br>-0.59,-0.27**
-0.24<br>-0.5,0.02	-0.16<br>-0.37,0.04	-0.19<br>-0.68,0.31	-0.08<br>-0.29,0.13	-0.09<br>-0.32,0.15	-0.12<br>-0.48,0.25	-0.04<br>-0.25,0.17	-0.03<br>-0.28,0.23	**PERPH**	NA	-0.11<br>-0.40, 0.17	NA	-0.05<br>-0.32, 0.21	NA	NA	NA	NA	NA	NA	NA	NA	NA
-0.27<br>-0.75,0.22	-0.18<br>-0.64,0.27	-0.21<br>-0.84,0.43	-0.1<br>-0.57,0.36	-0.11<br>-0.58,0.37	-0.14<br>-0.69,0.42	-0.06<br>-0.52,0.4	-0.05<br>-0.53,0.44	-0.02<br>-0.52,0.48	**ZUC**	NA	NA	NA	NA	NA	NA	NA	NA	NA	NA	NA	NA
**-0.27<br>-0.46,-0.07**	**-0.18<br>-0.3,-0.07**	-0.21<br>-0.67,0.25	-0.11<br>-0.23,0.01	-0.11<br>-0.26,0.05	-0.14<br>-0.47,0.19	-0.06<br>-0.18,0.06	-0.05<br>-0.23,0.14	-0.02<br>-0.24,0.2	0<br>-0.47,0.46	**ZIP**	NA	0.06<br>-0.22, 0.34	NA	0.27<br>-0.06, 0.60	NA	NA	NA	-0.00<br>-0.29, 0.29	NA	NA	**-0.47<br>-0.65,-0.29**
**-0.29<br>-0.49,-0.08**	**-0.21<br>-0.35,-0.07**	-0.23<br>-0.69,0.24	-0.13<br>-0.27,0.01	-0.13<br>-0.3,0.04	-0.16<br>-0.5,0.18	-0.08<br>-0.22,0.05	-0.07<br>-0.27,0.13	-0.04<br>-0.29,0.2	-0.02<br>-0.5,0.45	-0.02<br>-0.19,0.14	**SER**	NA	NA	NA	NA	NA	NA	NA	NA	NA	**-0.35<br>-0.56,-0.14**
**-0.29<br>-0.47,-0.11**	**-0.21<br>-0.31,-0.11**	-0.24<br>-0.68,0.22	**-0.13<br>-0.23,-0.03**	-0.13<br>-0.27,0.01	-0.17<br>-0.47,0.14	-0.09<br>-0.19,0.01	-0.07<br>-0.25,0.1	-0.05<br>-0.26,0.17	-0.03<br>-0.49,0.44	-0.02<br>-0.16,0.11	0<br>-0.16,0.15	**QUE**	NA	NA	-0.20<br>-0.50, 0.11	NA	NA	NA	NA	-0.18<br>-0.49, 0.13	**-0.42<br>-0.54,-0.30**
-0.32<br>-0.69,0.05	-0.23<br>-0.62,0.16	-0.26<br>-0.86,0.34	-0.16<br>-0.54,0.23	-0.16<br>-0.56,0.25	-0.19<br>-0.68,0.31	-0.11<br>-0.51,0.28	-0.1<br>-0.51,0.32	-0.07<br>-0.51,0.37	-0.05<br>-0.66,0.55	-0.05<br>-0.45,0.36	-0.03<br>-0.44,0.38	-0.03<br>-0.42,0.37	**FPX**	NA	NA	NA	NA	NA	NA	NA	NA
**-0.31<br>-0.5,-0.12**	**-0.23<br>-0.34,-0.11**	-0.25<br>-0.71,0.21	**-0.15<br>-0.26,-0.04**	-0.15<br>-0.3,0	-0.18<br>-0.51,0.15	-0.11<br>-0.22,0.01	-0.09<br>-0.27,0.09	-0.07<br>-0.29,0.16	-0.04<br>-0.51,0.43	-0.04<br>-0.18,0.1	-0.02<br>-0.18,0.14	-0.02<br>-0.15,0.11	0.01<br>-0.4,0.41	**ARI**	NA	-0.01<br>-0.30, 0.28	NA	NA	NA	-0.26<br>-0.66, 0.15	**-0.32<br>-0.44,-0.19**
**-0.36<br>-0.57,-0.16**	**-0.28<br>-0.42,-0.14**	-0.31<br>-0.77,0.16	**-0.2<br>-0.34,-0.07**	**-0.21<br>-0.37,-0.04**	-0.24<br>-0.58,0.1	**-0.16<br>-0.3,-0.02**	-0.15<br>-0.34,0.05	-0.12<br>-0.36,0.12	-0.1<br>-0.57,0.38	-0.1<br>-0.26,0.06	-0.08<br>-0.26,0.1	-0.07<br>-0.22,0.07	-0.05<br>-0.46,0.36	-0.05<br>-0.21,0.1	**LUR**	NA	NA	NA	NA	NA	**-0.37<br>-0.51,-0.24**
**-0.39<br>-0.6,-0.17**	**-0.3<br>-0.46,-0.15**	-0.33<br>-0.79,0.13	**-0.23<br>-0.38,-0.08**	**-0.23<br>-0.41,-0.05**	-0.26<br>-0.6,0.08	**-0.18<br>-0.34,-0.03**	-0.17<br>-0.38,0.03	-0.14<br>-0.39,0.11	-0.12<br>-0.6,0.36	-0.12<br>-0.29,0.05	-0.1<br>-0.29,0.09	-0.1<br>-0.26,0.07	-0.07<br>-0.49,0.34	-0.08<br>-0.24,0.08	-0.02<br>-0.21,0.17	**CAR**	NA	NA	NA	NA	**-0.32<br>-0.47,-0.16**
-0.49<br>-1.02,0.04	-0.4<br>-0.92,0.11	-0.43<br>-1.1,0.26	-0.33<br>-0.84,0.19	-0.33<br>-0.85,0.19	-0.36<br>-0.96,0.24	-0.28<br>-0.79,0.23	-0.27<br>-0.8,0.26	-0.24<br>-0.8,0.31	-0.22<br>-0.9,0.46	-0.22<br>-0.74,0.3	-0.2<br>-0.72,0.33	-0.2<br>-0.71,0.33	-0.17<br>-0.82,0.47	-0.18<br>-0.7,0.35	-0.12<br>-0.65,0.41	-0.1<br>-0.63,0.44	**ZOT**	NA	NA	NA	NA
**-0.4<br>-0.6,-0.19**	**-0.31<br>-0.45,-0.17**	-0.34<br>-0.8,0.13	**-0.23<br>-0.38,-0.09**	**-0.24<br>-0.41,-0.06**	-0.27<br>-0.61,0.07	**-0.19<br>-0.33,-0.05**	-0.18<br>-0.38,0.02	-0.15<br>-0.39,0.09	-0.13<br>-0.61,0.35	-0.13<br>-0.29,0.03	-0.11<br>-0.29,0.08	-0.1<br>-0.26,0.05	-0.08<br>-0.49,0.33	-0.09<br>-0.25,0.08	-0.03<br>-0.21,0.15	-0.01<br>-0.19,0.18	0.09<br>-0.44,0.62	**ILO**	NA	NA	**-0.27<br>-0.42,-0.11**
-0.51<br>-1.09,0.08	-0.43<br>-0.99,0.14	-0.45<br>-1.17,0.26	-0.35<br>-0.92,0.22	-0.35<br>-0.93,0.22	-0.38<br>-1.02,0.25	-0.31<br>-0.87,0.26	-0.29<br>-0.88,0.29	-0.27<br>-0.87,0.34	-0.25<br>-0.97,0.48	-0.24<br>-0.82,0.33	-0.22<br>-0.8,0.36	-0.22<br>-0.79,0.35	-0.19<br>-0.89,0.5	-0.2<br>-0.77,0.38	-0.15<br>-0.73,0.44	-0.12<br>-0.71,0.46	-0.02<br>-0.78,0.73	-0.12<br>-0.7,0.46	**LEV**	NA	NA
**-0.52<br>-0.73,-0.31**	**-0.44<br>-0.59,-0.29**	-0.46<br>-0.94,0	**-0.36<br>-0.51,-0.21**	**-0.36<br>-0.54,-0.19**	**-0.39<br>-0.73,-0.05**	**-0.32<br>-0.47,-0.17**	**-0.3<br>-0.5,-0.1**	**-0.28<br>-0.52,-0.03**	-0.26<br>-0.74,0.23	**-0.25<br>-0.43,-0.08**	**-0.23<br>-0.42,-0.04**	**-0.23<br>-0.38,-0.08**	-0.2<br>-0.62,0.2	**-0.21<br>-0.37,-0.05**	-0.16<br>-0.34,0.03	-0.13<br>-0.32,0.06	-0.03<br>-0.57,0.5	-0.13<br>-0.31,0.06	-0.01<br>-0.59,0.57	**BRE**	**-0.17<br>-0.31,-0.04**
**-0.69<br>-0.86,-0.52**	**-0.61<br>-0.68,-0.54**	**-0.64<br>-1.09,-0.19**	**-0.53<br>-0.6,-0.46**	**-0.53<br>-0.65,-0.42**	**-0.57<br>-0.88,-0.25**	**-0.49<br>-0.56,-0.41**	**-0.47<br>-0.63,-0.32**	**-0.45<br>-0.66,-0.24**	-0.43<br>-0.89,0.03	**-0.43<br>-0.53,-0.32**	**-0.4<br>-0.54,-0.27**	**-0.4<br>-0.49,-0.31**	-0.38<br>-0.77,0.01	**-0.38<br>-0.48,-0.28**	**-0.33<br>-0.45,-0.2**	**-0.3<br>-0.45,-0.16**	-0.21<br>-0.72,0.31	**-0.3<br>-0.43,-0.17**	-0.18<br>-0.75,0.39	**-0.17<br>-0.31,-0.04**	**PBO**

Table 11.1: Positive symptoms
Treatments are ranked according to their surface under the curve cumulative ranking (SUCRA) for positive symptoms starting with the best. Results of the **network meta-analysis** are presented in the **left lower half** and results from **pairwise comparisons** in the **upper right** half, if available. Comparisons between treatments should be read from left to right and the estimate is in the cell in common between the column-defining treatment and the row-defining treatment. In the left lower half, standard mean differences (SMDs) lower than 0 favor the column-defining treatment, in the upper right half SMDs lower than 0 favor the row defining treatment. Cells in bold print indicate significant results NA=not available. Drug abbreviations: AMI=Amisulpride, ARI=Aripiprazole, ASE=Asenapine, BRE=Brexpiprazole, CAR=Cariprazine, CPZ=Chlorpromazine, CLO=Clozapine, FPX=Flupenthixol, HAL=Haloperidol,

ILO=Iloperidone, LEV=Levomepromazine, LUR=Lurasidone, OLA=Olanzapine, PAL=Paliperidone, PERPH=Perphenazine, PBO=Placebo, QUE=Quetiapine, RIS=Risperidone, SER=Sertindole, ZIP=Ziprasidone, ZOT=Zotepine, ZUC=Zuclopenthixol.

Table 11.2: Negative symptoms

**CLO**	NA	NA	-0.22<br>-0.96, 0.51	NA	NA	0.01<br>-0.71, 0.72	NA	NA	0.01<br>-0.36, 0.37	NA	NA	NA	NA	NA	**-0.52<br>-0.84,-0.20**	NA	NA	NA	NA	NA	-0.46<br>-1.36, 0.44
-0.08<br>-0.38,0.23	**ZOT**	NA	NA	NA	NA	NA	NA	NA	-0.39<br>-0.81, 0.03	NA	NA	NA	NA	NA	-0.16<br>-0.47, 0.15	NA	NA	NA	NA	NA	**-0.52<br>-0.94,-0.10**
-0.11<br>-0.37,0.14	-0.04<br>-0.29,0.22	**AMI**	-0.00<br>-0.25, 0.24	NA	NA	-0.14<br>-0.42, 0.13	NA	NA	NA	NA	NA	NA	NA	NA	**-0.24<br>-0.43,-0.04**	NA	NA	NA	NA	**-0.44<br>-0.83,-0.05**	NA
-0.17<br>-0.39,0.06	-0.09<br>-0.32,0.15	-0.05<br>-0.18,0.08	**OLA**	0.10<br>-0.28, 0.48	0.01<br>-0.22, 0.24	-0.14<br>-0.30, 0.03	-0.07<br>-0.21, 0.06	-0.03<br>-0.29, 0.23	NA	-0.03<br>-0.25, 0.19	-0.10<br>-0.31, 0.12	NA	-0.09<br>-0.29, 0.11	-0.10<br>-0.38, 0.17	**-0.18<br>-0.32,-0.05**	NA	NA	NA	NA	-0.23<br>-1.04, 0.57	**-0.46<br>-0.55,-0.37**
-0.19<br>-0.45,0.06	-0.12<br>-0.38,0.14	-0.08<br>-0.26,0.1	-0.03<br>-0.16,0.11	**ASE**	NA	-0.38<br>-0.79, 0.02	NA	NA	NA	NA	NA	NA	NA	NA	-0.08<br>-0.36, 0.21	NA	NA	NA	NA	NA	**-0.36<br>-0.51,-0.21**
-0.2<br>-0.47,0.08	-0.12<br>-0.4,0.16	-0.08<br>-0.29,0.12	-0.03<br>-0.2,0.14	0<br>-0.21,0.21	**PERPH**	-0.10<br>-0.31, 0.10	NA	NA	NA	-0.03<br>-0.28, 0.22	NA	NA	-0.12<br>-0.35, 0.11	NA	NA	NA	NA	NA	NA	NA	NA
**-0.25<br>-0.47,-0.02**	-0.17<br>-0.41,0.06	-0.14<br>-0.27,0	**-0.08<br>-0.15,-0.01**	-0.06<br>-0.19,0.08	-0.05<br>-0.22,0.12	**RIS**	NA	0.21<br>-0.13, 0.55	NA	0.03<br>-0.13, 0.19	0.08<br>-0.17, 0.33	-0.15<br>-0.41, 0.10	0.04<br>-0.10, 0.19	NA	**-0.15<br>-0.28,-0.02**	-0.19<br>-0.62, 0.24	NA	-0.42<br>-1.06, 0.21	-0.16<br>-0.35, 0.03	NA	**-0.41<br>-0.50,-0.32**
-0.25<br>-0.49,0	-0.17<br>-0.41,0.08	-0.13<br>-0.29,0.03	-0.08<br>-0.18,0.02	-0.05<br>-0.21,0.11	-0.05<br>-0.24,0.14	0<br>-0.11,0.12	**PAL**	NA	NA	NA	NA	NA	NA	NA	NA	NA	NA	NA	NA	NA	**-0.39<br>-0.51,-0.27**
-0.25<br>-0.5,0	-0.17<br>-0.42,0.08	-0.13<br>-0.3,0.03	-0.08<br>-0.2,0.04	-0.05<br>-0.22,0.11	-0.05<br>-0.25,0.15	0<br>-0.12,0.12	0<br>-0.15,0.14	**SER**	NA	NA	NA	NA	NA	NA	-0.07<br>-0.24, 0.09	NA	NA	NA	NA	NA	**-0.24<br>-0.44,-0.05**
**-0.27<br>-0.51,-0.03**	-0.19<br>-0.45,0.06	-0.16<br>-0.36,0.05	-0.1<br>-0.27,0.07	-0.08<br>-0.28,0.13	-0.08<br>-0.3,0.16	-0.02<br>-0.19,0.15	-0.03<br>-0.21,0.16	-0.02<br>-0.22,0.18	**CPZ**	NA	NA	NA	-0.02<br>-0.22, 0.18	NA	NA	NA	NA	NA	NA	NA	-0.17<br>-0.52, 0.18
**-0.28<br>-0.52,-0.04**	-0.2<br>-0.45,0.04	**-0.17<br>-0.33,-0.01**	**-0.11<br>-0.22,-0.01**	-0.09<br>-0.25,0.07	-0.09<br>-0.27,0.09	-0.03<br>-0.13,0.07	-0.04<br>-0.17,0.1	-0.03<br>-0.18,0.11	-0.01<br>-0.2,0.17	**ZIP**	NA	NA	-0.09<br>-0.34, 0.15	NA	0.05<br>-0.20, 0.30	NA	NA	NA	-0.02<br>-0.28, 0.24	NA	**-0.32<br>-0.49,-0.16**
**-0.29<br>-0.53,-0.05**	-0.21<br>-0.46,0.03	**-0.18<br>-0.33,-0.02**	**-0.12<br>-0.22,-0.02**	-0.1<br>-0.25,0.06	-0.09<br>-0.28,0.09	-0.04<br>-0.14,0.06	-0.05<br>-0.17,0.08	-0.04<br>-0.18,0.1	-0.02<br>-0.2,0.16	-0.01<br>-0.13,0.12	**ARI**	0.08<br>-0.18, 0.33	NA	NA	-0.05<br>-0.27, 0.16	NA	0.16<br>-0.22, 0.55	NA	NA	NA	**-0.31<br>-0.42,-0.19**
**-0.29<br>-0.54,-0.04**	-0.22<br>-0.48,0.04	**-0.18<br>-0.36,-0.01**	-0.13<br>-0.26,0	-0.1<br>-0.27,0.07	-0.1<br>-0.3,0.1	-0.04<br>-0.17,0.08	-0.05<br>-0.2,0.1	-0.05<br>-0.21,0.12	-0.02<br>-0.22,0.17	-0.01<br>-0.16,0.14	0<br>-0.14,0.13	**CAR**	NA	NA	NA	NA	NA	NA	NA	NA	**-0.34<br>-0.48,-0.21**
**-0.31<br>-0.53,-0.08**	-0.23<br>-0.47,0.01	**-0.19<br>-0.34,-0.05**	**-0.14<br>-0.23,-0.05**	-0.11<br>-0.26,0.03	-0.11<br>-0.28,0.06	-0.06<br>-0.14,0.03	-0.06<br>-0.18,0.06	-0.06<br>-0.19,0.08	-0.04<br>-0.19,0.12	-0.02<br>-0.14,0.09	-0.02<br>-0.13,0.1	-0.01<br>-0.15,0.12	**QUE**	-0.02<br>-0.30, 0.25	0.10<br>-0.07, 0.28	NA	-0.16<br>-0.44, 0.12	NA	NA	NA	**-0.32<br>-0.43,-0.21**
**-0.33<br>-0.58,-0.08**	-0.25<br>-0.5,0	**-0.22<br>-0.38,-0.05**	**-0.16<br>-0.28,-0.04**	-0.13<br>-0.3,0.03	-0.13<br>-0.33,0.07	-0.08<br>-0.2,0.04	-0.08<br>-0.23,0.06	-0.08<br>-0.24,0.07	-0.06<br>-0.25,0.13	-0.05<br>-0.19,0.09	-0.04<br>-0.17,0.09	-0.03<br>-0.19,0.12	-0.02<br>-0.15,0.1	**LUR**	0.11<br>-0.22, 0.44	NA	NA	NA	NA	NA	**-0.31<br>-0.43,-0.18**
**-0.33<br>-0.55,-0.11**	**-0.25<br>-0.48,-0.02**	**-0.21<br>-0.34,-0.09**	**-0.16<br>-0.23,-0.09**	-0.13<br>-0.27,0	-0.13<br>-0.3,0.04	**-0.08<br>-0.15,-0.01**	-0.08<br>-0.19,0.03	-0.08<br>-0.19,0.04	-0.06<br>-0.23,0.11	-0.05<br>-0.15,0.06	-0.04<br>-0.14,0.06	-0.03<br>-0.16,0.09	-0.02<br>-0.11,0.06	0<br>-0.12,0.12	**HAL**	NA	NA	-0.01<br>-0.65, 0.62	-0.13<br>-0.43, 0.18	NA	**-0.29<br>-0.39,-0.20**
-0.44<br>-0.91,0.03	-0.36<br>-0.83,0.1	-0.33<br>-0.76,0.1	-0.28<br>-0.69,0.14	-0.25<br>-0.68,0.18	-0.25<br>-0.68,0.19	-0.19<br>-0.6,0.22	-0.2<br>-0.62,0.23	-0.19<br>-0.62,0.23	-0.17<br>-0.61,0.27	-0.16<br>-0.58,0.26	-0.15<br>-0.57,0.27	-0.15<br>-0.57,0.27	-0.14<br>-0.55,0.28	-0.11<br>-0.53,0.31	-0.11<br>-0.52,0.3	**ZUC**	NA	NA	NA	NA	NA
**-0.37<br>-0.62,-0.12**	**-0.29<br>-0.55,-0.04**	**-0.25<br>-0.42,-0.08**	**-0.2<br>-0.32,-0.08**	-0.17<br>-0.34,0	-0.17<br>-0.37,0.03	-0.12<br>-0.24,0	-0.12<br>-0.27,0.02	-0.12<br>-0.28,0.04	-0.1<br>-0.29,0.09	-0.09<br>-0.23,0.06	-0.08<br>-0.21,0.06	-0.07<br>-0.23,0.09	-0.06<br>-0.19,0.07	-0.04<br>-0.19,0.11	-0.04<br>-0.17,0.08	0.07<br>-0.35,0.5	**BRE**	NA	NA	NA	**-0.24<br>-0.36,-0.12**
-0.52<br>-1.06,0.01	-0.44<br>-0.98,0.09	-0.41<br>-0.92,0.09	-0.35<br>-0.85,0.12	-0.33<br>-0.83,0.17	-0.33<br>-0.85,0.17	-0.27<br>-0.76,0.2	-0.28<br>-0.77,0.2	-0.27<br>-0.78,0.21	-0.25<br>-0.76,0.25	-0.24<br>-0.74,0.24	-0.23<br>-0.74,0.25	-0.23<br>-0.73,0.26	-0.22<br>-0.71,0.26	-0.19<br>-0.7,0.29	-0.19<br>-0.68,0.28	-0.08<br>-0.7,0.54	-0.15<br>-0.66,0.33	**LEV**	NA	NA	NA
**-0.39<br>-0.65,-0.15**	**-0.32<br>-0.57,-0.07**	**-0.28<br>-0.45,-0.11**	**-0.23<br>-0.35,-0.11**	**-0.2<br>-0.37,-0.03**	-0.2<br>-0.39,0	**-0.15<br>-0.26,-0.03**	**-0.15<br>-0.3,-0.01**	-0.15<br>-0.3,0.01	-0.12<br>-0.32,0.07	-0.11<br>-0.25,0.02	-0.1<br>-0.24,0.03	-0.1<br>-0.26,0.06	-0.09<br>-0.22,0.04	-0.07<br>-0.22,0.09	-0.07<br>-0.19,0.05	0.05<br>-0.38,0.47	-0.03<br>-0.18,0.13	0.13<br>-0.36,0.62	**ILO**	NA	**-0.21<br>-0.35,-0.08**
**-0.52<br>-0.93,-0.11**	**-0.44<br>-0.86,-0.03**	**-0.41<br>-0.74,-0.07**	-0.35<br>-0.7,0	-0.33<br>-0.69,0.04	-0.33<br>-0.7,0.06	-0.27<br>-0.62,0.08	-0.28<br>-0.63,0.09	-0.27<br>-0.63,0.1	-0.25<br>-0.63,0.14	-0.24<br>-0.6,0.12	-0.23<br>-0.58,0.13	-0.23<br>-0.59,0.14	-0.21<br>-0.57,0.14	-0.19<br>-0.55,0.17	-0.19<br>-0.53,0.16	-0.08<br>-0.62,0.46	-0.15<br>-0.51,0.22	0<br>-0.58,0.62	-0.13<br>-0.49,0.24	**FPX**	NA
**-0.62<br>-0.84,-0.39**	**-0.54<br>-0.77,-0.31**	**-0.5<br>-0.64,-0.37**	**-0.45<br>-0.51,-0.39**	**-0.42<br>-0.55,-0.29**	**-0.42<br>-0.59,-0.25**	**-0.37<br>-0.43,-0.31**	**-0.37<br>-0.47,-0.28**	**-0.37<br>-0.48,-0.25**	**-0.35<br>-0.51,-0.18**	**-0.33<br>-0.43,-0.23**	**-0.33<br>-0.41,-0.24**	**-0.32<br>-0.44,-0.2**	**-0.31<br>-0.38,-0.24**	**-0.29<br>-0.39,-0.18**	**-0.29<br>-0.35,-0.23**	-0.17<br>-0.59,0.23	**-0.25<br>-0.36,-0.14**	-0.09<br>-0.57,0.4	**-0.22<br>-0.33,-0.11**	-0.1<br>-0.45,0.25	**PBO**

Table 11.2: Negative symptoms
Treatments are ranked according to their surface under the curve cumulative ranking (SUCRA) for negative symptoms starting with the best. Results of the **network meta-analysis** are presented in the **left lower half** and results from **pairwise comparisons** in the **upper right** half, if available. Comparisons between treatments should be read from left to right and the estimate is in the cell in common between the column-defining treatment and the row-defining treatment. In the left lower half, standard mean differences (SMDs) lower than 0 favor the column-defining treatment, in the upper right half SMDs lower than 0 favor the row defining treatment. Cells in bold print indicate significant results NA=not available. Drug abbreviations: AMI=Amisulpride, ARI=Aripiprazole, ASE=Asenapine, BRE=Brexpiprazole, CAR=Cariprazine, CPZ=Chlorpromazine, CLO=Clozapine, FPX=Flupenthixol, HAL=Haloperidol,

ILO=Iloperidone, LEV=Levomepromazine, LUR=Lurasidone, OLA=Olanzapine, PAL=Paliperidone, PERPH=Perphenazine, PBO=Placebo, QUE=Quetiapine, RIS=Risperidone, SER=Sertindole, ZIP=Ziprasidone, ZOT=Zotepine, ZUC=Zuclopenthixol.

Table 11.3: Depressive symptoms

SUL	NA	NA	NA	NA	NA	NA	NA	NA	NA	NA	NA	NA	NA	NA	NA	NA	NA	NA	NA	**-0.73<br>-1.16,-0.29**	NA	NA	NA	NA	NA	NA	NA	NA
-0.39<br>-0.89,0.11	CLO	NA	0.09<br>-0.65, 0.83	NA	NA	NA	NA	NA	NA	NA	NA	NA	NA	NA	NA	NA	NA	NA	NA	-0.25<br>-0.62, 0.13	**-0.60<br>-1.12,-0.09**	NA	NA	NA	NA	NA	NA	-0.39<br>-1.28, 0.51
-0.46<br>-0.95,0.02	-0.08<br>-0.41,0.25	AMI	0.04<br>-0.24, 0.31	NA	NA	NA	NA	NA	NA	NA	-0.13<br>-0.44, 0.19	NA	NA	NA	NA	NA	NA	NA	NA	NA	**-0.36<br>-0.56,-0.16**	NA	NA	NA	NA	NA	**-0.48<br>-0.87,-0.09**	NA
**-0.53<br>-0.99,-0.06**	-0.14<br>-0.44,0.15	-0.07<br>-0.23,0.09	OLA	NA	NA	-0.08<br>-0.23, 0.07	0.08<br>-0.31, 0.47	NA	NA	NA	-0.04<br>-0.31, 0.23	NA	NA	-0.14<br>-0.41, 0.12	NA	-0.27<br>-0.55, 0.02	NA	NA	NA	NA	**-0.23<br>-0.37,-0.09**	NA	NA	NA	NA	NA	NA	**-0.35<br>-0.46,-0.24**
-0.5<br>-1.04,0.03	-0.11<br>-0.53,0.31	-0.04<br>-0.37,0.29	0.03<br>-0.28,0.33	ARI	-0.04<br>-0.31, 0.23	NA	NA	NA	NA	NA	NA	NA	NA	NA	NA	NA	NA	NA	NA	NA	NA	NA	NA	NA	NA	NA	NA	**-0.41<br>-0.70,-0.11**
**-0.54<br>-1.07,-0.01**	-0.15<br>-0.56,0.25	-0.08<br>-0.39,0.23	-0.01<br>-0.29,0.27	-0.04<br>-0.31,0.23	CAR	NA	NA	NA	NA	NA	NA	NA	NA	NA	NA	NA	NA	NA	NA	NA	NA	NA	NA	NA	NA	NA	NA	**-0.37<br>-0.63,-0.10**
**-0.58<br>-1.05,-0.1**	-0.19<br>-0.51,0.12	-0.11<br>-0.31,0.07	-0.05<br>-0.17,0.07	-0.08<br>-0.39,0.24	-0.04<br>-0.33,0.26	PAL	NA	NA	NA	NA	NA	NA	NA	NA	NA	NA	NA	NA	NA	NA	NA	NA	NA	NA	NA	NA	NA	**-0.36<br>-0.48,-0.24**
**-0.58<br>-1.07,-0.11**	-0.2<br>-0.53,0.13	-0.12<br>-0.34,0.09	-0.06<br>-0.22,0.11	-0.08<br>-0.41,0.24	-0.04<br>-0.35,0.26	-0.01<br>-0.2,0.18	ASE	NA	NA	NA	NA	NA	NA	NA	NA	NA	NA	NA	NA	NA	-0.09<br>-0.39, 0.20	NA	NA	NA	NA	NA	NA	**-0.29<br>-0.45,-0.12**
-0.64<br>-1.29,0.01	-0.25<br>-0.79,0.3	-0.18<br>-0.68,0.34	-0.11<br>-0.61,0.39	-0.14<br>-0.72,0.43	-0.1<br>-0.66,0.47	-0.06<br>-0.57,0.45	-0.06<br>-0.57,0.46	ZOT	-0.01<br>-0.66, 0.64	NA	NA	NA	NA	NA	NA	NA	-0.08<br>-0.53, 0.36	NA	NA	NA	-0.10<br>-0.75, 0.54	NA	NA	NA	NA	NA	NA	NA
-0.65<br>-1.53,0.25	-0.26<br>-1.09,0.57	-0.18<br>-0.99,0.62	-0.12<br>-0.92,0.68	-0.15<br>-1,0.7	-0.11<br>-0.95,0.74	-0.07<br>-0.88,0.73	-0.06<br>-0.87,0.75	-0.01<br>-0.65,0.61	PERA	NA	NA	NA	NA	NA	NA	NA	NA	NA	NA	NA	NA	NA	NA	NA	NA	NA	NA	NA
**-0.67<br>-1.12,-0.21**	-0.28<br>-0.59,0.01	**-0.21<br>-0.4,-0.02**	**-0.14<br>-0.27,-0.01**	-0.17<br>-0.48,0.14	-0.13<br>-0.42,0.16	-0.09<br>-0.25,0.06	-0.08<br>-0.27,0.1	-0.03<br>-0.53,0.47	-0.02<br>-0.82,0.78	QUE	-0.06<br>-0.29, 0.18	NA	NA	NA	NA	-0.02<br>-0.30, 0.27	NA	NA	NA	-0.04<br>-0.31, 0.23	NA	-0.16<br>-0.45, 0.13	NA	NA	NA	NA	NA	**-0.24<br>-0.37,-0.12**
**-0.68<br>-1.15,-0.21**	-0.29<br>-0.6,0.01	**-0.21<br>-0.39,-0.05**	**-0.15<br>-0.27,-0.03**	-0.18<br>-0.49,0.15	-0.14<br>-0.43,0.15	-0.1<br>-0.26,0.05	-0.09<br>-0.28,0.09	-0.04<br>-0.54,0.46	-0.03<br>-0.83,0.77	-0.01<br>-0.15,0.13	RIS	0.00<br>-0.43, 0.43	NA	-0.20<br>-0.51, 0.10	NA	NA	NA	NA	0.14<br>-0.29, 0.58	NA	-0.04<br>-0.24, 0.16	NA	NA	NA	NA	NA	NA	-0.13<br>-0.39, 0.13
**-0.68<br>-1.31,-0.05**	-0.29<br>-0.82,0.23	-0.22<br>-0.68,0.24	-0.15<br>-0.59,0.29	-0.18<br>-0.7,0.34	-0.14<br>-0.65,0.37	-0.11<br>-0.55,0.35	-0.1<br>-0.56,0.37	-0.04<br>-0.7,0.62	-0.03<br>-0.94,0.88	-0.01<br>-0.46,0.44	0<br>-0.43,0.42	PERPH	NA	NA	NA	NA	NA	NA	NA	NA	NA	NA	NA	NA	NA	NA	NA	NA
-0.7<br>-1.47,0.05	-0.32<br>-1.01,0.38	-0.24<br>-0.93,0.44	-0.18<br>-0.85,0.5	-0.2<br>-0.94,0.52	-0.16<br>-0.88,0.56	-0.13<br>-0.8,0.54	-0.12<br>-0.81,0.56	-0.06<br>-0.87,0.76	-0.06<br>-1.08,0.97	-0.04<br>-0.7,0.64	-0.03<br>-0.7,0.65	-0.02<br>-0.83,0.77	PIM	NA	NA	NA	NA	NA	NA	-0.03<br>-0.67, 0.62	NA	NA	NA	NA	NA	NA	NA	NA
**-0.69<br>-1.17,-0.21**	-0.31<br>-0.64,0.02	**-0.23<br>-0.43,-0.03**	**-0.16<br>-0.32,-0.01**	-0.19<br>-0.52,0.14	-0.15<br>-0.46,0.15	-0.12<br>-0.3,0.06	-0.11<br>-0.31,0.1	-0.05<br>-0.56,0.45	-0.05<br>-0.85,0.76	-0.03<br>-0.2,0.15	-0.02<br>-0.18,0.15	-0.01<br>-0.46,0.44	0.01<br>-0.67,0.69	ZIP	NA	NA	NA	NA	NA	NA	-0.03<br>-0.35, 0.30	NA	NA	NA	NA	NA	NA	**-0.31<br>-0.53,-0.09**
-0.73<br>-1.58,0.1	-0.34<br>-1.14,0.45	-0.26<br>-1.05,0.51	-0.2<br>-0.98,0.57	-0.23<br>-1.05,0.57	-0.19<br>-1.01,0.62	-0.15<br>-0.94,0.62	-0.14<br>-0.93,0.64	-0.09<br>-0.97,0.8	-0.08<br>-1.17,0.99	-0.06<br>-0.83,0.71	-0.05<br>-0.83,0.72	-0.04<br>-0.92,0.83	-0.02<br>-1.01,0.93	-0.03<br>-0.82,0.74	PEN	NA	NA	NA	NA	0.00<br>-0.75, 0.75	NA	NA	NA	NA	NA	NA	NA	NA
**-0.7<br>-1.18,-0.23**	**-0.31<br>-0.63,-0.01**	**-0.24<br>-0.43,-0.05**	**-0.17<br>-0.3,-0.04**	-0.2<br>-0.51,0.12	-0.16<br>-0.45,0.13	-0.12<br>-0.28,0.03	-0.12<br>-0.31,0.07	-0.06<br>-0.57,0.45	-0.05<br>-0.85,0.75	-0.03<br>-0.18,0.12	-0.02<br>-0.18,0.14	-0.02<br>-0.47,0.43	0<br>-0.68,0.68	-0.01<br>-0.19,0.17	0.03<br>-0.74,0.81	LUR	NA	NA	NA	NA	0.11<br>-0.23, 0.45	NA	NA	NA	NA	NA	NA	**-0.23<br>-0.36,-0.11**
**-0.73<br>-1.35,-0.09**	-0.34<br>-0.89,0.2	-0.27<br>-0.77,0.24	-0.2<br>-0.68,0.29	-0.23<br>-0.8,0.35	-0.19<br>-0.75,0.37	-0.16<br>-0.65,0.34	-0.15<br>-0.65,0.37	-0.09<br>-0.48,0.31	-0.08<br>-0.82,0.66	-0.06<br>-0.55,0.43	-0.05<br>-0.55,0.44	-0.05<br>-0.7,0.59	-0.03<br>-0.83,0.77	-0.04<br>-0.54,0.46	-0.01<br>-0.88,0.87	-0.03<br>-0.53,0.47	THIOTH	NA	NA	0.02<br>-0.58, 0.62	NA	NA	NA	NA	NA	NA	NA	NA
**-0.74<br>-1.34,-0.12**	-0.35<br>-0.85,0.17	-0.27<br>-0.74,0.19	-0.21<br>-0.65,0.24	-0.24<br>-0.76,0.29	-0.2<br>-0.71,0.32	-0.16<br>-0.61,0.29	-0.15<br>-0.61,0.31	-0.09<br>-0.74,0.55	-0.09<br>-0.99,0.81	-0.07<br>-0.51,0.38	-0.06<br>-0.51,0.39	-0.05<br>-0.67,0.57	-0.03<br>-0.81,0.75	-0.04<br>-0.51,0.41	-0.01<br>-0.85,0.86	-0.03<br>-0.49,0.42	0<br>-0.63,0.64	TRIFLU	NA	NA	0.38<br>-0.50, 1.26	NA	NA	-0.11<br>-0.61, 0.39	NA	NA	NA	-0.49<br>-1.32, 0.35
**-0.74<br>-1.33,-0.16**	-0.35<br>-0.82,0.11	-0.28<br>-0.67,0.11	-0.21<br>-0.58,0.16	-0.24<br>-0.72,0.23	-0.2<br>-0.66,0.25	-0.16<br>-0.55,0.22	-0.16<br>-0.55,0.24	-0.1<br>-0.73,0.51	-0.09<br>-0.96,0.78	-0.07<br>-0.45,0.31	-0.06<br>-0.42,0.3	-0.06<br>-0.61,0.49	-0.03<br>-0.8,0.72	-0.05<br>-0.44,0.34	-0.01<br>-0.85,0.84	-0.04<br>-0.43,0.35	-0.01<br>-0.63,0.6	0<br>-0.59,0.56	ZUC	NA	0.46<br>-0.21, 1.13	NA	NA	NA	NA	NA	NA	NA
**-0.73<br>-1.15,-0.31**	**-0.34<br>-0.63,-0.06**	**-0.26<br>-0.51,-0.02**	-0.2<br>-0.41,0.01	-0.23<br>-0.58,0.13	-0.19<br>-0.52,0.14	-0.15<br>-0.38,0.08	-0.14<br>-0.39,0.11	-0.09<br>-0.58,0.4	-0.08<br>-0.88,0.71	-0.06<br>-0.26,0.14	-0.05<br>-0.27,0.17	-0.05<br>-0.52,0.42	-0.02<br>-0.66,0.61	-0.03<br>-0.28,0.21	0<br>-0.73,0.74	-0.03<br>-0.25,0.2	0<br>-0.47,0.47	0.01<br>-0.45,0.47	0.01<br>-0.41,0.43	CPZ	-0.00<br>-0.81, 0.81	NA	0.00<br>-0.75, 0.75	0.05<br>-0.35, 0.44	-0.06<br>-0.86, 0.74	NA	NA	0.01<br>-0.43, 0.44
**-0.73<br>-1.19,-0.26**	**-0.35<br>-0.64,-0.06**	**-0.27<br>-0.42,-0.12**	**-0.2<br>-0.3,-0.11**	-0.23<br>-0.54,0.08	-0.19<br>-0.47,0.09	**-0.16<br>-0.29,-0.02**	-0.15<br>-0.32,0.02	-0.09<br>-0.59,0.4	-0.09<br>-0.88,0.72	-0.07<br>-0.19,0.07	-0.06<br>-0.17,0.06	-0.05<br>-0.49,0.39	-0.03<br>-0.7,0.64	-0.04<br>-0.19,0.12	-0.01<br>-0.77,0.77	-0.03<br>-0.17,0.11	0<br>-0.49,0.48	0<br>-0.43,0.44	0.01<br>-0.36,0.38	-0.01<br>-0.21,0.2	HAL	NA	NA	-0.23<br>-0.89, 0.43	-0.05<br>-0.80, 0.70	-0.10<br>-0.31, 0.11	NA	**-0.19<br>-0.32,-0.05**
**-0.74<br>-1.22,-0.26**	**-0.35<br>-0.68,-0.04**	**-0.28<br>-0.49,-0.07**	**-0.21<br>-0.37,-0.06**	-0.24<br>-0.56,0.08	-0.2<br>-0.5,0.1	-0.17<br>-0.34,0	-0.16<br>-0.36,0.04	-0.1<br>-0.61,0.4	-0.1<br>-0.89,0.71	-0.07<br>-0.24,0.09	-0.07<br>-0.24,0.11	-0.06<br>-0.52,0.4	-0.04<br>-0.72,0.64	-0.05<br>-0.25,0.15	-0.02<br>-0.79,0.77	-0.04<br>-0.22,0.13	-0.01<br>-0.52,0.49	-0.01<br>-0.47,0.45	0<br>-0.39,0.39	-0.02<br>-0.25,0.23	-0.01<br>-0.17,0.15	BRE	NA	NA	NA	NA	NA	**-0.17<br>-0.31,-0.04**
**-0.84<br>-1.6,-0.1**	-0.45<br>-1.13,0.22	-0.38<br>-1.03,0.27	-0.31<br>-0.95,0.32	-0.34<br>-1.05,0.36	-0.3<br>-1.01,0.38	-0.26<br>-0.91,0.37	-0.26<br>-0.92,0.38	-0.2<br>-0.99,0.59	-0.19<br>-1.19,0.81	-0.17<br>-0.82,0.46	-0.16<br>-0.81,0.47	-0.16<br>-0.93,0.6	-0.14<br>-1.04,0.76	-0.15<br>-0.8,0.49	-0.11<br>-1.08,0.86	-0.14<br>-0.79,0.49	-0.11<br>-0.89,0.67	-0.1<br>-0.87,0.66	-0.1<br>-0.83,0.63	-0.11<br>-0.75,0.51	-0.11<br>-0.75,0.52	-0.1<br>-0.75,0.54	MOL	NA	NA	NA	NA	0.04<br>-0.71, 0.79
**-0.78<br>-1.29,-0.27**	**-0.39<br>-0.77,-0.01**	-0.32<br>-0.65,0	-0.25<br>-0.55,0.04	-0.28<br>-0.7,0.13	-0.24<br>-0.64,0.15	-0.21<br>-0.52,0.1	-0.2<br>-0.52,0.13	-0.14<br>-0.71,0.41	-0.14<br>-0.97,0.7	-0.11<br>-0.41,0.18	-0.11<br>-0.41,0.2	-0.1<br>-0.61,0.42	-0.08<br>-0.78,0.62	-0.09<br>-0.41,0.23	-0.06<br>-0.83,0.74	-0.08<br>-0.39,0.23	-0.05<br>-0.59,0.49	-0.05<br>-0.46,0.37	-0.04<br>-0.5,0.42	-0.06<br>-0.34,0.24	-0.05<br>-0.34,0.24	-0.04<br>-0.35,0.27	0.06<br>-0.61,0.75	LOX	NA	NA	NA	-0.16<br>-0.69, 0.37
**-0.84<br>-1.57,-0.11**	-0.46<br>-1.11,0.19	-0.38<br>-0.99,0.23	-0.32<br>-0.91,0.28	-0.34<br>-1.01,0.33	-0.3<br>-0.95,0.35	-0.27<br>-0.87,0.33	-0.26<br>-0.87,0.35	-0.2<br>-0.98,0.56	-0.2<br>-1.21,0.82	-0.18<br>-0.77,0.42	-0.17<br>-0.77,0.44	-0.16<br>-0.89,0.58	-0.14<br>-1.01,0.73	-0.15<br>-0.76,0.45	-0.12<br>-1.08,0.87	-0.14<br>-0.74,0.46	-0.11<br>-0.89,0.63	-0.11<br>-0.83,0.63	-0.11<br>-0.79,0.6	-0.12<br>-0.72,0.49	-0.11<br>-0.71,0.48	-0.1<br>-0.71,0.51	-0.01<br>-0.85,0.85	-0.06<br>-0.69,0.58	CPX	NA	NA	0.05<br>-0.71, 0.82
**-0.79<br>-1.29,-0.3**	**-0.41<br>-0.75,-0.07**	**-0.33<br>-0.56,-0.11**	**-0.27<br>-0.45,-0.07**	-0.29<br>-0.64,0.06	-0.25<br>-0.58,0.07	**-0.22<br>-0.43,-0.01**	-0.21<br>-0.44,0.03	-0.15<br>-0.68,0.36	-0.15<br>-0.96,0.67	-0.13<br>-0.33,0.08	-0.12<br>-0.32,0.09	-0.11<br>-0.59,0.36	-0.09<br>-0.78,0.6	-0.1<br>-0.33,0.12	-0.07<br>-0.85,0.72	-0.09<br>-0.31,0.12	-0.06<br>-0.58,0.45	-0.06<br>-0.53,0.41	-0.05<br>-0.46,0.36	-0.07<br>-0.33,0.2	-0.06<br>-0.24,0.12	-0.05<br>-0.28,0.18	0.05<br>-0.61,0.7	-0.01<br>-0.35,0.32	0.05<br>-0.57,0.67	SER	NA	-0.10<br>-0.35, 0.15
**-0.95<br>-1.57,-0.32**	**-0.56<br>-1.08,-0.04**	**-0.48<br>-0.88,-0.09**	-0.42<br>-0.84,0.01	-0.45<br>-0.96,0.07	-0.41<br>-0.91,0.1	-0.37<br>-0.81,0.07	-0.36<br>-0.81,0.09	-0.3<br>-0.95,0.35	-0.3<br>-1.2,0.6	-0.28<br>-0.71,0.17	-0.27<br>-0.7,0.16	-0.26<br>-0.87,0.33	-0.24<br>-1.03,0.55	-0.25<br>-0.69,0.19	-0.22<br>-1.09,0.66	-0.24<br>-0.69,0.19	-0.21<br>-0.86,0.43	-0.21<br>-0.82,0.41	-0.21<br>-0.76,0.35	-0.22<br>-0.68,0.26	-0.21<br>-0.64,0.21	-0.2<br>-0.65,0.25	-0.11<br>-0.86,0.66	-0.16<br>-0.67,0.35	-0.1<br>-0.84,0.63	-0.15<br>-0.61,0.31	FPX	NA
**-0.9<br>-1.36,-0.44**	**-0.52<br>-0.82,-0.23**	**-0.44<br>-0.6,-0.28**	**-0.37<br>-0.46,-0.29**	**-0.4<br>-0.69,-0.1**	**-0.36<br>-0.63,-0.09**	**-0.33<br>-0.44,-0.21**	**-0.32<br>-0.47,-0.17**	-0.26<br>-0.76,0.23	-0.26<br>-1.05,0.54	**-0.24<br>-0.34,-0.13**	**-0.23<br>-0.34,-0.11**	-0.22<br>-0.66,0.21	-0.2<br>-0.87,0.46	**-0.21<br>-0.36,-0.06**	-0.18<br>-0.94,0.6	**-0.2<br>-0.32,-0.09**	-0.17<br>-0.66,0.31	-0.17<br>-0.61,0.27	-0.16<br>-0.53,0.2	-0.18<br>-0.37,0.03	**-0.17<br>-0.26,-0.08**	**-0.16<br>-0.29,-0.03**	-0.06<br>-0.69,0.58	-0.12<br>-0.41,0.17	-0.06<br>-0.66,0.54	-0.11<br>-0.29,0.07	0.04<br>-0.39,0.47	PBO

Table 11.3: Depressive symptoms

Treatments are ranked according to their surface under the curve cumulative ranking (SUCRA) for negative symptoms starting with the best. Results of the **network meta-analysis** are presented in the **left lower half** and results from **pairwise comparisons** in the **upper right** half, if available. Comparisons between treatments should be read from left to right and the estimate is in the cell in common between the column-defining treatment and the row-defining treatment. In the left lower half, standard mean differences (SMDs) lower than 0 favor the column-defining treatment, in the upper right half SMDs lower than 0 favor the row defining treatment. Cells in bold print indicate significant results. NA=not available. Drug abbreviations: AMI=Amisulpride, ARI=Aripiprazole, ASE=Asenapine, BRE=Brexpiprazole, CAR=Cariprazine, CPZ=Chlorpromazine, CPX=Clopenthixol, CLO=Clozapine, FPX=Flupenthixol, HAL=Haloperidol, LOX=Loxapine, LUR=Lurasidone, MOL=Molindone, OLA=Olanzapine, PAL=Paliperidone, PEN=Penfluridol, PERA=Perazine, PERPH=Perphenazine, PIM=Pimozide, PBO=Placebo, QUE=Quetiapine, RIS=Risperidone, SER=Sertindole, SUL=Sulpiride, THIOTH=Thiothixene, TRIFLU=Trifluoperazine, ZIP=Ziprasidone, ZOT=Zotepine, ZUC=Zuclopenthixol.

Table 11.4: Social functioning

**THIOR**	NA	NA	NA	NA	NA	NA	NA	NA	NA	**-0.71<br>-1.28,-0.13**	NA	NA
-0.17<br>-0.75,0.42	**OLA**	-0.02<br>-0.18, 0.13	NA	NA	NA	NA	NA	NA	NA	**-0.51<br>-0.71,-0.32**	NA	NA
-0.18<br>-0.75,0.39	-0.01<br>-0.19,0.17	**PAL**	NA	NA	NA	NA	NA	NA	NA	**-0.52<br>-0.64,-0.39**	NA	NA
-0.22<br>-0.82,0.39	-0.05<br>-0.37,0.26	-0.04<br>-0.33,0.25	**QUE**	NA	-0.20<br>-0.43, 0.04	NA	NA	NA	NA	**-0.50<br>-0.74,-0.26**	NA	**-0.58<br>-0.88,-0.27**
-0.25<br>-0.86,0.37	-0.08<br>-0.42,0.27	-0.07<br>-0.39,0.25	-0.03<br>-0.4,0.34	**LUR**	NA	NA	NA	NA	NA	**-0.45<br>-0.68,-0.21**	NA	NA
-0.44<br>-1,0.13	**-0.28<br>-0.52,-0.04**	**-0.26<br>-0.46,-0.07**	-0.22<br>-0.47,0.02	-0.2<br>-0.51,0.13	**BRE**	-0.00<br>-0.35, 0.35	NA	NA	NA	**-0.25<br>-0.35,-0.14**	NA	NA
-0.46<br>-1.09,0.17	-0.3<br>-0.68,0.1	-0.28<br>-0.64,0.07	-0.24<br>-0.65,0.15	-0.21<br>-0.64,0.22	-0.02<br>-0.34,0.31	**ARI**	NA	NA	NA	-0.21<br>-0.56, 0.14	NA	NA
-0.6<br>-1.41,0.2	-0.43<br>-1.05,0.18	-0.42<br>-1.01,0.18	-0.38<br>-0.91,0.14	-0.35<br>-1,0.28	-0.16<br>-0.74,0.43	-0.14<br>-0.77,0.53	**SER**	NA	NA	NA	NA	-0.19<br>-0.56, 0.18
-0.68<br>-1.43,0.07	-0.51<br>-1.05,0.03	-0.5<br>-1.03,0.03	**-0.46<br>-0.9,-0.02**	-0.43<br>-1.01,0.12	-0.24<br>-0.74,0.27	-0.22<br>-0.82,0.37	-0.08<br>-0.56,0.41	**AMI**	NA	NA	-0.12<br>-0.47, 0.22	-0.12<br>-0.35, 0.12
-0.73<br>-1.5,0.03	**-0.57<br>-1.12,-0.03**	**-0.55<br>-1.09,-0.03**	**-0.51<br>-0.97,-0.06**	-0.48<br>-1.06,0.07	-0.29<br>-0.8,0.21	-0.27<br>-0.87,0.33	-0.13<br>-0.62,0.35	-0.05<br>-0.45,0.34	**ZIP**	NA	NA	-0.06<br>-0.31, 0.18
**-0.69<br>-1.24,-0.14**	**-0.53<br>-0.73,-0.33**	**-0.51<br>-0.66,-0.37**	**-0.47<br>-0.72,-0.22**	**-0.44<br>-0.72,-0.16**	**-0.25<br>-0.38,-0.12**	-0.23<br>-0.55,0.09	-0.09<br>-0.68,0.49	-0.01<br>-0.52,0.49	0.04<br>-0.47,0.55	**PBO**	NA	NA
-0.8<br>-1.66,0.03	-0.63<br>-1.31,0.02	-0.62<br>-1.29,0.01	**-0.58<br>-1.18,-0.02**	-0.55<br>-1.25,0.1	-0.36<br>-1,0.26	-0.34<br>-1.06,0.35	-0.2<br>-0.81,0.4	-0.12<br>-0.5,0.24	-0.07<br>-0.62,0.48	-0.11<br>-0.76,0.51	**FPX**	NA
**-0.79<br>-1.49,-0.09**	**-0.63<br>-1.09,-0.16**	**-0.61<br>-1.06,-0.16**	**-0.57<br>-0.92,-0.22**	**-0.54<br>-1.04,-0.06**	-0.35<br>-0.77,0.08	-0.33<br>-0.86,0.2	-0.19<br>-0.58,0.2	-0.11<br>-0.37,0.16	-0.06<br>-0.34,0.23	-0.1<br>-0.52,0.33	0.01<br>-0.45,0.48	**RIS**

Table 11.4: Social functioning
Treatments are ranked according to their surface under the curve cumulative ranking (SUCRA) for social functioning starting with the best. Results of the **network meta-analysis** are presented in the **left lower half** and results from **pairwise comparisons** in the **upper right** half, if available. Comparisons between treatments should be read from left to right and the estimate is in the cell in common between the column-defining treatment and the row-defining treatment. In the left lower half, standard mean differences (SMDs) lower than 0 favor the column-defining treatment, in the upper right half SMDs lower than 0 favor the row defining treatment. Cells in bold print indicate significant results. NA=not available. Drug abbreviations: AMI=Amisulpride, ARI=Aripiprazole, BRE=Brexpiprazole, FPX=Flupenthixol, LUR=Lurasidone, OLA=Olanzapine, PAL=Paliperidone, PBO=Placebo, QUE=Quetiapine, RIS=Risperidone, SER=Sertindole, THIOR=Thioridazine, ZIP=Ziprasidone.

Table 11.5: All-cause discontinuation

THIOTH	NA	0.49<br>0.20, 1.17	NA	NA	NA	NA	NA	NA	NA	0.73<br>0.30, 1.77	NA	NA	NA	0.93<br>0.20, 4.22	NA	NA	NA	NA	NA	NA	NA	NA	NA	NA	NA	1.17<br>0.60, 2.26	NA	NA	NA	0.20<br>0.01, 3.85	NA	0.77<br>0.46, 1.30
0.95<br>0.7,3.23	CPX	NA	NA	NA	NA	NA	NA	NA	NA	NA	NA	NA	1.00<br>0.15, 6.58	NA	NA	NA	NA	NA	NA	NA	NA	NA	NA	NA	0.94<br>0.02, 44.22	0.21<br>0.04, 1.14	NA	NA	NA	NA	NA	0.31<br>0.01, 7.08
0.86<br>0.74,1.16	0.9<br>0.24,1.32	THIOR	NA	NA	NA	NA	NA	NA	NA	1.09<br>0.02, 50.53	NA	NA	NA	0.69<br>0.40, 1.22	NA	NA	NA	NA	0.91<br>0.21, 4.02	NA	NA	NA	NA	NA	0.33<br>0.04, 2.57	1.01<br>0.44, 2.34	NA	0.50<br>0.10, 2.44	NA	NA	NA	**0.40**<br>**0.22, 0.72**
0.84<br>0.72,1.12	0.75<br>0.64,1.52	0.95<br>0.81,1.25	AMI	0.90<br>0.65, 1.25	NA	NA	1.18<br>0.78, 1.78	NA	1.50<br>0.29, 7.77	NA	NA	NA	NA	NA	NA	NA	NA	0.67<br>0.41, 1.11	NA	NA	NA	NA	NA	NA	**0.60**<br>**0.44, 0.83**	NA	NA	NA	NA	NA	NA	NA
0.83<br>0.72,1.06	0.82<br>0.21,1.27	0.93<br>0.8,1.18	0.98<br>0.85,1.09	OLA	1.00<br>0.85, 1.17	0.33<br>0.04, 2.86	0.82<br>0.68, 1.00	NA	NA	NA	NA	NA	0.83<br>0.64, 1.08	NA	0.84<br>0.68, 1.03	NA	**0.79**<br>**0.64, 0.98**	1.73<br>0.53, 5.62	NA	0.91<br>0.75, 1.11	NA	0.79<br>0.57, 1.09	**0.67**<br>**0.55, 0.81**	NA	**0.72**<br>**0.64, 0.81**	NA	NA	NA	NA	NA	**0.53**<br>**0.36, 0.78**	**0.72**<br>**0.67, 0.79**
0.83<br>0.72,1.06	0.82<br>0.21,1.27	0.93<br>0.8,1.19	0.98<br>0.83,1.11	1<br>0.92,1.07	PAL	NA	NA	NA	NA	NA	NA	NA	NA	NA	NA	NA	NA	NA	NA	NA	NA	NA	NA	NA	NA	NA	NA	NA	NA	NA	NA	**0.67**<br>**0.60, 0.75**
0.79<br>0.7,1.04	0.77<br>0.19,1.24	0.88<br>0.76,1.15	0.93<br>0.72,1.1	0.94<br>0.83,1.12	0.94<br>0.83,1.14	CLO	0.55<br>0.27, 1.10	NA	NA	0.70<br>0.31, 1.56	NA	1.18<br>0.76, 1.83	NA	NA	NA	NA	NA	NA	NA	NA	NA	NA	NA	NA	0.76<br>0.41, 1.41	0.79<br>0.54, 1.14	NA	NA	NA	NA	NA	NA
**0.79**<br>**0.7,0.99**	0.75<br>0.18,1.21	0.88<br>0.77,1.08	0.91<br>0.78,1.02	**0.93**<br>**0.87,0.98**	0.93<br>0.84,1.02	0.99<br>0.82,1.14	RIS	0.77<br>0.47, 1.27	NA	NA	0.97<br>0.79, 1.20	0.57<br>0.19, 1.68	1.02<br>0.81, 1.28	NA	1.06<br>0.78, 1.45	NA	0.93<br>0.80, 1.08	NA	NA	1.00<br>0.71, 1.40	NA	NA	**0.75**<br>**0.62, 0.90**	NA	**0.82**<br>**0.70, 0.96**	NA	NA	NA	0.79<br>0.58, 1.09	NA	0.81<br>0.54, 1.23	**0.73**<br>**0.67, 0.80**
0.75<br>0.42,1.05	0.76<br>0.17,1.23	0.89<br>0.58,1.14	0.92<br>0.67,1.13	0.94<br>0.73,1.13	0.95<br>0.72,1.14	1<br>0.74,1.21	1.01<br>0.81,1.19	ZUC	NA	NA	NA	NA	0.77<br>0.57, 1.05	NA	NA	NA	NA	NA	NA	NA	NA	NA	NA	NA	0.97<br>0.73, 1.30	0.69<br>0.43, 1.11	NA	NA	NA	NA	NA	NA
0.79<br>0.68,1.12	0.79<br>0.17,1.28	0.87<br>0.72,1.32	0.94<br>0.6,1.2	0.97<br>0.63,1.21	0.97<br>0.63,1.22	1.03<br>0.66,1.28	0.99<br>0.8,1.45	0.97<br>0.77,1.56	PERA	1.23<br>0.69, 2.22	NA	NA	NA	NA	NA	NA	NA	NA	NA	NA	NA	NA	NA	NA	0.75<br>0.38, 1.48	NA	NA	NA	NA	NA	NA	NA
**0.74**<br>**0.45,0.99**	0.75<br>0.18,1.22	0.88<br>0.61,1.1	0.91<br>0.71,1.08	0.93<br>0.76,1.08	0.94<br>0.75,1.1	0.99<br>0.78,1.17	1<br>0.84,1.15	0.98<br>0.81,1.32	1.01<br>0.69,1.24	ZOT	NA	NA	0.68<br>0.28, 1.61	NA	NA	NA	NA	NA	NA	NA	NA	NA	NA	NA	0.93<br>0.64, 1.35	0.76<br>0.47, 1.22	NA	NA	NA	NA	NA	0.76<br>0.47, 1.22
**0.78**<br>**0.69,0.96**	0.72<br>0.17,1.19	0.86<br>0.76,1.06	0.88<br>0.74,1.01	**0.91**<br>**0.85,0.98**	0.91<br>0.85,1.01	0.97<br>0.78,1.12	0.97<br>0.91,1.05	0.96<br>0.82,1.22	0.94<br>0.78,1.37	0.97<br>0.85,1.18	ILO	NA	NA	NA	NA	NA	NA	NA	NA	NA	NA	NA	1.03<br>0.77, 1.38	NA	**0.82**<br>**0.70, 0.98**	NA	NA	NA	NA	NA	NA	**0.77**<br>**0.68, 0.87**
0.77<br>0.67,1.16	0.77<br>0.16,1.26	0.85<br>0.71,1.33	0.93<br>0.54,1.22	0.9<br>0.74,1.35	0.9<br>0.74,1.37	1.01<br>0.63,1.26	0.96<br>0.77,1.49	0.94<br>0.75,1.61	0.93<br>0.72,1.75	0.95<br>0.76,1.54	1.05<br>0.67,1.31	LEV	NA	NA	NA	NA	NA	NA	NA	NA	NA	NA	NA	NA	1.11<br>0.45, 2.76	NA	NA	NA	NA	NA	NA	NA
**0.78**<br>**0.69,0.98**	0.72<br>0.17,1.18	0.86<br>0.75,1.08	0.88<br>0.71,1.03	0.9<br>0.78,1.02	0.91<br>0.77,1.04	0.97<br>0.77,1.14	0.97<br>0.88,1.1	0.96<br>0.82,1.23	0.99<br>0.65,1.25	0.97<br>0.84,1.19	1.01<br>0.87,1.12	1.02<br>0.64,1.3	PERPH	0.97<br>0.31, 3.00	NA	NA	0.92<br>0.72, 1.17	NA	NA	NA	0.40<br>0.11, 1.42	NA	**0.74**<br>**0.58, 0.95**	NA	NA	1.13<br>0.88, 1.44	NA	0.80<br>0.23, 2.73	NA	NA	NA	1.14<br>0.89, 1.46
0.78<br>0.69,1	0.73<br>0.17,1.2	0.86<br>0.75,1.1	0.89<br>0.66,1.09	0.91<br>0.8,1.1	0.91<br>0.79,1.12	0.98<br>0.73,1.18	0.97<br>0.84,1.2	0.95<br>0.79,1.32	0.93<br>0.76,1.45	0.96<br>0.81,1.28	1.01<br>0.81,1.18	1.03<br>0.61,1.3	0.99<br>0.84,1.27	LOX	NA	NA	NA	NA	NA	NA	NA	NA	NA	NA	0.74<br>0.36, 1.55	1.02<br>0.75, 1.37	NA	NA	NA	0.56<br>0.21, 1.51	NA	**0.47**<br>**0.27, 0.80**
**0.78**<br>**0.69,0.96**	0.71<br>0.63,1.25	0.85<br>0.75,1.05	0.88<br>0.73,1.01	**0.91**<br>**0.86,0.97**	0.91<br>0.85,1	0.94<br>0.83,1.15	0.97<br>0.91,1.05	0.96<br>0.82,1.2	0.93<br>0.78,1.37	0.96<br>0.85,1.16	0.99<br>0.91,1.1	0.94<br>0.76,1.49	0.99<br>0.88,1.15	0.98<br>0.84,1.23	ARI	NA	NA	NA	NA	NA	NA	NA	0.94<br>0.64, 1.37	0.78<br>0.48, 1.27	0.86<br>0.68, 1.10	NA	NA	NA	**0.70**<br>**0.50, 0.98**	NA	NA	**0.80**<br>**0.71, 0.89**
0.75<br>0.66,1.19	0.75<br>0.14,1.26	0.83<br>0.69,1.43	0.92<br>0.46,1.26	0.87<br>0.71,1.45	0.87<br>0.71,1.47	1.01<br>0.53,1.33	0.92<br>0.74,1.61	0.91<br>0.72,1.69	0.89<br>0.7,1.82	0.92<br>0.73,1.67	1.04<br>0.58,1.34	1.06<br>0.47,1.41	0.93<br>0.74,1.72	1.04<br>0.57,1.34	1.05<br>0.59,1.34	MOL	NA	NA	NA	NA	NA	NA	NA	NA	NA	0.20<br>0.01, 3.85	NA	NA	NA	0.84<br>0.51, 1.37	NA	0.31<br>0.01, 7.08
**0.77**<br>**0.69,0.94**	0.7<br>0.16,1.17	0.85<br>0.75,1.03	**0.86**<br>**0.72,0.98**	**0.88**<br>**0.81,0.95**	**0.88**<br>**0.79,0.98**	0.94<br>0.77,1.1	0.95<br>0.9,1.02	0.94<br>0.81,1.18	0.97<br>0.64,1.22	0.95<br>0.84,1.14	0.98<br>0.89,1.06	1<br>0.62,1.27	0.98<br>0.86,1.09	0.99<br>0.78,1.15	0.99<br>0.9,1.07	1.04<br>0.57,1.33	QUE	NA	NA	NA	NA	0.74<br>0.47, 1.15	0.81<br>0.64, 1.02	0.82<br>0.53, 1.26	**0.81**<br>**0.67, 0.97**	0.85<br>0.64, 1.13	NA	NA	NA	NA	NA	**0.80**<br>**0.71, 0.89**
0.76<br>0.66,1.09	0.73<br>0.15,1.22	0.83<br>0.7,1.22	0.88<br>0.57,1.13	0.88<br>0.74,1.22	0.88<br>0.74,1.23	0.97<br>0.59,1.24	0.93<br>0.77,1.33	0.92<br>0.75,1.45	0.9<br>0.72,1.56	0.93<br>0.76,1.43	0.95<br>0.78,1.41	0.91<br>0.72,1.67	0.94<br>0.77,1.43	0.94<br>0.76,1.52	1.01<br>0.67,1.25	0.92<br>0.71,2	0.97<br>0.79,1.43	FPX	NA	NA	NA	NA	NA	NA	NA	NA	NA	NA	NA	NA	NA	1.00<br>0.02, 47.38
0.74<br>0.65,1.15	0.72<br>0.13,1.22	0.81<br>0.68,1.32	0.88<br>0.45,1.23	0.85<br>0.71,1.37	0.85<br>0.71,1.39	0.97<br>0.5,1.3	0.89<br>0.73,1.54	0.88<br>0.71,1.61	0.87<br>0.69,1.72	0.89<br>0.72,1.59	0.91<br>0.74,1.59	0.88<br>0.7,1.82	0.91<br>0.74,1.64	0.91<br>0.73,1.67	1.01<br>0.57,1.32	0.88<br>0.69,2.17	0.93<br>0.75,1.64	1.05<br>0.5,1.38	FLU	NA	NA	NA	NA	NA	1.00<br>0.33, 3.05	1.15<br>0.26, 5.10	0.51<br>0.05, 5.70	NA	NA	NA	NA	0.79<br>0.20, 3.10
**0.76**<br>**0.68,0.93**	0.7<br>0.63,1.19	0.83<br>0.74,1.01	**0.83**<br>**0.68,0.97**	**0.88**<br>**0.83,0.94**	**0.88**<br>**0.81,0.97**	0.91<br>0.81,1.1	0.93<br>0.87,1.02	0.92<br>0.79,1.15	0.9<br>0.76,1.3	0.93<br>0.82,1.11	0.95<br>0.87,1.06	0.91<br>0.75,1.39	0.95<br>0.85,1.1	0.94<br>0.81,1.19	0.96<br>0.85,1.06	0.92<br>0.73,1.61	0.97<br>0.89,1.08	0.95<br>0.78,1.43	0.95<br>0.75,1.61	ASE	NA	NA	NA	NA	0.86<br>0.64, 1.15	NA	NA	NA	NA	NA	NA	**0.86**<br>**0.77, 0.97**
0.71<br>0.64,1.18	0.68<br>0.11,1.21	0.77<br>0.66,1.37	0.84<br>0.34,1.24	0.86<br>0.37,1.25	0.87<br>0.37,1.25	0.93<br>0.4,1.3	0.84<br>0.69,1.56	0.83<br>0.68,1.59	0.82<br>0.68,1.69	0.84<br>0.68,1.59	0.96<br>0.44,1.32	0.98<br>0.35,1.37	0.85<br>0.7,1.59	0.97<br>0.41,1.34	0.97<br>0.44,1.32	1.03<br>0.33,1.41	0.87<br>0.7,1.67	1.02<br>0.41,1.39	1.06<br>0.37,1.42	1.01<br>0.48,1.36	PEN	NA	NA	NA	NA	0.15<br>0.01, 2.57	NA	NA	NA	NA	NA	NA
**0.74**<br>**0.68,0.9**	0.64<br>0.14,1.11	**0.81**<br>**0.72,0.97**	**0.79**<br>**0.64,0.93**	**0.85**<br>**0.8,0.92**	**0.85**<br>**0.79,0.93**	0.88<br>0.69,1.05	**0.9**<br>**0.84,0.98**	0.89<br>0.78,1.1	0.88<br>0.74,1.23	0.9<br>0.8,1.08	0.92<br>0.8,1.02	0.93<br>0.56,1.22	0.92<br>0.83,1.05	0.92<br>0.71,1.1	0.92<br>0.81,1.02	0.89<br>0.72,1.54	0.94<br>0.87,1.03	0.97<br>0.63,1.23	1.01<br>0.54,1.29	0.97<br>0.85,1.07	0.95<br>0.72,1.96	LUR	NA	NA	0.96<br>0.73, 1.26	NA	NA	NA	NA	NA	NA	**0.86**<br>**0.77, 0.97**
**0.62**<br>**0.36,0.87**	0.63<br>0.14,1.1	**0.76**<br>**0.53,0.96**	**0.78**<br>**0.64,0.91**	**0.8**<br>**0.72,0.88**	**0.81**<br>**0.7,0.91**	0.87<br>0.69,1.04	**0.88**<br>**0.8,0.96**	0.88<br>0.78,1.09	0.9<br>0.57,1.16	0.89<br>0.79,1.05	0.91<br>0.81,1	0.93<br>0.55,1.21	0.91<br>0.78,1.03	0.92<br>0.7,1.09	0.91<br>0.82,1	0.97<br>0.51,1.28	0.93<br>0.84,1.01	0.96<br>0.62,1.22	1.01<br>0.54,1.29	0.96<br>0.84,1.06	1.08<br>0.53,1.39	1<br>0.88,1.09	ZIP	NA	1.00<br>0.77, 1.29	NA	NA	NA	NA	NA	NA	**0.77**<br>**0.66, 0.90**
**0.74**<br>**0.67,0.89**	0.69<br>0.62,1.12	**0.8**<br>**0.72,0.96**	**0.78**<br>**0.63,0.92**	**0.84**<br>**0.79,0.92**	**0.85**<br>**0.79,0.93**	0.88<br>0.78,1.04	**0.89**<br>**0.83,0.97**	0.88<br>0.77,1.1	0.86<br>0.74,1.22	0.88<br>0.79,1.06	0.91<br>0.83,1.01	0.87<br>0.73,1.3	0.91<br>0.82,1.04	0.9<br>0.79,1.11	0.91<br>0.79,1.02	0.88<br>0.71,1.49	0.93<br>0.85,1.02	0.91<br>0.75,1.33	0.91<br>0.72,1.52	0.95<br>0.82,1.07	0.94<br>0.72,1.92	0.98<br>0.89,1.12	0.99<br>0.9,1.12	BRE	NA	NA	NA	NA	NA	NA	NA	**0.83**<br>**0.73, 0.96**
**0.74**<br>**0.67,0.88**	0.62<br>0.13,1.09	**0.8**<br>**0.72,0.94**	**0.77**<br>**0.65,0.89**	**0.84**<br>**0.81,0.88**	**0.84**<br>**0.79,0.9**	0.86<br>0.69,1.01	**0.88**<br>**0.85,0.93**	0.88<br>0.78,1.06	0.86<br>0.74,1.18	0.88<br>0.8,1.03	**0.91**<br>**0.85,0.97**	0.87<br>0.73,1.28	0.9<br>0.83,1.01	0.9<br>0.79,1.08	**0.9**<br>**0.82,0.97**	0.88<br>0.71,1.45	**0.93**<br>**0.88,0.98**	0.95<br>0.62,1.2	0.99<br>0.53,1.28	0.94<br>0.85,1.03	0.93<br>0.72,1.85	0.98<br>0.91,1.08	0.99<br>0.92,1.08	1<br>0.89,1.09	HAL	0.29<br>0.03, 2.87	1.04<br>0.22, 4.95	0.81<br>0.30, 2.18	NA	0.90<br>0.53, 1.54	0.93<br>0.80, 1.09	**0.86**<br>**0.80, 0.93**
**0.74**<br>**0.67,0.88**	0.68<br>0.62,1.1	**0.8**<br>**0.72,0.94**	**0.77**<br>**0.6,0.92**	**0.83**<br>**0.78,0.92**	**0.83**<br>**0.78,0.93**	0.87<br>0.78,1.01	**0.88**<br>**0.81,0.97**	0.88<br>0.77,1.06	0.85<br>0.73,1.19	0.88<br>0.79,1.03	0.9<br>0.82,1.01	0.86<br>0.72,1.28	0.9<br>0.81,1.03	0.89<br>0.79,1.08	0.9<br>0.76,1.02	0.87<br>0.71,1.45	0.92<br>0.84,1.02	0.9<br>0.75,1.32	0.9<br>0.72,1.49	0.94<br>0.8,1.07	0.93<br>0.72,1.85	0.97<br>0.87,1.12	0.98<br>0.88,1.12	0.99<br>0.84,1.12	0.99<br>0.9,1.12	CPZ	1.00<br>0.02, 48.13	1.08<br>0.51, 2.28	NA	NA	NA	0.99<br>0.80, 1.22
0.66<br>0.61,1.27	0.66<br>0.04,1.31	0.69<br>0.62,1.54	0.78<br>0.15,1.3	0.8<br>0.16,1.31	0.81<br>0.16,1.31	0.87<br>0.17,1.36	0.73<br>0.63,1.79	0.72<br>0.63,1.89	0.89<br>0.15,1.38	0.73<br>0.63,1.82	0.9<br>0.19,1.38	0.92<br>0.16,1.4	0.91<br>0.2,1.38	0.91<br>0.18,1.39	0.91<br>0.2,1.38	0.97<br>0.16,1.44	0.75<br>0.64,1.92	0.96<br>0.19,1.43	0.98<br>0.19,1.44	0.95<br>0.21,1.4	1.07<br>0.18,1.49	0.99<br>0.24,1.42	0.78<br>0.65,2.22	1.01<br>0.24,1.43	1.01<br>0.25,1.43	1.02<br>0.25,1.45	PIM	NA	NA	NA	NA	NA
**0.71**<br>**0.65,0.91**	0.59<br>0.11,1.12	0.77<br>0.68,1	0.74<br>0.45,1	0.76<br>0.49,1	0.77<br>0.49,1.02	0.83<br>0.52,1.09	0.84<br>0.55,1.08	0.83<br>0.71,1.15	0.85<br>0.42,1.19	0.84<br>0.72,1.14	0.86<br>0.57,1.1	0.88<br>0.43,1.22	0.86<br>0.57,1.11	0.87<br>0.53,1.14	0.87<br>0.58,1.11	0.92<br>0.4,1.28	0.89<br>0.6,1.12	0.92<br>0.48,1.24	0.96<br>0.43,1.3	0.91<br>0.61,1.15	1.04<br>0.43,1.38	0.95<br>0.65,1.19	0.93<br>0.78,1.27	0.97<br>0.66,1.19	0.97<br>0.68,1.19	0.98<br>0.69,1.2	1.26<br>0.38,1.55	SUL	NA	2.00<br>0.20, 20.31	NA	NA
**0.73**<br>**0.67,0.87**	0.68<br>0.62,1.08	**0.79**<br>**0.71,0.93**	**0.75**<br>**0.59,0.89**	**0.83**<br>**0.78,0.88**	**0.83**<br>**0.77,0.91**	0.85<br>0.76,1.01	**0.87**<br>**0.81,0.94**	0.86<br>0.76,1.05	0.85<br>0.72,1.16	0.87<br>0.78,1.02	**0.88**<br>**0.82,0.98**	0.85<br>0.72,1.25	0.88<br>0.81,1.01	0.88<br>0.78,1.08	**0.88**<br>**0.76,0.98**	0.86<br>0.7,1.41	0.9<br>0.83,1	0.88<br>0.74,1.27	0.88<br>0.71,1.45	0.92<br>0.79,1.04	0.92<br>0.71,1.82	0.95<br>0.87,1.08	0.96<br>0.88,1.09	0.97<br>0.84,1.09	0.97<br>0.89,1.08	0.97<br>0.87,1.14	0.96<br>0.69,3.85	1<br>0.82,1.45	CAR	NA	NA	0.87<br>0.76, 1.00
**0.53**<br>**0.22,0.87**	0.53<br>0.1,1.03	**0.66**<br>**0.33,0.99**	**0.69**<br>**0.37,0.99**	0.71<br>0.4,1	0.71<br>0.4,1.01	0.77<br>0.42,1.09	0.79<br>0.46,1.07	0.8<br>0.69,1.15	0.8<br>0.37,1.16	0.8<br>0.69,1.11	0.81<br>0.48,1.1	0.83<br>0.35,1.21	0.81<br>0.47,1.11	0.81<br>0.47,1.1	0.82<br>0.48,1.1	0.82<br>0.52,1.07	0.83<br>0.5,1.11	0.87<br>0.41,1.21	0.91<br>0.36,1.28	0.86<br>0.52,1.14	1<br>0.37,1.37	0.9<br>0.55,1.18	0.87<br>0.74,1.25	0.92<br>0.56,1.19	0.92<br>0.58,1.19	0.93<br>0.58,1.21	1.22<br>0.32,1.53	0.99<br>0.56,1.29	0.95<br>0.59,1.22	TRIFLU	NA	2.60<br>0.31, 22.13
**0.71**<br>**0.66,0.85**	0.56<br>0.12,1.04	**0.77**<br>**0.7,0.91**	**0.71**<br>**0.56,0.85**	**0.73**<br>**0.63,0.83**	**0.74**<br>**0.61,0.85**	**0.8**<br>**0.6,0.97**	**0.81**<br>**0.7,0.9**	0.84<br>0.75,1.02	0.82<br>0.5,1.1	**0.84**<br>**0.76,0.99**	**0.83**<br>**0.71,0.95**	0.85<br>0.48,1.16	**0.84**<br>**0.68,0.98**	0.84<br>0.63,1.03	**0.84**<br>**0.72,0.95**	0.9<br>0.44,1.23	**0.86**<br>**0.74,0.96**	0.89<br>0.55,1.16	0.93<br>0.48,1.24	0.88<br>0.75,1	1.01<br>0.47,1.35	0.92<br>0.79,1.04	0.93<br>0.85,1.04	0.94<br>0.8,1.06	0.94<br>0.84,1.03	0.95<br>0.8,1.08	1.23<br>0.39,1.53	0.97<br>0.8,1.37	0.97<br>0.83,1.09	1.01<br>0.8,1.59	SER	0.98<br>0.83, 1.16
**0.71**<br>**0.66,0.81**	0.52<br>0.11,1	**0.76**<br>**0.69,0.87**	**0.67**<br>**0.55,0.78**	**0.69**<br>**0.65,0.74**	**0.69**<br>**0.62,0.77**	**0.75**<br>**0.59,0.91**	**0.83**<br>**0.8,0.85**	**0.82**<br>**0.74,0.96**	0.78<br>0.48,1.05	**0.82**<br>**0.75,0.93**	**0.79**<br>**0.71,0.87**	0.81<br>0.46,1.11	**0.79**<br>**0.68,0.9**	**0.8**<br>**0.6,0.97**	**0.8**<br>**0.73,0.86**	0.86<br>0.42,1.19	**0.85**<br>**0.82,0.89**	0.84<br>0.53,1.12	0.89<br>0.45,1.2	**0.84**<br>**0.76,0.92**	0.97<br>0.44,1.31	**0.88**<br>**0.8,0.96**	**0.9**<br>**0.85,0.96**	**0.89**<br>**0.8,0.98**	**0.9**<br>**0.85,0.95**	0.91<br>0.79,1.01	1.2<br>0.37,1.52	0.93<br>0.79,1.28	0.93<br>0.83,1.01	0.98<br>0.79,1.47	0.96<br>0.88,1.06	PBO

Table 11.5: All-cause discontinuation

Treatments are ranked according to their surface under the curve cumulative ranking (SUCRA) for all-cause discontinuation starting with the best. Results of the **network meta-analysis** are presented in the **left lower half** and results from **pairwise comparisons** in the **upper right half**, if available. Comparisons between treatments should be read from left to right and the estimate is in the cell in common between the column-defining treatment and the row-defining treatment. Risk ratios (RRs) lower than 1 favor the column-defining treatment. Cells in bold print indicate significant results. NA=not available. Drug abbreviations: AMI=Amisulpride, ARI=Aripiprazole, ASE=Asenapine, BRE=Brexpiprazole, CAR=Cariprazine, CPZ=Chlorpromazine, CPX=Clopenthixol, CLO=Clozapine, FPX=Flupenthixol, FLU=Fluphenazine, HAL=Haloperidol, ILO=Iloperidone, LEV=Levomepromazine, LOX=Loxapine, LUR=Lurasidone, MOL=Molindone, OLA=Olanzapine, PAL=Paliperidone, PEN=Penfluridol, PERA=Perazine, PERPH=Perphenazine, PIM=Pimozide, PBO=Placebo, QUE=Quetiapine, RIS=Risperidone, SER=Sertindole, SUL=Sulpiride, THIOR=Thioridazine, THIOTH=Thiothixene, TRIFLU=Trifluoperazine, ZIP=Ziprasidone, ZOT=Zotepine, ZUC=Zuclopenthixol.

Table 11.6: Weight gain

ZIP	0.24<br>-0.70, 1.18	NA	0.00<br>-1.51, 1.51	0.40<br>-1.13, 1.93	NA	NA	NA	NA	NA	**-1.94**<br>**-3.46,-0.42**	NA	NA	NA	NA	NA	NA	**-1.69**<br>**-2.97,-0.41**	NA	NA	NA	NA	**-1.68**<br>**-3.09,-0.27**	NA	NA	**-4.08**<br>**-5.06,-3.10**	NA
-0.15<br>-0.71,0.4	PBO	-0.36<br>-0.90, 0.19	-0.64<br>-1.31, 0.03	**-0.57**<br>**-1.12,-0.02**	NA	-0.78<br>-1.59, 0.04	NA	NA	NA	NA	NA	-4.00<br>-8.52, 0.52	**-1.16**<br>**-1.99,-0.34**	**-1.16**<br>**-1.92,-0.41**	NA	NA	**-1.70**<br>**-2.21,-1.18**	**-1.17**<br>**-1.72,-0.63**	NA	NA	**-1.89**<br>**-2.53,-1.26**	**-2.35**<br>**-3.08,-1.61**	**-3.69**<br>**-5.31,-2.07**	**-3.17**<br>**-4.50,-1.85**	**-2.52**<br>**-2.96,-2.07**	**-3.80**<br>**-5.64,-1.96**
-0.47<br>-1.23,0.28	-0.32<br>-0.85,0.21	LUR	NA	-0.10<br>-1.66, 1.46	NA	NA	NA	NA	NA	NA	NA	NA	NA	NA	NA	NA	NA	NA	NA	NA	**-1.50**<br>**-2.91,-0.09**	NA	NA	NA	**-3.10**<br>**-4.57,-1.63**	NA
-0.67<br>-1.36,0	**-0.52**<br>**-1.03,-0.02**	-0.2<br>-0.93,0.52	ARI	-0.22<br>-1.40, 0.96	NA	0.00<br>-1.40, 1.41	NA	NA	NA	NA	NA	NA	-0.91<br>-2.30, 0.48	NA	NA	NA	-0.33<br>-1.37, 0.70	NA	NA	NA	-1.70<br>-3.91, 0.51	NA	NA	NA	**-2.48**<br>**-4.13,-0.83**	NA
**-0.69**<br>**-1.31,-0.09**	**-0.54**<br>**-0.93,-0.17**	-0.22<br>-0.85,0.4	-0.02<br>-0.59,0.57	HAL	NA	NA	-0.52<br>-1.56, 0.53	NA	NA	NA	NA	NA	NA	-0.40<br>-1.87, 1.07	NA	NA	-0.63<br>-1.62, 0.36	NA	NA	0.82<br>-1.96, 3.60	**-1.84**<br>**-2.97,-0.71**	**-2.10**<br>**-3.75,-0.45**	NA	**-2.20**<br>**-3.25,-1.15**	**-2.08**<br>**-2.79,-1.37**	**-2.65**<br>**-4.12,-1.18**
-0.77<br>-2.98,1.48	-0.62<br>-2.77,1.59	-0.3<br>-2.5,1.96	-0.09<br>-2.29,2.15	-0.07<br>-2.23,2.14	CPX	NA	NA	NA	NA	NA	NA	NA	NA	NA	NA	NA	NA	NA	NA	NA	NA	NA	-1.72<br>-3.61, 0.17	NA	NA	NA
-0.88<br>-1.8,0.04	-0.73<br>-1.48,0.02	-0.41<br>-1.33,0.52	-0.21<br>-1.05,0.64	-0.19<br>-1.01,0.65	-0.11<br>-2.43,2.2	CAR	NA	NA	NA	NA	NA	NA	NA	NA	NA	NA	-0.73<br>-2.09, 0.62	NA	NA	NA	NA	NA	NA	NA	NA	NA
**-1**<br>**-1.81,-0.19**	**-0.85**<br>**-1.52,-0.18**	-0.53<br>-1.36,0.31	-0.32<br>-1.12,0.47	-0.31<br>-0.97,0.36	-0.23<br>-2.47,2.02	-0.12<br>-1.1,0.87	AMI	NA	-1.20<br>-2.95, 0.55	0.30<br>-2.56, 3.16	NA	NA	NA	NA	NA	NA	-1.16<br>-2.39, 0.06	NA	NA	NA	NA	NA	NA	NA	**-1.48**<br>**-2.46,-0.51**	NA
-0.56<br>-6.86,5.56	-0.41<br>-6.64,5.74	-0.09<br>-6.35,6.06	0.12<br>-6.16,6.26	0.14<br>-6.1,6.25	0.21<br>-6.24,6.55	0.32<br>-5.93,6.49	0.44<br>-5.83,6.62	ZUC	NA	NA	NA	NA	NA	NA	-1.60<br>-10.14, 6.94	NA	NA	NA	NA	NA	NA	NA	-1.90<br>-8.02, 4.22	NA	NA	NA
-1.17<br>-2.71,0.38	-1.02<br>-2.5,0.46	-0.7<br>-2.24,0.86	-0.49<br>-2.03,1.05	-0.47<br>-1.94,1.02	-0.4<br>-3.01,2.16	-0.29<br>-1.96,1.39	-0.17<br>-1.58,1.25	-0.61<br>-6.9,5.78	FPX	NA	NA	NA	NA	NA	NA	NA	NA	NA	NA	NA	NA	NA	NA	NA	**-3.40**<br>**-5.63,-1.17**	NA
-1.19<br>-2.47,0.09	-1.04<br>-2.34,0.28	-0.72<br>-2.12,0.69	-0.51<br>-1.88,0.85	-0.5<br>-1.8,0.82	-0.42<br>-2.93,2.06	-0.31<br>-1.8,1.17	-0.19<br>-1.54,1.2	-0.63<br>-6.86,5.72	-0.02<br>-1.89,1.86	PERA	NA	NA	NA	NA	NA	NA	NA	NA	NA	NA	NA	NA	NA	NA	**-2.49**<br>**-4.14,-0.84**	NA
-0.74<br>-14.74,13.28	-0.59<br>-14.59,13.4	-0.27<br>-14.3,13.67	-0.07<br>-14.1,13.9	-0.05<br>-14.1,13.94	0.03<br>-14.12,14.13	0.14<br>-13.95,14.14	0.26<br>-13.76,14.22	-0.18<br>-12.92,12.36	0.43<br>-13.67,14.5	0.45<br>-13.59,14.48	MOL	NA	NA	NA	NA	NA	NA	NA	-1.77<br>-4.38, 0.84	NA	NA	NA	NA	NA	NA	NA
-1.28<br>-4.9,2.23	-1.13<br>-4.7,2.36	-0.81<br>-4.43,2.73	-0.6<br>-4.19,2.92	-0.58<br>-4.18,2.94	-0.51<br>-4.51,3.48	-0.39<br>-4.07,3.16	-0.28<br>-3.92,3.3	-0.72<br>-7.78,6.35	-0.11<br>-3.94,3.68	-0.09<br>-3.89,3.61	-0.54<br>-14.96,14.01	LOX	NA	NA	NA	NA	NA	NA	NA	NA	NA	NA	-4.00<br>-8.42, 0.42	NA	NA	NA
**-1.36**<br>**-2.3,-0.4**	**-1.21**<br>**-2,-0.4**	-0.89<br>-1.84,0.06	-0.68<br>-1.56,0.2	-0.66<br>-1.53,0.21	-0.59<br>-2.91,1.7	-0.47<br>-1.55,0.6	-0.36<br>-1.38,0.67	-0.8<br>-6.96,5.48	-0.19<br>-1.85,1.48	-0.17<br>-1.68,1.34	-0.62<br>-14.61,13.43	-0.08<br>-3.66,3.59	BRE	NA	NA	NA	NA	NA	NA	NA	NA	NA	NA	NA	NA	NA
**-1.37**<br>**-2.24,-0.49**	**-1.22**<br>**-1.92,-0.51**	**-0.9**<br>**-1.77,-0.01**	-0.69<br>-1.55,0.17	-0.67<br>-1.43,0.11	-0.6<br>-2.9,1.68	-0.49<br>-1.51,0.55	-0.37<br>-1.32,0.59	-0.81<br>-6.98,5.5	-0.2<br>-1.84,1.44	-0.18<br>-1.65,1.27	-0.63<br>-14.62,13.39	-0.09<br>-3.65,3.54	-0.01<br>-1.07,1.06	ASE	NA	NA	-1.13<br>-3.04, 0.78	NA	NA	NA	NA	NA	NA	NA	-1.10<br>-2.76, 0.56	NA
-2.05<br>-12.63,8.66	-1.9<br>-12.47,8.78	-1.58<br>-12.13,9.16	-1.37<br>-11.98,9.31	-1.36<br>-11.97,9.3	-1.28<br>-12.04,9.48	-1.17<br>-11.77,9.53	-1.05<br>-11.61,9.72	-1.49<br>-10.26,7.13	-0.88<br>-11.59,9.92	-0.86<br>-11.52,9.92	-1.31<br>-10.18,7.66	-0.77<br>-12.03,10.43	-0.69<br>-11.31,10.06	-0.68<br>-11.21,10.06	SUL	NA	NA	NA	-0.30<br>-8.82, 8.22	NA	NA	NA	NA	NA	NA	NA
-1.74<br>-4.88,1.42	-1.59<br>-4.7,1.56	-1.27<br>-4.4,1.92	-1.06<br>-4.2,2.1	-1.04<br>-4.16,2.1	-0.97<br>-4.64,2.75	-0.86<br>-4.06,2.37	-0.74<br>-3.94,2.45	-1.18<br>-8.1,5.8	-0.57<br>-4.08,2.89	-0.55<br>-3.92,2.84	-1<br>-15.2,13.35	-0.46<br>-5.12,4.32	-0.38<br>-3.56,2.85	-0.37<br>-3.58,2.83	0.31<br>-10.9,11.17	LEV	NA	NA	NA	-0.31<br>-3.05, 2.43	NA	NA	NA	NA	NA	NA
**-1.59**<br>**-2.2,-1**	**-1.44**<br>**-1.82,-1.07**	**-1.12**<br>**-1.77,-0.49**	**-0.92**<br>**-1.48,-0.35**	**-0.9**<br>**-1.35,-0.44**	-0.82<br>-3.01,1.37	-0.71<br>-1.51,0.09	-0.59<br>-1.28,0.09	-1.04<br>-7.17,5.22	-0.42<br>-1.93,1.05	-0.4<br>-1.74,0.91	-0.85<br>-14.81,13.17	-0.32<br>-3.82,3.29	-0.24<br>-1.1,0.62	-0.23<br>-1.02,0.54	0.46<br>-10.24,11.03	0.14<br>-3,3.26	RIS	NA	NA	-0.92<br>-3.98, 2.14	-0.43<br>-1.73, 0.88	-0.60<br>-1.66, 0.46	NA	-0.60<br>-2.24, 1.04	**-2.21**<br>**-3.10,-1.32**	NA
**-1.63**<br>**-2.36,-0.93**	**-1.48**<br>**-1.98,-1**	**-1.16**<br>**-1.88,-0.45**	**-0.96**<br>**-1.65,-0.26**	**-0.94**<br>**-1.53,-0.35**	-0.87<br>-3.09,1.33	-0.75<br>-1.66,0.15	-0.64<br>-1.43,0.16	-1.08<br>-7.23,5.17	-0.47<br>-2.01,1.05	-0.45<br>-1.81,0.91	-0.89<br>-14.89,13.2	-0.36<br>-3.89,3.25	-0.28<br>-1.2,0.64	-0.27<br>-1.12,0.58	0.41<br>-10.26,11.03	0.1<br>-3.09,3.22	-0.04<br>-0.63,0.56	PAL	NA	NA	NA	NA	NA	NA	**-0.77**<br>**-1.39,-0.14**	NA
-2.5<br>-16.15,11.33	-2.35<br>-16.04,11.43	-2.03<br>-15.74,11.77	-1.83<br>-15.53,11.97	-1.81<br>-15.51,11.97	-1.74<br>-15.52,12.07	-1.62<br>-15.35,12.24	-1.51<br>-15.18,12.32	-1.95<br>-14.35,10.3	-1.34<br>-15.07,12.58	-1.32<br>-15.01,12.56	-1.76<br>-4.4,0.83	-1.23<br>-15.42,13.09	-1.15<br>-14.85,12.64	-1.14<br>-14.91,12.68	-0.46<br>-8.98,8.11	-0.77<br>-14.75,13.14	-0.91<br>-14.62,12.88	-0.87<br>-14.51,12.98	TRIFLU	NA	NA	NA	NA	NA	NA	NA
**-2.05**<br>**-3.63,-0.48**	**-1.9**<br>**-3.38,-0.41**	**-1.58**<br>**-3.15,-0.01**	-1.37<br>-2.92,0.17	-1.35<br>-2.85,0.14	-1.28<br>-3.77,1.21	-1.16<br>-2.82,0.51	-1.05<br>-2.63,0.53	-1.49<br>-7.75,4.89	-0.88<br>-2.95,1.2	-0.86<br>-2.8,1.09	-1.31<br>-15.35,12.8	-0.77<br>-4.54,3.03	-0.69<br>-2.36,0.99	-0.68<br>-2.33,0.95	0<br>-10.8,10.65	-0.31<br>-3.1,2.46	-0.45<br>-1.94,1.05	-0.41<br>-1.97,1.13	0.46<br>-13.34,14.19	CLO	NA	NA	-0.40<br>-2.76, 1.96	NA	3.00<br>-10.69,16.69	-0.65<br>-2.81, 1.51
**-2.08**<br>**-2.81,-1.36**	**-1.93**<br>**-2.44,-1.43**	**-1.61**<br>**-2.31,-0.91**	**-1.4**<br>**-2.08,-0.73**	**-1.39**<br>**-1.95,-0.82**	-1.31<br>-3.53,0.84	**-1.2**<br>**-2.11,-0.31**	**-1.08**<br>**-1.9,-0.28**	-1.52<br>-7.69,4.71	-0.91<br>-2.47,0.62	-0.89<br>-2.29,0.47	-1.34<br>-15.34,12.73	-0.8<br>-4.31,2.8	-0.72<br>-1.67,0.2	-0.71<br>-1.59,0.13	-0.03<br>-10.71,10.53	-0.34<br>-3.52,2.78	-0.49<br>-1.07,0.09	-0.44<br>-1.12,0.23	0.43<br>-13.38,14.11	-0.03<br>-1.57,1.49	QUE	NA	0.50<br>-1.08, 2.08	NA	-1.24<br>-2.72, 0.23	NA
**-2.33**<br>**-3.13,-1.53**	**-2.18**<br>**-2.86,-1.51**	**-1.86**<br>**-2.72,-0.99**	**-1.65**<br>**-2.47,-0.84**	**-1.64**<br>**-2.38,-0.9**	-1.56<br>-3.83,0.7	**-1.45**<br>**-2.45,-0.44**	**-1.33**<br>**-2.25,-0.41**	-1.77<br>-7.95,4.44	-1.16<br>-2.79,0.43	-1.14<br>-2.58,0.29	-1.59<br>-15.61,12.45	-1.05<br>-4.6,2.63	-0.97<br>-2.02,0.04	-0.96<br>-1.95,0.01	-0.28<br>-10.92,10.3	-0.59<br>-3.8,2.59	**-0.74**<br>**-1.46,-0.03**	-0.69<br>-1.53,0.13	0.18<br>-13.64,13.88	-0.28<br>-1.91,1.34	-0.25<br>-1.06,0.57	ILO	NA	NA	NA	NA
**-2.5**<br>**-3.57,-1.45**	**-2.35**<br>**-3.28,-1.44**	**-2.03**<br>**-3.08,-0.98**	**-1.83**<br>**-2.86,-0.8**	**-1.81**<br>**-2.77,-0.87**	-1.73<br>-3.68,0.22	**-1.62**<br>**-2.82,-0.45**	**-1.5**<br>**-2.62,-0.4**	-1.94<br>-8.07,4.23	-1.33<br>-3.06,0.37	-1.31<br>-2.87,0.27	-1.76<br>-15.73,12.19	-1.22<br>-4.69,2.37	-1.14<br>-2.37,0.05	**-1.13**<br>**-2.31,-0.02**	-0.45<br>-11.09,10.09	-0.76<br>-3.92,2.38	-0.91<br>-1.89,0.04	-0.86<br>-1.89,0.15	0<br>-13.7,13.61	-0.45<br>-1.98,1.05	-0.42<br>-1.39,0.53	-0.17<br>-1.31,0.95	CPZ	NA	-0.60<br>-2.24, 1.04	-1.00<br>-2.84, 0.84
**-2.62**<br>**-3.53,-1.73**	**-2.47**<br>**-3.23,-1.7**	**-2.15**<br>**-3.06,-1.22**	**-1.94**<br>**-2.82,-1.05**	**-1.92**<br>**-2.69,-1.15**	-1.85<br>-4.17,0.4	**-1.73**<br>**-2.78,-0.68**	**-1.62**<br>**-2.58,-0.66**	-2.06<br>-8.27,4.24	-1.45<br>-3.09,0.18	-1.43<br>-2.92,0.05	-1.88<br>-15.89,12.22	-1.34<br>-4.91,2.34	**-1.26**<br>**-2.35,-0.16**	**-1.25**<br>**-2.27,-0.24**	-0.57<br>-11.31,10.03	-0.88<br>-4.08,2.31	**-1.02**<br>**-1.8,-0.24**	**-0.98**<br>**-1.86,-0.09**	-0.11<br>-13.9,13.62	-0.57<br>-2.23,1.07	-0.54<br>-1.41,0.35	-0.29<br>-1.28,0.71	-0.12<br>-1.29,1.08	SER	-0.80<br>-2.32, 0.72	NA
**-2.93**<br>**-3.51,-2.36**	**-2.78**<br>**-3.12,-2.46**	**-2.46**<br>**-3.06,-1.86**	**-2.25**<br>**-2.82,-1.7**	**-2.24**<br>**-2.64,-1.83**	**-2.16**<br>**-4.34,-0.01**	**-2.05**<br>**-2.87,-1.24**	**-1.93**<br>**-2.58,-1.29**	-2.37<br>-8.5,3.87	**-1.76**<br>**-3.22,-0.3**	**-1.74**<br>**-3.04,-0.44**	-2.19<br>-16.13,11.85	-1.65<br>-5.16,1.94	**-1.57**<br>**-2.43,-0.73**	**-1.56**<br>**-2.33,-0.82**	-0.88<br>-11.59,9.72	-1.19<br>-4.35,1.95	**-1.34**<br>**-1.77,-0.91**	**-1.29**<br>**-1.81,-0.78**	-0.42<br>-14.23,13.22	-0.88<br>-2.38,0.62	**-0.85**<br>**-1.4,-0.29**	-0.6<br>-1.33,0.12	-0.43<br>-1.36,0.51	-0.31<br>-1.09,0.44	OLA	NA
**-3.35**<br>**-4.54,-2.18**	**-3.2**<br>**-4.27,-2.12**	**-2.88**<br>**-4.07,-1.68**	**-2.68**<br>**-3.84,-1.51**	**-2.66**<br>**-3.7,-1.59**	**-2.58**<br>**-4.9,-0.28**	**-2.47**<br>**-3.76,-1.16**	**-2.35**<br>**-3.58,-1.12**	-2.79<br>-8.95,3.51	**-2.18**<br>**-3.98,-0.39**	**-2.16**<br>**-3.83,-0.51**	-2.61<br>-16.65,11.45	-2.08<br>-5.68,1.62	**-2**<br>**-3.31,-0.66**	**-1.98**<br>**-3.27,-0.71**	-1.3<br>-12.02,9.3	-1.61<br>-4.82,1.51	**-1.76**<br>**-2.85,-0.64**	**-1.72**<br>**-2.86,-0.54**	-0.85<br>-14.65,12.82	-1.31<br>-2.81,0.2	**-1.27**<br>**-2.41,-0.11**	-1.02<br>-2.27,0.24	-0.85<br>-2.06,0.39	-0.73<br>-2,0.53	-0.42<br>-1.51,0.67	ZOT

Table 11.6: Weight gain

Treatments are ranked according to their surface under the curve cumulative ranking (SUCRA) for weight gain in kilogramm starting with the best.Results of the **network meta-analysis** are presented in the **left lower half** and results from **pairwise comparisons** in the **upper right** half, if available. Comparisons between treatments should be read from left to right and the estimate is in the cell in common between the column-defining treatment and the row-defining treatment. In the left lower half, mean differences (MDs) lower than 0 favor the column-defining treatment, in the upper right half MDs lower than 0 favor the row defining treatment. Cells in bold print indicate significant results. NA=not available. Drug abbreviations: AMI=Amisulpride, ARI=Aripiprazole, ASE=Asenapine, BRE=Brexpiprazole, CAR=Cariprazine, CPZ=Chlorpromazine, CPX=Clopenthixol, CLO=Clozapine, FPX=Flupenthixol, HAL=Haloperidol, ILO=Iloperidone, LEV=Levomepromazine, LOX=Loxapine, LUR=Lurasidone, MOL=Molindone, OLA=Olanzapine, PAL=Paliperidone, PERA=Perazine, PIM=Pimozide, PBO=Placebo, QUE=Quetiapine, RIS=Risperidone, SER=Sertindole, SUL=Sulpiride, TRIFLU=Trifluoperazine, ZIP=Ziprasidone, ZOT=Zotepine, ZUC=Zuclopenthixol.

Table 11.7: Use of antiparkinson medication

CLO	NA	NA	NA	0.20<br>0.01, 3.97	NA	NA	NA	NA	NA	NA	NA	NA	NA	NA	0.40<br>0.02, 8.13	NA	NA	NA	0.18<br>0.04, 0.79	NA	NA	NA	NA	NA	NA	NA	NA	NA	0.35<br>0.18, 0.68	NA	NA	NA
0.83<br>0.2,2.02	PERA	NA	NA	NA	NA	NA	NA	NA	NA	0.60<br>0.16, 2.25	NA	NA	NA	NA	NA	NA	0.66<br>0.19, 2.24	NA	NA	NA	NA	NA	NA	NA	NA	NA	NA	NA	0.15<br>0.04, 0.60	NA	NA	NA
0.5<br>0.18,1.02	0.8<br>0.23,1.74	SER	0.71<br>0.43, 1.18	1.60<br>0.81, 3.16	NA	NA	NA	NA	NA	NA	NA	NA	NA	NA	NA	NA	NA	NA	NA	NA	NA	NA	NA	NA	NA	NA	NA	NA	0.29<br>0.20, 0.42	NA	NA	NA
0.46<br>0.19,0.88	0.73<br>0.22,1.56	0.92<br>0.64,1.41	PBO	0.85<br>0.59, 1.22	1.14<br>0.73, 1.76	0.88<br>0.50, 1.52	NA	0.87<br>0.59, 1.27	NA	NA	0.61<br>0.28, 1.34	0.68<br>0.37, 1.25	0.66<br>0.49, 0.90	0.66<br>0.45, 0.97	0.64<br>0.45, 0.90	0.50<br>0.35, 0.70	0.75<br>0.16, 3.42	0.51<br>0.28, 0.92	0.56<br>0.34, 0.94	NA	NA	NA	NA	0.38<br>0.04, 3.42	NA	NA	0.22<br>0.07, 0.67	NA	0.39<br>0.32, 0.47	0.15<br>0.04, 0.61	0.40<br>0.12, 1.33	NA
0.46<br>0.18,0.89	0.58<br>0.31,1.64	0.9<br>0.62,1.43	0.98<br>0.78,1.27	OLA	NA	NA	NA	0.50<br>0.05, 5.46	NA	NA	NA	NA	0.63<br>0.38, 1.04	0.54<br>0.32, 0.93	0.86<br>0.50, 1.50	0.59<br>0.32, 1.11	NA	NA	NA	NA	NA	NA	NA	NA	0.12<br>0.02, 0.93	NA	NA	NA	0.39<br>0.29, 0.51	NA	NA	NA
0.44<br>0.17,0.87	0.69<br>0.2,1.52	0.85<br>0.56,1.45	0.95<br>0.68,1.28	0.97<br>0.65,1.37	QUE	NA	NA	NA	NA	NA	NA	NA	NA	NA	0.82<br>0.39, 1.72	0.55<br>0.25, 1.19	NA	NA	0.66<br>0.27, 1.59	NA	NA	NA	NA	NA	NA	NA	NA	NA	0.34<br>0.24, 0.49	NA	NA	NA
0.39<br>0.24,0.93	0.5<br>0.27,1.64	0.77<br>0.45,1.64	0.83<br>0.52,1.54	0.84<br>0.51,1.61	0.88<br>0.52,1.75	ASE	NA	NA	NA	NA	NA	NA	NA	NA	0.56<br>0.24, 1.26	NA	NA	NA	NA	NA	NA	NA	NA	NA	NA	NA	NA	NA	0.42<br>0.23, 0.74	NA	NA	NA
0.58<br>0.07,1.91	0.43<br>0.22,3.57	0.64<br>0.28,4.35	0.68<br>0.31,4.55	0.69<br>0.31,4.55	0.72<br>0.31,5	1.5<br>0.22,3.64	LEV	NA	NA	NA	NA	NA	NA	NA	0.60<br>0.15, 2.37	NA	NA	NA	NA	NA	NA	NA	NA	NA	NA	NA	NA	NA	0.41<br>0.11, 1.48	NA	NA	NA
0.36<br>0.24,0.74	0.46<br>0.26,1.32	0.7<br>0.46,1.23	0.75<br>0.55,1.11	0.76<br>0.53,1.19	0.79<br>0.54,1.32	0.83<br>0.49,1.75	0.61<br>0.27,3.7	ARI	NA	NA	NA	NA	NA	NA	0.59<br>0.27, 1.30	NA	NA	NA	NA	NA	NA	NA	NA	NA	NA	NA	NA	NA	0.49<br>0.33, 0.74	NA	NA	NA
0.39<br>0.1,0.99	0.63<br>0.13,1.64	0.83<br>0.28,1.74	0.87<br>0.33,1.72	0.89<br>0.32,1.77	0.93<br>0.34,1.88	1.05<br>0.32,2.21	1.27<br>0.14,3.24	1.16<br>0.41,2.31	THIOR	NA	NA	NA	NA	NA	NA	NA	0.36<br>0.02, 8.30	NA	0.44<br>0.17, 1.13	NA	NA	NA	NA	NA	NA	NA	NA	NA	0.91<br>0.56, 1.46	0.46<br>0.18, 1.16	NA	NA
0.34<br>0.23,0.67	0.43<br>0.26,1.14	0.64<br>0.42,1.11	0.68<br>0.49,1.04	0.69<br>0.48,1.1	0.72<br>0.49,1.19	0.75<br>0.45,1.59	0.56<br>0.25,3.33	0.87<br>0.55,1.56	0.78<br>0.4,2.27	AMI	NA	NA	NA	NA	1.06<br>0.64, 1.78	NA	NA	NA	NA	NA	NA	NA	NA	0.70<br>0.40, 1.23	NA	NA	NA	NA	0.53<br>0.37, 0.74	NA	NA	NA
0.34<br>0.09,0.83	0.41<br>0.23,1.41	0.6<br>0.34,1.52	0.65<br>0.38,1.47	0.65<br>0.38,1.52	0.68<br>0.38,1.67	0.9<br>0.29,1.9	0.53<br>0.24,3.85	1<br>0.38,1.96	0.74<br>0.35,2.7	1.11<br>0.41,2.12	ILO	NA	NA	0.78<br>0.40, 1.52	NA	NA	NA	NA	NA	NA	NA	NA	NA	NA	NA	NA	NA	NA	NA	NA	NA	NA
0.32<br>0.21,0.76	0.4<br>0.23,1.33	0.58<br>0.34,1.33	0.63<br>0.39,1.25	0.63<br>0.38,1.35	0.66<br>0.38,1.45	0.84<br>0.3,1.72	0.51<br>0.24,3.57	0.94<br>0.38,1.75	0.71<br>0.35,2.44	1.04<br>0.41,1.97	0.82<br>0.4,2.7	BRE	NA	NA	NA	NA	NA	NA	NA	NA	NA	NA	NA	NA	NA	NA	NA	NA	NA	NA	NA	NA
0.27<br>0.1,0.57	0.39<br>0.24,1.03	0.57<br>0.39,0.95	0.62<br>0.47,0.85	0.63<br>0.41,0.9	0.65<br>0.45,1.03	0.75<br>0.35,1.33	0.92<br>0.13,2.42	0.83<br>0.47,1.3	0.7<br>0.37,1.92	0.93<br>0.51,1.47	0.98<br>0.37,1.88	1.02<br>0.43,1.87	PAL	NA	NA	NA	NA	NA	NA	NA	NA	NA	NA	NA	NA	NA	NA	NA	NA	NA	NA	NA
0.26<br>0.1,0.54	0.42<br>0.12,0.98	0.56<br>0.32,0.9	0.59<br>0.41,0.81	0.6<br>0.4,0.85	0.64<br>0.39,0.96	0.72<br>0.34,1.27	0.88<br>0.12,2.31	0.8<br>0.46,1.24	0.84<br>0.29,1.71	0.89<br>0.51,1.38	0.92<br>0.39,1.69	0.98<br>0.41,1.8	0.99<br>0.6,1.48	ZIP	0.90<br>0.61, 1.33	NA	NA	NA	NA	NA	NA	NA	NA	NA	NA	NA	NA	NA	0.54<br>0.33, 0.89	NA	NA	NA
0.25<br>0.1,0.49	0.4<br>0.12,0.91	0.54<br>0.39,0.82	0.56<br>0.42,0.71	0.57<br>0.41,0.77	0.6<br>0.4,0.85	0.68<br>0.35,1.15	0.83<br>0.12,2.19	0.75<br>0.47,1.11	0.66<br>0.36,1.67	0.84<br>0.53,1.21	0.89<br>0.36,1.7	0.93<br>0.41,1.68	0.94<br>0.61,1.36	0.94<br>0.69,1.35	RIS	NA	NA	NA	NA	NA	0.83<br>0.40, 1.74	NA	0.52<br>0.26, 1.05	NA	NA	NA	NA	NA	0.62<br>0.50, 0.77	NA	NA	NA
0.22<br>0.08,0.45	0.34<br>0.22,0.84	0.48<br>0.34,0.78	0.52<br>0.4,0.7	0.52<br>0.39,0.75	0.54<br>0.39,0.81	0.6<br>0.28,1.09	0.74<br>0.1,2.03	0.67<br>0.38,1.06	0.58<br>0.33,1.52	0.75<br>0.41,1.2	0.79<br>0.3,1.58	0.82<br>0.34,1.56	0.79<br>0.53,1.3	0.82<br>0.56,1.33	0.86<br>0.61,1.32	LUR	NA	NA	NA	NA	NA	NA	NA	NA	NA	NA	NA	NA	0.46<br>0.25, 0.85	NA	NA	NA
0.21<br>0.07,0.48	0.34<br>0.1,0.78	0.47<br>0.21,0.88	0.49<br>0.25,0.85	0.5<br>0.25,0.89	0.53<br>0.26,0.95	0.6<br>0.23,1.21	0.74<br>0.09,2.11	0.67<br>0.31,1.21	0.7<br>0.22,1.51	0.74<br>0.34,1.32	0.79<br>0.25,1.69	0.83<br>0.28,1.7	0.84<br>0.39,1.48	0.87<br>0.42,1.51	0.9<br>0.47,1.5	1.04<br>0.5,1.79	ZOT	NA	1.28<br>0.65, 2.50	NA	NA	NA	NA	NA	NA	NA	NA	NA	0.66<br>0.41, 1.07	NA	NA	NA
0.25<br>0.19,0.53	0.3<br>0.2,0.88	0.42<br>0.27,0.89	0.45<br>0.29,0.85	0.45<br>0.29,0.89	0.47<br>0.29,0.96	0.55<br>0.19,1.18	0.38<br>0.2,2.27	0.62<br>0.24,1.21	0.5<br>0.27,1.61	0.69<br>0.26,1.36	0.57<br>0.3,1.69	0.76<br>0.23,1.64	0.67<br>0.39,1.52	0.69<br>0.4,1.56	0.73<br>0.42,1.56	0.82<br>0.45,1.89	0.81<br>0.42,2.22	CAR	NA	NA	NA	NA	NA	NA	NA	NA	NA	NA	NA	NA	NA	NA
0.26<br>0.2,0.45	0.31<br>0.21,0.74	0.43<br>0.3,0.72	0.46<br>0.34,0.68	0.46<br>0.34,0.71	0.48<br>0.35,0.75	0.52<br>0.23,0.99	0.39<br>0.2,1.2	0.59<br>0.31,0.98	0.53<br>0.32,1.2	0.65<br>0.34,1.1	0.59<br>0.33,1.47	0.72<br>0.28,1.44	0.69<br>0.46,1.2	0.72<br>0.48,1.22	0.76<br>0.52,1.2	0.85<br>0.54,1.49	0.85<br>0.52,1.67	1.09<br>0.44,2.04	CPZ	1.00<br>0.21, 4.80	NA	NA	NA	NA	NA	0.65<br>0.29, 1.45	0.60<br>0.35, 1.00	NA	0.64<br>0.34, 1.19	NA	0.54<br>0.19, 1.54	0.47<br>0.23, 0.97
0.18<br>0.04,0.5	0.3<br>0.05,0.88	0.4<br>0.12,0.94	0.42<br>0.14,0.93	0.43<br>0.14,0.97	0.45<br>0.14,1.04	0.51<br>0.14,1.24	0.63<br>0.06,2	0.57<br>0.18,1.31	0.45<br>0.25,1.59	0.64<br>0.19,1.42	0.68<br>0.16,1.71	0.71<br>0.17,1.7	0.72<br>0.23,1.57	0.6<br>0.33,1.67	0.78<br>0.26,1.63	0.89<br>0.29,1.89	0.7<br>0.36,2.27	1.07<br>0.28,2.37	1.01<br>0.35,2.04	SUL	NA	NA	NA	1.00<br>0.15, 6.74	NA	NA	NA	NA	0.71<br>0.29, 1.71	NA	NA	NA
0.16<br>0.04,0.41	0.26<br>0.05,0.72	0.36<br>0.24,0.76	0.38<br>0.26,0.75	0.38<br>0.14,0.77	0.4<br>0.26,0.83	0.45<br>0.14,1	0.55<br>0.06,1.72	0.51<br>0.18,1.05	0.43<br>0.25,1.28	0.56<br>0.2,1.15	0.6<br>0.16,1.42	0.63<br>0.18,1.46	0.64<br>0.24,1.3	0.57<br>0.34,1.33	0.61<br>0.36,1.32	0.79<br>0.3,1.58	0.67<br>0.36,1.85	0.95<br>0.28,2.05	0.9<br>0.36,1.71	0.77<br>0.36,2.94	PERPH	NA	NA	NA	NA	NA	1.22<br>0.59, 2.50	NA	NA	NA	0.53<br>0.26, 1.06	NA
0.15<br>0.03,0.45	0.24<br>0.17,0.79	0.31<br>0.21,0.86	0.33<br>0.21,0.86	0.33<br>0.21,0.88	0.34<br>0.22,0.95	0.42<br>0.09,1.13	0.53<br>0.04,1.83	0.47<br>0.12,1.19	0.37<br>0.22,1.41	0.53<br>0.13,1.31	0.56<br>0.11,1.55	0.59<br>0.12,1.59	0.47<br>0.26,1.49	0.48<br>0.27,1.54	0.51<br>0.28,1.59	0.74<br>0.19,1.75	0.56<br>0.29,2.04	0.89<br>0.19,2.22	0.84<br>0.23,1.9	0.65<br>0.3,2.94	0.71<br>0.33,3.12	MOL	NA	1.02<br>0.75, 1.39	NA	NA	NA	NA	NA	NA	NA	NA
0.14<br>0.04,0.34	0.23<br>0.05,0.62	0.31<br>0.12,0.64	0.32<br>0.14,0.62	0.33<br>0.14,0.64	0.35<br>0.15,0.7	0.4<br>0.14,0.87	0.49<br>0.05,1.52	0.44<br>0.18,0.88	0.47<br>0.12,1.15	0.5<br>0.2,0.98	0.53<br>0.15,1.26	0.55<br>0.17,1.26	0.56<br>0.23,1.1	0.58<br>0.25,1.12	0.6<br>0.28,1.1	0.7<br>0.29,1.34	0.74<br>0.27,1.52	0.84<br>0.27,1.83	0.81<br>0.33,1.55	0.99<br>0.26,2.23	1.04<br>0.33,2.19	1.28<br>0.29,2.85	ZUC	NA	NA	NA	NA	NA	0.68<br>0.36, 1.27	NA	NA	NA
0.14<br>0.04,0.35	0.23<br>0.05,0.63	0.31<br>0.11,0.67	0.32<br>0.13,0.66	0.33<br>0.13,0.68	0.35<br>0.13,0.73	0.4<br>0.12,0.91	0.49<br>0.05,1.6	0.44<br>0.16,0.93	0.46<br>0.12,1.14	0.5<br>0.18,1.03	0.53<br>0.14,1.3	0.55<br>0.16,1.31	0.56<br>0.21,1.15	0.52<br>0.31,1.19	0.6<br>0.24,1.18	0.7<br>0.26,1.4	0.6<br>0.33,1.59	0.84<br>0.24,1.87	0.79<br>0.32,1.52	0.95<br>0.27,2.1	1.02<br>0.32,2.16	1.09<br>0.49,1.94	0.85<br>0.42,2.7	TRIFLU	NA	1.06<br>0.37, 3.07	1.08<br>0.60, 1.93	NA	0.62<br>0.28, 1.41	NA	NA	NA
0.12<br>0.03,0.34	0.23<br>0.17,0.61	0.3<br>0.21,0.65	0.31<br>0.22,0.65	0.31<br>0.22,0.67	0.32<br>0.22,0.71	0.34<br>0.09,0.85	0.27<br>0.18,1.49	0.39<br>0.12,0.89	0.34<br>0.21,1.11	0.42<br>0.15,0.87	0.38<br>0.23,1.22	0.48<br>0.12,1.21	0.43<br>0.26,1.09	0.45<br>0.27,1.12	0.47<br>0.28,1.12	0.51<br>0.29,1.37	0.51<br>0.28,1.54	0.74<br>0.18,1.77	0.71<br>0.22,1.54	0.58<br>0.29,2.44	0.65<br>0.32,2.44	0.68<br>0.31,3.57	0.72<br>0.35,2.5	0.72<br>0.34,2.78	FPX	NA	NA	NA	NA	NA	NA	NA
0.12<br>0.02,0.39	0.22<br>0.17,0.69	0.28<br>0.19,0.79	0.29<br>0.2,0.78	0.29<br>0.06,0.8	0.3<br>0.2,0.85	0.35<br>0.07,1.02	0.44<br>0.03,1.6	0.4<br>0.08,1.08	0.32<br>0.2,1.2	0.44<br>0.09,1.18	0.47<br>0.08,1.4	0.49<br>0.09,1.42	0.5<br>0.11,1.32	0.4<br>0.23,1.39	0.42<br>0.24,1.41	0.63<br>0.14,1.59	0.47<br>0.25,1.79	0.75<br>0.14,1.99	0.7<br>0.18,1.67	0.53<br>0.26,2.7	0.58<br>0.28,2.7	1.06<br>0.2,2.58	0.64<br>0.29,3.03	0.69<br>0.33,2.5	1.26<br>0.21,3.04	PEN	NA	NA	NA	NA	NA	NA
0.11<br>0.03,0.26	0.24<br>0.18,0.52	0.3<br>0.22,0.52	0.32<br>0.24,0.5	0.32<br>0.24,0.52	0.33<br>0.24,0.55	0.31<br>0.12,0.66	0.39<br>0.04,1.25	0.35<br>0.15,0.67	0.36<br>0.23,0.82	0.4<br>0.17,0.75	0.42<br>0.13,0.98	0.44<br>0.14,0.97	0.45<br>0.3,0.83	0.46<br>0.31,0.86	0.48<br>0.33,0.85	0.53<br>0.34,1.03	0.53<br>0.33,1.16	0.68<br>0.23,1.43	0.63<br>0.34,1.04	0.61<br>0.32,1.85	0.69<br>0.4,1.61	0.74<br>0.36,2.5	0.75<br>0.4,1.96	0.78<br>0.44,1.79	1.14<br>0.33,2.42	0.85<br>0.39,3.57	LOX	NA	NA	0.77<br>0.40, 1.49	NA	NA
0.11<br>0.01,0.38	0.2<br>0.16,0.68	0.24<br>0.17,0.79	0.25<br>0.18,0.79	0.25<br>0.18,0.81	0.26<br>0.18,0.86	0.31<br>0.04,1.02	0.23<br>0.16,1.54	0.35<br>0.05,1.06	0.27<br>0.18,1.25	0.39<br>0.06,1.17	0.29<br>0.19,1.39	0.43<br>0.06,1.41	0.33<br>0.2,1.33	0.34<br>0.2,1.39	0.35<br>0.21,1.41	0.38<br>0.21,1.67	0.38<br>0.21,1.85	0.67<br>0.09,1.96	0.64<br>0.1,1.81	0.42<br>0.22,2.78	0.46<br>0.23,2.78	0.48<br>0.23,3.85	0.51<br>0.25,2.94	0.51<br>0.24,3.12	1.12<br>0.14,2.99	0.55<br>0.24,5.26	0.61<br>0.27,3.57	FLU	1.06<br>0.60, 1.85	NA	NA	NA
0.1<br>0.04,0.19	0.24<br>0.18,0.45	0.3<br>0.25,0.4	0.32<br>0.29,0.36	0.32<br>0.28,0.38	0.33<br>0.28,0.41	0.28<br>0.14,0.51	0.28<br>0.18,1.06	0.32<br>0.2,0.47	0.35<br>0.24,0.74	0.36<br>0.23,0.53	0.39<br>0.26,0.79	0.4<br>0.17,0.78	0.45<br>0.35,0.62	0.46<br>0.36,0.62	0.48<br>0.41,0.58	0.53<br>0.4,0.75	0.53<br>0.36,0.91	0.62<br>0.27,1.16	0.59<br>0.37,0.89	0.61<br>0.34,1.54	0.67<br>0.39,1.49	0.71<br>0.36,2.33	0.75<br>0.45,1.49	0.75<br>0.43,1.69	1.05<br>0.39,2.05	0.83<br>0.38,3.23	0.91<br>0.57,1.72	1.52<br>0.31,3.31	HAL	NA	0.80<br>0.33, 1.96	0.42<br>0.23, 0.78
0.07<br>0.01,0.21	0.12<br>0.02,0.38	0.16<br>0.04,0.42	0.17<br>0.05,0.42	0.17<br>0.05,0.44	0.18<br>0.05,0.46	0.21<br>0.05,0.57	0.26<br>0.02,0.96	0.23<br>0.06,0.61	0.22<br>0.06,0.54	0.26<br>0.07,0.68	0.28<br>0.05,0.8	0.29<br>0.06,0.81	0.3<br>0.08,0.75	0.31<br>0.21,0.78	0.32<br>0.09,0.8	0.38<br>0.1,0.92	0.35<br>0.22,1.01	0.46<br>0.09,1.21	0.43<br>0.12,1.01	0.54<br>0.1,1.5	0.57<br>0.12,1.46	0.7<br>0.12,1.93	0.46<br>0.25,1.67	0.47<br>0.25,1.67	0.79<br>0.15,2.06	0.89<br>0.14,2.41	0.73<br>0.21,1.64	1.17<br>0.13,3.14	0.76<br>0.22,1.67	THIOTH	NA	NA
0.17<br>0.16,0.27	0.18<br>0.16,0.36	0.21<br>0.17,0.39	0.22<br>0.17,0.39	0.22<br>0.17,0.4	0.22<br>0.18,0.42	0.16<br>0.04,0.43	0.2<br>0.16,0.75	0.18<br>0.05,0.46	0.23<br>0.17,0.58	0.2<br>0.05,0.51	0.25<br>0.18,0.64	0.23<br>0.05,0.64	0.27<br>0.19,0.61	0.28<br>0.2,0.61	0.28<br>0.2,0.62	0.3<br>0.21,0.74	0.3<br>0.2,0.81	0.35<br>0.08,0.97	0.33<br>0.09,0.8	0.33<br>0.2,1.22	0.38<br>0.24,0.95	0.38<br>0.21,1.67	0.39<br>0.23,1.28	0.4<br>0.23,1.33	0.43<br>0.23,1.75	0.43<br>0.22,2.22	0.47<br>0.26,1.39	0.47<br>0.22,3.33	0.49<br>0.28,1.32	0.63<br>0.29,3.12	CPX	NA
0.01<br>0,0.04	0.02<br>0,0.07	0.16<br>0.15,0.21	0.16<br>0.15,0.21	0.03<br>0.01,0.09	0.16<br>0.15,0.22	0.04<br>0.01,0.11	0.05<br>0,0.21	0.04<br>0.01,0.12	0.17<br>0.15,0.25	0.05<br>0.01,0.13	0.05<br>0.01,0.16	0.05<br>0.01,0.17	0.06<br>0.01,0.15	0.18<br>0.16,0.27	0.18<br>0.16,0.27	0.07<br>0.02,0.19	0.18<br>0.16,0.31	0.09<br>0.02,0.26	0.08<br>0.02,0.21	0.19<br>0.16,0.4	0.11<br>0.02,0.33	0.14<br>0.02,0.47	0.2<br>0.16,0.42	0.2<br>0.16,0.44	0.16<br>0.02,0.5	0.18<br>0.02,0.62	0.15<br>0.03,0.41	0.24<br>0.02,0.91	0.15<br>0.04,0.39	0.25<br>0.18,0.88	0.35<br>0.05,1.09	PIM

Table 11.7: Use of antiparkinson medication

Treatments are ranked according to their surface under the curve cumulative ranking (SUCRA) for use of antiparkinson medication starting with the best. Results of the **network meta-analysis** are presented in the **left lower half** and results from **pairwise comparisons** in the **upper right half**, if available. Comparisons between treatments should be read from left to right and the estimate is in the cell in common between the column-defining treatment and the row-defining treatment. Risk ratios (RRs) lower than 1 favor the column-defining treatment. Cells in bold print indicate significant results.

NA=not available. Drug abbreviations: AMI=Amisulpride, ARI=Aripiprazole, ASE=Asenapine, BRE=Brexpiprazole, CAR=Cariprazine, CPZ=Chlorpromazine, CPX=Clopenthixol, CLO=Clozapine, FPX=Flupenthixol, HAL=Haloperidol, ILO=Iloperidone, LEV=Levomepromazine, LOX=Loxapine, LUR=Lurasidone, MOL=Molindone, OLA=Olanzapine, PAL=Paliperidone, PEN=Penfluridol, PERA=Perazine, PERPH=Perphenazine, PIM=Pimozide, PBO=Placebo, QUE=Quetiapine, RIS=Risperidone, SER=Sertindole, SUL=Sulpiride, THIOR=Thioridazine, THIOTH=Thiothixene, TRIFLU=Trifluoperazine, ZIP=Ziprasidone, ZOT=Zotepine, ZUC=Zuclopenthixol.

Table 11.8: Akathisia

CLO	NA	NA	NA	NA	NA	NA	NA	NA	NA	NA	NA	NA	NA	NA	0.38<br>0.02, 8.02	NA	NA	NA	NA	NA	0.20<br>0.01, 3.13	NA	NA	NA	NA	NA	NA	NA	NA	NA
0.68<br>0,3.82	PERA	NA	NA	NA	NA	NA	NA	0.40<br>0.09, 1.86	NA	NA	NA	NA	NA	0.20<br>0.03, 1.54	NA	NA	NA	NA	NA	NA	NA	NA	NA	NA	NA	NA	NA	NA	NA	NA
0.24<br>0,1.78	0.76<br>0.08,2.51	SER	NA	1.15<br>0.66, 2.03	0.52<br>0.24, 1.10	NA	NA	NA	NA	NA	NA	NA	NA	NA	NA	NA	NA	NA	NA	NA	**0.19<br>0.12, 0.30**	NA	NA	NA	NA	NA	NA	NA	NA	NA
0.4<br>0,2.73	0.37<br>0.13,5.26	0.97<br>0.35,5.26	ILO	NA	4.52<br>0.24, 84.29	NA	NA	NA	NA	NA	**0.20<br>0.07, 0.59**	NA	NA	NA	NA	NA	NA	NA	NA	NA	NA	NA	NA	NA	NA	NA	NA	NA	NA	NA
0.18<br>0,1.35	0.29<br>0.12,1.96	0.74<br>0.47,1.25	0.77<br>0.15,2.11	OLA	0.81<br>0.53, 1.25	3.29<br>0.14, 77.10	NA	NA	**0.54<br>0.32, 0.89**	0.20<br>0.01, 4.03	**0.43<br>0.22, 0.84**	NA	NA	NA	NA	**0.06<br>0.00, 0.96**	NA	NA	NA	NA	**0.32<br>0.24, 0.42**	**0.43<br>0.21, 0.88**	NA	NA	NA	NA	NA	NA	0.17<br>0.01, 3.35	NA
0.17<br>0,1.31	0.54<br>0.06,1.8	0.71<br>0.45,1.22	0.74<br>0.15,2.03	0.98<br>0.71,1.32	PBO	0.71<br>0.31, 1.63	0.88<br>0.57, 1.36	2.00<br>0.18, 21.66	1.03<br>0.72, 1.48	0.66<br>0.44, 1.00	0.80<br>0.45, 1.45	NA	**0.44<br>0.24, 0.81**	NA	2.00<br>0.18, 21.66	**0.41<br>0.26, 0.64**	0.11<br>0.01, 1.99	**0.35<br>0.22, 0.57**	NA	0.17<br>0.02, 1.29	**0.35<br>0.26, 0.48**	**0.27<br>0.16, 0.44**	NA	NA	NA	NA	NA	NA	NA	NA
0.17<br>0,1.28	0.52<br>0.06,1.77	0.68<br>0.4,1.32	0.73<br>0.13,2.09	0.96<br>0.58,1.5	0.99<br>0.63,1.46	QUE	0.65<br>0.23, 1.83	NA	NA	NA	NA	NA	NA	NA	**0.37<br>0.15, 0.90**	**0.46<br>0.23, 0.91**	NA	NA	NA	NA	**0.26<br>0.16, 0.43**	**0.22<br>0.05, 0.93**	NA	NA	NA	NA	NA	NA	NA	NA
0.07<br>0.07,1.02	0.23<br>0.1,1.54	0.54<br>0.3,1.12	0.36<br>0.16,1.75	0.71<br>0.43,1.32	0.74<br>0.48,1.25	0.76<br>0.44,1.47	BRE	NA	NA	1.39<br>0.27, 7.03	NA	NA	NA	NA	NA	NA	NA	NA	NA	NA	NA	NA	NA	NA	NA	NA	NA	NA	NA	NA
0.14<br>0,1.05	0.38<br>0.05,1.21	0.65<br>0.17,1.71	0.63<br>0.07,2.18	0.84<br>0.24,2.08	0.87<br>0.26,2.09	0.91<br>0.26,2.24	1.17<br>0.31,2.94	ZOT	NA	NA	NA	NA	NA	NA	1.00<br>0.06, 15.74	NA	1.12<br>0.39, 3.17	NA	NA	NA	0.07<br>0.00, 1.15	NA	NA	NA	NA	NA	NA	NA	NA	NA
**0.12<br>0,0.94**	0.21<br>0.1,1.37	**0.5<br>0.29,0.99**	0.53<br>0.09,1.54	0.7<br>0.4,1.11	0.68<br>0.44,1.1	0.69<br>0.4,1.33	0.97<br>0.45,1.75	0.78<br>0.32,2.94	PAL	NA	NA	NA	NA	NA	NA	NA	NA	NA	NA	NA	NA	NA	NA	NA	NA	NA	NA	NA	NA	NA
**0.07<br>0.07,0.66**	**0.17<br>0.09,0.96**	**0.38<br>0.24,0.69**	0.26<br>0.13,1.11	**0.49<br>0.34,0.79**	**0.51<br>0.37,0.76**	**0.52<br>0.33,0.92**	0.64<br>0.38,1.25	0.59<br>0.26,2.04	0.7<br>0.43,1.37	ARI	1.27<br>0.47, 3.40	NA	NA	NA	NA	1.20<br>0.67, 2.16	NA	0.66<br>0.33, 1.33	NA	NA	**0.42<br>0.23, 0.77**	NA	NA	NA	NA	NA	NA	NA	NA	NA
**0.08<br>0,0.61**	**0.25<br>0.03,0.88**	**0.36<br>0.19,0.6**	**0.34<br>0.07,0.94**	**0.46<br>0.3,0.68**	**0.48<br>0.32,0.68**	**0.5<br>0.29,0.82**	0.65<br>0.33,1.14	0.55<br>0.25,1.85	0.71<br>0.38,1.21	0.95<br>0.58,1.46	ZIP	NA	NA	NA	NA	1.04<br>0.73, 1.48	NA	NA	NA	NA	**0.44<br>0.29, 0.67**	NA	NA	NA	NA	NA	NA	NA	NA	NA
**0.08<br>0,0.62**	**0.25<br>0.02,0.91**	**0.37<br>0.13,0.79**	0.36<br>0.05,1.17	**0.48<br>0.2,0.98**	**0.49<br>0.21,0.98**	0.51<br>0.21,1.03	0.67<br>0.24,1.46	0.53<br>0.23,1.85	0.73<br>0.27,1.57	0.99<br>0.39,1.97	0.89<br>0.45,2.17	THIOR	NA	NA	0.82<br>0.35, 1.93	NA	0.61<br>0.23, 1.58	NA	NA	0.72<br>0.13, 4.03	0.80<br>0.46, 1.42	NA	NA	NA	NA	NA	NA	NA	NA	NA
**0.07<br>0.07,0.59**	**0.15<br>0.09,0.88**	**0.31<br>0.18,0.68**	0.22<br>0.11,1.04	**0.4<br>0.24,0.82**	**0.42<br>0.25,0.81**	**0.43<br>0.24,0.93**	0.52<br>0.28,1.25	0.48<br>0.21,1.85	0.57<br>0.3,1.35	0.9<br>0.39,1.7	0.84<br>0.45,1.85	0.82<br>0.37,2.44	ASE	NA	NA	NA	NA	NA	NA	NA	0.58<br>0.30, 1.13	NA	NA	NA	NA	NA	NA	NA	NA	NA
**0.07<br>0.07,0.56**	**0.15<br>0.09,0.75**	**0.3<br>0.17,0.69**	0.21<br>0.11,1.02	**0.39<br>0.22,0.83**	**0.4<br>0.23,0.85**	**0.41<br>0.23,0.94**	0.5<br>0.26,1.28	0.46<br>0.2,1.79	0.54<br>0.28,1.39	0.74<br>0.39,1.79	0.8<br>0.42,1.89	0.78<br>0.35,2.38	0.85<br>0.4,2.56	AMI	NA	0.54<br>0.11, 2.74	NA	NA	NA	NA	0.65<br>0.35, 1.20	NA	NA	NA	NA	NA	NA	NA	NA	NA
**0.07<br>0.07,0.53**	**0.14<br>0.09,0.75**	**0.29<br>0.17,0.64**	**0.21<br>0.11,0.97**	**0.37<br>0.22,0.77**	**0.39<br>0.23,0.77**	**0.4<br>0.24,0.79**	0.56<br>0.22,1.15	0.46<br>0.21,1.54	0.52<br>0.28,1.3	0.83<br>0.36,1.6	0.78<br>0.42,1.72	0.79<br>0.43,1.79	1.03<br>0.37,2.2	1.08<br>0.37,2.33	CPZ	NA	NA	NA	NA	0.84<br>0.29, 2.47	2.00<br>0.22, 18.57	NA	NA	0.50<br>0.05, 5.23	0.10<br>0.01, 1.79	0.74<br>0.05, 10.91	NA	NA	NA	NA
**0.06<br>0,0.47**	**0.19<br>0.02,0.69**	**0.29<br>0.19,0.48**	**0.26<br>0.05,0.77**	**0.36<br>0.23,0.52**	**0.37<br>0.25,0.5**	**0.39<br>0.23,0.6**	**0.5<br>0.26,0.85**	0.44<br>0.2,1.41	**0.55<br>0.3,0.91**	0.74<br>0.45,1.11	0.77<br>0.54,1.12	0.74<br>0.38,1.75	0.93<br>0.42,1.72	0.97<br>0.42,1.83	1.01<br>0.45,1.85	RIS	NA	0.99<br>0.51, 1.92	NA	NA	**0.65<br>0.45, 0.93**	NA	NA	NA	NA	NA	NA	NA	NA	NA
**0.06<br>0,0.49**	**0.19<br>0.02,0.7**	**0.29<br>0.09,0.7**	**0.28<br>0.04,0.97**	**0.38<br>0.13,0.85**	**0.39<br>0.14,0.87**	**0.41<br>0.14,0.92**	0.53<br>0.16,1.28	0.43<br>0.21,1.3	0.58<br>0.18,1.39	0.79<br>0.26,1.76	0.68<br>0.33,1.92	0.86<br>0.33,1.75	0.98<br>0.28,2.34	1.03<br>0.29,2.45	1.02<br>0.36,2.18	1.09<br>0.38,2.35	THIOTH	NA	NA	0.93<br>0.27, 3.15	NA	NA	0.78<br>0.38, 1.60	NA	NA	NA	NA	NA	NA	NA
**0.07<br>0.07,0.42**	**0.13<br>0.08,0.58**	**0.24<br>0.16,0.44**	**0.18<br>0.1,0.67**	**0.3<br>0.2,0.51**	**0.32<br>0.22,0.49**	**0.32<br>0.2,0.58**	**0.41<br>0.19,0.78**	0.36<br>0.17,1.23	**0.42<br>0.25,0.83**	0.61<br>0.33,1	0.61<br>0.38,1.09	0.6<br>0.29,1.54	0.77<br>0.31,1.53	0.81<br>0.3,1.69	0.7<br>0.36,1.72	0.79<br>0.5,1.37	0.74<br>0.33,2.27	CAR	NA	NA	NA	NA	NA	NA	NA	NA	NA	NA	NA	NA
**0.06<br>0,0.42**	**0.17<br>0.01,0.78**	**0.18<br>0.1,0.85**	0.24<br>0.02,1	0.32<br>0.05,1.06	0.23<br>0.11,1.09	0.24<br>0.12,1.15	0.45<br>0.07,1.49	0.27<br>0.12,1.89	0.5<br>0.07,1.61	0.68<br>0.11,2.14	0.43<br>0.17,2.38	0.43<br>0.17,2.56	0.85<br>0.12,2.68	0.89<br>0.12,2.87	0.87<br>0.15,2.6	0.54<br>0.2,3.12	0.53<br>0.2,3.45	1.18<br>0.19,3.52	PERPH	NA	NA	NA	NA	1.00<br>0.21, 4.68	NA	0.66<br>0.37, 1.17	NA	NA	NA	NA
**0.05<br>0,0.4**	**0.12<br>0.08,0.58**	**0.22<br>0.13,0.56**	**0.22<br>0.03,0.76**	**0.27<br>0.15,0.67**	**0.28<br>0.16,0.68**	**0.29<br>0.16,0.72**	**0.41<br>0.13,0.99**	0.33<br>0.16,1.22	0.37<br>0.19,1.09	0.61<br>0.21,1.39	0.54<br>0.27,1.49	0.55<br>0.27,1.52	0.77<br>0.22,1.85	0.81<br>0.22,1.97	0.78<br>0.3,1.62	0.68<br>0.33,1.89	0.69<br>0.33,2	1.07<br>0.34,2.42	0.79<br>0.28,5	LOX	NA	NA	0.70<br>0.29, 1.68	NA	NA	NA	NA	NA	NA	NA
**0.04<br>0,0.31**	**0.11<br>0.08,0.45**	**0.21<br>0.15,0.31**	**0.16<br>0.09,0.51**	**0.26<br>0.21,0.33**	**0.27<br>0.22,0.33**	**0.27<br>0.2,0.39**	**0.32<br>0.17,0.54**	**0.3<br>0.16,0.88**	**0.35<br>0.24,0.58**	**0.47<br>0.3,0.71**	**0.51<br>0.37,0.72**	0.5<br>0.28,1.05	0.59<br>0.28,1.07	0.62<br>0.28,1.15	0.64<br>0.31,1.16	**0.65<br>0.49,0.91**	0.61<br>0.31,1.61	0.83<br>0.47,1.34	0.7<br>0.25,4	0.78<br>0.38,2	HAL	1.22<br>0.63, 2.35	1.33<br>0.44, 4.07	0.76<br>0.22, 2.64	1.00<br>0.49, 2.04	NA	0.47<br>0.14, 1.55	NA	NA	**0.33<br>0.14, 0.78**
**0.04<br>0,0.29**	**0.11<br>0.08,0.46**	**0.2<br>0.14,0.34**	**0.16<br>0.03,0.5**	**0.25<br>0.18,0.38**	**0.25<br>0.19,0.37**	**0.26<br>0.18,0.43**	**0.31<br>0.14,0.57**	**0.29<br>0.14,0.92**	**0.33<br>0.21,0.62**	**0.46<br>0.24,0.79**	**0.48<br>0.3,0.83**	0.46<br>0.24,1.16	0.58<br>0.24,1.15	0.61<br>0.23,1.25	0.63<br>0.26,1.26	0.6<br>0.39,1.04	0.57<br>0.27,1.67	0.81<br>0.39,1.44	0.66<br>0.24,4	0.93<br>0.3,2.08	0.99<br>0.59,1.51	LUR	NA	NA	NA	NA	NA	NA	NA	NA
**0.03<br>0,0.25**	**0.1<br>0.01,0.39**	**0.15<br>0.05,0.36**	**0.15<br>0.02,0.51**	**0.2<br>0.07,0.44**	**0.21<br>0.08,0.45**	**0.22<br>0.08,0.49**	**0.28<br>0.09,0.67**	**0.25<br>0.13,0.85**	**0.31<br>0.1,0.73**	**0.42<br>0.14,0.95**	0.39<br>0.21,1	0.47<br>0.16,1.08	0.53<br>0.15,1.3	0.55<br>0.16,1.36	0.55<br>0.2,1.19	0.58<br>0.21,1.28	0.61<br>0.2,1.39	0.74<br>0.24,1.71	0.97<br>0.19,2.75	0.77<br>0.29,1.66	0.9<br>0.35,1.83	0.97<br>0.33,2.13	TRIFLU	0.67<br>0.12, 3.61	1.20<br>0.49, 2.96	0.88<br>0.31, 2.53	NA	NA	NA	NA
**0.03<br>0,0.25**	**0.1<br>0.01,0.43**	**0.15<br>0.04,0.41**	**0.14<br>0.02,0.55**	**0.2<br>0.05,0.5**	**0.2<br>0.05,0.51**	**0.21<br>0.06,0.55**	**0.28<br>0.07,0.74**	**0.22<br>0.11,0.95**	**0.3<br>0.07,0.82**	0.41<br>0.1,1.07	0.35<br>0.17,1.14	0.35<br>0.16,1.23	0.52<br>0.12,1.43	0.55<br>0.12,1.53	0.54<br>0.14,1.37	0.57<br>0.16,1.43	0.42<br>0.18,1.75	0.73<br>0.18,1.9	0.89<br>0.17,2.48	0.8<br>0.18,2.11	0.88<br>0.25,2.06	0.95<br>0.24,2.41	0.79<br>0.33,2.78	SUL	NA	NA	NA	NA	NA	NA
**0.03<br>0,0.24**	**0.1<br>0.07,0.42**	**0.15<br>0.1,0.38**	**0.14<br>0.02,0.49**	**0.19<br>0.11,0.46**	**0.19<br>0.12,0.47**	**0.19<br>0.12,0.51**	**0.26<br>0.07,0.67**	**0.22<br>0.11,0.87**	**0.24<br>0.13,0.72**	**0.39<br>0.11,0.96**	0.34<br>0.17,1.01	0.34<br>0.17,1.15	0.48<br>0.12,1.28	0.51<br>0.12,1.34	0.51<br>0.15,1.22	0.43<br>0.21,1.28	0.42<br>0.19,1.54	0.68<br>0.19,1.69	0.49<br>0.18,3.12	0.74<br>0.19,1.83	0.83<br>0.27,1.83	0.89<br>0.25,2.12	0.79<br>0.37,2.27	0.76<br>0.29,3.7	MOL	NA	NA	NA	NA	NA
**0.03<br>0,0.23**	**0.09<br>0.07,0.45**	**0.13<br>0.09,0.46**	**0.13<br>0.01,0.55**	**0.18<br>0.03,0.56**	**0.16<br>0.1,0.58**	**0.17<br>0.1,0.61**	**0.25<br>0.04,0.81**	0.18<br>0.1,1	**0.28<br>0.05,0.89**	0.38<br>0.07,1.17	0.28<br>0.13,1.25	0.28<br>0.13,1.35	0.47<br>0.07,1.53	0.5<br>0.08,1.64	0.49<br>0.09,1.43	0.34<br>0.15,1.59	0.34<br>0.15,1.79	0.67<br>0.11,2.03	0.53<br>0.27,1.47	0.71<br>0.13,2.08	0.81<br>0.16,2.28	0.87<br>0.15,2.53	0.65<br>0.26,2.63	0.66<br>0.25,3.33	1.17<br>0.2,3.38	PEN	NA	NA	NA	NA
**0.02<br>0,0.15**	**0.06<br>0,0.31**	**0.09<br>0.07,0.36**	**0.08<br>0,0.38**	**0.11<br>0.01,0.42**	**0.11<br>0.08,0.45**	**0.11<br>0.08,0.48**	**0.15<br>0.01,0.61**	**0.11<br>0.08,0.77**	**0.17<br>0.02,0.66**	**0.23<br>0.02,0.88**	**0.15<br>0.09,0.93**	0.15<br>0.09,1.09	0.29<br>0.02,1.15	0.31<br>0.03,1.22	0.32<br>0.03,1.24	0.18<br>0.09,1.2	0.17<br>0.09,1.52	0.41<br>0.04,1.54	0.61<br>0.03,2.65	0.47<br>0.04,1.88	0.5<br>0.05,1.78	0.54<br>0.05,1.94	0.27<br>0.11,2.7	0.27<br>0.11,3.45	0.8<br>0.06,3.07	1.01<br>0.05,3.9	PIM	0.80<br>0.29, 2.20	NA	NA
**0.02<br>0,0.13**	**0.07<br>0.07,0.41**	**0.08<br>0.07,0.51**	**0.07<br>0.07,0.53**	**0.08<br>0.07,0.64**	**0.08<br>0.07,0.65**	**0.08<br>0.07,0.7**	**0.17<br>0,0.91**	0.09<br>0.07,1.04	**0.09<br>0.07,0.99**	0.25<br>0.01,1.28	0.1<br>0.07,1.37	0.1<br>0.07,1.56	0.31<br>0.01,1.66	0.33<br>0.01,1.73	0.34<br>0.01,1.77	0.11<br>0.07,1.79	0.11<br>0.07,2.08	0.44<br>0.01,2.19	0.12<br>0.07,3.57	0.12<br>0.08,2.7	0.14<br>0.08,2.78	0.14<br>0.08,3.03	0.15<br>0.08,3.85	0.15<br>0.08,4.76	0.15<br>0.08,4.76	0.16<br>0.08,6.25	0.43<br>0.15,4.35	FLU	NA	NA
**0.01<br>0,0.06**	**0.02<br>0,0.1**	**0.03<br>0,0.1**	**0.03<br>0,0.13**	**0.04<br>0.01,0.13**	**0.04<br>0.01,0.14**	**0.04<br>0.01,0.15**	**0.06<br>0.01,0.2**	**0.07<br>0.01,0.25**	**0.06<br>0.01,0.22**	**0.09<br>0.01,0.29**	**0.09<br>0.01,0.3**	**0.1<br>0.01,0.36**	**0.11<br>0.02,0.38**	**0.12<br>0.02,0.4**	**0.12<br>0.02,0.41**	**0.12<br>0.02,0.4**	**0.14<br>0.02,0.5**	**0.16<br>0.02,0.51**	**0.23<br>0.02,0.98**	**0.18<br>0.02,0.65**	**0.19<br>0.03,0.6**	**0.21<br>0.03,0.67**	**0.26<br>0.03,0.9**	0.3<br>0.03,1.13	0.3<br>0.03,1.1	0.39<br>0.03,1.58	0.92<br>0.04,3.73	2.16<br>0.03,7.75	ZUC	NA
**0.01<br>0,0.03**	**0.07<br>0.07,0.28**	**0.07<br>0.07,0.37**	**0.07<br>0.07,0.34**	**0.07<br>0.07,0.48**	**0.07<br>0.07,0.5**	**0.07<br>0.07,0.53**	**0.08<br>0,0.65**	**0.07<br>0.07,0.74**	**0.07<br>0.07,0.72**	**0.13<br>0,0.95**	0.07<br>0.07,1.02	0.07<br>0.07,1.1	0.16<br>0,1.16	0.18<br>0,1.27	0.18<br>0,1.27	0.07<br>0.07,1.35	0.07<br>0.07,1.47	0.23<br>0,1.62	0.07<br>0.07,2.22	0.07<br>0.07,1.82	0.07<br>0.07,2.08	0.07<br>0.07,2.17	0.07<br>0.07,2.63	0.07<br>0.07,3.12	0.07<br>0.07,3.23	0.07<br>0.07,3.7	0.07<br>0.07,8.33	0.07<br>0.07,20	0.07<br>0.07,20	FPX

Table 11.8: Akathisia
Treatments are ranked according to their surface under the curve cumulative ranking (SUCRA) for akathisia starting with the best. Drugs are reported in order of efficacy ranking. Results of the **network meta-analysis** are presented in the **left lower half** and results from **pairwise comparisons** in the **upper right half**, if available. Comparisons between treatments should be read from left to right and the estimate is in the cell in common between the column-defining treatment and the row-defining treatment risk ratios (RRs) lower than 1 favor the column-defining treatment. Cells in bold print indicate significant results.
NA=not available. Drug abbreviations: AMI=Amisulpride, ARI=Aripiprazole, ASE=Asenapine, BRE=Brexpiprazole, CAR=Cariprazine, CPZ=Chlorpromazine, CLO=Clozapine, FPX=Flupenthixol, FLU=Fluphenazine, HAL=Haloperidol, ILO=Iloperidone, LOX=Loxapine, LUR=Lurasidone, MOL=Molindone, OLA=Olanzapine, PAL=Paliperidone, PEN=Penfluridol, PERA=Perazine, PERPH=Perphenazine, PIM=Pimozide, PBO=Placebo, QUE=Quetiapine, RIS=Risperidone, SER=Sertindole, SUL=Sulpiride, THIOR=Thioridazine, THIOTH=Thiothixene, TRIFLU=Trifluoperazine, ZIP=Ziprasidone, ZOT=Zotepine, ZUC=Zuclopenthixol.

Table 11.9: Prolactin elevation

CLO	**-35.44<br>-53.79,-17.09**	NA	NA	NA	NA	NA	NA	NA	NA	NA	NA	NA	NA	NA	NA	NA	NA	NA	NA	NA
**-34.82<br>-52.03,-17.26**	ZOT	NA	NA	**-30.00<br>-48.39,-11.61**	NA	NA	NA	NA	NA	NA	NA	NA	NA	NA	NA	NA	NA	NA	NA	NA
**-69.95<br>-113.38,-25.95**	-35.13<br>-75.39,5.57	ARI	NA	NA	-2.33<br>-15.43, 10.76	NA	-4.65<br>-10.28, 0.99	-4.65<br>-13.08, 3.77	NA	-7.20<br>-21.82, 7.42	NA	NA	NA	NA	NA	NA	**-24.93<br>-36.42,-13.44**	NA	**-55.55<br>-64.17,-46.93**	NA
**-66.6<br>-116.9,-15.58**	-31.78<br>-79.37,16.16	3.35<br>-25.3,31.87	FPX	NA	NA	NA	NA	NA	NA	NA	23.20<br>-32.01, 78.41	NA	NA	NA	NA	NA	NA	**-52.90<br>-85.97,-19.83**	NA	NA
**-63.6<br>-87.76,-39.28**	**-28.78<br>-46.1,-11.49**	6.35<br>-30.49,43.07	2.99<br>-41.92,48.12	PERA	NA	NA	NA	NA	NA	NA	NA	NA	NA	NA	NA	NA	NA	**-48.00<br>-86.71, -9.29**	NA	NA
**-73.86<br>-117.64,-29.86**	-39.04<br>-79.44,1.7	-3.91<br>-10.58,2.84	-7.26<br>-36.29,22.13	-10.25<br>-47.38,26.87	CAR	NA	-3.08<br>-10.33, 4.18	NA	NA	NA	NA	NA	NA	NA	NA	NA	NA	NA	**-39.46<br>-52.35,-26.57**	NA
**-75.88<br>-119.23,-32.27**	**-41.06<br>-81.53,-0.52**	**-5.93<br>-10.98,-0.91**	-9.28<br>-37.66,19.47	-12.27<br>-49.09,24.59	-2.02<br>-8.87,4.84	QUE	-0.94<br>-5.67, 3.78	NA	NA	NA	**-40.06<br>-66.43,-13.70**	NA	NA	-10.00<br>-27.59, 7.59	-7.98<br>-19.78, 3.82	NA	**-15.91<br>-22.44, -9.38**	NA	**-46.20<br>-56.72,-35.68**	NA
**-77.05<br>-120.23,-33.54**	**-42.23<br>-82.42,-1.78**	**-7.1<br>-11.17,-3.09**	-10.45<br>-38.75,18.22	-13.44<br>-50.27,23.64	-3.19<br>-9.21,2.8	-1.17<br>-4.52,2.27	PBO	-3.27<br>-9.16, 2.63	NA	-8.20<br>-20.52, 4.12	**-4.86<br>-8.61, -1.10**	**-8.09<br>-15.38, -0.81**	-5.75<br>-11.79, 0.30	NA	**-7.55<br>-12.25, -2.86**	**-8.62<br>-16.56, -0.68**	**-16.95<br>-21.29,-12.62**	NA	**-35.74<br>-40.63,-30.85**	**-48.35<br>-53.97,-42.72**
**-78<br>-121.42,-33.97**	**-43.18<br>-83.53,-2.09**	**-8.05<br>-13.28,-2.88**	-11.4<br>-40.08,17.48	-14.39<br>-51.42,22.63	-4.14<br>-11.67,3.17	-2.12<br>-7.86,3.54	-0.95<br>-5.62,3.64	BRE	NA	NA	NA	NA	NA	NA	NA	NA	NA	NA	NA	NA
-73.14<br>-168.76,23.83	-38.32<br>-132.16,56.76	-3.19<br>-89.24,82.63	-6.54<br>-97.86,84.86	-9.53<br>-102.37,84.2	0.72<br>-85.58,86.5	2.74<br>-83.99,88.18	3.91<br>-82.66,89.56	4.86<br>-81.61,90.81	PIM	NA	NA	NA	NA	NA	NA	NA	-55.66<br>-158.65, 47.33	NA	NA	NA
**-79.8<br>-123.36,-35.93**	**-44.98<br>-85.31,-4.23**	**-9.85<br>-15.62,-4.07**	-13.2<br>-41.97,15.47	-16.19<br>-53.22,21.04	-5.94<br>-13.64,1.66	-3.92<br>-9.58,1.65	-2.75<br>-7.66,2.14	-1.8<br>-8.34,4.84	-6.66<br>-92.02,79.9	ZIP	26.80<br>-13.38, 66.98	-0.70<br>-12.83, 11.43	NA	NA	NA	NA	**-13.71<br>-20.81, -6.60**	NA	**-44.16<br>-54.50,-33.82**	NA
**-81.52<br>-125.09,-37.79**	**-46.7<br>-87.12,-6.1**	**-11.57<br>-16.41,-6.8**	-14.92<br>-43.3,13.58	-17.91<br>-54.85,19.18	**-7.66<br>-14.29,-1.04**	**-5.64<br>-9.94,-1.4**	**-4.47<br>-7.38,-1.6**	-3.52<br>-8.95,1.86	-8.38<br>-94.08,78.15	-1.72<br>-7.17,3.64	OLA	NA	-2.01<br>-11.13, 7.11	NA	-1.41<br>-12.83, 10.01	-8.16<br>-20.77, 4.45	**-13.67<br>-20.57, -6.76**	NA	**-18.40<br>-36.48, -0.32**	**-49.29<br>-57.07,-41.52**
**-81.84<br>-125.63,-37.81**	**-47.02<br>-87.88,-6.17**	**-11.89<br>-18.78,-4.94**	-15.24<br>-44.07,13.75	-18.23<br>-55.36,19.05	-7.98<br>-16.23,0.39	-5.96<br>-12.62,0.63	-4.79<br>-10.66,1.05	-3.84<br>-11.24,3.63	-8.7<br>-94.63,77.69	-2.04<br>-8.84,4.79	-0.32<br>-6.67,6.11	ILO	NA	NA	NA	NA	**-21.30<br>-34.95, -7.65**	NA	**-38.80<br>-54.02,-23.58**	NA
**-82.1<br>-125.76,-38.4**	**-47.28<br>-87.91,-6.54**	**-12.15<br>-18.56,-5.75**	-15.5<br>-44.35,13.35	-18.49<br>-55.6,18.2	**-8.24<br>-16.1,-0.35**	**-6.22<br>-12.3,-0.19**	**-5.05<br>-10.14,-0.01**	-4.1<br>-10.92,2.72	-8.96<br>-94.7,77.47	-2.3<br>-9.27,4.62	-0.58<br>-6,4.89	-0.26<br>-7.93,7.41	ASE	NA	NA	NA	-16.00<br>-32.55, 0.55	NA	**-60.65<br>-80.76,-40.54**	NA
**-85.75<br>-132.37,-39.18**	**-50.93<br>-94.95,-7.4**	-15.8<br>-33.26,1.38	-19.16<br>-51.83,14.21	-22.15<br>-62.94,17.94	-11.9<br>-29.92,5.87	-9.88<br>-26.7,6.73	-8.7<br>-25.75,8.16	-7.75<br>-25.42,9.84	-12.61<br>-99.55,75.33	-5.96<br>-23.64,11.68	-4.24<br>-21.42,12.88	-3.92<br>-21.94,13.7	-3.65<br>-21.4,14.11	CPZ	NA	NA	NA	NA	NA	NA
**-84.09<br>-127.88,-40.13**	**-49.27<br>-89.62,-8.72**	**-14.14<br>-19.78,-8.51**	-17.49<br>-46.04,11.47	-20.49<br>-57.84,16.59	**-10.24<br>-17.37,-3.1**	**-8.21<br>-13.25,-3.23**	**-7.04<br>-11.05,-3.03**	**-6.09<br>-12.24,-0.03**	-10.95<br>-96.71,75.49	-4.29<br>-10.49,2.06	-2.57<br>-7.27,2.15	-2.25<br>-9.42,4.78	-1.99<br>-8.44,4.41	1.66<br>-15.54,19.2	LUR	NA	-14.00<br>-28.34, 0.34	NA	NA	NA
**-87.17<br>-130.91,-43.22**	**-52.35<br>-92.5,-11.74**	**-17.22<br>-23.93,-10.33**	-20.57<br>-49.19,8.49	-23.56<br>-60.66,13.45	**-13.31<br>-21.46,-5.06**	**-11.29<br>-17.61,-4.92**	**-10.12<br>-15.63,-4.52**	**-9.17<br>-16.34,-1.98**	-14.03<br>-100.11,72.56	**-7.37<br>-14.51,-0.34**	-5.65<br>-11.55,0.26	-5.33<br>-13.3,2.58	-5.07<br>-12.41,2.42	-1.41<br>-19.1,16.44	-3.08<br>-9.79,3.67	SER	-6.99<br>-15.66, 1.68	NA	NA	NA
**-95.53<br>-138.79,-51.96**	**-60.71<br>-100.93,-20.35**	**-25.58<br>-30.18,-21.05**	**-28.94<br>-57.17,-0.68**	-31.93<br>-68.64,4.85	**-21.68<br>-28.3,-15.14**	**-19.66<br>-23.44,-15.89**	**-18.49<br>-21.39,-15.6**	**-17.54<br>-22.91,-12.13**	-22.4<br>-107.81,64.3	**-15.74<br>-20.43,-11.01**	**-14.02<br>-17.6,-10.41**	**-13.7<br>-19.88,-7.66**	**-13.43<br>-19.07,-7.82**	-9.78<br>-26.71,7.5	**-11.44<br>-16.15,-6.73**	**-8.37<br>-14.15,-2.7**	HAL	-6.50<br>-18.75, 5.75	**-9.57<br>-16.93, -2.20**	NA
**-103.92<br>-146.15,-61.72**	**-69.1<br>-107.91,-29.85**	**-33.97<br>-45.83,-22.36**	**-37.33<br>-64.79,-9.53**	**-40.32<br>-75.95,-4.91**	**-30.07<br>-42.93,-17.5**	**-28.05<br>-39.61,-16.62**	**-26.87<br>-38.19,-15.63**	**-25.92<br>-38.11,-13.88**	-30.78<br>-117.4,56.86	**-24.12<br>-36.14,-12.36**	**-22.41<br>-33.89,-11.01**	**-22.09<br>-34.45,-9.77**	**-21.82<br>-34.11,-9.49**	-18.17<br>-38.1,2.05	**-19.83<br>-31.79,-7.96**	**-16.76<br>-29.15,-4.57**	-8.39<br>-19.38,2.47	AMI	NA	NA
**-115.02<br>-158.54,-71.33**	**-80.21<br>-120.71,-39.89**	**-45.08<br>-49.71,-40.48**	**-48.43<br>-76.97,-19.93**	**-51.42<br>-88.26,-14.55**	**-41.17<br>-47.74,-34.63**	**-39.15<br>-43.46,-34.81**	**-37.98<br>-41.38,-34.64**	**-37.03<br>-42.56,-31.51**	-41.89<br>-127.52,44.36	**-35.23<br>-40.46,-29.95**	**-33.51<br>-37.71,-29.34**	**-33.19<br>-39.51,-26.79**	**-32.93<br>-38.92,-27.02**	**-29.27<br>-46.25,-11.87**	**-30.93<br>-36.08,-25.82**	**-27.86<br>-34.24,-21.56**	**-19.49<br>-23.3,-15.72**	-11.1<br>-22.56,0.64	RIS	NA
**-125.56<br>-169.23,-81.76**	**-90.74<br>-130.84,-50.13**	**-55.61<br>-62.04,-49.32**	**-58.97<br>-87.66,-30.13**	**-61.96<br>-98.79,-24.69**	**-51.71<br>-59.44,-43.99**	**-49.69<br>-55.58,-43.77**	**-48.51<br>-53.51,-43.52**	**-47.57<br>-54.43,-40.71**	-52.42<br>-137.78,33.61	**-45.77<br>-52.73,-38.83**	**-44.05<br>-49.35,-38.84**	**-43.73<br>-51.35,-36.15**	**-43.46<br>-50.5,-36.51**	**-39.81<br>-57.41,-22.02**	**-41.47<br>-47.79,-35.13**	**-38.4<br>-45.76,-30.99**	**-30.03<br>-35.73,-24.41**	**-21.64<br>-33.76,-9.31**	**-10.54<br>-16.45,-4.52**	PAL

Table 11.9: Prolactin elevation

Treatments are ranked according to their surface under the curve cumulative ranking (SUCRA) for prolactin elevation in ng/ml starting with the best. Drugs are reported in order of their ranking. Results of the **network meta-analysis** are presented in the **left lower half** and results from **pairwise comparisons** in the **upper right** half, if available. Comparisons between treatments should be read from left to right and the estimate is in the cell in common between the column-defining treatment and the row-defining treatment. In the left lower half, mean differences (MDs) lower than 0 favor the column-defining treatment, in the upper right half MDs lower than 0 favor the row defining treatment. Cells in bold print indicate significant results. NA=not available. Drug abbreviations: AMI=Amisulpride, ARI=Aripiprazole, ASE=Asenapine, BRE=Brexpiprazole, CAR=Cariprazine, CPZ=Chlorpromazine, CLO=Clozapine, FPX=Flupenthixol, FLU=Fluphenazine, HAL=Haloperidol, ILO=Iloperidone, LUR=Lurasidone, OLA=Olanzapine, PAL=Paliperidone, PERA=Perazine, PIM=Pimozide, PBO=Placebo, QUE=Quetiapine, RIS=Risperidone, SER=Sertindole, ZIP=Ziprasidone, ZOT=Zotepine.

Table 11.10: QTc prolongation

LUR	NA	NA	NA	-2.32<br>-4.70, 0.06	NA	-2.50<br>-9.10, 4.10	**-7.50<br>-12.43, -2.57**	**-5.60<br>-10.88, -0.32**	NA	NA	NA	NA	NA	NA
-0.75<br>-4.7,3.32	BRE	NA	NA	-1.48<br>-4.35, 1.40	NA	NA	NA	NA	NA	NA	NA	NA	NA	NA
-0.76<br>-5.9,4.44	-0.01<br>-5.72,5.75	CAR	NA	-0.57<br>-5.75, 4.61	NA	NA	NA	NA	**-7.07<br>-12.37, -1.77**	NA	NA	NA	NA	NA
-1.78<br>-5.65,2.13	-1.03<br>-5.61,3.49	-1.01<br>-6.69,4.55	ARI	0.55<br>-3.34, 4.43	NA	-4.87<br>-10.07, 0.34	NA	NA	**-7.25<br>-12.99, -1.51**	NA	NA	-6.50<br>-13.74, 0.74	NA	NA
-2.21<br>-4.54,0.15	-1.46<br>-4.71,1.81	-1.45<br>-6.2,3.2	-0.43<br>-3.62,2.77	PBO	0.15<br>-4.05, 4.35	**-3.15<br>-5.48, -0.82**	**-4.16<br>-6.99, -1.32**	-1.92<br>-5.24, 1.39	**-4.49<br>-7.12, -1.86**	-6.20<br>-13.30, 0.90	**-6.05<br>-8.53, -3.58**	**-10.10<br>-13.69, -6.50**	NA	**-23.84<br>-28.01,-19.67**
-3.41<br>-8.01,1.26	-2.67<br>-7.92,2.61	-2.65<br>-8.89,3.52	-1.64<br>-6.78,3.48	-1.21<br>-5.31,2.89	PAL	NA	NA	-1.55<br>-5.93, 2.83	NA	NA	NA	NA	NA	NA
**-3.89<br>-6.71,-1.03**	-3.15<br>-6.89,0.62	-3.13<br>-8.21,1.88	-2.12<br>-5.48,1.25	-1.69<br>-3.64,0.23	-0.48<br>-4.91,3.98	HAL	1.14<br>-7.44, 9.72	**-6.34<br>-11.48, -1.19**	-3.30<br>-14.66, 8.06	NA	1.30<br>-4.57, 7.17	**-9.27<br>-12.34, -6.19**	NA	**-23.82<br>-28.26,-19.37**
**-5.64<br>-8.78,-2.49**	**-4.89<br>-9.04,-0.82**	-4.88<br>-10.17,0.29	-3.86<br>-7.84,0.09	**-3.43<br>-6,-0.94**	-2.23<br>-6.97,2.54	-1.74<br>-4.81,1.23	QUE	-3.79<br>-26.02, 18.44	-3.60<br>-8.24, 1.04	NA	NA	NA	NA	NA
**-6.5<br>-9.61,-3.37**	**-5.75<br>-9.78,-1.73**	**-5.74<br>-10.98,-0.72**	**-4.72<br>-8.51,-0.93**	**-4.29<br>-6.68,-1.91**	-3.09<br>-7.2,1.03	-2.6<br>-5.24,0.06	-0.86<br>-4.1,2.46	OLA	2.50<br>-2.76, 7.76	NA	NA	**-6.88<br>-11.52, -2.23**	**-9.80<br>-15.28, -4.32**	**-19.00<br>-28.57, -9.43**
**-6.98<br>-10,-3.94**	**-6.23<br>-10.1,-2.44**	**-6.22<br>-11.01,-1.58**	**-5.21<br>-8.7,-1.7**	**-4.77<br>-6.87,-2.68**	-3.57<br>-8.11,0.95	**-3.09<br>-5.69,-0.56**	-1.34<br>-4.22,1.54	-0.48<br>-3.28,2.3	RIS	-0.20<br>-7.13, 6.73	**-3.83<br>-7.45, -0.21**	-4.30<br>-9.52, 0.92	NA	**-17.80<br>-28.65, -6.95**
**-7.8<br>-14.55,-0.81**	-7.06<br>-14.34,0.28	-7.04<br>-14.79,0.84	-6.03<br>-13.12,1.16	-5.6<br>-12,0.94	-4.39<br>-12.06,3.25	-3.91<br>-10.59,2.89	-2.17<br>-9,4.7	-1.31<br>-8.08,5.65	-0.82<br>-7.22,5.61	ASE	NA	NA	NA	NA
**-9.14<br>-12.45,-5.76**	**-8.39<br>-12.42,-4.33**	**-8.38<br>-13.61,-3.18**	**-7.37<br>-11.22,-3.56**	**-6.93<br>-9.36,-4.49**	**-5.73<br>-10.35,-0.97**	**-5.25<br>-8.05,-2.41**	**-3.5<br>-6.78,-0.05**	-2.64<br>-5.83,0.6	-2.16<br>-4.9,0.66	-1.34<br>-8.16,5.53	ILO	1.10<br>-3.41, 5.61	NA	NA
**-11.91<br>-15.11,-8.8**	**-11.16<br>-15.26,-7.26**	**-11.15<br>-16.33,-6.12**	**-10.13<br>-13.73,-6.6**	**-9.7<br>-12.04,-7.43**	**-8.5<br>-13.05,-3.98**	**-8.02<br>-10.36,-5.7**	**-6.27<br>-9.55,-3.08**	**-5.41<br>-8.24,-2.66**	**-4.93<br>-7.58,-2.24**	-4.11<br>-11.01,2.64	-2.77<br>-5.77,0.15	ZIP	NA	NA
**-16.3<br>-22.89,-9.61**	**-15.56<br>-22.64,-8.5**	**-15.54<br>-23.46,-7.7**	**-14.53<br>-21.51,-7.58**	**-14.1<br>-20.45,-7.71**	**-12.89<br>-19.95,-5.77**	**-12.41<br>-18.8,-6**	**-10.66<br>-17.4,-3.96**	**-9.8<br>-15.7,-3.84**	**-9.32<br>-15.85,-2.84**	-8.5<br>-17.47,0.42	**-7.16<br>-13.83,-0.57**	-4.39<br>-10.83,2.12	AMI	NA
**-26.1<br>-30.12,-22.12**	**-25.35<br>-30.01,-20.76**	**-25.34<br>-31.06,-19.64**	**-24.33<br>-28.87,-19.79**	**-23.9<br>-27.33,-20.56**	**-22.69<br>-28,-17.54**	**-22.21<br>-25.72,-18.69**	**-20.46<br>-24.62,-16.36**	**-19.6<br>-23.41,-15.74**	**-19.12<br>-22.82,-15.26**	**-18.3<br>-25.74,-11.17**	**-16.96<br>-21.08,-13.01**	**-14.19<br>-18,-10.4**	**-9.8<br>-16.91,-2.91**	SER

Table 11.10: QTc prolongation
Treatments are ranked according to their surface under the curve cumulative ranking (SUCRA) for QTc prolongation in milliseconds starting with the best. Results of the **network meta-analysis** are presented in the **left lower half** and results from **pairwise comparisons** in the **upper right** half, if available. Comparisons between treatments should be read from left to right and the estimate is in the cell in common between the column-defining treatment and the row-defining treatment. In the left lower half, mean differences (MDs) lower than 0 favor the column-defining treatment, in the upper right half MDs lower than 0 favor the row defining treatment. Cells in bold print indicate significant results. NA=not available. Drug abbreviations: AMI=Amisulpride, ARI=Aripiprazole, ASE=Asenapine, BRE=Brexpiprazole, CAR=Cariprazine, HAL=Haloperidol, ILO=Iloperidone, LUR=Lurasidone, OLA=Olanzapine, PAL=Paliperidone, PBO=Placebo, QUE=Quetiapine, RIS=Risperidone, SER=Sertindole, ZIP=Ziprasidone, ZOT=Zotepine.

Table 11.11: Sedation

PIM	NA	NA	0.33<br>0.02, 7.30	NA	NA	NA	NA	NA	NA	NA	NA	NA	NA	NA	NA	NA	NA	NA	NA	NA	NA	NA	NA	NA	NA	NA	**0.56**<br>**0.33, 0.97**	NA	NA	NA	NA	NA
0.91<br>0.17,1.97	PBO	6.00<br>0.78, 46.34	0.95<br>0.27, 3.32	0.91<br>0.49, 1.67	NA	NA	0.76<br>0.51, 1.14	0.63<br>0.33, 1.19	1.07<br>0.07, 15.59	1.09<br>0.61, 1.95	0.64<br>0.40, 1.01	NA	0.16<br>0.01, 2.91	0.64<br>0.29, 1.42	**0.45**<br>**0.30, 0.68**	**0.51**<br>**0.37, 0.70**	NA	**0.55**<br>**0.40, 0.75**	0.38<br>0.14, 1.02	**0.37**<br>**0.18, 0.76**	**0.46**<br>**0.26, 0.80**	0.69<br>0.27, 1.77	**0.40**<br>**0.30, 0.52**	**0.32**<br>**0.21, 0.49**	**0.45**<br>**0.33, 0.60**	NA	**0.24**<br>**0.15, 0.39**	NA	**0.03**<br>**0.00, 0.46**	0.36<br>0.09, 1.49	NA	NA
0.56<br>0.32,2.56	0.9<br>0.58,1.67	PERPH	NA	NA	NA	0.98<br>0.49, 1.96	NA	NA	NA	NA	NA	NA	NA	NA	NA	0.58<br>0.31, 1.06	NA	NA	NA	2.00<br>0.38, 10.44	NA	NA	NA	NA	NA	NA	NA	NA	NA	NA	NA	NA
0.58<br>0.32,3.7	0.91<br>0.48,3.33	0.92<br>0.45,4	FLU	NA	NA	NA	NA	NA	NA	NA	NA	NA	NA	NA	NA	NA	NA	NA	0.40<br>0.15, 1.07	NA	NA	NA	NA	NA	NA	NA	**0.33**<br>**0.13, 0.85**	NA	NA	NA	NA	NA
0.55<br>0.32,2.44	0.89<br>0.62,1.47	0.9<br>0.54,2.04	0.73<br>0.39,2.63	CAR	NA	NA	NA	NA	NA	NA	NA	NA	NA	NA	NA	**0.52**<br>**0.28, 0.95**	NA	NA	NA	NA	NA	NA	NA	NA	NA	NA	NA	NA	NA	NA	NA	NA
0.53<br>0.3,3.12	0.83<br>0.47,2.44	0.83<br>0.45,3.12	1.15<br>0.2,2.46	1.17<br>0.36,2.24	LEV	NA	NA	NA	NA	NA	NA	NA	NA	NA	NA	0.78<br>0.35, 1.72	NA	0.61<br>0.29, 1.26	NA	NA	NA	NA	NA	NA	NA	NA	NA	NA	NA	NA	0.14<br>0.01, 2.66	NA
0.52<br>0.3,2.44	0.81<br>0.5,1.79	0.87<br>0.53,1.82	1.02<br>0.21,2.15	1.05<br>0.39,1.91	1.14<br>0.28,2.26	PEN	NA	NA	NA	NA	NA	NA	0.94<br>0.36, 2.49	NA	NA	NA	NA	NA	NA	NA	NA	NA	NA	NA	NA	NA	0.34<br>0.08, 1.37	NA	NA	NA	NA	NA
0.48<br>0.3,1.75	**0.75**<br>**0.59,0.99**	0.75<br>0.49,1.47	0.83<br>0.2,1.75	0.84<br>0.45,1.33	0.92<br>0.27,1.78	0.79<br>0.47,1.85	PAL	NA	NA	NA	NA	NA	NA	NA	NA	NA	NA	NA	NA	NA	NA	NA	**0.52**<br>**0.38, 0.69**	NA	NA	NA	NA	NA	NA	NA	NA	NA
0.47<br>0.29,1.89	0.74<br>0.52,1.2	0.74<br>0.46,1.67	0.86<br>0.19,1.85	0.87<br>0.39,1.5	0.7<br>0.39,2.22	0.78<br>0.45,2.08	0.96<br>0.61,1.82	ILO	NA	NA	NA	NA	NA	NA	NA	NA	NA	NA	NA	NA	NA	NA	NA	**0.46**<br>**0.30, 0.70**	NA	NA	NA	NA	NA	NA	NA	NA
0.47<br>0.29,2.04	0.72<br>0.45,1.54	0.72<br>0.42,1.96	0.9<br>0.17,2.02	0.92<br>0.32,1.75	1<br>0.23,2.05	0.77<br>0.43,2.27	0.93<br>0.53,2.33	1.14<br>0.4,2.05	MOL	NA	NA	NA	1.08<br>0.45, 2.60	NA	NA	NA	NA	0.90<br>0.51, 1.58	NA	NA	NA	NA	NA	NA	NA	NA	**0.13**<br>**0.02, 0.88**	NA	NA	NA	NA	NA
0.65<br>0.11,1.59	0.73<br>0.48,1.02	0.81<br>0.37,1.41	0.8<br>0.19,1.72	0.82<br>0.41,1.34	0.89<br>0.26,1.77	0.91<br>0.35,1.67	1<br>0.61,1.47	1.02<br>0.52,1.63	1.04<br>0.41,1.85	SER	NA	NA	NA	NA	NA	1.04<br>0.39, 2.77	NA	0.67<br>0.42, 1.05	NA	NA	NA	NA	0.40<br>0.13, 1.25	NA	NA	NA	NA	NA	NA	NA	NA	NA
0.45<br>0.29,1.56	**0.68**<br>**0.54,0.9**	0.68<br>0.45,1.32	0.57<br>0.34,1.69	0.71<br>0.49,1.23	0.65<br>0.38,1.82	0.71<br>0.44,1.64	0.88<br>0.63,1.37	0.85<br>0.56,1.56	0.81<br>0.48,1.92	0.9<br>0.62,1.49	ARI	NA	NA	1.20<br>0.21, 6.98	NA	0.81<br>0.43, 1.52	NA	0.59<br>0.29, 1.20	NA	NA	NA	NA	0.29<br>0.06, 1.27	**0.51**<br>**0.29, 0.88**	NA	NA	NA	NA	NA	NA	NA	NA
0.43<br>0.28,1.64	0.64<br>0.45,1.12	0.65<br>0.41,1.49	0.54<br>0.32,1.85	0.66<br>0.43,1.41	0.61<br>0.36,1.92	0.67<br>0.4,1.79	0.81<br>0.52,1.64	0.79<br>0.49,1.82	0.76<br>0.44,2.13	0.84<br>0.53,1.72	0.9<br>0.56,1.85	AMI	NA	NA	NA	NA	0.83<br>0.58, 1.20	0.82<br>0.52, 1.28	NA	NA	NA	NA	NA	NA	NA	NA	NA	NA	NA	NA	NA	NA
0.58<br>0.09,1.49	0.65<br>0.33,1.11	0.72<br>0.28,1.35	0.72<br>0.15,1.62	0.74<br>0.31,1.39	0.8<br>0.21,1.67	0.79<br>0.31,1.48	0.91<br>0.43,1.52	0.92<br>0.39,1.65	0.9<br>0.38,1.58	0.94<br>0.45,1.58	1.01<br>0.49,1.67	1.09<br>0.44,1.91	TRIFLU	NA	NA	NA	NA	2.50<br>0.27, 22.95	NA	0.85<br>0.57, 1.26	NA	0.38<br>0.12, 1.15	NA	NA	NA	NA	NA	3.00<br>0.13, 69.39	NA	NA	NA	NA
0.42<br>0.28,1.61	0.62<br>0.43,1.1	0.62<br>0.39,1.45	0.52<br>0.32,1.79	0.65<br>0.41,1.39	0.6<br>0.35,1.92	0.65<br>0.39,1.79	0.79<br>0.49,1.61	0.77<br>0.46,1.75	0.73<br>0.42,2.17	0.81<br>0.5,1.75	0.99<br>0.46,1.64	1.08<br>0.42,1.92	0.88<br>0.51,2.44	BRE	NA	NA	NA	NA	NA	NA	NA	NA	NA	NA	0.51<br>0.16, 1.68	NA	NA	NA	NA	NA	NA	NA
0.4<br>0.28,1.23	**0.57**<br>**0.47,0.72**	0.57<br>0.4,1.03	0.58<br>0.13,1.3	**0.59**<br>**0.3,0.97**	0.65<br>0.19,1.36	0.6<br>0.39,1.28	0.72<br>0.53,1.08	0.74<br>0.38,1.21	0.67<br>0.42,1.52	0.74<br>0.53,1.15	0.82<br>0.51,1.2	0.89<br>0.42,1.48	0.81<br>0.51,1.54	0.92<br>0.43,1.57	LUR	NA	NA	0.90<br>0.50, 1.62	NA	NA	NA	NA	0.77<br>0.44, 1.37	NA	**0.40**<br>**0.19, 0.81**	NA	NA	NA	NA	NA	NA	NA
0.44<br>0.07,1.15	**0.49**<br>**0.4,0.59**	**0.55**<br>**0.28,0.93**	0.55<br>0.13,1.23	**0.56**<br>**0.31,0.88**	0.61<br>0.19,1.26	0.62<br>0.26,1.16	**0.69**<br>**0.47,0.95**	0.7<br>0.39,1.11	0.73<br>0.3,1.34	0.71<br>0.54,1.02	0.77<br>0.54,1.06	0.84<br>0.43,1.36	0.78<br>0.52,1.43	0.87<br>0.43,1.46	0.97<br>0.69,1.29	RIS	NA	0.89<br>0.74, 1.07	NA	NA	0.82<br>0.36, 1.85	NA	0.92<br>0.69, 1.23	0.72<br>0.46, 1.13	0.75<br>0.55, 1.02	NA	NA	NA	NA	NA	0.83<br>0.22, 3.16	0.80<br>0.63, 1.03
0.36<br>0.26,1.45	0.51<br>0.35,1.15	0.51<br>0.33,1.47	0.44<br>0.29,1.69	0.62<br>0.18,1.36	0.49<br>0.31,1.82	0.53<br>0.33,1.75	0.63<br>0.39,1.64	0.61<br>0.37,1.82	0.59<br>0.35,2.04	0.65<br>0.39,1.75	0.85<br>0.28,1.71	0.84<br>0.4,1.42	0.7<br>0.39,2.27	0.96<br>0.26,1.96	0.84<br>0.48,2.44	0.88<br>0.5,2.44	FPX	NA	NA	NA	NA	NA	0.38<br>0.02, 8.66	NA	NA	NA	NA	NA	NA	NA	NA	NA
0.37<br>0.27,1.04	**0.52**<br>**0.46,0.59**	**0.52**<br>**0.39,0.87**	0.49<br>0.12,1.13	**0.5**<br>**0.28,0.81**	0.51<br>0.33,1.16	0.54<br>0.38,1.06	**0.65**<br>**0.52,0.87**	0.63<br>0.46,1.01	0.6<br>0.4,1.23	**0.66**<br>**0.52,0.93**	**0.7**<br>**0.49,0.97**	0.76<br>0.4,1.23	0.72<br>0.49,1.27	0.8<br>0.38,1.36	0.87<br>0.67,1.19	0.91<br>0.78,1.09	1.03<br>0.38,1.86	HAL	1.13<br>0.80, 1.57	1.67<br>0.23, 11.87	0.59<br>0.19, 1.78	NA	0.89<br>0.72, 1.10	1.02<br>0.67, 1.54	**0.54**<br>**0.37, 0.79**	NA	0.43<br>0.14, 1.34	0.32<br>0.01, 7.50	0.54<br>0.26, 1.16	0.54<br>0.16, 1.75	0.60<br>0.33, 1.09	**0.32**<br>**0.16, 0.67**
**0.33**<br>**0.05,0.9**	**0.38**<br>**0.22,0.62**	**0.43**<br>**0.17,0.83**	**0.42**<br>**0.09,0.98**	**0.44**<br>**0.19,0.8**	0.48<br>0.12,1.11	0.49<br>0.17,1	**0.55**<br>**0.29,0.92**	0.56<br>0.24,1.01	0.57<br>0.2,1.14	**0.57**<br>**0.28,0.97**	0.62<br>0.32,1.01	0.67<br>0.27,1.22	0.62<br>0.42,1.19	0.7<br>0.27,1.29	0.78<br>0.42,1.24	0.81<br>0.47,1.21	0.91<br>0.28,1.78	0.89<br>0.54,1.3	THIOR	1.01<br>0.66, 1.55	NA	9.00<br>0.51,159.77	NA	NA	NA	NA	0.89<br>0.73, 1.09	NA	NA	NA	NA	NA
**0.34**<br>**0.26,0.91**	**0.46**<br>**0.37,0.61**	**0.46**<br>**0.35,0.79**	**0.41**<br>**0.09,0.98**	**0.42**<br>**0.2,0.78**	0.47<br>0.12,1.06	**0.48**<br>**0.34,0.93**	**0.56**<br>**0.42,0.86**	**0.54**<br>**0.25,0.97**	0.53<br>0.36,1.05	**0.57**<br>**0.42,0.9**	**0.6**<br>**0.33,0.95**	0.65<br>0.29,1.16	0.63<br>0.45,1.02	0.68<br>0.29,1.25	0.73<br>0.52,1.18	0.76<br>0.56,1.15	0.89<br>0.28,1.75	0.86<br>0.55,1.23	0.94<br>0.65,1.61	LOX	NA	1.18<br>0.56, 2.48	NA	NA	NA	NA	0.80<br>0.50, 1.27	NA	NA	NA	NA	NA
**0.34**<br>**0.26,0.94**	**0.46**<br>**0.37,0.62**	**0.46**<br>**0.34,0.83**	0.4<br>0.28,1.02	**0.47**<br>**0.35,0.78**	0.44<br>0.31,1.06	0.47<br>0.33,1	**0.56**<br>**0.41,0.87**	**0.54**<br>**0.39,0.97**	0.52<br>0.35,1.14	**0.57**<br>**0.42,0.92**	**0.6**<br>**0.33,0.96**	0.65<br>0.28,1.18	0.61<br>0.41,1.2	0.62<br>0.41,1.28	0.73<br>0.52,1.19	0.76<br>0.56,1.15	0.7<br>0.41,2	0.83<br>0.6,1.27	0.93<br>0.59,1.82	0.95<br>0.62,1.75	ASE	NA	0.56<br>0.19, 1.60	NA	NA	NA	NA	NA	NA	NA	NA	NA
**0.32**<br>**0.05,0.92**	**0.37**<br>**0.19,0.62**	**0.42**<br>**0.15,0.83**	0.41<br>0.08,1.01	**0.42**<br>**0.17,0.82**	0.46<br>0.11,1.1	0.47<br>0.15,1	**0.53**<br>**0.25,0.92**	0.54<br>0.22,1.02	0.54<br>0.19,1.11	**0.55**<br>**0.25,0.97**	0.6<br>0.28,1.03	0.65<br>0.25,1.23	0.61<br>0.41,1.14	0.68<br>0.24,1.3	0.76<br>0.38,1.25	0.79<br>0.42,1.24	0.89<br>0.25,1.79	0.86<br>0.47,1.33	1.02<br>0.52,1.64	1.03<br>0.55,1.58	1.04<br>0.49,1.72	THIOTH	NA	NA	NA	NA	1.03<br>0.68, 1.55	NA	0.63<br>0.25, 1.60	NA	NA	NA
**0.34**<br>**0.26,0.88**	**0.46**<br>**0.41,0.52**	**0.46**<br>**0.35,0.74**	**0.4**<br>**0.09,0.96**	**0.41**<br>**0.22,0.66**	**0.45**<br>**0.13,0.98**	**0.48**<br>**0.34,0.9**	**0.56**<br>**0.47,0.71**	**0.52**<br>**0.28,0.84**	0.54<br>0.22,1.05	**0.57**<br>**0.45,0.79**	**0.58**<br>**0.39,0.81**	0.63<br>0.31,1.06	0.62<br>0.43,1.08	0.66<br>0.32,1.15	0.74<br>0.52,1	**0.78**<br>**0.65,0.93**	0.87<br>0.3,1.63	0.84<br>0.68,1	0.94<br>0.65,1.54	1.02<br>0.65,1.44	1.02<br>0.64,1.44	0.97<br>0.63,1.79	OLA	0.95<br>0.71, 1.28	1.09<br>0.69, 1.71	NA	NA	NA	NA	NA	1.50<br>0.29, 7.77	NA
**0.3**<br>**0.05,0.85**	**0.34**<br>**0.26,0.44**	**0.39**<br>**0.18,0.69**	**0.39**<br>**0.09,0.92**	**0.39**<br>**0.21,0.65**	**0.43**<br>**0.12,0.95**	**0.44**<br>**0.17,0.87**	**0.49**<br>**0.32,0.71**	**0.5**<br>**0.28,0.78**	0.52<br>0.2,1.03	**0.51**<br>**0.31,0.77**	**0.55**<br>**0.37,0.78**	0.61<br>0.29,1.04	0.61<br>0.29,1.06	0.63<br>0.29,1.13	**0.71**<br>**0.47,0.99**	**0.73**<br>**0.56,0.93**	0.83<br>0.28,1.59	0.81<br>0.62,1.02	0.97<br>0.56,1.44	0.98<br>0.61,1.42	0.98<br>0.6,1.42	1.02<br>0.55,1.61	0.97<br>0.76,1.2	ZIP	NA	NA	NA	NA	NA	NA	NA	NA
**0.27**<br>**0.04,0.75**	**0.31**<br>**0.24,0.38**	**0.35**<br>**0.17,0.62**	**0.34**<br>**0.08,0.82**	**0.35**<br>**0.18,0.57**	**0.39**<br>**0.11,0.86**	**0.39**<br>**0.15,0.78**	**0.44**<br>**0.29,0.63**	**0.45**<br>**0.23,0.74**	**0.46**<br>**0.18,0.92**	**0.52**<br>**0.41,0.71**	**0.5**<br>**0.32,0.72**	**0.54**<br>**0.26,0.93**	**0.56**<br>**0.4,0.93**	**0.56**<br>**0.27,0.99**	**0.63**<br>**0.43,0.87**	**0.68**<br>**0.57,0.83**	0.75<br>0.25,1.46	**0.73**<br>**0.57,0.9**	0.83<br>0.57,1.33	0.88<br>0.56,1.27	0.89<br>0.54,1.3	0.85<br>0.56,1.52	0.88<br>0.68,1.09	0.9<br>0.7,1.19	QUE	NA	1.29<br>0.73, 2.29	NA	NA	NA	NA	NA
**0.28**<br>**0.24,0.96**	**0.33**<br>**0.26,0.83**	**0.34**<br>**0.26,0.99**	0.32<br>0.03,1.05	0.33<br>0.05,1	0.37<br>0.04,1.15	0.37<br>0.05,1.12	0.42<br>0.07,1.16	0.43<br>0.07,1.23	0.44<br>0.06,1.31	0.38<br>0.28,1.23	0.47<br>0.08,1.3	0.51<br>0.08,1.41	0.41<br>0.28,1.49	0.54<br>0.08,1.47	0.61<br>0.11,1.55	0.47<br>0.31,1.67	0.71<br>0.09,1.88	0.69<br>0.14,1.68	0.56<br>0.33,2.38	0.84<br>0.16,1.97	0.85<br>0.15,2	0.57<br>0.33,2.38	0.84<br>0.17,1.92	0.59<br>0.34,2.44	0.64<br>0.36,2.7	PERA	NA	NA	0.87<br>0.39, 1.97	NA	NA	NA
**0.31**<br>**0.26,0.67**	**0.39**<br>**0.34,0.46**	**0.4**<br>**0.31,0.6**	**0.35**<br>**0.27,0.72**	**0.3**<br>**0.15,0.53**	**0.38**<br>**0.29,0.78**	**0.41**<br>**0.31,0.7**	**0.46**<br>**0.38,0.62**	**0.45**<br>**0.35,0.7**	**0.44**<br>**0.33,0.79**	**0.47**<br>**0.37,0.67**	**0.43**<br>**0.26,0.66**	**0.47**<br>**0.21,0.85**	**0.51**<br>**0.38,0.82**	**0.49**<br>**0.21,0.9**	**0.58**<br>**0.45,0.83**	**0.61**<br>**0.49,0.79**	0.57<br>0.36,1.37	**0.66**<br>**0.53,0.85**	0.74<br>0.54,1.11	0.76<br>0.57,1.08	0.78<br>0.44,1.2	0.76<br>0.53,1.25	0.76<br>0.6,1.03	0.79<br>0.6,1.11	0.87<br>0.68,1.18	0.92<br>0.44,4.17	CPZ	0.84<br>0.52, 1.36	0.94<br>0.53, 1.67	0.78<br>0.50, 1.21	**0.74**<br>**0.60, 0.92**	NA
**0.21**<br>**0.03,0.66**	**0.24**<br>**0.09,0.49**	**0.27**<br>**0.08,0.63**	**0.27**<br>**0.05,0.75**	**0.28**<br>**0.09,0.62**	**0.3**<br>**0.06,0.8**	**0.31**<br>**0.08,0.73**	**0.35**<br>**0.13,0.71**	**0.36**<br>**0.12,0.79**	**0.36**<br>**0.1,0.83**	**0.37**<br>**0.13,0.76**	**0.4**<br>**0.15,0.81**	**0.44**<br>**0.13,0.95**	**0.45**<br>**0.32,0.92**	**0.45**<br>**0.14,0.98**	0.51<br>0.2,1	0.53<br>0.22,1	0.61<br>0.14,1.44	0.59<br>0.24,1.08	0.63<br>0.41,1.35	0.71<br>0.29,1.3	0.73<br>0.26,1.38	0.65<br>0.41,1.52	0.72<br>0.3,1.3	0.67<br>0.43,1.41	0.83<br>0.36,1.46	1.31<br>0.23,2.67	0.93<br>0.44,1.53	SUL	NA	NA	NA	NA
**0.16**<br>**0.02,0.5**	**0.19**<br>**0.1,0.32**	**0.21**<br>**0.08,0.44**	**0.21**<br>**0.04,0.54**	**0.21**<br>**0.08,0.42**	**0.24**<br>**0.05,0.61**	**0.24**<br>**0.07,0.54**	**0.27**<br>**0.13,0.48**	**0.28**<br>**0.11,0.55**	**0.28**<br>**0.09,0.63**	**0.28**<br>**0.13,0.52**	**0.31**<br>**0.15,0.56**	**0.34**<br>**0.13,0.69**	**0.34**<br>**0.13,0.67**	**0.35**<br>**0.13,0.74**	**0.4**<br>**0.19,0.69**	**0.42**<br>**0.21,0.69**	0.48<br>0.13,1.08	**0.46**<br>**0.24,0.75**	**0.56**<br>**0.26,0.98**	**0.57**<br>**0.28,0.98**	0.57<br>0.26,1.03	0.58<br>0.28,1.02	**0.56**<br>**0.3,0.91**	**0.59**<br>**0.31,0.98**	0.66<br>0.35,1.06	1.01<br>0.25,2.07	0.75<br>0.43,1.17	0.92<br>0.36,1.71	ZOT	NA	NA	NA
**0.27**<br>**0.24,0.58**	**0.32**<br>**0.27,0.49**	**0.32**<br>**0.26,0.56**	**0.3**<br>**0.25,0.65**	**0.2**<br>**0.06,0.49**	**0.31**<br>**0.26,0.69**	**0.32**<br>**0.26,0.64**	**0.35**<br>**0.28,0.63**	**0.35**<br>**0.28,0.67**	**0.34**<br>**0.27,0.73**	**0.36**<br>**0.28,0.66**	**0.29**<br>**0.09,0.65**	**0.32**<br>**0.08,0.77**	**0.38**<br>**0.28,0.79**	**0.34**<br>**0.09,0.83**	**0.42**<br>**0.31,0.84**	**0.43**<br>**0.32,0.85**	0.41<br>0.29,1.18	**0.46**<br>**0.33,0.91**	0.5<br>0.34,1.16	0.51<br>0.34,1.14	0.55<br>0.17,1.16	0.51<br>0.34,1.23	0.52<br>0.35,1.09	0.53<br>0.36,1.16	0.57<br>0.37,1.27	0.59<br>0.32,3.33	0.72<br>0.27,1.36	0.67<br>0.39,2.13	0.81<br>0.45,2.33	CPX	NA	NA
**0.28**<br>**0.25,0.53**	**0.33**<br>**0.3,0.4**	**0.33**<br>**0.28,0.49**	**0.31**<br>**0.26,0.56**	**0.2**<br>**0.09,0.38**	**0.33**<br>**0.27,0.59**	**0.34**<br>**0.28,0.56**	**0.38**<br>**0.32,0.51**	**0.37**<br>**0.3,0.56**	**0.36**<br>**0.29,0.61**	**0.38**<br>**0.32,0.53**	**0.29**<br>**0.16,0.49**	**0.32**<br>**0.13,0.61**	**0.4**<br>**0.32,0.64**	**0.33**<br>**0.13,0.66**	**0.45**<br>**0.36,0.65**	**0.47**<br>**0.38,0.63**	0.44<br>0.31,1	**0.5**<br>**0.4,0.67**	**0.55**<br>**0.41,0.88**	**0.56**<br>**0.42,0.85**	**0.54**<br>**0.28,0.91**	**0.56**<br>**0.41,0.96**	**0.57**<br>**0.44,0.8**	**0.58**<br>**0.45,0.85**	**0.63**<br>**0.48,0.92**	0.66<br>0.36,2.94	**0.71**<br>**0.49,0.98**	0.76<br>0.48,1.64	0.92<br>0.59,1.72	1.18<br>0.46,2.07	CLO	NA
**0.09**<br>**0.01,0.3**	**0.1**<br>**0.03,0.21**	**0.11**<br>**0.03,0.28**	**0.11**<br>**0.02,0.35**	**0.11**<br>**0.03,0.27**	**0.13**<br>**0.02,0.38**	**0.13**<br>**0.03,0.35**	**0.14**<br>**0.05,0.32**	**0.15**<br>**0.04,0.36**	**0.15**<br>**0.04,0.41**	**0.15**<br>**0.05,0.34**	**0.16**<br>**0.05,0.36**	**0.18**<br>**0.05,0.43**	**0.18**<br>**0.05,0.45**	**0.19**<br>**0.05,0.47**	**0.22**<br>**0.07,0.47**	**0.22**<br>**0.08,0.46**	**0.26**<br>**0.05,0.69**	**0.25**<br>**0.09,0.51**	**0.31**<br>**0.1,0.7**	**0.32**<br>**0.1,0.7**	**0.32**<br>**0.1,0.68**	**0.33**<br>**0.1,0.76**	**0.31**<br>**0.11,0.63**	**0.33**<br>**0.12,0.68**	**0.37**<br>**0.13,0.75**	0.64<br>0.09,1.71	**0.43**<br>**0.15,0.88**	0.54<br>0.14,1.25	0.64<br>0.2,1.35	0.75<br>0.18,1.69	0.65<br>0.22,1.27	ZUC

Table 11.11: Sedation

Treatments are ranked according to their surface under the curve cumulative ranking (SUCRA) for sedation starting with the best. Results of the **network meta-analysis** are presented in the **left lower half** and results from **pairwise comparisons** in the **upper right half**, if available. Comparisons between treatments should be read from left to right and the estimate is in the cell in common between the column-defining treatment and the row-defining treatment. Risk ratios (RRs) lower than 1 favor the column-defining treatment. Cells in bold print indicate significant results.

NA=not available. Drug abbreviations: AMI=Amisulpride, ARI=Aripiprazole, ASE=Asenapine, BRE=Brexpiprazole, CAR=Cariprazine, CPZ=Chlorpromazine, CPX=Clopenthixol, CLO=Clozapine, FPX=Flupenthixol, FLU=Fluphenazine, HAL=Haloperidol, ILO=Iloperidone, LEV=Levomepromazine, LOX=Loxapine, LUR=Lurasidone, MOL=Molindone, OLA=Olanzapine, PAL=Paliperidone, PEN=Penfluridol, PERA=Perazine, PERPH=Perphenazine, PIM=Pimozide, PBO=Placebo, QUE=Quetiapine, RIS=Risperidone, SER=Sertindole, SUL=Sulpiride, THIOR=Thioridazine, THIOTH=Thiothixene, TRIFLU=Trifluoperazine, ZIP=Ziprasidone, ZOT=Zotepine, ZUC=Zuclopenthixol.

Table 11.12: Anticholinergic side effects

BRE	NA	0.69<br>0.35, 1.38	NA	NA	NA	NA	NA	NA	1.11<br>0.10, 12.15	NA	NA	NA	NA	NA	NA	NA	NA	NA	NA	NA	NA	NA	NA	NA	NA	NA	NA	NA	NA	NA	NA	**0.16**<br>**0.04, 0.71**
1.15<br>0.22,3.13	MOL	NA	NA	NA	NA	NA	NA	NA	NA	NA	NA	NA	NA	NA	NA	NA	NA	NA	0.83<br>0.39, 1.80	0.50<br>0.21, 1.17	NA	NA	NA	NA	NA	NA	NA	NA	NA	NA	NA	NA
0.72<br>0.32,1.34	0.9<br>0.24,2.23	PBO	0.94<br>0.52, 1.72	1.07<br>0.66, 1.74	NA	NA	NA	1.25<br>0.37, 4.23	0.60<br>0.34, 1.08	0.77<br>0.56, 1.06	0.77<br>0.45, 1.30	0.33<br>0.04, 3.07	0.64<br>0.38, 1.08	0.98<br>0.60, 1.62	0.62<br>0.33, 1.16	NA	NA	0.19<br>0.01, 3.79	NA	0.71<br>0.47, 1.09	NA	NA	**0.62**<br>**0.42, 0.92**	0.51<br>0.15, 1.80	NA	**0.08**<br>**0.01, 0.57**	NA	**0.39**<br>**0.19, 0.78**	0.50<br>0.05, 5.44	NA	0.08<br>0.00, 1.36	**0.42**<br>**0.28, 0.63**
0.58<br>0.28,1.69	0.58<br>0.24,2.7	0.91<br>0.52,1.89	ASE	NA	NA	NA	NA	NA	NA	1.50<br>0.43, 5.21	NA	NA	NA	NA	NA	NA	NA	NA	NA	NA	NA	NA	**0.16**<br>**0.03, 0.83**	NA	NA	NA	NA	NA	NA	NA	NA	NA
0.68<br>0.26,1.4	0.57<br>0.24,2.33	0.88<br>0.58,1.45	1.02<br>0.43,2	LUR	NA	NA	NA	NA	NA	NA	NA	NA	NA	NA	NA	NA	NA	NA	NA	1.22<br>0.31, 4.70	NA	NA	0.64<br>0.30, 1.40	NA	NA	NA	NA	NA	NA	NA	NA	**0.22**<br>**0.07, 0.71**
0.78<br>0.19,2.02	0.96<br>0.16,2.85	0.85<br>0.4,2.44	1.18<br>0.3,2.83	1.21<br>0.37,2.72	PIM	NA	NA	NA	NA	NA	NA	NA	NA	NA	NA	NA	NA	NA	NA	1.03<br>0.57, 1.87	NA	NA	NA	NA	NA	NA	NA	**0.30**<br>**0.09, 0.96**	NA	NA	NA	NA
0.8<br>0.17,2.25	0.53<br>0.2,3.33	0.81<br>0.36,2.86	1.21<br>0.28,3.16	0.86<br>0.36,3.45	0.76<br>0.29,4.17	LEV	NA	NA	NA	1.29<br>0.56, 2.95	NA	NA	NA	NA	NA	NA	NA	NA	NA	0.86<br>0.42, 1.75	NA	NA	NA	NA	0.14<br>0.01, 2.69	NA	NA	NA	NA	NA	NA	NA
0.72<br>0.18,1.82	0.87<br>0.16,2.56	0.99<br>0.36,2.08	1.08<br>0.3,2.62	1.11<br>0.35,2.46	1.15<br>0.25,2.96	1.21<br>0.23,3.3	SUL	1.00<br>0.15, 6.81	NA	NA	NA	NA	NA	NA	NA	NA	NA	NA	0.33<br>0.01, 7.78	0.71<br>0.21, 2.36	NA	NA	NA	NA	NA	NA	NA	0.67<br>0.27, 1.66	NA	NA	NA	NA
0.63<br>0.18,1.51	0.78<br>0.15,2.27	0.76<br>0.4,1.72	0.96<br>0.3,2.15	0.99<br>0.36,2.05	0.71<br>0.29,2.78	1.08<br>0.23,2.82	0.79<br>0.35,2.63	PERPH	NA	0.82<br>0.32, 2.13	NA	NA	NA	NA	NA	1.38<br>0.63, 3.01	NA	NA	NA	NA	NA	NA	NA	NA	NA	NA	0.38<br>0.11, 1.29	NA	NA	NA	NA	NA
0.5<br>0.27,1.19	0.5<br>0.22,2	0.78<br>0.53,1.22	0.76<br>0.4,1.79	0.83<br>0.48,1.67	0.72<br>0.33,2.33	0.7<br>0.3,2.63	0.79<br>0.36,2.44	0.88<br>0.43,2.33	ARI	0.82<br>0.43, 1.57	1.38<br>0.61, 3.14	NA	NA	NA	NA	NA	NA	NA	NA	**0.19**<br>**0.05, 0.73**	NA	NA	0.50<br>0.05, 5.31	NA	NA	NA	NA	NA	NA	NA	NA	NA
0.55<br>0.23,1.07	0.68<br>0.17,1.73	**0.76**<br>**0.58,0.96**	0.83<br>0.39,1.5	0.86<br>0.49,1.37	0.9<br>0.3,1.98	0.94<br>0.26,2.21	0.77<br>0.37,2.13	1.01<br>0.44,1.92	0.99<br>0.61,1.49	RIS	1.26<br>0.73, 2.17	NA	1.36<br>0.69, 2.68	NA	0.94<br>0.32, 2.80	NA	0.42<br>0.13, 1.37	NA	NA	0.89<br>0.65, 1.23	NA	NA	**0.50**<br>**0.29, 0.88**	NA	1.00<br>0.20, 5.12	NA	NA	NA	NA	NA	NA	**0.42**<br>**0.23, 0.77**
0.54<br>0.21,1.09	0.66<br>0.17,1.74	0.75<br>0.51,1.04	0.82<br>0.35,1.54	0.85<br>0.45,1.41	0.88<br>0.28,1.96	0.92<br>0.25,2.23	0.94<br>0.32,2.05	0.99<br>0.39,1.97	0.96<br>0.56,1.5	0.99<br>0.68,1.38	ZIP	NA	NA	NA	NA	NA	NA	NA	NA	0.94<br>0.53, 1.67	NA	NA	0.97<br>0.46, 2.05	NA	NA	0.85<br>0.41, 1.77	NA	NA	NA	NA	NA	NA
0.56<br>0.16,1.34	0.67<br>0.15,1.85	0.78<br>0.33,1.49	0.86<br>0.27,1.93	0.89<br>0.33,1.84	0.91<br>0.23,2.24	0.96<br>0.2,2.48	0.97<br>0.26,2.36	1.03<br>0.32,2.37	1.02<br>0.38,2.07	1.04<br>0.44,1.98	0.9<br>0.46,2.22	THIOTH	NA	NA	NA	NA	NA	NA	0.71<br>0.28, 1.83	NA	NA	NA	NA	2.79<br>0.30, 25.57	NA	NA	NA	0.64<br>0.37, 1.11	0.66<br>0.27, 1.63	NA	0.20<br>0.01, 4.03	NA
0.53<br>0.19,1.15	0.45<br>0.2,1.85	0.69<br>0.44,1.19	0.81<br>0.31,1.67	0.74<br>0.41,1.56	0.65<br>0.29,2.22	0.64<br>0.26,2.44	0.7<br>0.32,2.33	0.79<br>0.38,2.22	0.97<br>0.46,1.71	0.91<br>0.56,1.64	0.93<br>0.54,1.82	0.88<br>0.43,2.5	CAR	NA	NA	NA	NA	NA	NA	NA	NA	NA	NA	NA	NA	NA	NA	NA	NA	NA	NA	NA
0.53<br>0.2,1.13	0.66<br>0.15,1.8	0.69<br>0.45,1.18	0.81<br>0.32,1.67	0.84<br>0.39,1.52	0.65<br>0.29,2.22	0.91<br>0.22,2.29	0.7<br>0.32,2.27	0.79<br>0.38,2.22	0.96<br>0.47,1.69	0.91<br>0.56,1.64	0.93<br>0.55,1.79	0.88<br>0.43,2.5	1.07<br>0.48,1.98	PAL	NA	NA	NA	NA	NA	NA	NA	NA	0.88<br>0.48, 1.62	NA	NA	NA	NA	NA	NA	NA	NA	NA
0.53<br>0.19,1.15	0.66<br>0.15,1.77	0.74<br>0.41,1.18	0.81<br>0.32,1.65	0.84<br>0.39,1.53	0.87<br>0.25,2.05	0.91<br>0.22,2.24	0.7<br>0.33,2.22	0.98<br>0.35,2.11	0.96<br>0.46,1.69	0.98<br>0.54,1.6	0.93<br>0.55,1.75	0.88<br>0.43,2.44	1.07<br>0.48,2.01	1.07<br>0.49,1.95	SER	NA	NA	NA	NA	0.98<br>0.52, 1.84	NA	NA	0.57<br>0.27, 1.24	NA	NA	NA	NA	NA	NA	NA	NA	NA
0.59<br>0.12,1.64	0.74<br>0.11,2.36	0.62<br>0.28,2	0.9<br>0.2,2.35	0.93<br>0.24,2.32	0.58<br>0.23,2.78	1.01<br>0.16,2.94	0.65<br>0.26,2.94	0.78<br>0.37,2.13	1.07<br>0.28,2.6	0.81<br>0.35,2.7	0.82<br>0.35,2.94	0.8<br>0.32,3.33	1.19<br>0.3,2.91	1.19<br>0.3,2.88	0.83<br>0.34,3.33	PEN	NA	NA	NA	NA	NA	NA	NA	NA	NA	NA	NA	1.36<br>0.53, 3.47	NA	NA	NA	NA
0.43<br>0.22,1.2	0.44<br>0.19,1.85	0.66<br>0.38,1.32	0.65<br>0.31,1.79	0.7<br>0.37,1.64	0.62<br>0.27,2.13	0.61<br>0.25,2.5	0.68<br>0.3,2.27	0.75<br>0.35,2.27	0.81<br>0.43,1.82	0.86<br>0.5,1.72	0.88<br>0.48,1.92	0.84<br>0.39,2.5	0.88<br>0.44,2.22	0.88<br>0.44,2.22	0.89<br>0.45,2.17	0.8<br>0.32,3.33	AMI	NA	NA	0.66<br>0.34, 1.27	0.68<br>0.34, 1.36	NA	NA	NA	NA	NA	NA	NA	NA	NA	NA	NA
0.85<br>0.07,3.33	0.32<br>0.12,5	0.48<br>0.18,5	1.25<br>0.12,4.52	0.51<br>0.18,5.88	0.45<br>0.16,6.25	0.44<br>0.15,6.67	0.49<br>0.17,7.14	0.55<br>0.19,6.67	1.49<br>0.15,5.21	0.62<br>0.22,6.67	0.63<br>0.22,7.14	0.62<br>0.21,7.69	1.64<br>0.16,5.39	0.64<br>0.22,7.69	0.64<br>0.21,7.69	0.58<br>0.19,9.09	1.73<br>0.16,5.74	FLU	NA	NA	NA	NA	NA	NA	NA	NA	NA	0.82<br>0.18, 3.81	NA	NA	0.42<br>0.11, 1.64	NA
0.53<br>0.13,1.4	0.57<br>0.17,1.35	0.74<br>0.26,1.6	0.81<br>0.21,2.01	0.84<br>0.26,1.92	0.86<br>0.18,2.3	0.91<br>0.17,2.6	0.92<br>0.21,2.39	0.97<br>0.24,2.4	0.96<br>0.3,2.17	0.98<br>0.34,2.09	0.8<br>0.38,2.33	1.04<br>0.34,2.33	1.07<br>0.32,2.46	1.07<br>0.33,2.42	1.07<br>0.33,2.45	1.2<br>0.23,3.25	1.12<br>0.32,2.64	1.56<br>0.11,5.03	TRIFLU	2.50<br>0.27, 23.26	NA	NA	NA	0.65<br>0.28, 1.47	NA	NA	NA	NA	NA	NA	NA	NA
**0.48**<br>**0.2,0.95**	0.44<br>0.21,1.54	**0.67**<br>**0.52,0.88**	0.74<br>0.34,1.36	0.71<br>0.46,1.22	0.63<br>0.31,1.72	0.62<br>0.28,2	0.68<br>0.34,1.85	0.76<br>0.4,1.79	0.87<br>0.52,1.35	0.88<br>0.67,1.18	0.89<br>0.63,1.32	0.85<br>0.46,1.96	0.98<br>0.52,1.64	0.9<br>0.55,1.61	0.9<br>0.56,1.61	0.81<br>0.36,2.78	1.02<br>0.52,1.78	1.43<br>0.13,4.21	0.9<br>0.43,2.44	HAL	NA	NA	**0.57**<br>**0.37, 0.88**	1.00<br>0.31, 3.19	1.05<br>0.49, 2.24	NA	NA	0.71<br>0.34, 1.49	0.47<br>0.20, 1.11	0.48<br>0.14, 1.71	0.96<br>0.59, 1.56	**0.32**<br>**0.15, 0.68**
0.45<br>0.09,1.29	0.32<br>0.14,1.96	0.47<br>0.22,1.54	0.68<br>0.15,1.87	0.5<br>0.22,1.89	0.44<br>0.18,2.27	0.43<br>0.17,2.5	0.48<br>0.2,2.38	0.53<br>0.22,2.44	0.81<br>0.21,2.03	0.61<br>0.27,2.08	0.62<br>0.27,2.22	0.6<br>0.24,2.7	0.91<br>0.21,2.34	0.62<br>0.27,2.5	0.63<br>0.27,2.44	0.56<br>0.22,3.45	0.87<br>0.3,1.88	0.41<br>0.14,5.88	0.62<br>0.24,3.33	0.68<br>0.3,2.33	FPX	NA	0.23<br>0.01, 4.43	NA	NA	NA	NA	NA	NA	NA	NA	NA
0.58<br>0.02,2.73	0.73<br>0.02,3.28	0.21<br>0.1,4.17	0.9<br>0.03,3.79	0.92<br>0.03,4	0.2<br>0.09,5.26	1.02<br>0.03,4.29	0.21<br>0.1,5.56	0.23<br>0.1,5.56	1.07<br>0.04,4.37	0.25<br>0.1,5.56	0.26<br>0.1,5.88	0.26<br>0.11,5.88	1.18<br>0.04,4.69	1.17<br>0.04,4.77	0.26<br>0.11,6.25	1.31<br>0.04,5.19	1.23<br>0.04,4.94	1.76<br>0.02,6.65	0.26<br>0.11,7.14	1.21<br>0.05,4.84	1.75<br>0.05,6.36	PERA	NA	NA	NA	NA	NA	NA	1.00<br>0.15, 6.55	NA	NA	NA
**0.36**<br>**0.15,0.73**	0.45<br>0.11,1.2	**0.52**<br>**0.41,0.68**	0.56<br>0.25,1.04	**0.58**<br>**0.33,0.93**	0.49<br>0.24,1.37	0.63<br>0.17,1.54	0.52<br>0.27,1.45	0.58<br>0.32,1.39	0.66<br>0.39,1.05	**0.67**<br>**0.51,0.93**	0.68<br>0.49,1.03	0.65<br>0.35,1.54	0.74<br>0.4,1.26	0.69<br>0.45,1.18	0.69<br>0.44,1.22	0.62<br>0.28,2.13	0.78<br>0.37,1.44	1.11<br>0.09,3.47	0.68<br>0.33,1.96	0.77<br>0.55,1.04	1.12<br>0.32,2.66	0.62<br>0.17,16.67	OLA	NA	0.33<br>0.04, 2.90	NA	NA	NA	NA	NA	NA	**0.29**<br>**0.13, 0.66**
**0.36**<br>**0.1,0.9**	0.32<br>0.16,1.18	0.45<br>0.25,1.01	0.56<br>0.17,1.31	0.48<br>0.25,1.27	0.43<br>0.2,1.59	0.63<br>0.13,1.74	0.46<br>0.21,1.64	0.51<br>0.24,1.64	0.66<br>0.24,1.4	0.58<br>0.31,1.35	0.6<br>0.31,1.43	0.59<br>0.29,1.64	0.74<br>0.25,1.62	0.6<br>0.3,1.61	0.6<br>0.3,1.61	0.54<br>0.22,2.38	0.78<br>0.25,1.78	1.08<br>0.08,3.51	0.65<br>0.32,1.75	0.76<br>0.33,1.49	1.12<br>0.23,3.02	0.56<br>0.16,16.67	0.85<br>0.43,2.04	LOX	NA	NA	NA	0.33<br>0.01, 8.00	NA	NA	0.93<br>0.36, 2.40	NA
**0.34**<br>**0.11,0.77**	0.31<br>0.15,1.18	**0.46**<br>**0.29,0.82**	0.53<br>0.19,1.12	0.49<br>0.28,1.04	0.44<br>0.21,1.33	0.43<br>0.2,1.52	0.47<br>0.23,1.43	0.52<br>0.27,1.41	0.63<br>0.28,1.16	0.6<br>0.36,1.09	0.6<br>0.36,1.18	0.59<br>0.3,1.47	0.7<br>0.3,1.35	0.61<br>0.33,1.35	0.61<br>0.34,1.33	0.56<br>0.25,2.08	0.74<br>0.28,1.49	0.41<br>0.14,3.7	0.61<br>0.29,1.92	0.67<br>0.41,1.2	0.71<br>0.29,2.94	0.56<br>0.16,16.67	0.87<br>0.51,1.67	0.88<br>0.43,2.44	CLO	NA	NA	0.90<br>0.59, 1.36	NA	NA	NA	NA
**0.29**<br>**0.07,0.76**	0.25<br>0.12,1.14	**0.36**<br>**0.19,0.86**	0.44<br>0.12,1.09	0.38<br>0.19,1.06	0.34<br>0.16,1.37	0.33<br>0.15,1.49	0.36<br>0.17,1.41	0.4<br>0.19,1.41	0.53<br>0.17,1.19	0.46<br>0.23,1.16	0.47<br>0.24,1.16	0.44<br>0.2,1.61	0.59<br>0.18,1.37	0.47<br>0.23,1.35	0.47<br>0.23,1.37	0.43<br>0.19,2.04	0.63<br>0.17,1.53	0.89<br>0.06,3.18	0.47<br>0.2,1.92	0.62<br>0.21,1.34	0.9<br>0.16,2.55	0.43<br>0.14,14.29	0.66<br>0.32,1.75	0.66<br>0.28,2.44	0.93<br>0.27,2.12	ILO	NA	NA	NA	NA	NA	NA
**0.28**<br>**0.05,0.83**	0.22<br>0.11,1.2	0.31<br>0.15,1.01	0.43<br>0.08,1.21	0.33<br>0.16,1.19	0.29<br>0.14,1.43	0.28<br>0.13,1.61	0.32<br>0.14,1.49	0.37<br>0.18,1.22	0.51<br>0.12,1.34	0.39<br>0.19,1.35	0.4<br>0.18,1.47	0.38<br>0.17,1.67	0.57<br>0.13,1.53	0.4<br>0.18,1.61	0.4<br>0.18,1.56	0.38<br>0.17,1.85	0.6<br>0.12,1.66	0.28<br>0.11,3.45	0.4<br>0.17,2.08	0.43<br>0.2,1.54	0.47<br>0.18,3.03	0.38<br>0.12,12.5	0.56<br>0.24,2.04	0.56<br>0.23,2.63	0.6<br>0.25,2.33	0.68<br>0.25,3.85	CPX	0.88<br>0.30, 2.51	NA	NA	NA	NA
**0.27**<br>**0.1,0.57**	**0.27**<br>**0.14,0.9**	**0.39**<br>**0.28,0.58**	**0.42**<br>**0.17,0.82**	**0.41**<br>**0.26,0.75**	**0.37**<br>**0.2,0.99**	0.36<br>0.18,1.19	0.4<br>0.22,1.02	0.45<br>0.25,1.01	**0.5**<br>**0.25,0.85**	**0.5**<br>**0.35,0.78**	**0.51**<br>**0.33,0.85**	0.5<br>0.28,1.05	0.56<br>0.26,1.01	0.51<br>0.31,1.01	**0.52**<br>**0.31,0.99**	0.48<br>0.23,1.49	0.59<br>0.26,1.13	0.36<br>0.13,2.86	0.52<br>0.26,1.45	**0.56**<br>**0.39,0.85**	0.6<br>0.26,2.17	0.47<br>0.14,12.5	0.73<br>0.48,1.16	0.74<br>0.39,1.79	0.79<br>0.51,1.35	0.9<br>0.42,2.78	0.96<br>0.4,3.57	CPZ	1.00<br>0.14, 6.98	NA	**0.58**<br>**0.34, 0.99**	0.71<br>0.46, 1.09
**0.27**<br>**0.08,0.68**	**0.33**<br>**0.06,0.96**	**0.37**<br>**0.15,0.76**	0.41<br>0.12,1.01	**0.42**<br>**0.15,0.92**	0.44<br>0.11,1.15	0.46<br>0.09,1.3	0.47<br>0.12,1.23	0.5<br>0.14,1.22	0.49<br>0.18,1.06	0.5<br>0.2,1.02	0.52<br>0.2,1.09	0.53<br>0.2,1.11	0.55<br>0.18,1.22	0.55<br>0.19,1.19	0.55<br>0.19,1.2	0.61<br>0.14,1.71	0.58<br>0.18,1.33	0.81<br>0.06,2.83	0.63<br>0.17,1.56	0.57<br>0.24,1.11	0.84<br>0.17,2.35	1.92<br>0.09,6.27	0.76<br>0.3,1.51	0.86<br>0.26,2.01	0.85<br>0.3,1.8	1.14<br>0.3,2.83	1.34<br>0.27,3.53	1.01<br>0.42,1.97	ZOT	NA	NA	NA
**0.26**<br>**0.02,0.95**	0.32<br>0.02,1.31	0.36<br>0.04,1.22	0.4<br>0.04,1.42	0.41<br>0.04,1.44	0.42<br>0.03,1.57	0.45<br>0.03,1.77	0.45<br>0.04,1.69	0.48<br>0.05,1.75	0.47<br>0.05,1.59	0.48<br>0.06,1.59	0.5<br>0.06,1.68	0.54<br>0.05,1.94	0.53<br>0.06,1.81	0.53<br>0.05,1.8	0.53<br>0.06,1.77	0.6<br>0.04,2.31	0.56<br>0.06,1.94	0.8<br>0.02,3.37	0.62<br>0.05,2.37	0.54<br>0.06,1.76	0.82<br>0.06,3.07	2.11<br>0.03,7.21	0.73<br>0.09,2.32	0.85<br>0.08,2.8	0.83<br>0.09,2.75	1.11<br>0.1,3.77	1.3<br>0.1,4.35	0.99<br>0.11,3.08	1.12<br>0.11,3.55	ZUC	NA	NA
**0.2**<br>**0.07,0.47**	**0.25**<br>**0.05,0.71**	**0.29**<br>**0.14,0.51**	**0.31**<br>**0.11,0.68**	**0.32**<br>**0.13,0.64**	**0.33**<br>**0.09,0.82**	**0.35**<br>**0.08,0.97**	**0.36**<br>**0.1,0.86**	**0.38**<br>**0.13,0.86**	**0.37**<br>**0.16,0.73**	**0.38**<br>**0.19,0.69**	**0.38**<br>**0.23,0.75**	**0.37**<br>**0.2,0.91**	**0.42**<br>**0.17,0.86**	**0.42**<br>**0.17,0.86**	**0.42**<br>**0.17,0.85**	0.47<br>0.11,1.25	**0.44**<br>**0.16,0.93**	0.6<br>0.05,2.01	0.39<br>0.2,1.16	**0.43**<br>**0.22,0.76**	0.64<br>0.14,1.72	1.71<br>0.06,6	0.58<br>0.28,1.05	0.65<br>0.25,1.33	0.65<br>0.28,1.25	0.88<br>0.25,2.12	1.03<br>0.24,2.7	0.77<br>0.41,1.29	0.71<br>0.34,1.96	0.73<br>0.25,6.67	THIOR	NA
**0.18**<br>**0.07,0.37**	**0.23**<br>**0.06,0.62**	**0.26**<br>**0.18,0.35**	**0.28**<br>**0.12,0.55**	**0.29**<br>**0.16,0.48**	**0.3**<br>**0.1,0.7**	**0.32**<br>**0.08,0.82**	**0.29**<br>**0.17,0.75**	**0.34**<br>**0.14,0.7**	**0.34**<br>**0.19,0.55**	**0.36**<br>**0.27,0.51**	**0.37**<br>**0.26,0.57**	**0.36**<br>**0.21,0.78**	**0.38**<br>**0.19,0.66**	**0.38**<br>**0.2,0.66**	**0.37**<br>**0.24,0.67**	0.43<br>0.12,1.08	**0.4**<br>**0.18,0.78**	0.57<br>0.05,1.93	0.37<br>0.2,1.02	**0.39**<br>**0.26,0.56**	0.58<br>0.15,1.48	1.57<br>0.06,5.68	**0.52**<br>**0.34,0.75**	0.6<br>0.24,1.23	0.59<br>0.31,1.03	0.8<br>0.28,1.79	0.94<br>0.25,2.38	0.71<br>0.45,1.07	0.69<br>0.36,1.69	0.71<br>0.25,5.88	0.88<br>0.51,1.79	QUE

Table 11.12: Anticholinergic side effects

Treatments are ranked according to their surface under the curve cumulative ranking (SUCRA) for anticholinergic side effects starting with the best. Results of the **network meta-analysis** are presented in the **left lower half** and results from **pairwise comparisons** in the **upper right half**, if available. Comparisons between treatments should be read from left to right and the estimate is in the cell in common between the column-defining treatment and the row-defining treatment. Risk ratios (RRs) lower than 1 favor the column-defining treatment. Cells in bold print indicate significant results.

NA=not available. Drug abbreviations: AMI=Amisulpride, ARI=Aripiprazole, ASE=Asenapine, BRE=Brexpiprazole, CAR=Cariprazine, CPZ=Chlorpromazine, CPX=Clopenthixol, CLO=Clozapine, FPX=Flupenthixol, FLU=Fluphenazine, HAL=Haloperidol, ILO=Iloperidone, LEV=Levomepromazine, LOX=Loxapine, LUR=Lurasidone, MOL=Molindone, OLA=Olanzapine, PAL=Paliperidone, PEN=Penfluridol, PERA=Perazine, PERPH=Perphenazine, PIM=Pimozide, PBO=Placebo, QUE=Quetiapine, RIS=Risperidone, SER=Sertindole, SUL=Sulpiride, THIOR=Thioridazine, THIOTH=Thiothixene, TRIFLU=Trifluoperazine, ZIP=Ziprasidone, ZOT=Zotepine, ZUC=Zuclopenthixol.

Appendix 12: Quality of life: Results from the pairwise meta-analysis

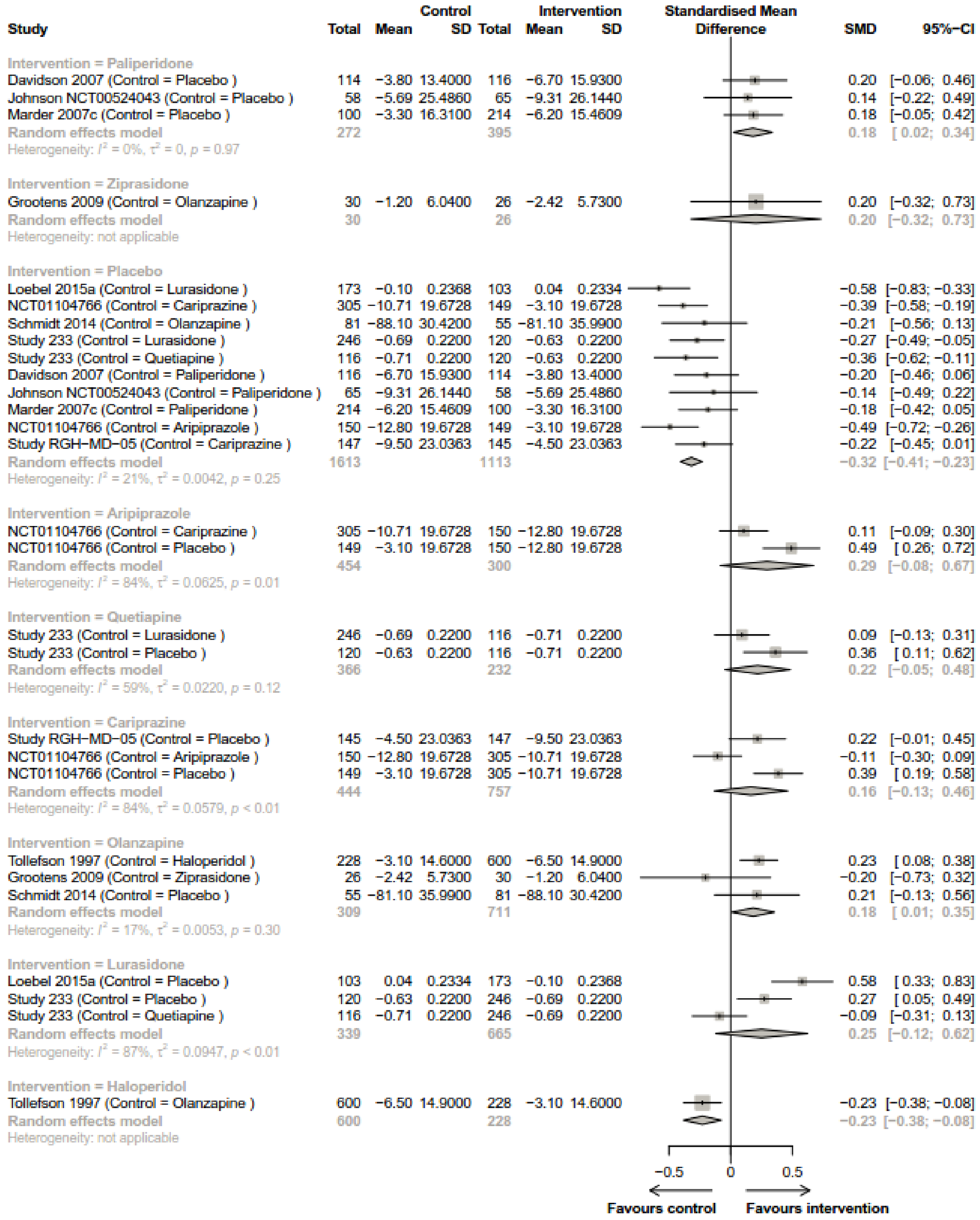

Figure 12: Results for quality of life

As results for quality of life showed a high inconsistency, we present the result from the pairwise comparisons, separated by drugs. SMD: standardized mean difference,. CI: Confidence interval, SD=standard deviation.

Appendix 13: Evaluation of heterogeneity and inconsistency

We inferred the magnitude of heterogeneity by comparing the estimated τ^2 to empirical distributions of heterogeneity typically found in meta-analyses.[7] The predictive τ^2 distribution for mental health outcomes according to Rhodes et al has a median of 0.049 and an IQR of 0.01 to 0.242. Low heterogeneity could be considered when the estimated tau2 is less than the 25% quantile of the empirical distribution, moderate heterogeneity for τ^2 between 25% and 50% quantile and high heterogeneity for τ^2 larger than the 50% quantile. We evaluated global consistency under the assumption of a full design-by-treatment interaction model, using the *decompose.design* function in R package netmeta.

Outcome	Between study variance (τ^2)	Heterogeneity assessment	Percentage of loops showing inconsistency using SIDE splitting	Q	df	p
**Overall symptoms**	0.014	low	10.48%	122.772	113	0.249
**Positive symptoms**	0.012	low	6.90%	81.409	69	0.146
**Negative symptoms**	0.007	low	6.25%	68.492	75	0.689
**Depressive symptoms**	0.009	low	3.92%	48.13	44	0.309
**Quality of life***	0.002	low	50.00%	4.51	2	0.105
**Social functioning**	0	low	0.00%	0.553	3	0.907
**All-cause discontinuation**	0.006	low	9.48%	145.761	125	0.099
**Weight gain (continous)**	0.034	low to moderate	14.06%	53.567	72	0.949
**Use of antiparkinson medication**	0.090	moderate to high	2.63%	64.807	66	0.518
**Akathisia**	0.020	low	8.33%	72.912	64	0.208
**Prolactin elevation (SMD)****	0.048	low to moderate	11.63%	60.342	51	0.174
**Prolactin elevation (MD)****	30.976	high	25.58%	110.768	51	<0.001
**QTc prolongation**	0.001	low	8.33%	41.959	38	0.303
**Sedation**	0.007	low	10.53%	94.764	90	0.345
**Anticholinergic side effects**	0.038	low to moderate	11.36%	3.316	7	0.854
**Discontinuation due to inefficacy**	0.023	low	10.57%	138.067	110	0.036
**Participants with weight gain >7%**	0.031	low to moderate	5.88%	45.609	37	0.157
**Response study defined**	0.104	moderate to high	9.47%	78.201	100	0.948

Table 13: Heterogeneity and inconsistency for all outcomes:
*: Due to high inconsistency, only a pairwise meta-analysis was conducted.
**: The heterogeneity of the prolactin network using MD as effect size is much higher compared to SMD. This may be caused by several issues. Prolactin is measured by assays differing from study to study. Further prolactin as a sexual hormone is diffeerent between men and women.
τ^2: heterogeneity variance. SIDE: Separate Indirect and Direct Evidence approach using netsplit command in R. Q: statistic for assessing inconsistency under the assumption of a full design-by-treatment interaction random effects model using decomp.design command in R, df: degrees of freedom for Q, p: p-value for Q. SMD=standardized mean difference, MD=weighted mean difference.

Appendix 14: Networkplots for secondary outcomes

14.1 Positive symptoms

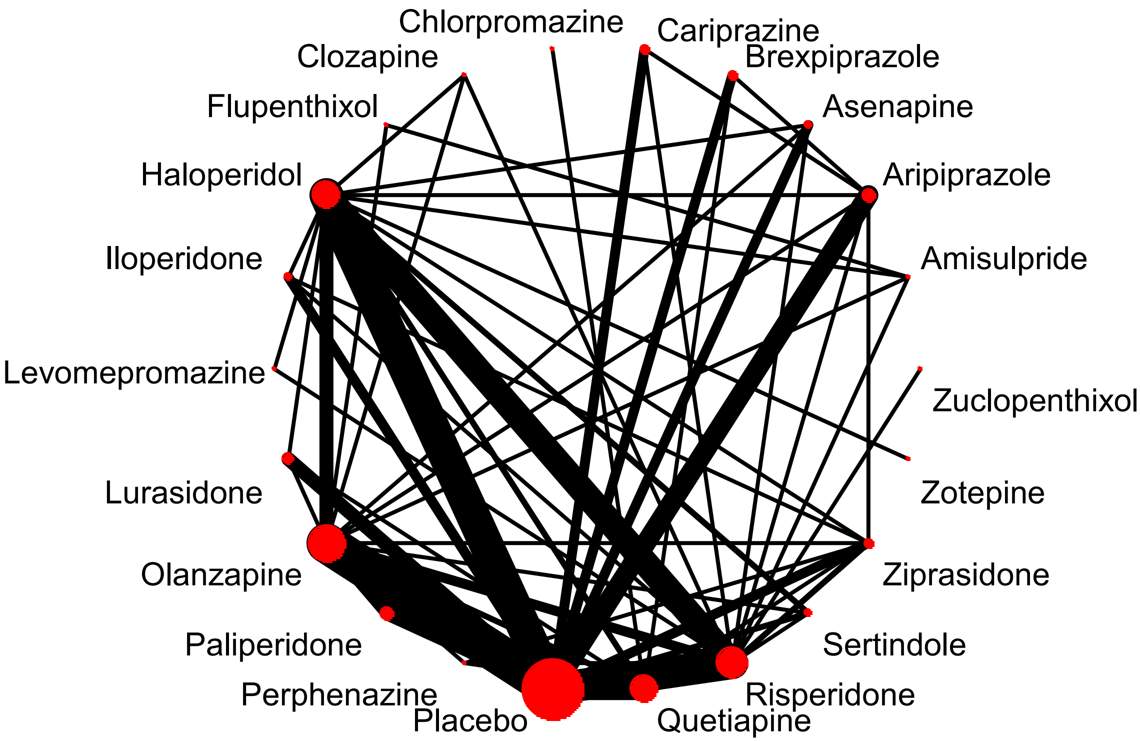

Figure 14.1: Network plot positive symptoms

The size of the nodes corresponds to the number of participants randomized to each treatment. Treatments with direct comparisons are linked with a line; its thickness corresponds to the number of trials evaluating the comparison.

14.2 Negative symptoms

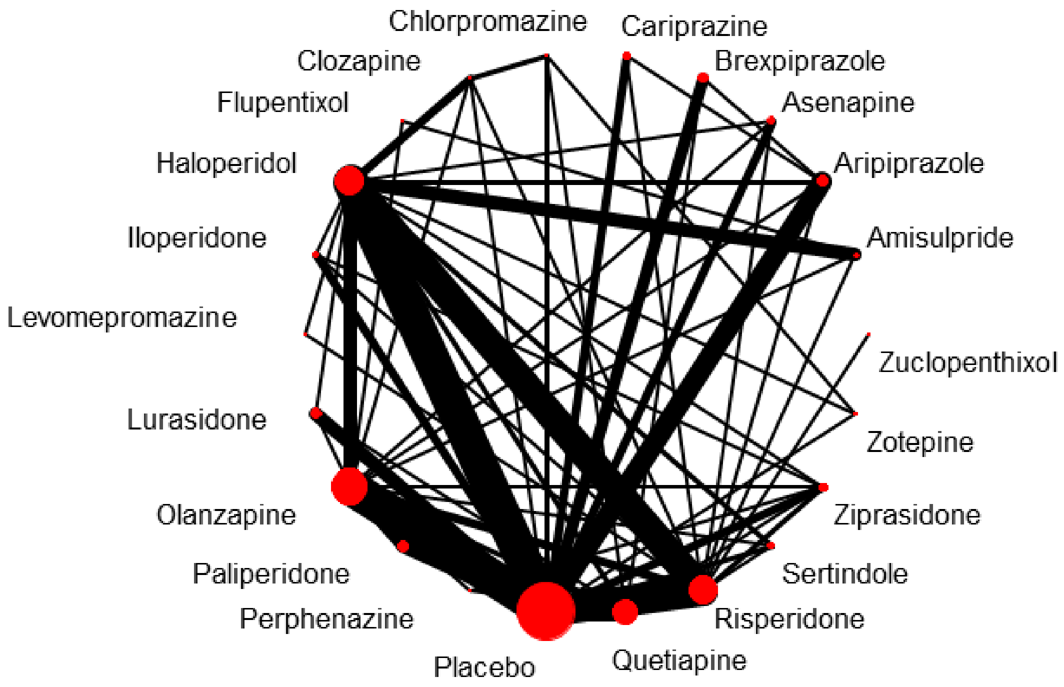

Figure 14.2: Network plot negative symptoms
The size of the nodes corresponds to the number of participants randomized to each treatment. Treatments with direct comparisons are linked with a line; its thickness corresponds to the number of trials evaluating the comparison.

14.3 Depressive symptoms

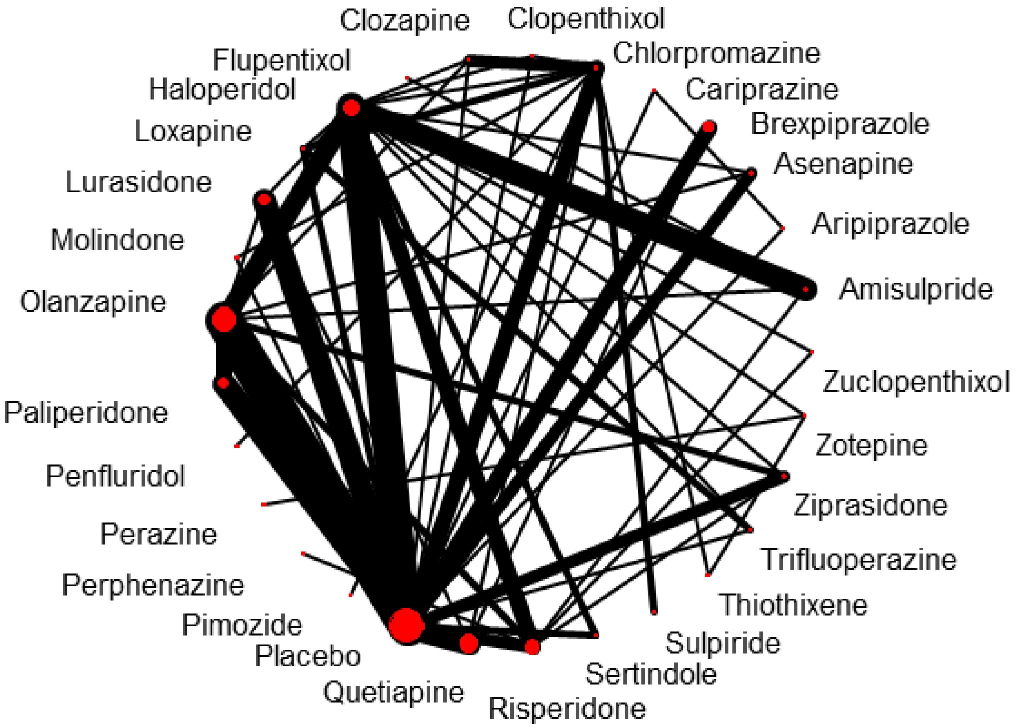

Figure 14.3: Network plot depressive symptoms

The size of the nodes corresponds to the number of participants randomized to each treatment. Treatments with direct comparisons are linked with a line; its thickness corresponds to the number of trials evaluating the comparison.

14.4 Quality of life

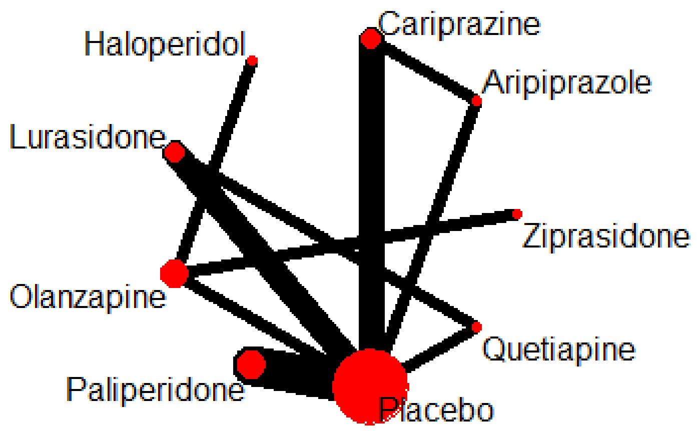

Figure 14.4: Network plot quality of life
The size of the nodes corresponds to the number of participants randomized to each treatment. Treatments with direct comparisons are linked with a line; its thickness corresponds to the number of trials evaluating the comparison. **As 50% of the loops in the network were inconsistent, we only present data from the pairwise meta-analysis.**

Social functioning

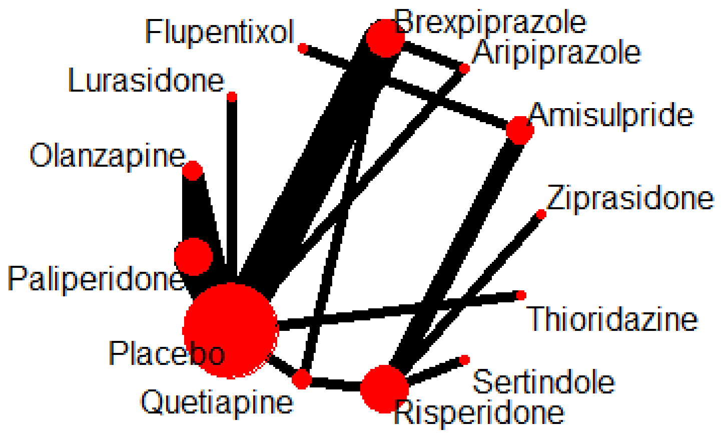

Figure 14.5: Network plot social functioning
The size of the nodes corresponds to the number of participants randomized to each treatment. Treatments with direct comparisons are linked with a line; its thickness corresponds to the number of trials evaluating the comparison.

14.6 All-cause discontinuation

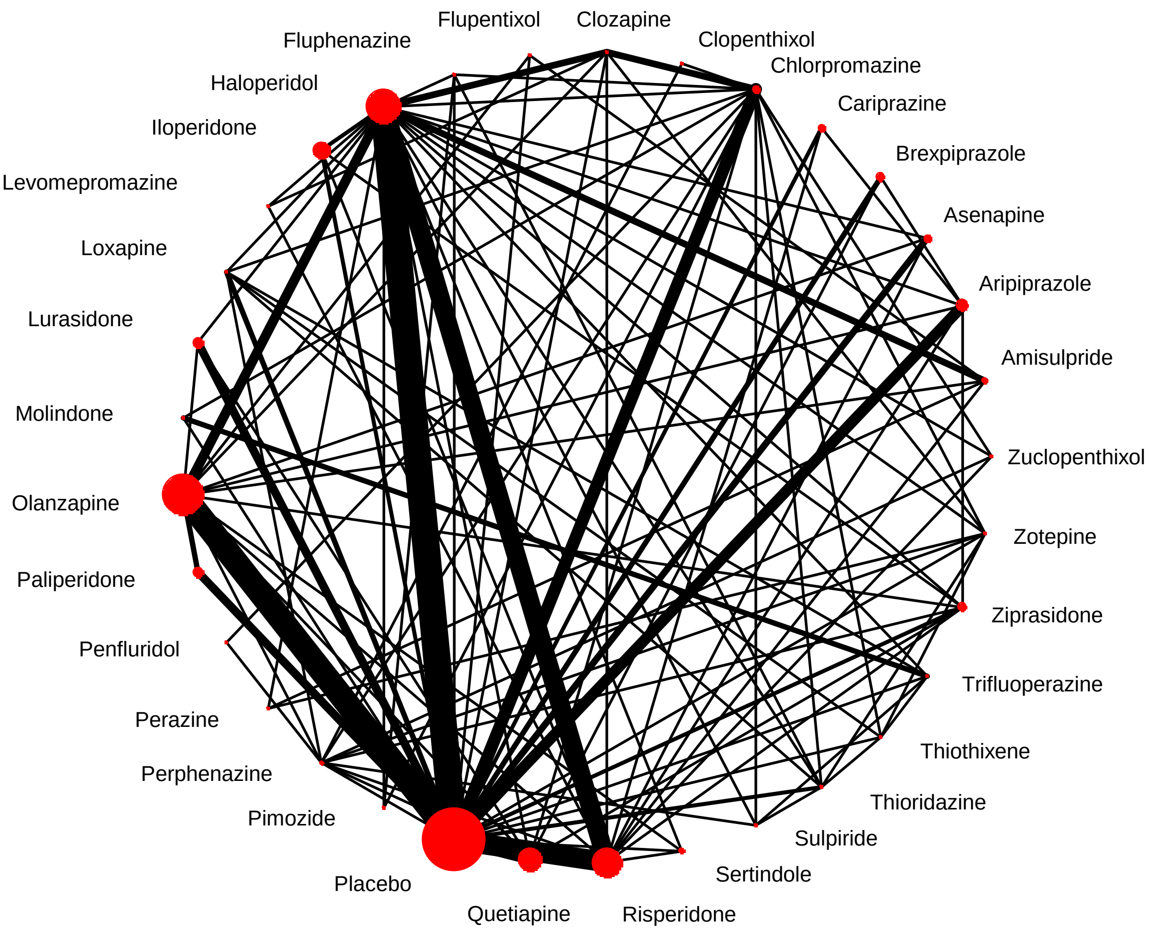

Figure 14.6: Network plot all-cause discontinuation
The size of the nodes corresponds to the number of participants randomized to each treatment. Treatments with direct comparisons are linked with a line; its thickness corresponds to the number of trials evaluating the comparison.

14.7 Weight gain

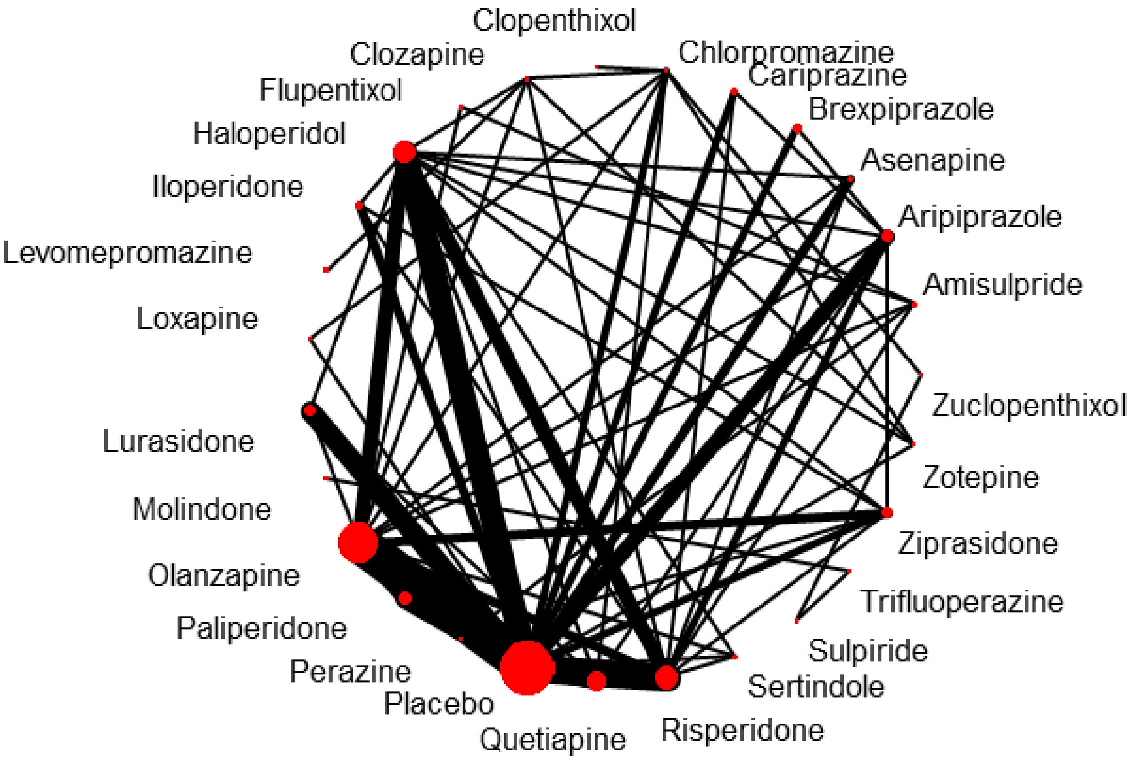

Figure 14.7: Network plot weight gain
The size of the nodes corresponds to the number of participants randomized to each treatment. Treatments with direct comparisons are linked with a line; its thickness corresponds to the number of trials evaluating the comparison.

14.8 Use of antiparkinson medication

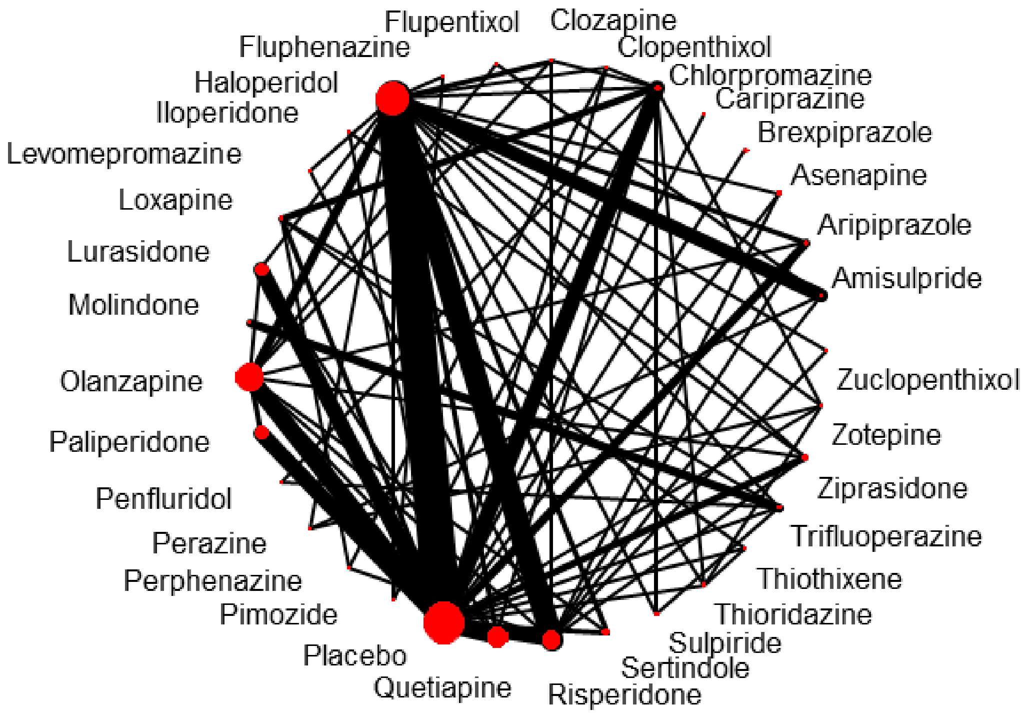

Figure 14.8: Network plot use of antiparkinson medication
The size of the nodes corresponds to the number of participants randomized to each treatment. Treatments with direct comparisons are linked with a line; its thickness corresponds to the number of trials evaluating the comparison.

14.9 Akathisia

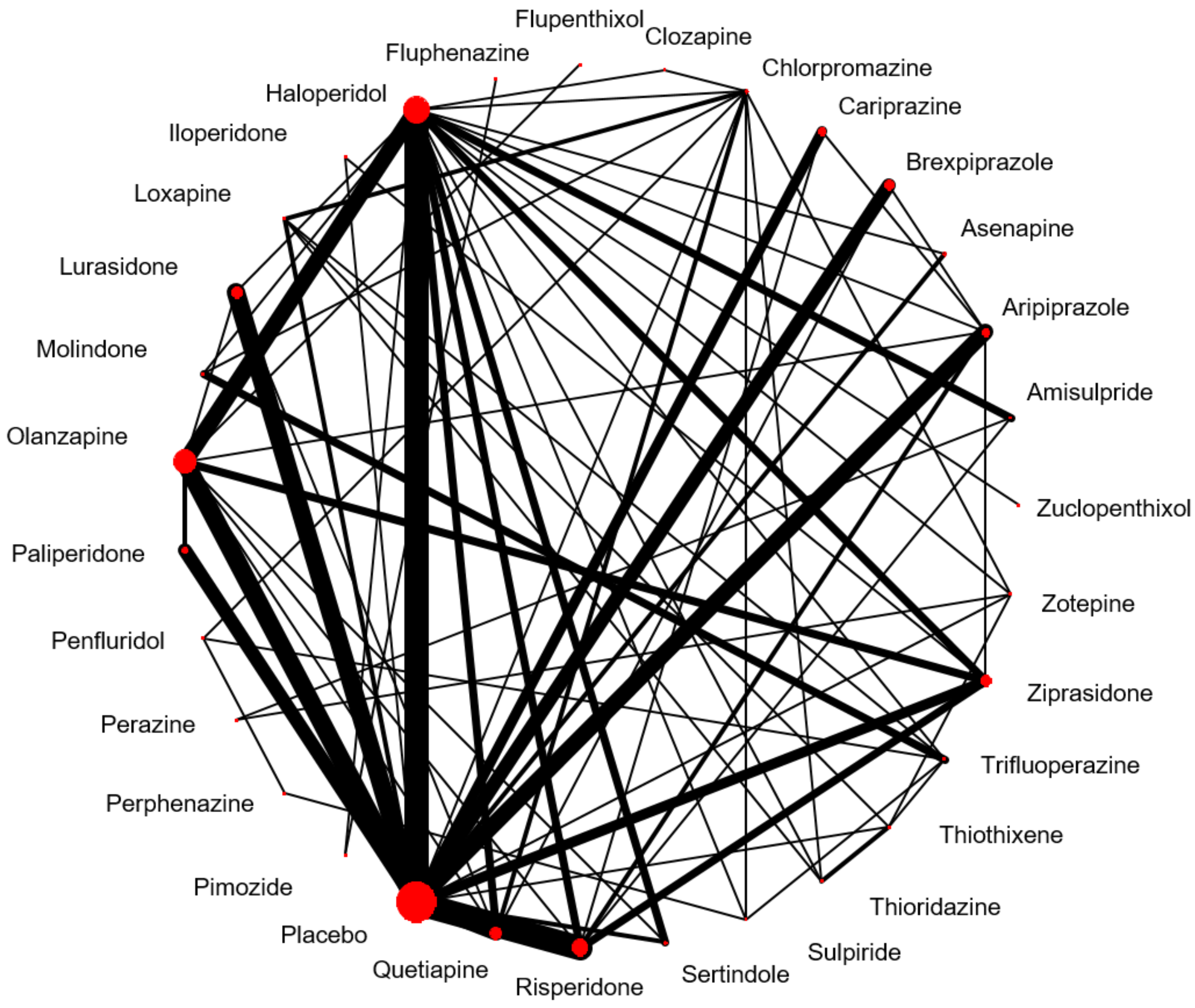

Figure 14.9: Network plot akathisia
The size of the nodes corresponds to the number of participants randomized to each treatment. Treatments with direct comparisons are linked with a line; its thickness corresponds to the number of trials evaluating the comparison.

14.10 Prolactin

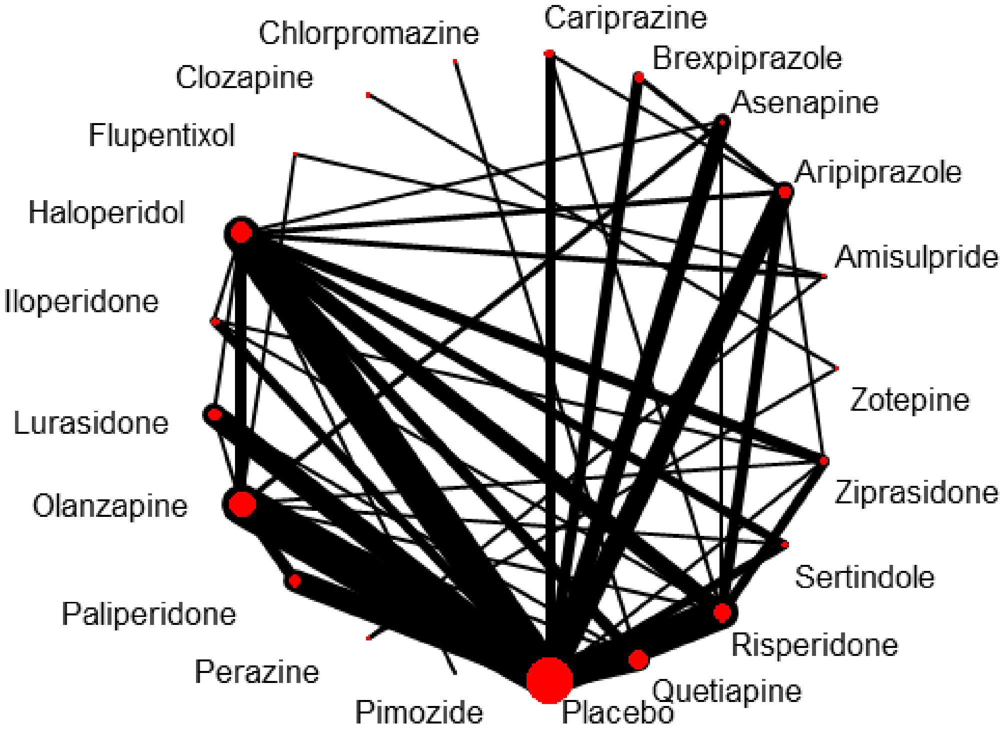

Figure 14.10: Network plot prolactin elevation
The size of the nodes corresponds to the number of participants randomized to each treatment. Treatments with direct comparisons are linked with a line; its thickness corresponds to the number of trials evaluating the comparison.

14.12 QTc prolongation

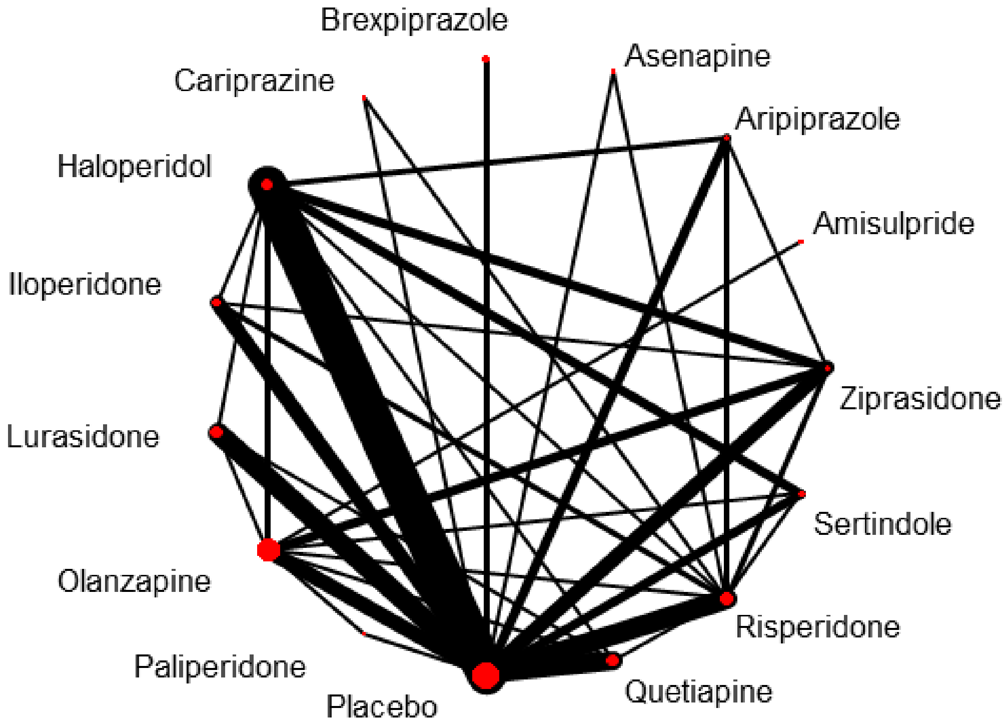

Figure 14.12: Network plot QTc prolongation
The size of the nodes corresponds to the number of participants randomized to each treatment. Treatments with direct comparisons are linked with a line; its thickness corresponds to the number of trials evaluating the comparison.

14.13 Sedation

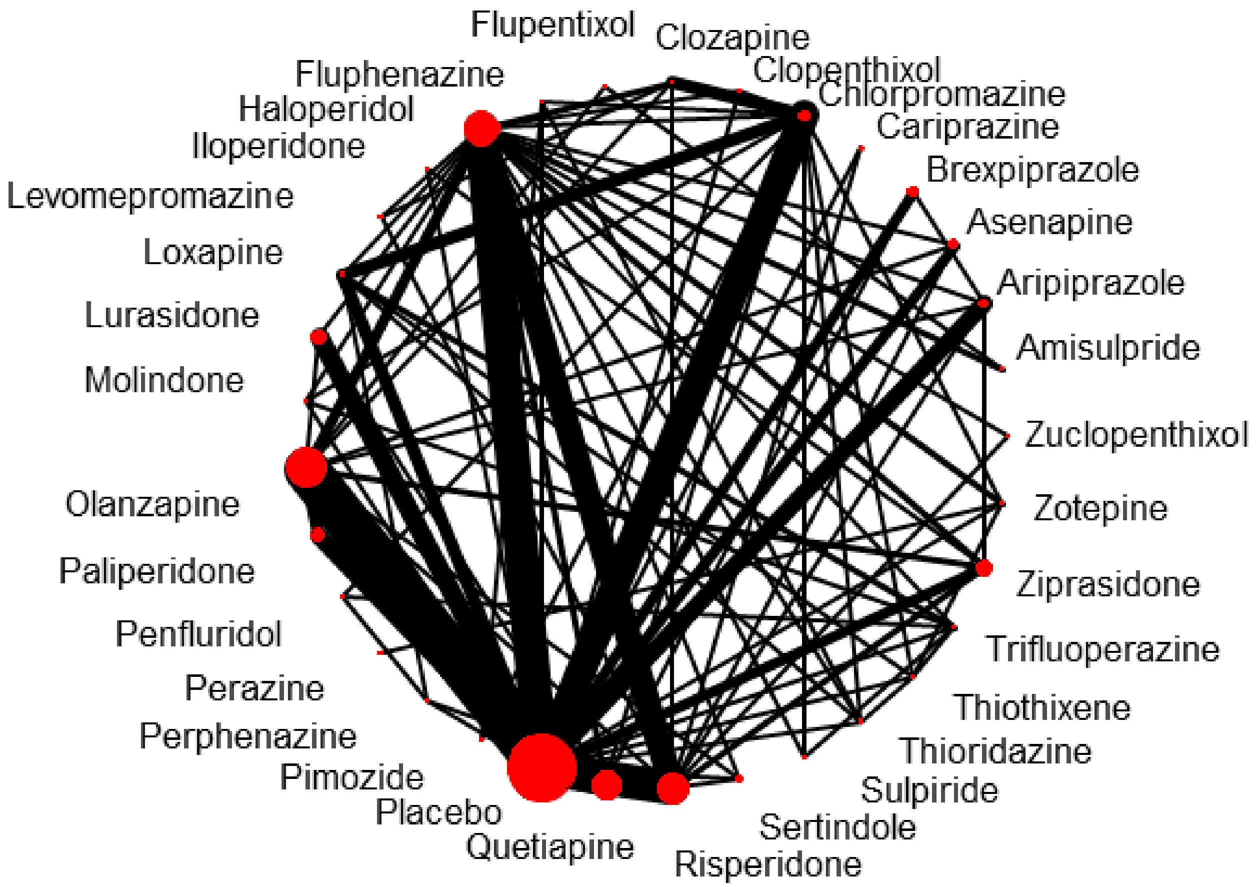

Figure 14.13: Network plot sedation
The size of the nodes corresponds to the number of participants randomized to each treatment. Treatments with direct comparisons are linked with a line; its thickness corresponds to the number of trials evaluating the comparison.

14.14 Anticholinergic side effects

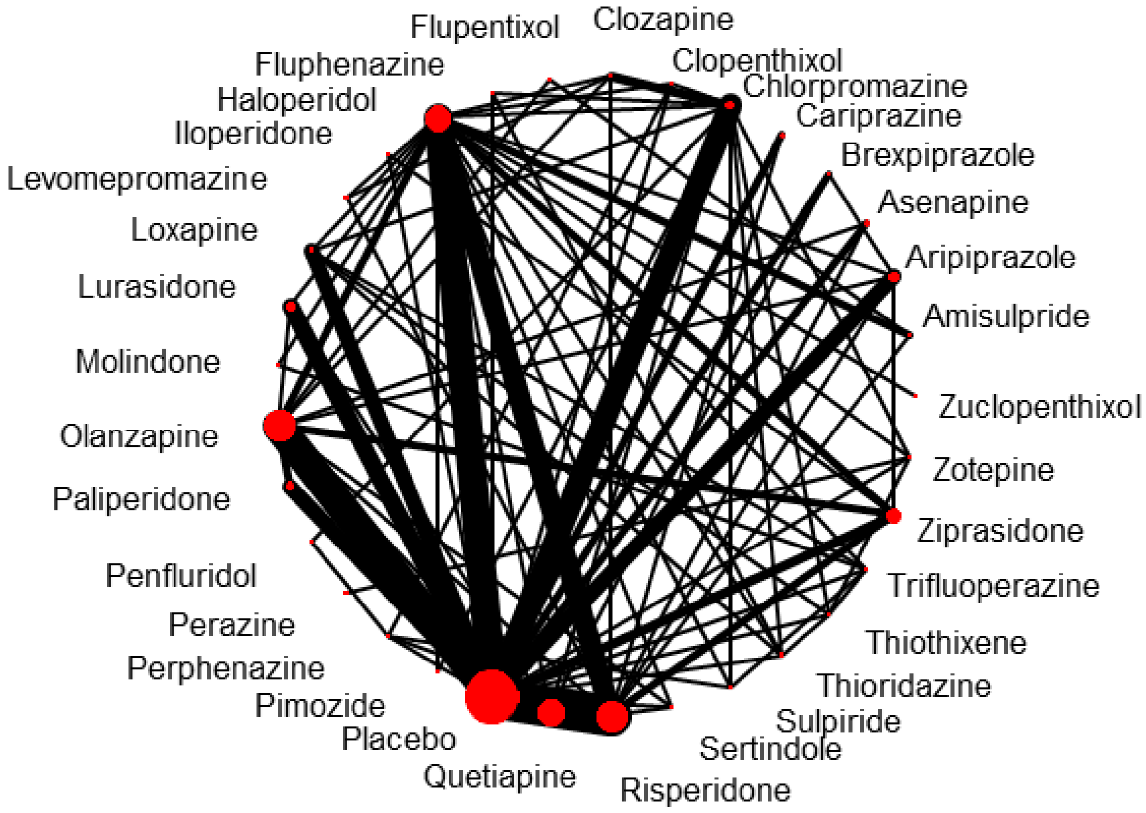

Figure 14.14: Network plot anticholinergic side effects
The size of the nodes corresponds to the number of participants randomized to each treatment. Treatments with direct comparisons are linked with a line; its thickness corresponds to the number of trials evaluating the comparison.

Appendix 15: Grading the evidence of the network meta-analysis using CINeMA

Our judgements described below are based on the recommendations of the online documentation of CINeMA (http://cinema.ispm.ch/#doc). We graded all outcomes individually using the approach below.

15.1 Reasons for downgrading

1. Within-study bias:

We classified an overall risk of bias for every study based on the individual risk of bias items. The classification is based on the following reference: "Furukawa, Toshi A.; Salanti, Georgia; Atkinson, Lauren Z.; Leucht, Stefan; Ruhe, Henricus G.; Turner, Erick H. et al. (2016): Comparative efficacy and acceptability of first-generation and second-generation antidepressants in the acute treatment of major depression. Protocol for a network meta-analysis. In: *BMJ open* 6 (7), e010919. DOI: 10.1136/bmjopen-2015-010919."

The table below shows how the global rating is coded base on the individual items of the risk of bias tool:

Global rating	Items with high risk	Items with unclear risk
**low risk**	0	=< 3
**moderate**	0	>3
**moderate**	1	any
**high risk**	>1	any

Table 15.1: Global risk of bias rating based on the individual risk of bias items

2. Across-study bias (Publication bias):

Our search was comprehensive including study registers and pharmaceutical registers. So even if we missed small unpublished trials it seems not very likely that they would influence the results strongly. Nevertheless we assessed publication bias with the following procedure. We conducted funnel-plots for antipsychotics vs. placebo and for haloperidol versus all other antipsychotics. Haloperidol was chosen as the reference because it had served as a comparator antipsychotic for many older and newer antipsychotics.

1) Antipsychotics vs. placebo:

We first produced funnel plots for every outcome comparing all antipsychotics against placebo. If an overall publication bias was detected we assumed publication bias for all comparisons except for the following situation: In case more than 10 studies were available for the respective comparison (e.g. haloperidol vs. placebo), and there was no evidence for publication bias by visual inspection, we did not downgrade for publication bias. Funnel plots with less than ten studies are not meaningful (Higgins 2011). All other comparisons were downgraded one level for publication bias. This procedure is illustrated below.

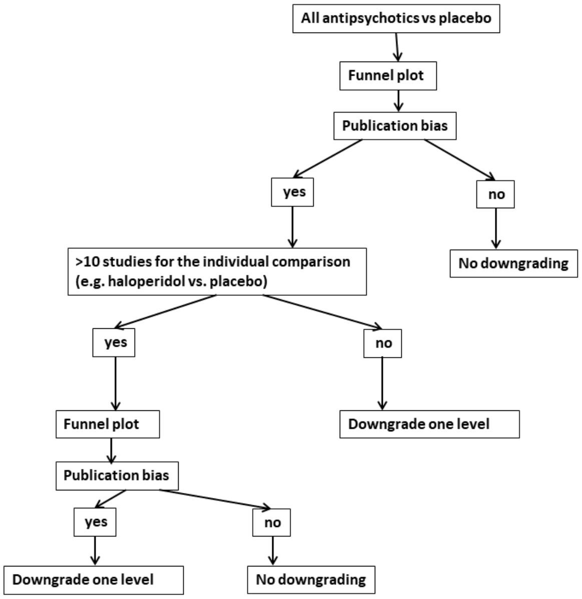

Figure 1: Evaluation of publication bis for placebo comparisons

2) Antipsychotics vs. haloperidol:

We produced funnel plots for every outcome comparing haloperidol with all other antipsychotics pooled. If an overall publication bias was detected we assumed publication bias for all comparisons except for the following situation: In case more than 10 studies were available for the respective comparison (e.g. haloperidol vs. risperidone), and there was no evidence for publication bias by visual inspection, we did not downgrade for publication bias. Funnel plots with less than ten studies are not meaningful (Higgins 2011). All other comparisons were downgraded one level for publication bias. This procedure is illustrated below.

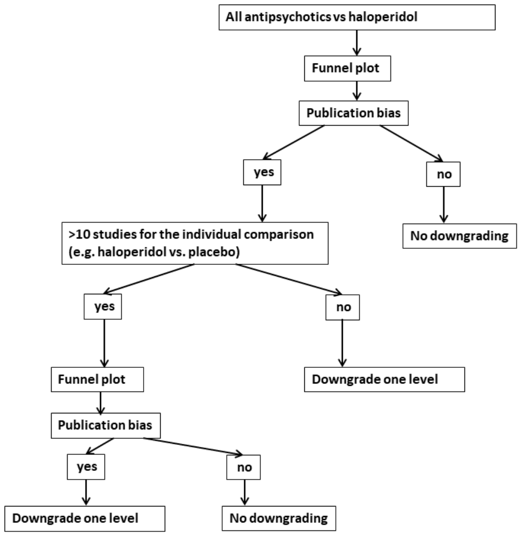

Figure2: Evaluation of publication bis for placebo comparisons

3. Indirectness: To control for transitivity studies with treatment-resistant patients, predominant negative symptoms, first episode, children or elderly were excluded a priori, as they are known to be different from the adult acute episode patient. Therefore no indirectness was assumed and no comparison was downgraded for this reason.

4. Imprecision:

For placebo comparisons the clinically meaningful threshold was set at a standardized mean difference of higher or lower than 0. For dichotomous outcomes an odds ratio lower or higher than 1 was considered clinically meaningful. If the confidence interval crossed the threshold the comparison was downgraded one level. For comparisons of two antipsychotics the clinically meaningful threshold was set at standardized mean differences of -0.1 and 0.1 for continuous outcomes and at odds ratio of 0.8 and 1.25 for dichotomous outcomes. If the confidence interval crossed one threshold the comparison was downgraded one level. Crossing both thresholds resulted in a downgrading of two levels.

5. Heterogeneity: We made use of prediction intervals to prepopulate judgments on heterogeneity and its implications on the quality of the network treatment effects. In particular, we judged the agreement of conclusions based on confidence and prediction intervals in relation to the clinically important effect size.

The same thresholds for clinically meaningful thresholds as above were used and the recommendations automatically provided by CINeMA were followed (http://cinema.ispm.ch/#doc).

6. Incoherence: We assessed incoherence both globally using the design-by-treatment interaction model and locally using the Separate Indirect from Direct Evidence (SIDE, or node-splitting approach).[8] We did not downgrade comparisons that had only direct evidence with respect to incoherence. The same judgement was made for comparisons that had both direct and indirect evidence with the contribution of direct evidence being more than 90%. For comparisons that had only indirect evidence, we downgraded due to incoherence one or two levels depending on whether the p-value of the design by treatment interaction model was between 0.01 and 0.10 or less than 0.01 respectively. We use the rules described in the table below to infer about our confidence regarding incoherence in network treatment effects informed by less than 90% from direct evidence.

		**Design-by-treatment interaction model**		
		p-value>0.1	0.01<p-value<0.1	p-value<0.01
**SIDE**	p-value>0.1	no downgrading	no downgrading	one level
	0.01<p-value<0.1	one level	one level	two levels
	p-value<0.01	one level	two levels	two levels

Figure 15.1: Assessing incoherence
Summary of recommendations on judging incoherence of NMA treatment effects for mixed evidence comparisons which are informed less than 90% by direct evidence (http://cinema.ispm.ch/#doc). SIDE=Separate Indirect from Direct Evidence.

15.2 Confidence in evidence for all antipsychotics compared to placebo

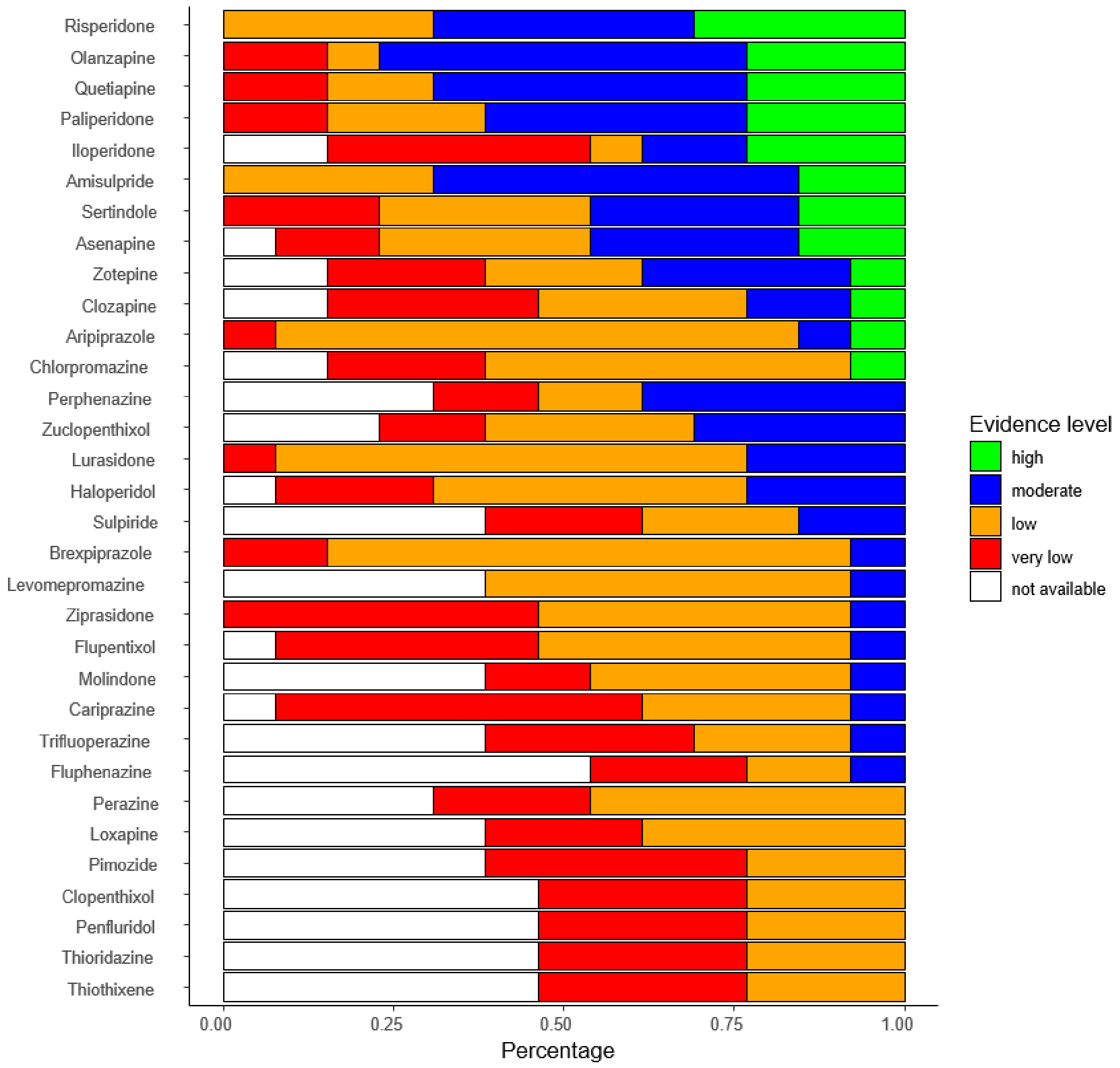

Figure 15.2: Confidence in evidence for all antipsychotics compared to placebo
In this figure we present a summary of the confidence in the evidence for the individual drugs compared to placebo according to CINeMA (Confidence in Network Meta-analysis). The 13 outcomes presented in Figures 2 and 3 were considered. The bars present the percentage of outcomes with each evidence level (e.g. for olanzapine 23% of the reported outcomes had a high evidence level, 54% a moderate, 8% a low and 15% a very low one). The antipsychotics with the largest proportion of outcomes ranked with high certainty of evidence are presented on top (e.g. for risperidone 31% of the outcomes had a high level of evidence). As CINeMA does not consider comparisons for which no data are available, we added this information in the white bars (e.g. for thiothixene for 46% of the outcomes no data were available at all). Colour code: green=high, blue=moderate, orange=low, red=very low, white=percentage of outcomes with no data available.

15.3 *CINeMA for the primary outcome "overall change in symptoms"*

Comparison	Nature of evidence	Confidence level	Downgrading
Amisulpride vs. Aripiprazole	indirect	moderate	Heterogeneity
Amisulpride vs. Asenapine	indirect	moderate	Heterogeneity
Amisulpride vs. Brexpiprazole	indirect	high	
Amisulpride vs. Cariprazine	indirect	low	Study limitations, Heterogeneity
Amisulpride vs. Chlorpromazine	indirect	moderate	Imprecision
Amisulpride vs. Clopenthixol	indirect	low	Imprecision, Heterogeneity
Amisulpride vs. Clozapine	indirect	low	Imprecision, Heterogeneity
Amisulpride vs. Flupentixol	mixed	very low	Study limitations, Imprecision, Incoherence
Amisulpride vs. Fluphenazine	indirect	moderate	Imprecision
Amisulpride vs. Haloperidol	mixed	moderate	Heterogeneity
Amisulpride vs. Iloperidone	indirect	high	
Amisulpride vs. Levomepromazine	indirect	moderate	Imprecision
Amisulpride vs. Loxapine	indirect	moderate	Imprecision
Amisulpride vs. Lurasidone	indirect	moderate	Heterogeneity
Amisulpride vs. Molindone	indirect	low	Imprecision, Heterogeneity
Amisulpride vs. Olanzapine	mixed	very low	Imprecision, Heterogeneity, Incoherence
Amisulpride vs. Paliperidone	indirect	moderate	Imprecision
Amisulpride vs. Penfluridol	indirect	very low	Study limitations, Serious Imprecision
Amisulpride vs. Perazine	mixed	moderate	Imprecision
Amisulpride vs. Perphenazine	indirect	low	Imprecision, Heterogeneity

Comparison	Nature of evidence	Confidence level	Downgrading
Amisulpride vs. Pimozide	indirect	very low	Study limitations, Imprecision, Heterogeneity
Amisulpride vs. Placebo	indirect	moderate	Publication Bias
Amisulpride vs. Quetiapine	indirect	moderate	Heterogeneity
Amisulpride vs. Risperidone	mixed	low	Imprecision, Incoherence
Amisulpride vs. Sertindole	indirect	moderate	Heterogeneity
Amisulpride vs. Sulpiride	indirect	low	Serious Imprecision
Amisulpride vs. Thioridazine	indirect	very low	Study limitations, Serious Imprecision
Amisulpride vs. Thiothixene	indirect	very low	Study limitations, Serious Imprecision
Amisulpride vs. Trifluoperazine	indirect	low	Study limitations, Heterogeneity
Amisulpride vs. Ziprasidone	indirect	moderate	Heterogeneity
Amisulpride vs. Zotepine	indirect	low	Serious Imprecision
Amisulpride vs. Zuclopenthixol	indirect	low	Imprecision, Heterogeneity
Aripiprazole vs. Asenapine	indirect	very low	Study limitations, Serious Imprecision
Aripiprazole vs. Brexpiprazole	mixed	very low	Imprecision, Heterogeneity, Incoherence
Aripiprazole vs. Cariprazine	mixed	very low	Study limitations, Serious Imprecision, Incoherence
Aripiprazole vs. Chlorpromazine	indirect	very low	Study limitations, Serious Imprecision
Aripiprazole vs. Clopenthixol	indirect	very low	Study limitations, Serious Imprecision
Aripiprazole vs. Clozapine	indirect	moderate	Study limitations
Aripiprazole vs. Flupentixol	indirect	very low	Study limitations, Serious Imprecision
Aripiprazole vs. Fluphenazine	indirect	very low	Study limitations, Serious Imprecision
Aripiprazole vs. Haloperidol	mixed	very low	Study limitations, Imprecision, Heterogeneity, Incoherence
Aripiprazole vs. Iloperidone	indirect	very low	Study limitations, Imprecision, Heterogeneity

Comparison	Nature of evidence	Confidence level	Downgrading
Aripiprazole vs. Levomepromazine	indirect	low	Serious Imprecision
Aripiprazole vs. Loxapine	indirect	very low	Study limitations, Serious Imprecision
Aripiprazole vs. Lurasidone	indirect	low	Serious Imprecision
Aripiprazole vs. Molindone	indirect	very low	Study limitations, Serious Imprecision
Aripiprazole vs. Olanzapine	mixed	very low	Study limitations, Imprecision, Heterogeneity
Aripiprazole vs. Paliperidone	indirect	low	Imprecision, Heterogeneity
Aripiprazole vs. Penfluridol	indirect	very low	Study limitations, Serious Imprecision
Aripiprazole vs. Perazine	indirect	low	Serious Imprecision
Aripiprazole vs. Perphenazine	indirect	low	Imprecision, Heterogeneity
Aripiprazole vs. Pimozide	indirect	very low	Study limitations, Serious Imprecision
Aripiprazole vs. Placebo	mixed	low	Study limitations, Publication Bias
Aripiprazole vs. Quetiapine	indirect	low	Serious Imprecision
Aripiprazole vs. Risperidone	mixed	very low	Imprecision, Heterogeneity, Incoherence
Aripiprazole vs. Sertindole	indirect	very low	Study limitations, Serious Imprecision
Aripiprazole vs. Sulpiride	indirect	very low	Study limitations, Serious Imprecision
Aripiprazole vs. Thioridazine	indirect	very low	Study limitations, Serious Imprecision
Aripiprazole vs. Thiothixene	indirect	very low	Study limitations, Serious Imprecision
Aripiprazole vs. Trifluoperazine	indirect	very low	Study limitations, Serious Imprecision
Aripiprazole vs. Ziprasidone	mixed	very low	Study limitations, Serious Imprecision
Aripiprazole vs. Zotepine	indirect	low	Imprecision, Heterogeneity
Aripiprazole vs. Zuclopenthixol	indirect	very low	Study limitations, Serious Imprecision
Asenapine vs. Brexpiprazole	indirect	low	Imprecision, Heterogeneity

Comparison	Nature of evidence	Confidence level	Downgrading
Asenapine vs. Cariprazine	indirect	very low	Study limitations, Serious Imprecision
Asenapine vs. Chlorpromazine	indirect	very low	Study limitations, Serious Imprecision
Asenapine vs. Clopenthixol	indirect	very low	Study limitations, Serious Imprecision
Asenapine vs. Clozapine	indirect	moderate	Study limitations
Asenapine vs. Flupentixol	indirect	very low	Study limitations, Serious Imprecision
Asenapine vs. Fluphenazine	indirect	very low	Study limitations, Serious Imprecision
Asenapine vs. Haloperidol	mixed	very low	Study limitations, Imprecision, Heterogeneity, Incoherence
Asenapine vs. Iloperidone	indirect	very low	Study limitations, Serious Imprecision
Asenapine vs. Levomepromazine	indirect	low	Serious Imprecision
Asenapine vs. Loxapine	indirect	very low	Study limitations, Serious Imprecision
Asenapine vs. Lurasidone	indirect	low	Serious Imprecision
Asenapine vs. Molindone	indirect	very low	Study limitations, Serious Imprecision
Asenapine vs. Olanzapine	mixed	low	Study limitations, Imprecision
Asenapine vs. Paliperidone	indirect	low	Imprecision, Heterogeneity
Asenapine vs. Penfluridol	indirect	very low	Study limitations, Serious Imprecision
Asenapine vs. Perazine	indirect	low	Serious Imprecision
Asenapine vs. Perphenazine	indirect	low	Imprecision, Heterogeneity
Asenapine vs. Pimozide	indirect	very low	Study limitations, Serious Imprecision
Asenapine vs. Placebo	mixed	low	Study limitations, Publication Bias
Asenapine vs. Quetiapine	indirect	low	Serious Imprecision
Asenapine vs. Risperidone	mixed	very low	Imprecision, Heterogeneity, Incoherence
Asenapine vs. Sertindole	indirect	very low	Study limitations, Serious Imprecision

Comparison	Nature of evidence	Confidence level	Downgrading
Asenapine vs. Sulpiride	indirect	very low	Study limitations, Serious Imprecision
Asenapine vs. Thioridazine	indirect	very low	Study limitations, Serious Imprecision
Asenapine vs. Thiothixene	indirect	very low	Study limitations, Serious Imprecision
Asenapine vs. Trifluoperazine	indirect	very low	Study limitations, Serious Imprecision
Asenapine vs. Ziprasidone	indirect	very low	Study limitations, Serious Imprecision
Asenapine vs. Zotepine	indirect	low	Imprecision, Heterogeneity
Asenapine vs. Zuclopenthixol	indirect	very low	Study limitations, Serious Imprecision
Brexpiprazole vs. Cariprazine	indirect	very low	Study limitations, Serious Imprecision
Brexpiprazole vs. Chlorpromazine	indirect	very low	Study limitations, Imprecision, Heterogeneity
Brexpiprazole vs. Clopenthixol	indirect	very low	Study limitations, Serious Imprecision
Brexpiprazole vs. Clozapine	indirect	moderate	Study limitations
Brexpiprazole vs. Flupentixol	indirect	low	Serious Imprecision
Brexpiprazole vs. Fluphenazine	indirect	very low	Study limitations, Serious Imprecision
Brexpiprazole vs. Haloperidol	indirect	low	Study limitations, Imprecision
Brexpiprazole vs. Iloperidone	indirect	low	Serious Imprecision
Brexpiprazole vs. Levomepromazine	indirect	low	Serious Imprecision
Brexpiprazole vs. Loxapine	indirect	very low	Study limitations, Imprecision, Heterogeneity
Brexpiprazole vs. Lurasidone	indirect	low	Imprecision, Heterogeneity
Brexpiprazole vs. Molindone	indirect	very low	Study limitations, Serious Imprecision
Brexpiprazole vs. Olanzapine	indirect	moderate	Heterogeneity
Brexpiprazole vs. Paliperidone	indirect	moderate	Imprecision
Brexpiprazole vs. Penfluridol	indirect	very low	Study limitations, Serious Imprecision

Comparison	Nature of evidence	Confidence level	Downgrading
Brexpiprazole vs. Perazine	indirect	low	Serious Imprecision
Brexpiprazole vs. Perphenazine	indirect	moderate	Imprecision
Brexpiprazole vs. Pimozide	indirect	very low	Study limitations, Serious Imprecision
Brexpiprazole vs. Placebo	mixed	very low	Publication Bias, Heterogeneity, Serious Incoherence
Brexpiprazole vs. Quetiapine	mixed	very low	Study limitations, Imprecision, Heterogeneity, Incoherence
Brexpiprazole vs. Risperidone	indirect	moderate	Heterogeneity
Brexpiprazole vs. Sertindole	indirect	low	Imprecision, Heterogeneity
Brexpiprazole vs. Sulpiride	indirect	very low	Study limitations, Serious Imprecision
Brexpiprazole vs. Thioridazine	indirect	very low	Study limitations, Imprecision, Heterogeneity
Brexpiprazole vs. Thiothixene	indirect	low	Study limitations, Imprecision
Brexpiprazole vs. Trifluoperazine	indirect	very low	Study limitations, Serious Imprecision
Brexpiprazole vs. Ziprasidone	indirect	low	Imprecision, Heterogeneity
Brexpiprazole vs. Zotepine	indirect	moderate	Imprecision
Brexpiprazole vs. Zuclopenthixol	indirect	very low	Study limitations, Imprecision, Heterogeneity
Cariprazine vs. Chlorpromazine	indirect	very low	Study limitations, Serious Imprecision
Cariprazine vs. Clopenthixol	indirect	very low	Study limitations, Serious Imprecision
Cariprazine vs. Clozapine	indirect	moderate	Study limitations
Cariprazine vs. Flupentixol	indirect	very low	Study limitations, Serious Imprecision
Cariprazine vs. Fluphenazine	indirect	very low	Study limitations, Serious Imprecision
Cariprazine vs. Haloperidol	indirect	very low	Study limitations, Imprecision, Heterogeneity
Cariprazine vs. Iloperidone	indirect	very low	Study limitations, Serious Imprecision
Cariprazine vs. Levomepromazine	indirect	very low	Study limitations, Serious Imprecision

Comparison	Nature of evidence	Confidence level	Downgrading
Cariprazine vs. Loxapine	indirect	very low	Study limitations, Serious Imprecision
Cariprazine vs. Lurasidone	indirect	low	Serious Imprecision
Cariprazine vs. Molindone	indirect	very low	Study limitations, Serious Imprecision
Cariprazine vs. Olanzapine	indirect	low	Study limitations, Imprecision
Cariprazine vs. Paliperidone	indirect	very low	Study limitations, Imprecision, Heterogeneity
Cariprazine vs. Penfluridol	indirect	very low	Study limitations, Serious Imprecision
Cariprazine vs. Perazine	indirect	very low	Study limitations, Serious Imprecision
Cariprazine vs. Perphenazine	indirect	very low	Study limitations, Imprecision, Heterogeneity
Cariprazine vs. Pimozide	indirect	very low	Study limitations, Serious Imprecision
Cariprazine vs. Placebo	mixed	very low	Study limitations, Publication Bias, Heterogeneity, Serious Incoherence
Cariprazine vs. Quetiapine	indirect	very low	Study limitations, Imprecision, Heterogeneity
Cariprazine vs. Risperidone	mixed	low	Study limitations, Imprecision
Cariprazine vs. Sertindole	indirect	very low	Study limitations, Serious Imprecision
Cariprazine vs. Sulpiride	indirect	very low	Study limitations, Serious Imprecision
Cariprazine vs. Thioridazine	indirect	very low	Study limitations, Serious Imprecision
Cariprazine vs. Thiothixene	indirect	very low	Study limitations, Serious Imprecision
Cariprazine vs. Trifluoperazine	indirect	very low	Study limitations, Serious Imprecision
Cariprazine vs. Ziprasidone	indirect	very low	Study limitations, Serious Imprecision
Cariprazine vs. Zotepine	indirect	low	Study limitations, Imprecision
Cariprazine vs. Zuclopenthixol	indirect	very low	Study limitations, Serious Imprecision
Chlorpromazine vs. Clopenthixol	mixed	very low	Study limitations, Serious Imprecision
Chlorpromazine vs. Clozapine	mixed	low	Study limitations, Incoherence

Comparison	Nature of evidence	Confidence level	Downgrading
Chlorpromazine vs. Flupentixol	indirect	very low	Study limitations, Serious Imprecision
Chlorpromazine vs. Fluphenazine	mixed	very low	Study limitations, Serious Imprecision
Chlorpromazine vs. Haloperidol	mixed	very low	Study limitations, Serious Imprecision
Chlorpromazine vs. Iloperidone	indirect	very low	Study limitations, Imprecision, Heterogeneity
Chlorpromazine vs. Levomepromazine	indirect	low	Serious Imprecision
Chlorpromazine vs. Loxapine	mixed	very low	Study limitations, Serious Imprecision
Chlorpromazine vs. Lurasidone	indirect	low	Serious Imprecision
Chlorpromazine vs. Molindone	mixed	very low	Study limitations, Serious Imprecision
Chlorpromazine vs. Olanzapine	indirect	very low	Study limitations, Imprecision, Heterogeneity
Chlorpromazine vs. Paliperidone	indirect	low	Serious Imprecision
Chlorpromazine vs. Penfluridol	mixed	very low	Study limitations, Serious Imprecision
Chlorpromazine vs. Perazine	indirect	very low	Study limitations, Serious Imprecision
Chlorpromazine vs. Perphenazine	indirect	low	Serious Imprecision
Chlorpromazine vs. Pimozide	mixed	very low	Study limitations, Serious Imprecision
Chlorpromazine vs. Placebo	mixed	low	Study limitations, Publication Bias
Chlorpromazine vs. Quetiapine	mixed	very low	Study limitations, Serious Imprecision
Chlorpromazine vs. Risperidone	indirect	very low	Study limitations, Imprecision, Heterogeneity
Chlorpromazine vs. Sertindole	indirect	very low	Study limitations, Serious Imprecision
Chlorpromazine vs. Sulpiride	mixed	low	Serious Imprecision
Chlorpromazine vs. Thioridazine	mixed	very low	Study limitations, Serious Imprecision
Chlorpromazine vs. Thiothixene	indirect	very low	Study limitations, Serious Imprecision
Chlorpromazine vs. Trifluoperazine	indirect	very low	Study limitations, Serious Imprecision

Comparison	Nature of evidence	Confidence level	Downgrading
Chlorpromazine vs. Ziprasidone	indirect	very low	Study limitations, Serious Imprecision
Chlorpromazine vs. Zotepine	mixed	very low	Imprecision, Heterogeneity, Serious Incoherence
Chlorpromazine vs. Zuclopenthixol	mixed	low	Serious Imprecision
Clopenthixol vs. Clozapine	indirect	low	Study limitations, Imprecision
Clopenthixol vs. Flupentixol	indirect	very low	Study limitations, Serious Imprecision
Clopenthixol vs. Fluphenazine	indirect	very low	Study limitations, Serious Imprecision
Clopenthixol vs. Haloperidol	mixed	very low	Study limitations, Serious Imprecision
Clopenthixol vs. Iloperidone	indirect	very low	Study limitations, Serious Imprecision
Clopenthixol vs. Levomepromazine	indirect	low	Serious Imprecision
Clopenthixol vs. Loxapine	indirect	very low	Study limitations, Serious Imprecision
Clopenthixol vs. Lurasidone	indirect	low	Serious Imprecision
Clopenthixol vs. Molindone	indirect	very low	Study limitations, Serious Imprecision
Clopenthixol vs. Olanzapine	indirect	very low	Study limitations, Serious Imprecision
Clopenthixol vs. Paliperidone	indirect	low	Serious Imprecision
Clopenthixol vs. Penfluridol	indirect	very low	Study limitations, Serious Imprecision
Clopenthixol vs. Perazine	indirect	very low	Study limitations, Serious Imprecision
Clopenthixol vs. Perphenazine	mixed	low	Serious Imprecision
Clopenthixol vs. Pimozide	indirect	very low	Study limitations, Serious Imprecision
Clopenthixol vs. Placebo	mixed	very low	Study limitations, Publication Bias, Imprecision, Serious Incoherence
Clopenthixol vs. Quetiapine	indirect	very low	Study limitations, Serious Imprecision
Clopenthixol vs. Risperidone	indirect	very low	Study limitations, Serious Imprecision
Clopenthixol vs. Sertindole	indirect	very low	Study limitations, Serious Imprecision

Comparison	Nature of evidence	Confidence level	Downgrading
Clopenthixol vs. Sulpiride	indirect	very low	Study limitations, Serious Imprecision
Clopenthixol vs. Thioridazine	indirect	very low	Study limitations, Serious Imprecision
Clopenthixol vs. Thiothixene	indirect	very low	Study limitations, Serious Imprecision
Clopenthixol vs. Trifluoperazine	indirect	very low	Study limitations, Serious Imprecision
Clopenthixol vs. Ziprasidone	indirect	very low	Study limitations, Serious Imprecision
Clopenthixol vs. Zotepine	indirect	low	Serious Imprecision
Clopenthixol vs. Zuclopenthixol	indirect	very low	Study limitations, Serious Imprecision
Clozapine vs. Flupentixol	indirect	low	Study limitations, Imprecision
Clozapine vs. Fluphenazine	indirect	moderate	Study limitations
Clozapine vs. Haloperidol	mixed	low	Study limitations, Incoherence
Clozapine vs. Iloperidone	indirect	moderate	Study limitations
Clozapine vs. Levomepromazine	indirect	high	
Clozapine vs. Loxapine	indirect	low	Study limitations, Heterogeneity
Clozapine vs. Lurasidone	indirect	high	
Clozapine vs. Molindone	indirect	low	Study limitations, Imprecision
Clozapine vs. Olanzapine	mixed	very low	Study limitations, Heterogeneity, Serious Incoherence
Clozapine vs. Paliperidone	indirect	low	Study limitations, Heterogeneity
Clozapine vs. Penfluridol	indirect	very low	Study limitations, Serious Imprecision
Clozapine vs. Perazine	indirect	low	Study limitations, Imprecision
Clozapine vs. Perphenazine	indirect	moderate	Imprecision
Clozapine vs. Pimozide	indirect	low	Study limitations, Imprecision
Clozapine vs. Placebo	mixed	low	Study limitations, Publication Bias

Comparison	Nature of evidence	Confidence level	Downgrading
Clozapine vs. Quetiapine	indirect	moderate	Study limitations
Clozapine vs. Risperidone	mixed	very low	Study limitations, Heterogeneity, Serious Incoherence
Clozapine vs. Sertindole	indirect	moderate	Study limitations
Clozapine vs. Sulpiride	indirect	very low	Study limitations, Imprecision, Heterogeneity
Clozapine vs. Thioridazine	indirect	low	Study limitations, Imprecision
Clozapine vs. Thiothixene	indirect	very low	Study limitations, Serious Imprecision
Clozapine vs. Trifluoperazine	indirect	moderate	Study limitations
Clozapine vs. Ziprasidone	indirect	moderate	Study limitations
Clozapine vs. Zotepine	mixed	low	Imprecision, Incoherence
Clozapine vs. Zuclopenthixol	indirect	low	Study limitations, Imprecision
Flupentixol vs. Fluphenazine	indirect	very low	Study limitations, Serious Imprecision
Flupentixol vs. Haloperidol	indirect	very low	Study limitations, Serious Imprecision
Flupentixol vs. Iloperidone	indirect	low	Serious Imprecision
Flupentixol vs. Levomepromazine	indirect	low	Serious Imprecision
Flupentixol vs. Loxapine	indirect	very low	Study limitations, Serious Imprecision
Flupentixol vs. Lurasidone	indirect	low	Serious Imprecision
Flupentixol vs. Molindone	indirect	very low	Study limitations, Serious Imprecision
Flupentixol vs. Olanzapine	mixed	very low	Study limitations, Serious Imprecision
Flupentixol vs. Paliperidone	indirect	low	Serious Imprecision
Flupentixol vs. Penfluridol	indirect	very low	Study limitations, Serious Imprecision
Flupentixol vs. Perazine	indirect	very low	Study limitations, Serious Imprecision
Flupentixol vs. Perphenazine	indirect	low	Serious Imprecision

Comparison	Nature of evidence	Confidence level	Downgrading
Flupentixol vs. Pimozide	indirect	very low	Study limitations, Serious Imprecision
Flupentixol vs. Placebo	mixed	low	Publication Bias, Imprecision
Flupentixol vs. Quetiapine	indirect	low	Serious Imprecision
Flupentixol vs. Risperidone	indirect	low	Serious Imprecision
Flupentixol vs. Sertindole	indirect	low	Serious Imprecision
Flupentixol vs. Sulpiride	indirect	very low	Study limitations, Serious Imprecision
Flupentixol vs. Thioridazine	indirect	very low	Study limitations, Serious Imprecision
Flupentixol vs. Thiothixene	indirect	very low	Study limitations, Serious Imprecision
Flupentixol vs. Trifluoperazine	indirect	very low	Study limitations, Serious Imprecision
Flupentixol vs. Ziprasidone	indirect	very low	Study limitations, Serious Imprecision
Flupentixol vs. Zotepine	indirect	low	Serious Imprecision
Flupentixol vs. Zuclopenthixol	indirect	very low	Study limitations, Serious Imprecision
Fluphenazine vs. Haloperidol	mixed	very low	Study limitations, Serious Imprecision
Fluphenazine vs. Iloperidone	indirect	very low	Study limitations, Serious Imprecision
Fluphenazine vs. Levomepromazine	indirect	low	Serious Imprecision
Fluphenazine vs. Loxapine	indirect	very low	Study limitations, Serious Imprecision
Fluphenazine vs. Lurasidone	indirect	low	Serious Imprecision
Fluphenazine vs. Molindone	indirect	very low	Study limitations, Serious Imprecision
Fluphenazine vs. Olanzapine	indirect	very low	Study limitations, Imprecision, Heterogeneity
Fluphenazine vs. Paliperidone	indirect	very low	Study limitations, Serious Imprecision
Fluphenazine vs. Penfluridol	indirect	very low	Study limitations, Serious Imprecision
Fluphenazine vs. Perazine	indirect	very low	Study limitations, Serious Imprecision

Comparison	Nature of evidence	Confidence level	Downgrading
Fluphenazine vs. Perphenazine	indirect	low	Serious Imprecision
Fluphenazine vs. Pimozide	mixed	very low	Study limitations, Serious Imprecision
Fluphenazine vs. Placebo	mixed	very low	Study limitations, Publication Bias, Serious Imprecision
Fluphenazine vs. Quetiapine	indirect	very low	Study limitations, Serious Imprecision
Fluphenazine vs. Risperidone	indirect	very low	Study limitations, Imprecision, Heterogeneity
Fluphenazine vs. Sertindole	indirect	very low	Study limitations, Serious Imprecision
Fluphenazine vs. Sulpiride	indirect	very low	Study limitations, Serious Imprecision
Fluphenazine vs. Thioridazine	mixed	very low	Study limitations, Serious Imprecision
Fluphenazine vs. Thiothixene	indirect	very low	Study limitations, Serious Imprecision
Fluphenazine vs. Trifluoperazine	indirect	very low	Study limitations, Serious Imprecision
Fluphenazine vs. Ziprasidone	indirect	very low	Study limitations, Serious Imprecision
Fluphenazine vs. Zotepine	indirect	low	Imprecision, Heterogeneity
Fluphenazine vs. Zuclopenthixol	indirect	very low	Study limitations, Serious Imprecision
Haloperidol vs. Iloperidone	mixed	very low	Study limitations, Imprecision, Heterogeneity
Haloperidol vs. Levomepromazine	mixed	very low	Serious Imprecision, Serious Incoherence
Haloperidol vs. Loxapine	mixed	very low	Study limitations, Serious Imprecision, Incoherence
Haloperidol vs. Lurasidone	mixed	low	Imprecision, Heterogeneity
Haloperidol vs. Molindone	indirect	very low	Study limitations, Serious Imprecision
Haloperidol vs. Olanzapine	mixed	very low	Study limitations, Imprecision, Heterogeneity
Haloperidol vs. Paliperidone	indirect	low	Serious Imprecision
Haloperidol vs. Penfluridol	indirect	very low	Study limitations, Serious Imprecision
Haloperidol vs. Perazine	indirect	low	Serious Imprecision

Comparison	Nature of evidence	Confidence level	Downgrading
Haloperidol vs. Perphenazine	indirect	low	Serious Imprecision
Haloperidol vs. Pimozide	mixed	very low	Study limitations, Serious Imprecision
Haloperidol vs. Placebo	mixed	moderate	Study limitations
Haloperidol vs. Quetiapine	mixed	very low	Study limitations, Imprecision, Heterogeneity, Incoherence
Haloperidol vs. Risperidone	mixed	low	Imprecision, Heterogeneity
Haloperidol vs. Sertindole	mixed	very low	Imprecision, Heterogeneity, Incoherence
Haloperidol vs. Sulpiride	indirect	very low	Study limitations, Serious Imprecision
Haloperidol vs. Thioridazine	mixed	very low	Study limitations, Serious Imprecision
Haloperidol vs. Thiothixene	indirect	very low	Study limitations, Serious Imprecision
Haloperidol vs. Trifluoperazine	mixed	very low	Study limitations, Imprecision, Heterogeneity, Incoherence
Haloperidol vs. Ziprasidone	mixed	very low	Study limitations, Imprecision, Heterogeneity, Incoherence
Haloperidol vs. Zotepine	mixed	very low	Imprecision, Heterogeneity, Serious Incoherence
Haloperidol vs. Zuclopenthixol	mixed	very low	Study limitations, Serious Imprecision
Iloperidone vs. Levomepromazine	indirect	low	Serious Imprecision
Iloperidone vs. Loxapine	indirect	very low	Study limitations, Serious Imprecision
Iloperidone vs. Lurasidone	indirect	low	Serious Imprecision
Iloperidone vs. Molindone	indirect	very low	Study limitations, Serious Imprecision
Iloperidone vs. Olanzapine	indirect	low	Study limitations, Heterogeneity
Iloperidone vs. Paliperidone	indirect	low	Imprecision, Heterogeneity
Iloperidone vs. Penfluridol	indirect	very low	Study limitations, Serious Imprecision
Iloperidone vs. Perazine	indirect	low	Serious Imprecision
Iloperidone vs. Perphenazine	indirect	moderate	Imprecision

Comparison	Nature of evidence	Confidence level	Downgrading
Iloperidone vs. Pimozide	indirect	very low	Study limitations, Serious Imprecision
Iloperidone vs. Placebo	mixed	moderate	Publication Bias
Iloperidone vs. Quetiapine	indirect	low	Imprecision, Heterogeneity
Iloperidone vs. Risperidone	mixed	moderate	Imprecision
Iloperidone vs. Sertindole	indirect	low	Serious Imprecision
Iloperidone vs. Sulpiride	indirect	very low	Study limitations, Serious Imprecision
Iloperidone vs. Thioridazine	indirect	very low	Study limitations, Imprecision, Heterogeneity
Iloperidone vs. Thiothixene	indirect	very low	Study limitations, Imprecision, Heterogeneity
Iloperidone vs. Trifluoperazine	indirect	very low	Study limitations, Serious Imprecision
Iloperidone vs. Ziprasidone	mixed	very low	Study limitations, Imprecision, Heterogeneity, Incoherence
Iloperidone vs. Zotepine	indirect	moderate	Imprecision
Iloperidone vs. Zuclopenthixol	indirect	very low	Study limitations, Imprecision, Heterogeneity
Levomepromazine vs. Loxapine	indirect	low	Serious Imprecision
Levomepromazine vs. Lurasidone	indirect	low	Serious Imprecision
Levomepromazine vs. Molindone	indirect	low	Serious Imprecision
Levomepromazine vs. Olanzapine	indirect	low	Imprecision, Heterogeneity
Levomepromazine vs. Paliperidone	indirect	low	Serious Imprecision
Levomepromazine vs. Penfluridol	indirect	very low	Study limitations, Serious Imprecision
Levomepromazine vs. Perazine	indirect	low	Serious Imprecision

Comparison	Nature of evidence	Confidence level	Downgrading
Levomepromazine vs. Perphenazine	indirect	low	Imprecision, Heterogeneity
Levomepromazine vs. Pimozide	indirect	very low	Study limitations, Serious Imprecision
Levomepromazine vs. Placebo	indirect	low	Publication Bias, Imprecision
Levomepromazine vs. Quetiapine	indirect	low	Serious Imprecision
Levomepromazine vs. Risperidone	mixed	very low	Imprecision, Heterogeneity, Serious Incoherence
Levomepromazine vs. Sertindole	indirect	low	Serious Imprecision
Levomepromazine vs. Sulpiride	indirect	low	Serious Imprecision
Levomepromazine vs. Thioridazine	indirect	low	Serious Imprecision
Levomepromazine vs. Thiothixene	indirect	very low	Study limitations, Serious Imprecision
Levomepromazine vs. Trifluoperazine	indirect	low	Serious Imprecision
Levomepromazine vs. Ziprasidone	indirect	low	Serious Imprecision
Levomepromazine vs. Zotepine	indirect	low	Imprecision, Heterogeneity
Levomepromazine vs. Zuclopenthixol	indirect	low	Serious Imprecision
Loxapine vs. Lurasidone	indirect	low	Serious Imprecision
Loxapine vs. Molindone	indirect	very low	Study limitations, Serious Imprecision
Loxapine vs. Olanzapine	indirect	very low	Study limitations, Imprecision, Heterogeneity

Comparison	Nature of evidence	Confidence level	Downgrading
Loxapine vs. Paliperidone	indirect	very low	Study limitations, Serious Imprecision
Loxapine vs. Penfluridol	indirect	very low	Study limitations, Serious Imprecision
Loxapine vs. Perazine	indirect	very low	Study limitations, Serious Imprecision
Loxapine vs. Perphenazine	indirect	low	Serious Imprecision
Loxapine vs. Pimozide	indirect	very low	Study limitations, Serious Imprecision
Loxapine vs. Placebo	mixed	low	Study limitations, Publication Bias
Loxapine vs. Quetiapine	indirect	very low	Study limitations, Serious Imprecision
Loxapine vs. Risperidone	indirect	very low	Study limitations, Imprecision, Heterogeneity
Loxapine vs. Sertindole	indirect	very low	Study limitations, Serious Imprecision
Loxapine vs. Sulpiride	indirect	very low	Study limitations, Serious Imprecision
Loxapine vs. Thioridazine	mixed	very low	Study limitations, Serious Imprecision
Loxapine vs. Thiothixene	mixed	very low	Study limitations, Serious Imprecision, Incoherence
Loxapine vs. Trifluoperazine	mixed	very low	Study limitations, Imprecision, Heterogeneity, Incoherence
Loxapine vs. Ziprasidone	indirect	very low	Study limitations, Serious Imprecision
Loxapine vs. Zotepine	indirect	low	Serious Imprecision
Loxapine vs. Zuclopenthixol	indirect	very low	Study limitations, Serious Imprecision
Lurasidone vs. Molindone	indirect	low	Serious Imprecision
Lurasidone vs. Olanzapine	mixed	low	Imprecision, Incoherence
Lurasidone vs. Paliperidone	indirect	low	Imprecision, Heterogeneity
Lurasidone vs. Penfluridol	indirect	very low	Study limitations, Serious Imprecision
Lurasidone vs. Perazine	indirect	low	Serious Imprecision
Lurasidone vs. Perphenazine	indirect	low	Imprecision, Heterogeneity

Comparison	Nature of evidence	Confidence level	Downgrading
Lurasidone vs. Pimozide	indirect	very low	Study limitations, Serious Imprecision
Lurasidone vs. Placebo	mixed	low	Publication Bias, Incoherence
Lurasidone vs. Quetiapine	mixed	low	Imprecision, Heterogeneity
Lurasidone vs. Risperidone	indirect	moderate	Imprecision
Lurasidone vs. Sertindole	indirect	low	Serious Imprecision
Lurasidone vs. Sulpiride	indirect	low	Serious Imprecision
Lurasidone vs. Thioridazine	indirect	low	Serious Imprecision
Lurasidone vs. Thiothixene	indirect	very low	Study limitations, Serious Imprecision
Lurasidone vs. Trifluoperazine	indirect	low	Serious Imprecision
Lurasidone vs. Ziprasidone	indirect	low	Serious Imprecision
Lurasidone vs. Zotepine	indirect	low	Imprecision, Heterogeneity
Lurasidone vs. Zuclopenthixol	indirect	low	Serious Imprecision
Molindone vs. Olanzapine	indirect	very low	Study limitations, Serious Imprecision
Molindone vs. Paliperidone	indirect	low	Serious Imprecision
Molindone vs. Penfluridol	indirect	very low	Study limitations, Serious Imprecision
Molindone vs. Perazine	indirect	very low	Study limitations, Serious Imprecision
Molindone vs. Perphenazine	indirect	low	Serious Imprecision
Molindone vs. Pimozide	indirect	very low	Study limitations, Serious Imprecision
Molindone vs. Placebo	mixed	very low	Study limitations, Publication Bias, Imprecision
Molindone vs. Quetiapine	indirect	very low	Study limitations, Serious Imprecision
Molindone vs. Risperidone	indirect	very low	Study limitations, Serious Imprecision
Molindone vs. Sertindole	indirect	very low	Study limitations, Serious Imprecision

Comparison	Nature of evidence	Confidence level	Downgrading
Molindone vs. Sulpiride	indirect	very low	Study limitations, Serious Imprecision
Molindone vs. Thioridazine	indirect	very low	Study limitations, Serious Imprecision
Molindone vs. Thiothixene	indirect	very low	Study limitations, Serious Imprecision
Molindone vs. Trifluoperazine	mixed	very low	Study limitations, Serious Imprecision
Molindone vs. Ziprasidone	indirect	very low	Study limitations, Serious Imprecision
Molindone vs. Zotepine	indirect	low	Serious Imprecision
Molindone vs. Zuclopenthixol	indirect	very low	Study limitations, Serious Imprecision
Olanzapine vs. Paliperidone	mixed	low	Imprecision, Heterogeneity
Olanzapine vs. Penfluridol	indirect	very low	Study limitations, Serious Imprecision
Olanzapine vs. Perazine	indirect	low	Serious Imprecision
Olanzapine vs. Perphenazine	mixed	low	Serious Imprecision
Olanzapine vs. Pimozide	indirect	very low	Study limitations, Serious Imprecision
Olanzapine vs. Placebo	mixed	moderate	Study limitations
Olanzapine vs. Quetiapine	mixed	low	Imprecision, Heterogeneity
Olanzapine vs. Risperidone	mixed	very low	Serious Heterogeneity, Serious Incoherence
Olanzapine vs. Sertindole	mixed	low	Imprecision, Heterogeneity
Olanzapine vs. Sulpiride	indirect	very low	Study limitations, Serious Imprecision
Olanzapine vs. Thioridazine	indirect	very low	Study limitations, Serious Imprecision
Olanzapine vs. Thiothixene	indirect	very low	Study limitations, Serious Imprecision
Olanzapine vs. Trifluoperazine	indirect	low	Study limitations, Imprecision
Olanzapine vs. Ziprasidone	mixed	very low	Study limitations, Imprecision, Heterogeneity
Olanzapine vs. Zotepine	indirect	low	Serious Imprecision

Comparison	Nature of evidence	Confidence level	Downgrading
Olanzapine vs. Zuclopenthixol	indirect	very low	Study limitations, Serious Imprecision
Paliperidone vs. Penfluridol	indirect	very low	Study limitations, Serious Imprecision
Paliperidone vs. Perazine	indirect	low	Serious Imprecision
Paliperidone vs. Perphenazine	indirect	low	Serious Imprecision
Paliperidone vs. Pimozide	indirect	very low	Study limitations, Serious Imprecision
Paliperidone vs. Placebo	mixed	high	
Paliperidone vs. Quetiapine	indirect	low	Imprecision, Heterogeneity
Paliperidone vs. Risperidone	indirect	low	Imprecision, Heterogeneity
Paliperidone vs. Sertindole	indirect	low	Imprecision, Heterogeneity
Paliperidone vs. Sulpiride	indirect	low	Serious Imprecision
Paliperidone vs. Thioridazine	indirect	very low	Study limitations, Serious Imprecision
Paliperidone vs. Thiothixene	indirect	very low	Study limitations, Serious Imprecision
Paliperidone vs. Trifluoperazine	indirect	very low	Study limitations, Imprecision, Heterogeneity
Paliperidone vs. Ziprasidone	indirect	low	Imprecision, Heterogeneity
Paliperidone vs. Zotepine	indirect	low	Serious Imprecision
Paliperidone vs. Zuclopenthixol	indirect	low	Serious Imprecision
Penfluridol vs. Perazine	indirect	very low	Study limitations, Serious Imprecision
Penfluridol vs. Perphenazine	indirect	very low	Study limitations, Serious Imprecision
Penfluridol vs. Pimozide	indirect	very low	Study limitations, Serious Imprecision
Penfluridol vs. Placebo	indirect	very low	Study limitations, Publication Bias, Serious Imprecision
Penfluridol vs. Quetiapine	indirect	very low	Study limitations, Serious Imprecision
Penfluridol vs. Risperidone	indirect	very low	Study limitations, Serious Imprecision

Comparison	Nature of evidence	Confidence level	Downgrading
Penfluridol vs. Sertindole	indirect	very low	Study limitations, Serious Imprecision
Penfluridol vs. Sulpiride	indirect	very low	Study limitations, Serious Imprecision
Penfluridol vs. Thioridazine	indirect	very low	Study limitations, Serious Imprecision
Penfluridol vs. Thiothixene	indirect	very low	Study limitations, Serious Imprecision
Penfluridol vs. Trifluoperazine	indirect	very low	Study limitations, Serious Imprecision
Penfluridol vs. Ziprasidone	indirect	very low	Study limitations, Serious Imprecision
Penfluridol vs. Zotepine	indirect	very low	Study limitations, Serious Imprecision
Penfluridol vs. Zuclopenthixol	indirect	very low	Study limitations, Serious Imprecision
Perazine vs. Perphenazine	indirect	low	Serious Imprecision
Perazine vs. Pimozide	indirect	very low	Study limitations, Serious Imprecision
Perazine vs. Placebo	indirect	low	Publication Bias, Imprecision
Perazine vs. Quetiapine	indirect	low	Serious Imprecision
Perazine vs. Risperidone	indirect	low	Serious Imprecision
Perazine vs. Sertindole	indirect	low	Serious Imprecision
Perazine vs. Sulpiride	indirect	low	Serious Imprecision
Perazine vs. Thioridazine	indirect	very low	Study limitations, Serious Imprecision
Perazine vs. Thiothixene	indirect	very low	Study limitations, Serious Imprecision
Perazine vs. Trifluoperazine	indirect	very low	Study limitations, Serious Imprecision
Perazine vs. Ziprasidone	indirect	low	Serious Imprecision
Perazine vs. Zotepine	mixed	very low	Study limitations, Serious Imprecision
Perazine vs. Zuclopenthixol	indirect	low	Serious Imprecision
Perphenazine vs. Pimozide	indirect	very low	Study limitations, Serious Imprecision

Comparison	Nature of evidence	Confidence level	Downgrading
Perphenazine vs. Placebo	mixed	moderate	Publication Bias
Perphenazine vs. Quetiapine	mixed	very low	Imprecision, Heterogeneity, Incoherence
Perphenazine vs. Risperidone	mixed	low	Serious Imprecision
Perphenazine vs. Sertindole	indirect	low	Imprecision, Heterogeneity
Perphenazine vs. Sulpiride	indirect	low	Serious Imprecision
Perphenazine vs. Thioridazine	indirect	very low	Study limitations, Serious Imprecision
Perphenazine vs. Thiothixene	indirect	very low	Study limitations, Serious Imprecision
Perphenazine vs. Trifluoperazine	indirect	very low	Study limitations, Imprecision, Heterogeneity
Perphenazine vs. Ziprasidone	mixed	low	Imprecision, Heterogeneity
Perphenazine vs. Zotepine	indirect	low	Serious Imprecision
Perphenazine vs. Zuclopenthixol	mixed	very low	Study limitations, Serious Imprecision
Pimozide vs. Placebo	indirect	very low	Study limitations, Publication Bias, Serious Imprecision
Pimozide vs. Quetiapine	indirect	very low	Study limitations, Serious Imprecision
Pimozide vs. Risperidone	indirect	very low	Study limitations, Serious Imprecision
Pimozide vs. Sertindole	indirect	very low	Study limitations, Serious Imprecision
Pimozide vs. Sulpiride	indirect	very low	Study limitations, Serious Imprecision
Pimozide vs. Thioridazine	indirect	very low	Study limitations, Serious Imprecision
Pimozide vs. Thiothixene	indirect	very low	Study limitations, Serious Imprecision
Pimozide vs. Trifluoperazine	indirect	very low	Study limitations, Serious Imprecision
Pimozide vs. Ziprasidone	indirect	very low	Study limitations, Serious Imprecision
Pimozide vs. Zotepine	indirect	very low	Study limitations, Serious Imprecision
Pimozide vs. Zuclopenthixol	indirect	very low	Study limitations, Serious Imprecision

Comparison	Nature of evidence	Confidence level	Downgrading
Placebo vs. Quetiapine	mixed	moderate	Publication Bias
Placebo vs. Risperidone	mixed	high	
Placebo vs. Sertindole	mixed	low	Study limitations, Publication Bias
Placebo vs. Sulpiride	indirect	low	Study limitations, Publication Bias
Placebo vs. Thioridazine	mixed	very low	Study limitations, Publication Bias, Incoherence
Placebo vs. Thiothixene	mixed	very low	Study limitations, Publication Bias, Serious Incoherence
Placebo vs. Trifluoperazine	mixed	very low	Study limitations, Publication Bias, Imprecision, Heterogeneity
Placebo vs. Ziprasidone	mixed	low	Study limitations, Publication Bias
Placebo vs. Zotepine	mixed	moderate	Publication Bias
Placebo vs. Zuclopenthixol	indirect	low	Study limitations, Publication Bias
Quetiapine vs. Risperidone	mixed	low	Imprecision, Heterogeneity
Quetiapine vs. Sertindole	indirect	low	Serious Imprecision
Quetiapine vs. Sulpiride	indirect	very low	Study limitations, Serious Imprecision
Quetiapine vs. Thioridazine	indirect	very low	Study limitations, Serious Imprecision
Quetiapine vs. Thiothixene	indirect	very low	Study limitations, Serious Imprecision
Quetiapine vs. Trifluoperazine	indirect	very low	Study limitations, Serious Imprecision
Quetiapine vs. Ziprasidone	mixed	low	Serious Imprecision
Quetiapine vs. Zotepine	indirect	low	Imprecision, Heterogeneity
Quetiapine vs. Zuclopenthixol	indirect	very low	Study limitations, Serious Imprecision
Risperidone vs. Sertindole	mixed	very low	Imprecision, Heterogeneity, Incoherence
Risperidone vs. Sulpiride	indirect	very low	Study limitations, Serious Imprecision
Risperidone vs. Thioridazine	indirect	very low	Study limitations, Serious Imprecision

Comparison	Nature of evidence	Confidence level	Downgrading
Risperidone vs. Thiothixene	indirect	very low	Study limitations, Serious Imprecision
Risperidone vs. Trifluoperazine	indirect	low	Study limitations, Imprecision
Risperidone vs. Ziprasidone	mixed	very low	Imprecision, Heterogeneity, Incoherence
Risperidone vs. Zotepine	indirect	low	Serious Imprecision
Risperidone vs. Zuclopenthixol	mixed	very low	Study limitations, Serious Imprecision
Sertindole vs. Sulpiride	indirect	very low	Study limitations, Serious Imprecision
Sertindole vs. Thioridazine	indirect	very low	Study limitations, Serious Imprecision
Sertindole vs. Thiothixene	indirect	very low	Study limitations, Serious Imprecision
Sertindole vs. Trifluoperazine	indirect	very low	Study limitations, Serious Imprecision
Sertindole vs. Ziprasidone	indirect	very low	Study limitations, Serious Imprecision
Sertindole vs. Zotepine	indirect	low	Imprecision, Heterogeneity
Sertindole vs. Zuclopenthixol	indirect	very low	Study limitations, Serious Imprecision
Sulpiride vs. Thioridazine	mixed	very low	Study limitations, Serious Imprecision
Sulpiride vs. Thiothixene	indirect	very low	Study limitations, Serious Imprecision
Sulpiride vs. Trifluoperazine	mixed	very low	Study limitations, Serious Imprecision
Sulpiride vs. Ziprasidone	indirect	very low	Study limitations, Serious Imprecision
Sulpiride vs. Zotepine	indirect	low	Serious Imprecision
Sulpiride vs. Zuclopenthixol	indirect	low	Serious Imprecision
Thioridazine vs. Thiothixene	mixed	very low	Study limitations, Serious Imprecision, Serious Incoherence
Thioridazine vs. Trifluoperazine	indirect	very low	Study limitations, Imprecision, Heterogeneity
Thioridazine vs. Ziprasidone	indirect	very low	Study limitations, Serious Imprecision
Thioridazine vs. Zotepine	indirect	very low	Study limitations, Serious Imprecision

Comparison	Nature of evidence	Confidence level	Downgrading
Thioridazine vs. Zuclopenthixol	indirect	very low	Study limitations, Serious Imprecision
Thiothixene vs. Trifluoperazine	indirect	very low	Study limitations, Imprecision, Heterogeneity
Thiothixene vs. Ziprasidone	indirect	very low	Study limitations, Serious Imprecision
Thiothixene vs. Zotepine	indirect	very low	Study limitations, Serious Imprecision
Thiothixene vs. Zuclopenthixol	indirect	very low	Study limitations, Serious Imprecision
Trifluoperazine vs. Ziprasidone	indirect	very low	Study limitations, Serious Imprecision
Trifluoperazine vs. Zotepine	indirect	moderate	Imprecision
Trifluoperazine vs. Zuclopenthixol	indirect	very low	Study limitations, Serious Imprecision
Ziprasidone vs. Zotepine	indirect	low	Imprecision, Heterogeneity
Ziprasidone vs. Zuclopenthixol	indirect	very low	Study limitations, Serious Imprecision
Zotepine vs. Zuclopenthixol	indirect	low	Serious Imprecision

Tabelle 15.3: CINeMA ratings for all comparisons of the primary outcome

In this table we present the evidence levels for all comparisons of the primary outcome "overall change in symptoms". Comparison: names of the antipsychotics compared. Nature of evidence: mixed=combination of direct and indirect evidence, indirect=only indirect evidence available. Confidence level: high, moderate, low, very low. Downgrading: CINeMA items responsible for downgrading. CINeMA (Confidence in Network Meta-analysis).

References

1 Higgins JPT, editor. Cochrane handbook for systematic reviews of interventions, Reprinted. Chichester: Wiley-Blackwell, 2011.

2 Furukawa TA, Barbui C, Cipriani A, Brambilla P, Watanabe N. Imputing missing standard deviations in meta-analyses can provide accurate results. *Journal of clinical epidemiology* 2006; **59**: 7–10.

3 Rhodes KM, Turner RM, Higgins JPT. Empirical evidence about inconsistency among studies in a pair-wise meta-analysis. *Research synthesis methods* 2016; **7**: 346–70.

4 Leucht S, Leucht C, Huhn M *et al.* Sixty years of placebo-controlled antipsychotic drug trials in acute schizophrenia: Systematic review, bayesian meta-analysis, and meta-regression of efficacy predictors. *The American journal of psychiatry* 2017;: appiajp201716121358.

Appendix 16: Definition of covariates for metagressions

Placebo response rate

We extracted the change of overall schizophrenia symptoms in the placebo arms. If only baseline and endpoint values were given, we calculated the change value using these numbers. We used the "Positive and Negative Syndrome Scale for Schizophrenia" (PANSS) [9] with a range of 1-7. If the study used a range of 0-6 we transformed the score by adding 30 points corresponding to one point for each item. In case of the "Brief Psychiatric Rating Scale" (BPRS) [10], we transformed the values to total PANSS score using the linking tables published by Leucht et al. [5]

Study sample size

We extracted the sample sizes of the individual drug arms. If different numbers for different samples (e.g. efficacy sample and safety sample) were given, we always used the number randomized and not the number analyzed.

Publication year

We extracted the year of the first available paper publication of the study.

Baseline severity

We extracted the baseline severity of the overall schizophrenia symptom scale. We used the "Positive and Negative Syndrome Scale for Schizophrenia" (PANSS) [9] with a range of 1-7. If the study used a range of 0-6 we transformed the score by adding 30 points corresponding to one point for each item. In case of the "Brief Psychiatric Rating Scale" (BPRS) [10], we transformed the values to total PANSS score using the linking tables published by Leucht et al. [5]

Sponsorship

Sponsorship was rated for each study arm individually. If the study was sponsored by the company marketing the drug in question we rated the individual arm as sponsored. In case the company only provided the drug, but did not give further funding we rated the individual arm as "not sponsored". If there was no clear information for "sponsoring" or "not sponsoring" we rated "not indicated". We compared sponsored with "not sponsored/unclear".

Mean age

We extracted the mean age for each drug arm individually. If this parameter was not presented we rated as "not indicated".

Percentage male

We extracted the number randomized and the number of men in each arm individually and calculated the percentage. If the percentage men was not indicated, we rated as "not indicated".

Appendix 17: Definition of sensitivity analyses

Duration

We excluded studies with a duration less than four or more than eight weeks of duration.

Studies with high risk of bias

We excluded studies with a high risk of bias. High of bias was defined using the criteria of Cipriani et al [11]: At first two independent reviewers assessed RoB of each study for the primary outcome of efficacy in the following domains: sequence generation, allocation concealment, blinding of participant, blinding of therapist, blinding of assessor, missing outcomes, selective reporting and other bias. Then the overall RoB of each study was calculated as follows: Studies were classified as having low risk of bias if none of the domains above was rated as high risk of bias and three or less were rated as unclear risk; moderate if one was rated as high risk of bias or none was rated as high risk of bias but four or more were rated as unclear risk, and all other cases were assumed to pertain to high risk of bias.

Studies with imputed standard deviation

In case standard deviations were not given by the study authors, we re-calculated them using appropriate formulas [2]. We excluded these studies with imputed standard deviations for this subgroup analysis.

Placebo

To examine the possible influence of placebo controls we performed the following two sensitivity analyses (“head-to-head studies” and “only active arms”):

a) Head to head studies

We excluded placebo-controlled trials from the data set. Studies with placebo arms were excluded altogether even if they had two or more active arms.

b) Only active arms

We excluded the placebo arms, but kept the remaining active arms of the studies. Two arm placebo controlled studies were excluded.

Studies using intent-to-treat-analysis

We examined only studies that used intent-to-treat analysis and excluded studies that used completer data. In case of doubt or if the type of data analysis was not clearly stated, we excluded the study in this subgroup analysis.

Only fair doses

We calculated the mean olanzapine equivalence doses for each drug arm according to the “International Consensus Study on Antipsychotic dose” [6]. For this analysis we excluded studies in which the mean dose of one drug was 50% less than the mean dose of the other in olanzapine equivalents. For example, if a study compared haloperidol 2mg/day (olanzapine equivalence dose =4mg) with olanzapine 10mg/day we excluded the study.

Older Studies

We assumed that older studies are different concerning patient charateristics and study quality. To check if this influenced the results considerably we excluded studies published before 1990 folowwing a reviewer request.

Failed trials

Three (or more) arm trials (new antipsychotic versus versus placebo and versus an already licensed antipsychotic as an active comparator) in which neither the test drug nor the active comparator was significantly superior to placebo are called "failed trials". Such trials were excluded in a sensitivity analysis following a reviewer request.

Appendix 18: Description of outcome parameters

Outcome	Description	subjective/ objective
Change in overall symptoms	We extracted scales that measured overall symptoms of schizophrenia with the following hierarchy: PANSS total change, BPRS total change, PANSS endpoint, BPRS endpoint and other published rating scale of overall symptoms of schizophrenia.	subjective
Positive symptoms	We extracted scales or subscales that measured positive symptoms of schizophrenia with the following hierarchy: PANSS positive change, SAPS positive change, PANSS positive endpoint, SAPS positive endpoint, BPRS positive change/endpoint	subjective
Negative symptoms	We extracted scales or subscales that measured negative symptoms of schizophrenia with the following hierarchy: PANSS negative change, SANS change, PANSS negative endpoint, SANS endpoint, BPRS negative change/endpoint (CAVE, various terms for BPRS negative exist, e.g. anergia subscore).	subjective
Depressive symptoms	We extracted scales or subscales that measured depressive symptoms of schizophrenia with the following hierarchy: CDSS change, HAM-D change, MADRS change, then the values at endpoint of these rating scales, then PANSS depression change, BPRS depression and anxiety subscore change.<br><br>As an exception to the general rule of not taking single items we here use also the single item BPRS depressive mood.	subjective
Quality of life	We extracted scales that measured quality of line in schizophrenia. As quality of life scales have not been used much in schizophrenia trials, we did not define a hierarchy, but rather used what was used by the author.	subjective
Social functioning	We extracted scales that measured social functioning in schizophrenia. As functioning scales have not been used much in schizophrenia trials, it is difficult to predefine a hierarchy. But generally speaking more recent scales (e.g. PSP) were preferred to older ones (e.g. GAF).	subjective
All-cause discontinuation	Number of patients, who dropped out of the individual arm before end of study for whatever reason. We put “zero dropouts” only if it was clearly stated that there had been no dropouts.	subjective
Discontinuation due to inefficacy	Number of participants who dropped out of the individual arm before the end of a study due to inefficacy. We put “zero dropouts” only if it was clearly stated that there had been no dropouts due to inefficacy.	subjective
Weight gain continuous*	Mean weight gain or weight at endpoint in kilogram. If other units were given, they had been transformed to kg.	objective
Weight gain dichotomous	Patients who had ≥ 7% weight gain from baseline to endpoint.	objective
Use of antiparkinson medication	Number of patients who received antiparkinson medication at least once in the individual arm, e.g. biperiden, benztropin, procyclidine, “anticholinergic medication”.	subjective

Akathisia	Number of patients with akathisia. We only used the data, if the authors explicitly used the term “akathisia”. Other terms like “restlessness” were not accepted.	subjective
Prolactin elevation*	Mean prolactin change or mean prolactin level at endpoint in ng/ml. If other units were given, they had been transformed to ng/ml.	objective
QTc prolongation*	Mean change of QTc time in ms or mean QTc time at endpoint. If QTc was presented by both Bazett’s and Fridericia’s adjustment, we prefered Fridericia.	objective
Sedation	Number of patients who felt sedated. Terms used were “somnolence”, “tiredness”, “sleepiness”, “drowsiness”, “fatigue”.	subjective
Anticholinergic side effects	Number of patients with the following adverse effects: blurred vision, constipation, dry mouth or hyposalivation and urinary retention.	subjective
Response study defined	Number of patients who responded according the authors’ original definition of response	subjective

PANSS=Positive and negative syndrom scale for schizophrenia, SAPS=Scale for the assessment of positive symptoms, SANS=Scale for the assessment of negative symptoms, BPRS=Brief psychiatric rating scale, CDSS=Calgary depression scale for schizophrenia, MADRS=Montgomery-Asberg depression rating scale, HAM-D=Hamilton depression rating scale, PSP=Personal and social performance scale, GAF=Global assessment of functioning. *Outcome reported as MD (mean difference) to provide better clinical interpretability compared to standardized mean difference (SMD).

Appendix 19: Classification of individual risk of bias items

We classified the individual risk of bias items for each study using the criteria below. For the items "incomplete outcome data" and "selective reporting" we focused on the primary outcome.

Risk of bias item	Low risk	Unclear risk	High risk
Randomisation	The investigators describe a random component in the sequence generation process such as:<br><br>• Referring to a random number table;<br><br>• Using a computer random number generator;<br><br>• Coin tossing;<br><br>• Shuffling cards or envelopes;<br><br>• Throwing dice;<br><br>• Drawing of lots;<br><br>• Minimization*.	Insufficient information about the sequence generation process to permit judgement of 'Low risk' or 'High risk (usually studies called „randomised" without any further description)	• Sequence generated by odd or even date of birth;<br><br>• Sequence generated by some rule based on date (or day) of admission;<br><br>• Sequence generated by some rule based on hospital or clinic record number.<br><br>Other non-random approaches happen much less frequently than the systematic approaches mentioned above and tend to be obvious. They usually involve judgement or some method of non-random categorization of participants, for example:<br><br>• Allocation by judgement of the clinician;
Allocation concealment	• Central allocation (including telephone, web-based and pharmacy-controlled randomization);<br><br>• Sequentially numbered drug containers of identical appearance;<br><br>• Sequentially numbered, opaque, sealed envelopes.	Insufficient information to permit judgement of 'Low risk' or 'High risk'. This is usually the case if the method of concealment is not described or not described in sufficient detail to allow a definite judgement – for example if the use of assignment envelopes is described, but it remains unclear whether envelopes were sequentially numbered, opaque and sealed. Or if the study is just	Participants or investigators enrolling participants could possibly foresee assignments and thus introduce selection bias, such as allocation based on:<br><br>• Using an open random allocation schedule (e.g. a list of random numbers);<br><br>• Assignment envelopes were used without appropriate safeguards (e.g. if envelopes were unsealed or non-opaque or not sequentially numbered);<br><br>• Alternation or rotation;

Risk of bias item	Low risk	Unclear risk	High risk
		described as „randomised“ without any further description	• Date of birth;<br>• Case record number;<br>• Any other explicitly unconcealed procedure
Blinding of participants and personnel	• Double blind with at least some further detail (e.g. identical capsules would be sufficient)<br>• No blinding or incomplete blinding, but the review authors judge that the outcome is not likely to be influenced by lack of blinding (as we focus on efficacy, subjectively measured by rating scales, this is usually not the case, but if our primary outcome were death, such an objective, undisputable outcome would be less affected by blinding);<br>• Blinding of participants and key study personnel ensured, and unlikely that the blinding could have been broken.	• Insufficient information to permit judgement of 'Low risk' or 'High risk';<br>• The study did not address this outcome<br>• studies in which it was said that part of the patients received placebo capsules, without<br>mentioning the term blinding or masking<br>• only stated double blind, but not further details	• No blinding or incomplete blinding, and the outcome is likely to be influenced by lack of blinding;<br>• Blinding of key study participants and personnel attempted, but likely that the blinding could have been broken, and the outcome is likely to be influenced by lack of blinding (in our reviews we do not automatically assume that side-effects could break the blind, unless the study authors present evidence for this)
Blinding of outcome assessment	• To judge „rater blinded“ (single blind) studies to be free of bias, we need at least a few details (for example, raters came from another department), the statment „raters blinded to treatment“ is not enough<br>• Studies which were „double blind“ with a few more details how blinding was achieved (e.g.	• stated rater blind, but no further details such as „the raters were from another department“<br>• studies in which it was said that part of the patients received placebo capsules, without mentioning the term blinding or masking<br>• only stated double blind, but no further details are provided how	• No blinding of outcome assessment, and the outcome measurement is likely to be influenced<br>by lack of blinding;<br>• Blinding of outcome assessment, but likely that the blinding could have been broken, and the<br>outcome measurement is likely to be influenced by lack of blinding (in our reviews we do not

Risk of bias item	Low risk	Unclear risk	High risk
	„identical capsules" would be sufficient)<br><br>• No blinding of outcome assessment, but the review authors judge that the outcome<br><br>measurement is not likely to be influenced by lack of blinding (as we focus on efficacy, subjectively measured by rating scales, this is usually not the case, but if our primary outcome were death, such an objective, undisputable outcome would be less affected by blinding);<br><br>• Blinding of outcome assessment ensured, and unlikely that the blinding could have been broken (in our reviews we do not automatically assume that side-effects could break the blind,<br><br>unless the study authors present evidence for this).	blinding was made (e.g. not even the term „identical capsules" is used)<br><br>• Insufficient information to permit judgement of 'Low risk' or 'High risk';<br><br>• The study did not address this outcome.	automatically assume that side-effects could break the blind, unless the study authors present evidence for this).
Incomplete outcome data	• No missing outcome data;<br><br>• dropouts with reasons reported<br><br>• "reasonable" intention to treat analysis (e.g. at least one dose and one post baseline assessment)	• abstract or poster only<br><br>• Insufficient reporting of attrition/exclusions to permit judgement of 'Low risk' or 'High risk' (e.g.<br><br>number randomized not stated, dropouts or reasons for dropouts not indicated);	• only completer analysis (unless there were no dropouts)

Risk of bias item	Low risk	Unclear risk	High risk
Selective reporting	• The study protocol is available and all of the study's pre-specified (primary and secondary) outcomes that are of interest in the review have been reported in the pre-specified way; as we usually do not have the protocol, we will compare what is described in the method section with what is reported in the results.<br><br>• The study protocol is not available but it is clear that the published reports include all expected outcomes, including those that were pre-specified	• only abstract or poster<br><br>• Insufficient information to permit judgement of 'Low risk' or 'High risk'. It is likely that the majority of studies will fall into this category.	• Not all of the study's pre-specified primary outcomes have been reported;<br><br>• One or more primary outcomes is reported using measurements, analysis methods or subsets of the data (e.g. subscales) that were not pre-specified;<br><br>• One or more reported primary outcomes were not pre-specified (unless clear justification for their reporting is provided, such as an unexpected adverse effect);<br><br>• One or more outcomes of interest in the review are reported incompletely so that they cannot be entered in a meta-analysis;<br><br>• The study report fails to include results for a key outcome that would be expected to have been reported for such a study (IN OUR CASE USUALLY overall efficacy (continous or dichotomous responder) AND dropouts + dropout reasons inefficacy and adverse events)
Other bias	The study appears to be free of other sources of bias.	• Insufficient information to assess whether an important risk of bias exists (one reason could be that the study is only available as an abstract or poster); or<br><br>• Insufficient rationale or evidence that an identified problem will introduce bias.	• Statistically significant baseline imbalance in an important outcome (in our case primarily<br><br>overall efficacy)<br><br>• Premature termination of the study<br><br>• Study has been claimed to be fraudulent

References

1 Higgins JPT, Green S. Cochrane Handbook for Systematic Reviews of Interventions Version 5.1.0 [updated March 2011]. Chichester, UK: Wiley and Sons, 2011.

2 Furukawa TA, Barbui C, Cipriani A, Brambilla P, Watanabe N. Imputing missing standard deviations in meta-analyses can provide accurate results. *Journal of clinical epidemiology* 2006; **59:** 7–10. doi:10.1016/j.jclinepi.2005.06.006.

3 Plummer M, Stukalov A, Denwood M. Rjags: Bayesian Graphical Models using MCMC (version 4-6) 2016. https://CRAN.R-project.org/package=rjags.

4 Leucht S, Leucht C, Huhn M, et al. Sixty Years of Placebo-Controlled Antipsychotic Drug Trials in Acute Schizophrenia. Systematic Review, Bayesian Meta-Analysis, and Meta-Regression of Efficacy Predictors. *The American journal of psychiatry* 2017: appiajp201716121358. doi:10.1176/appi.ajp.2017.16121358.

5 Leucht S, Rothe P, Davis JM, Engel RR. Equipercentile linking of the BPRS and PANSS. *European Neuropsychopharmacology* 2012; **in press**.

6 Gardner DM, Murphy AL, O'Donnell H, Centorrino F, Baldessarini RJ. International consensus study of antipsychotic dosing. *The American journal of psychiatry* 2010; **167:** 686–93. doi:10.1176/appi.ajp.2009.09060802.

7 Rhodes KM, Turner RM, Higgins JPT. Predictive distributions were developed for the extent of heterogeneity in meta-analyses of continuous outcome data. *Journal of clinical epidemiology* 2015; **68:** 52–60. doi:10.1016/j.jclinepi.2014.08.012.

8 Gerta Rücker, Guido Schwarzer, Ulrike Krahn, Jochem König, Orestis Efthimiou. netmeta: Network Meta-Analysis using Frequentist Methods 2018.

9 Kay SR, Fiszbein A, Opler LA. The positive and negative syndrome scale (PANSS) for schizophrenia. *Schizophr.Bull.* 1987; **13:** 261–75.

10 Overall JE, Gorham DR. The Brief Psychiatric Rating Scale. *Psychol.Rep.* 1962; **10:** 790–812.

11 Cipriani A, Furukawa TA, Salanti G, et al. Comparative efficacy and acceptability of 21 antidepressant drugs for the acute treatment of adults with major depressive disorder: a systematic review and network meta-analysis. *The Lancet* 2018; **391:** 1357–66. doi:10.1016/S0140-6736(17)32802-7.

Appendix 20: Further outcomes

20.1 Response to treatment (study defined)

There are several issues with the outcome “Response to treatment (study defined)” that need to be considered:

1. The definitions vary widely. For example, they span between a 20% and 50% reduction of the PANSS/BRPRS total score, or from at least minimally improved to much improved on the CGI. This is in contrast to major depressive disorder where response is almost always defined as at 50% reduction of the Hamilton Depression Scale or the Montgomery Asberg Depression Rating Scale.
2. The BPRS is sometimes rated on a 0-6 and sometimes on a 1-7 item scale, and this is not always indicated.
3. The PANSS/BPRS total score and not response was usually the primary outcome in the included studies. It is therefore possible that sometimes the cutoff for defining response was chosen post-hoc by the original authors to find the most favorable one for the examined antipsychotic compared to the control antipsychotic or placebo.
4. If the 1-7 scaling system was used for the PANSS/BPRS, 30/18 minimum points must be subtracted before calculating percentage PANSS/BPRS reduction. This may not always have been done and would underestimate the response rates.
5. Fewer studies presented data for the outcome “response to treatment” than for “overall efficacy” (usually PANSS/BPRS total score), therefore the evidence base for response is smaller (35115 vs. 42576 participants).

These reasons may explain why the results below are not identical to those of our primary outcome. Moreover, the large credible intervals of some drugs should be considered in interpreting the results.

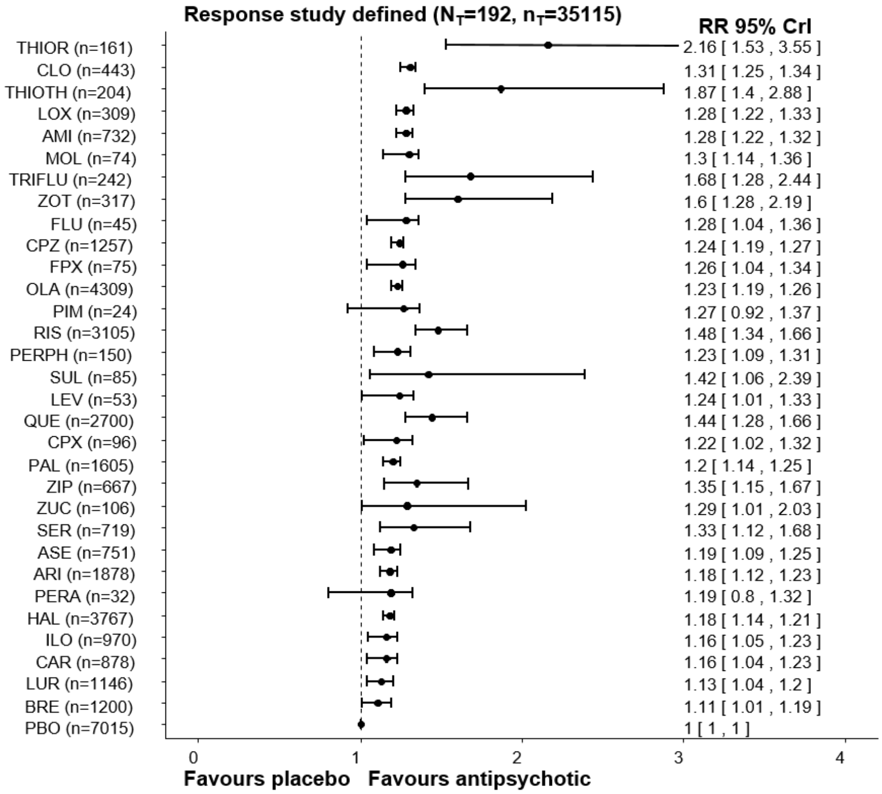

Forest plot for response rates study defined:

Treatments are ranked according to their surface under the curve cumulative ranking (SUCRA) and compared to placebo. Effect sizes are presented as risk ratios (RR with 95% credible intervals (CrI). RR=risk ratio. CrI=credible interval. N_T=total number of trials reporting the outcome. n_T=total number of participants available for the respective outcome. n=number of participants available for each drug for the respective outcome, Drug abbreviations: AMI=Amisulpride, ARI=Aripiprazole, ASE=Asenapine, BRE=Brexpiprazole, CAR=Cariprazine, CPZ=Chlorpromazine, CPX=Clopenthixol, CLO=Clozapine, FPX=Flupenthixol, FLU=Fluphenazine, HAL=Haloperidol, ILO=Iloperidone, LEV=Levomepromazine, LOX=Loxapine, LUR=Lurasidone, MOL=Molindone, OLA=Olanzapine, PAL=Paliperidone, PEN=Penfluridol, PERA=Perazine, PERPH=Perphenazine, PIM=Pimozide, PBO=Placebo, QUE=Quetiapine, RIS=Risperidone, SER=Sertindole, SUL=Sulpiride, THIOR=Thioridazine, THIOTH=Thiothixene, TRIFLU=Trifluoperazine, ZIP=Ziprasidone, ZOT=Zotepine, ZUC=Zuclopenthixol.

20.2 Discontinuation due to inefficacy

Discontinuing a study prematurely (“dropping out”) due to inefficacy is another outcome for overall efficacy. The results are presented in the plot below.

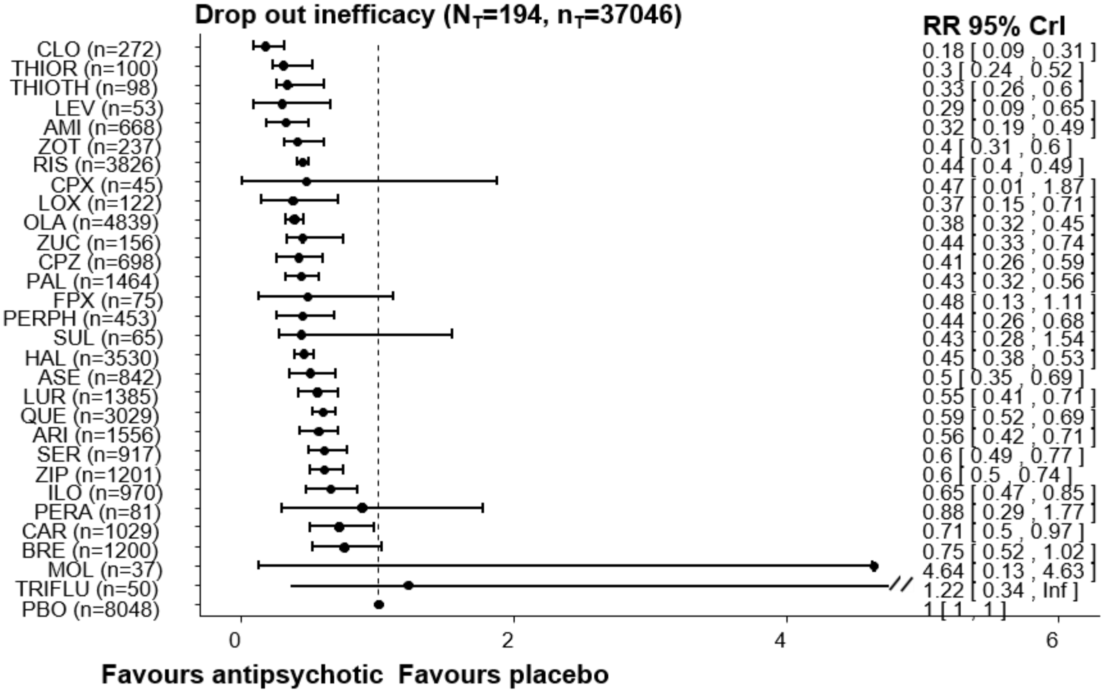

Forest plot for discontinuation due to inefficacy:
Treatments are ranked according to their surface under the curve cumulative ranking (SUCRA) and compared to placebo. Effect sizes are presented as risk ratios (RR with 95% credible intervals (CrI). RR=risk ratio. CrI=credible interval. N_T=total number of trials reporting the outcome. n_T=total number of participants available for the respective outcome. n=number of participants available for each drug for the respective outcome, Drug abbreviations: AMI=Amisulpride, ARI=Aripiprazole, ASE=Asenapine, BRE=Brexpiprazole, CAR=Cariprazine, CPZ=Chlorpromazine, CPX=Clopenthixol, CLO=Clozapine, FPX=Flupenthixol, FLU=Fluphenazine, HAL=Haloperidol, ILO=Iloperidone, LEV=Levomepromazine, LOX=Loxapine, LUR=Lurasidone, MOL=Molindone, OLA=Olanzapine, PAL=Paliperidone, PEN=Penfluridol, PERA=Perazine, PERPH=Perphenazine, PIM=Pimozide, PBO=Placebo, QUE=Quetiapine, RIS=Risperidone, SER=Sertindole, SUL=Sulpiride, THIOR=Thioridazine, THIOTH=Thiothixene, TRIFLU=Trifluoperazine, ZIP=Ziprasidone, ZOT=Zotepine, ZUC=Zuclopenthixol.

20.3 Participants with weight gain ≥7%

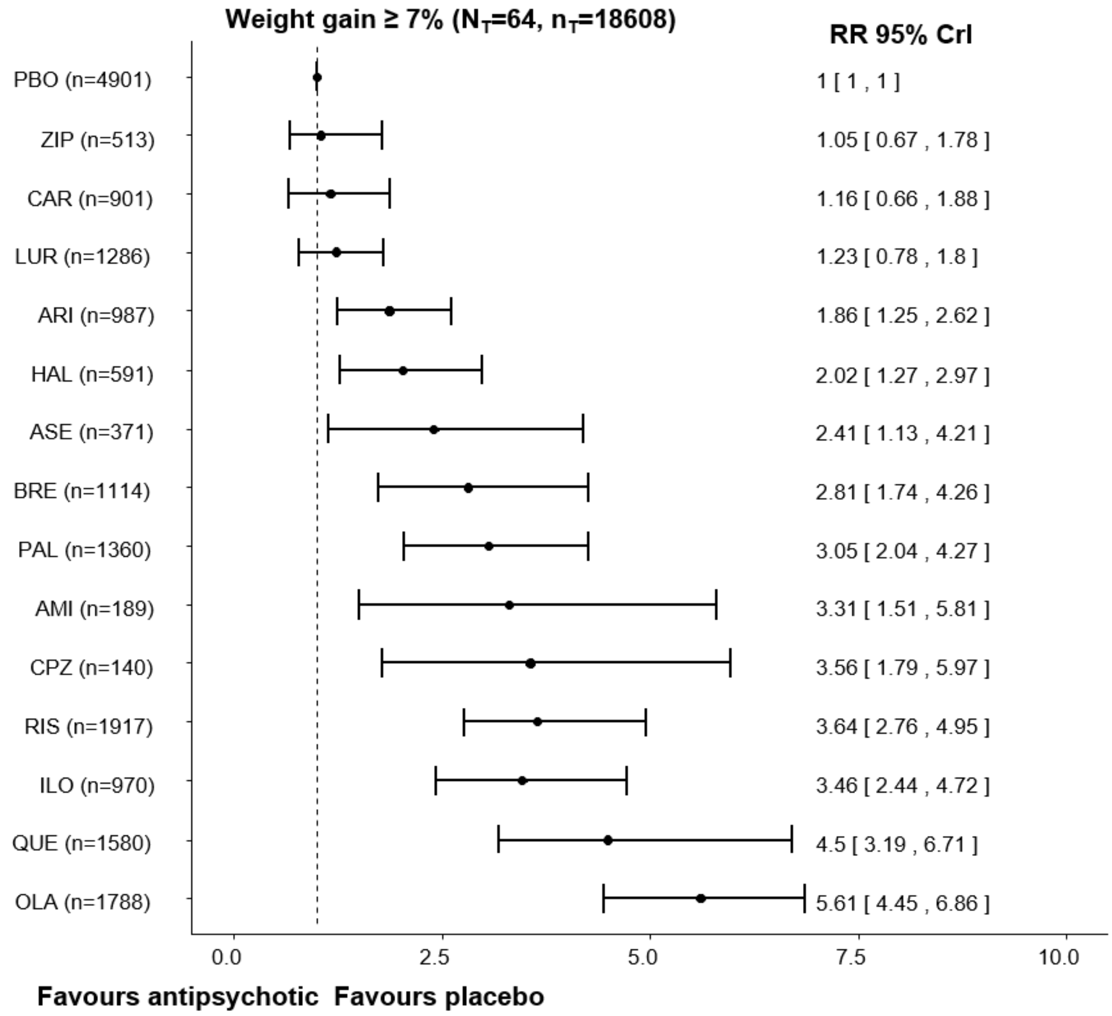

Forest plot for participants with weight gain ≥ 7%:

Treatments are ranked according to their surface under the curve cumulative ranking (SUCRA) and compared to placebo. Effect sizes are presented as risk ratios (RR with 95% credible intervals (CrI). RR=risk ratio. CrI=credible interval. N_T=total number of trials reporting the outcome. n_T=total number of participants available for the respective outcome. n=number of participants available for each drug for the respective outcome, Drug abbreviations: AMI=Amisulpride, ARI=Aripiprazole, ASE=Asenapine, BRE=Brexpiprazole, CAR=Cariprazine, CPZ=Chlorpromazine, CPX=Clopenthixol, CLO=Clozapine, FPX=Flupenthixol, FLU=Fluphenazine, HAL=Haloperidol, ILO=Iloperidone, LEV=Levomepromazine, LOX=Loxapine, LUR=Lurasidone, MOL=Molindone, OLA=Olanzapine, PAL=Paliperidone, PEN=Penfluridol, PERA=Perazine, PERPH=Perphenazine, PIM=Pimozide, PBO=Placebo, QUE=Quetiapine, RIS=Risperidone, SER=Sertindole, SUL=Sulpiride, THIOR=Thioridazine, THIOTH=Thiothixene, TRIFLU=Trifluoperazine, ZIP=Ziprasidone, ZOT=Zotepine, ZUC=Zuclopenthixol.

20.4 Prolactin elevation (SMD results)

We had originally presented all continuous outcomes using standardized mean differences (SMD). A peer-reviewer requested the presentation of weight gain, QTc prolongation and prolactin data in original units as mean difference (MD), because MDs are more readily interpretable for clinicians. Using MDs the prolactin results were clearly more heterogeneous than when SMDs were used as an effect size (Heterogeneity MD: $p<0.001$, SMD $p=0.174$, details appendix 13).

A possible interpretation for the higher heterogeneity of the MD versus the SMD results for prolactin can be that prolactin is a very vulnerable outcome for several reasons:

- It is a female sexual hormone so men and women have very different values, even in healthy populations. But the studies often present the data only pooled for men and women. So if a study included more men than women, the results can differ much from a study in which more women than men were included.
- Baseline imbalances for prolactin are common and can distort endpoint and change values.
- Prolactin is measured with sophisticated laboratory assays, which can differ between different laboratories or different countries.
- Carry over effects from antipsychotic treatment before study start can lead to abnormal prolactin values at baseline.

Standardized mean differences can in part reduce heterogeneity resulting from these issues. We, therefore, present the prolactin results for antipsychotics versus placebo using SMD in addition in the graph below.

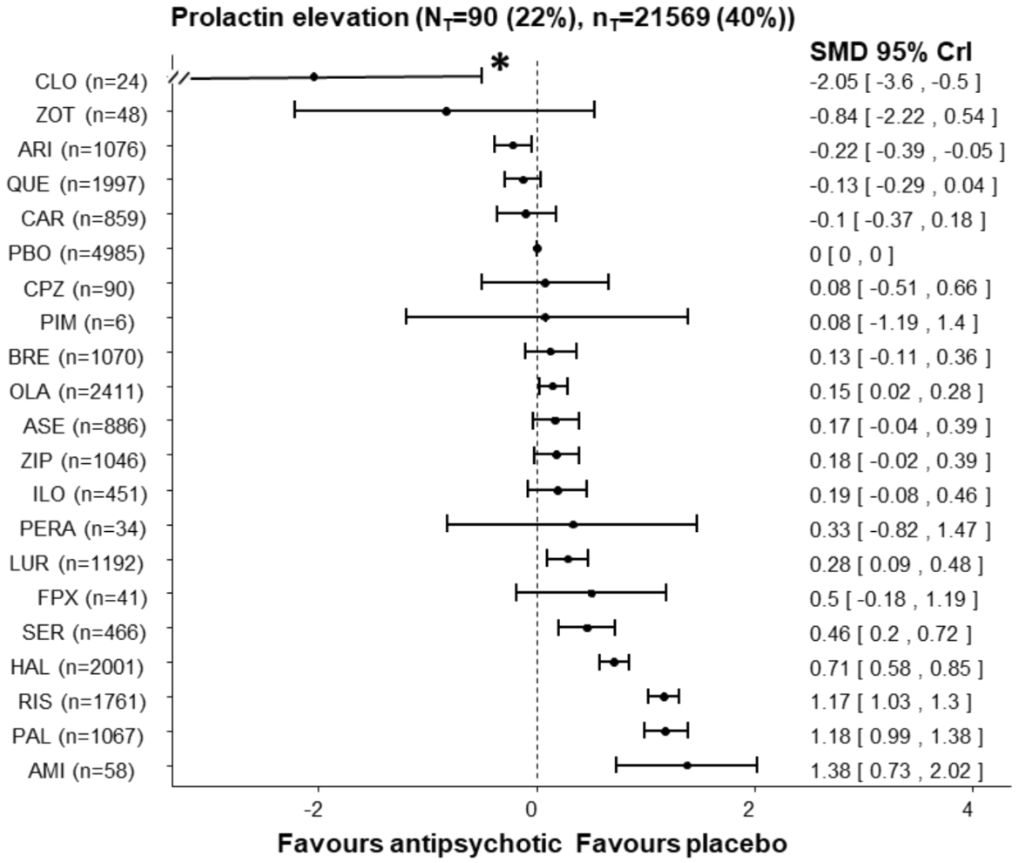

Figure 20.4: Forest plot for prolactin elevation

Treatments are ranked according to their surface under the curve cumulative ranking (SUCRA) and compared to placebo. Effect sizes are presented as standardized mean difference (SMD with 95% credible intervals (CrI)). *=The clozapine and zotepine results may be statistical artifacts driven by two small outlier studies. The wide confidence intervals should also be noted. CrI=credible interval. N_T=total number of trials reporting the outcome. n_T=total number of participants available for the respective outcome. n=number of participants available for each drug for the respective outcome, Drug abbreviations: AMI=Amisulpride, ARI=Aripiprazole, ASE=Asenapine, BRE=Brexpiprazole, CAR=Cariprazine, CPZ=Chlorpromazine, CLO=Clozapine, FPX=Flupenthixol, HAL=Haloperidol, ILO=Iloperidone, LUR=Lurasidone, OLA=Olanzapine, PAL=Paliperidone, PERA=Perazine, PIM=Pimozide, PBO=Placebo, QUE=Quetiapine, RIS=Risperidone, SER=Sertindole, ZIP=Ziprasidone, ZOT=Zotepine.

Appendix 21: Characteristics of the sample

21.1 Descriptives of the overall sample

**Parameter**	
Number of studies	402
Number of participants	53463
Female/male participants	23514 / 29949
Number of comparators	33
Antipsychotic drug (Number of studies/number of participants)	Placebo (151/9522), Olanzapine (80/6913), Haloperidol (128/6134), Risperidone (84/4973), Quetiapine (33/3496), Ziprasidone (26/2443), Aripiprazole (27/2361), Chlorpromazine (76/2297), Iloperidone (8/2239), Paliperidone (12/1675), Lurasidone (7/1385), Brexpiprazole (6/1264), Asenapine (8/1121), Cariprazine (4/1029), Sertindole (7/917), Amisulpride (16/817), Clozapine (31/732), Perphenazine (17/572), Loxapine (24/566), Zotepine (14/469), Trifluoperazine (26/424), Thioridazine (21/399), Sulpiride (11/312), Zuclopenthixol (9/283), Thiothixene (13/227), Molindone (9/165), Perazine (6/158), Pimozide (11/125), Clopenthixol (5/114), Penfluridol (4/103), Flupentixol (4/90), Fluphenazine (7/85), Levomepromazine (2/53)
Mean age (SD)	37.40 (5.96)
Mean duration of illness in years (SD)	11.90 (5.19)
Mean trial duration in weeks (SD)	7.07 (3.08)
Overall dropout rate	35.0%
Blinding (d/s/o)	337 / 21 / 44
<u>Diagnostic criteria:</u><br>Clinical diagnosis/<br>DSM-IV/DSM-III/<br>ICD-9/ICD-10/<br>Other/DSM-V	155/<br>149/57/<br>20/14/<br>5/2
Pharma sponsored	196
Number of studies including patients with schizoaffective disorder	58

Table 21: Descriptives of the sample
SD=standard deviation, d=double, s=single, o=open

21.2 Descriptives of the overall sample separated by antipsychotics

Drug	N (s)	n (s)	Male (s)	Male (%)	Age (m)	Duration ill in years (m)	Duration in weeks (m)	Publication year (m)	Dose (m)	Olanzapine equivalent (m)	Doubleblind (%)	High risk of bias (%)	op diagnostic criteria (%)	Placebo controls (%)	Pharma sponsored (%)
Amisulpride	16	817	463	56.67	36.03	8.92	6.42	1998	602.21	17.47	75.00	31.25	68.75	0	83.33
Aripiprazole	27	2361	1538	65.14	37.05	14.26	5.85	2009	18.65	12.52	62.96	40.74	74.07	37.04	65.38
Asenapine	8	1121	647	57.72	40.33	12.50	8.25	2012	13.31	13.31	75.00	12.50	62.50	75.00	85.71
Brexpiprazole	6	1264	763	60.36	40.15	15.06	6.00	2016	3.01	15.06	83.33	16.67	100	83.33	100
Cariprazine	4	1029	725	70.46	37.93	14.25	6.00	2013	4.14	13.79	100	0	75.00	100	100
Chlorpromazine	76	2297	906	39.44	35.57	10.41	7.24	1976	520.94	17.19	90.79	26.32	40.79	50	8.62
Clopenthixol	5	114	37	32.46	39.38	8.70	7.40	1971	141.90	46.83	100	20	40	40	25.00
Clozapine	31	732	251	34.29	36.26	10.35	7.20	1987	325.32	16.27	80.65	45.16	51.61	6.45	41.67
Flupenthixol	4	90	38	42.22	34.73	5.50	6.50	1992	17.61	35.23	100	0	75.00	25.00	0
Fluphenazine	7	85	49	57.65	42.27	13.05	8.00	1972	9.84	16.40	100	0	28.57	28.57	0
Haloperidol	128	6134	3049	49.71	37.77	12.67	6.93	1994	13.14	26.28	81.25	24.22	68.75	22.66	3.96
Iloperidone	8	2239	690	30.82	37.76	12.68	5.75	2009	15.17	12.64	87.50	12.50	87.50	50	87.50
Levomepromazine	2	53	29	54.72	39.30	NA	4.14	1984	140.13	NA	100	50	100	0	0
Loxapine	24	566	253	44.70	34.97	8.05	8.04	1976	80.88	26.69	95.83	16.67	25.00	25.00	57.89
Lurasidone	7	1385	971	70.11	39.50	13.48	6.00	2011	85.19	10.65	100	0	85.71	100	100
Molindone	9	165	51	30.91	39.90	17.50	9.22	1974	84.24	16.85	100	22.22	33.33	11.11	50
Olanzapine	80	6913	4155	60.10	37.44	11.63	7.28	2007	14.41	14.41	71.25	31.25	76.25	26.25	30.43
Paliperidone	12	1675	900	53.73	37.98	12.88	7.50	2009	7.99	17.73	75.00	25.00	66.67	58.33	81.82
Penfluridol	4	103	65	63.11	31.00	NA	8.25	1979	50	16.50	100	50	50	25.00	0
Perazine	6	158	63	39.87	34.50	5.75	5.17	1993	458.67	15.14	83.33	33.33	50	0	0
Perphenazine	17	572	338	59.09	39.36	7.25	7.24	1979	38.77	26.02	94.12	17.65	64.71	11.76	0
Pimozide	11	125	55	44.00	43.77	16.43	6.73	1976	13.07	32.66	72.73	0	45.45	9.09	11.11
Placebo	151	9522	5912	62.09	38.39	13.21	6.48	1992	0	NA	97.35	11.26	70.86	100	0
Quetiapine	33	3496	2262	64.70	38.41	13.78	6.91	2005	519.52	14.03	63.64	33.33	72.73	30.30	64.29
Risperidone	84	4973	3202	64.39	36.55	11.37	7.26	2005	5.82	19.39	66.67	32.14	71.43	21.43	30.30
Sertindole	7	917	616	67.18	37.30	13.20	8.53	2002	17.16	17.16	100	0	57.14	42.86	100
Sulpiride	11	312	86	27.56	36.60	NA	7.03	1982	935.80	23.39	72.73	36.36	36.36	9.09	20

Thioridazine	21	399	120	30.08	36.50	10.07	7.29	1976	435.45	17.42	95.24	19.05	38.10	42.86	21.43
Thiothixene	13	227	105	46.26	32.46	12.80	7.01	1976	33.73	22.60	100	15.38	23.08	23.08	16.67
Trifluoperazine	26	424	166	39.15	38.78	14.10	8.53	1972	31.44	31.44	88.46	23.08	34.62	19.23	4.76
Ziprasidone	26	2443	1132	46.34	36.06	12.56	8.28	2006	127.30	15.91	61.54	23.08	80.77	15.38	75.00
Zotepine	14	469	160	34.12	36.14	9.08	6.34	1995	237.51	15.91	85.71	21.43	64.29	7.14	100
Zuclopenthixol	9	283	152	53.71	37.41	15.45	8.65	1992	44.06	17.62	66.67	22.22	55.56	0	25.00
**All**	**887**	**53463**	**29949**	**56.02**	**37.40**	**11.90**	**7.07**	**1992**	**152.92**	**19.82**	**85.66**	**21.60**	**59.84**	**33.21**	**42.12**

Table 2: Descriptives of the sample separated by antipsychotics
N=number of studies, n=number of participants, SD=standard deviation, NA=not available, %=percentage, m=mean, s=sum, op=operationalized.

Appendix 22: Funnel plots

Figure 22a:

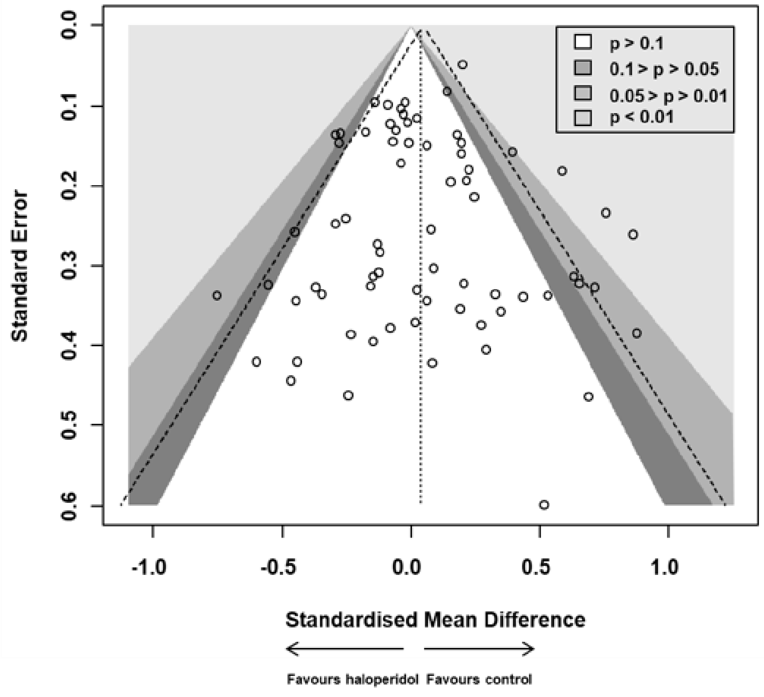

Figure 22b:

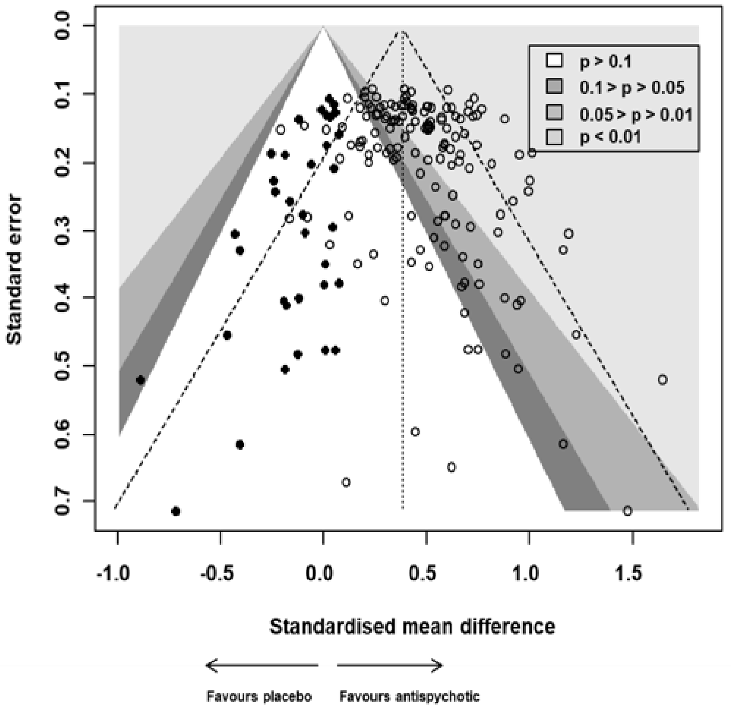

Figure 22a: Contour-enhanced funnel plot for all antipsychotics compared to haloperidol
Change in overall symptoms of all antipsychotics compared to haloperidol. Circles represent effect sizes of individual studies measured as standardized mean difference.

Figure 22b: Contour-enhanced funnel plot for all antipsychotics compared to placebo
Change in overall symptoms of all antipsychotics compared to placebo. Circles represent effect sizes of individual studies measured as standardized mean difference. Missing studies were estimated using the trim and fill method. Original effect sizes are represented by empty circles and estimated ones by filled circles.

23. References

1 Higgins JPT, Green S. Cochrane Handbook for Systematic Reviews of Interventions Version 5.1.0 [updated March 2011]. Chichester, UK: Wiley and Sons, 2011.

2 Furukawa TA, Barbui C, Cipriani A, Brambilla P, Watanabe N. Imputing missing standard deviations in meta-analyses can provide accurate results. *Journal of clinical epidemiology* 2006; **59:** 7–10. doi:10.1016/j.jclinepi.2005.06.006.

3 Plummer M, Stukalov A, Denwood M. Rjags: Bayesian Graphical Models using MCMC (version 4-6) 2016. https://CRAN.R-project.org/package=rjags.

4 Leucht S, Leucht C, Huhn M, et al. Sixty Years of Placebo-Controlled Antipsychotic Drug Trials in Acute Schizophrenia. Systematic Review, Bayesian Meta-Analysis, and Meta-Regression of Efficacy Predictors. *The American journal of psychiatry* 2017: appiajp201716121358. doi:10.1176/appi.ajp.2017.16121358.

5 Leucht S, Rothe P, Davis JM, Engel RR. Equipercentile linking of the BPRS and PANSS. *European Neuropsychopharmacology* 2012; **in press**.

6 Gardner DM, Murphy AL, O'Donnell H, Centorrino F, Baldessarini RJ. International consensus study of antipsychotic dosing. *The American journal of psychiatry* 2010; **167:** 686–93. doi:10.1176/appi.ajp.2009.09060802.

7 Rhodes KM, Turner RM, Higgins JPT. Predictive distributions were developed for the extent of heterogeneity in meta-analyses of continuous outcome data. *Journal of clinical epidemiology* 2015; **68:** 52–60. doi:10.1016/j.jclinepi.2014.08.012.

8 Gerta Rücker, Guido Schwarzer, Ulrike Krahn, Jochem König, Orestis Efthimiou. netmeta: Network Meta-Analysis using Frequentist Methods 2018.

9 Kay SR, Fiszbein A, Opler LA. The positive and negative syndrome scale (PANSS) for schizophrenia. *Schizophr.Bull.* 1987; **13:** 261–75.

10 Overall JE, Gorham DR. The Brief Psychiatric Rating Scale. *Psychol.Rep.* 1962; **10:** 790–812.

11 Cipriani A, Furukawa TA, Salanti G, et al. Comparative efficacy and acceptability of 21 antidepressant drugs for the acute treatment of adults with major depressive disorder: a systematic review and network meta-analysis. *The Lancet* 2018; **391:** 1357–66. doi:10.1016/S0140-6736(17)32802-7.
